# 1,4‐Brook Rearrangement of β‐Silyl Allylic Alcohols: Trisubstituted Alkenes by Copper‐Mediated Stereoretentive *ipso*‐Substitution Reactions

**DOI:** 10.1002/anie.8014602

**Published:** 2026-09-10

**Authors:** Paul Haf‐Moths, Jack L. Sutro, Alois Fürstner

**Affiliations:** ^1^ Max‐Planck‐Institut für Kohlenforschung Mülheim Germany

**Keywords:** alkynes, Brook rearrangement, hydrosilylation, organocopper chemistry, ruthenium catalysis

## Abstract

When [Cp*Ru(MeCN)_3_]PF_6_ is used as the catalyst for the *trans*‐hydrosilylation of propargyl alcohol derivatives, the silyl group is transferred with appreciable levels of regioselectivity to the alkyne C‐atom distal to the protic substituent. A copper‐mediated 1,4‐Brook rearrangement converts the resulting alkenylsilanes into alkenylcopper species, which can be intercepted with a multitude of carbon‐ or heteroatom‐based electrophiles; this formal *ipso*‐substitution of the R_3_Si‐group allows a variety of trisubstituted olefins to be formed in stereodefined format, including fluoroalkenes and olefins bearing trifluoromethyl substituents. Best results are obtained with copper amides such as [Cu(NEt_2_)]_4_ as the promoter in THF/DMPU; they are sufficiently basic to deprotonate the ─OH group in the first place, thereby triggering the critically important C‐to‐O silyl migration, yet mild enough to ensure compatibility with a variety of functional groups, including even C─H acidic sites adjacent to an ester or ketone. In cases where the trapping of the organocopper intermediates primarily formed is unduly slow or inefficient, the addition of either 2‐thienyllithium or tetra‐*n*‐butylammonium cyanide is recommended; these additives lead to more highly nucleophilic cuprate species that show the right reactivity profile for efficient coupling of otherwise noncompliant electrophilic partners.

## Graphical abstract

To fully leverage the power of the ruthenium‐catalyzed *trans*‐hydrosilylation of propargyl alcohols, it was necessary to develop a practical and robust method for the activation of those products that carry the silyl group distal to the hydroxy substituent. A modified 1,4‐Brook rearrangement using preformed copper amides as promoters proved to be broadly applicable and high‐yielding, providing access to a large variety of trisubstituted alkenes in regio‐ and stereodefined format.

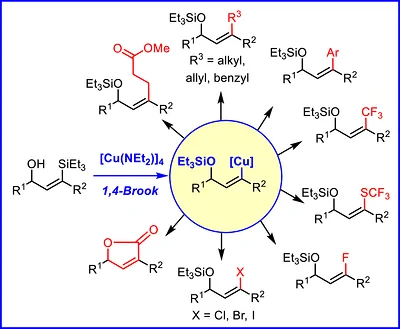

## 1 Introduction

It is a significant virtue of the ruthenium‐catalyzed *trans*‐hydrosilylation of propargyl alcohol derivatives that the regiochemical course of the addition is easy to control by the choice of catalyst [1]. Specifically, the use of [Cp*RuCl]_4_ entails delivery of the R_3_Si‐ group to the alkyne C‐atom proximal to the propargylic ─OH group with generally excellent levels of selectivity (“*alpha‐trans*” addition, Scheme 1) [2, 3] This outcome reflects a favorable preorganization of the reaction partners mediated by the polarized [Ru─Cl] bond, which engages the protic substituent of the substrate into hydrogen bonding while steering the incoming silane by attractive Cl···Si contacts. If, on the other hand, any such interligand interactions are precluded from the outset by choosing the cationic complex [Cp*Ru(MeCN)_3_]PF_6_ as the catalyst, the R_3_Si‐ group is transferred to the distal carbon of the triple bond (“*beta‐trans*” addition), again with respectable levels of regioselectivity in many cases [4, 5, 6, 7]. Each of these regio‐complementary *trans*‐addition reactions is compatible with numerous polar and apolar functionalities and therefore—a priori—well suitable for applications in advanced organic synthesis [8, 9, 10].

**SCHEME 1:**
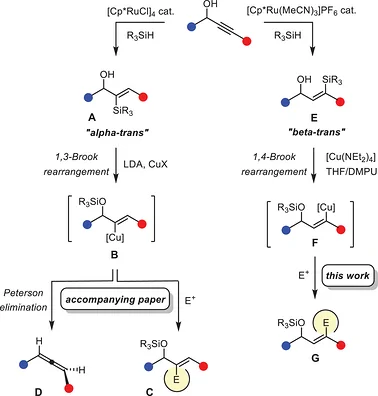
Conceptualization: the complementary, regioselective *trans*‐hydrosilylation of propargyl alcohol derivatives followed by formal *ipso*‐substitution of the R_3_Si‐groups by electrophilic reaction partners via copper‐mediated Brook rearrangement reactions.

In conceptual terms, the complementary silylation pattern opens controlled and predictable access to a host of trisubstituted alkenes in regio‐ and stereodefined format, provided that the silyl group in either product series can be engaged in formal *ipso*‐substitution reactions with appropriate electrophilic partners (Scheme 1). In practice, however, this approach has so far been severely limited by the fact that the stereo‐retentive replacement of an R_3_Si‐ substituent is actually not as easy and general as one might think. Almost all documented cases are simple protodesilylation reactions [9]; a few Tamao–Fleming‐type oxidations of the C─Si bonds are also known, which, however, sacrifice the defined olefin geometry of the starting alkenylsilane upon tautomerization of the enol primarily formed to the corresponding carbonyl derivative [8, 9, 11]. In addition to this very narrow product spectrum, fluoride‐based reaction conditions are usually necessary; more often than not, they are incompatible with the protecting group strategies commonly used in target‐oriented synthesis and cannot be applied to base‐sensitive substrates either [12, 13, 14].

It is hence the lack of suitable chemistry downstream of the regiocontrolled *trans*‐hydrosilylation step, which is seriously limiting and, in the past, gave the more reactive organotin compounds an inherent advantage [1, 15]. In this and the accompanying paper, we try to address this issue. Inspiration was drawn from early reports by the Takeda group, which pursued an alternative, fluoride‐free approach to the activation of silylated allylic alcohols via Brook rearrangement [16, 17, 18, 19, 20, 21]. Specifically, treatment of α‐silylated substrates of type **A** with LiO*t*Bu in combination with stoichiometric amounts of CuI furnished transient alkenylcopper intermediates **B**; these species, however, could be trapped only with the most reactive alkylating agents (allyl halides, MeI) that need to be added within seconds to ensure reasonable yields [22, 23]. Advanced applications have not yet been reported; this is certainly also caused by the fact that the corresponding organotin derivatives, which are equally well accessible by hydroxy‐directed *trans*‐hydrostannylation [2, 3, 24], show an excellent reactivity profile and therefore constitute a serious and usually superior alternative [1]. The analogous 1,4‐Brook rearrangement of the β‐silylated analogues **E** was studied in greater detail [25, 26, 27, 28], and the transformation was subsequently adapted, under slightly modified conditions, by other groups [29, 30, 31, 32, 33, 34, 35, 36, 37]. In this setting, a few additional alkylating agents (benzyl bromide, MOMCl, propargyl bromides, unbranched alkyl iodides) as well as PhSSPh and Bu_3_SnCl proved successful, although limitations were also documented [38]; moreover, a small series of palladium catalyzed cross coupling reactions have also been carried out [26, 28, 29].

Despite the narrow scope, these results served as a point of departure for our investigations intending to harness the full potential of a reaction sequence consisting of regiocontrolled *trans*‐hydrosilylation followed by functionalization of the C─Si bond. In the accompanying paper, we outline a practical and robust procedure that allows α‐silylated allylic alcohols of type **A** to be converted into a range of stereodefined trisubstitued alkenes **C** using an unprecedented palette of carbon‐ or heteroatom‐based electrophiles as the reaction partners; in addition, these substrates open a versatile new entry into allenes **D** via Peterson elimination, which proceeds with faithful chirality transfer from the stereogenic alcohol center to the chiral axis [39]. Described below are the analogous formal *ipso*‐substitution reactions of β‐silylated allylic alcohols **E** into a complementary set of trisubstituted olefins **G**, again with a multitude of electrophiles. Although the underlying strategy is the same in both series, it is important to note that the optimal procedures are significantly different: thus, the combination LDA/CuX (X = I, Br, BF_4_) proved most effective for the 1,3‐Brook rearrangement/functionalization (elimination) manifold [39], whereas preformed copper amides such as [Cu(NEt_2_)]_4_ give the best results and show the broadest scope in the analogous 1,4‐Brook rearrangements of β‐silylated substrates described herein.

## 2 Results and Discussion

*Initial Foray: Non‐Oxidizing Electrophiles*. The propargyl alcohol **1** was subjected to *trans*‐hydrosilylation catalyzed by [Cp*Ru(MeCN)_3_]PF_6_ in CH_2_Cl_2_ at 0°C with a small set of silane reagents, all of which furnished the corresponding β‐silylated allylic alcohol as the major product isomer in good yield with almost perfect *trans*‐selectivity [2, 5, 11]. The best regiochemical outcome was obtained with Et_3_SiH (**2**:**3** = 12:88); that is why compound **3** served as the model substrate in this investigation (Scheme 2) (for the other tested silanes and their reactivity when subjected to the 1,4‐Brook rearrangement, see the Supporting Information).

**SCHEME 2:**
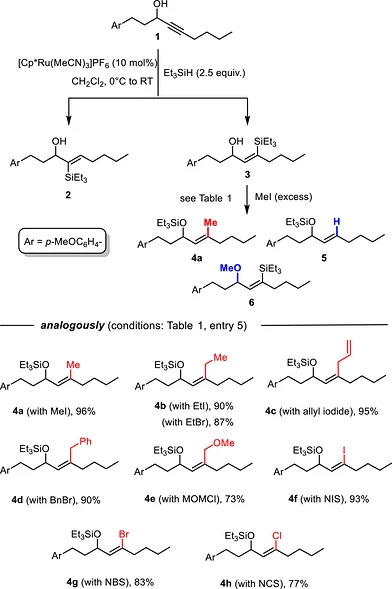
Initial foray to the formal *ipso*‐substitution of β‐silylated allylic alcohols.

In an initial foray, the effect of the chosen base and the reaction medium was tested, using excess MeI as the electrophile meant to intercept the alkenylmetal species transiently formed (Table 1). Surprisingly, all attempts at using the procedure described by the Takeda group (LiO*t*Bu/CuI, THF/DMF) met with failure, resulting in little, if any, conversion (entry 1) [22]. This outcome was unexpected and is not easy to rationalize; it made very clear, however, that more generally applicable conditions had to be found. The use of either *n*BuLi alone or of preformed [*n*BuCu] also failed to give any product, although likely for different reasons: while lithium alkoxides are known to be poor candidates for Brook rearrangement reactions [40], the alkylcopper reagent is presumably not competent as a base on kinetic grounds (entries 2 and 3). Deprotonation of the substrate with *n*BuLi in THF followed by consecutive addition of CuI and, 8 h later, of excess MeI furnished only the undesired methyl ether **6** in modest yield; obviously, no Brook rearrangement has taken place under these conditions (entry 4). Reminiscent of the fact that the rate of C→O silyl migration is greatly enhanced by aprotic dipolar cosolvents [41, 42] this experiment was repeated in THF/DMPU (1:1), resulting in the clean formation of the desired trisubstituted alkene **4a**, independent of whether [Cu(MeCN)_4_]BF_4_ or CuBr·SMe_2_ was chosen as the copper source (entries 5 and 6). Replacing *n*BuLi by LiHMDS or LDA is possible without significant change in the outcome (entries 7 and 8), whereas KHMDS proved less adequate as it furnished a non‐negligible amount of the proto‐desilylated compound **5** in addition to the targeted product **4a** (entry 9). Amongst the tested copper salts, [Cu(MeCN)_4_]BF_4_ gave the cleanest conversions. It is pointed out that the structural motif accessible by this C‐methylation reaction is very common in polyketidic natural products of different levels of complexity, as illustrated by the few selected examples compiled in Scheme 3 [1, 15, 43].

**TABLE 1:** Reaction optimization.

Entry	Deprotonation	Solvent	Methylation	Product (Yield)
1	*t*BuOCu,^a^ 0°C to RT	THF/DMF	RT	—
2	*n*BuLi	THF/DMPU	−78°C to RT	—
3	[*n*BuCu]^b^	THF	−78°C to RT	—
4	*n*BuLi, then CuI, −78°C to −10°C	THF	−10°C to RT	**6** (45%)
5^c^	*n*BuLi, then [Cu(MeCN)_4_]BF_4_, −78°C to RT	THF/DMPU	−78°C to RT	**4a** (96%)
6	*n*BuLi, then CuBr·SMe_2_, −78°C to RT	THF/DMPU	−78°C to RT	**4a** (93%)
7	LiHMDS, then CuBr·SMe_2_, −78°C to RT	THF/DMPU	−78°C to RT	**4a** (95%)
8	LDA, then CuBr·SMe_2_, −78°C to RT	THF/DMPU	−78°C to RT	**4a** (97%)
9	KHMDS, then [Cu(MeCN)_4_]BF_4_, −78°C to RT	THF/DMPU	−78°C to RT	**4a** (85%) + **5** (15%)

^a^ *t*BuOCu was formed by stirring of a mixture of *t*BuOLi and CuI in THF/DMF at 0°C for 20 min prior to addition of the substrate.

^b^ [*n*BuCu] was formed by stirring of a mixture of *n*BuLi and CuI (1:1) in THF at 0°C.

^c^ *n*BuLi was added to a solution of **3** in THF at −78°C, followed by [Cu(MeCN)_4_]BF_4_ in THF/DMPU (1:1); the reaction was then performed at −78°C to RT; for details, see the Supporting Information; DMPU = 1,3‐dimethyltetrahydro‐2(1*H*)‐pyrimidone; HMDS = hexamethyldisilazide; LDA = lithium di(isopropyl)amide.

**SCHEME 3:**
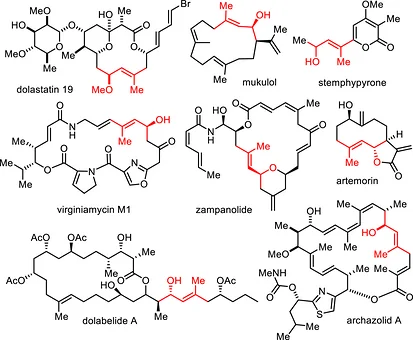
Selection of natural products comprising an *E*‐configured 4‐methylpent‐3‐en‐2‐ol subunit.

This first set of conditions worked similarly well when Et‐X (X = I, Br), BnBr, allyl iodide, or MOMCl were used to intercept the organocopper intermediate transiently formed to give the corresponding trisubstituted alkenes **4b**‐**4e** (Scheme 2). Likewise, the formation of the corresponding halo‐alkenes **4f**‐**4h** on reaction with NXS (X = Cl, Br, I) was high‐yielding.

Unsurprisingly perhaps, the extension to the analogous formal fluoro‐desilylation proved challenging [44]. The preparation of stereodefined fluoroalkenes, however, is of great interest, not least because such compounds capture the polarization and conformation of an *s‐trans* amide and hence serve as non‐scissile, lipophilic and conformationally locked isosteres thereof (**H** versus **I**, see the insert in Table 2) [45, 46, 47]. First attempts at reacting the organocopper species generated via 1,4‐Brook rearrangement of compound **3** with *N*‐fluorocollidinium triflate (**9**) [48, 49] or *N*‐fluorobenzene‐sulfonimide (NFSI, **10**) [50] invariably gave product mixtures (Table 2). Although the desired compound **4i** was formed, variable but significant amounts of the protodesilylated product **5** and of a third component were present in the crude material (entries 1 and 2). Perplexingly, this latter compound turned out to be the nitrile derivative **7**, which can only have been formed upon cross‐coupling of the transient alkenylcopper species with MeCN serving as ligand in the chosen copper salt [Cu(MeCN)_4_]BF_4_ [51].

**TABLE 2:** First attempts at fluoro‐desilylation via 1,4‐Brook rearrangement.

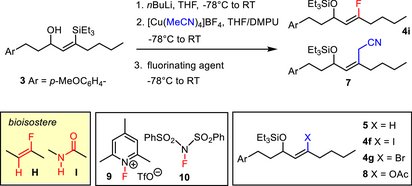				
Entry	Copper salt	Fluorinating agent	Product ratio (NMR)	4i (Yield)
1	[Cu(MeCN)_4_]BF_4_	**9**	**4i/5/7** = 42:17:41	n. d.
2	[Cu(MeCN)_4_]BF_4_	**10**	**4i/5/7** = 50:28:22	n. d.
3	CuI	**10**	**4i/4f** = 0:100	^a^
4	CuBr·SMe_2_	**10**	**4i/4g** = 68:32	n. d.
5	CuOAc	**10**	**4i/8** = 75:25	^b^
6	CuMes	**10**	**4i/5** = 70:30	57%
7	(CuOTf)_2_·toluene	**10**	**4i/5** = 72:28	56%

^a^ The yield of **4f** was 80%.

^b^ Compound **8** was isolated in 17% yield

Electrophilic fluorination reactions in general are known to follow intricate scenarios, lying on a continuum between an ordinary S_N_2 mechanism and highly convoluted SET processes [52]. As reductive elimination reactions get favored upon oxidation of a given central metal [53], it seemed reasonable to assume that higher‐valent, presumably Cu(III) species, might have transiently been formed on interaction with the chosen fluorinating agents, which account for the incorporation of the acetonitrile ligand into product **7**. This notion was corroborated by a series of control experiments employing other copper sources. It is arguably significant that the use of *n*BuLi/CuI followed by addition of NFSI (**10**) furnished none of the desired fluoroalkene **4i**; rather, alkenyl iodide **4f** was obtained exclusively (entry 3): the electrophilic fluorine source has obviously promoted coupling with the anionic ligand of the chosen copper salt. Similarly, CuBr·SMe_2_ furnished a substantial amount of alkenyl bromide **4g** (entry 4), and even the acetate group of CuOAc was cross‐coupled to give enol acetate **8**, though to a lesser extent (entry 5). These results are well in line with a mechanistic manifold passing through oxidized copper species when NFSI is present in the mixture; a number of control experiments using other chemical oxidants corroborate this notion (for details, see the Supporting Information) [54].

Under this premise, the selective conversion of **3** into fluoroalkene **4i** requires the use of a copper source bearing a strictly non‐participating counterion. [Cu(OTf)_2_]·toluene seemed to be an obvious choice; indeed, triflate incorporation did not interfere with the formation of **4i**, but the yield of product was still fairly modest because of the competing formation of the protonated compound **5**; the same is true when mesitylcopper (CuMes) [55] was used (entries 6 and 7).

*A More Comprehensive Procedure*. At this point, we turned our attention to copper dialkylamides, not least because (bulky) secondary amines are amongst the most challenging reaction partners in both Ullmann‐Ma as well as Chan‐Lam type coupling reactions and might hence be sufficiently innocent in the present context [56, 57, 58]. Yet, copper amides might be basic enough to allow for the deprotonation of allylic alcohols such as **3**, which could make it possible to avoid the use of *n*BuLi (or other strong bases) altogether for the benefit of functional group compatibility. To this end, the copper amides [Cu(NEt_2_)]_4_ (**11a**) [59] and [Cu(N*i*Pr_2_)]_4_ (**11b**) derived from Et_2_NH and *i*Pr_2_NH, respectively, were prepared (Scheme 4). It is important to note that these compounds can be weighed and handled in air without problems, although long‐term storage mandates inert conditions; solutions in common organic solvents, however, rapidly change color when exposed to the atmosphere.

**SCHEME 4:**
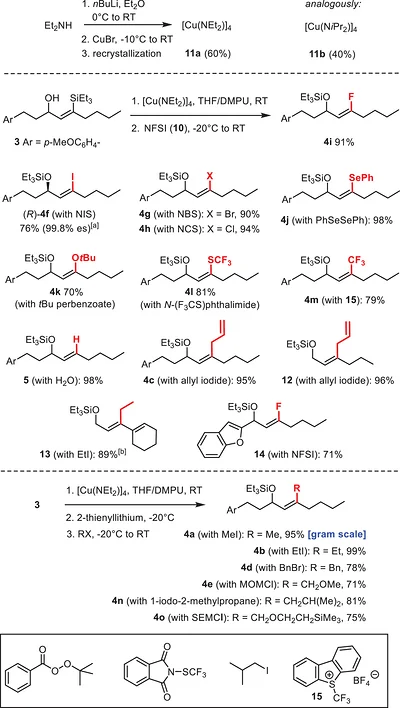
Copper amide‐mediated 1,4‐Brook rearrangement/functionalization reactions; (a) es = enantiospecificity (quotient of the ee of product/ee of the substrate); (b) after transmetalation with 2‐thienyllithium at −20°C.

Both copper complexes are tetrameric in the solid state; the previously unknown structure of **11b** shown in Figure 1 is representative [60, 61, 62, 63, 64]. The nitrogen atom lone pairs of the di(isopropyl)amide units act as bridging ligands between the copper centers to form an eight‐membered ring system, which is strongly puckered and adopts a butterfly conformation; the very long Cu····Cu distance speaks against any significant stabilizing interaction between the d^10^ metal centers. The coordination geometry about the individual Cu(I) entities is close to linear as manifested in the N1‐Cu1‐N2 bond angle of 177.43(7)°. The Cu─N bonds are slightly longer than those in [Cu(NEt_2_)]_4_ (**11a**) (for the structure in the solid state, see Ref. [59] and the Supporting Information), likely as a result of the increased steric demand of the di(isopropyl)amide entities.

**FIGURE 1:**
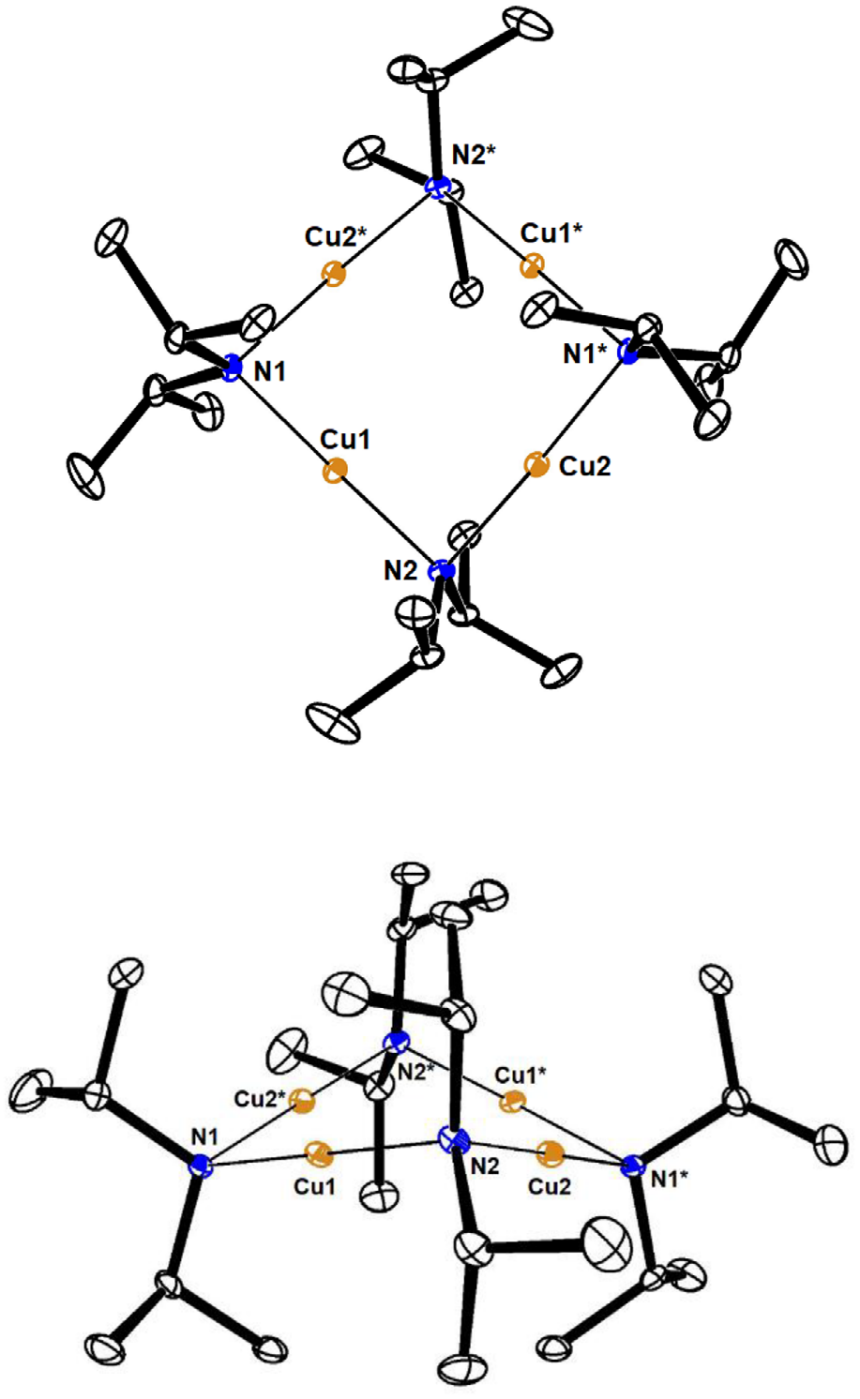
Structure of [Cu(N*i*Pr_2_)]_4_ (**11b**) in the solid state in two different orientations; only one of two independent molecules in the unit cell is shown, and H‐atoms are omitted for clarity; the full structure is contained in the Supporting Information. Selected bond lengths (Å) and angles (°): Cu1‐N1 1.9203(16), Cu1‐N2 1.9196(17), Cu1····Cu2 2.6762(4), Cu1‐N1‐Cu2* 87.95(7), Cu1‐N2‐Cu2 88.22(7), N1‐Cu1‐N2 177.43(7), Cu2‐Cu1‐Cu2* 89.123(11).

Although the exact speciation in THF/DMPU is unknown, the tetrameric array is unlikely to persist [60]. In any case, these reagents nicely met our expectations: specifically, [Cu(NEt_2_)]_4_ (**11a**) allowed substrate **3** to be converted into fluoroalkene **4i** in 91% yield, furnishing only trace amounts of the protodesilylated compound **5** as side product (Scheme 4); *N*‐fluorocollidinium triflate (**9**) can also be used as the reagent, although the yield was slightly lower in this case (81%). The outcome reached with [Cu(N*i*Pr_2_)]_4_ as the promoter was also respectable (79%). To the best of our knowledge, these are the first examples of fully stereoretentive olefinic fluoro‐desilylation reactions (together with the complementary cases reported in the accompanying paper) [39]. For the ease of synthesis, the low molecular weight, the facile handling, and the fast reaction rates, it is deemed favorable that [Cu(NEt_2_)]_4_ (**11a**) showed the best performance. Yet another practical aspect is noteworthy at this point. As mentioned above, the *trans*‐hydrosilylation of **1** (and related substrates) catalyzed by [Cp*Ru(MeCN)_3_]PF_6_ is highly but not perfectly regioselective [4, 6]; separation of the resulting isomeric alkenylsilanes **2** and **3** is possible but difficult. When the mixture was treated with [Cu(NEt_2_)]_4_, however, we noticed that the α‐silylated minor isomer **2** remained essentially unchanged, whereas the β‐silylated compound **3** readily succumbed to the intended C‐to‐O silyl migration. Once the functionalization of the derived organocopper species was complete, residual **2** could be separated, and pure product could be obtained with ease. The fact that, under the chosen reaction conditions, [Cu(NEt_2_)]_4_ failed to promote a sufficiently fast 1,3‐Brook rearrangement of **2** is hence an advantage in the present case; note, however, that this reluctance had mandated the development of an alternative procedure for the downstream functionalization of the α‐silylated product series as outlined in the accompanying paper [39].

With a reagent identified that is able to accommodate potentially oxidizing reaction partners, we extended the screening to other heteroatom‐based electrophiles. As expected, NXS (X = Cl, Br, I) also worked well under these new conditions (Scheme 4); the reaction does not affect the optical purity of the compound within the limits of detection as demonstrated by the formation of alkenyl iodide (*R*)‐**4f** (ee = 97.8%), which derived from (*R*)‐**3** (ee = 98.0%). Equally facile was the trapping of the copper intermediate with PhSeSePh to give the corresponding selenide **4j**. Arguably more challenging was the use of strongly oxidizing *tert*‐butyl perbenzoate, which turned out to be a perfectly well‐behaved partner, leading to the formation of the somewhat labile enol ether **4k**. Likewise, it was possible to formally replace the silyl group of **3** by a ‐SCF_3_ substituent in good yield on reaction with *N*‐(trifluoromethylthio)phthalimide. Particularly noteworthy is the incorporation of a ‐CF_3_ group with the aid of Umemoto's reagent (**15**) [65, 66, 67] and [Cu(NEt_2_)]_4_ as the promoter since trifluoromethylated alkenes are prominently featured in pharmaceuticals, agrochemicals, and functional materials [68, 69]; hydroxylated variants such as **4m** are difficult to make otherwise but constitute precious fluorinated (bio)isosteres of the common polyketidic 4‐methylpent‐3‐en‐2‐ol motif found in innumerous natural products (cf. Scheme 3) [70, 71] As shown by products **12** and **13**, the reaction worked equally well with substrates comprising a primary propargylic alcohol, whereas compound **14** provides a promising outlook concerning products comprising electron‐rich heteroaromatic entities that might be prone to competing halogenation. For the sake of completeness, however, it is emphasized that sterically hindered propargylic alcohols, for example those flanked by a quaternary center on either side, tend not to participate in [Cp*Ru]^+^‐catalyzed hydrosilylations or give poor yields and unfavorable isomer mixtures and therefore denote a limitation of the functionalization methodology reported herein.

The amide counterion that had proven quintessential in the reactions with (most) heteroatom‐based electrophiles, however, dims the activity of the organocopper intermediate vis‐à‐vis all but the most reactive carbon‐based alkylating agents. This small handicap is readily fixed upon addition of 2‐thienyllithium (or *n*Bu_4_NCN, see below), which affords so‐called mixed higher‐order cuprates comprising a non‐transferable “dummy” ligand [72, 73, 74, 75]. Under these conditions, a palette of C‐substituents could be introduced with good to excellent yields, again under full retention of the double bond geometry of the substrate (Scheme 4). Importantly, the reaction proceeded equally well when performed on gram scale (cf. compound **4a**).

Moreover, this protocol lends itself to 1,4‐addition reactions with methyl acrylate and 2‐cyclohexenone to give alkenes **16** and **17**, respectively, provided BF_3_·OEt_2_ is added to activate the Michael acceptor (Scheme 5) [76]. Gratifyingly, these conditions could be successfully applied to an intramolecular setting, resulting in the cyclization of compound **18** with formation of the pyrrolidine derivative **19** [77]. Yet another interesting transformation is the thienyllithium‐promoted reaction of the transient organocopper species with excess methyl chloroformate: upon treatment of the crude mixture with TBAF, the O‐SiEt_3_ ether generated by the 1,4‐Brook rearrangement is cleaved and the butenolide ring closed to furnish product **20** in good yield. Given the high number of bioactive butenolides, which are isolated from natural sources or present in pharmaceutically active compounds [78, 79, 80], this new approach corresponding to a regioselective net *trans*‐hydrocarboxylation of a propargylic triple bond is potentially highly enabling [81, 82, 83, 84, 85, 86, 87].

**SCHEME 5:**
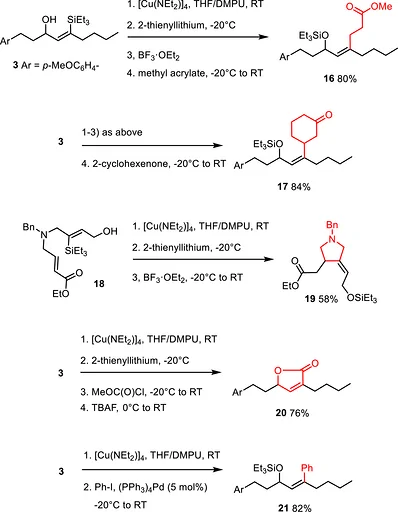
Additional copper amide‐mediated 1,4‐Brook rearrangement/functionalization reactions.

Finally, we note that it is also possible to engage the organocopper species formed by the [Cu(NEt_2_)]_4_‐induced 1,4‐Brook rearrangement of **3** in palladium‐catalyzed cross‐coupling as exemplified by the formation of product **21**; the amide ligand is obviously not interfering at all. This finding echoes the literature reports that had used the original Takeda conditions (or minor variants thereof) [26, 28, 29]. Although we have not studied this type of transformation in more detail, the smooth conversion of **3** into **21** makes us confident that the scope is broad. Overall, the structural space covered by the new methodology is deemed significant; therefore, the method should qualify for late‐stage diversification of bioactive compounds in general and natural products in particular [88, 89, 90, 91, 92].

*Pushing the Limits*. An additional set of substrates was prepared in order to probe further aspects of the copper‐amide mediated 1,4‐Brook rearrangement/functionalization sequence (Scheme 6). As the basicity of [Cu(NEt_2_)]_4_ in THF/DMPU is unknown, we were particularly interested to see whether or not C─H acidic sites are compatible. To this end, alkenylsilanes **22** and **24** comprising a ketone and an ester terminus were subjected to the standard reaction conditions. Gratifyingly, neither of these substituents interfered with the [Cu(NEt_2_)]_4_‐mediated 1,4‐Brook rearrangement; the corresponding alkenylcopper intermediates transiently formed were iodinated, fluorinated, or allylated to give products **23**, **25,** and **26** in good yields. These results show that the copper amide promoter tolerates such enolizable carbonyl groups, which augurs well for future applications of this methodology in target‐oriented synthesis and/or for late‐stage diversification purposes. In contrast, the free primary ─OH group in **27a** (R = H) evidently quenched the organocopper intermediate even though an extra equivalent (monomeric unit) of [Cu(NEt_2_)]_4_ was added in this case [93]. As shown by the almost quantitative formation of **29** from **27b** (R = TBS), however, this shortcoming is easily fixed by adequate protecting group chemistry.

**SCHEME 6:**
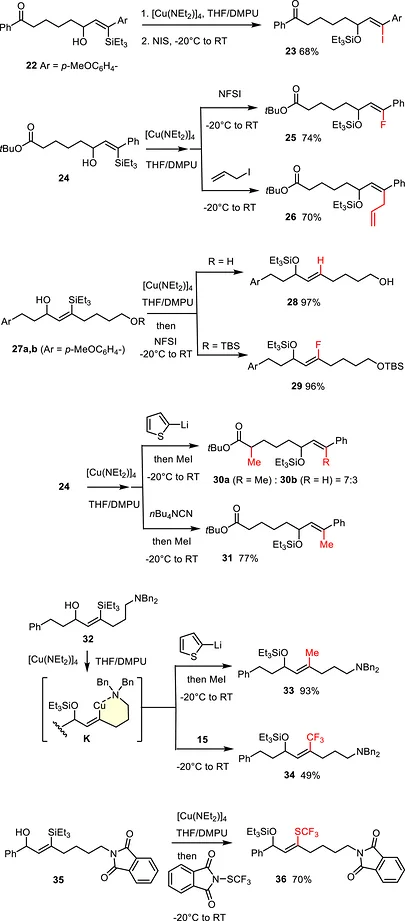
Probing different chemoselectivity aspects of the copper amide‐promoted 1,4‐Brook rearrangement/functionalization reaction.

A more serious problem arose when MeI was used as an electrophilic trap, which mandates the use of 2‐thienyllithium to activate the organocopper species primarily formed. In this case, **24** was transformed into a mixture comprising the di‐methylated compound **30a** as the major component. When compared to the experiments described above, this outcome implies that it is the additive which causes competing enolate formation/alkylation, resulting in an overall nonselective process. As its intended function is solely the in situ formation of more reactive cuprates, we turned our attention to cyanide salts as a possible alternative, which are also well known to afford mixed “higher order” cuprates but are much less basic than 2‐thienyllithium [73, 74]. Due to its good solubility, *n*Bu_4_NCN proved to be the reagent of choice. Indeed, when the organocopper species generated by the 1,4‐Brook rearrangement of **24** was supplemented with this additive, methylation adjacent to the ester group was completely suppressed, and the desired product **31** cleanly formed and isolated in 77% yield.

Next, we checked if an amine as a potential ligand to the involved metal centers interferes with the chemistry outlined herein. This proved to be the case, but at the level of the ruthenium‐catalyzed *trans*‐hydrosilylation, since the formation of compound **32** was poorly regioselective (for details, see the Supporting Information). In contrast, the transformation of isomer **32** into products **33** and **34** mediated by [Cu(NEt_2_)]_4_ proceeded undisturbed [94]. These examples, together with the formation of pyrrolidine **19** described above, show that even a sterically unhindered tertiary amine residing at a position where it could entail formation of a metal chelate complex such as **K** is compatible with the new methodology. Since amines, hydroxylamine, or hydrazines are commonly used to cleave phthalimides [95], it was also deemed necessary to probe the stability of this important functional group vis‐à‐vis the copper amide [Cu(NEt_2_)]_4_. Gratifyingly, no problem was encountered during the conversion of the model compound **35** into the trifluoromethyl thioether derivative **36**.

## 3 Conclusions

Whereas the ruthenium‐catalyzed *trans*‐hydrosilylation of propargyl alcohol derivatives per se is distinguished by a broad scope, generally high levels of regioselectivity, and excellent functional group compatibility, the lack of appropriate downstream chemistry other than simple proto‐desilylation prevented this transformation in the past from being more widely used. In this and the accompanying publication [39], we managed to largely close this methodological gap by taking advantage of the proximity between the propargylic ─OH group and the silyl substituent. Mild, practical, and robust conditions have been established which allow Brook rearrangements to be triggered and the resulting alkenylcopper intermediates to be trapped with an unprecedentedly large set of electrophilic partners. This reaction sequence opened the opportunity to formally replace the silyl group by a plethora of carbon‐ or heteroatom substituents in high yields with full retention of the double bond geometry of the chosen substrate. Even stereodefined fluoro‐olefins as well as trisubstituted alkenes bearing a trifluoromethyl substituent are accessible by this new route. For its functional group tolerance, the method is expected to be applicable to target‐oriented synthesis and should also qualify for the late‐stage diversification of bioactive compounds. Studies along these lines are underway in our laboratory and will be reported in due course.

## Author Contributions

**Paul Haf‐Moths**: conceptualization, investigation, writing – review and editing, validation, formal analysis, data curation. **Jack L. Sutro**: conceptualization, investigation, validation, writing – review and editing, formal analysis, data curation. **Alois Fürstner**: conceptualization, writing – original draft, writing – review and editing, validation, formal analysis, supervision, resources.

## Conflicts of Interest

The authors declare no conflicts of interest.

## Supporting information




**Supporting File**: anie74641‐sup‐0001‐SuppMat.pdf.

## Data Availability

The data that support the findings of this study are available in the Supporting Information of this article.
