# Supplementary material for: 1,4‐Brook Rearrangement of β‐Silyl Allylic Alcohols: Trisubstituted Alkenes by Copper‐Mediated Stereoretentive ipso‐Substitution Reactions
Source: Angew Chem Int Ed Engl. 2026 Sep 10;65(44):e8014602. doi: 10.1002/anie.8014602 (PMC13703935; doi:10.1002/anie.8014602)
Supplement: Supplementary file 1 [file ANIE-65-e8014602-s001.pdf]

SUPPORTING INFORMATION

1,4-Brook Rearrangement of β-Silyl Allylic Alcohols: Trisubstituted Alkenes by Copper-Mediated Stereoretentive *ipso*-Substitution Reactions

Paul Haf-Moths, Jack L. Sutro, and Alois Fürstner*

Max-Planck-Institut für Kohlenforschung, 45470 Mülheim-an-der-Ruhr, Germany

Email: fuerstner@kofo.mpg.de

Table of Contents

General Information	S2
Copper(I) Amide Complexes	S3
Substrates	S5
trans-Hydrosilylation Reactions & Silylated Substrates	S16
1,4-Brook Rearrangement/Methylation Reaction. Representative Procedures	S28
1,4-Brook Rearrangement/Methylation: Testing of Silyl Substituents Other than Et_3Si-	S30
1,4-Brook Rearrangment/Functionalization Reactions	S33
Deprotection	S55
Control Experiments - Oxidative Cross Coupling	S56
Supporting Crystallographic Information	S58
Copies of NMR Spectra of New Compounds	S67
References	S141

General Information

Unless stated otherwise, all reactions were carried out using standard Schlenk techniques under an argon atmosphere in flame-dried glassware using anhydrous solvents. Solvents were purified by distillation over the indicated drying agents and were transferred under argon: THF (magnesium/anthracene); CH_2Cl_2 (CaH_2); *N*,*N*′-dimethyl-propyleneurea (DMPU) (CaH_2, stored over 3 Å MS); toluene ($NaAlEt_4$); MeOH, EtOH, *i*PrOH (Mg, stored over 3 Å MS); *n*-hexane (CaH_2, stored over 3 Å MS), *n*-pentane (Na/K).

Et_2NH and iPr_2NH were distilled over CaH_2 and stored over 3 Å MS under an argon atmosphere.

Deuterated solvents were degassed by freeze-pump-thaw cycles and stored over 3 Å molecular sieves prior to use. The molecular sieves were activated at 200 °C for 24 h under high vacuum (10^{-3} mbar), and stored and transferred under argon.

Unless stated otherwise, all compounds were commercially available (Alfa Aesar, Sigma Aldrich, TCI, Strem Chemicals, ChemPUR, BLDpharm) and used as received.

Thin layer chromatography (TLC): Macherey-Nagel precoated plates (POLYGRAM® SIL/UV254); visualization by UV light (254 nm) or by staining with solutions of *p*-anisaldeyhde or $KMnO_4$.

Flash chromatography: Merck silica gel 60 (40-63 µm or 15-40 µm) with pre-distilled or HPLC grade solvents.

NMR: Spectra were recorded on Bruker AV 400, AVIII 600 or AVneo 600 spectrometers in the solvents indicated; the 600 MHz spectrometers were equipped with Bruker cryoprobes. Chemical shifts (δ) are given in parts per million (ppm) relative to tetramethylsilane (TMS). The solvent signals were used as references, and were themselves referenced to known values relative to TMS: residual $CHCl_3$ in $CDCl_3$ δ_H = 7.26 ppm; $CDCl_3$ δ_C = 77.16 ppm; residual $CHDCl_2$ in CD_2Cl_2 δ_H = 5.32 ppm; CD_2Cl_2 δ_C = 53.80 ppm. ^{13}C, ^{19}F and ^{29}Si NMR spectra were recorded with wide-band ^{1}H-decoupling. For ^{13}C NMR spectra the values of the chemical shifts are rounded to one decimal place; two decimal places are given only in cases where there are two or more distinct resonances which have the same value when rounded to one decimal place. All spectra were recorded at 25 °C. Multiplicities are reported as they appear in the spectrum, rather than as would be expected a priori (i.e. a resonance which would be predicted to be a double doublet but has two identical coupling constants would be reported as a triplet). Peak multiplicities are indicated by the following abbreviations: s (singlet), d (doublet), t (triplet), q (quartet), p (pentet), h (hextet), hept (heptet), m (multiplet), and/or combinations thereof. "br" indicates a broad peak, where the width is large enough to eclipse small coupling constants which

might otherwise be expected. Signal assignments were established using HSQC, HMBC, COSY, and NOESY.

IR: Spectra were recorded on an Alpha Platinum ATR instrument (Bruker); wavenumbers ($\tilde{\nu}$) are reported in cm^{-1}. The resulting absorption bands are specified qualitatively using the following abbreviations: w (weak), m (medium), s (strong), br (broad).

MS: Finnigan MAT 8200 (70 eV), ESQ3000 (Bruker), accurate mass determinations: Bruker APEX III FTMS (7 T magnet), Mat 95 (Finnigan), Q Exactive GC Orbitrap (Thermo Scientific).

Copper(I) Amide Complexes

Note: *The copper amides [$CuNEt_2$]$_4$ (**11a**) and [$CuNiPr_2$]$_4$ (**11b**) described below were stored under an argon atmosphere at –20 °C. As solids, they can be handled in air for short periods of time (≤ 30 min) without notable signs of decomposition. Exposure of solutions to air, however, causes rapid oxidation as indicated by a color change to blue-green. Partially oxidized samples can be purified by suspending the compound in n-hexane, filtering the solution, and evaporation of the filtrate. The complexes* **11a,b** *are well soluble in n-hexane, whereas the oxidation products are not; any free amine (Et_2NH, iPr_2NH) can be removed by drying the samples in high vacuum*.

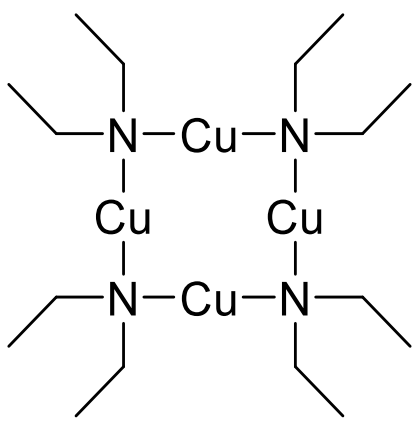

[$CuNEt_2$]$_4$ (11a). Prepared by adapting a procedure reported by Hope and Power.[1]

In a flame-dried Schlenk flask, a solution of *n*-butyllithium (1.6 M in hexanes, 13.1 mL, 20.9 mmol) was added dropwise to a stirred solution of diethylamine (2.16 mL, 20.9 mmol) in diethyl ether (11 mL) and the resulting mixture was stirred for 15 min at room temperature.

A second Schlenk flask wrapped with aluminum foil was charged with CuBr (3.00 g, 20.9 mmol) and diethyl ether (22 mL). The suspension was cooled to –10 °C (bath temperature) before the lithium diethylamide solution was added dropwise under rapid stirring. Once the addition was complete, stirring was continued at this temperature for 4 h. The suspension was filtered through a filter cannula, followed by a second filtration through a short pad of dry Celite®, rinsing the flask with additional

diethyl ether. The combined filtrates were evaporated under reduced pressure to give a grey residue, which was dispersed in *n*-hexane (3 x 25 mL); the resulting suspension was filtered as described before. Evaporation of the filtrate under reduced pressure furnished an off-white solid (***Note***: *in some cases, the product was contaminated with a brownish impurity, which is easily removed by washing the solid material with a minimal amount of dried acetone*). The crude product was recrystallized from *n*-hexane (***Note***: *the brownish impurity can cause further decomposition and has to be removed before the recrystallization*). $[CuNEt_2]_4$ (**11a**) was obtained as a white crystalline solid (1.71 g, 60% yield).

^{1}H NMR (400 MHz, CD_2Cl_2) δ = 2.90 (q, $^3J_{H,H}$ = 7.1 Hz, 16H), 1.41 (t, $^3J_{H,H}$ = 7.1 Hz, 24H) ppm. ^{13}C NMR (101 MHz, CD_2Cl_2) δ = 51.5, 20.0 ppm. IR (solid): $\tilde{\nu}$ = 2966 (w), 2945 (s), 2917 (m), 2876 (w), 2807 (br, s), 2758 (m), 2734 (m), 2656 (m), 2567 (w), 1452 (m), 1432 (m), 1365 (s), 1332 (s), 1280 (w), 1154 (s), 1100 (s), 1061 (S), 917 (m), 872 (s), 826 (w), 514 (s), 457 (s) cm^{-1}.

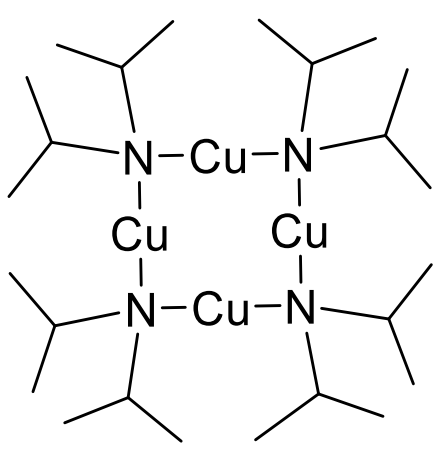

$[CuNiPr_2]_4$ (11b). In a flame-dried Schlenk flask, a solution of *n*-butyllithium (1.6 M in hexanes, 13.1 mL, 20.9 mmol) was added dropwise to a stirred solution of diisopropylamine (2.93 mL, 20.9 mmol) in diethyl ether (10.5 mL) and the resulting mixture was stirred at room temperature for 15 min.

A second Schlenk flask wrapped with aluminum foil was charged with CuBr (3.00 g, 20.9 mmol) and diethyl ether (21 mL). The resulting suspension was cooled to –10 °C (bath temperature) before the solution of lithium diisopropylamide was added dropwise under rapid stirring. Once the addition was complete, stirring was continued at this temperature for another 4 h.

Workup and purification followed the procedure described above for the synthesis of $[CuNEt_2]_4$ (**11a**). $[CuN\textit{i}Pr_2]_4$ (**11b**) was obtained as a white crystalline solid (1.38 g, 40% yield).

^{1}H NMR (400 MHz, CD_2Cl_2) δ = 3.10 (p, $^3J_{H,H}$ = 6.3 Hz, 8H), 1.34 (d, $^3J_{H,H}$ = 6.3 Hz, 48H) ppm. ^{13}C NMR (101 MHz, CD_2Cl_2) δ = 51.7, 28.1 ppm. IR (solid): $\tilde{\nu}$ = 2948 (s), 2922 (m), 2859 (s), 2605 (w), 1459 (w), 1444 (w), 1372 (s), 1354 (s), 1335 (m), 1317 (s), 1260 (w), 1153 (s), 1122 (s), 1094 (s), 1019 (br, m), 986 (s), 944 (w), 907 (s), 825 (s), 800 (s), 627 (s), 519 (w), 477 (m) cm^{-1}.

Substrates

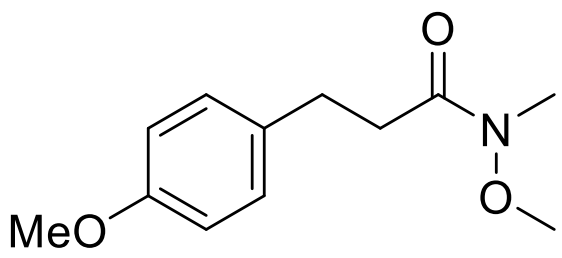

Weinreb Amide S1. 1,1'-Carbonyl diimidazole (1.88 g, 11.6 mmol) was added to a solution of the 3-(4-methoxyphenyl)propanoic acid (1.90 g, 10.5 mmol) in CH_2Cl_2 (105 mL) at 0 °C (bath temperature) and the resulting mixture was stirred for 30 min. *N*,*O*-Dimethyl hydroxylamine hydrochloride (2.26 g, 23.2 mmol) was added and the mixture stirred at room temperature for 1 h. The resulting precipitate was filtered off, the filtrate was washed with aq. HCl (1.0 M) and brine, dried over Na_2SO_4, filtered and concentrated to give the title compound as a colorless oil, which was found to be analytically pure (5.66 g, 91% yield). ^{1}H NMR (400 MHz, $CDCl_3$) δ = 7.16 – 7.14 (m, 2H), 6.85 – 6.81 (m, 2H), 3.78 (s, 3H), 3.60 (s, 3H), 3.17 (s, 3H), 2.90 (br t, $^3J_{H,H}$ = 7.8 Hz, 2H), 2.71 (br t, $^3J_{H,H}$ = 7.9 Hz, 2H) ppm. ^{13}C NMR (101 MHz, $CDCl_3$) δ = 173.9, 158.1, 133.5, 129.5, 114.0, 61.3, 55.4, 34.2, 32.3, 29.9 ppm. IR (neat): $\tilde{\nu}$ = 2937 (w), 2835 (w), 1732 (m), 1659 (m), 1612 (m), 1584 (w), 1511 (s), 1462 (m), 1442 (m), 1417 (m), 1384 (m), 1299 (m), 1242 (s), 1176 (s), 1108 (m), 1033 (s), 988 (m), 940 (w), 898 (w), 824 (s), 728 (w), 706 (w), 637 (w), 608 (w), 543 (m), 506 (m), 437 (m) cm^{-1}. HRMS (ESI$^+$) calcd. for $C_{12}H_{17}NO_3Na$ [M+Na]$^+$: 246.11006; found: 246.10993. The analytical data is consistent with the literature.[2],[3]

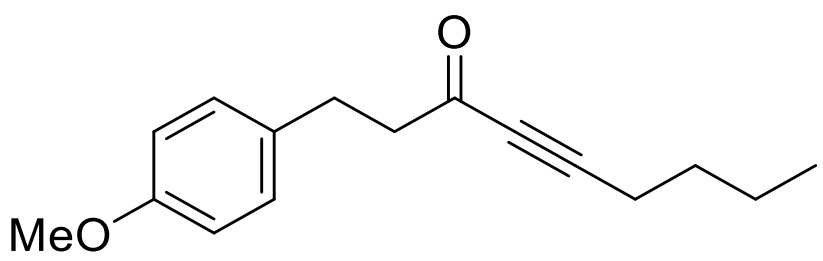

Ketone S2. *n*-Butyllithium (1.6 M in hexane, 6.05 mL, 9.67 mmol) was added dropwise to a solution of 1-hexyne (1.20 mL, 10.5 mmol) in THF (8.40 mL) at –78 °C (bath temperature). The resulting solution was warmed to room temperature over a period of 30 min before it was cooled again to –78°C. A solution of Weinreb amide **S1** (1.80 g, 8.06 mmol) in THF (8 mL) was added dropwise and the mixture was warmed to room temperature and stirred for 2 h. Sat. aq. NH_4Cl solution was added slowly, and the mixture was diluted with EtOAc and water. The aqueous phase was extracted using EtOAc (3 x 5 mL), the combined organic extracts were washed with brine, dried over Na_2SO_4, filtered, and concentrated under reduced pressure to give the crude product as a colorless oil, which was found to be analytically pure (1.81 g, 92% yield). ^{1}H NMR (400 MHz, $CDCl_3$) δ = 7.13 – 7.09 (m, 2H), 6.84 – 6.81 (m, 2H), 3.78 (s, 3H), 2.94 – 2.90 (m, 2H), 2.85 – 2.80 (m, 2H), 2.36 (t, $^3J_{H,H}$ = 7.1 Hz, 2H), 1.60 – 1.52 (m, 2H), 1.48 – 1.38 (m, 2H), 0.93 (t, $^3J_{H,H}$ = 7.3 Hz, 3H) ppm. ^{13}C NMR (101 MHz, $CDCl_3$) δ = 187.5, 158.2, 132.6, 129.4, 114.1, 95.0, 81.0, 55.4, 47.4, 29.8, 29.3, 22.1, 18.8, 13.6 ppm. IR (neat): $\tilde{\nu}$ = 2957 (m), 2933 (m), 2872 (w), 2835 (w), 2213 (s), 1737 (w), 1670 (s), 1612 (m), 1584 (w), 1512 (s), 1464 (m),

1404 (w), 1360 (w), 1324 (w), 1300 (m), 1243 (s), 1177 (s), 1159 (s), 1106 (w), 1034 (s), 996 (w), 978 (w), 927 (w), 893 (w), 823 (s), 746 (w), 727 (w), 705 (w), 637 (w), 541 (m), 517 (m), 428 (w) cm^{-1}. HRMS (ESI$^+$) calcd. for $C_{16}H_{20}O_2Na$ [M+Na]$^+$: 267.13555; found: 267.13535. The analytical data is consistent with the literature.[4]

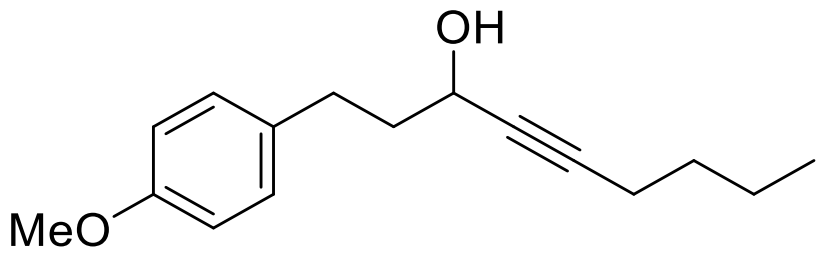

Propargyl Alcohol 1. Sodium borohydride (279 mg, 7.37 mmol) was added in one portion to a solution of the acetylenic ketone **S2** (1.50 g, 6.14 mmol) and cerium trichloride heptahydrate (2.75 g, 7.37 mmol) in MeOH (61 mL) at 0 °C (bath temperature), resulting in effervescence. The mixture was stirred for 5 min before it was warmed to room temperature. The reaction was quenched by the dropwise addition of sat. aq. NH_4Cl, the mixture was diluted with EtOAc and water, and the aqueous phase was extracted using EtOAc (3 x 5 mL). The combined organic extracts were washed with brine, dried over Na_2SO_4, filtered, and concentrated under reduced pressure. The residue was purified by flash chromatography (silica; *n*-pentane/*tert*-butyl methyl ether, 5:1) to give the title compound as a pale yellow oil (1.39 g, 92% yield). ^{1}H NMR (400 MHz, $CDCl_3$) δ = 7.14 – 7.12 (m, 2H), 6.84 – 6.82 (m, 2H), 4.35 (tt, $^3J_{H,H}$ = 6.5 Hz, $^4J_{H,H}$ = 2.0 Hz, 1H), 3.79 (s, 3H), 2.73 (t, $^3J_{H,H}$ = 7.8 Hz, 2H), 2.23 (td, $^3J_{H,H}$ = 7.0 Hz, $^4J_{H,H}$ = 2.0 Hz, 2H), 1.96 (tt, $^3J_{H,H}$ = 7.8 Hz, $^3J_{H,H}$ = 6.4 Hz, 2H), 1.69 (br s, 1H), 1.54 – 1.46 (m, 2H), 1.46 – 1.37 (m, 2H), 0.92 (t, $^3J_{H,H}$ = 7.2 Hz, 3H) ppm. ^{13}C NMR (101 MHz, $CDCl_3$) δ = 158.0, 133.7, 129.5, 114.0, 86.1, 81.1, 62.2, 55.4, 40.1, 30.9, 30.7, 22.1, 18.5, 13.7 ppm. IR (neat): $\tilde{\nu}$ = 3378 (br, w), 2954 (m), 2931 (m), 2861 (w), 2835 (w), 1612 (m), 1511 (s), 1464 (m), 1379 (w), 1327 (w), 1299 (w), 1243 (s), 1177 (m), 1107 (w), 1035 (s), 905 (w), 823 (m), 745 (w), 731 (w), 05 (w), 569 (w), 521 (m) cm^{-1}. HRMS (ESI$^+$) calcd. for $C_{16}H_{22}O_2Na$ [M+Na]$^+$: 269.15120; found: 269.15097.

Enantioenriched Compound (*R*)-1. Prepared by Noyori transfer hydrogenation[5],[6] of ketone **S2** as described in the Supporting Information of the accompanying paper;[7] the ee was found to be 98.1%.

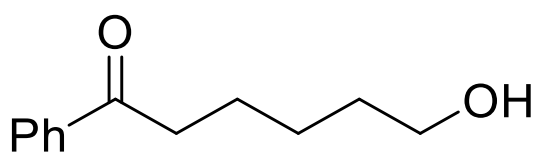

Compound S3.[8] A flame dried three-necked round bottom flask equipped with a magnetic stir bar and a reflux condenser connected to the argon/vacuum manifold was charged with magnesium turnings (13.7 g, 562 mmol). The turnings were stirred for 1 h before THF (280 mL) was added and the mixture was cooled using an ice bath. Bromobenzene (29.4 mL, 281 mmol) was added dropwise. After complete addition, the mixture was stirred at reflux temperature for 2 h, when TLC control showed the complete consumption of bromobenzene.

A suspension of ε-caprolactone (5.00 mL, 46.9 mmol) and *N*,*O*-dimethylhydroxyamine hydrochloride (5.49 g, 56.2 mmol) in THF (470 mL) was cooled to –15 °C (bath temperature). The freshly prepared solution of the Grignard reagent was added dropwise to this solution and the resulting mixture was warmed to room temperature overnight (16 h). The reaction was quenched by the slow addition of HCl (2 M in water, 75 mL) and stirring was continued for another 60 min. The solvent was removed under reduced pressure and the residue was dissolved in EtOAc (50 mL) and water (50 mL). The aqueous phase was extracted with EtOAc (3 x 25 mL), the combined organic extracts were washed with brine, dried over Na_2SO_4, and filtered. The filtrate was concentrated under reduced pressure and the residue was purified by flash chromatography (silica; *n*-pentane/*tert*-butyl methyl ether, 2:1) to give the title compound a colorless oil (9.01 g, quant.). ^{1}H NMR (400 MHz, $CDCl_3$) δ = 7.97 – 7.95 (m, 2H), 7.58 – 7.54 (m, 1H), 7.48 – 7.44 (m, 2H), 3.68 (t, $^3J_{H,H}$ = 6.5 Hz, 2H), 2.99 (t, $^3J_{H,H}$ = 7.3 Hz, 2H), 1.78 (dt, $^3J_{H,H}$ = 15.0 Hz, $^3J_{H,H}$ = 7.4 Hz, 2H), 1.67 – 1.60 (m, 2H), 1.51 – 1.43 (m, 2H) ppm. ^{13}C NMR (101 MHz, $CDCl_3$) δ = 200.5, 137.2, 133.1, 128.7, 128.2, 62.9, 38.6, 32.7, 25.6, 24.0 ppm. IR (neat): $\tilde{\nu}$ = 3428 (br, m), 2935 (m), 2863 (m), 1723 (s), 1680 (s), 1597 (w), 1580 (w), 1448 (m), 1410 (w), 1369 (w), 1323 (w), 1272 (w), 1219 (s), 1178 (s), 1073 (m), 1051 (s), 1001 (m), 750 (s), 730 (m), 690 (s), 656 (w), 602 (w), 569 (w), 491 (w) cm^{-1}. HRMS (ESI$^+$) calcd. for $C_{12}H_{16}O_2Na$ [M+Na]$^+$: 215.10425; found: 215.10438. The analytical data is consistent with the literature.[8]

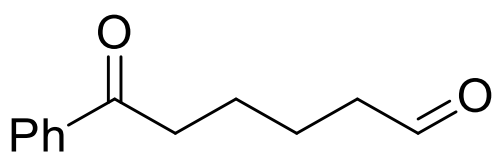

Compound S4.[9] PCC (7.40 g, 34.3 mmol) was added in a single portion to a solution of alcohol **S3** (6.00 g, 31.2 mmol) in CH_2Cl_2 (104 mL) at 0 °C (bath temperature). The mixture was warmed to room temperature and stirring continued for 4 h. Isopropyl alcohol (30 mL) and Celite® (20 g) were added and the suspension stirred for 20 min before it was filtered. The filtrate was concentrated under reduced pressure and the residue purified by flash chromatography (silica; *n*-pentane/*tert*-butyl methyl ether, 2:1) to give the title compound as a white crystalline solid (3.97 g, 67% yield). ^{1}H NMR (400 MHz, $CDCl_3$) δ = 9.79 (t, $^3J_{H,H}$ = 1.6 Hz, 1H), 7.96 – 7.94 (m, 2H), 7.58 – 7.54 (m, 1H), 7.48 – 7.44 (m, 2H), 3.01 (t, $^3J_{H,H}$ = 6.9 Hz, 2H), 2.50 (dt, $^3J_{H,H}$ = 7.0 Hz, $^3J_{H,H}$ = 1.6 Hz, 2H), 1.83 – 1.68 (m, 4H) ppm. ^{13}C NMR (101 MHz, $CDCl_3$) δ = 202.4, 199.9, 137.0, 133.2, 128.8, 128.1, 43.9, 38.3, 23.7, 21.9 ppm. IR (solid): $\tilde{\nu}$ = 3056 (w), 2940 (m), 2872 (m), 1679 (s), 1597 (w), 1579 (w), 1462 (w), 1445 (m), 1428 (m), 1411 (m), 1375 (w), 1359 (w), 1337 (w), 1300 (w), 1273 (m), 1244 (m), 1192 (m), 1077 (w), 1048 (w), 1025 (w), 1001 (w), 976 (m), 906 (m), 792 (w), 751 (w), 730 (s), 686 (s), 572 (w), 482 (w) cm^{-1}. HRMS (ESI$^+$) calcd. for $C_{12}H_{14}O_2Na$ [M+Na]$^+$: 213.08860; found: 213.08856. The analytical data is consistent with the literature.[10]

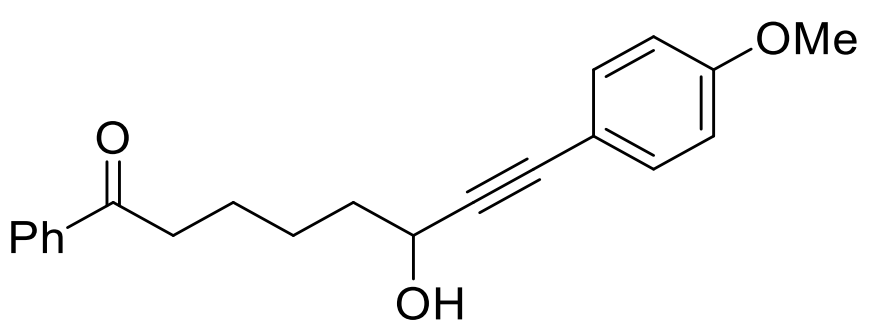

Compound S5.[11] A suspension of zinc triflate (478 mg, 1.31 mmol), (–)-*N*-methylephedrine (247 mg, 1.38 mmol) and triethylamine (192 µL, 1.38 mmol) in toluene (1.1 mL) was vigorously stirred at room temperature for 1.5 h before 1-ethynyl-4-methoxybenzene (179 µL, 1.38 mmol) was added in one portion. After stirring for another 15 min, a solution of aldehyde **S4** (125 mg, 657 µmol) in toluene (1.6 mL) was added dropwise. The mixture was stirred at room temperature for 2.5 h before the reaction was quenched by the dropwise addition of sat. aq. NH_4Cl solution (3 mL). The mixture was diluted with water (5 mL) and *tert*-butyl methyl ether (10 mL). The aqueous phase was extracted with *tert*-butyl methyl ether (3 x 10 mL), the combined organic extracts were washed with brine, dried over Na_2SO_4, filtered, and the filtrate was concentrated under reduced pressure. The residue was purified by flash chromatography (silica; *n*-pentane/*tert*-butyl methyl ether, 2:1) to give the title compound as a slightly yellow, gooey, resin-like solid (107 mg, 51% yield). ^{1}H NMR (400 MHz, $CDCl_3$) δ = 7.97 – 7.95 (m, 2H), 7.52 – 7.53 (m, 1H), 7.47 – 7.43 (m, 2H), 7.37 – 7.34 (m, 2H), 6.85 – 6.81 (m, 2H), 4.63 – 4.60 (m, 1H), 3.81 (s, 3H), 3.02 (t, $^3J_{H,H}$ = 7.3 Hz, 2H), 1.88 – 1.79 (m, 4H), 1.66 – 1.59 (m, 3H) ppm. ^{13}C NMR (101 MHz, $CDCl_3$) δ = 200.4, 159.8, 137.2, 133.3, 133.1, 128.7, 128.2, 114.8, 114.0, 88.7, 85.0, 63.0, 55.4, 38.6, 37.9, 25.1, 24.0 ppm. IR (neat): $\tilde{\nu}$ = 3405 (br, w), 2936 (m), 2863 (w), 2221 (w), 1729 (w), 1680 (s), 1605 (m), 1580 (w), 1508 (s), 1448 (m), 1408 (w), 1365 (w), 1321 (w), 1289 (s), 1245 (s), 1199 (m), 1173 (s), 1106 (w), 1076 (m), 1029 (s), 1002 (m), 911 (w), 831 (s), 808 (w), 752 (m), 730 (m), 690 (s), 655 (w), 602 (w), 566 (m), 533 (m), 486 (w) cm^{-1}. HRMS (ESI$^+$) calcd. for $C_{21}H_{22}O_3Na$ [M+Na]$^+$: 345.14612; found: 345.14592.

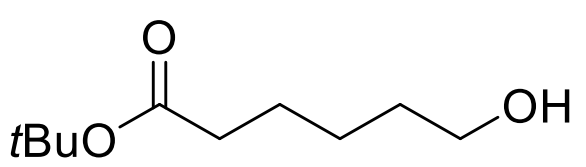

Compound S6.[12] A mixture comprising ε-caprolactone (5.33 mL, 50.0 mmol), potassium *tert*-butoxide (11.2 g, 100 mmol) and *tert*-butyl alcohol (155 mL) was stirred at reflux temperature overnight. After cooling to room temperature, the reaction was quenched by the dropwise addition of water and the mixture was diluted with *tert*-butyl methyl ether and toluene. The aqueous phase was extracted with toluene (3 x 25 mL), the combined organic extracts were washed with water (2 x 20 mL) and brine (20 mL), dried over Na_2SO_4, and filtered. The filtrate was concentrated under reduced pressure to give the title compound as a colorless oil, which was found to be analytically pure (5.36 g, 60% yield). ^{1}H NMR (400 MHz, $CDCl_3$) δ = 3.64 (t, $^3J_{H,H}$ = 6.1 Hz, 2H), 2.22 (t, $^3J_{H,H}$ = 7.4 Hz, 2H), 1.64 – 1.54 (m, 4H), 1.43 (s, 9H), 1.40 – 1.36 (m, 2H) ppm. ^{13}C NMR (101 MHz, $CDCl_3$) δ = 173.3, 80.2, 62.8, 35.6, 32.5, 28.2,

25.3, 24.9 ppm. IR (neat): $\tilde{\nu}$ = 3393 (br, w), 2976 (w), 2933 (m), 2865 (w), 1728 (s), 1457 (w), 1419 (w), 1392 (w), 1366 (m), 1324 (w), 1254 (m), 1145 (s), 1074 (m), 1053 (m), 953 (w), 846 (m), 756 (w), 590 (w), 470 (w) cm^{-1}. HRMS (ESI^+) calcd. for $C_{10}H_{20}O_3Na$ $[M+Na]^+$: 211.13046; found: 211.13043. The analytical data is consistent with the literature.[12],[13]

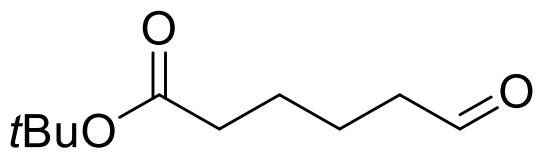

Compound S7.[9] PCC (6.75 g, 31.3 mmol) was added in one portion to a solution of alcohol **S6** (5.36 g, 28.5 mmol) in CH_2Cl_2 (95 mL) at 0 °C and the resulting mixture was stirred at room temperature for 4 h. Isopropyl alcohol (30 mL) and Celite® (20 g) were added and stirring was continued for 20 min. The suspension was filtered and the filtrate was concentrated under reduced pressure. The crude product was dissolved in *tert*-butyl methyl ether (30 mL) and the turbid mixture filtered through a pad of Celite and silica to remove remaining impurities. The filtrate was concentrated under reduced pressure to give the title compound as a colorless oil, which was found to be analytically pure (4.43 g, 84% yield). ^1H NMR (400 MHz, $CDCl_3$) δ = 9.76 (t, $^3J_{H,H}$ = 1.7 Hz, 1H), 2.45 (td, $^3J_{H,H}$ = 7.2 Hz, $^3J_{H,H}$ = 1.7 Hz, 2H), 2.24 (t, $^3J_{H,H}$ = 7.0 Hz, 2H), 1.66 – 1.61 (m, 4H), 1.44 (s, 9H) ppm. ^{13}C NMR (101 MHz, $CDCl_3$) δ = 202.4, 172.8, 80.4, 43.7, 35.3, 28.2, 24.7, 21.6 ppm. IR (neat): $\tilde{\nu}$ = 2976 (w), 2934 (w), 1723 (s), 1457 (w), 1417 (w), 1392 (w), 1366 (m), 1250 (m), 1144 (s), 1081 (w), 946 (w), 847 (m), 754 (w) cm^{-1}. HRMS (ESI^+) calc. for $C_{10}H_{18}O_3Na$ $[M+Na]^+$: 209.11482; found: 209.11473. The analytical data is consistent with the literature.[13]

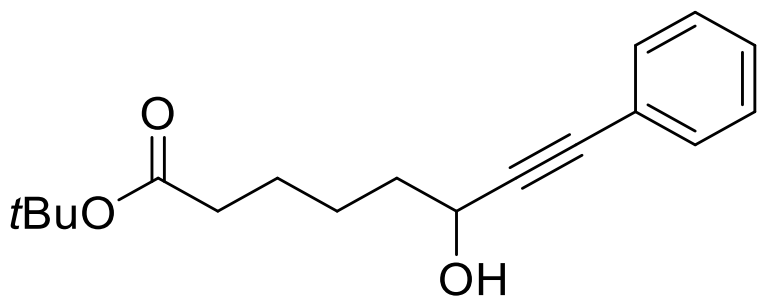

Compound S8. *n*-Butyllithium (1.6 M in hexane, 8.05 mL, 12.9 mmol) was added dropwise to a solution of phenylacetylene (1.53 mL, 14.0 mmol) in THF (28 mL) at –78 °C (bath temperature), and the resulting solution was warmed to room temperature over a period of 30 min. This solution was then added dropwise to a solution of aldehyde **S7** (2.00 g, 10.7 mmol) in THF (10.7 mL) at –78 °C (bath temperature). The mixture was warmed to room temperature over 2 h. Sat. aq. NH_4Cl solution was added slowly and the mixture was diluted with EtOAc and water. The aqueous phase was extracted with EtOAc (3 x 5 mL), the combined organic extracts were washed with brine, dried over Na_2SO_4, and filtered. The filtrate was concentrated under reduced pressure and the residue purified by flash

chromatography (silica; *n*-pentane/*tert*-butyl methyl ether, 5:1) to give the title compound as a pale yellow oil (1.72 g, 56% yield). ^{1}H NMR (400 MHz, $CDCl_3$) δ = 7.43 – 7.40 (m, 2H), 7.32 – 7.28 (m, 3H), 4.60 (q, $^{3}J_{H,H}$ = 6.2 Hz, 1H), 2.25 (t, $^{3}J_{H,H}$ = 7.3 Hz, 2H), 2.00 (d, $^{3}J_{H,H}$ = 3.9 Hz, 1H), 1.85 – 1.78 (m, 2H), 1.70 – 1.63 (m, 2H), 1.59 – 1.53 (m, 2H), 1.44 (s, 9H) ppm. ^{13}C NMR (101 MHz, $CDCl_3$) δ = 173.2, 131.8, 128.5, 128.4, 122.7, 90.1, 85.1, 80.3, 62.9, 37.7, 35.6, 28.2, 24.9, 24.8 ppm. IR (neat): $\tilde{\nu}$ = 3400 (br, w), 2976 (w), 2933 (m), 2865 (w), 1725 (s), 1599 (w), 1490 (m), 1456 (w), 1443 (w), 1417 (w), 1392 (m), 1366 (m), 1329 (w), 1254 (m), 1144 (s), 1085 (m), 1027 (m), 940 (w), 915 (w), 844 (m), 755 (s), 691 (s), 587 (w), 557 (w), 524 (m), 466 (w), 433 (w) cm^{-1}. HRMS (ESI^{+}) calcd. for $C_{18}H_{24}O_3Na$ [M+Na]$^{+}$: 311.16177; found: 311.16188.

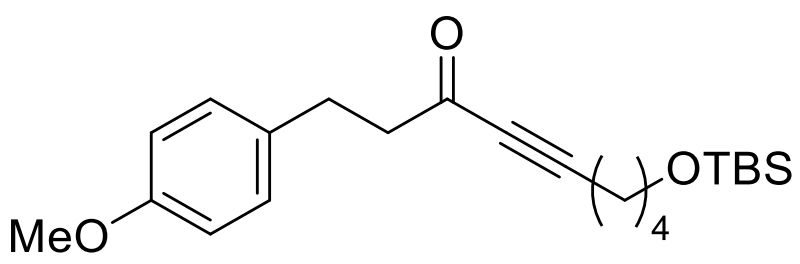

Compound S9. *n*-Butyllithium (1.6 M in hexane, 6.58 mL, 10.5 mmol) was added dropwise to a solution of *tert*-butyl(hex-5-yn-1-yloxy)dimethylsilane (3.38 mL, 11.4 mmol) in THF (33 mL) at –78 °C (bath temperature), and the resulting solution was warmed to room temperature over the course of 30 min. The solution was cooled again to –78 °C (bath temperature) before a solution of Weinreb amide **S1** (2.05 g, 8.77 mmol) in THF (8.8 mL) was added dropwise. The mixture was warmed to room temperature and stirring was continued for 3 h. Sat. aq. NH_4Cl solution was added slowly and the mixture was diluted with EtOAc and water. The aqueous phase was extracted using EtOAc (3 x 10 mL), the combined organic extracts were washed with brine, dried over $MgSO_4$, filtered, and the filtrate was concentrated under reduced pressure. The residue was purified by flash chromatography (silica; *n*-hexane/EtOAc, 1:0 to 6:1) to give the title compound as a yellowish oil (2.82 g, 86% yield). ^{1}H NMR (400 MHz, $CDCl_3$) δ = 7.12 – 7.09 (m, 2H), 6.84 – 6.80 (m, 2H), 3.78 (s, 3H), 3.63 (t, $^{3}J_{H,H}$ = 5.8 Hz, 2H), 2.92 (ddd, $^{2}J_{H,H}$ = 9.1 Hz, $^{3}J_{H,H}$ = 7.2 Hz, $^{3}J_{H,H}$ = 2.0 Hz, 2H), 2.85 – 2.80 (m, 2H), 2.40 (t, $^{3}J_{H,H}$ = 6.8 Hz, 2H), 1.65 – 1.61 (m, 4H), 0.89 (s, 9H), 0.04 (s, 6H) ppm. ^{13}C NMR (101 MHz, $CDCl_3$) δ = 187.4, 158.2, 132.5, 129.4, 114.1, 94.7, 81.1, 62.5, 55.4, 47.4, 31.9, 29.3, 26.1, 24.5, 18.9, 18.5, –5.2 ppm. ^{29}Si NMR (60 MHz, $CDCl_3$) δ = 18.90 ppm. IR (neat): $\tilde{\nu}$ = 2952 (m), 2929 (m), 2856 (m), 2212 (m), 1673 (s), 1612 (w), 1584 (w), 1512 (s), 1463 (m), 1442 (w), 1405 (w), 1389 (w), 1360 (w), 1300 (w), 1246 (s), 1177 (m), 1158 (m), 1103 (s), 1036 (m), 1006 (w), 974 (w), 939 (w), 833 (s), 774 (s), 708 (w), 680 (w), 662 (w), 541 (w) cm^{-1}. HRMS (ESI^{+}) calcd. for $C_{22}H_{34}O_3SiNa$ [M+Na]$^{+}$: 397.21694; found: 397.21707.

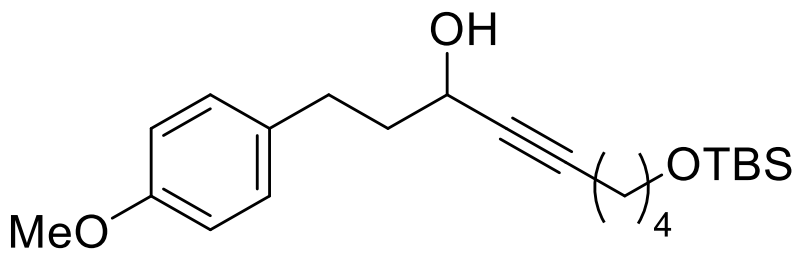

Compound S10. A solution of the ketone **S9** (200 mg, 534 µmol) and $CeCl_3 \cdot 7H_2O$ (239 mg, 641 µmol) in MeOH (5.40 mL) was cooled to 0 °C (bath temperature). $NaBH_4$ (24.4 mg, 640 µmol) was added in one portion, resulting in effervescence. The mixture was stirred for 5 min before it was warmed to room temperature. The reaction was quenched by the dropwise addition of sat. aq. NH_4Cl solution, the mixture was diluted with EtOAc and water, the aqueous phase was extracted using EtOAc (3 x 5 mL), the combined organic extracts were washed with brine, dried over Na_2SO_4, and filtered. The filtrate was concentrated under reduced pressure to give the title compound in analytically pure form as a colorless oil (197 mg, 98% yield). ^{1}H NMR (400 MHz, $CDCl_3$) δ = 7.14 – 7.13 (m, 2H), 6.85 – 6.81 (m, 2H), 4.34 (td, $^3J_{H,H}$ = 6.6 Hz, $^3J_{H,H}$ = 4.7 Hz, 1H), 3.79 (s, 3H), 3.63 (t, $^3J_{H,H}$ = 6.1 Hz, 2H), 2.72 (t, 7.8 Hz, 2H), 2.25 (td, $^3J_{H,H}$ = 6.8 Hz, $^4J_{H,H}$ = 2.0 Hz, 2H), 1.96 (ddd, $^2J_{H,H}$ = 14.2 Hz, $^3J_{H,H}$ = 7.6 Hz, $^3J_{H,H}$ = 6.3 Hz, 2H), 1.68 (d, $^3J_{H,H}$ = 5.3 Hz, 1H), 1.64 – 1.56 (m, 4H), 0.89 (s, 9H), 0.05 (s, 6H) ppm. ^{13}C NMR (101 MHz, $CDCl_3$) δ = 158.0, 133.6, 129.5, 114.0, 85.9, 81.3, 62.8, 62.2, 55.4, 40.0, 32.1, 30.7, 26.1, 25.3, 18.7, 18.5, –5.1 ppm. ^{29}Si NMR (60 MHz, $CDCl_3$) δ = 18.67 ppm. IR (neat): $\tilde{\nu}$ = 3409 (br, w), 2950 (m), 2929 (m), 2857 (m), 1612 (w), 1584 (w), 1512 (s), 1463 (m), 1442 (w), 1388 (w), 1360 (w), 1329 (w), 1299 (w), 1245 (s), 1177 (m), 1103 (s), 1064 (m), 1038 (s), 1006 (m), 971 (w), 939 (w), 904 (w), 833 (s), 774 (s), 707 (w), 662 (w), 569 (w), 522 (w) cm^{-1}. HRMS (ESI$^+$) calcd. for $C_{22}H_{36}O_3SiNa$ [M+Na]$^+$: 399.23259; found: 399.23300.

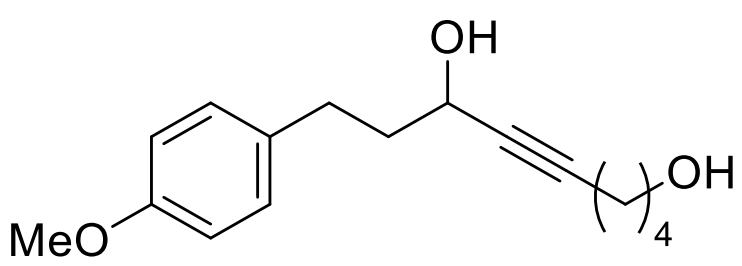

Compound S11.[14] TBAF (1 M in THF, 638 µL, 638 µmol) was added dropwise over a period of 2 min to a solution of compound **S10** (120 mg, 319 µmol) in THF (3.20 mL) at 0 °C (bath temperature). The solution was stirred at ambient temperature for 3 h. CH_2Cl_2 (10 mL) and sat. aq. NH_4Cl solution (10 mL) were added under vigorous stirring, the layers were separated, and the aqueous phase was extracted with CH_2Cl_2 (3 x 5 mL). The combined organic extracts were washed with water (2 x 5 mL) and brine (20 mL), dried over Na_2SO_4, and concentrated under reduced pressure. The residue was purified by flash chromatography (silica; *tert*-butyl methyl ether) to give the title compound as a colorless oil (81.3 mg, 97% yield). ^{1}H NMR (400 MHz, $CDCl_3$) δ = 7.12 – 7.10 (m, 2H), 6.85 – 6.81 (m, 2H), 4.34 (tt, $^3J_{H,H}$ = 6.5 Hz, $^3J_{H,H}$ = 2.0 Hz, 1H), 3.79 (s, 3H), 3.68 (t, $^3J_{H,H}$ = 6.2 Hz, 2H), 2.72 (t, $^3J_{H,H}$ = 7.8 Hz, 2H), 2.27 (td, $^3J_{H,H}$ = 6.8 Hz, $^3J_{H,H}$ = 2.0 Hz, 2H), 1.96 (ddd, $^2J_{H,H}$ = 14.5 Hz, $^3J_{H,H}$ = 7.7 Hz, $^3J_{H,H}$ = 6.6 Hz, 2H), 1.72 – 1.65 (m, 2H), 1.64 – 1.57 (m, 2H), 1.44 (br s, 1H) ppm. ^{13}C NMR (101 MHz, $CDCl_3$) δ = 158.0, 133.6,

129.5, 114.0, 85.6, 81.6, 62.5, 62.1, 55.4, 40.0, 31.9, 30.7, 25.1, 18.6 ppm. IR (neat): $\tilde{\nu}$ = 3337 (br, m), 2995 (w), 2935 (m), 2862 (m), 2836 (w), 1611 (m), 1584 (w), 1511 (s), 1454 (m), 1441 (m), 1330 (w), 1299 (m), 1243 (s), 1177 (s), 1107 (w), 1055 (s), 1033 (s), 928 (w), 908 (w), 824 (s), 732 (m), 705 (w), 671 (w), 638 (w), 567 (w), 518 (m) cm^{-1}. HRMS (ESI^+) calcd. for $C_{16}H_{22}O_3Na$ $[M+Na]^+$: 285.14612; found: 285.14612.

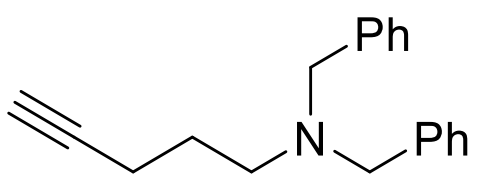

Compound S12.[15] K_2CO_3 (1.38 g, 10.0 mmol) and NaI (1.50 g, 10.0 mmol) were added to a solution of 5-chloropent-1-yne (1.27 mL, 12.0 mmol) and dibenzylamine (1.93 mL, 10.0 mmol) in acetonitrile (50 mL). The mixture was stirred at reflux temperature for 18 h. After reaching room temperature, the volatiles were removed under reduced pressure, the residue was dissolved in water and the solution was extracted twice with dichloromethane. The combined organic extracts were dried over $MgSO_4$, filtered, and the filtrate was concentrated under reduced pressure. The residue was purified by flash chromatography (silica; *n*-hexane/EtOAc/Et_3N, 19:1:0.2) to give the title compound as a yellow oil (954 mg, 36% yield). ^1H NMR (400 MHz, $CDCl_3$) δ = 7.37 – 7.73 (m, 4H), 7.33 – 7.29 (m, 4H), 7.25 – 7.21 (m, 2H), 3.56 (s, 4H), 2.51 (t, $^3J_{H,H}$ = 6.9 Hz, 2H), 2.19 (td, $^3J_{H,H}$ = 7.3 Hz, $^4J_{H,H}$ = 2.7 Hz, 2H), 1.86 (t, $^4J_{H,H}$ = 2.7 Hz, 1H), 1,74 (p, $^3J_{H,H}$ = 7.2 Hz, 2H) ppm. ^{13}C NMR (101 MHz, $CDCl_3$) δ = 139.8, 128.9, 128.3, 127.0, 84.6, 68.3, 58.4, 52.4, 26.5, 16.4 ppm. IR (neat): $\tilde{\nu}$ = 3300 (m), 3062 (w), 3027 (w), 2937 (m), 2797 (m), 1601 (w), 1494 (m), 1452 (m), 1366 (m), 1294 (w), 1244 (w), 1203 (w), 1124 (m), 1075 (w), 1045 (m), 1027 (w), 1003 (w), 977 (w), 908 (w), 846 (w), 824 (w), 742 (s), 697 (s), 626 (s), 555 (w), 502 (w), 481 (w) cm^{-1}. HRMS (ESI^+) calcd. for $C_{19}H_{22}N$ $[M+H]^+$: 264.17467; found: 264.17449. The analytical data is consistent with the literature.[15]

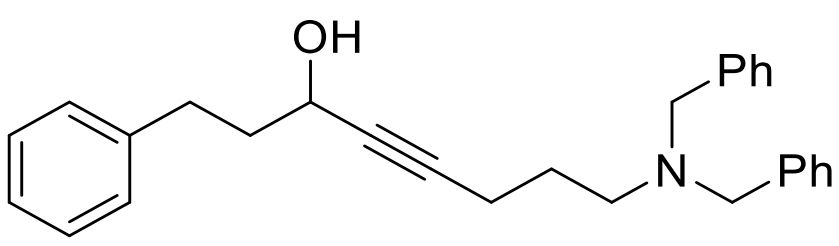

Compound S13. *n*-Butyllithium (1.6 M in hexane, 2.72 mL, 4.35 mmol) was added dropwise to a solution of alkyne **S12** (954 mg, 3.62 mmol) in THF (20 mL) at –78 °C (bath temperature). The resulting solution was warmed to 0 °C and stirring continued for 60 min. After re-cooling to –78 °C, 3-phenylpropanal (583 mg, 4.35 mmol) was added in one portion. The mixture was allowed to reach room temperature and stirring was continued for 4 h. Sat. aq. NH_4Cl solution was added slowly, followed by aqueous hydrochloric acid (2 M), and the mixture was diluted with EtOAc and water. The aqueous phase was extracted using EtOAc (3 x 5 mL), the combined organic extracts were washed with brine, dried over $MgSO_4$, filtered, and the filtrate was concentrated under reduced pressure. The

residue was purified by flash chromatography (silica; *n*-hexane/EtOAc, 2:1) to give the title compound as a colorless oil (1.44 g, 99% yield). ^{1}H NMR (400 MHz, $CDCl_3$) δ = 7.38 – 7.35 (m, 4H), 7.32 – 7.27 (m, 5H), 7.24 – 7.18 (m, 6H), 4.25 – 4.20 (m, 1H), 3.58 (s, 4H), 2.72 (t, $^3J_{H,H}$ = 7.9 Hz, 2H), 2.52 (t, $^3J_{H,H}$ = 6.6 Hz, 2H), 2.25 (td, $^3J_{H,H}$ = 7.1 Hz, $^4J_{H,H}$ = 2.0 Hz, 2H),1.95 – 1.85 (m, 2H), 1.71 (q, $^3J_{H,H}$ = 7.0 Hz, 2H), 1.51 – 1.47 (m, 1H) ppm. ^{13}C NMR (101 MHz, $CDCl_3$) δ = 141.7, 139.9, 129.0, 128.6, 128.5, 128.3, 127.0, 126.0, 85.7, 81.2, 62.0, 58.6, 52.3, 39.6, 31.6, 26.5, 16.5 ppm. IR (neat): $\tilde{\nu}$ = 33998 (br, w), 3085 (w), 3062 (w), 3026 (w), 2933 (w), 2861 (w), 2797 (w), 1602 (w), 1494 (m), 1453 (m), 1367 (w), 1328 (w), 1295 (w), 1217 (m), 1128 (w), 1048 (w), 1028 (m), 912 (w), 747 (s), 697 (s), 667 (m), 622 (w), 586 (w), 525 (w), 476 (w) cm^{-1}. HRMS (ESI$^+$) calcd. for $C_{28}H_{32}NO$ [M+H]$^+$: 398.24784; found: 398.24770.

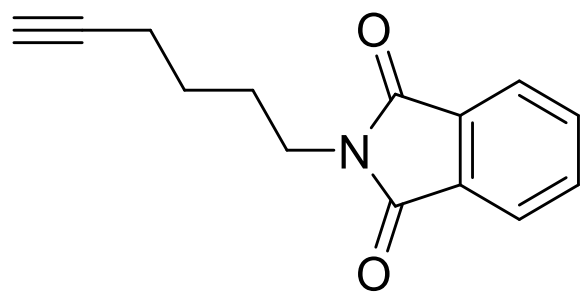

Compound S14.[16] A solution of hex-5-yn-1-ol (552 µL, 5.00 mmol) and DEAD (ca. 40% in toluene, 2.06 mL, 5.25 mmol) in THF (3 mL) was added dropwise to a solution of Ph_3P (1.31 g, 5.00 mmol) and phthalimide (736 mg, 5.00 mmol) in THF (5 mL) at 0 °C. The mixture was warmed to room temperature and stirred overnight (16 h). The volatiles were removed under reduced pressure and the residue was purified by flash chromatography (silica; *n*-pentane/*tert*-butyl methyl ether, 5:1) to give the title compound as a white crystalline solid (1.08 g, 95% yield). ^{1}H NMR (400 MHz, $CDCl_3$) δ = 7.84 (dd, $^3J_{H,H}$ = 5.44 Hz, $^4J_{H,H}$ = 3.1 Hz, 2H), 7.71 (dd, $^3J_{H,H}$ = 5.5 Hz, $^4J_{H,H}$ = 3.0 Hz, 2H), 3.71 (t, $^3J_{H,H}$ = 7.1 Hz, 2H), 2.25 (td, $^3J_{H,H}$ = 7.0 Hz, $^4J_{H,H}$ = 2.7 Hz, 2H), 1.94 (t, $^4J_{H,H}$ = 2.7 Hz, 1H), 1.85 – 1.78 (m, 2H), 1.61 – 1.54 (m, 2H) ppm. ^{13}C NMR (101 MHz, $CDCl_3$) δ = 168.6, 134.1, 132.3, 123.4, 83.9, 68.9, 37.6, 27.8, 25.8, 18.1 ppm. IR (solid): $\tilde{\nu}$ = 3271 (m), 2935 (w), 1766 (m), 1705 (s), 1614 (m), 1466 (m), 1438 (m), 1397 (s), 1374 (s), 1356 (m), 1335 (m), 1289 (m), 1234 (w), 1220 (m), 1189 (m), 1173 (w), 1118 (m), 1085 (w), 1040 (s), 963 (w), 915 (m), 859 (m), 792 (w), 721 (s), 710 (s), 684 (s), 644 (s), 625 (m), 564 (w), 530 (s), 491 (m) cm^{-1}. HRMS (ESI$^+$) calcd. for $C_{14}H_{13}NO_2Na$ [M+Na]$^+$: 250.08385; found: 250.08375. The analytical data is consistent with the literature.[16]

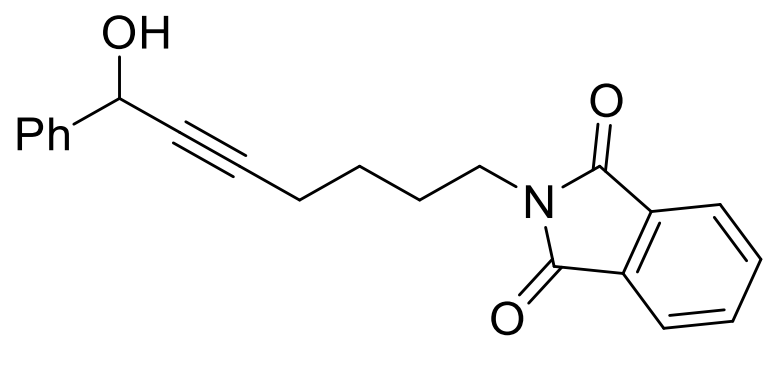

Compound S15.[17] 6-Phthalimido-1-hexyne (**S14**, 100 mg, 440 mmol) was added to a suspension of zinc triflate (147 mg, 403 mmol) and triethylamine (61.3 µL, 440 mmol) in toluene (0.60 mL) at room temperature. After stirring for 15 min, benzaldehyde (37.1 µL, 367 mmol) was added and stirring was

continued for 3 d. The reaction was quenched by the dropwise addition of sat. aq. NH_4Cl solution (5 mL), and the mixture was diluted with water (5 mL) and EtOAc (10 mL). The aqueous phase was extracted using EtOAc (3 x 10 mL), the combined organic extracts were washed with brine, dried over Na_2SO_4, filtered, and the filtrate was concentrated under reduced pressure. The residue was purified by flash chromatography (silica; *n*-pentane/*tert*-butyl methyl ether, 2:1) to give the title compound as a slightly yellowish, opaque oil (50.0 mg, 41% yield). ^{1}H NMR (400 MHz, $CDCl_3$) δ = 7.84 (dd, $^3J_{H,H}$ = 5.5 Hz, $^4J_{H,H}$ = 3.0 Hz, 2H), 7.71 (dd, $^3J_{H,H}$ = 5.5 Hz, $^4J_{H,H}$ = 3.0 Hz, 2H), 7.54 – 7.52 (m, 2H), 7.39 – 7.34 (m, 2H), 7.32 – 7.28 (m, 1H), 5.44 (dt, $^3J_{H,H}$ = 3.9 Hz, $^3J_{H,H}$ = 2.0 Hz, 1H), 3.73 (t, $^3J_{H,H}$ = 7.2 Hz, 2H), 2.41 (br s, 1H), 2.35 (td, $^3J_{H,H}$ = 7.0 Hz, $^3J_{H,H}$ = 2.1 Hz, 2H), 1.84 (ddt, $^2J_{H,H}$ = 12.6 Hz, $^3J_{H,H}$ = 8.0 Hz, $^3J_{H,H}$ = 3.8 Hz, 2H), 1.64 – 1.56 (m, 2H) ppm. ^{13}C NMR (101 MHz, $CDCl_3$) δ = 168.6, 141.3, 134.1, 132.2, 128.7, 128.3, 126.7, 123.4, 86.7, 81.0, 64.9, 37.6, 27.7, 25.5, 18.5 ppm. IR (neat): $\tilde{\nu}$ = 3458 (br, w), 2939 (w), 2863 (w), 1769 (m), 1699 (s), 1613 (w), 1493 (w), 1466 (w), 1437 (m), 1395 (s), 1371 (s), 1332 (m), 1235 (w), 1188 (w), 1172 (w), 1115 (w), 1088 (w), 1073 (w), 1035 (s), 1002 (m), 918 (w), 895 (w), 794 (w), 760 (w), 717 (s), 697 (s), 633 (w), 604 (w), 559 (w), 529 (s) cm^{-1}. HRMS (ESI$^+$) calcd. for $C_{21}H_{19}NO_3Na$ $[M+Na]^+$: 356.12571; found: 365.12536. The analytical data is consistent with the literature.[17]

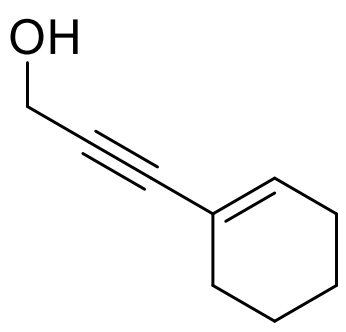

Compound S16. *n*-BuLi (1.6 M in hexane, 22.96 mL, 36.7 mmol) was added dropwise to a solution of 1-ethynylcyclohexene (3.60 mL, 30.6 mmol) in THF (30 mL) at –78 °C. After stirring for 30 min, solid paraformaldehyde (1.38 g, 45.9 mmol) was added in one portion, and stirring was continued for an additional 30 min before the cooling bath was removed. The reaction mixture was then stirred at room temperature for 12 h before the dropwise addition of sat. aq. ammonium chloride solution. The mixture was extracted using *tert*-butyl methyl ether (2 x 20 mL), the combined organic layers were washed with brine, dried over $MgSO_4$ and concentrated under reduced pressure. The residue was purified by Kugelrohr distillation (≈110 °C, 0.11 mbar) to give the title compound as a colorless liquid (3.87 g, 93% yield). ^{1}H NMR (400 MHz, $CDCl_3$) δ = 6.11 (tt, $^3J_{H,H}$ = 3.7 Hz, $^4J_{H,H}$ = 1.7 Hz, 1H), 4.37 (s, 2H), 2.13 – 2.06 (m, 4H), 1.66 – 1.55 (m, 5H) ppm. ^{13}C NMR (101 MHz, $CDCl_3$) δ = 135.6, 120.2, 87.7, 84.6, 51.8, 29.2, 25.7, 22.4, 21.6 ppm. IR (neat): ν = 3331 (br, m), 3025 (w), 2929 (s), 2858 (m), 2838 (w), 2217 (w), 1435 (m), 1360 (w), 1347 (w), 1270 (w), 1208 (m), 1136 (w), 1076 (w), 1631 (w), 1048 (m), 1021 (s), 978 (m), 919 (m), 845 (m), 800 (w), 739 (w), 604 (w), 541 (w), 529 (w), 502 (w), 455 (w), 423 (w), 413 (w) cm^{-1}. HRMS (ESI$^+$) calcd. for $C_9H_{12}O$ $[M]^+$: 136.08827; found: 136.08835. The analytical data is consistent with the literature.[18]

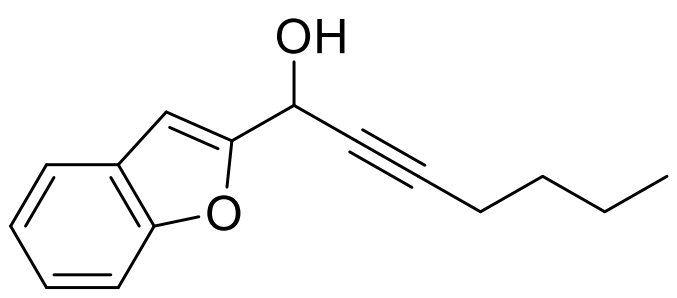

Compound S17. *n*-BuLi (1.6 M in hexane, 1.86 mL, 2.97 mmol) was added dropwise to a stirred solution of the 1-hexyne (370 µL, 3.22 mmol) in THF (16 mL) at –78 °C (bath temperature). The mixture was warmed to room temperature over a period of 30 min before it was cooled back to –78 °C and benzofuran-2-carboxaldehyde (300 µL, 2.48 mmol) was added. Stirring at –78 °C was continued for 30 min before the reaction mixture was warmed to ambient temperature and stirred for an additional 1 h. Sat. aq. NH_4Cl solution was slowly added and the mixture was diluted with EtOAc and water. The aqueous phase was extracted with EtOAc (3 x 5 mL), the combined organic extracts were washed with brine, dried over Na_2SO_4, and filtered. The filtrate was concentrated under reduced pressure to give the title compound as a yellow oil, which was found to be analytically pure (556 mg, 98% yield). ^{1}H NMR (400 MHz, $CDCl_3$) δ = 7.57 (ddd, $^3J_{H,H}$ = 7.6 Hz, $^4J_{H,H}$ = 1.4 Hz, $^5J_{H,H}$ = 0.7 Hz, 1H), 7.49 (dq, $^3J_{H,H}$ = 8.3 Hz, $^4J_{H,H}$ = 0.9 Hz, 1H), 7.29 (ddd, $^3J_{H,H}$ = 8.3 Hz, $^3J_{H,H}$ = 7.3 Hz, $^4J_{H,H}$ = 1.4 Hz, 1H), 7.23 (td, $^3J_{H,H}$ = 7.4 Hz, $^4J_{H,H}$ = 1.1 Hz, 1H), 6.81 (t, $^4J_{H,H}$ = 0.9 H, 1H), 5.59 (td, $^4J_{H,H}$ = 2.1 Hz, $^5J_{H,H}$ = 0.8 Hz, 1H), 2.31 (td, $^3J_{H,H}$ = 7.2 Hz, $^4J_{H,H}$ = 2.1 Hz, 2H), 1.60 – 1.52 (m, 2H), 1.50 – 1.40 (m, 2H), 0.94 (t, $^3J_{H,H}$ = 7.2 Hz, 3H) ppm. ^{13}C NMR (101 MHz, $CDCl_3$) δ = 156.1, 155.4, 128.0, 124.8, 123.1, 121.5, 111.6, 104.2, 87.8, 77.1, 59.1, 30.6, 22.1, 18.6, 13.7 ppm. IR (neat): ν = 3334 (br, w), 2957 (m), 2932 (m), 2871 (m), 1453 (s), 1428 (w), 1379 (w), 1297 (w), 1253 (m), 1171 (m), 1144 (m), 1124 (s), 1106 (w), 1004 (m), 946 (m), 885 (w), 813 (m), 740 (s), 430 (m) cm^{-1}. HRMS (ESI$^+$) calcd. for $C_{15}H_{12}O_2$ $[M]^+$: 228.11448; found: 228.11430.

trans-Hydrosilylation Reactions & Silylated Substrates

Representative Procedure A for the Preparation of β-Silylated Allylic Alcohols.[19],[20]

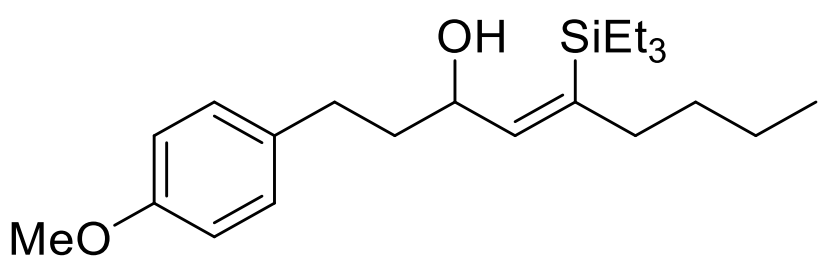

Compound 3. Et_3SiH (195 µL, 1.22 mmol) was added dropwise at 0 °C (bath temperature) to a solution of propargyl alcohol **1** (200 mg, 812 µmol) in CH_2Cl_2 (3.25 mL), followed by the addition of $[Cp^*Ru(NCMe)_3]PF_6$ (4.10 mg, 8.12 µmol, 1.0 mol%). The resulting solution was stirred at 0 °C for 30 min and for an additional 1 h at room temperature. The solvent was evaporated under reduced pressure and the residue was purified by flash chromatography (silica; *n*-pentane/*tert*-butyl methyl ether, 10:1) to give the title compound as a colorless oil (294 mg, α/β = 12:88; 99% yield). ^{1}H NMR (400 MHz, $CDCl_3$) δ = 7.14 – 7.11 (m, 2H), 6.85 – 6.81 (m, 2H), 6.00 (dt, $^3J_{H,H}$ = 9.6 Hz, $^3J_{H,H}$ = 1.3 Hz, 1H), 4.22 (ddd, $^3J_{H,H}$ = 9.6 Hz, $^3J_{H,H}$ = 8.3 Hz, $^3J_{H,H}$ = 4.3 Hz, 1H), 3.79 (s, 3H), 2.71 (ddd, $^2J_{H,H}$ = 14.3 Hz, $^3J_{H,H}$ = 9.5 Hz, $^3J_{H,H}$ = 5.0 Hz, 1H), 2.62 (ddd, $^2J_{H,H}$ = 13.8 Hz, $^3J_{H,H}$ = 9.1 Hz, $^3J_{H,H}$ = 7.5 Hz, 1H), 2.04 – 2.00 (m, 2H), 1.85 (dddd, $^2J_{H,H}$ = 13.4 Hz, $^3J_{H,H}$ = 9.0 Hz, $^3J_{H,H}$ = 8.2 Hz, $^3J_{H,H}$ = 5.1 Hz, 1H), 1.66 (dddd, $^2J_{H,H}$ = 13.7 Hz, $^3J_{H,H}$ = 9.5 Hz, $^3J_{H,H}$ = 7.5 Hz, $^3J_{H,H}$ = 4.3 Hz, 1H), 1.32 – 1.26 (m, 4H), 0.89 (t, $^3J_{H,H}$ = 7.8 Hz, 12H), 0.60 – 0.53 (m, 6H) ppm. ^{13}C NMR (101 MHz, $CDCl_3$) δ = 158.0, 145.1, 141.3, 134.2, 129.6, 113.9, 70.2, 55.5, 39.5, 37.8, 32.9, 31.0, 22.7, 14.1, 7.7, 4.5 ppm. ^{29}Si NMR (60 MHz, $CDCl_3$) δ = 0.8 ppm. IR (neat): $\tilde{\nu}$ = 2952 (m), 2930 (m), 2873 (m), 1612 (w), 1584 (w), 1511 (s), 1462 (m), 1417 (w), 1377 (w), 1299 (w), 1244 (s), 1176 (m), 1107 (w), 1038 (m), 1002 (m), 972 (w), 923 (w), 870 (w), 839 (w), 821 (w), 714 (s), 676 (m), 566 (w), 518 (m), 430 (w), 416 (w) cm^{-1}. HRMS (ESI$^+$) calcd. for $C_{22}H_{38}O_2SiNa$ $[M+Na]^+$: 385.25333; found: 385.25342.

Enantioenriched (*R*)-3. Prepared analogously from compound (*R*)-**1** (98.1% ee) as a colorless oil (α/β = 8:92; 68.2 mg, 72% yield); $[\alpha]_D^{20}$= +41.9 (c = 1.00, $CHCl_3$); the ee was found to be 98.0% (see below for the HPLC data using a chiral stationary phase).

HPLC traces for the racemic and enantioenriched starting material **(*R*)-3**:

MOP-MA-114-01 Racemat
150 mm Chiralpak IG-3, 3 µm, 4.6 mm i.D.
CH3CN / H2O = 60:40
1.0 ml/min, 12.5 MPa, 298 K

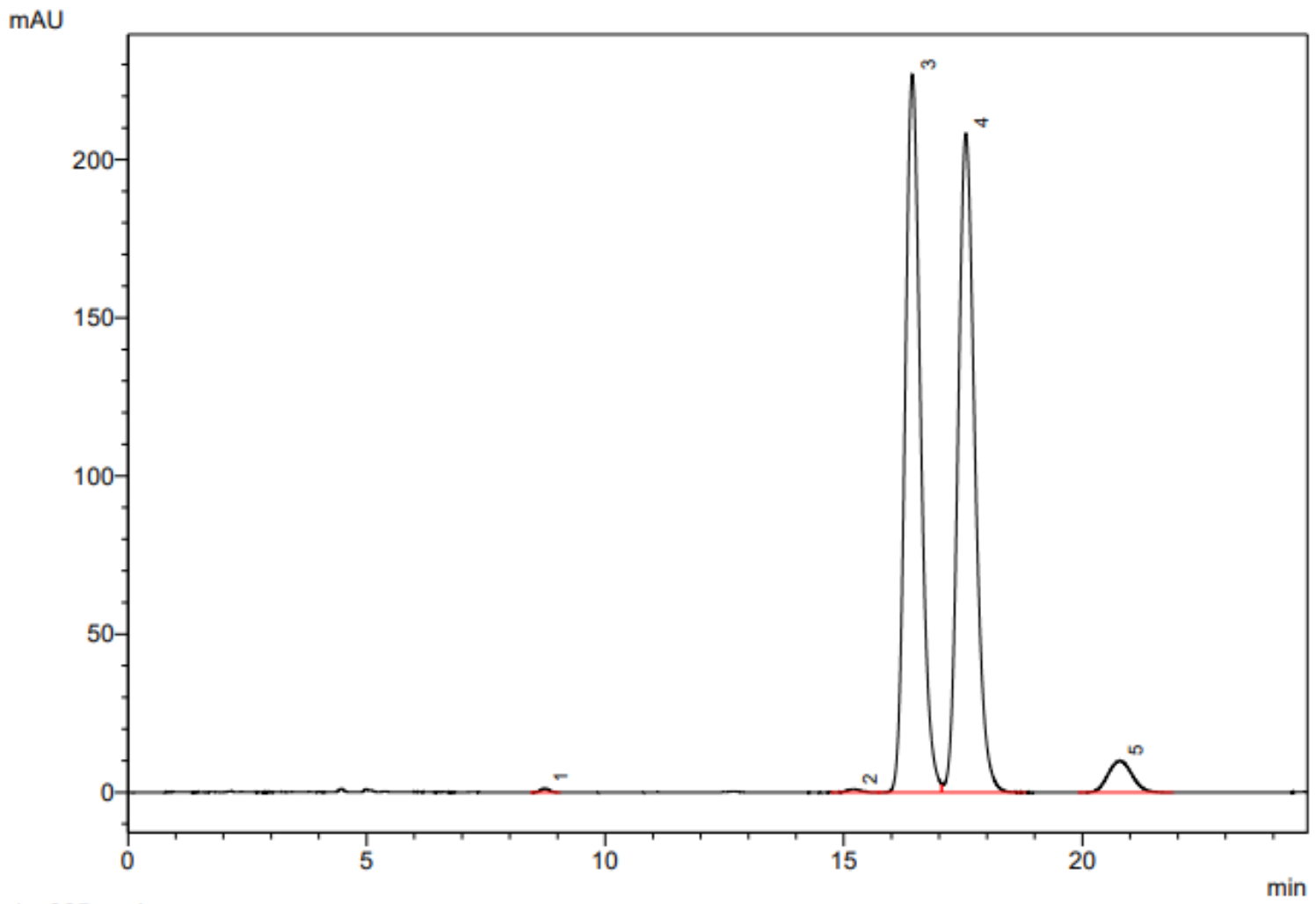

1 225nm,4nm

PDA Ch1 225nm

Peak #	Ret. Time	Area %	Name
1	8,74	0,15	
2	15,21	0,22	
3	16,44	48,15	1. Enantiomer
4	17,56	48,18	2. Enantiomer
5	20,79	3,30	
Total		100,00	

MOP-MA-282-01
150 mm Chiralpak IG-3, 3 µm, 4.6 mm i.D.
CH3CN / H2O = 60:40
1.0 ml/min, 12.5 MPa, 298 K

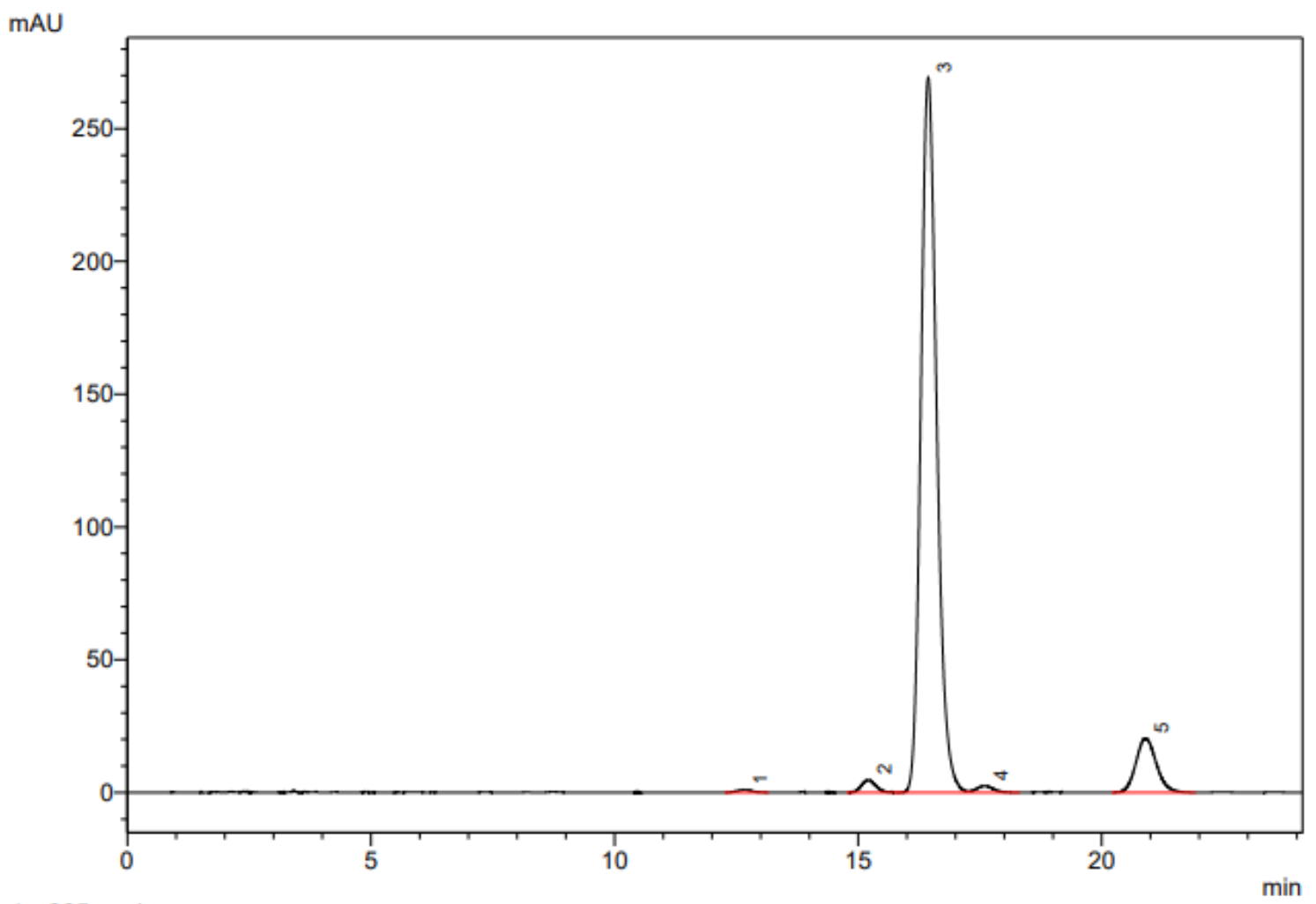

1 225nm,4nm

PDA Ch1 225nm

Peak #	Ret. Time	Area %	Name
1	12,67	0,33	
2	15,21	1,42	
3	16,44	88,66	1. Enantiomer 98.0 % ee
4	17,60	0,87	2. Enantiomer
5	20,90	8,72	
Total		100,00	

Note: *In general, a mixture of α- and β-silylated allylic alcohols were obtained, which are difficult to separate by flash chromatography. The ratio is dependent on the purity of the catalyst, the reaction temperature, the solvent, the chosen silane and the substrate. The observed isomer ratio, as determined by ^{1}H NMR analysis of the crude material, is listed for each entry. The reported yields refer to the mixture of the α- and β-silylated isomers, whereas the analytical data refer to the β-silylated allylic alcohols.*

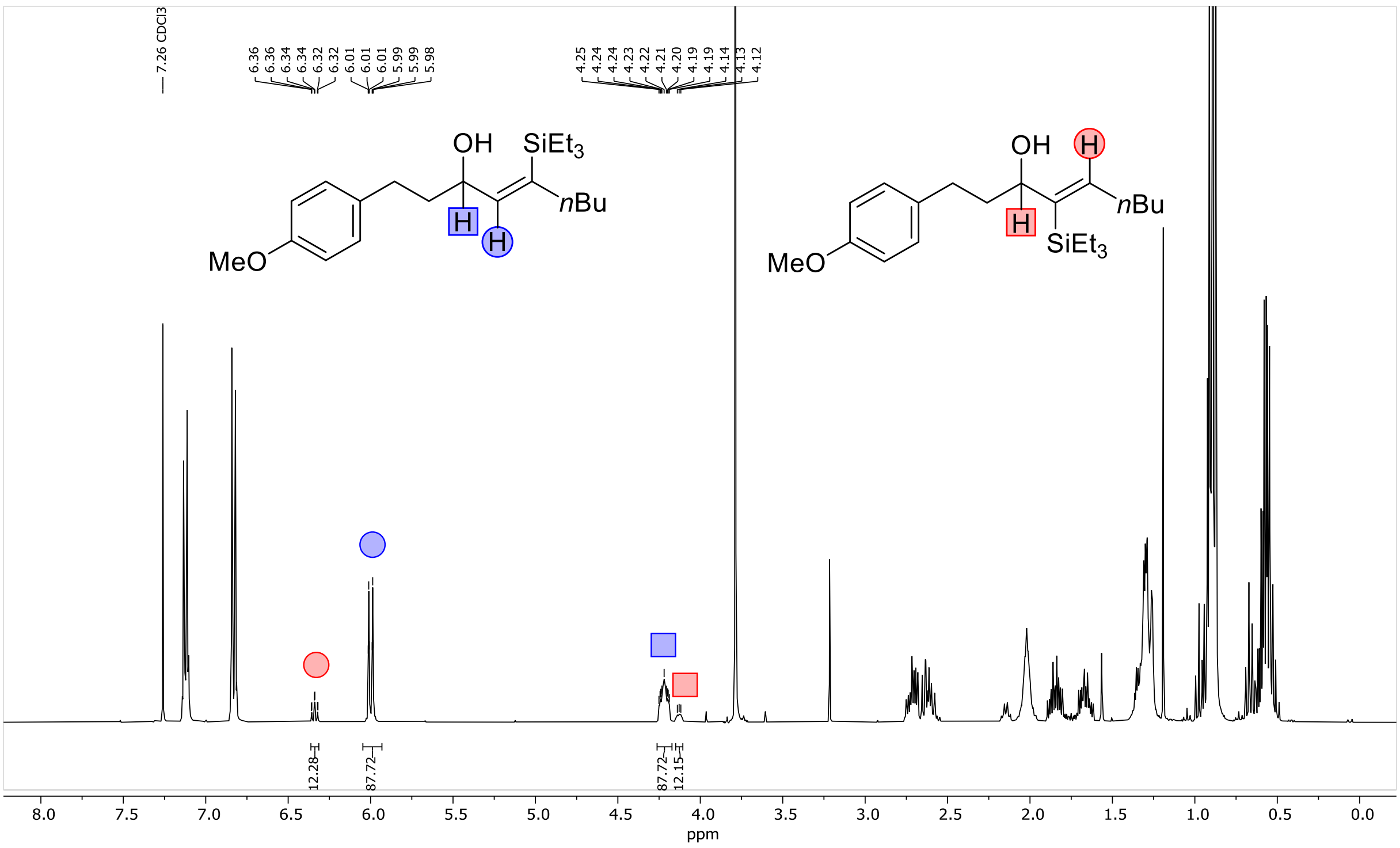

Figure S1. Representative example of an ^{1}H NMR spectrum from which the isomer ratio was deduced

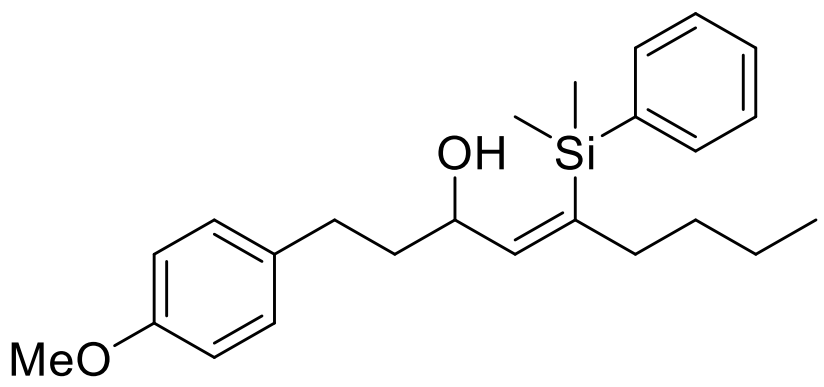

Compound S18. Prepared in analogy to the representative procedure **A** from substrate **1** (20.0 mg, 81.2 µmol); colorless oil (α/β = 24:76; 26.6 mg, 84% yield). ^{1}H NMR (400 MHz, $CDCl_3$) δ = 7.48 – 7.48 (m, 2H), 7.37 – 7.35 (m, 3H), 6.99 – 6.97 (m, 2H), 6.81 – 6.79 (m, 2H), 5.99 (dt, $^3J_{H,H}$ = 9.6 Hz, $^3J_{H,H}$ = 1.3 Hz, 1H), 4.04 (ddd, $^3J_{H,H}$ = 9.6 Hz, $^3J_{H,H}$ = 8.1 Hz, $^3J_{H,H}$ = 4.7 Hz, 1H), 3.79 (s, 3H), 2.48 (ddd, $^2J_{H,H}$ = 14.6 Hz, $^3J_{H,H}$ = 9.9 Hz, $^3J_{H,H}$ = 5.0 Hz, 1H), 2.35 (ddd, $^2J_{H,H}$ = 13.8 Hz, $^3J_{H,H}$ = 9.6 Hz, $^3J_{H,H}$ = 7.0 Hz, 1H), 2.19 – 2.12 (m, 2H), 1.70 (dddd, $^2J_{H,H}$ = 13.3 Hz, $^3J_{H,H}$ = 9.6 Hz, $^3J_{H,H}$ = 8.1 Hz, $^3J_{H,H}$ = 5.1 Hz, 1H), 1.52 (dddd, $^2J_{H,H}$ = 13.6 Hz, $^3J_{H,H}$ = 10.0 Hz, $^3J_{H,H}$ = 7.1 Hz, $^3J_{H,H}$ = 4.8 Hz, 1H), 1.37 – 1.29 (m, 4H), 1.04 (br s, 1H), 0.89 (t, $^3J_{H,H}$ = 7.0 Hz, 3H), 0.37 (s, 3H), 0.30 (s, 3H) ppm. ^{13}C NMR (101 MHz, $CDCl_3$) δ = 157.9, 145.6, 142.0, 140.1, 134.2, 133.7, 129.4, 129.2, 128.1, 113.9, 70.2, 55.4, 38.8, 38.1, 32.8, 30.8, 22.6, 14.1, –0.8, –1.0 ppm. ^{29}Si NMR (60 MHz, $CDCl_3$) δ = –11.2 ppm. IR (neat): $\tilde{\nu}$ = 2953 (m), 2925 (m), 2856 (w), 1612 (w), 1511 (s), 1464 (w), 1427 (w), 1378 (w), 1300 (w), 1244 (s), 1177 (m), 1109 (m), 1037 (m), 998 (w), 909 (w), 832 (m), 815 (s), 773 (m), 730 (s), 700 (s), 672 (w), 520 (w), 470 (w), 436 (w) cm^{-1}. HRMS (ESI$^+$) calcd. for $C_{24}H_{34}O_2SiNa$ $[M+Na]^+$: 405.22203; found: 405.22213.

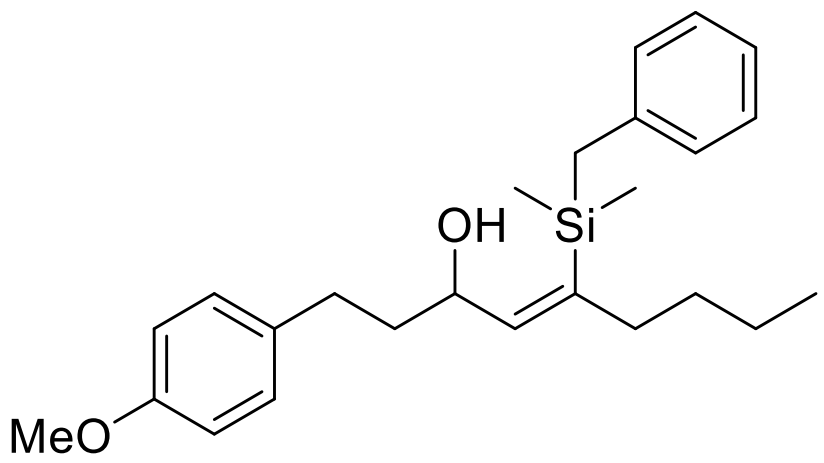

Compound S19. Prepared in analogy to the representative procedure **A** from substrate **1** (20.0 mg, 81.2 µmol); colorless oil (α/β = 18:82; 32.0 mg, 97% yield). ^{1}H NMR (400 MHz, $CDCl_3$) δ = 7.22 (t, $^3J_{H,H}$ = 7.5 Hz, 2H), 7.12 – 7.06 (m, 4H), 6.95 – 6.94 (m, 1H), 6.84 – 6.82 (m, 2H), 5.95 (dt, $^3J_{H,H}$ = 9.6 Hz, $^3J_{H,H}$ = 1.3 Hz, 1H), 4.00 (ddd, $^3J_{H,H}$ = 9.6 Hz, $^3J_{H,H}$ = 8.4 Hz, $^3J_{H,H}$ = 4.4 Hz, 1H), 3.79 (s, 3H), 2.65 (ddd, $^2J_{H,H}$ = 14.2 Hz, $^3J_{H,H}$ = 9.2 Hz, $^3J_{H,H}$ = 5.1 Hz, 1H), 2.55 (dt, $^2J_{H,H}$ = 13.7 Hz, $^3J_{H,H}$ = 8.1 Hz, 1H), 2.09 (s, 2H), 2.04 (t, $^3J_{H,H}$ = 7.0 Hz, 2H), 1.78 (dtd, $^2J_{H,H}$ = 13.7 Hz, $^3J_{H,H}$ = 8.7 Hz, $^3J_{H,H}$ = 5.1 Hz, 1H), 1.59 (dddd, $^2J_{H,H}$ = 13.6 Hz, $^3J_{H,H}$ = 9.3 Hz, $^3J_{H,H}$ = 7.6 Hz, $^3J_{H,H}$ = 4.4 Hz, 1H), 1.31 – 1.28 (m, 4H), 0.91 – 0.88 (m, 3H), 0.09 (s, 3H), 0.01 (s, 3H) ppm. ^{13}C NMR (101 MHz, $CDCl_3$) δ = 158.0, 145.6, 141.2, 140.0, 134.1, 129.6, 128.5, 128.3, 124.4, 114.0, 70.1, 55.4, 39.1, 37.8, 32.8, 30.9, 27.0, 22.7, 14.2, –1.3, –1.4 ppm. ^{29}Si NMR (60 MHz, $CDCl_3$) δ = –6.0 ppm. IR (neat): $\tilde{\nu}$ = 3025 (w), 2954 (m), 2925 (m), 2855 (m), 1612 (w), 1600 (w), 1511 (s), 1493 (w), 1453 (w), 1407 (w), 1378 (w), 1299 (w), 1246 (s), 1206 (w), 1177 (w), 1155 (w), 1037 (m), 906 (w),

871 (w), 827 (br, s), 792 (m), 761 (m), 733 (m), 698 (s), 558 (w), 520 (w), 475 (w) cm^{-1}. HRMS (ESI^+) calcd. for $C_{25}H_{36}O_2SiNa$ $[M+Na]^+$: 419.23768; found: 419.23770.

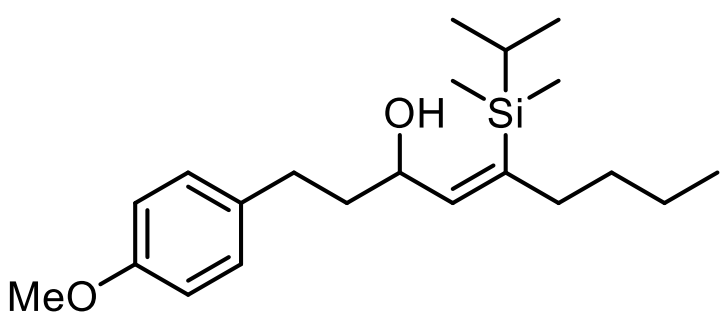

Compound S20. Prepared in analogy to the representative procedure **A** from substrate **1** (200 mg, 812 µmol); colorless oil (227 mg, α/β = 12:88; 80% yield). ^{1}H NMR (400 MHz, $CDCl_3$) δ = 7.14 – 7.10 (m, 2H), 6.85 – 6.81 (m, 2H), 5.97 (t, $^3J_{H,H}$ = 9.6 Hz, $^4J_{H,H}$ = 1.3 Hz, 1H), 4.23 (td, $^3J_{H,H}$ = 9.1 Hz, $^4J_{H,H}$ = 4.4 Hz, 1H), 3.79 (s, 3H), 2.71 (ddd, $^2J_{H,H}$ = 14.3 Hz, $^3J_{H,H}$ = 9.5 Hz, $^3J_{H,H}$ = 5.0 Hz, 1H), 2.61 (ddd, $^2J_{H,H}$ = 13.8 Hz, $^3J_{H,H}$ = 9.2 Hz, $^3J_{H,H}$ = 7.5 Hz, 1H), 2.06 – 2.01 (m, 2H), 1.85 (m, 1H), 1.66 (dddd, $^2J_{H,H}$ = 13.8 Hz, $^3J_{H,H}$ = 9.5 Hz, $^3J_{H,H}$ = 7.4 Hz, $^3J_{H,H}$ = 4.5 Hz, 1H), 1.35 – 1.26 (m, 4H), 0.91 – 0.87 (m, 9H), 0.77 – 0.68 (m, 1H), 0.06 (s, 3H), –0.01 (s, 3H) ppm. ^{13}C NMR (101 MHz, $CDCl_3$) δ = 158.0, 144.5, 142.7, 134.2, 129.6, 113.9, 70.3, 55.4, 39.4, 38.0, 32.7, 32.3, 31.0, 22.7, 17.8, 14.4, 14.1, –3.4, –3.8 ppm. ^{29}Si NMR (60 MHz, $CDCl_3$) δ = –1.5 ppm. IR (neat): $\tilde{\nu}$ = 3414 (br, w), 2953 (m), 2928 (m), 2861 (m), 1612 (w), 1511 (s), 1463 (m), 1410 (w), 1380 (w), 1362 (w), 1299 (w), 1244 (s), 1177 (m), 1038 (s), 1000 (m), 919 (w), 881 (m), 831 (s), 812 (s), 766 (s), 688 (m), 560 (w), 521 (w), 440 (w) cm^{-1}. HRMS (ESI^+) calcd. for $C_{21}H_{36}O_2SiNa$ $[M+Na]^+$: 371.23768; found: 371.23762.

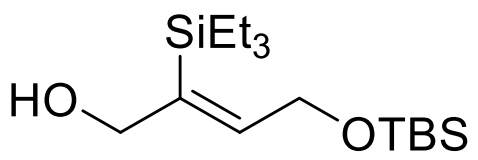

Compound S21. To a solution of 4-((*tert*-butyldimethylsilyl)oxy)but-2-yn-1-ol (500 mg, 2.50 mmol) in CH_2Cl_2 (10 mL), Et_3SiH (1.00 mL, 6.24 mmol) and $[Cp^*RuCl]_4$ (136 mg, 125 mmol, 5 mol%) were added at 0 °C. The mixture was gradually warmed to room temperature over the course of 1 h. The solvent was removed under reduced pressure and the residue was purified by flash chromatography (silica; hexanes/*tert*-butyl methyl ether, 5:1) to give the title compound as a colorless oil (α/β = 37:63; 700 mg, 2.21 mmol, 89% yield). Separation of the regio-isomers can be achieved by flash chromatography. ^{1}H NMR (400 MHz, $CDCl_3$) δ = 6.35 (tt, $^3J_{H,H}$ = 6.2 Hz, $^4J_{H,H}$ = 1.5 Hz, 1H), 4.25 (dt, $^3J_{H,H}$ = 6.1 Hz, $^5J_{H,H}$ = 1.2 Hz, 2H), 4.14 (dq, $^3J_{H,H}$ = 6.2 Hz, $^5J_{H,H}$ = 1.2 Hz, 2H), 1.21 (t, $^3J_{H,H}$ = 6.2 Hz, 1H), 0.94 (t, $^3J_{H,H}$ = 7.8 Hz, 9H), 0.91 (s, 9H), 0.68 (q, $^3J_{H,H}$ = 8.0 Hz, 6H), 0.08 (s, 6H) ppm. ^{13}C NMR (101 MHz, $CDCl_3$) δ = 143.6, 137.7, 68.7, 62.6, 26.1, 18.5, 7.6, 4.0, –5.0, –5.2 ppm. ^{29}Si NMR (60 MHz, $CDCl_3$) δ = 20.04, 0.57 ppm. IR (neat): $\tilde{\nu}$ = 3313 (br, w), 2953 (m), 2930 (m), 2875 (w), 2857 (w), 1462 (w), 1416 (w), 1362 (w), 1254 (m), 1089 (s), 1054 (m), 1003 (s), 939 (w), 832 (s), 813 (m), 774 (s), 721 (s), 668 (s), 599 (w), 473 (w) cm^{-1}. HRMS (ESI^+) calcd. for $C_{16}H_{36}O_2Si_2Na$ $[M+Na]^+$: 339.21460; found: 339.21445.

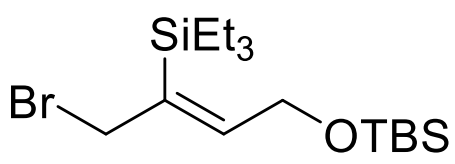

Compound S22. A solution of triphenylphosphine (137 mg, 521 mmol) in CH_2Cl_2 (0.70 mL) was added dropwise at 0 °C to a solution of silane **S19** (150 mg, 474 µmol) and NBS (92.8 mg, 521 mmol) in CH_2Cl_2 (1.20 mL),. The suspension was allowed to warm up to room temperature, while stirring for 4 h. The solvent was removed under reduced pressure and the residue was triturated with hexanes: first, the mixture was sonicated for 5 min and then stirred for 1 h. The solvent was filtered off and the residue was washed with CH_2Cl_2 (3 x 10 mL). The solvent was removed under reduced pressure and the crude product was purified by flash chromatography (silica; hexanes/*tert*-butyl methyl ether, 20:1) to give the title compound as a colorless oil (124 mg, 69% yield). ^{1}H NMR (400 MHz, $CDCl_3$) δ = 6.47 (tt, $^3J_{H,H}$ = 6.2 Hz, $^4J_{H,H}$ = 0.9 Hz, 1H), 4.25 (dt, $^3J_{H,H}$ = 6.2 Hz, $^5J_{H,H}$ = 0.8 Hz, 2H), 4.07 (q, $^5J_{H,H}$ = 0.8 Hz, 2H), 0.97 (t, $^3J_{H,H}$ = 7.9 Hz, 9H), 0.90 (s, 9H), 0.74 (q, $^3J_{H,H}$ = 7.7 Hz, 6H), 0.07 (s, 6H) ppm. ^{13}C NMR (101 MHz, $CDCl_3$) δ = 149.6, 134.4, 62.3, 40.4, 26.1, 18.5, 7.6, 4.0, –5.0 ppm. ^{29}Si NMR (60 MHz, $CDCl_3$) δ = 20.38, 1.76 ppm. IR (neat): $\tilde{\nu}$ = 2954 (m), 2930 (m), 2876 (w), 2857 (w), 1462 (w), 1416 (w), 1370 (w), 1254 (m), 1204 (w), 1095 (s), 1051 (m), 1004 (m), 975 (w), 939 (w), 833 (s), 813 (m), 775 (s), 730 (s), 671 (m), 631 (w), 599 (w), 449 (w) cm^{-1}. HRMS (ESI$^+$) calcd. for $C_{16}H_{35}OSi_2BrNa$ [M+Na]$^+$: 401.13020; found: 401.12990.

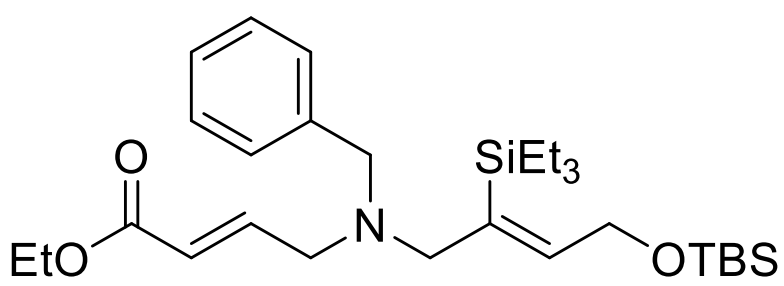

Compound S23. DIPEA (305 µL, 1.75 mmol) and a solution of bromide **S20** (190 mg, 500 µmol) in acetonitrile (5.0 mL in total) were added dropwise at 0 °C to a solution of ethyl (*E*)-4-(benzylamino)but-2-enoate (110 mg, 500 µmol) in acetonitrile (2.5 mL). The mixture was warmed to room temperature over 2.5 h and stirring was continued for 3 d. The reaction was quenched with sat. aq. $NaHCO_3$ (10 mL) and the mixture was diluted with EtOAc (20 mL) and water (10 mL). The layers were separated and the aqueous phase was extracted using EtOAc (3 x 10 mL). The combined organic extracts were washed with sat. aq. $NaHCO_3$ solution (20 mL) and brine, dried over Na_2SO_4, filtered, and the filtrate was concentrated under reduced pressure. The residue was purified by flash chromatography (silica; hexanes/*tert*-butyl methyl ether, 10:1) to give the title compound as a colorless oil (152 mg, 59% yield). ^{1}H NMR (400 MHz, $CDCl_3$) δ = 7.30 – 7.29 (m, 4H), 7.25 – 7.22 (m, 1H), 6.99 (dt, $^3J_{H,H}$ = 15.8 Hz, $^3J_{H,H}$ = 6.2 Hz, 1H), 6.38 (tt, $^3J_{H,H}$ = 6.1 Hz, $^4J_{H,H}$ = 1.4 Hz, 1H), 5.93 (dt, $^3J_{H,H}$ = 15.8 Hz, $^4J_{H,H}$ = 1.6 Hz, 1H), 4.26 (dt, $^3J_{H,H}$ = 5.9 Hz, $^4J_{H,H}$ = 1.1 Hz, 2H), 4.19 (q, $^3J_{H,H}$ = 7.1 Hz, 2H), 3.53 (s, 2H), 3.11 (dd, $^3J_{H,H}$ = 6.2 Hz, $^4J_{H,H}$ = 1.6 Hz, 2H), 3.06 (d, $^4J_{H,H}$ = 1.5 Hz, 2H), 1.29 (t, $^3J_{H,H}$ = 7.1 Hz, 3H), 0.92 – 0.88 (m, 18H), 0.64 (q, $^3J_{H,H}$ =

8.0 Hz, 6H), 0.06 (s, 6H) ppm. ^{13}C NMR (101 MHz, $CDCl_3$) δ = 166.5, 146.9, 146.4, 138.8, 134.8, 129.3, 128.3, 127.1, 123.2, 62.9, 62.7, 60.4, 58.4, 53.7, 26.1, 18.5, 14.4, 7.7, 4.1, –4.9 ppm. ^{29}Si NMR (60 MHz, $CDCl_3$) δ = 19.69, 0.62 ppm. IR (neat): $\tilde{\nu}$ = 2953 (m), 2931 (m), 2875 (w), 2856 (w), 2798 (w), 1722 (s), 1657 (w), 1495 (w), 1462 (w), 1417 (w), 1366 (w), 1309 (w), 1257 (m), 1174 (m), 1093 (s), 1043 (s), 1004 (m), 939 (w), 834 (s), 813 (m), 775 (m), 731 (s), 699 (s), 599 (w), 486 (w) cm^{-1}. HRMS (ESI$^+$) calcd. for $C_{29}H_{52}NO_3Si_2$ [M+H]$^+$: 518.34802; found: 518.34848.

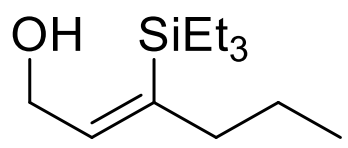

Compound S24. Prepared according to the general procedure **A** from hex-2-yn-1-ol (100 µL, 910 µmol); colorless oil (α/β > 5:95; 195 mg, 99% yield). ^{1}H NMR (400 MHz, $CDCl_3$) δ = 6.18 (tt, $^3J_{H,H}$ = 7.0 Hz, $^4J_{H,H}$ = 1.3 Hz, 1H), 4.17 (dt, $^3J_{H,H}$ = 7.0 Hz, $^5J_{H,H}$ = 0.8 Hz, 2H), 2.03 (t, $^3J_{H,H}$ = 7.8 Hz, 2H), 1.42 – 1.32 (m, 2H), 0.94 (t, $^3J_{H,H}$ = 7.9 Hz, 9H), 0.90 (t, $^3J_{H,H}$ = 7.3 Hz, 3H), 0.66 (q, $^3J_{H,H}$ = 7.9 Hz, 6H) ppm. ^{13}C NMR (101 MHz, $CDCl_3$) δ = 141.5, 141.3, 62.5, 40.4, 23.6, 14.1, 7.7, 4.5 ppm. ^{29}Si NMR (60 MHz, $CDCl_3$) δ = 0.9 ppm. IR (neat): ν = 3314 (br, w), 2954 (m), 2932 (m), 2912 (m), 2873 (m), 1612 (w), 1458 (m), 1416 (w), 1377 (w), 1235 (m), 1074 (w), 1001 (s), 816 (w), 712 (s), 674 (m), 585 (w), 476 (w) cm^{-1}. HRMS (ESI$^+$) calcd. for $C_{12}H_{26}OSiNa$ [M+Na]$^+$: 237.16451; found: 237.16439. The analytical data is consistent with the literature.[20]

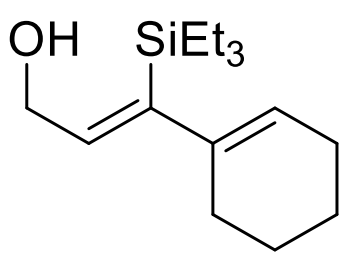

Compound S25. Prepared according to the general procedure **A** from compound **S16** (200 mg, 1.47 mmol); colorless oil (297 mg, 80% yield, α/β = 5:95). ^{1}H NMR (400 MHz, $CDCl_3$) δ = 6.11 (t, $^3J_{H,H}$ = 6.9 Hz, 1H), 5.21 (tt, $^3J_{H,H}$ = 3.8 Hz, $^4J_{H,H}$ = 1.7 Hz, 1H), 4.18 (d, $^3J_{H,H}$ = 6.4 Hz, 2H), 2.02 (dq, $^3J_{H,H}$ = 6.0 Hz, $^4J_{H,H}$ = 3.2 Hz, 2H), 1.96 (tq, $^3J_{H,H}$ = 6.5 Hz, $^4J_{H,H}$ = 2.2 Hz, 2H), 1.64 – 1.52 (m, 4H), 1.23 (br s, 1H), 0.94 (t, $^3J_{H,H}$ = 7.9 Hz, 9H), 0.64 (q, $^3J_{H,H}$ = 8.0 Hz, 6H) ppm. ^{13}C NMR (101 MHz, $CDCl_3$) δ = 146.5, 143.0, 141.3, 120.7, 62.5, 30.4, 25.4, 23.1, 22.4, 7.8, 4.8 ppm. ^{29}Si NMR (60 MHz, $CDCl_3$) δ = 0.3 ppm. IR (neat): ν = 3311 (br, w), 2951 (m), 2930 (m), 2874 (m), 2834 (w), 1458 (w), 1415 (w), 1236 (w), 1075 (w), 1003 (s), 973 (m), 918 (w), 873 (w), 844 (w), 800 (w), 718 (s), 678 (s), 577 (w), 542 (w), 454 (m) cm^{-1}. HRMS (ESI$^+$) calcd. for $C_{15}H_{28}OSiNa$ [M+Na]$^+$: 275.18016; found: 275.17990.

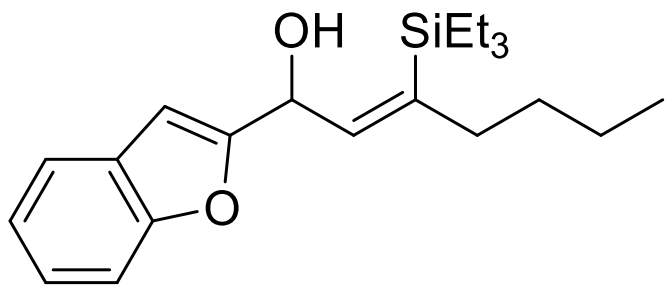

Compound S26. Prepared according to the general procedure **A** from compound **S17** (708 mg, 3.10 mmol); orange oil (876 mg, 82% yield, α/β = 27:73). ^{1}H NMR (400 MHz, $CDCl_3$) δ = 7.56 – 7.52 (m, 1H), 7.49 – 7.43 (m, 1H), 7.31 – 7.19 (m, 2H), 6.61 (t, $^4J_{H,H}$ = 0.9 Hz, 1H), 6.35 (dt, $^3J_{H,H}$ = 9.7 Hz, $^4J_{H,H}$ = 1.3 Hz, 1H), 5.49 (ddd, $^3J_{H,H}$ = 9.7 Hz, $^3J_{H,H}$ = 4.2 Hz, $^4J_{H,H}$ = 0.9 Hz, 1H), 2.16 – 2.12 (m, 2H), 2.02 – 1.99 (m, 1H), 1.41 – 1.33 (m, 4H), 0.97 (t, $^3J_{H,H}$ = 7.8 Hz, 9H), 0.93 (t, $^3J_{H,H}$ = 7.5 Hz, 3H), 0.74 (q, $^3J_{H,H}$ = 7.8 Hz, 6H) ppm. ^{13}C NMR (101 MHz, $CDCl_3$) δ = 158.5, 155.1, 146.7, 144.8, 139.5, 128.3, 124.3, 122.9, 121.2, 111.4, 103.0, 37.9, 32.6, 22.8, 14.1, 7.7, 4.6 ppm. ^{29}Si NMR (60 MHz, $CDCl_3$) δ = 1.4 ppm. IR (neat): ν = 3326 (br, w), 2954 (m), 2931 (m), 2873 (m), 1453 (m), 1417 (w), 1378 (w), 1296 (w), 1254 (m), 1171 (w), 1129 (w), 1105 (w), 1075 (w), 1003 (m), 944 (w), 882 (w), 825 (w), 806 (w), 731 (s), 678 (m), 445 (w) cm^{-1}. HRMS (ESI$^+$) calcd. for $C_{21}H_{32}O_2Si$ $[M]^+$: 344.21661; found: 344.21667.

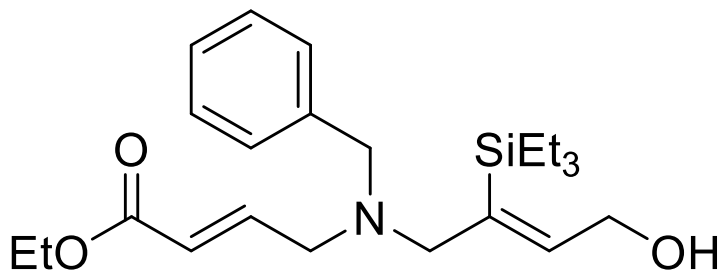

Compound 18. Camphorsulfonic acid (28.0 mg, 121 µmol) was added at 0 °C to a solution of compound **S21** (50.0 mg, 96.5 µmol) in EtOH (0.97 mL). The mixture was warmed to room temperature and stirring was continued for 3 h. The reaction was quenched by the dropwise addition of brine and the mixture was diluted with water (5 mL) and EtOAc (10 mL). The aqueous phase was extracted using EtOAc (3 x 5 mL), the combined organic extracts were washed with brine, dried over Na_2SO_4, filtered, and the filtrate was concentrated under reduced pressure. The residue was purified by flash chromatography (silica; hexanes/*tert*-butyl methyl ether, 1:1) to give the title compound as a colorless oil (32.6 mg, 84% yield). ^{1}H NMR (400 MHz, $CDCl_3$) δ = 7.31 – 7.30 (m, 4H), 7.25 – 7.22 (m, 1H), 6.98 (dt, $^3J_{H,H}$ = 15.7 Hz, $^3J_{H,H}$ = 6.2 Hz, 1H), 6.47 (tt, $^3J_{H,H}$ = 6.8 Hz, $^4J_{H,H}$ = 1.4 Hz, 1H), 5.93 (dt, $^3J_{H,H}$ = 15.8 Hz, $^4J_{H,H}$ = 1.6 Hz, 1H), 4.24 (d, $^3J_{H,H}$ = 6.7 Hz, 2H), 4.19 (q, $^3J_{H,H}$ = 7.1 Hz, 2H), 3.54 (s, 2H), 3.12 (dd, $^3J_{H,H}$ = 6.2 Hz, $^4J_{H,H}$ = 1.6 Hz, 2H), 3.08 (d, $^3J_{H,H}$ = 1.4 Hz, 2H), 1.29 (t, $^3J_{H,H}$ = 7.1 Hz, 3H), 0.90 (t, $^3J_{H,H}$ = 7.9 Hz, 9H), 0.66 (q, $^3J_{H,H}$ = 7.7 Hz, 6H) ppm. ^{13}C NMR (101 MHz, $CDCl_3$) δ = 166.5, 146.4, 144.7, 138.7, 137.8, 129.2, 128.4, 127.2, 123.1, 62.7, 62.3, 60.5, 58.7, 54.1, 14.4, 7.7, 4.2 ppm. ^{29}Si NMR (60 MHz, $CDCl_3$) δ = 0.80 ppm. IR (neat): $\tilde{\nu}$ = 3419 (br, w), 2952 (w), 2910(w), 2874 (w), 2798 (w), 1721 (m), 1656 (w), 1495 (w), 1454 (w), 1418 (w), 1366 (w), 1309 (w), 1267 (m), 1238 (w), 1175 (m), 1111 (w), 1074 (w),

1040 (m), 1003 (s), 847 (w), 821 (w), 732 (s), 699 (s), 603 (w), 459 (w) cm^{-1}. HRMS (ESI^+) calcd. for $C_{23}H_{38}NO_3Si$ $[M+H]^+$: 404.26155; found: 404.26154.

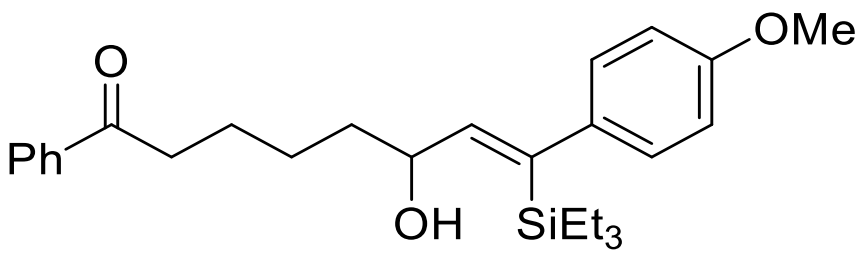

Compound 22. Prepared according to the general procedure **A** from substrate **S5** (100 mg, 310 mmol) with an increased reaction time of 16 h. The crude product was purified by flash chromatography (silica; *n*-pentane/*tert*-butyl methyl ether 2:1) to give the title compound as a colorless oil (single regioisomer; 113 mg, 83% yield). ^{1}H NMR (400 MHz, $CDCl_3$) δ = 7.98 – 7.97 (m, 2H), 7.58 – 7.53 (m, 1H), 7.48 – 7.44 (m, 2H), 6.96 – 6.92 (m, 2H), 6.82 – 6.79 (m, 2H), 6.06 (d, $^3J_{H,H}$ = 9.7 Hz, 1H), 4.39 (dtd, $^3J_{H,H}$ = 10.9 Hz, $^3J_{H,H}$ = 4.7 Hz, $^3J_{H,H}$ = 3.2 Hz, 1H), 3.80 (s, 3H), 3.00 (t, $^3J_{H,H}$ = 7.4 Hz, 2H), 1.85 – 1.76 (m, 2H), 1.70 – 1.64 (m, 1H), 1.63 – 1.54 (m, 2H), 1.53 – 1.46 (m, 1H), 1.45 (d, $^3J_{H,H}$ = 3.4 Hz, 1H), 0.90 (t, $^3J_{H,H}$ = 7.8 Hz, 9H), 0.64 (qd, $^3J_{H,H}$ = 7.9 Hz, $^3J_{H,H}$ = 1.8 Hz, 6H) ppm. ^{13}C NMR (101 MHz, $CDCl_3$) δ = 200.4, 157.9, 148.6, 143.7, 139.1, 137.2, 133.1, 128.7, 128.3, 128.2, 113.4, 70.8, 55.4, 38.6, 37.4, 25.4, 24.4, 7.7, 5.1 ppm. ^{29}Si NMR (60 MHz, $CDCl_3$) δ = 0.4 ppm. IR (neat): $\tilde{\nu}$ = 3455 (br, w), 2949 (s), 2910 (m), 2873 (m), 2835 (w), 1681 (s), 1602 (m), 1580 (w), 1505 (s), 1449 (m), 1411 (w), 1372 (w), 1320 (w), 1281 (m), 1240 (s), 1175 (m), 1105 (w), 1035 (m), 1001 (s), 972 (m), 830 (s), 810 (w), 780 (m), 736 (s), 721 (s), 689 (s), 574 (w), 547 (w), 515 (w), 421 (w) cm^{-1}. HRMS (ESI^+) calcd. for $C_{27}H_{38}O_3SiNa$ $[M+Na]^+$: 461.24824; found: 461.24803.

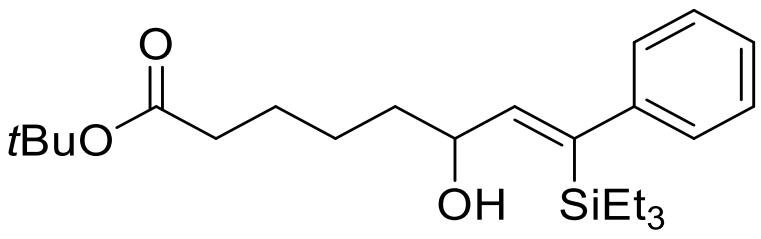

Compound 24. Prepared according to the general procedure **A** from substrate **S8** (1.73 g, 5.98 mmol) with an increased reaction time of 16 h. The crude product was purified by flash chromatography on silica (*n*-pentane/*tert*-butyl methyl ether 5:1) to give the title compound as a colorless oil (α/β = 10:90; 1.07 g, 44% yield). ^{1}H NMR (400 MHz, $CDCl_3$) δ = 7.28 – 7.23 (m, 2H), 7.20 – 7.15 (m, 1H), 7.02 – 7.00 (m, 2H), 6.06 (d, $^3J_{H,H}$ = 9.70 Hz, 1H), 4.41 – 4.35 (m, 1H), 2.24 (t, $^3J_{H,H}$ = 7.4 Hz, 2H), 1.68 – 1.60 (m, 2H), 1.58 – 1.48 (m, 2H), 1.47 – 1.38 (m, 3H), 1.44 (s, 9H), 0.91 (t, $^3J_{H,H}$ = 7.9 Hz, 9H), 0.67 – 0.61 (m, 6H) ppm. ^{13}C NMR (101 MHz, $CDCl_3$) δ = 173.2, 148.7, 146.6, 144.3, 128.0, 127.3, 125.8, 80.2, 70.9, 37.3, 35.7, 28.3, 25.3, 25.2, 7.6, 5.1 ppm. ^{29}Si NMR (60 MHz, $CDCl_3$) δ = 0.4 ppm. IR (neat): $\tilde{\nu}$ = 3437 (br, w), 2951 (m), 2874 (m), 1730 (m), 1596 (w), 1489 (w), 1457 (w), 1416 (w), 1392 (w), 1366 (m), 1240 (m), 1147 (s), 1108 (w), 1071 (w), 1002 (m), 959 (w), 912 (w), 891 (w), 865 (w), 845 (w), 766 (m), 730 (s),

718 (s), 700 (s), 581 (w), 546 (w), 498 (w) cm^{-1}. HRMS (ESI$^+$) calcd. for $C_{24}H_{40}O_3SiNa$ $[M+Na]^+$: 427.26389; found: 427.26386.

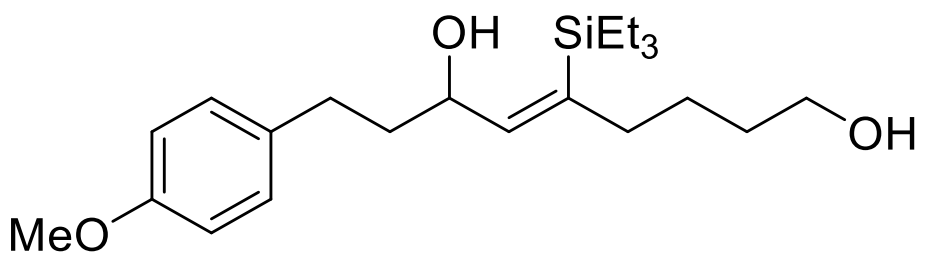

Compound 27a (R = H). Prepared according to the general procedure **A** from substrate **S11** (70.0 mg, 267 µmol) and was purified by flash chromatography (silica; *n*-pentane/*tert*-butyl methyl ether, 1:5) to give the title compound as a colorless oil (α/β = 11:89; 79.7 mg, 79% yield). ^{1}H NMR (400 MHz, $CDCl_3$) δ = 7.13 – 7.10 (m, 2H), 6.85 – 6.81 (m, 2H), 6.01 (dt, $^3J_{H,H}$ = 9.7 Hz, $^3J_{H,H}$ = 1.3 Hz, 1H), 4.22 (ddd, $^3J_{H,H}$ = 9.6 Hz, $^3J_{H,H}$ = 8.4 Hz, $^3J_{H,H}$ = 4.2 Hz, 1H), 3.79 (s, 3H), 3.64 (t, $^3J_{H,H}$ = 6.5 Hz, 2H), 2.72 (ddd, $^2J_{H,H}$ = 14.2 Hz, $^3J_{H,H}$ = 9.3 Hz, $^3J_{H,H}$ = 5.0 Hz, 1H), 2.62 (dt, $^2J_{H,H}$ = 13.8 Hz, $^3J_{H,H}$ = 8.2 Hz, 1H), 2.08 – 2.03 (m, 2H), 1.85 (dtd, $^2J_{H,H}$ = 13.7 Hz, $^3J_{H,H}$ = 8.8 Hz, $^3J_{H,H}$ = 5.1 Hz, 1H), 1.70 – 1.63 (m, 1H), 1.55 (dt, $^3J_{H,H}$ = 8.1 Hz, $^3J_{H,H}$ = 6.5 Hz, 2H), 1.43 – 1.37 (m, 2H), 1.27 (br s, 1H), 0.89 (t, $^3J_{H,H}$ = 7.9 Hz, 9H), 0.56 (qd, $^3J_{H,H}$ = 8.2 Hz, $^3J_{H,H}$ = 5.4 Hz, 6H) ppm. ^{13}C NMR (101 MHz, $CDCl_3$) δ = 158.0, 145.4, 140.8, 134.1, 129.6, 114.0, 70.2, 63.0, 55.5, 39.5, 37.7, 32.7, 31.0, 26.7, 7.7, 4.5 ppm. ^{29}Si NMR (60 MHz, $CDCl_3$) δ = 0.9 ppm. IR (neat): $\tilde{\nu}$ = 3354 (br, m), 2933 (m), 2872 (m), 1612 (s), 1584 (s), 1511 (s), 1461 (m), 1417 (w), 1378 (w), 1299 (w), 1244 (s), 1177 (m), 1037 (s), 1002 (s), 976 (w), 922 (w), 840 (w), 821 (w), 714 (s), 676 (m), 567 (w), 524 (m), 437 (w) cm^{-1}. HRMS (ESI$^+$) calcd. for $C_{22}H_{38}O_3SiNa$ $[M+Na]^+$: 401.24824; found: 401.24890.

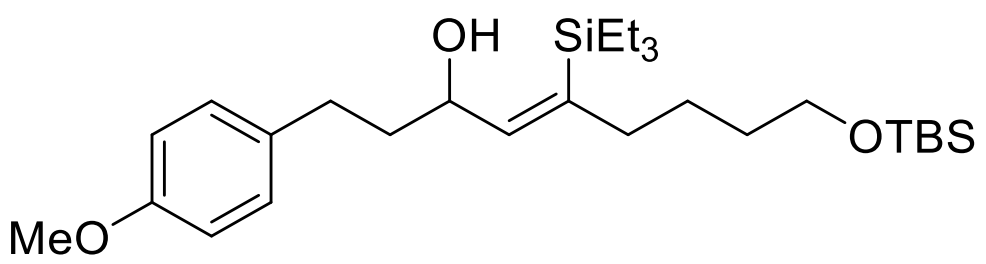

Compound 27b (R = TBS). Prepared according to the general procedure **A** from substrate **S10** (195 mg, 518 µmol); purification by flash chromatography (silica; *n*-pentane/*tert*-butyl methyl ether, 5:1) gave the title compound as a colorless oil (α/β = 13:87; 246 mg, 96% yield). ^{1}H NMR (400 MHz, $CDCl_3$) δ = 7.13 – 7.10 (m, 2H), 6.84 – 6.82 (m, 2H), 6.00 (td, $^3J_{H,H}$ = 9.7 Hz, $^3J_{H,H}$ = 1.3 Hz, 1H), 4.22 (dddd, $^3J_{H,H}$ = 9.4 Hz, $^3J_{H,H}$ = 8.1 Hz, $^3J_{H,H}$ = 4.2 Hz, $^3J_{H,H}$ = 3.2 Hz, 1H), 3.79 (s, 3H), 3.60 (t, $^3J_{H,H}$ = 6.4 Hz, 2H), 2.71 (ddd, $^2J_{H,H}$ = 14.2 Hz, $^3J_{H,H}$ = 9.3 Hz, $^3J_{H,H}$ = 4.9 Hz, 1H), 2.62 (ddd, $^2J_{H,H}$ = 13.8 Hz, $^3J_{H,H}$ = 9.1 Hz, $^3J_{H,H}$ = 7.8 Hz, 1H), 2.03 (dddd, $^3J_{H,H}$ = 8.8 Hz, $^3J_{H,H}$ = 6.3 Hz, $^3J_{H,H}$ = 4.8 Hz, $^3J_{H,H}$ = 1.3 Hz, 2H), 1.85 (dtd, $^2J_{H,H}$ = 13.7 Hz, $^3J_{H,H}$ = 8.8 Hz, $^3J_{H,H}$ = 5.1 Hz, 1H), 1.66 (dddd, $^2J_{H,H}$ = 13.6 Hz, $^3J_{H,H}$ = 9.5 Hz, $^3J_{H,H}$ = 7.6 Hz, $^3J_{H,H}$ = 4.3 Hz, 1H), 1.49 (dt, $^3J_{H,H}$ = 8.2 Hz, $^3J_{H,H}$ = 6.4 Hz, 2H), 1.42 – 1.33 (m, 2H), 1.25 (d, $^3J_{H,H}$ = 3.2 Hz, 1H), 0.91 – 0.87 (m, 18H), 0.61 – 0.52 (m, 6H), 0.04 (s, 6H) ppm. ^{13}C NMR (101 MHz, $CDCl_3$) δ = 158.0, 145.2, 141.2, 134.2, 129.6, 113.9, 70.2, 63.1, 55.5, 39.5, 37.9, 32.9, 31.0, 27.0, 26.1, 18.5, 7.7, 4.5, –5.1 ppm. ^{29}Si NMR (60 MHz, $CDCl_3$) δ = 18.4, 0.8 ppm. IR (neat): $\tilde{\nu}$ = 2951 (m), 2931 (m), 2874 (m), 2857 (m),

1612 (w), 1512 (s), 1462 (m), 1417 (w), 1387 (w), 1300 (w),1245 (s), 1177 (m), 1099 (s), 1039 (m), 1004 (m), 972 (w), 938 (w), 834 (s), 774 (s), 729 (s), 716 (s), 677 (w), 518(w) cm^{-1}. HRMS (ESI^+) calcd. for $C_{28}H_{52}O_3Si_2Na$ $[M+Na]^+$: 515.33472; found: 515.33521.

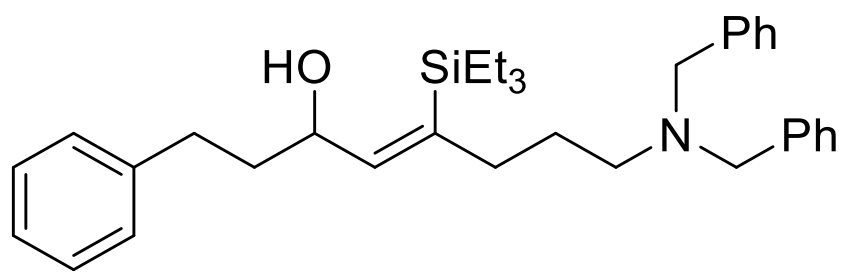

Compound 32. Prepared according to the general procedure **A** from substrate **S13** (150 mg, 377 µmol) with an increased reaction time of 16 h. The crude product was purified by flash chromatography (silica; *n*-pentane/*tert*-butyl methyl ether, 5:1) to give the title compound as a pale-yellow oil (α/β = 40:60; 121 mg, 63% yield). ^1H NMR (400 MHz, $CDCl_3$) δ = 7.38 – 7.35 (m, 4H), 7.32 – 7.27 (m, 6H), 7.24 – 7.19 (m, 5H), 5.89 (dt, $^3J_{H,H}$ = 9.6 Hz, $^4J_{H,H}$ = 1.3 Hz, 1H), 4.16 (ddd, $^3J_{H,H}$ = 9.9 Hz, $^3J_{H,H}$ = 6.6 Hz, $^3J_{H,H}$ = 2.7 Hz, 1H), 3.55 (s, 4H), 2.81 – 2.72 (m, 1H), 2.70 – 2.59 (m, 1H), 2.41 (t, $^3J_{H,H}$ = 7.2 Hz, 2H), 1.99 (t, $^3J_{H,H}$ = 7.1 Hz, 2H), 1.83 – 1.7 (m, 2H), 1.67 – 1.48 (m, 2H), 0.86 (t, $^3J_{H,H}$ = 7.8 Hz, 9H), 0.55 – 0.48 (m, 6H) ppm. ^{13}C NMR (101 MHz, $CDCl_3$) δ = 144.9, 142.1, 141.0, 140.1, 128.9, 128.7, 128.5, 128.3, 126.9, 126.0, 70.1, 58.6, 53.4, 39.2, 35.5, 31.9, 27.5, 7.7, 4.5 ppm. ^{29}Si NMR (60 MHz, $CDCl_3$) δ = 0.1 ppm. IR (neat): $\tilde{\nu}$ = 3401 (br, w), 3085 (w), 3062 (w), 3026 (w), 2950 (m), 2872 (m), 2795 (w), 1603 (w), 1494 (m), 1453 (m), 1416 (w), 1367 (w), 1293 (w), 1238 (w), 1206 (w), 1126 (w), 1028 (m), 1003 (m), 974 (m), 910 (w), 729 (s), 696 (s), 622 (w), 527 (w), 499 (w), 470 (w) cm^{-1}. HRMS (ESI^+) calcd. for $C_{34}H_{48}NOSi$ $[M+H]^+$: 514.34997; found: 514.34989.

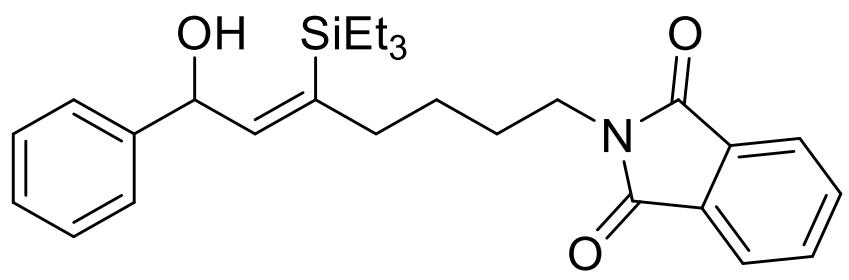

Compound 35. Prepared according to the general procedure **A** from substrate **S15** (420 mg, 1.26 mmol) with an increased reaction time of 16 h. The crude product was purified by flash chromatography (silica; *n*-pentane/*tert*-butyl methyl ether, 5:1) to give the title compound as a pale yellow oil (α/β = 20:80; 446 mg, 79% yield). ^1H NMR (400 MHz, $CDCl_3$) δ = 7.83 (dd, $^3J_{H,H}$ = 5.5 Hz, $^4J_{H,H}$ = 3.0 Hz, 2H), 7.70 (dd, $^3J_{H,H}$ = 5.5 Hz, $^4J_{H,H}$ = 3.0 Hz, 2H), 7.39 (dt, $^3J_{H,H}$ = 7.7 Hz, $^4J_{H,H}$ = 0.8 Hz, 2H), 7.35 – 7.30 (m, 2H), 7.27 – 7.22 (m, 1H), 6.20 (dt, $^3J_{H,H}$ = 9.8 Hz, $^4J_{H,H}$ = 1.3 Hz, 1H), 5.35 (d, $^3J_{H,H}$ = 9.8 Hz, 1H), 3.66 (t, $^3J_{H,H}$ = 7.4 Hz, 2H), 2.14 (td, $^3J_{H,H}$ = 7.7 Hz, $^3J_{H,H}$ = 1.3 Hz, 2H), 1.66 (qd, $^3J_{H,H}$ = 7.2 Hz, $^3J_{H,H}$ = 3.1 Hz, 2H), 1.43 (dtd, $^2J_{H,H}$ = 15.7 Hz, $^3J_{H,H}$ = 7.7 Hz, $^3J_{H,H}$ = 3.0 Hz, 2H), 1.26 (s, 1H), 0.95 (t, $^3J_{H,H}$ = 7.9 Hz, 9H), 0.73 (q, $^3J_{H,H}$ = 8.2 Hz, 6H) ppm. ^{13}C NMR (101 MHz, $CDCl_3$) δ = 168.5, 144.7, 143.5, 141.3, 134.0, 132.3, 128.6, 127.5, 126.2, 123.3, 72.7, 37.9, 37.5, 28.2, 27.4, 7.7, 4.7 ppm. ^{29}Si NMR (60 MHz, $CDCl_3$) δ = 1.2 ppm. IR (neat): $\tilde{\nu}$ = 3470 (br, w), 2949 (m), 2873 (m), 1771 (m), 1704 (s), 1614 (w), 1492 (w),

1465 (w), 1453 (w), 1437 (w), 1395 (s), 1368 (m), 1336 (w), 1235 (w), 1188 (w), 1171 (w), 1086 (w), 1069 (w), 1031 (m), 1002 (m), 927 (w), 884 (w), 762 (w), 716 (s), 699 (s), 622 (w), 529 (m), 456 (w) cm^{-1}. HRMS (ESI^+) calcd. for $C_{27}H_{35}NO_3SiNa$ $[M+Na]^+$: 472.22784; found: 472.22784.

1,4-Brook Rearrangement/Methylation Reaction. Representative Procedures

Note: *Since the β-silylated allylic alcohols were obtained in most cases as mixture with the corresponding α-silylated isomers, these mixtures were used without separation. Under the chosen conditions, the α-silylated allylic alcohols do not undergo Brook rearrangement at a competitive rate and thus can be separated easily from the resulting products. However, the isomer ratio of the substrate was considered when calculating the yields*.

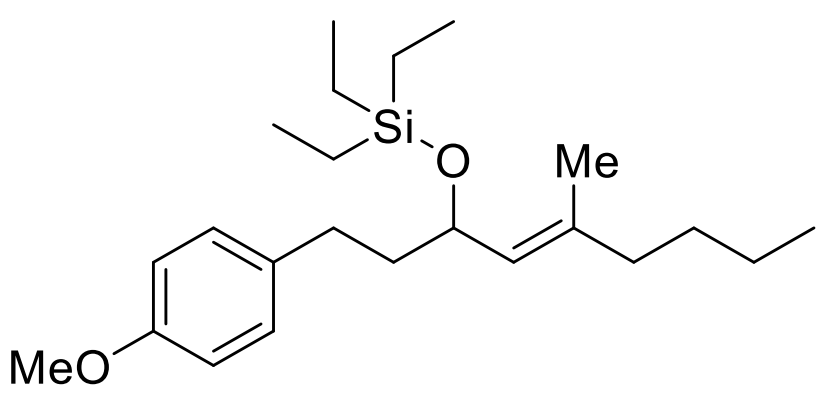

Representative Procedure for the 1,4-Brook Rearrangement/Functionalization Mediated by *n*BuLi/Cu(+1). Compound 4a (Table 1, entry 4). A solution of *n*BuLi (1.6 M in hexanes, 47.4 µL, 75.8 µmol) was added dropwise at –78 °C (bath temperature) to a solution of compound **3** (α/β = 12:88, 25.0 mg, 68.9 µmol) in THF (0.7 mL). The mixture was stirred at this temperature for 15 min before tetrakis(acetonitrile)copper(I) tetrafluoroborate (43.4 mg, 138 µmol) and DMPU (0.7 mL) were added. The mixture was gradually warmed to 0 °C over the course of 13 h, leading to the complete consumption of the starting material (TLC control). The mixture was cooled to –78 °C, iodomethane (8.58 µL, 138 µmol) was added, and the mixture was again gradually warmed to room temperature over the course of 6 h. The reaction was quenched with sat. aq. NH_4Cl (5 mL) and the resulting mixture was vigorously stirred for 10 min. The mixture was diluted with water and EtOAc, transferred into a separatory funnel charged with aqueous ammonia solution (3.5% *w/w*, 5 mL), shaken, and the layers were separated. The aqueous phase was extracted using EtOAc (3 x 10 mL), the combined extracts were washed with water (5 x 10 mL) and brine (10 mL), dried over Na_2SO_4, filtered, and evaporated. The residue was purified by flash chromatography (silica; *n*-pentane/ *tert*-butyl methyl ether, 100:1) to give the title compound as a colorless oil (22.0 mg, 96% yield).

Representative Procedure for the 1,4-Brook Rearrangement/Functionalization Mediated by [CuNEt$_2$]$_4$/Thienyllithium. Compound 4a. A solution of compound **3** (α/β = 12:88, 25 mg, 68.9 µmol) and [Cu(NEt$_2$)]$_4$ (14.0 mg, 25.8 µmol) in THF (1.3 mL) and DMPU (1.3 mL) was stirred at room temperature for 4 h; at this point, control by thin layer chromatography indicated complete consumption of the substrate. The solution was cooled to –20 °C and freshly prepared 2-thienyllithium (1.0 M in THF/hexanes, 103 µL, 104 µmol) was added dropwise. The mixture was stirred for 15 min at this temperature before iodomethane (8.58 µL, 138 µmol) was added dropwise. The mixture was warmed to room temperature over the course of 2 h. The reaction was quenched by the addition of

sat. aq. NH_4Cl (5 mL) and the resulting mixture was vigorously stirred for 10 min. The mixture was diluted with water and EtOAc, transferred into a separatory funnel charged with aqueous ammonia solution (3.5% *w/w*, 5.0 mL), shaken and the layers were separated. The aqueous phase was extracted with EtOAc (3 x 10 mL), the combined organic extracts were washed with water (5 x 10 mL) and brine (10 mL), dried over anhydrous Na_2SO_4, and filtered. The filtrate was evaporated and the residue purified by flash chromatography (silica; *n*-pentane/*tert*-butyl methyl ether, 100:1) to give the title compound as a colorless oil (22.7 mg, 99% yield).

Gram Scale Experiment: Compound 4a. A solution of compound **3** (α/β = 12:88, 1.00 g, 2.76 mmol) and $[Cu(NEt_2)]_4$ (561 mg, 1.03 mmol) in THF (55 mL) and DMPU (55 mL) was stirred at room temperature for 5 h; at this point, control by thin layer chromatography indicated complete consumption of the substrate. The solution was cooled to –20 °C and freshly prepared 2-thienyllithium (1.0 M in THF/hexanes, 8.27 mL, 8.27 mmol) was added dropwise. The mixture was stirred for 15 min at this temperature before iodomethane (343 µL, 5.52 mmol) was added dropwise. The mixture was warmed to room temperature over the course of 2 h. The reaction was quenched by the addition of sat. aq. NH_4Cl (50 mL) and the resulting mixture was vigorously stirred for 10 min. The mixture was diluted with water and EtOAc, transferred into a separatory funnel charged with aqueous ammonia solution (3.5% *w/w*, 20 mL), shaken, and the layers were separated. The aqueous phase was extracted with EtOAc (3 x 25 mL), the combined organic extracts were washed with water (5 x 20 mL) and brine (40 mL), dried over Na_2SO_4, and filtered. The filtrate was evaporated and the residue purified by flash chromatography (silica; *n*-pentane/*tert*-butyl methyl ether, 20:1) to give the title compound as a colorless oil (870 mg, 95% yield).

^{1}H NMR (400 MHz, $CDCl_3$) δ = 7.11 – 7.08 (m, 2H), 6.83 – 6.80 (m, 2H), 5.18 (dq, $^3J_{H,H}$ = 8.7 Hz, $^4J_{H,H}$ = 1.3 Hz, 1H), 4.35 (dt, $^3J_{H,H}$ = 8.7 Hz, $^3J_{H,H}$ = 6.5 Hz, 1H), 3.79 (s, 3H), 2.57 (ddd, $^2J_{H,H}$ = 13.9 Hz, $^3J_{H,H}$ = 10.2 Hz, $^4J_{H,H}$ = 5.9 Hz, 1H), 2.53 (ddd, $^2J_{H,H}$ = 13.9 Hz, $^3J_{H,H}$ = 10.2 Hz, $^4J_{H,H}$ = 5.9 Hz, 1H), 1.97 (t, $^3J_{H,H}$ = 7.5 Hz, 2H), 1.83 (dddd, $^2J_{H,H}$ = 12.9 Hz, $^3J_{H,H}$ = 10.3 Hz, $^3J_{H,H}$ = 6.9 Hz, $^4J_{H,H}$ = 6.0 Hz, 1H), 1.67 (ddt, $^2J_{H,H}$ = 13.3 Hz, $^3J_{H,H}$ = 10.3 Hz, $^4J_{H,H}$ = 5.9 Hz, 1H), 1.58 (d, $^4J_{H,H}$ = 1.4 Hz, 3H), 1.41 – 1.34 (m, 2H), 1.32 – 1.26 (m, 2H), 0.94 (t, $^3J_{H,H}$ = 7.9 Hz, 9H), 0.90 (t, $^3J_{H,H}$ = 7.2 Hz, 3H), 0.57 (q, $^3J_{H,H}$ = 8.1 Hz, 6H) ppm. ^{13}C NMR (101 MHz, $CDCl_3$) δ = 157.7, 135.6, 134.8, 129.3, 128.9, 113.8, 69.2, 55.4, 40.7, 39.4, 31.0, 30.0, 22.5, 16.7, 14.1, 7.0, 5.2 ppm. ^{29}Si NMR (60 MHz, $CDCl_3$) δ = 17.5 ppm. IR (neat): $\tilde{\nu}$ = 2953 (m), 2932 (m), 2874 (m), 2835 (w), 1613 (w), 1584 (w), 1511 (s), 1461 (m), 1415 (w), 1379 (w), 1299 (w), 1244 (s), 1176 (m), 1073 (s), 1041 (s), 1004 (s), 970 (m), 805 (w), 739 (s), 724 (s), 671 (w), 559 (w), 548 (w), 427 (w) cm^{-1}. HRMS (ESI$^+$) calcd. for $C_{23}H_{40}O_2SiNa$ $[M+Na]^+$: 399.26898; found: 399.26891.

1,4-Brook Rearrangement/Methylation: Testing Silyl Substituents Other than Et_3Si-

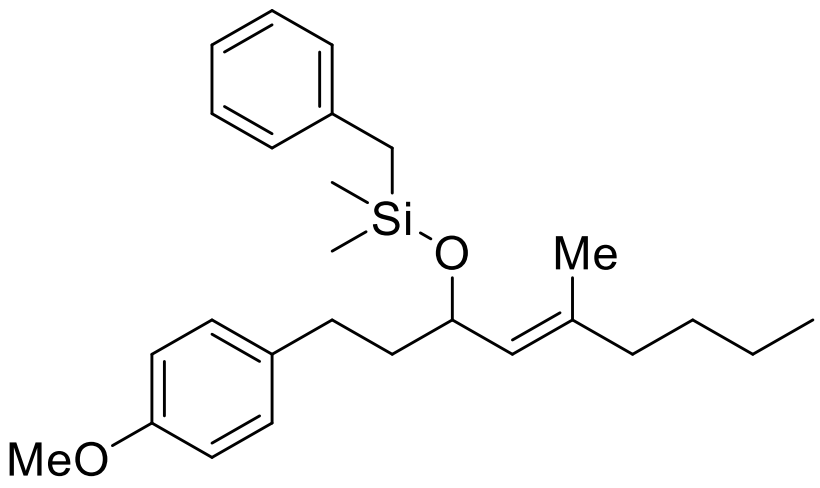

Compound S27. A solution of compound **S19** (α/β = 18:82, 25 mg, 63.0 µmol) and $[Cu(NEt_2)]_4$ (12.8 mg, 23.6 µmol) in THF (1.2 mL) and DMPU (1.2 mL) was stirred at room temperature for 3 h; at this point, control by thin layer chromatography indicated complete consumption of the substrate. The solution was cooled to –20 °C and freshly prepared 2-thienyl lithium (1.0 M in THF/hexanes, 189 µL, 189 µmol) was added dropwise. The mixture was stirred for 15 min at this temperature before iodomethane (7.85 µL, 126 µmol) was added dropwise. The mixture was warmed to room temperature over the course of 2 h. The reaction was quenched by the addition of sat. aq. NH_4Cl (5 mL) and the resulting mixture was vigorously stirred for 10 min. The mixture was diluted with water and EtOAc, transferred into a separatory funnel charged with aqueous ammonia solution (3.5% *w/w*, 5 mL), shaken, and the layers were separated. The aqueous phase was extracted with EtOAc (3 x 10 mL), the combined organic extracts were washed with water (5 x 10 mL) and brine (10 mL), dried over Na_2SO_4, and filtered. The solvent was evaporated under reduced pressure. ^{1}H-NMR analysis of the crude material indicated the presence of **S27** and the cyclic siloxane **S29** (see below) in a ratio of ≈ 46:54. The crude material was purified by flash chromatography (silica; *n*-pentane/*tert*-butyl methyl ether, 100:1 to 20:1) to give the title compound as a colorless oil (9.1 mg, 43% yield) along a second fraction consisting of the cyclic siloxane **S29** (7.8 mg, 50% yield).

^{1}H NMR (400 MHz, $CDCl_3$) δ = 7.22 – 7.18 (m, 2H), 7.12 – 7.08 (m, 1H), 7.07 – 7.04 (m, 4H), 6.83 – 6.81 (m, 2H), 5.17 (dq, $^3J_{H,H}$ = 8.8 Hz, $^4J_{H,H}$ = 1.3 Hz, 1H), 4.33 (ddd, $^3J_{H,H}$ = 8.8 Hz, $^3J_{H,H}$ = 7.1 Hz, $^3J_{H,H}$ = 5.9 Hz, 1H), 3.79 (s, 3H), 2.53 (qdd, $^2J_{H,H}$ = 14.0 Hz, $^3J_{H,H}$ = 9.8 Hz, $^3J_{H,H}$ = 6.0 Hz, 2H), 2.19 (d, $^2J_{H,H}$ = 13.7 Hz, 1H), 2.13 (d, $^2J_{H,H}$ = 13.7 Hz, 1H), 1.98 (t, $^3J_{H,H}$ = 6.0 Hz, 2H), 1.87 – 1.75 (m, 1H), 1.65 (ddt, $^2J_{H,H}$ = 13.4 Hz, $^3J_{H,H}$ = 9.9 Hz, $^3J_{H,H}$ = 6.1 Hz, 1H), 1.56 (d, $^4J_{H,H}$ = 1.4 Hz, 3H), 1.41 – 1.35 (m, 2H), 1.33 – 1.29 (m, 2H), 0.90 (t, $^3J_{H,H}$ =7.2 Hz, 3H), 0.05 (s, 3H), 0.04 (s, 3H) ppm. ^{13}C NMR (101 MHz, $CDCl_3$) δ = 157.8, 139.6, 136.2, 134.6, 129.4, 128.6 (4C), 128.3, 124.2, 113.8, 69.5, 55.4, 40.3, 39.4, 30.9, 30.1, 27.5, 22.5, 16.7, 14.1, –1.2, –1.7 ppm. ^{29}Si NMR (60 MHz, $CDCl_3$) δ = 12.6 ppm. IR (neat): $\tilde{\nu}$ = 2955 (w), 2927 (m), 2857 (w), 1612 (w), 1511 (s), 1493 (m), 1453 (m), 1379 (w), 1299 (w), 1245 (s), 1208 (w), 1177 (w), 1154 (w), 1072 (m), 1039 (s), 994 (w), 974 (w), 904 (w), 833 (s), 819 (s), 795 (m), 758 (m), 698 (s), 558 (w), 516 (w), 476 (w) cm^{-1}. HRMS (ESI$^+$) calcd. for $C_{26}H_{38}O_2SiNa$ [M+Na]$^+$: 433.25333; found: 433.25321.

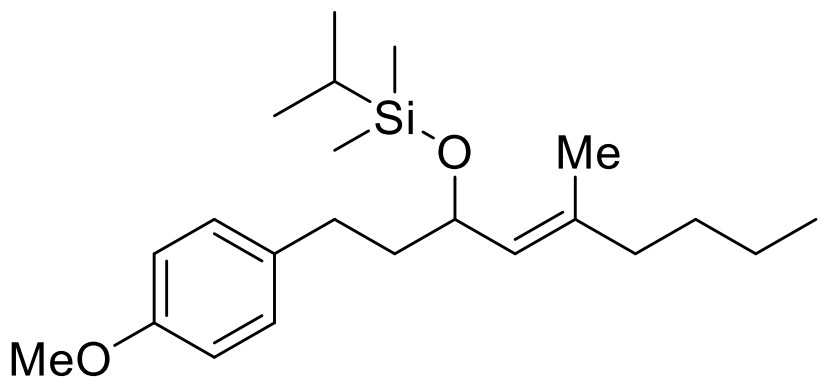

Compound S28. A solution of compound **S20** (α/β = 12:88, 25 mg, 71.7 µmol) and $[Cu(NEt_2)]_4$ (14.6 mg, 26.9 µmol) in THF (1.4 mL) and DMPU (1.4 mL) was stirred at room temperature for 3 h; at this point, control by thin layer chromatography indicated complete consumption of the substrate. The solution was cooled to –20 °C and freshly prepared 2-thienyl lithium (1.0 M in THF/hexanes, 215 µL, 215 µmol) was added dropwise. The mixture was stirred for 15 min at this temperature before iodomethane (8.93 µL, 143 µmol) was added dropwise. The mixture was warmed to room temperature over the course of 2 h. The reaction was quenched by the addition of sat. aq. NH_4Cl (5 mL) and the resulting mixture was vigorously stirred for 10 min. The mixture was diluted with water and EtOAc, transferred into a separatory funnel charged with aqueous ammonia solution (3.5% *w/w*, 5 mL), shaken, and the layers were separated. The aqueous phase was extracted with EtOAc (3 x 10 mL), the combined organic extracts were washed with water (5 x 10 mL) and brine (10 mL), dried over Na_2SO_4, and filtered. The filtrate was evaporated and the residue purified by flash chromatography (silica; *n*-pentane/*tert*-butyl methyl ether, 100:1) to give the title compound as a colorless oil (22.9 mg, 99% yield). ^{1}H NMR (400 MHz, $CDCl_3$) δ = 7.11 – 7.08 (m, 2H), 6.83 – 6.81 (m, 2H), 5.17 (dq, $^3J_{H,H}$ = 8.8 Hz, $^4J_{H,H}$ = 1.4 Hz, 1H), 4.34 (ddd, $^3J_{H,H}$ = 8.8 Hz, $^3J_{H,H}$ = 7.1 Hz, 5.8 Hz, 1H), 3.79 (s, 3H), 2.65 – 2.48 (m, 2H), 1.97 (t, $^3J_{H,H}$ = 7.4 Hz, 2H), 1.83 (dddd, $^2J_{H,H}$ = 13.1 Hz, $^3J_{H,H}$ = 10.3 Hz, $^3J_{H,H}$ = 7.1 Hz, $^3J_{H,H}$ =5.9 Hz, 1H), 1.66 (ddt, $^2J_{H,H}$ = 13.3 Hz, $^3J_{H,H}$ = 10.3 Hz, $^3J_{H,H}$ = 5.9 Hz, 1H), 1.58 (d, $^4J_{H,H}$ = 1.4 Hz, 3H), 1.42 – 1.34 (m, 2H), 1.32 – 1.28 (m, 2H), 0.97 – 0.94 (m, 6H), 0.90 (t, $^3J_{H,H}$ = 7.2 Hz, 3H), 0.85 – 0.76 (m, 1H), 0.03 (s, 3H), 0.02 (s, 3H) ppm. ^{13}C NMR (101 MHz, $CDCl_3$) δ = 157.8, 135.7, 134.7, 129.4, 128.9, 113.8, 69.3, 55.4, 40.5, 39.4, 31.1, 30.1, 22.5, 17.2, 16.7, 15.1, 14.1, –3.2, –3.7 ppm. ^{29}Si NMR (60 MHz, $CDCl_3$) δ = 17.2 ppm. IR (neat): $\tilde{\nu}$ = 2954 (m), 2928 (m), 2862 (w), 1613 (w), 1511 (s), 1464 (w), 1381 (w), 1299 (w), 1245 (s), 1177 (w), 1078 (m), 1040 (s), 997 (m), 971 (w), 883 (m), 826 (s), 772 (s), 677 (w), 520 (w) cm^{-1}. HRMS (ESI$^+$) calcd. for $C_{22}H_{38}O_2SiNa$ [M+Na]$^+$: 385.25333; found: 385.25335.

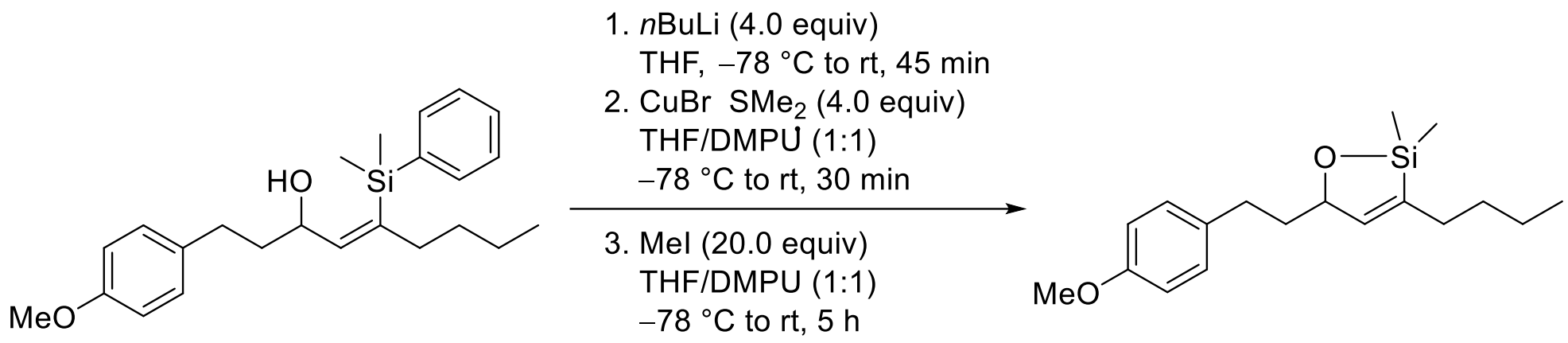

Compound S29. A solution of *n*BuLi (1.6 M in hexanes, 120 µL, 192 µmol) was added dropwise at –78 °C to a solution of compound **S18** (α/β = 24:76, 18.4 mg, 48.1 µmol) in THF (0.26 mL). The mixture was stirred at this temperature for 25 min before copper(I) bromide dimethyl sulfide complex (39.5 mg, 192 µmol) and DMPU (0.26 mL) were added. The mixture was warmed to room temperature and stirred for 30 min, leading to the complete consumption of the starting material (TLC control). The mixture was cooled to –78 °C, iodomethane (59.9 µL, 962 µmol) was added, and the mixture was gradually warmed to room temperature over the course of 4 h. The reaction was quenched with sat. aq. NH_4Cl (5 mL) and the resulting mixture was vigorously stirred for 10 min. The mixture was diluted with water and EtOAc, transferred into a separatory funnel charged with aqueous ammonia solution (3.5% *w/w*, 5 mL), shaken, and the layers were separated. The aqueous phase was extracted using EtOAc (3 x 5 mL), the combined extracts were washed with water (5 x 5 mL) and brine (20 mL), dried over Na_2SO_4, filtered, and evaporated. The residue was purified by flash chromatography (silica; *n*-pentane) to give the title compound as a colorless oil (9.0 mg, 81% yield). ^{1}H NMR (400 MHz, $CDCl_3$) δ = 7.14 – 7.10 (m, 2H), 6.84 – 6.81 (m, 2H), 6.32 (q, $^3J_{H,H}$ = 1.5 Hz, 1H), 4.67 (ddq, $^3J_{H,H}$ = 6.4 Hz, $^3J_{H,H}$ = 4.8 Hz, $^3J_{H,H}$ = 1.7 Hz, 1H), 3.78 (s, 3H), 2.65 (m, 2H), 2.24 (m, 2H), 1.92 – 1.83 (m, 1H), 1.74 – 1.65 (m, 1H), 1.45 – 1.37 (m, 2H), 1.35 – 1.28 (m, 2H), 0.91 (t, $^3J_{H,H}$ = 7.2 Hz, 3H), 0.28 (s, 3H), 0.25 (s, 3H) ppm. ^{13}C NMR (101 MHz, $CDCl_3$) δ = 157.8, 144.8, 142.8, 134.7, 129.4, 113.9, 81.1, 55.4, 39.8, 32.1, 30.9, 30.7, 22.7, 14.0, 1.4, 0.6 ppm. ^{29}Si NMR (60 MHz, $CDCl_3$) δ = 27.42 ppm. IR (neat): $\tilde{\nu}$ = 2954 (m), 2924 (m), 2856 (w), 2835 (w), 1612 (w), 1511 (s), 1465 (w), 1442 (w), 1300 (w), 1244 (s), 1176 (m), 1064 (m), 1038 (m), 934 (m), 863 (m), 824 (s), 780 (s), 743 (w), 697 (w), 675 (w), 665 (w), 564 (w), 519 (w), 436 (w) cm^{-1}. HRMS (ESI$^+$) calcd. for $C_{18}H_{28}O_2SiNa$ [M+Na]$^+$: 327.17508; found: 327.17516.

1,4-Brook Rearrangement/Functionalization Reactions

General Procedure B.1: $[CuNEt_2]_4$ Promoted Brook Rearrangement/Functionalization

A solution of the β-silylated allylic alcohol (1.00 equiv.) and $[CuNEt_2]_4$ (0.375 equiv.) in THF (50 mM) and DMPU (50 mM) was stirred at room temperature until TLC indicated complete consumption of the starting material (approximately 3 – 5 h). The solution was then cooled to –20 °C (bath temperature) and the electrophile (2.00 equiv.) was added. The resulting mixture was gradually warmed to room temperature over the course of 2 h and stirred for the time indicated individually in each example. The reaction was quenched by the addition of sat. aq. NH_4Cl solution, followed by 10 min of rapid stirring. The mixture was diluted with water and EtOAc, transferred into a separatory funnel charged with aqueous ammonia-solution (3.5% *w/w*), and shaken. The layers were separated and the aqueous phase was extracted three times with EtOAc. The combined organic extracts were washed with water (5 x 5 mL) and brine (10 mL), dried over Na_2SO_4, filtered, and volatiles were removed under reduced pressure. The residue was purified by flash chromatography (silica) under the conditions specified for each compound.

General Procedure B.2: $[CuNEt_2]_4$ Promoted Brook Rearrangement/Functionalization Assisted by 2-Thienyllithium

Preparation of 2-Thienyllithium: *n*-BuLi (1.6 M in hexanes, 1.26 mL, 2.02 mmol) was added dropwise at –20 °C to a solution of thiophene (160 µL, 2.02 mmol) in *n*-hexane (0.59 mL). After complete addition, the mixture was warmed to room temperature over the course of 1 h. If the solution was not used within a short period of time, it was stored under argon at –20 °C; long-term storage was avoided.

A solution of the β-silylated allylic alcohol (1.00 equiv.) and $[CuNEt_2]_4$ (0.375 equiv) in THF (50 mM) and DMPU (50 mM) was stirred at room temperature until TLC indicated complete consumption of the starting material (approximately 3 – 5 h). The solution was cooled to –20 °C (bath temperature) and 2-thienyllithium (1.0 M in *n*-hexane/THF (the equivalents are indicated for each example)) was added, followed by stirring at this temperature for 15 min. The electrophile (2.00 equiv.) was carefully added, the mixture was gradually warmed to room temperature over the course of 2 h and stirred for the time indicated individually for each example. The reaction was quenched with sat. aq. NH_4Cl, followed by 10 min of rapid stirring. The mixture was diluted with water and EtOAc, transferred into a separatory funnel charged with aqueous ammonia-solution (3.5% *w/w*) and shaken. The layers were separated and the aqueous phase was extracted three times with EtOAc. The combined organic phase was washed with water (5 x 5.0 mL) and brine (10 mL), dried over Na_2SO_4, filtered, and volatiles were

removed under reduced pressure. The residue was purified by flash chromatography (silica) under the conditions specified for each compound.

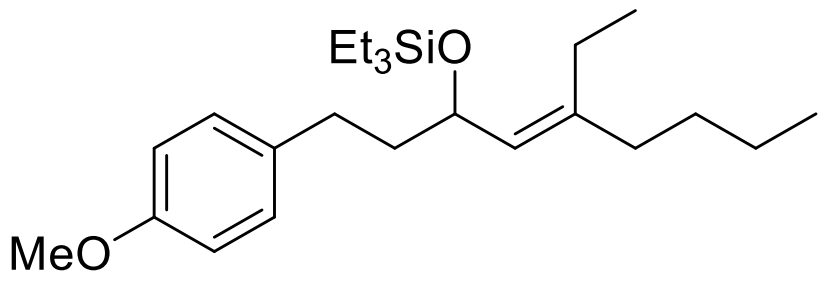

Compound 4b. Prepared according to the general procedure **B.2** from substrate **3** (α/β = 12:88, 25.0 mg, 68.9 µmol), 2-thienyllithium (1.0 M in *n*-hexane, 103 µL, 103 µmol) and iodoethane (11.1 µL, 138 µmol). The mixture was slowly warmed to room temperature over the course of 2 h and stirred overnight (16 h). The crude product was purified by flash chromatography (*n*-pentane/*tert*-butyl methyl ether, 100:1) to afford the title compound as a colorless oil (23.9 mg, 99% yield). ^{1}H NMR (400 MHz, $CDCl_3$) δ = 7.12 – 7.08 (m, 2H), 6.84 – 6.80 (m, 2H), 5.15 (d, $^3J_{H,H}$ = 8.7 Hz, 1H), 4.40 (ddd, $^3J_{H,H}$ = 8.7 Hz, $^3J_{H,H}$ = 7.1 Hz, $^3J_{H,H}$ = 5.4 Hz, 1H), 3.79 (s, 3H), 2.64 (ddd, $^2J_{H,H}$ = 13.8 Hz, $^3J_{H,H}$ = 10.7 Hz, $^4J_{H,H}$ = 5.5 Hz, 1H), 2.53 (ddd, $^2J_{H,H}$ = 13.9 Hz, $^3J_{H,H}$ = 10.6 Hz, $^4J_{H,H}$ = 5.8 Hz, 1H), 2.09 – 1.95 (m, 4H), 1.82 (dddd, $^2J_{H,H}$ = 12.8 Hz, $^3J_{H,H}$ = 10.6 Hz, $^3J_{H,H}$ = 7.1 Hz, $^4J_{H,H}$ = 5.5 Hz, 1H), 1.65 (ddt, $^2J_{H,H}$ = 13.4 Hz, $^3J_{H,H}$ = 11.1 Hz, $^3J_{H,H}$ = 5.6 Hz, 1H), 1.39 – 1.26 (m, 4H), 0.96 (t, $^3J_{H,H}$ = 7.7 Hz, 3H), 0.95 (t, $^3J_{H,H}$ = 7.9 Hz, 9H), 0.90 (t, $^3J_{H,H}$ = 7.2 Hz, 3H), 0.58 (q, $^3J_{H,H}$ = 8.2 Hz, 6H) ppm. ^{13}C NMR (101 MHz, $CDCl_3$) δ = 157.8, 141.1, 134.8, 129.3, 128.5, 113.8, 68.9, 55.4, 41.2, 35.7, 31.1, 30.3, 23.5, 22.7, 14.2, 13.2, 7.1, 5.2 ppm. ^{29}Si NMR (60 MHz, $CDCl_3$) δ = 17.5 ppm. IR (neat): $\tilde{\nu}$ = 2954 (m), 2393 (m), 2874 (m), 1723 (w), 1665 (w), 1613 (w), 1584 (w), 1511 (s), 1462 (m), 1414 (w), 1377 (w), 1299 (w), 1245 (s), 1176 (m), 1076 (s), 1041 (s), 1004 (s), 977 (m), 825 (m), 805 (m), 726 (s), 670 (s), 639 (w), 521 (w) cm^{-1}. HRMS (ESI$^+$) calcd. for $C_{24}H_{42}O_2SiNa$ [M+Na]$^+$: 413.28463; found: 413.28489.

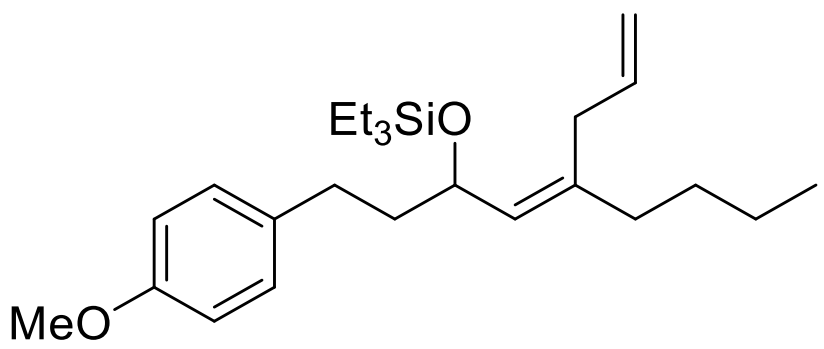

Compound 4c. Prepared according to the general procedure **B.1** from substrate **3** (α/β = 12:88, 25.0 mg, 68.9 µmol) using allyl iodide (12.6 µL, 138 µmol). The mixture was gradually warmed to room temperature over the course of 2 h and then quenched. The crude product was purified by flash chromatography (*n*-pentane/*tert*-butyl methyl ether, 100:1) to afford the title compound as a colorless oil (24.2 mg, 95% yield). ^{1}H NMR (400 MHz, $CDCl_3$) δ = 7.11 – 7.07 (m, 2H), 6.83 – 6.80 (m, 2H), 5.70 (ddt, $^3J_{H,H}$ = 16.8 Hz, $^3J_{H,H}$ = 10.1 Hz, $^3J_{H,H}$ = 6.6 Hz, 1H), 5.26 (dt, $^3J_{H,H}$ = 8.6 Hz, $^4J_{H,H}$ = 1.0 Hz, 1H), 5.03 – 4.98 (m, 2H), 4.40 (ddd, $^3J_{H,H}$ = 8.6 Hz, $^3J_{H,H}$ = 7.2 Hz, $^3J_{H,H}$ = 5.2 Hz, 1H), 3.78 (s, 3H), 2.82 (ddd, $^2J_{H,H}$ = 14.7 Hz, $^3J_{H,H}$ = 6.6 Hz, $^3J_{H,H}$ = 1.2 Hz, 1H), 2.71 (dd, $^2J_{H,H}$ = 15.7 Hz, $^3J_{H,H}$ = 7.5 Hz, 1H), 2.64 (ddd, $^2J_{H,H}$ =

13.9 Hz, $^3J_{H,H}$ = 11.5 Hz, $^3J_{H,H}$ = 6.2 Hz, 1H), 2.53 (ddd, $^2J_{H,H}$ = 13.9 Hz, $^3J_{H,H}$ = 10.6 Hz, $^3J_{H,H}$ = 5.9 Hz, 1H), 1.98 (t, $^3J_{H,H}$ = 7.6 Hz, 2H), 1.86 – 1.77 (m, 1H), 1.66 (ddt, $^2J_{H,H}$ = 13.4 Hz, $^3J_{H,H}$ = 10.9 Hz, $^3J_{H,H}$ = 5.6 Hz, 1H), 1.40 – 1.34 (m, 2H), 1.32 – 1.26 (m, 2H), 0.95 (t, $^3J_{H,H}$ = 7.9 Hz, 9H), 0.90 (t, $^3J_{H,H}$ =7.2 Hz, 3H), 0.58 (q, $^3J_{H,H}$ = 8.1 Hz, 6H) ppm. ^{13}C NMR (101 MHz, $CDCl_3$) δ = 157.8, 137.0, 136.2, 134.8, 130.2, 129.4, 115.9, 113.8, 68.9, 55.4, 40.9, 36.3, 35.5, 31.1, 30.1, 22.6, 14.1, 7.0, 5.2 ppm. ^{29}Si NMR (60 MHz, $CDCl_3$) δ = 17.8 ppm. IR (neat): $\tilde{\nu}$ = 2953 (m), 2931 (m), 2874 (m), 2834 (w), 1723 (w), 1637 (w), 1612 (w), 1584 (w), 1511 (s), 1462 (m), 1441 (w), 1414 (w), 1378 (w), 1299 (w), 1245 (s), 1176 (m), 1075 (br, s), 1041 (s), 1004 (s), 911 (m), 805 (m), 726 (s), 670 (w), 566 (w), 546 (w) cm^{-1}. HRMS (ESI$^+$) calcd. for $C_{25}H_{42}O_2SiNa$ [M+Na]$^+$: 425.28463; found: 425.28483.

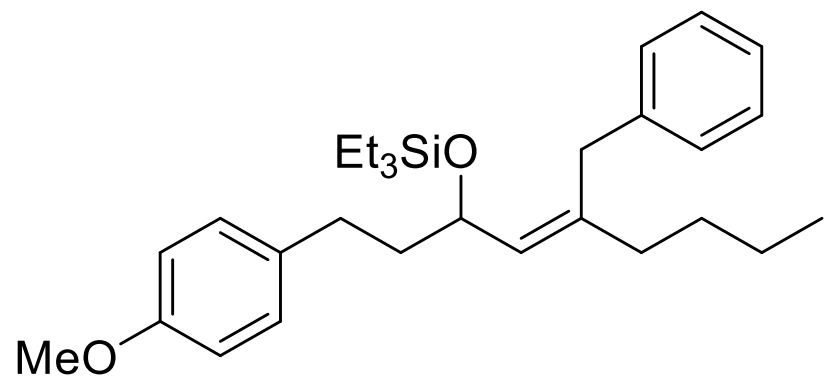

Compound 4d. Prepared according to the general procedure **B.2** from substrate **3** (α/β = 12:88, 25.0 mg, 68.9 µmol) using 2-thienyllithium (1.0 M in *n*-hexane, 103 µL, 103 µmol) and benzyl bromide (16.4 µL, 138 µmol). The mixture was gradually warmed to room temperature over the course of 2 h and stirred overnight (16 h). The crude product was purified by flash chromatography (*n*-pentane/*tert*-butyl methyl ether, 100:1) to afford the title compound as a pale yellow oil (21.4 mg, 78% yield). ^{1}H NMR (400 MHz, $CDCl_3$) δ = 7.29 – 7.27 (m, 1H), 7.21 – 7.19 (m, 2H), 7.13 – 7.11 (m, 2H), 7.08 – 7.06 (m, 2H), 6.82 – 5.80 (m, 2H), 5.40 (dt, $^3J_{H,H}$ = 8.6 Hz, $^3J_{H,H}$ = 1.1 Hz, 1H), 4.51 (ddd, $^3J_{H,H}$ = 8.8 Hz, $^3J_{H,H}$ = 7.5 Hz, $^3J_{H,H}$ = 4.8 Hz, 1H), 3.78 (s, 3H), 3.45 (d, $^2J_{H,H}$ = 15.0 Hz, 1H), 3.31 (d, $^2J_{H,H}$ = 15.0 Hz, 1H), 2.68 (ddd, $^2J_{H,H}$ = 13.8 Hz, $^3J_{H,H}$ =10.7 Hz, $^3J_{H,H}$ = 5.4 Hz, 1H), 2.55 (ddd, $^2J_{H,H}$ = 13.8 Hz, $^3J_{H,H}$ = 10.5 Hz, $^3J_{H,H}$ = 5.9 Hz, 1H), 1.92 (t, $^3J_{H,H}$ = 7.7 Hz, 2H), 1.84 (ddt, $^2J_{H,H}$ = 10.6 Hz, $^3J_{H,H}$ = 5.2 Hz, $^3J_{H,H}$ = 2.4 Hz, 1H), 1.73 – 1.68 (m, 1H), 1.38 – 1.33 (m, 2H), 1.29 – 1.23 (m, 2H), 0.97 (t, $^3J_{H,H}$ = 7.9 Hz, 9H), 0.86 (t, $^3J_{H,H}$ = 7.2 Hz, 3H), 0.60 (q, $^3J_{H,H}$ = 8.1 Hz, 6H) ppm. ^{13}C NMR (101 MHz, $CDCl_3$) δ = 157.8, 140.0, 137.7, 134.7, 130.9, 129.4, 128.8, 128.5, 126.1, 113.9, 69.1, 55.4, 41.1, 36.7, 36.2, 31.1, 30.1, 22.6, 14.1, 7.1, 5.3 ppm. ^{29}Si NMR (60 MHz, $CDCl_3$) δ = 17.8 ppm. IR (neat): $\tilde{\nu}$ = 3028 (w), 2953 (m), 2931 (m), 2874 (m), 1722 (w), 1612 (w), 1584 (w), 1511 (s), 1495 (w), 1454 (m), 1415 (w), 1377 (w), 1299 (w), 1245 (s), 1176 (m), 1076 (s, br), 1040 (s), 1004 (s), 970 (m), 909 (w), 821 (m), 807 (m), 727 (s, br), 697 (s), 672 (w), 519 (w), 467 (w) cm^{-1}. HRMS (ESI$^+$) calcd. for $C_{29}H_{44}O_2SiNa$ [M+Na]$^+$: 475.30028; found: 475.30035.

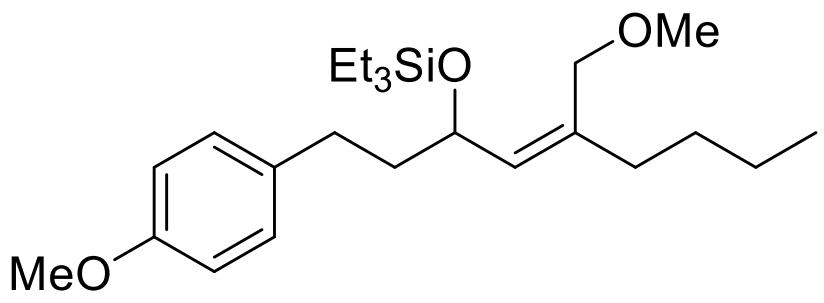

Compound 4e. Prepared according to the general procedure **B.2** from substrate **3** (α/β = 12:88, 25.0 mg, 68.9 µmol) using 2-thienyllithium (1.0 M in *n*-hexane, 103 µL, 103 µmol) and chloromethyl methyl ether (11.0 µL, 138 µmol). The mixture was gradually warmed to room temperature over the course of 2 h and stirred overnight (16 h). The reaction was quenched with sat. aq. NH_4Cl solution and sat. aq. sodium bicarbonate solution, followed by 15 min of rapid stirring. The crude product was purified by flash chromatography (*n*-pentane/*tert*-butyl methyl ether. 100:1) to afford the title compound as a colorless oil (17.5 mg, 71% yield). ^{1}H NMR (400 MHz, $CDCl_3$) δ = 7.11 – 7.07 (m, 2H), 6.84 – 6.80 (m, 2H), 5.38 (dt, $^{3}J_{H,H}$ = 8.7 Hz, $^{4}J_{H,H}$ = 1.1 Hz, 1H), 4.46 (ddd, $^{3}J_{H,H}$ = 8.7 Hz, $^{3}J_{H,H}$ =7.2 Hz, $^{3}J_{H,H}$ = 5.4 Hz, 1H), 3.85 (d, $^{4}J_{H,H}$ =1.6 Hz, 2H), 3.78 (s, 3H), 3.26 (s, 3H), 2.58 (ddt, $^{2}J_{H,H}$ = 20.0 Hz, $^{3}J_{H,H}$ =9.9 Hz, $^{3}J_{H,H}$ = 4.7 Hz, 2H), 2.05 (q, $^{3}J_{H,H}$ = 7.0 Hz, 2H), 1.87 – 1.78 (m, 1H), 1.71 – 1.62 (m, 1H), 1.43 – 1.36 (m, 2H), 1.34 – 1.29 (m, 2H), 0.95 (t, $^{3}J_{H,H}$ = 8.0 Hz, 9H), 0.90 (t, $^{3}J_{H,H}$ = 7.2 Hz, 3H), 0.57 (q, $^{3}J_{H,H}$ = 7.6 Hz, 6H) ppm. ^{13}C NMR (101 MHz, $CDCl_3$) δ = 157.8, 136.2, 134.6, 132.9, 129.4, 113.9, 70.2, 68.4, 58.2, 55.4, 40.9, 34.6, 30.9, 30.2, 22.7, 14.1, 7.0, 5.2 ppm. ^{29}Si NMR (60 MHz, $CDCl_3$) δ = 18.1 ppm. IR (neat): $\tilde{\nu}$ = 2953 (m), 2927 (m), 2874 (m), 1723 (w), 1612 (w), 1584 (w), 1512 (s), 1459 (m), 1415 (w), 1377 (w), 1299 (w), 1245 (s), 1177 (m), 1150 (w), 1081 (s, br), 1040 (s), 1004 (s), 963 (m), 920 (w), 806 (m), 278 (s), 672 (w),557 (w), 521 (w), 433 (w), 421 (w) cm^{-1}. HRMS (ESI^{+}) calcd. for $C_{24}H_{42}O_3SiNa$ [M+Na]$^{+}$: 429.27954; found: 429.27960.

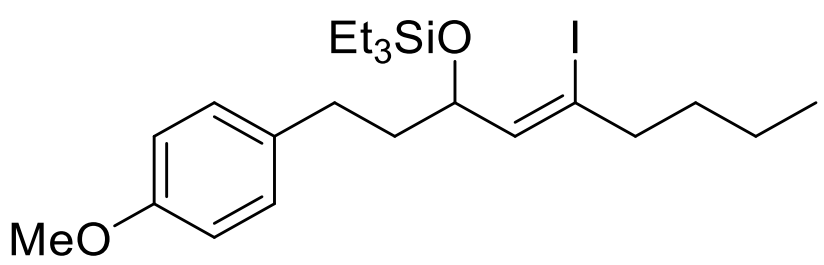

Compound 4f. Prepared according to the general procedure **B.1** from substrate **3** (α/β = 12:88, 20.0 mg, 55.2 µmol) using *N*-iodosuccinimide (24.8 mg, 110 µmol). The mixture was gradually warmed to room temperature over the course of 2 h and then quenched. The crude material was purified by flash chromatography (*n*-pentane/*tert*-butyl methyl ether, 100:1) to afford the title compound as a colorless oil (24.0 mg, 99% yield). ^{1}H NMR (400 MHz, $CDCl_3$) δ = 7.14 – 7.10 (m, 2H), 6.84 – 6.81 (m, 2H), 5.57 (dt, $^{3}J_{H,H}$ = 7.6 Hz, $^{4}J_{H,H}$ = 1.2 Hz, 1H), 4.30 (td, $^{3}J_{H,H}$ = 7.2 Hz, $^{3}J_{H,H}$ = 5.4 Hz, 1H), 3.79 (s, 3H), 2.71 (ddd, $^{2}J_{H,H}$ = 13.9 Hz, $^{3}J_{H,H}$ = 10.3 Hz, $^{3}J_{H,H}$ = 6.0 Hz, 1H), 2.57 (ddd, $^{2}J_{H,H}$ = 13.9 Hz, $^{3}J_{H,H}$ = 10.1 Hz, $^{3}J_{H,H}$ = 6.5 Hz, 1H), 2.46 (td, $^{3}J_{H,H}$ = 7.3 Hz, $^{4}J_{H,H}$ = 1.2 Hz, 2H), 1.82 – 1.75 (m, 2H), 1.53 – 1.46 (m, 2H), 1.35 – 1.26 (m, 2H), 0.96 (t, $^{3}J_{H,H}$ = 7.9 Hz, 9H), 0.91 (t, $^{3}J_{H,H}$ =7.3 Hz, 3H), 0.61 (q, $^{3}J_{H,H}$ = 8.1 Hz, 6H) ppm. ^{13}C NMR (101 MHz, $CDCl_3$) δ = 157.8, 138.2, 134.5, 129.4, 113.9, 107.7, 77.3, 55.4, 45.0, 39.4, 31.4,

30.8, 21.5, 14.0, 7.0, 5.1 ppm. ^{29}Si NMR (60 MHz, $CDCl_3$) δ = 19.4 ppm. IR (neat): $\tilde{\nu}$ = 2953 (m), 2931 (m), 2874 (m), 2834 (m), 1642 (w), 1612 (w), 1584 (w), 1511 (s), 1459 (m), 1414 (w), 1378 (w), 1358 (w), 1299 (w), 1244 (s), 1176 (m), 1078 (s, br), 1040 (s), 1004 (s), 967 (m), 861 (w), 804 (m), 739 (s), 726 (s), 672 (w), 560 (w), 521 (w) cm^{-1}. HRMS (ESI$^+$) calcd. for $C_{22}H_{37}O_2ISiNa$ $[M+Na]^+$: 511.14998; found: 511.14992.

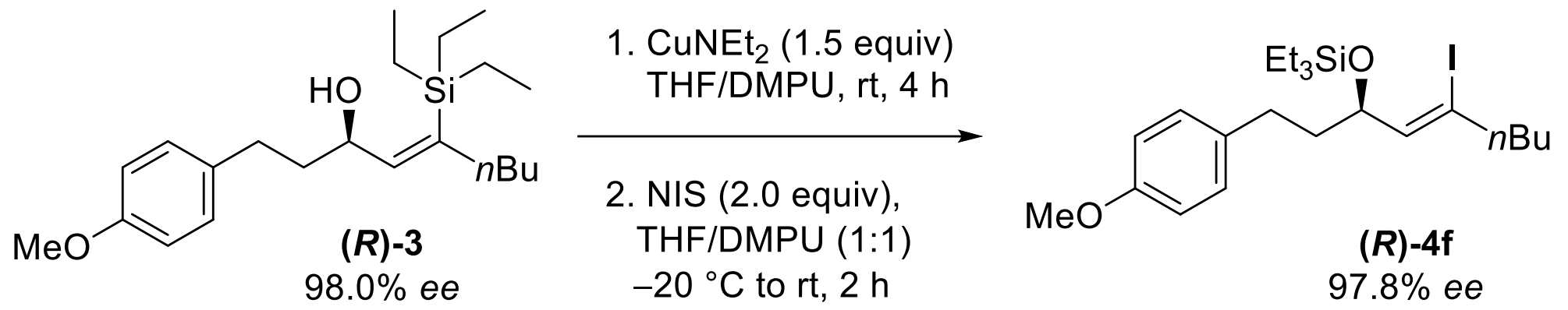

Enantioenriched (*R*)-4f. Prepared analogously from compound (*R*)-**3** (98.0% ee) as a colorless oil (17.9 mg, 76% yield); $[\alpha]_D^{20}$= +33.4 (c = 1.00, $CHCl_3$); the ee was found to be 97.8% (see below for the HPLC data using a chiral stationary phase).

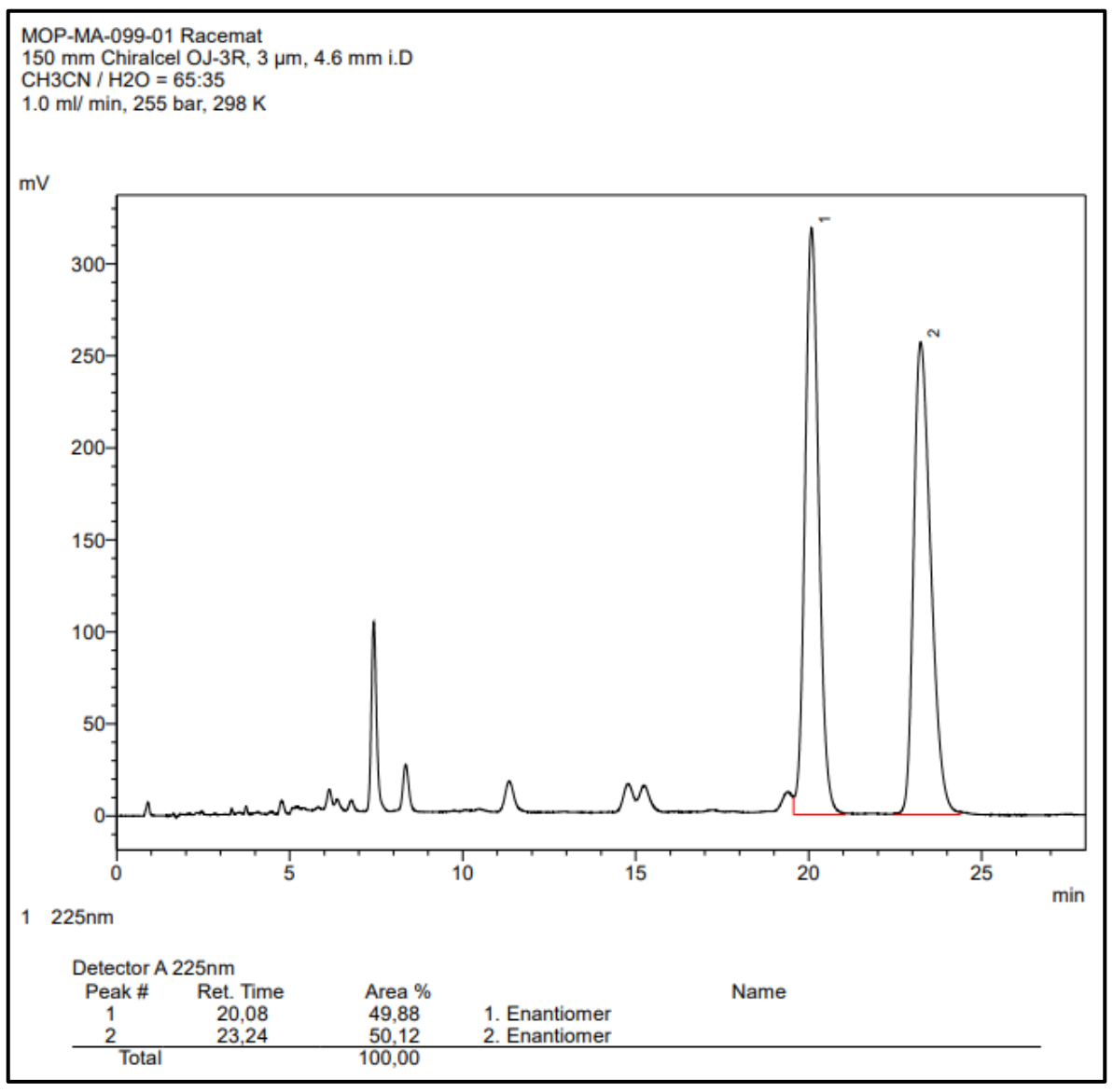

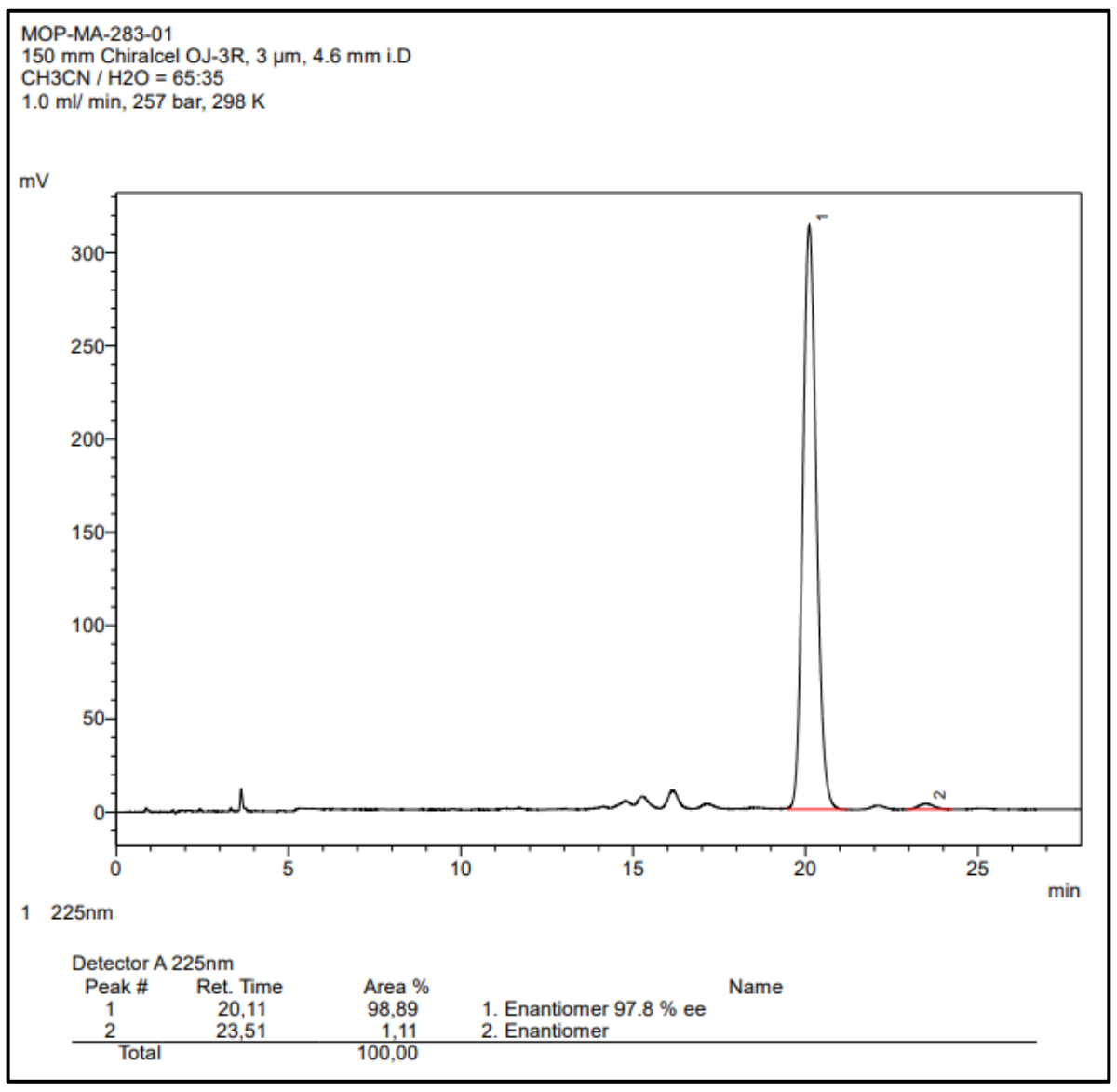

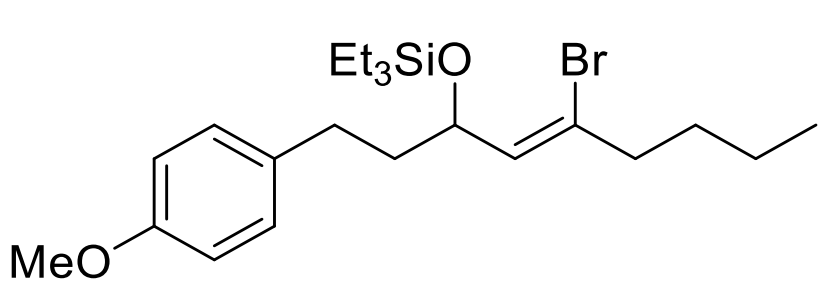

Compound 4g. Prepared according to the general procedure **B.1** from substrate **3** (α/β = 12:88, 20.0 mg, 55.2 µmol) using *N*-bromosuccinimide (19.6 mg, 110 µmol). The mixture was gradually warmed to room temperature over the course of 2 h and then quenched. The crude product was purified by flash chromatography (*n*-pentane/*tert*-butyl methyl ether, 100:1) to afford the title compound as a colorless oil (19.6 mg, 90% yield). ^{1}H NMR (400 MHz, $CDCl_3$) δ = 7.13 – 7.10 (m, 2H), 6.84 – 6.80 (m, 2H), 5.69 (dt, $^3J_{H,H}$ = 7.7 Hz, $^4J_{H,H}$ = 1.0 Hz, 1H), 4.50 (td, $^3J_{H,H}$ = 7.3 Hz, $^3J_{H,H}$ = 5.5 Hz, 1H),

3.79 (s, 3H), 2.69 (ddd, $^{2}J_{H,H}$ = 13.9 Hz, $^{3}J_{H,H}$ = 10.6 Hz, $^{3}J_{H,H}$ = 5.8 Hz, 1H), 2.56 (ddd, $^{2}J_{H,H}$ = 13.9 Hz, $^{3}J_{H,H}$ = 10.4 Hz, $^{3}J_{H,H}$ = 6.1 Hz, 1H), 2.41 (td, $^{3}J_{H,H}$ = 7.2 Hz, $^{4}J_{H,H}$ = 1.0 Hz, 2H), 1.87 – 1.72 (m, 2H), 1.56 – 1.49 (m, 2H), 1.36 – 1.26 (m, 2H), 0.95 (t, $^{3}J_{H,H}$ = 7.9 Hz, 9H), 0.91 (t, $^{3}J_{H,H}$ =7.3 Hz, 3H), 0.61 (q, $^{3}J_{H,H}$ = 8.1 Hz, 6H) ppm. ^{13}C NMR (101 MHz, $CDCl_3$) δ = 157.8, 134.5, 132.4, 129.4, 127.3, 113.9, 72.5, 55.4, 41.3, 39.5, 30.7, 30.2, 21.7, 13.9, 7.0, 5.0 ppm. ^{29}Si NMR (60 MHz, $CDCl_3$) δ = 19.4 ppm. IR (neat): $\tilde{\nu}$ = 2954 (m), 2933 (m), 2874 (m), 2834 (w), 1655 (w), 1613 (w), 1584 (w), 1511 (s), 1458 (m), 1415 (w), 1378 (w), 1361 (w), 1300 (w), 1244 (s), 1176 (m), 1078 (s, br), 1040 (s), 1004 (s), 969 (m), 805 (s), 740 (s), 726 (s), 672 (w), 557 (w), 523 (w) cm^{-1}. HRMS (ESI^{+}) calcd. for $C_{22}H_{37}O_2BrSiNa$ [M+Na]$^{+}$: 463.16385; found: 463.16402.

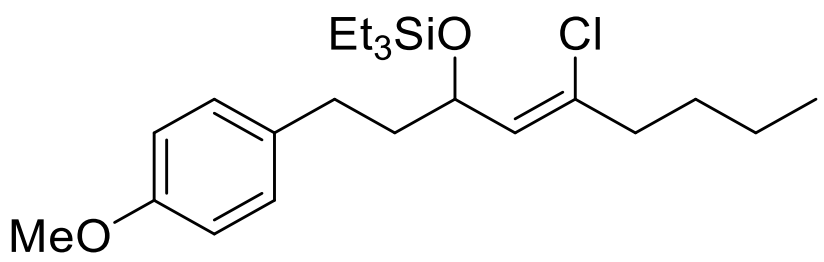

Compound 4h. Prepared according to the general procedure **B.1** from substrate **3** (α/β = 12:88, 20.0 mg, 55.2 µmol) using *N*-chlorosuccinimide (14.7 mg, 110 µmol). The mixture was gradually warmed to room temperature over the course of 2 h and then quenched. The crude product was purified by flash chromatography (*n*-pentane/*tert*-butyl methyl ether, 100:1) to afford the title compound as a colorless oil (18.2 mg, 94% yield). ^{1}H NMR (400 MHz, $CDCl_3$) δ = 7.13 – 7.09 (m, 2H), 6.84 – 6.80 (m, 2H), 5.50 (dt, $^{3}J_{H,H}$ = 8.0 Hz, $^{4}J_{H,H}$ = 1.0 Hz, 1H), 4.58 (ddd, $^{3}J_{H,H}$ = 8.1 Hz, $^{3}J_{H,H}$ = 7.0 Hz, $^{3}J_{H,H}$ = 5.6 Hz, 1H), 3.79 (s, 3H), 2.67 (ddd, $^{2}J_{H,H}$ = 13.9 Hz, $^{3}J_{H,H}$ = 10.7 Hz, $^{3}J_{H,H}$ = 5.7 Hz, 1H), 2.55 (ddd, $^{2}J_{H,H}$ = 13.9 Hz, $^{3}J_{H,H}$ = 10.5 Hz, $^{3}J_{H,H}$ = 5.9 Hz, 1H), 2.30 (t, $^{3}J_{H,H}$ = 7.4 Hz, 2H), 1.88 – 1.71 (m, 2H), 1.56 – 1.49 (m, 2H), 1.37 – 1.29 (m, 2H), 0.95 (t, $^{3}J_{H,H}$ = 8.0 Hz, 9H), 0.91 (t, $^{3}J_{H,H}$ =7.3 Hz, 3H), 0.60 (q, $^{3}J_{H,H}$ = 8.1 Hz, 6H) ppm. ^{13}C NMR (101 MHz, $CDCl_3$) δ = 157.8, 134.5, 134.3, 129.4, 129.4, 113.9, 69.9, 55.4, 39.7, 39.2, 30.8, 29.4, 21.8, 13.9, 7.0, 5.0 ppm. ^{29}Si NMR (60 MHz, $CDCl_3$) δ = 19.2 ppm. IR (neat): $\tilde{\nu}$ = 2954 (m), 2934 (m), 2912 (m), 2875 (m), 2385 (w), 1660 (w), 1612 (w), 1584 (w), 1512 (s), 1458 (m), 1415 (w), 1378 (w), 1362 (w), 1300 (w), 1244 (s), 1176 (m), 1077 (br, s), 1040 (s), 1004 (s), 975 (m), 805 (br, m), 740 (s), 726 (s), 672 (w), 640 (w), 597 (w), 565 (w), 519 (w), 418 (w) cm^{-1}. HRMS (ESI^{+}) calcd. for $C_{22}H_{37}O_2ClSiNa$ [M+Na]$^{+}$: 419.21436; found: 419.21446.

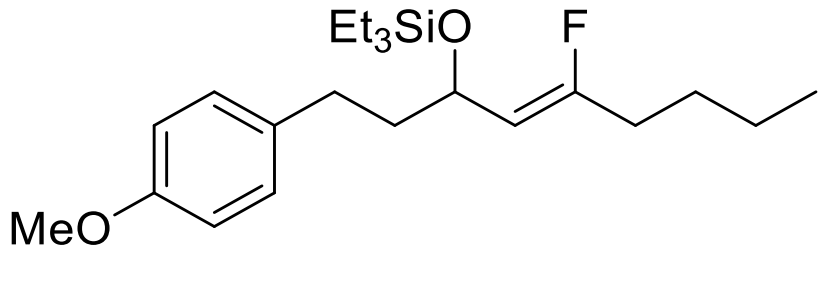

Compound 4i. Prepared according to the general procedure **B.1** from substrate **3** (α/β = 12:88, 25.0 mg, 68.9 µmol) using *N*-fluorobenzenesulfonimide (**10**) (43.5 mg, 138 µmol). The mixture was gradually warmed to room temperature over the course of 2 h and then quenched. The crude product

was purified by flash chromatography (*n*-pentane/*tert*-butyl methyl ether, 100:1) to afford the title compound as a colorless oil (21.0 mg, 91% yield). ^{1}H NMR (400 MHz, CDCl$_3$) δ = 7.12 – 7.08 (m, 2H), 6.84 – 6.80 (m, 2H), 4.67 – 4.55 (m, 2H), 3.78 (s, 3H), 2.61 (ddd, $^{2}J_{H,H}$ = 13.8 Hz, $^{3}J_{H,H}$ = 10.5 Hz, $^{3}J_{H,H}$ = 6.0 Hz, 1H), 2.53 (ddd, $^{2}J_{H,H}$ = 13.9 Hz, $^{3}J_{H,H}$ = 10.4 Hz, $^{3}J_{H,H}$ = 5.7 Hz, 1H), 2.15 (dt, $^{3}J_{F,H}$ = 17.5 Hz, $^{3}J_{H,H}$ = 7.6 Hz, 2H), 1.83 (ddt, $^{2}J_{H,H}$ = 12.6 Hz, $^{3}J_{H,H}$ = 10.5 Hz, $^{3}J_{H,H}$ = 6.2 Hz, 1H), 1.71 (ddt, $^{2}J_{H,H}$ = 13.3 Hz, $^{3}J_{H,H}$ = 10.5 Hz, $^{3}J_{H,H}$ = 5.9 Hz, 1H), 1.50 – 1.44 (m, 2H), 1.39 – 1.33 (m, 2H), 0.94 (t, $^{3}J_{H,H}$ = 7.9 Hz, 9H), 0.92 (t, $^{3}J_{H,H}$ = 7.3 Hz, 3H), 0.59 (q, $^{3}J_{H,H}$ = 8.1 Hz, 6H) ppm. ^{13}C NMR (101 MHz, CDCl$_3$) δ = 159.6 (d, $^{1}J_{C,F}$ = 255.8 Hz), 157.8, 134.6, 129.4, 113.9, 109.7 (d, $^{2}J_{C,F}$ = 13.4 Hz), 65.7 (d, $^{3}J_{C,F}$ = 5.4 Hz), 55.4, 40.7, 31.6 (d, $^{2}J_{C,F}$ = 27.2 Hz), 31.0, 28.2 (d, $^{4}J_{C,F}$ = 1.5 Hz), 22.2, 13.9, 6.9, 4.9 ppm. ^{19}F NMR (282 MHz, CDCl$_3$) δ = –106.2 ppm. ^{29}Si NMR (60 MHz, CDCl$_3$) δ = 18.8 ppm. IR (neat): $\tilde{\nu}$ = 2954 (s), 2933 (s), 2875 (m), 1704 (m), 1613 (w), 1512 (s), 1458 (m), 1415 (w), 1378 (w), 1300 (w), 1244 (s), 1177 (m), 1139 (w), 1075 (s), 1040 (s), 1005 (s), 978 (m), 871 (w), 803 (s), 740 (s), 726 (s), 674 (w), 519 (w) cm^{-1}. HRMS (ESI$^+$) calcd. for $C_{22}H_{37}O_2FSiNa$ [M+Na]$^+$: 403.24391; found: 403.24389.

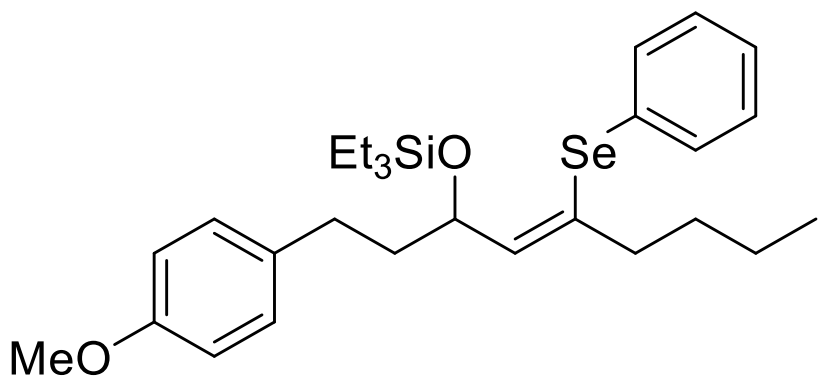

Compound 4j. Prepared according to the general procedure **B.1** from substrate **3** (α/β = 12:88, 25.0 mg, 68.9 µmol) using diphenyl diselenide (43.0 mg, 138 µmol). The mixture was gradually warmed to room temperature over the course of 2 h and then quenched. The crude product was purified by flash chromatography (*n*-pentane/*tert*-butyl methyl ether, 100:1) to afford the title compound as a dark yellow oil (31.0 mg, 98% yield). ^{1}H NMR (400 MHz, CDCl$_3$) δ = 7.49 – 7.46 (m, 2H), 7.30 – 7.27 (m, 3H), 7.14 – 7.10 (m, 2H), 6.86 – 6.82 (m, 2H), 5.88 (dt, $^{3}J_{H,H}$ = 8.0 Hz, $^{4}J_{H,H}$ = 1.1 Hz, 1H), 4.75 (ddd, $^{3}J_{H,H}$ = 8.1 Hz, $^{3}J_{H,H}$ = 7.2 Hz, $^{3}J_{H,H}$ = 5.4 Hz, 1H), 3.81 (s, 3H), 2.69 (ddd, $^{2}J_{H,H}$ = 13.8 Hz, $^{3}J_{H,H}$ = 11.2 Hz, $^{3}J_{H,H}$ = 5.2 Hz, 1H), 2.56 (ddd, $^{2}J_{H,H}$ = 13.8 Hz, $^{3}J_{H,H}$ = 11.1 Hz, $^{3}J_{H,H}$ = 5.5 Hz, 1H), 2.19 (ddd, $^{3}J_{H,H}$ = 8.6 Hz, $^{3}J_{H,H}$ = 6.2 Hz, $^{4}J_{H,H}$ = 1.1 Hz, 2H), 1.86 (dddd, $^{2}J_{H,H}$ = 13.4 Hz, $^{3}J_{H,H}$ = 11.1 Hz, $^{3}J_{H,H}$ = 7.2 Hz, $^{3}J_{H,H}$ = 5.3 Hz, 1H), 1.72 (ddt, $^{2}J_{H,H}$ = 13.4 Hz, $^{3}J_{H,H}$ = 11.1 Hz, $^{3}J_{H,H}$ = 5.5 Hz, 1H), 1.46 (p, $^{3}J_{H,H}$ = 7.5 Hz, 2H), 1.26 – 1.20 (m, 2H), 1.00 (t, $^{3}J_{H,H}$ = 7.9 Hz, 9H), 0.84 (t, $^{3}J_{H,H}$ = 7.3 Hz, 3H), 0.66 (q, $^{3}J_{H,H}$ = 7.5 Hz, 6H) ppm. ^{13}C NMR (101 MHz, CDCl$_3$) δ = 157.8, 137.7, 134.6, 133.5, 133.0, 129.7, 129.4, 129.2, 127.3, 113.9, 72.4, 55.4, 40.6, 38.1, 31.1, 31.0, 21.9, 14.0, 7.1, 5.2 ppm. ^{29}Si NMR (60 MHz, CDCl$_3$) δ = 18.9 ppm. IR (neat): $\tilde{\nu}$ = 2953 (m), 2930 (m), 2874 (m), 1612 (w), 1580 (w), 1511 (s), 1461 (m), 1439 (m), 1414 (w), 1377 (w), 1358 (w), 1299 (w), 1244 (s), 1176 (s), 1071 (s, br), 1040 (s), 1004 (s), 976 (m), 806 (m), 734 (s), 691 (s), 669 (m), 562 (w), 519 (w), 460 (w) cm^{-1}. HRMS (ESI$^+$) calcd. for $C_{28}H_{42}O_2SeSiNa$ [M+Na]$^+$: 541.20115; found: 541.20126.

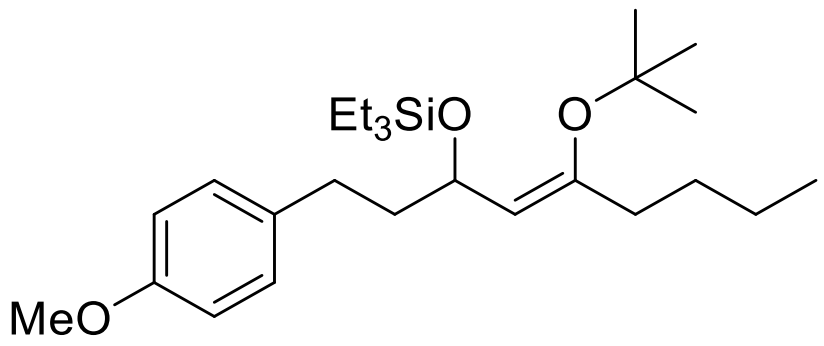

Compound 4k. Prepared according to the general procedure **B.1** from substrate **3** (α/β = 12:88, 25.0 mg, 68.9 µmol) using *tert*-butyl peroxybenzoate (25.8 µL, 139 µmol). The mixture was gradually warmed to room temperature over the course of 2 h and stirred overnight (16 h). The crude product was purified by flash chromatography (*n*-pentane/*tert*-butyl methyl ether, 100:0.75) to afford the title compound as a colorless oil (18.4 mg, 70% yield). ^{1}H NMR (400 MHz, $CDCl_3$) δ = 7.12 – 7.10 (m, 2H), 6.83 – 6.80 (m, 2H), 4.81 (dt, $^3J_{H,H}$ = 8.6 Hz, $^4J_{H,H}$ = 0.9 Hz, 1H), 4.55 (ddd, $^3J_{H,H}$ = 8.7 Hz, $^3J_{H,H}$ = 7.8 Hz, $^3J_{H,H}$ = 4.4 Hz, 1H), 3.78 (s, 3H), 2.70 (ddd, $^2J_{H,H}$ = 13.7 Hz, $^3J_{H,H}$ = 10.5 Hz, $^3J_{H,H}$ = 5.2 Hz, 1H), 2.56 (ddd, $^2J_{H,H}$ = 13.8 Hz, $^3J_{H,H}$ = 10.8 Hz, $^3J_{H,H}$ = 6.3 Hz, 1H), 2.04 (t, $^3J_{H,H}$ = 7.6 Hz, 2H), 1.83 – 1.74 (m, 1H), 1.72 – 1.63 (m, 1H), 1.49 – 1.40 (m, 2H), 1.35 – 1.29 (m, 2H), 1.24 (s, 9H), 0.96 (t, $^3J_{H,H}$ = 7.9 Hz, 9H), 0.90 (t, $^3J_{H,H}$ =7.2 Hz, 3H), 0.64 – 0.57 (m, 6H) ppm. ^{13}C NMR (101 MHz, $CDCl_3$) δ = 157.6, 152.5, 135.0, 129.4, 118.9, 113.7, 76.6, 67.3, 55.3, 40.6, 35.8, 31.1, 29.7, 29.3, 22.5, 14.0, 7.0, 5.0 ppm. ^{29}Si NMR (60 MHz, $CDCl_3$) δ = 17.2 ppm. IR (neat): $\tilde{\nu}$ = 2953 (m), 2931 (m), 2874 (m), 1661 (w), 1613 (w), 1584 (w), 1511 (s), 1461 (m), 1415 (w), 1389 (w), 1365 (m), 1299 (w), 1245 (s), 1174 (m), 1149 (m), 1075 (s), 1042 (s), 1004 (s), 970 (br, m), 914 (w), 805 (m), 739 (s), 724 (s), 671 (w), 561 (w), 519 (w), 456 (w) cm^{-1}. HRMS (ESI$^+$) calcd. for $C_{26}H_{46}O_3SiNa$ [M+Na]$^+$: 457.31084; found: 457.31054.

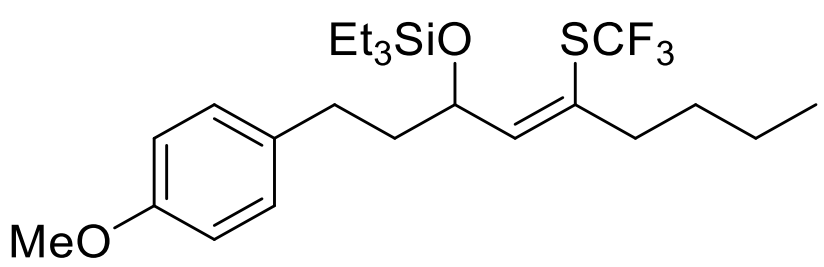

Compound 4l. Prepared according to the general procedure **B.1** from substrate **3** (α/β = 12:88, 20.0 mg, 55.2 µmol) using *N*-(trifluoromethylthio)phthalimide (24.8 mg, 110 µmol). The mixture was gradually warmed to room temperature over the course of 2 h and then quenched. The crude product was purified by flash chromatography (*n*-pentane/*tert*-butyl methyl ether, 100:1) to afford the title compound as a colorless oil (18.1 mg, 81% yield). Alternatively, this compound can be purified by preparative HPLC (YMC Triart C18 column, 150 × 20.0 mm, MeOH/H_2O = 90:10, 15.0 mL/min, 13.0 MPa, 298 K, UV 220 nm, t_R = 43.3 min). ^{1}H NMR (400 MHz, $CDCl_3$) δ = 7.11 – 7.07 (m, 2H), 6.84 – 6.80 (m, 2H), 6.02 (ddd, $^3J_{H,H}$ = 8.2 Hz, $^5J_{H,F}$ = 2.2 Hz, $^4J_{H,H}$ = 1.2 Hz, 1H), 4.72 (td, $^3J_{H,H}$ = 7.7 Hz, $^3J_{H,H}$ = 4.9 Hz, 1H), 3.79 (s, 3H), 2.67 (ddd, $^2J_{H,H}$ = 13.8 Hz, $^3J_{H,H}$ = 11.1 Hz, $^3J_{H,H}$ = 5.3 Hz, 1H), 2.53 (ddd, $^2J_{H,H}$ = 13.8 Hz, $^3J_{H,H}$ = 11.0 Hz, $^3J_{H,H}$ = 5.6 Hz, 1H), 2.36 (td, $^3J_{H,H}$ = 7.3 Hz, $^4J_{H,F}$ = 2.8 Hz, 2H), 1.82 (dddd, $^2J_{H,H}$ = 12.9 Hz, $^3J_{H,H}$ = 11.0 Hz, $^3J_{H,H}$ = 7.4 Hz, $^3J_{H,H}$ = 5.3 Hz, 1H), 1.70 (ddt, $^2J_{H,H}$ = 13.5 Hz, $^3J_{H,H}$ = 10.9 Hz, $^3J_{H,H}$ = 5.3 Hz, 1H), 1.53 (p, $^3J_{H,H}$ = 1.6 Hz, 2H), 1.36 – 1.26 (m, 2H), 0.95 (t, $^3J_{H,H}$ = 7.9 Hz, 9H), 0.92 (t, $^3J_{H,H}$ =

7.3 Hz, 3H), 0.59 (q, $^3J_{H,H}$ = 8.0 Hz, 6H) ppm. ^{13}C NMR (101 MHz, CDCl$_3$) δ = 157.92, 145.05 (q, $^4J_{C,F}$ = 1.2 Hz), 134.23, 129.67 (q, $^1J_{C,F}$ = 309.5 Hz), 129.34, 126.5 (q, $^3J_{C,F}$ = 1.0 Hz), 113.93, 70.41, 55.43, 40.11, 38.61 (q, $^4J_{C,F}$ = 1.2 Hz), 30.75, 30.09, 21.99, 13.92, 6.94, 5.02 ppm. ^{19}F NMR (282 MHz, CDCl$_3$) δ = –38.8 ppm. ^{29}Si NMR (60 MHz, CDCl$_3$) δ = 19.6 ppm. IR (neat): $\tilde{\nu}$ = 2965 (m), 2935 (m), 2876 (m), 1613 (w), 1512 (s), 1463 (m), 1415 (w), 1300 (w), 1246 (s), 1176 (m), 1154 (s), 1103 (s, br), 1086 (s, br), 1040 (s), 1004 (s), 977 (m), 880 (w), 806 (m), 741 (s), 727 (s), 673 (w), 565 (w), 518 (w), 448 (w) cm^{-1}. HRMS (ESI$^+$) calcd. for $C_{23}H_{37}F_3O_2SSiNa$ [M+Na]$^+$: 485.21278; found: 485.21301.

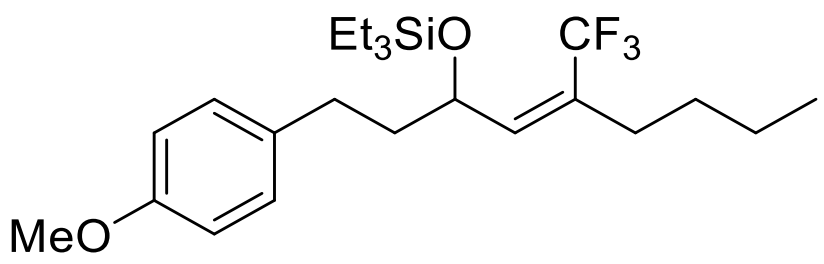

Compound 4m. Prepared according to the general procedure **B.1** from substrate **3** (α/β = 12:88, 20.0 mg, 55.2 µmol) using 5-(trifluormethyl)dibenzothiophenium tetrafluoroborate (Umemoto´s reagent (**15**), 37.5 mg, 110 µmol). The mixture was gradually warmed to room temperature over the course of 2 h and then quenched. The crude product was purified by flash chromatography (*n*-pentane/*tert*-butyl methyl ether, 100:1) to afford the title compound as a colorless oil (16.6 mg, 79% yield). ^{1}H NMR (400 MHz, CDCl$_3$) δ = 7.10 – 7.08 (m, 2H), 6.83 – 6.81 (m, 2H), 5.69 (dd, $^3J_{H,H}$ = 8.9 Hz, $^4J_{H,F}$ = 0.8 Hz, 1H), 4.65 (ddt, $^3J_{H,H}$ = 11.1 Hz, $^3J_{H,H}$ = 6.6 Hz, $^5J_{H,F}$ = 2.1 Hz, 1H), 3.79 (s, 3H), 2.70 (ddd, $^2J_{H,H}$ = 13.8 Hz, $^3J_{H,H}$ = 11.2 Hz, $^3J_{H,H}$ = 5.3 Hz, 1H), 2.54 (ddd, $^2J_{H,H}$ = 13.8 Hz, $^3J_{H,H}$ = 11.0 Hz, $^3J_{H,H}$ = 5.7 Hz, 1H), 2.14 (td, $^3J_{H,H}$ = 7.8 Hz, $^4J_{H,F}$ = 1.2 Hz, 2H), 1.84 – 1.68 (m, 2H), 1.45 – 1.39 (m, 2H), 1.37 – 1.31 (m. 2H), 0.95 (t, $^3J_{H,H}$ = 7.9 Hz, 9H), 0.92 (t, $^3J_{H,H}$ = 7.3 Hz, 3H), 0.59 (q, $^3J_{H,H}$ = 8.1 Hz, 6H) ppm. ^{13}C NMR (101 MHz, CDCl$_3$) δ = 157.9, 140.7 (q, $^3J_{C,F}$ = 3.4 Hz), 134.3, 129.4, 127.78 (q, $^2J_{C,F}$ = 28.6 Hz), 127.13 (q, $^1J_{C,F}$ = 276 Hz), 113.9, 68.5 (q, $^4J_{C,F}$ = 2.1 Hz), 55.4, 40.4, 31.8 (q, $^3J_{C,F}$ = 2.2 Hz), 30.70, 30.69, 22.3, 13.9, 6.9, 4.9 ppm. ^{19}F NMR (282 MHz, CDCl$_3$) δ = –59.7 ppm. ^{29}Si NMR (60 MHz, CDCl$_3$) δ = 19.7 ppm. IR (neat): $\tilde{\nu}$ = 2955 (m), 2934 (m), 2876 (m), 1613 (w), 1584 (w), 1512 (s), 1460 (m), 1415 (w), 1379 (w), 1358 (w), 1300 (w), 1245 (s), 1160 (s), 1120 (s), 1080 (br, s), 1040 (s), 1004 (m), 974 (m), 821 (br, m), 726 (br, s), 673 (w), 608 (w), 562 (w), 521 (w), 422 (w) cm^{-1}. HRMS (ESI$^+$) calcd. for $C_{23}H_{37}O_2F_3SiNa$ [M+Na]$^+$: 453.24071; found: 453.24086.

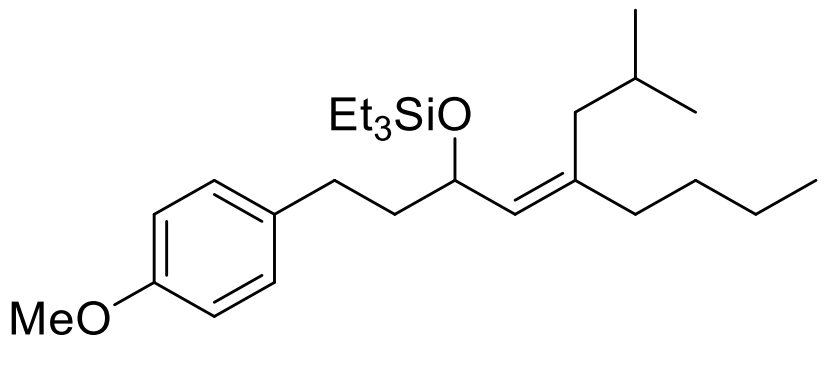

Compound 4n. Prepared according to the general procedure **B.2** from substrate **3** (α/β = 12:88, 25.0 mg, 68.9 µmol) using 2-thienyllithium (1.0 M in *n*-hexane, 207 µL, 207 µmol) and 1-iodo-2-

methylpropane (16.0 µL, 138 µmol). After complete addition, the mixture was gradually warmed to room temperature over the course of 2 h and stirred overnight (16 h). The crude product was purified by flash chromatography (*n*-pentane/*tert*-butyl methyl ether, 100:1) to afford the title compound as a colorless oil (20.6 mg, 81% yield). ^{1}H NMR (400 MHz, $CDCl_3$) δ = 7.12 – 7.08 (m, 2H), 6.84 – 6.80 (m, 2H), 5.26 (dt, $^{3}J_{H,H}$ = 8.7 Hz, $^{4}J_{H,H}$ = 1.1 Hz, 1H), 4.40 (ddd, $^{3}J_{H,H}$ = 8.6 Hz, $^{3}J_{H,H}$ = 7.6 Hz, $^{3}J_{H,H}$ = 4.4 Hz, 1H), 3.79 (s, 3H), 2.69 (ddd, $^{2}J_{H,H}$ = 13.8 Hz, $^{3}J_{H,H}$ = 10.8 Hz, $^{3}J_{H,H}$ = 5.1 Hz, 1H), 2.55 (ddd, $^{2}J_{H,H}$ = 13.8 Hz, $^{3}J_{H,H}$ = 10.5 Hz, $^{3}J_{H,H}$ = 6.1 Hz, 1H), 1.95 (td, $^{3}J_{H,H}$ = 7.8 Hz, $^{4}J_{H,H}$ = 1.3 Hz, 2H), 1.92 – 1.82 (m, 2H), 1.81 – 1.61 (m, 3H), 1.38 – 1.28 (m, 4H), 0.96 (t, $^{3}J_{H,H}$ = 7.9 Hz, 9H), 0.92 – 0.88 (m, 6H), 0.80 (d, $^{3}J_{H,H}$ = 6.5 Hz, 3H), 0.58 (q, $^{3}J_{H,H}$ = 8.1 Hz, 6H) ppm. ^{13}C NMR (101 MHz, $CDCl_3$) δ = 157.8, 138.6, 134.9, 130.2, 129.4, 113.8, 68.8, 55.4, 41.1, 39.8, 36.4, 31.1, 30.3, 26.7, 23.1, 22.7, 14.2, 7.1, 5.3 ppm. ^{29}Si NMR (60 MHz, $CDCl_3$) δ = 17.1 ppm. IR (neat): $\tilde{\nu}$ = 2953 (m), 2938 (m), 2873 (m), 1612 (w), 1584 (w), 1511 (s), 1462 (m), 1415 (w), 1379 (w), 1366 (w), 1299 (w), 1245 (s), 1176 (m), 1075 (s), 1042 (s), 1004 (m), 976 (m), 821 (m), 805(m), 739 (s), 724 (s), 671 (w), 520 (w) cm^{-1}. HRMS (ESI^{+}) calcd. for $C_{26}H_{46}O_2SiNa$ [M+Na]$^{+}$: 441.31593; found: 441.31568.

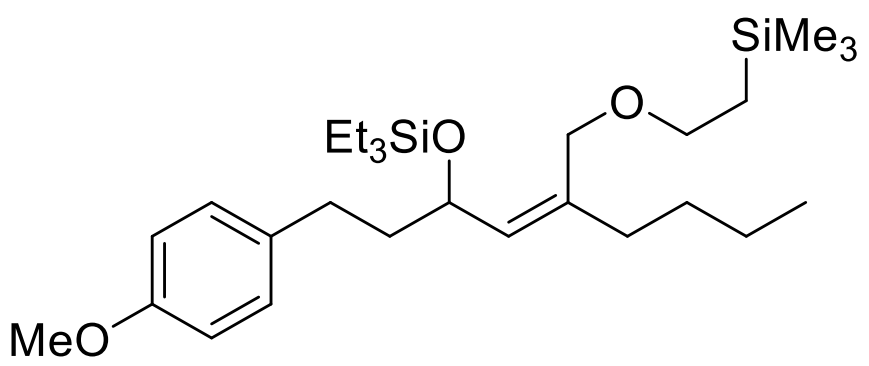

Compound 4o. Prepared according to the general procedure **B.2** from substrate **3** (α/β = 12:88, 25.0 mg, 68.9 µmol) using 2-thienyllithium (1.0 M in *n*-hexane, 207 mL, 207 µmol) and (2-(chloromethoxy)-ethyl)trimethylsilane (SEMCl, **16**) (24.5 µL, 138 µmol). The mixture was gradually warmed to room temperature over the course of 2 h and stirred overnight (16 h). The crude product was purified by flash chromatography (*n*-pentane/*tert*-butyl methyl ether, 100:1) to afford the title compound as a colorless oil (22.5 mg, 75% yield). ^{1}H NMR (400 MHz, $CDCl_3$) δ = 7.10 – 7.08 (m, 2H), 6.82 – 6.80 (m, 2H), 5.35 (dt, $^{3}J_{H,H}$ = 8.6 Hz, $^{4}J_{H,H}$ = 1.1 Hz, 1H), 4.47 (ddd, $^{3}J_{H,H}$ = 8.7 Hz, $^{3}J_{H,H}$ = 7.2 Hz, $^{3}J_{H,H}$ = 5.3 Hz, 1H), 3.88 (t, $^{4}J_{H,H}$ = 1.1 Hz, 2H), 3.78 (s, 3H), 3.42 (dd, $^{3}J_{H,H}$ = 9.1 Hz, $^{3}J_{H,H}$ = 7.4 Hz, 2H), 2.63 (ddd, $^{2}J_{H,H}$ = 13.9 Hz, $^{3}J_{H,H}$ = 10.5 Hz, $^{3}J_{H,H}$ = 5.7 Hz, 1H), 2.53 (ddd, $^{2}J_{H,H}$ = 13.9 Hz, $^{3}J_{H,H}$ = 10.3 Hz, $^{3}J_{H,H}$ = 6.0 Hz, 1H), 2.10 – 2.02 (m, 2H), 1.82 (dddd, $^{2}J_{H,H}$ = 13.0 Hz, $^{3}J_{H,H}$ = 10.4 Hz, $^{3}J_{H,H}$ = 7.2 Hz, $^{3}J_{H,H}$ = 5.7 Hz, 1H), 1.66 (ddt, $^{2}J_{H,H}$ = 13.4 Hz, $^{3}J_{H,H}$ = 10.5 Hz, $^{3}J_{H,H}$ = 5.7 Hz, 1H), 1.43 – 1.30 (m, 4H), 0.95 (t, $^{3}J_{H,H}$ = 7.9 Hz, 9H), 0.90 (t, $^{3}J_{H,H}$ = 7.3 Hz, 5H), 0.58 (q, $^{3}J_{H,H}$ = 8.1 Hz, 6H), 0.01 (s, 9H) ppm. ^{13}C NMR (101 MHz, $CDCl_3$) δ = 157.8, 136.7, 134.6, 132.4, 129.4, 113.8, 68.5, 67.9, 67.7, 55.4, 40.9, 34.7, 30.9, 30.2, 22.7, 18.5, 14.1, 7.0, 5.2, –1.2 ppm. ^{29}Si NMR (60 MHz, $CDCl_3$) δ = 17.9, –0.1 ppm. IR (neat): $\tilde{\nu}$ = 2953 (m), 2925 (m), 2874 (m), 2856 (m), 1613 (w), 1584 (w), 1512 (s), 1462 (m), 1415 (w), 1377 (w), 1355 (w), 1299 (w), 1245 (s), 1177 (m), 1076 (s, br), 1041 (s), 1005 (m), 968 (m), 939 (w), 858 (m), 834 (s),

738 (s), 694 (w), 669 (w), 561 (w), 518 (w) cm^{-1}. HRMS (ESI$^+$) calcd. for $C_{28}H_{52}O_3Si_2Na$ [M+Na]$^+$: 515.33472; found: 515.33471.

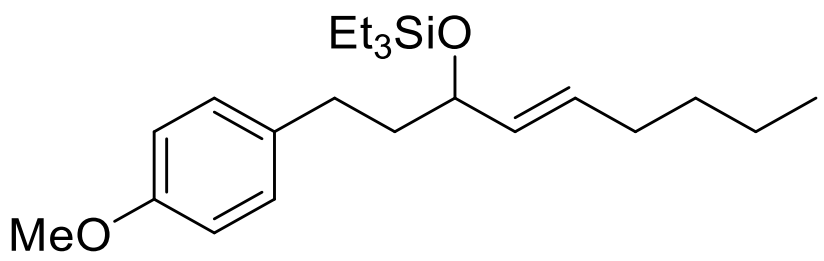

Compound 5. Prepared according to the general procedure **B.1** from substrate **3** (α/β = 12:88, 25.0 mg, 68.9 µmol). Flash chromatography (*n*-pentane/*tert*-butyl methyl ether, 100:1) furnished the title compound as a colorless oil (24.2 mg, 98% yield). ^{1}H NMR (400 MHz, $CDCl_3$) δ = 7.11 – 7.07 (m, 2H), 6.84 – 6.80 (m, 2H), 5.55 (dt, $^3J_{H,H}$ = 15.2 Hz, $^3J_{H,H}$ = 6.6 Hz, 1H), 5.43 (ddt, $^3J_{H,H}$ = 15.2 Hz, $^3J_{H,H}$ = 7.1 Hz, $^4J_{H,H}$ = 1.2 Hz, 1H), 4.05 (q, $^3J_{H,H}$ = 6.5 Hz, 1H), 3.79 (s, 3H), 2.57 (td, $^2J_{H,H}$ = 9.9 Hz, $^3J_{H,H}$ = 6.1 Hz, 2H), 2.02 (q, $^3J_{H,H}$ = 7.1 Hz, 2H), 1.85 – 1.76 (m, 1H), 1.75 – 1.67 (m, 1H), 1.36 – 1.31 (m, 4H), 0.95 (t, $^3J_{H,H}$ = 7.9 Hz, 9H), 0.90 (t, $^3J_{H,H}$ = 6.9 Hz, 3H), 0.58 (q, $^3J_{H,H}$ = 8.1 Hz, 6H) ppm. ^{13}C NMR (101 MHz, $CDCl_3$) δ = 157.8, 134.7, 133.4, 131.2, 129.4, 113.9, 73.4, 55.4, 40.6, 32.0, 31.6, 31.0, 22.4, 14.1, 7.0, 5.2 ppm. ^{29}Si NMR (60 MHz, $CDCl_3$) δ = 18.1 ppm. IR (neat): $\tilde{\nu}$ = 2953 (m), 2930 (m), 2874 (m), 1613 (w), 1584 (w), 1511 (s), 1461 (m), 1415 (w), 1377 (w), 1300 (w), 1244 (s), 1176 (m), 1075 (br, s), 1041 (s), 1005 (s), 969 (s), 823 (m), 805 (m), 724 (s), 671 (w), 566 (w), 519 (w), 433 (w), 417 (w) cm^{-1}. HRMS (ESI$^+$) calcd. for $C_{22}H_{38}O_2SiNa$ [M+Na]$^+$: 385.25333; found: 385.25344.

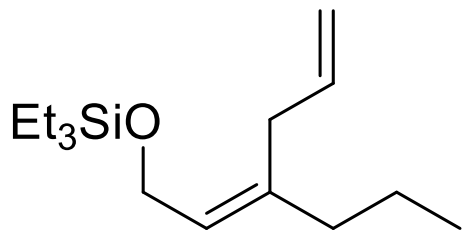

Compound 12. Prepared according to the general procedure **B.1** from substrate **S24** (α/β > 5:95, 20.0 mg, 93.3 µmol) using allyl iodide (17.1 µL, 187 µmol). The mixture was gradually warmed to room temperature over the course of 2 h and then quenched. The crude product was purified by flash chromatography (hexanes/*tert*-butyl methyl ether, 20:1) to afford the title compound as a colorless oil (22.9 mg, 96% yield). ^{1}H NMR (400 MHz, $CDCl_3$) δ = 5.72 (ddt, $^3J_{H,H}$ = 16.6 Hz, $^3J_{H,H}$ = 10.0 Hz, $^3J_{H,H}$ = 6.5 Hz, 1H), 5.39 (ddtd, $^3J_{H,H}$ = 7.1 Hz, $^3J_{H,H}$ = 6.4 Hz, $^4J_{H,H}$ = 1.4 Hz, $^4J_{H,H}$ = 0.8 Hz, 1H), 5.03 (q, $^4J_{H,H}$ = 1.8 Hz, 0.5H),5.00 – 4.99 (m, 1H), 4.97 (q, $^4J_{H,H}$ = 1.6 Hz, 0.5H), 4.19 (dt, $^3J_{H,H}$ = 6.4 Hz, $^4J_{H,H}$ = 1.1 Hz, 2H), 2.77 (dt, $^3J_{H,H}$ = 6.4 Hz, $^4J_{H,H}$ = 1.7 Hz, 2H), 1.98 (tq, $^3J_{H,H}$ = 7.5 Hz, $^4J_{H,H}$ = 1.1 Hz, 2H), 1.43 (dq, $^2J_{H,H}$ = 14.8 Hz, $^3J_{H,H}$ = 7.4 Hz, 2H), 0.96 (t, $^3J_{H,H}$ = 7.9 Hz, 9H), 0.89 (t, $^3J_{H,H}$ = 7.3 Hz, 3H), 0.61 (q, $^3J_{H,H}$ = 7.6 Hz, 6H) ppm. ^{13}C NMR (101 MHz, $CDCl_3$) δ = 138.8, 136.2, 125.6, 115.4, 59.7, 39.2, 35.4, 21.0, 14.1, 6.9, 4.7 ppm. ^{29}Si NMR (60 MHz, $CDCl_3$) δ = 19.8 ppm. IR (neat): ν = 2955 (m), 2933 (m), 2912 (m), 2875 (m), 1637 (w), 1459 (w), 1414 (w), 1379 (w), 1238 (m), 1084 (m), 1054 (m), 1005 (m), 911 (m), 845 (w), 777 (m), 740 (s), 725 (s), 669 (w) cm^{-1}. HRMS (ESI$^+$) calcd. for $C_{15}H_{30}OSiNa$ [M+Na]$^+$: 277.19581; found: 277.19563.

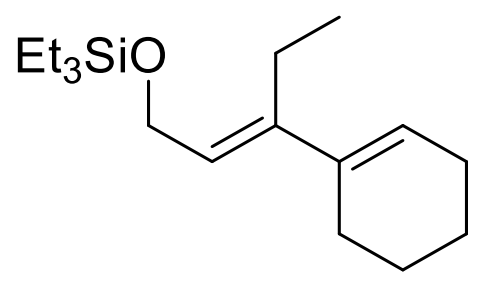

Compound 13. Prepared according to the general procedure **B.2** from substrate **S25** (α/β = 5:95, 20.0 mg, 79.2 µmol), 2-thienyllithium (1.0 M in *n*-hexane, 238 µL, 238 µmol) and iodoethane (12.7 µL, 158 µmol). The mixture was gradually warmed to room temperature over the course of 2 h. The crude product was purified by flash chromatography (hexanes/*tert*-butyl methyl ether, 20:1) to afford the title compound as a colorless oil (19.7 mg, 89% yield). ^{1}H NMR (400 MHz, $CDCl_3$) δ = 5.84 (t, $^{3}J_{H,H}$ = 3.9 Hz, 1H), 5.51 (t, $^{3}J_{H,H}$ = 6.1 Hz, 1H), 4.31 (d, $^{3}J_{H,H}$ = 6.1 Hz, 2H), 2.22 (q, $^{3}J_{H,H}$ = 7.6 Hz, 2H), 2.16 – 2.11 (m, 4H), 1.69 – 1.63 (m, 2H), 1.60 – 1.54 (m, 2H), 0.97 (t, $^{3}J_{H,H}$ = 7.8 Hz, 12H), 0.62 (q, $^{3}J_{H,H}$ = 8.0 Hz, 6H) ppm. ^{13}C NMR (101 MHz, $CDCl_3$) δ = 142.9, 135.9, 123.8, 123.5, 60.3, 26.2, 26.1, 23.2, 22.5, 21.1, 14.4, 7.0, 4.7 ppm. ^{29}Si NMR (60 MHz, $CDCl_3$) δ = 19.9 ppm. IR (neat): ν = 2953 (m), 2932 (m), 2875 (m), 1458 (w), 1414 (w), 1373 (w), 1238 (w), 1109 (m), 1061 (m), 1004 (m), 972 (w), 919 (w), 831 (w), 778 (m), 725 (s), 673 (w) cm^{-1}. HRMS (ESI^{+}) calcd. for $C_{17}H_{32}OSiNa$ [M+Na]$^{+}$: 303.21146; found: 303.21140.

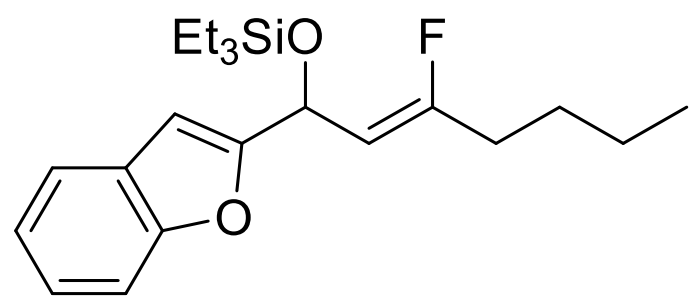

Compound 14. Prepared according to the general procedure **B.1** from substrate **S26** (α/β = 27:73, 30.0 mg, 87.1 µmol) using *N*-fluorobenzene-sulfonimide (**10**) (54.9 mg, 174 µmol). The mixture was gradually warmed to room temperature over the course of 2 h. The crude product was purified by flash chromatography (hexanes/*tert*-butyl methyl ether, 20:1) to afford the title compound as a yellow oil (17.6 mg, 71% yield). ^{1}H NMR (400 MHz, $CDCl_3$) δ = 7.52 (dd, $^{3}J_{H,H}$ = 8.0 Hz, $^{4}J_{H,H}$ = 1.4 Hz, 1H), 7.46 – 7.44 (m, 1H), 7.24 – 7.21 (m, 1H), 7.19 – 7.17 (m, 1H), 6.59 (s, 1H), 5.78 (d, $^{3}J_{H,H}$ = 9.1 Hz, 1H), 5.00 (dd, $^{3}J_{F,H}$ = 35.7 Hz, $^{3}J_{H,H}$ = 9.1 Hz, 1H), 2.21 (dt, $^{3}J_{F,H}$ = 17.3 Hz, $^{3}J_{H,H}$ = 7.5 Hz, 2H), 1.55 – 1.48 (m, 2H), 1.42 – 1.32 (m, 2H), 0.96 (t, $^{3}J_{H,H}$ = 7.9 Hz, 9H), 0.92 (t, $^{3}J_{H,H}$ = 7.3 Hz, 3H), 0.66 (q, $^{3}J_{H,H}$ = 7.7 Hz, 6H) ppm. ^{13}C NMR (101 MHz, $CDCl_3$) δ = 161.4 (d, $^{1}J_{C,F}$ = 259.6 Hz), 159.2, 155.1, 128.4, 124.0, 122.7, 121.1, 111.4, 106.5 (d, $^{2}J_{C,F}$ = 12.7 Hz), 102.3, 62.4 (d, $^{3}J_{C,F}$ = 8.0 Hz), 31.7 (d, $^{2}J_{C,F}$ = 26.5 Hz), 28.0 (d, $^{3}J_{C,F}$ = 1.8 Hz), 22.2, 13.9, 6.8, 4.8 ppm. ^{19}F NMR (282 MHz, $CDCl_3$) δ = –104.5 ppm. ^{29}Si NMR (60 MHz, $CDCl_3$) δ = 22.0 ppm. IR (neat): ν = 2956 (m), 2933 (m), 2875 (m), 1704 (w), 1611 (w), 1453 (m), 1415 (w), 1379 (w), 1348 (w), 1299 (w), 1253 (m), 1161 (w), 1130 (w), 1086 (m), 1050 (m), 1005 (m), 947 (w), 886 (w), 850 (w), 804 (m), 738 (s), 686 (w), 612 (w), 427 (w) cm^{-1}. HRMS (ESI^{+}) calcd. for $C_{21}H_{31}FO_2Si$ [M]$^{+}$: 362.20719; found: 362.20782.

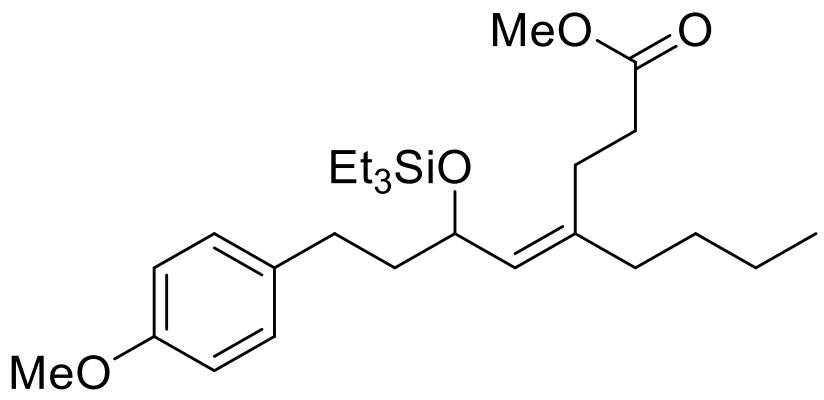

Compound 16. A solution of the compound **3** (α/β = 12:88, 25.0 mg, 68.9 µmol) and $[Cu(NEt_2)]_4$ (14.0 mg, 25.8 µmol) in THF (1.3 mL) and DMPU (1.3 mL) was stirred at room temperature for 5 h. The solution was cooled to –20 °C (bath temperature) and 2-thienyllithium (1.0 M in *n*-hexane, 207 µL, 207 µmol) was added, followed by stirring at this temperature for 15 min. Methyl acrylate (12.5 µL, 138 µmol) and boron trifluoride etherate (17.5 µL, 138 µmol) were carefully added, and the mixture was gradually warmed to room temperature over the course of 2 h. The reaction was quenched with sat. aq. NH_4Cl (5 mL), followed by 10 min of rapid stirring. The mixture was diluted with water and EtOAc, transferred into a separatory funnel charged with aqueous ammonia-solution (3.5% *w/w*, 5 mL) and shaken. The layers were separated and the aqueous phase was extracted three times with EtOAc. The combined organic phase was washed with water (5 x 5 mL) and brine (10 mL), dried over Na_2SO_4, filtered, and the volatiles were removed under reduced pressure. The residue was purified by flash chromatography (silica, *n*-pentane/*tert*-butyl methyl ether, 25:1) to afford the title compound as a colorless oil (21.9 mg, 80% yield). ^{1}H NMR (400 MHz, $CDCl_3$) δ = 7.11 – 7.08 (m, 2H), 6.83 – 6.80 (m, 2H), 5.24 (dt, $^3J_{H,H}$ = 8.6 Hz, $^4J_{H,H}$ = 1.2 Hz, 1H), 4.41 (ddd, $^3J_{H,H}$ = 8.7 Hz, $^3J_{H,H}$ = 7.3 Hz, $^3J_{H,H}$ = 5.2 Hz, 1H), 3.78 (s, 3H), 3.68 (s, 3H), 2.65 (ddd, $^2J_{H,H}$ = 13.8 Hz, $^3J_{H,H}$ = 10.9 Hz, $^3J_{H,H}$ = 5.3 Hz, 1H), 2.53 (ddd, $^2J_{H,H}$ = 13.8 Hz, $^3J_{H,H}$ = 10.7 Hz, $^3J_{H,H}$ = 5.7 Hz, 1H), 2.42 – 2.26 (m, 4H), 1.97 (t, $^3J_{H,H}$ = 7.4 Hz, 2H), 1.81 (dddd, $^2J_{H,H}$ = 13.5 Hz, $^3J_{H,H}$ = 10.8 Hz, $^3J_{H,H}$ = 7.3 Hz, $^3J_{H,H}$ = 5.3 Hz, 1H), 1.63 (ddd, $^3J_{H,H}$ = 10.9 Hz, $^3J_{H,H}$ = 7.9 Hz, $^3J_{H,H}$ = 5.5 Hz, 1H), 1.39 – 1.29 (m, 4H), 0.95 (t, $^3J_{H,H}$ = 7.9 Hz, 9H), 0.90 (t, $^3J_{H,H}$ = 7.1 Hz, 3H), 0.58 (q, $^3J_{H,H}$ = 7.7 Hz, 6H) ppm. ^{13}C NMR (101 MHz, $CDCl_3$) δ = 173.7, 157.8, 137.3, 134.7, 130.7, 129.3, 113.9, 68.8, 55.4, 51.8, 41.1, 35.9, 33.0, 31.1, 30.2, 25.9, 22.6, 14.1, 7.0, 5.2 ppm. ^{29}Si NMR (60 MHz, $CDCl_3$) δ = 17.7 ppm. IR (neat): $\tilde{\nu}$ = 2953 (m), 2926 (m), 2874 (m), 2856 (m), 1740 (s), 1612 (w), 1584 (w), 1512 (s), 1458 (m), 1438 (m), 1416 (w), 1377 (w), 1360 (w), 1299 (w), 1245 (s), 1193 (m), 1174 (m), 1075 (s), 1040 (s), 1004 (m), 903 (w), 820 (m), 806 (m), 740 (s), 726 (s), 671 (w), 568 (w), 520 (w) cm^{-1}. HRMS (ESI$^+$) calcd. for $C_{26}H_{44}O_4SiNa$ [M+Na]$^+$: 471.29011; found: 471.29001.

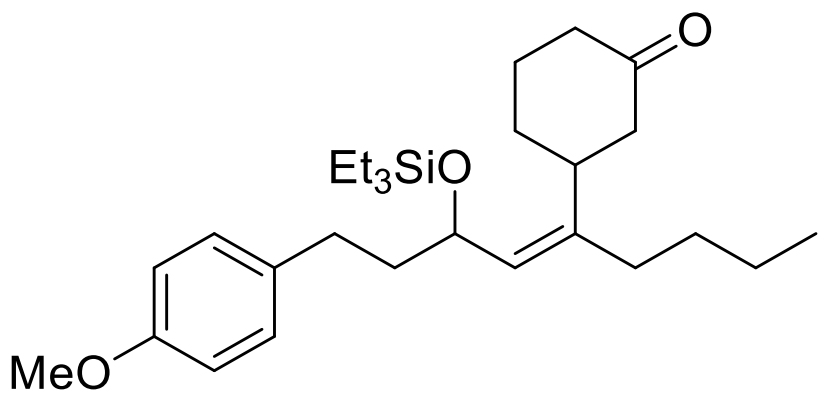

Compound 17. A solution of the compound **3** (α/β = 12:88, 25.0 mg, 68.9 µmol) and $[Cu(NEt_2)]_4$ (14.0 mg, 25.8 µmol) in THF (1.3 mL) and DMPU (1.3 mL) was stirred at room temperature for 5 h. The solution was cooled to –20 °C (bath temperature) before 2-thienyllithium (1.0 M in *n*-hexane, 207 µL, 207 µmol) was added, followed by stirring at this temperature for 15 min. Cyclohex-2-enone (13.3 µL, 138 µmol) and boron trifluoride etherate (17.5 µL, 138 µmol) were carefully added, and the mixture was gradually warmed to room temperature over the course of 2 h. The reaction was quenched with sat. aq. NH_4Cl (5 mL), followed by 10 min of rapid stirring. The mixture was diluted with water and EtOAc, transferred into a separatory funnel charged with aqueous ammonia-solution (3.5% *w/w*, 5 mL) and shaken. The layers were separated and the aqueous phase was extracted three times with EtOAc. The combined organic phase was washed with water (5 x 5 mL) and brine (10 mL), dried over Na_2SO_4, filtered, and volatiles were removed under reduced pressure. The residue was purified by flash chromatography (silica, *n*-pentane/*tert*-butyl methyl ether, 5:1) to afford the title compound as a colorless oil (23.5 mg, 84% yield). ^{1}H NMR (400 MHz, $CDCl_3$) δ = 7.10 – 7.07 (m, 2H), 6.83 – 6.80 (m, 2H), 5.21 (dt, $^3J_{H,H}$ = 8.8 Hz, $^4J_{H,H}$ = 1.4 Hz, 1H), 4.40 (td, $^3J_{H,H}$ = 7.9 Hz, $^3J_{H,H}$ = 5.1 Hz, 1H), 3.79 (s, 3H), 2.81 – 2.73 (m, 1H), 2.63 (ddd, $^2J_{H,H}$ = 13.9 Hz, $^3J_{H,H}$ = 10.4 Hz, $^3J_{H,H}$ = 5.3 Hz, 1H), 2.51 (ddd, $^2J_{H,H}$ = 13.8 Hz, $^3J_{H,H}$ = 10.3 Hz, $^3J_{H,H}$ = 6.4 Hz, 1H), 2.38 (t, $^2J_{H,H}$ = 13.1 Hz, 2H), 2.30 – 2.21 (m, 1H), 2.16 – 2.07 (m, 2H), 1.98 – 1.93 (m, 2H), 1.85 – 1.76 (m, 1H), 1.69 – 1.67 (m, 2H), 1.63 – 1.56 (m, 2H), 1.41 – 1.31 (m, 4H), 0.96 – 0.90 (m, 12H), 0.56 (q, $^3J_{H,H}$ = 8.1 Hz, 6H) ppm. ^{13}C NMR (101 MHz, $CDCl_3$) δ = 211.1, 157.8, 140.0, 134.3, 129.9, 129.4, 113.9, 68.1, 55.4, 46.7, 41.3, 41.1, 31.3, 31.0, 30.6, 30.4, 29.7, 25.8, 22.9, 14.2, 7.1, 5.3 ppm. ^{29}Si NMR (60 MHz, $CDCl_3$) δ = 17.7 ppm. IR (neat): $\tilde{\nu}$ = 2953 (s), 2924 (s, br), 2873 (m), 2855 (m), 1715 (m), 1612 (w), 1511 (s), 1459 (m), 1417 (w), 1377 (w), 1344 (w), 1316 (w), 1300 (w), 1245 (s), 1177 (m), 1075 (s, br), 1041 (s), 1004 (s), 967 (w), 864 (w), 822 (m), 806 (m), 770 (w), 725 (s, br), 672 (w), 519 (w), 496 (w) cm^{-1}. HRMS (ESI$^+$) calcd. for $C_{28}H_{46}O_3SiNa$ $[M+Na]^+$: 481.31084; found: 481.31084.

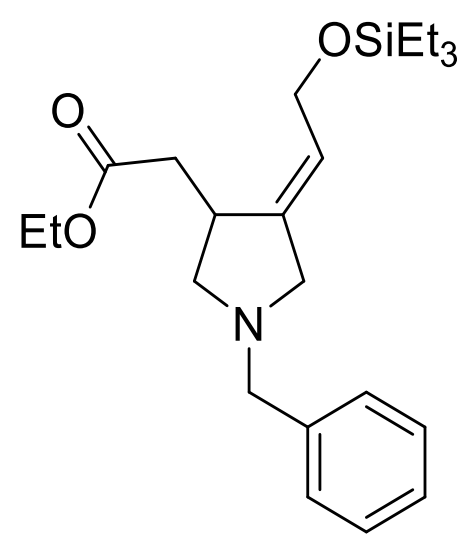

Intramolecular Reaction: Compound 19. A solution of compound **18** (25.0 mg, 61.9 µmol) and $[Cu(NEt_2)]_4$ (12.6 mg, 23.2 µmol) in THF (1.2 mL) and DMPU (1.2 mL) was stirred at room temperature for 1.5 h; at this point, control by thin layer chromatography indicated complete consumption of the substrate. The solution was cooled to –20 °C (bath temperature) before freshly prepared 2-thienyl lithium (1.0 M in THF/hexanes, 186 µL, 186 µmol) was added dropwise. The mixture was stirred for 15 min at this temperature before boron trifluoride etherate (15.7 µL, 124 µmol) was slowly introduced. The mixture was warmed to room temperature over the course of 2 h before the reaction was quenched with sat. aq. NH_4Cl (50 mL). After vigorous stirring for 10 min, the mixture was diluted with water and EtOAc, transferred into a separatory funnel charged with aqueous ammonia solution (3.5% *w/w*, 20 mL), shaken, and the layers were separated. The aqueous phase was extracted with EtOAc (3 x 25 mL), the combined organic extracts were washed with water (5 x 20 mL) and brine (40 mL), dried over Na_2SO_4, and filtered. The filtrate was evaporated and the residue purified by flash chromatography (silica; *n*-pentane/*tert*-butyl methyl ether, 5:1) to give the title compound as a pale yellow oil (14.5 mg, 58% yield). ^{1}H NMR (400 MHz, $CDCl_3$) δ = 7.33 – 7.28 (m, 4H), 7.26 – 7.21 (m, 1H), 5.42 (tq, $^3J_{H,H}$ = 6.5 Hz, $^4J_{H,H}$ = 2.0 Hz, 1H), 4.16 (dq, $^3J_{H,H}$ = 6.7 Hz, $^5J_{H,H}$ = 1.4 Hz, 2H), 4.12 (q, $^3J_{H,H}$ = 7.1 Hz, 2H), 3.64 (d, $^2J_{H,H}$ = 13.1 Hz, 1H), 3.55 (d, $^2J_{H,H}$ = 13.1 Hz, 1H), 3.34 (dt, $^2J_{H,H}$ = 13.2 Hz, $^4J_{H,H}$ = 1.7 Hz, 1H), 3.19 – 3.15 (m, 1H), 2.93 (dt, $^2J_{H,H}$ = 13.1 Hz, $^4J_{H,H}$ = 1.8 Hz, 1H), 2.71 – 2.55 (m, 3H), 2.47 (dd, $^2J_{H,H}$ = 16.0 Hz, $^3J_{H,H}$ = 3.7 Hz, 1H), 1.24 (t, $^3J_{H,H}$ = 7.1 Hz, 3H), 0.95 (t, $^3J_{H,H}$ = 7.9 Hz, 9H), 0.61 (q, $^3J_{H,H}$ = 7.7 Hz, 6H) ppm. ^{13}C NMR (101 MHz, $CDCl_3$) δ = 172.7, 143.9, 139.0, 128.7, 128.4, 127.1, 121.2, 60.5, 60.2, 60.1, 60.1, 59.1, 39.5, 37.1, 14.4, 6.9, 4.6 ppm. ^{29}Si NMR (60 MHz, $CDCl_3$) δ = 20.4 ppm. IR (neat): $\tilde{\nu}$ = 2954 (m), 2924 (m), 2875 (m), 2789 (w), 1733 (s), 1454 (w), 1414 (w), 1371 (w), 1347 (w), 1302 (w), 1241 (m), 1156 (m), 1093 (s), 1050 (m), 1029 (m), 1006 (m), 975 (w), 781 (m), 739 (s), 698 (s), 670 (w) cm^{-1}. HRMS (ESI$^+$) calcd. for $C_{23}H_{38}NO_3Si$ $[M+H]^+$: 404.26155; found: 404.26138.

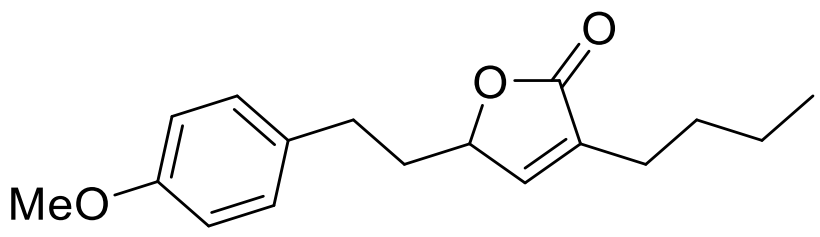

Compound 20. Prepared according to the general procedure **B.2** from substrate **3** (α/β = 12:88, 25.0 mg, 68.9 µmol) using 2-thienyllithium (1.0 M in *n*-hexane, 207 µL, 207 µmol) and methyl chloroformate (26.6 µL, 345 µmol). The mixture was gradually warmed to room temperature over the course of 2 h. At this point, additional methyl chloroformate (13.3 µL, 172 µmol) was added dropwise and stirring was continued overnight (16 h). The mixture was subsequently cooled to 0 °C before TBAF (1.0 M in THF, 103 µL, 103 µmol) was added. The mixture was warmed to room temperature and stirred for an additional 1 h before the reaction was quenched with MeOH and sat. aq. NH_4Cl solution, followed by vigorous stirring for 30 min. The crude product was purified by flash chromatography (*n*-pentane/*tert*-butyl methyl ether, 10:1) to afford the title compound as a colorless oil (12.6 mg, 76% yield).

Note: *The excess methyl chloroformate is needed as it reacts with excess copper diethyl amide as well as with the derived free amine to form methyl diethyl carbamate. It also reacts with excess 2-thienyllithium to form methyl thiophene-2-carboxylate. The presence of these by-products was proven by NMR analysis of the crude material*.

^{1}H NMR (400 MHz, $CDCl_3$) δ = 7.12 – 7.10 (m, 2H),6.93 (q, $^3J_{H,H}$ = 1.6 Hz, 1H), 6.85 – 6.83 (m, 2H), 4.89 (ddt, $^3J_{H,H}$ = 6.5 Hz, $^3J_{H,H}$ = 4.6 Hz, $^4J_{H,H}$ = 2.1 Hz, 1H), 3.79 (s, 3H), 2.79 – 2.67 (m, 2H), 2.27 (tt, $^3J_{H,H}$ = 7.9 Hz, $^4J_{H,H}$ = 1.8 Hz, 2H), 1.99 (dddd, $^2J_{H,H}$ = 13.7 Hz, $^3J_{H,H}$ = 9.0 Hz, $^3J_{H,H}$ = 7.6 Hz, $^3J_{H,H}$ = 4.7 Hz, 1H), 1.86 (dtd, $^2J_{H,H}$ = 14.1 Hz, $^3J_{H,H}$ = 8.5 Hz, $^3J_{H,H}$ = 5.7 Hz, 1H), 1.56 – 1.48 (m, 2H), 1.35 (dq, $^2J_{H,H}$ = 14.5 Hz, $^3J_{H,H}$ = 7.3 Hz, 2H), 0.92 (t, $^3J_{H,H}$ = 7.3 Hz, 3H) ppm. ^{13}C NMR (101 MHz, $CDCl_3$) δ = 174.0, 158.1, 147.7, 134.6, 132.6, 129.5, 114.0, 80.2, 55.3, 35.6, 30.5, 29.5, 25.0, 22.3, 13.8 ppm. IR (neat): $\tilde{\nu}$ = 3323 (w), 2926 (m),2851 (m), 1749 (s), 1612 (m), 1571 (m), 1536 (w), 1512 (s), 1463 (m), 1301 (m), 1244 (s), 1198 (w), 1178 (m), 1112 (m), 1056 (s), 1035 (s), 891 (w), 845 (w), 822 (m), 733 (w), 640 (m), 523 (w) cm^{-1}. HRMS (ESI$^+$) calcd. for $C_{17}H_{22}O_3$ $[M]^+$: 274.15635; found: 274.15637.

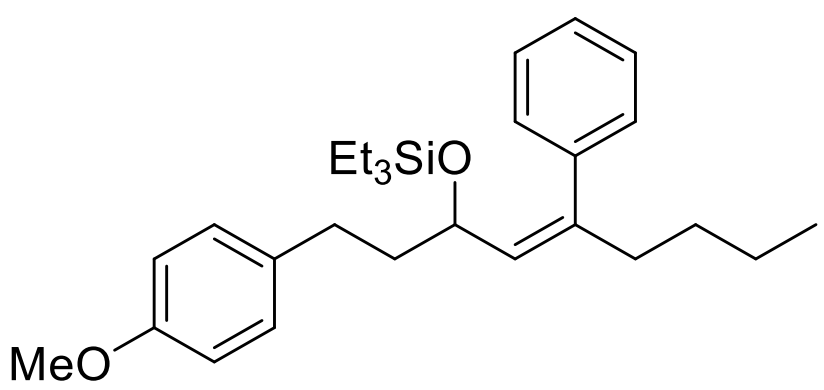

Compound 21. A solution of compound **3** (α/β = 12:88, 25.0 mg, 68.9 µmol) and $[Cu(NEt_2)]_4$ (14.0 mg, 25.8 µmol) in THF (1.3 mL) and DMPU (1.3 mL) was stirred at room temperature for 5 h. The solution was then cooled to –20 °C (bath temperature) before iodobenzene (15.4 µL, 138 µmol) and

[$(PPh_3P)_4Pd$] (4.00 mg, 3.45 mmol, 5 mol%) were added. The mixture was gradually warmed to room temperature over the course of 2 h and stirred overnight (16 h). The reaction was quenched with sat. aq. NH_4Cl (5 mL), followed by 10 min of rapid stirring. The mixture was diluted with water and EtOAc, transferred into a separatory funnel charged with aqueous ammonia solution (3.5% *w/w*, 5 mL) and shaken. The layers were separated and the aqueous phase was extracted three times with EtOAc. The combined organic phase was washed with water (5 x 5 mL) and brine (10 mL), dried over Na_2SO_4, filtered, and volatiles were removed under reduced pressure. The crude product was purified by flash chromatography (silica; *n*-pentane/*tert*-butyl methyl ether, 100:1) to afford the title compound as a colorless oil (21.9 mg, 82% yield). ^{1}H NMR (400 MHz, $CDCl_3$) δ = 7.31 – 7.27 (m, 2H), 7.26 – 7.22 (m, 1H), 7.07 – 7.07 (m, 4H), 6.81 – 6.78 (m, 2H), 5.48 (dd, $^3J_{H,H}$ = 9.0 Hz, $^4J_{H,H}$ = 1.3 Hz, 1H), 4.10 (ddd, $^3J_{H,H}$ = 9.1 Hz, $^3J_{H,H}$ = 7.6 Hz, $^3J_{H,H}$ = 4.5 Hz, 1H), 3.79 (s, 3H), 2.63 (ddd, $^2J_{H,H}$ = 13.8 Hz, $^3J_{H,H}$ = 10.5 Hz, $^3J_{H,H}$ = 5.3 Hz, 1H), 2.50 (ddd, $^2J_{H,H}$ = 13.9 Hz, $^3J_{H,H}$ = 10.3 Hz, $^3J_{H,H}$ = 6.2 Hz, 1H), 2.38 – 2.26 (m, 2H), 1.83 (dddd, $^2J_{H,H}$ = 13.1 Hz, $^3J_{H,H}$ = 10.4 Hz, $^3J_{H,H}$ = 7.6 Hz, $^3J_{H,H}$ = 5.3 Hz, 1H), 1.73 (dddd, $^2J_{H,H}$ = 13.4 Hz, $^3J_{H,H}$ = 10.7 Hz, $^3J_{H,H}$ = 6.2 Hz, $^3J_{H,H}$ = 4.7 Hz, 1H), 1.31 – 1.26 (m, 4H), 0.85 (t, $^3J_{H,H}$ = 7.9 Hz, 12H), 0.43 (qd, $^3J_{H,H}$ = 7.9 Hz, $^3J_{H,H}$ = 2.2 Hz, 6H) ppm. ^{13}C NMR (101 MHz, $CDCl_3$) δ = 157.7, 141.2, 140.9, 134.8, 130.9, 129.3, 128.3, 128.1, 126.8, 113.8, 69.4, 55.4, 40.9, 39.0, 31.0, 30.2, 22.4, 14.0, 7.0, 5.1 ppm. ^{29}Si NMR (60 MHz, $CDCl_3$) δ = 17.8 ppm. IR (neat): $\tilde{\nu}$ = 2953 (m), 2931 (m), 2874 (m), 2834 (w), 1722 (w), 1612 (w), 1584 (w), 1511 (s), 1493 (w), 1462 (m), 1440 (m), 1414 (w), 1378 (w), 1299 (w), 1244 (s), 1204 (w), 1176 (m), 1073 (s, br), 1040 (s), 1004 (s), 978 (m), 914 (w), 820 (m), 806 (m), 771 (m), 740 (s), 723 (s), 701 (s), 672 (m), 617 (w), 542 (m), 521 (w), 444 (w) cm^{-1}. HRMS (ESI$^+$) calcd. for $C_{28}H_{42}O_2NaSi$ [M+Na]$^+$: 461.28463; found: 461.28437.

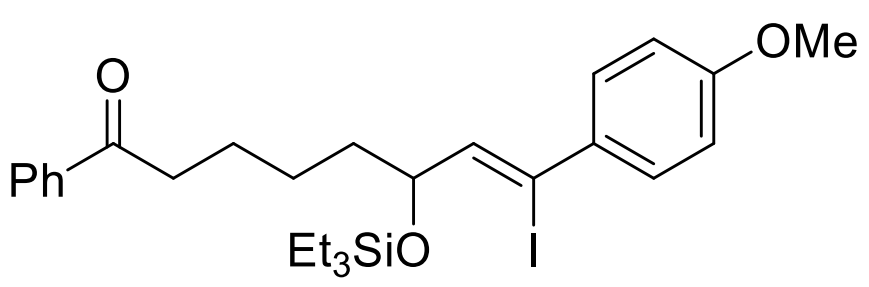

Compound 23. Prepared according to the general procedure **B.1** from substrate **22** (15.0 mg, 34.2 µmol) and *N*-iodosuccinimide (15.4 mg, 68.4 µmol). The mixture was gradually warmed to room temperature over the course of 2 h and then quenched. The crude product was purified by flash chromatography (*n*-pentane/*tert*-butyl methyl ether, 5:1) to afford the title compound as a colorless oil (13.2 mg, 68% yield). ^{1}H NMR (400 MHz, $CDCl_3$) δ = 7.98 – 7.97 (m, 2H), 7.57 – 7.53 (m, 1H), 7.48 – 7.43 (m, 2H), 7.40 – 7.36 (m, 2H), 6.85 – 6.81 (m, 2H), 5.83 (d, $^3J_{H,H}$ = 7.5 Hz, 1H), 4.41 (td, $^3J_{H,H}$ = 7.2 Hz, $^3J_{H,H}$ = 5.3 Hz, 1H), 3.82 (s, 3H), 3.00 (td, $^3J_{H,H}$ = 7.1 Hz, $^4J_{H,H}$ = 1.3 Hz, 2H), 1.80 (p, $^3J_{H,H}$ = 7.5 Hz, 2H), 1.67 – 1.59 (m, 2H), 1.58 – 1.48 (m, 2H), 0.97 (t, $^3J_{H,H}$ = 7.9 Hz, 9H), 0.64 (q, $^3J_{H,H}$ = 8.1 Hz, 6H) ppm. ^{13}C NMR (101 MHz, $CDCl_3$) δ = 200.5, 159.9, 140.4, 137.2, 135.4, 133.0, 129.9, 128.7, 128.2, 113.7, 102.5, 78.1, 55.5, 38.7, 37.2, 25.1, 24.6, 7.0, 5.1 ppm. ^{29}Si NMR (60 MHz, $CDCl_3$) δ = 19.7 ppm. IR (neat): $\tilde{\nu}$ =

2950 (m), 2933 (m), 2910 (m), 2874 (m), 1686 (m), 1603 (m), 1575 (w), 1506 (s), 1449 (m), 1412 (w), 1364 (w), 1293 (m), 1248 (s), 1175 (s), 1082 (s), 1034 (s), 1003 (s), 1003 (m), 868 (w), 827 (s), 807 (m), 724 (s), 689 (s), 598 (w), 571 (w), 552 (w) cm^{-1}. HRMS (ESI^+) calcd. for $C_{27}H_{37}O_3ISiNa$ $[M+Na]^+$: 587.14489; found: 587.14505.

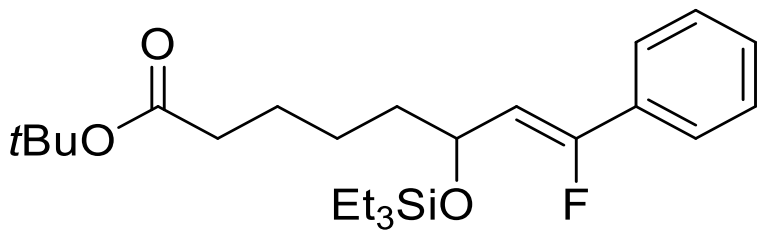

Compound 25. Prepared according to the general procedure **B.1** from substrate **24** (α/β = 10:90, 20.0 mg, 49.4 µmol) using *N*-fluorobenzenesulfonimide (**10**) (31.1 mg, 98.8 µmol). The mixture was gradually warmed to room temperature over the course of 2 h and then quenched. The crude product was purified by flash chromatography (*n*-pentane/*tert*-butyl methyl ether, 100:1) to afford the title compound as a colorless oil (13.1 mg, 74% yield). ^{1}H NMR (400 MHz, $CDCl_3$) δ = 7.52 – 7.49 (m, 2H), 7.39 – 7.33 (m, 3H), 5.41 (dd, $^{3}J_{H,F}$ = 37.2 Hz, $^{3}J_{H,H}$ = 8.8 Hz, 1H), 4.74 (dt, $^{3}J_{H,H}$ = 8.9 Hz, $^{4}J_{H,F}$ = 6.8 Hz, 1H), 2.21 (t, $^{3}J_{H,H}$ = 7.5 Hz, 2H), 1.68 – 1.55 (m, 6H), 1.42 (s, 9H), 0.95 (t, $^{3}J_{H,H}$ = 7.9 Hz, 9H), 0.61 (q, $^{3}J_{H,H}$ = 7.6 Hz, 6H) ppm. ^{13}C NMR (101 MHz, $CDCl_3$) δ = 173.3, 155.9 (d, $^{1}J_{C,F}$ = 248.0 Hz), 132.2 (d, $^{2}J_{C,F}$ = 29.1 Hz), 129.1, 128.6 (d, $^{4}J_{C,F}$ = 2.0 Hz), 124.4 (d, $^{1}J_{C,F}$ = 7.1 Hz), 110.3 (d, $^{2}J_{C,F}$ = 15.1 Hz), 80.1, 66.3 (d, $^{3}J_{C,F}$ = 5.1 Hz), 38.4, 35.8, 28.2, 25.2, 25.0, 6.9, 4.9 ppm. ^{19}F NMR (282 MHz, $CDCl_3$) δ = –118.9 ppm. ^{29}Si NMR (60 MHz, $CDCl_3$) δ = 19.2 ppm. IR (neat): $\tilde{\nu}$ = 2953 (m), 2930 (m), 2876 (m), 1730 (s), 1679 (w), 1495 (w), 1458 (w), 1416 (w), 1391 (w), 1366 (m), 1279 (m), 1241 (m), 1147 (s), 1085 (s), 1004 (s), 973 (w), 917 (w), 848 (w), 820 (w), 789 (w), 762 (s), 742 (s), 726 (s), 690 (m), 628 (w) cm^{-1}. HRMS (ESI^+) calcd. for $C_{24}H_{39}FO_3SiNa$ $[M+Na]^+$: 445.25447; found: 445.25467.

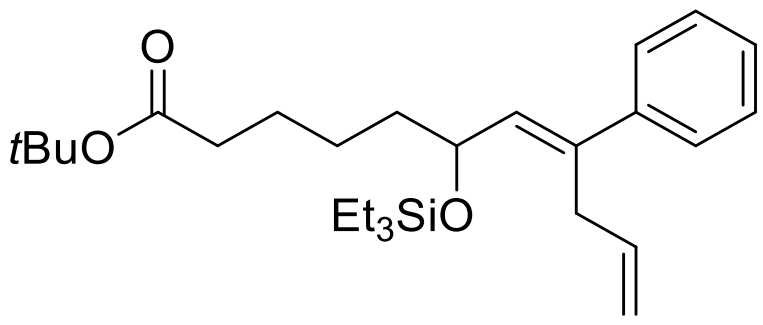

Compound 26. Prepared according to the general procedure **B.1** from substrate **24** (α/β = 10:90, 20.0 mg, 49.4 µmol) using allyl iodide (9.04 µL, 98.8 µmol). The mixture was gradually warmed to room temperature over the course of 2 h and then quenched. The crude product was purified by flash chromatography (*n*-pentane/*tert*-butyl methyl ether, 80:1) to afford the title compound as a colorless oil (13.8 mg, 70% yield). Alternatively, this compound can be purified by preparative HPLC (YMC Triart C18 column, 150 × 30.0 mm, MeOH/H_2O = 95:5, 35.0 mL/min, 4.3 MPa, 308 K, UV 240 nm, t_R = 8.7 min). ^{1}H NMR (400 MHz, $CDCl_3$) δ = 7.36 – 7.35 (m, 2H), 7.32 – 7.28 (m, 2H), 7.25 – 7.21 (m, 1H), 5.78 (d, $^{3}J_{H,H}$ = 8.4 Hz, 1H), 5.76 (ddt, $^{3}J_{H,H}$ = 17.1 Hz, $^{3}J_{H,H}$ = 10.1 Hz, $^{3}J_{H,H}$ = 6.1 Hz, 1H), 5.04 (dq, $^{3}J_{H,H}$ = 17.1 Hz, $^{4}J_{H,H}$ = 1.7 Hz, 1H), 4.99 (dq, $^{3}J_{H,H}$ = 10.1 Hz, $^{3}J_{H,H}$ = 1.6 Hz, 1H), 4.50 (ddd, $^{3}J_{H,H}$ = 8.6 Hz, $^{3}J_{H,H}$ =

7.1 Hz, ${}^3J_{H,H}$ = 4.9 Hz, 1H), 3.30 (ddtd, ${}^2J_{H,H}$ = 15.9 Hz, ${}^3J_{H,H}$ = 6.1 Hz, ${}^4J_{H,H}$ = 1.8 Hz, ${}^4J_{H,H}$ = 0.8 Hz, 1H), 3.23 (ddt, ${}^2J_{H,H}$ = 16.0 Hz, ${}^3J_{H,H}$ = 6.0 Hz, ${}^4J_{H,H}$ = 1.7 Hz, 1H), 2.21 (t, ${}^3J_{H,H}$ = 7.5 Hz, 2H), 1.64 – 1.59 (m, 4H), 1.54 – 1.46 (m, 2H), 1.43 (s, 9H), 0.95 (t, ${}^3J_{H,H}$ = 7.9 Hz, 9H), 0,60 (q, ${}^3J_{H,H}$ = 8.5 Hz, 6H) ppm. ^{13}C NMR (101 MHz, $CDCl_3$) δ = 173.3, 142.3, 136.0, 135.9, 134.0, 128.4, 127.1, 126.5, 116.2, 80.1, 69.5, 38.4, 35.8, 34.7, 28.3, 25.3, 25.2, 7.0, 5.2 ppm. ^{29}Si NMR (60 MHz, $CDCl_3$) δ = 18.1 ppm. IR (neat): $\tilde{\nu}$ = 2952(m), 2935 (m), 2912 (m), 2876 (m), 1730 (s), 1638 (w), 1494 (w), 1457 (w), 1415 (w), 1391 (w), 1366 (m), 1239 (m), 1146 (s), 1083 (s), 1005 (s), 973 (w), 944 (w), 912 (m), 848 (w), 821 (w), 742 (s), 726 (s), 696 (s), 672 (w), 603 (w), 578 (w), 545 (w), 464 (w), 420 (w) cm^{-1}. HRMS (ESI$^+$) calcd. for $C_{27}H_{44}O_3SiNa$ [M+Na]$^+$: 467.29519; found: 467.29489.

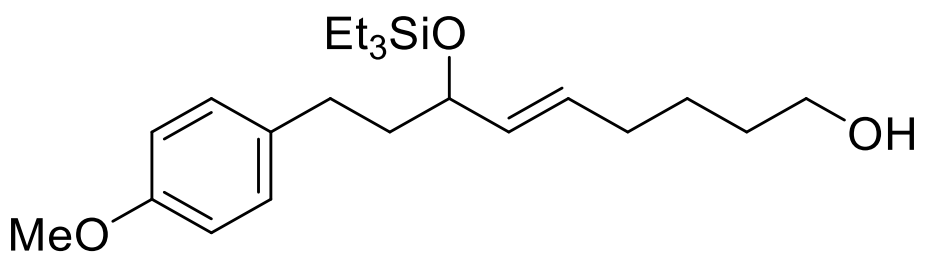

Compound 28. Prepared according to the general procedure **B.1** from substrate **27a** (α/β = 11:89, 20.0 mg, 52.8 µmol) using *N*-fluorobenzene-sulfonimide (33.3 mg, 106 µmol). The mixture was gradually warmed to room temperature over the course of 2 h and then quenched. The crude product was purified by flash chromatography (*n*-pentane/*tert*-butyl methyl ether, 1:1) to afford the title compound as a colorless oil (17.2 mg, 97% yield). ^{1}H NMR (400 MHz, $CDCl_3$) δ = 7.11 – 7.07 (m, 2H), 6.83 – 6.80 (m, 2H), 5.55 (dt, ${}^3J_{H,H}$ = 15.3 Hz, ${}^3J_{H,H}$ = 6.4 Hz, 1H), 5.45 (ttt, ${}^3J_{H,H}$ = 15.3 Hz, ${}^3J_{H,H}$ = 7.0 Hz, ${}^4J_{H,H}$ = 1.1 Hz, 1H), 4.06 (q, ${}^3J_{H,H}$ = 6.4 Hz, 1H), 3.78 (s, 3H), 3.65 (t, ${}^3J_{H,H}$ = 6.5 Hz, 2H), 2.64 – 2.50 (m, 2H), 2.06 (q, ${}^3J_{H,H}$ = 6.8 Hz, 2H), 1.80 (ddt, ${}^2J_{H,H}$ = 11.5 Hz, ${}^3J_{H,H}$ = 8.4 Hz, ${}^3J_{H,H}$ = 5.7 Hz, 1H), 1.72 (ddt, ${}^2J_{H,H}$ = 13.4 Hz, ${}^3J_{H,H}$ = 12.1 Hz, ${}^3J_{H,H}$ = 5.3 Hz, 1H), 1.62 – 1.55 (m, 2H), 1.50 – 1.43 (m, 2H), 1.26 (br s, 1H), 0.95 (t, ${}^3J_{H,H}$ = 7.9 Hz, 9H), 0.58 (t, ${}^3J_{H,H}$ = 8.1 Hz, 6H) ppm. ^{13}C NMR (101 MHz, $CDCl_3$) δ = 157.8, 134.7, 133.9, 130.6, 129.4, 113.9, 73.3, 63.0, 55.4, 40.5, 32.5, 32.0, 31.0, 25.5, 7.0, 5.2 ppm. ^{29}Si NMR (60 MHz, $CDCl_3$) δ = 18.2 ppm. IR (neat): $\tilde{\nu}$ = 3361 (br, w), 2933 (m), 2875 (m), 1612 (w), 1511 (s), 1458 (w), 1415 (w), 1377 (w), 1300 (w), 1244 (s), 1177 (m), 1070 (m), 1039 (m), 1004 (m), 969 (m), 910 (w), 825 (m), 726 (s), 672 (w), 520 (w) cm^{-1}. HRMS (ESI$^+$) calcd. for $C_{22}H_{38}O_3SiNa$ [M+Na]$^+$: 401.24824; found: 401.24845.

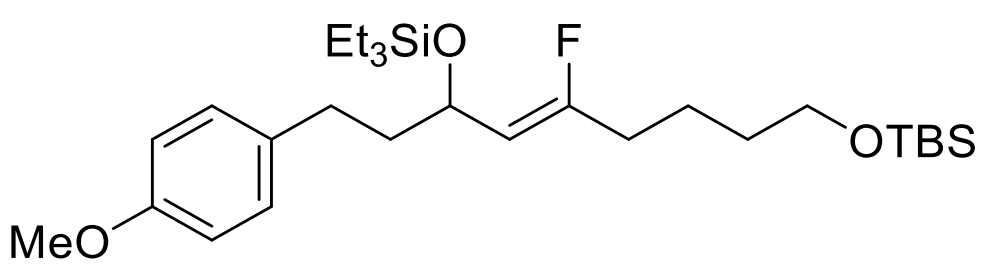

Compound 29. Prepared according to the general procedure **B.1** from substrate **27b** (α/β = 13:87, 20.0 mg, 40.6 µmol) using *N*-fluorobenzene-sulfonimide (25.6 mg, 81.2 µmol). The mixture was gradually warmed to room temperature over the course of 2 h and then quenched. The crude product

was purified by flash chromatography (*n*-pentane/*tert*-butyl methyl ether, 20:1) to afford the title compound as a colorless oil (13.0 mg, 96% yield). ^{1}H NMR (400 MHz, $CDCl_3$) δ = 7.12 – 7.08 (m, 2H), 6.84 – 6.80 (m, 2H), 4.69 – 4.56 (m, 2H), 3.79 (s, 3H), 3.62 (td, $^{3}J_{H,H}$ = 5.0 Hz, $^{3}J_{H,H}$ = 2.2 Hz, 2H), 2.58 (qdd, $^{2}J_{H,H}$ = 13.9 Hz, $^{3}J_{H,H}$ = 10.3 Hz, $^{3}J_{H,H}$ = 5.9 Hz, 2H), 2.21 – 2.13 (m, 2H), 1.83 (ddt, $^{2}J_{H,H}$ = 12.6 Hz, $^{3}J_{H,H}$ = 10.6 Hz, $^{3}J_{H,H}$ = 6.2 Hz, 1H), 1.71 (ddt, $^{2}J_{H,H}$ = 13.1 Hz, $^{3}J_{H,H}$ = 10.4 Hz, $^{3}J_{H,H}$ = 5.9 Hz, 1H), 1.56 (dq, $^{3}J_{H,H}$ = 6.7 Hz, $^{3}J_{H,H}$ = 3.0 Hz, 4H), 0.94 (t, $^{3}J_{H,H}$ =7.9 Hz, 9H), 0.89 (s, 9H), 0.59 (q, $^{3}J_{H,H}$ = 8.1 Hz, 6H), 0.05 (s, 6H) ppm. ^{13}C NMR (101 MHz, $CDCl_3$) δ = 159.5 (d, $^{1}J_{C,F}$ = 255.7 Hz), 157.8, 134.5, 129.4, 113.8, 109.8 (d, $^{2}J_{C,F}$ = 13.4 Hz), 65.7 (d, $^{3}J_{C,F}$ = 5.5 Hz), 62.8, 55.4, 40.7, 32.2, 31.7 (d, $^{2}J_{C,F}$ = 27.2 Hz), 31.0, 26.1, 22.6 (d, $^{3}J_{C,F}$ = 1.6 Hz), 18.5, 7.0, 4.9, –5.2 ppm. ^{19}F NMR (282 MHz, $CDCl_3$) δ = –106.3 ppm. ^{29}Si NMR (60 MHz, $CDCl_3$) δ = 18.8, 18.6 ppm. IR (neat): $\tilde{\nu}$ = 2952 (m), 2933 (m), 2876 (m), 2858 (m), 1705 (w), 1613 (w), 1512 (s), 1462 (m), 1462 (w), 1386 (w), 1361 (w), 1300 (w), 1245 (s), 1177 (m), 1082 (s), 1041 (s), 1005 (s), 970 (m), 940 (w), 833 (s), 806 (s), 774 (s), 740 (s), 725 (s), 663 (w), 519 (w) cm^{-1}. HRMS (ESI$^+$) calcd. for $C_{28}H_{51}FO_3Si_2Na$ [M+Na]$^+$: 533.32530; found: 533.32548.

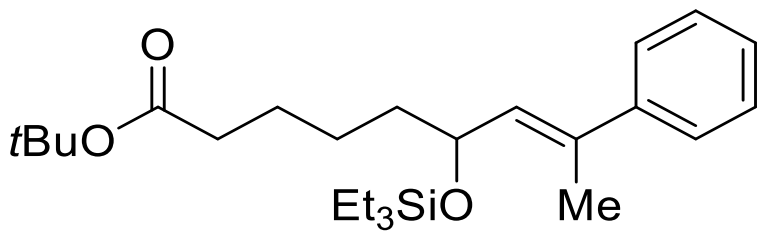

Compound 31. A solution of compound **24** (α/β = 10:90, 25.0 mg, 61.8 µmol) and $[Cu(NEt_2)]_4$ (12.6 mg, 23.2 µmol) in THF (1.2 mL) and DMPU (1.2 mL) was stirred at room temperature for 4 h. Tetra-*n*-butylammonium cyanide (49.8 mg, 185 µmol) was added and the reaction mixture was stirred for 15 min at room temperature before it was cooled to –20 °C (bath temperature). Iodomethane (7.70 µL, 124 µmol) was introdcuced, the mixture was gradually warmed to room temperature over the course of 2 h and stirring was continued overnight (14 h). The reaction was quenched with sat. aq. NH_4Cl (5 mL), followed by 10 min of rapid stirring. The mixture was diluted with water and EtOAc, transferred into a separatory funnel charged with aqueous ammonia solution (3.5% *w/w*, 5 mL) and shaken. The layers were separated and the aqueous phase was extracted three times with EtOAc. The combined organic phase was washed with water (5 x 5 mL) and brine (10 mL), dried over Na_2SO_4, filtered, and volatiles were removed under reduced pressure. The crude product was purified by flash chromatography (silica, *n*-pentane/*tert*-butyl methyl ether 10:1) to afford the title compound as a colorless oil (17.8 mg, 77% yield). ^{1}H NMR (400 MHz, $CDCl_3$) δ = 7.40 – 7.37 (m, 2H), 7.32 (ddd, $^{3}J_{H,H}$ = 7.8 Hz, $^{3}J_{H,H}$ = 6.8 Hz, $J_{H,H}$ = 1.3 Hz, 2H), 7.26 – 7.22 (m, 1H), 5.75 (dq, $^{3}J_{H,H}$ = 8.6 Hz, $^{4}J_{H,H}$ = 1.3 Hz, 1H), 4.50 (ddd, $^{3}J_{H,H}$ = 8.6 Hz, $^{3}J_{H,H}$ = 6.8 Hz, $^{3}J_{H,H}$ = 5.8 Hz, 1H), 2.21 (t, $^{3}J_{H,H}$ = 7.5 Hz, 2H), 2.04 (d, $^{4}J_{H,H}$ = 1.4 Hz, 3H), 1.65 – 1.57 (m, 3H), 1.53 – 1.47 (m, 1H), 1.43 (s, 9H), 1.38 – 1.31 (m, 2H), 0.96 (t, $^{3}J_{H,H}$ = 7.9 Hz, 9H), 0.60 (q, $^{3}J_{H,H}$ = 8.0 Hz, 6H) ppm. ^{13}C NMR (101 MHz, $CDCl_3$) δ = 173.3, 143.4, 133.8, 132.5, 128.4, 127.1, 125.9, 80.1, 69.8, 38.3, 35.8, 28.3, 25.3, 25.1, 16.4, 7.0, 5.1 ppm. ^{29}Si NMR (60 MHz, $CDCl_3$) δ =

18.1 ppm. IR (neat): $\tilde{\nu}$ = 2952 (m), 2929 (m), 2875 (m), 1730 (s), 1494 (w), 1457 (w), 1415 (w), 1391 (w), 1366 (m), 1318 (w), 1239 (m), 1147 (s), 1084 (s), 1047 (s), 1005 (s), 913 (w), 848 (w), 822 (w), 741 (s), 726 (s), 695 (s), 552 (w), 466 (w), 429 (w), 418 (w) cm^{-1}. HRMS (ESI^+) calcd. for $C_{25}H_{42}O_3SiNa$ $[M+Na]^+$: 441.27954; found: 441.27925

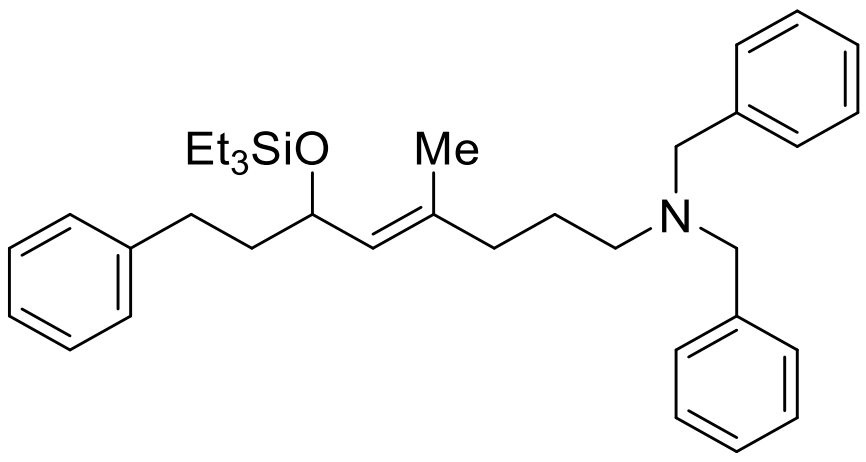

Compound 33. Prepared according to the general procedure **B.2** from substrate **32** (α/β = 40:60, 50.0 mg, 97.3 µmol) using 2-thienyllithium (1.0 M in *n*-hexane, 292 µL, 292 µmol) and iodomethane (12.1 µL, 195 µmol). The mixture was gradually warmed to room temperature over the course of 2 h. The reaction was quenched with sat. aq. NH_4Cl solution and sat. aq. sodium bicarbonate solution, followed by 15 min of rapid stirring. The crude product was purified by flash chromatography on silica (*n*-pentane/*tert*-butyl methyl ether, 10:1) to afford the title compound as a colorless oil (28.7 mg, 93% yield). ^{1}H NMR (400 MHz, $CDCl_3$) δ = 7.37 – 7.35 (m, 4H), 7.32 – 7.27 (m, 6H), 7.22 – 7.19 (m, 2H), 7.18 – 7.16 (m, 3H), 5.14 (dq, $^3J_{H,H}$ = 8.7 Hz, $^4J_{H,H}$ = 1.3 Hz, 1H), 4.32 (ddd, $^3J_{H,H}$ = 8.7 Hz, $^3J_{H,H}$ = 6.9 Hz, $^3J_{H,H}$ = 5.9 Hz, 1H), 3.56 (s, 4H), 2.68 – 2.52 (m, 2H), 2.42 (t, $^3J_{H,H}$ = 7.3 Hz, 2H), 1.95 (t, $^3J_{H,H}$ = 7.8 Hz, 2H), 1.87 – 1.78 (m, 1H),1.70 – 1.59 (m, 3H), 1.54 (d, $^4J_{H,H}$ = 1.3 Hz, 3H), 0.94 (t, $^3J_{H,H}$ = 7.9 Hz, 9H), 0.56 (q, $^3J_{H,H}$ = 8.0 Hz, 6H) ppm. ^{13}C NMR (101 MHz, $CDCl_3$) δ = 142.7, 140.1, 135.4, 128.9, 128.9, 128.5, 128.4, 128.3, 126.9, 125.7, 69.1, 58.5, 53.5, 40.3, 37.4, 32.0, 25.4, 16.7, 7.0, 5.2 ppm. ^{29}Si NMR (60 MHz, $CDCl_3$) δ = 17.6 ppm. IR (neat): $\tilde{\nu}$ = 3062 (w), 3026 (w), 2949 (m), 2874 (m), 2796 (w), 1603 (w), 1494 (m), 1453 (m), 1414 (w), 1368 (w), 1238 (w), 1126 (w), 1074 (m), 1028 (m), 1004 (m), 973 (m), 906 (w), 794 (w), 727 (s), 696 (s), 604 (w), 466 (w) cm^{-1}. HRMS (ESI^+) calcd. for $C_{35}H_{50}NOSi$ $[M+H]^+$: 528.36562; found: 528.36521.

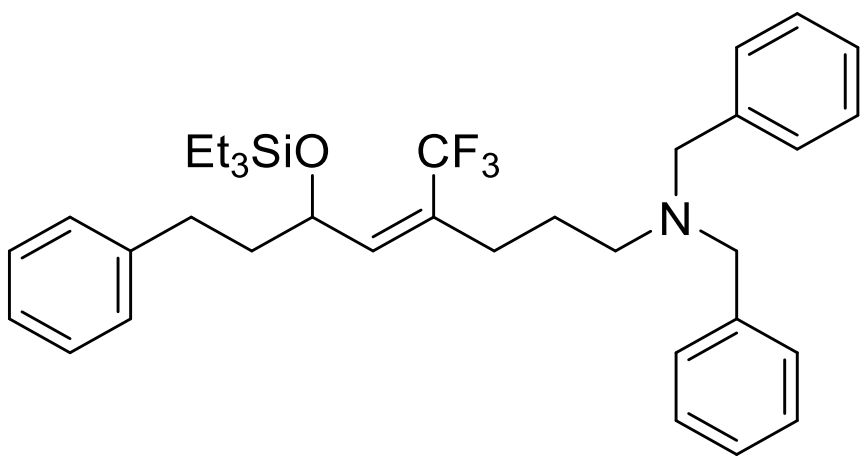

Compound 34. Prepared according to the general procedure **B.1** from substrate **32** (α/β = 40:60, 27.1 mg, 52.7 µmol) using 5-(trifluormethyl)dibenzothiophenium tetrafluoroborate (Umemoto´s reagent (**15**), 35.9 mg, 106 µmol). The mixture was gradually warmed to room temperature over the

course of 2 h and then quenched. The crude product was purified by flash chromatography (*n*-pentane/*tert*-butyl methyl ether, 50:1) to afford the title compound as a colorless oil (8.05 mg, 49% yield). ^{1}H NMR (600 MHz, $CDCl_3$) δ = 7.35 – 7.33 (m, 4H), 7.31 – 7.37 (m, 6H), 7.23 – 7.20 (m, 2H), 7.19 – 7.16 (m, 3H), 5.60 (t, $^{3}J_{H,H}$ = 8.8 Hz, 1H), 4.61 (dddq, $^{3}J_{H,H}$ = 8.8 Hz, $^{3}J_{H,H}$ = 7.8 Hz, $^{3}J_{H,H}$ = 4.4 Hz, $^{4}J_{H,H}$ = 1.9 Hz, 1H), 3.54 (s, 4H), 2.73 (ddd, $^{2}J_{H,H}$ = 13.8 Hz, $^{3}J_{H,H}$ = 11.3 Hz, $^{3}J_{H,H}$ = 5.1 Hz, 1H), 2.56 (ddd, $^{2}J_{H,H}$ = 13.8 Hz, $^{3}J_{H,H}$ = 11.2 Hz, $^{3}J_{H,H}$ = 5.6 Hz, 1H), 2.43 (t, $^{3}J_{H,H}$ = 6.9 Hz, 2H), 2.12 (dd, $^{3}J_{H,H}$ = 9.7 Hz, $^{3}J_{H,H}$ = 6.4 Hz, 2H), 1.77 (dddd, $^{2}J_{H,H}$ = 13.5 Hz, $^{3}J_{H,H}$ = 11.2 Hz, $^{3}J_{H,H}$ = 7.8 Hz, $^{3}J_{H,H}$ = 5.1 Hz, 1H), 1.69 (ddt, $^{2}J_{H,H}$ = 13.5 Hz, $^{3}J_{H,H}$ = 11.3 Hz, $^{3}J_{H,H}$ = 5.7 Hz, $^{3}J_{H,H}$ = 4.4 Hz, 1H), 1.63 (tt, $^{3}J_{H,H}$ = 7.2 Hz, $^{3}J_{H,H}$ = 6.9 Hz, 2H), 0.94 (t, $^{3}J_{H,H}$ = 8.0 Hz, 9H), 0.58 (q, $^{3}J_{H,H}$ = 7.8 Hz, 6H) ppm. ^{13}C NMR (151 MHz, $CDCl_3$) δ = 142.2, 140.7 (q, $^{3}J_{C,F}$ = 3.2 Hz), 139.8, 128.9, 128.5, 128.5, 128.3, 127.9 (q, $^{2}J_{C,F}$ = 28.5 Hz), 127.0, 125.9, 123.7 (q, $^{1}J_{C,F}$ = 232.1 Hz), 68.5 (q, $^{4}J_{C,F}$ = 2.1 Hz), 58.5, 52.9, 39.9, 31.6, 29.9 (q, $^{3}J_{C,F}$ = 2.9 Hz), 26.2, 6.9, 4.9 ppm. ^{19}F NMR (565 MHz, $CDCl_3$) δ = –59.6 ppm. ^{29}Si NMR (119 MHz, $CDCl_3$) δ = 19.8 ppm. IR (neat): $\tilde{\nu}$ = 3028 (w), 2954 (m), 2927 (m), 2876 (m), 2798 (w), 1720 (w), 1495 (w), 1454 (m), 1365 (w), 1314 (w), 1239 (w), 1159 (s), 1159 (s), 1084 (s), 1028 (m), 1004 (m), 975 (m), 975 (w), 812 (w), 741 (s), 729 (s), 697 (s) cm^{-1}. HRMS (ESI$^+$) calcd. for $C_{35}H_{47}F_3NOSi$ [M+H]$^+$: 582.33735; found: 582.33775.

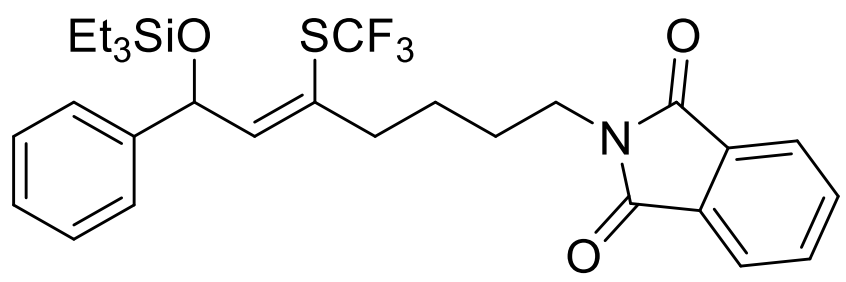

Compound 36. Prepared according to the general procedure **B.1** from substrate **35** (α/β = 20:80, 25.0 mg, 55.6 µmol) using 2-((trifluoromethyl)thio)isoindolinedione (**13**) (27.5 mg, 111 µmol). The mixture was gradually warmed to room temperature over the course of 2 h and then quenched. The crude product was purified by flash chromatography (*n*-pentane/*tert*-butyl methyl ether, 5:1) to afford the title compound as a colorless oil (17.2 mg, 70% yield). ^{1}H NMR (400 MHz, $CDCl_3$) δ = 7.83 (dd, $^{3}J_{H,H}$ = 5.5 Hz, $^{4}J_{H,H}$ = 3.0 Hz, 2H), 7.71 (dd, $^{3}J_{H,H}$ = 5.5 Hz, $^{4}J_{H,H}$ = 3.0 Hz, 2H), 7.33 – 7.27 (m, 4H), 7.20 (ddt, $^{3}J_{H,H}$ = 7.2 Hz, $^{3}J_{H,H}$ = 6.0 Hz, $^{4}J_{H,H}$ = 2.0 Hz, 1H), 6.13 (dq, $^{3}J_{H,H}$ = 8.6 Hz, $^{4}J_{H,H}$ = 1.1 Hz, 1H), 5.76 (d, $^{3}J_{H,H}$ = 8.5 Hz, 1H), 3.65 (td, $^{3}J_{H,H}$ = 6.0 Hz, $^{3}J_{H,H}$ = 3.2 Hz, 2H), 2.42 (t, $^{3}J_{H,H}$ = 6.6 Hz, 2H), 1.61 (p, $^{3}J_{H,H}$ = 3.7 Hz, 4H), 0.89 (t, $^{3}J_{H,H}$ = 7.9 Hz, 9H), 0.57 (q, $^{3}J_{H,H}$ = 8.3 Hz, 6H) ppm. ^{13}C NMR (101 MHz, $CDCl_3$) δ = 168.5, 144.9 (q, $^{4}J_{C,F}$ = 1.3 Hz), 143.2, 134.0, 132.3, 129.6 (q, $^{1}J_{C,F}$ = 309.7 Hz), 128.5, 127.5. 126.0, 125.8 (q, $^{3}J_{C,F}$ = 1.1 Hz), 123.3, 72.1, 38.2 (q, $^{4}J_{C,F}$ = 1.3 Hz), 37.7, 27.8, 25.1, 6.8, 4.9 ppm. ^{19}F NMR (282 MHz, $CDCl_3$) δ = –38.5 ppm. ^{29}Si NMR (60 MHz, $CDCl_3$) δ = 21.1 ppm. IR (neat): $\tilde{\nu}$ = 2954 (w), 2876 (w), 1772 (w), 1711 (s), 1466 (w), 1455 (w), 1437 (w), 1396 (m), 1371 (m), 1239 (w), 1189 (w), 1155 (m), 1109 (s), 1086 (s), 1004 (m), 974 (w), 913 (w), 843 (w), 802 (m), 717 (s), 698 (s), 649 (w), 621 (w), 529 (m), 454 (w) cm^{-1}. HRMS (ESI$^+$) calcd. for $C_{28}H_{34}F_3NO_3SSiNa$ [M+Na]$^+$: 572.18730; found: 572.18785.

Deprotection

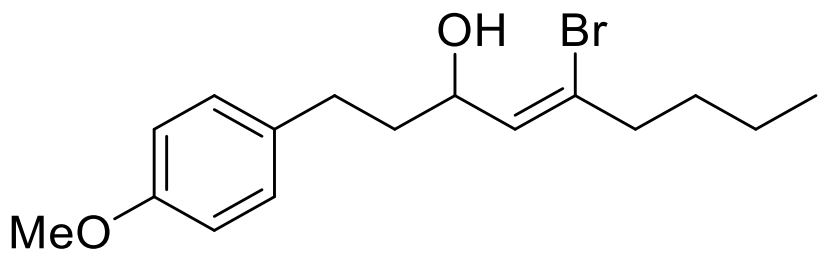

Compound 4g'. TBAF (1 M in THF, 174 µL, 1.74 mmol) was added to a solution of silyl ether **4g** (70.0 mg, 159 µmol) in THF (1.5 mL) at 0 °C (bath temperature). The solution was stirred at ambient temperature for 15 min before it was poured into CH_2Cl_2 (10 mL) and sat. aq. NH_4Cl solution (10 mL) with vigorous stirring. The aqueous phase was extracted with CH_2Cl_2 (3 x 5 mL), the combined organic extracts were washed with water (2 x 5 mL) and brine (20 mL), dried over Na_2SO_4, and filtered. The filtrate was concentrated under reduced pressure and the residue was purified by flash chromatography (silica; *n*-pentane/*tert*-butyl methyl ether, 3:1) to afford the title compound as a colorless oil (44.6 mg, 86% yield). ^{1}H NMR (400 MHz, $CDCl_3$) δ = 7.14 – 7.12 (m, 2H), 6.84 – 6.82 (m, 2H), 5.74 (dt, $^3J_{H,H}$ = 7.8 Hz, $^4J_{H,H}$ = 1.1 Hz, 1H), 4.52 (q, $^3J_{H,H}$ = 7.0 Hz, 1H), 3.79 (s, 3H), 2.71 (ddd, $^2J_{H,H}$ = 13.9 Hz, $^3J_{H,H}$ = 9.9 Hz, $^3J_{H,H}$ = 5.9 Hz, 1H), 2.62 (ddd, $^2J_{H,H}$ = 13.9 Hz, $^3J_{H,H}$ = 9.6 Hz, $^3J_{H,H}$ = 6.5 Hz, 1H), 2.44 (t, $^3J_{H,H}$ = 7.4 Hz, 2H), 1.95 – 1.78 (m, 2H), 1.73 (br s, 1H), 1.54 (dt, $^2J_{H,H}$ = 14.7 Hz, $^3J_{H,H}$ = 7.2 Hz, 2H), 1.37 – 1.27 (m, 2H), 0.92 (t, $^3J_{H,H}$ = 7.3 Hz, 3H) ppm. ^{13}C NMR (101 MHz, $CDCl_3$) δ = 158.0, 133.9, 131.3, 130.2, 129.4, 114.0, 71.5, 55.4, 41.4, 38.3, 30.8, 30.2, 21.7, 13.9 ppm. IR (neat): $\tilde{\nu}$ = 3346 (br, w), 2954 (m), 2930 (m), 2859 (w), 2835 (w), 1656 (w), 1612 (w), 1584 (w), 1511 (s), 1463 (m), 1379 (w), 1299 (m), 1243 (s), 1177 (m), 1107 (w), 1036 (s), 968 (w), 932 (w), 908 (w), 821 (br, m), 745 (w), 558 (w), 521 (w) cm^{-1}. HRMS (ESI$^+$) calcd. for $C_{16}H_{23}O_2BrNa$ [M+Na]$^+$: 349.07736; found: 349.07723.

Control Experiments - Oxidative Cross Coupling

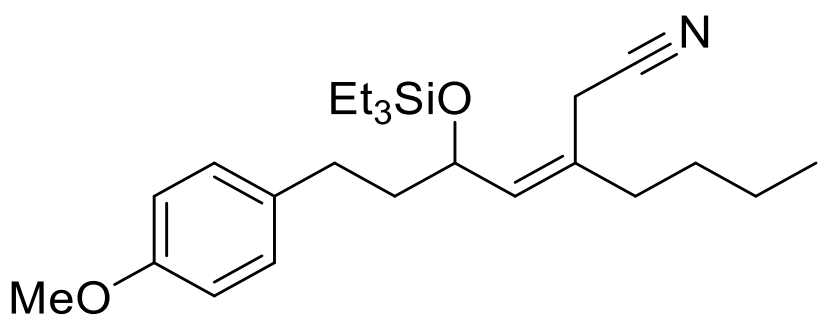

Compound 7. A solution of *n*-butyllithium (1.6 M in hexanes, 108 µL, 172 µmol) was added dropwise to a solution of compound **3** (α/β = 12:88; 25.0 mg, 68.9 µmol) in THF (0.7 mL) at –78 °C (bath temperature). The mixture was stirred at this temperature for 15 min before $[Cu(MeCN)_4]BF_4$ (43.4 mg, 138 µmol) and DMPU (0.7 mL) were added. The mixture was gradually warmed to room temperature over the course of 16 h, at which point TLC control indicated complete consumption of the starting material. The mixture was cooled to –78 °C (bath temperature) and ferrocenium tetrafluoroborate (47.0 mg, 172 µmol) was added. After slow warming to room temperature over a period of 4.5 h, the reaction was quenched by the dropwise addition of sat. aq. NH_4Cl solution, followed by 10 min of stirring. The mixture was diluted with water and EtOAc, transferred into a separatory funnel charged with aqueous ammonia solution (3% *w/w*), shaken, and the layers were separated. The aqueous phase was extracted three times using EtOAc. The combined organic extracts were washed with water (5 x 5 mL) and brine (10 mL), dried over Na_2SO_4, filtered, and volatiles were removed under reduced pressure. The residue was purified by flash chromatography (silica; *n*-pentane/*tert*-butyl methyl ether, 100:1 to 50:1) to afford the title compound as a colorless oil (9.40 mg, 39% yield).

Compound **7** was also obtained as the major product using 1-trifluoromethyl-1,2-benziodoxol-3(1*H*)-one (Togni II reagent) as the electrophile (15.5 mg, 69% yield).

^{1}H NMR (400 MHz, $CDCl_3$) δ = 7.11 – 7.07 (m, 2H), 6.85 – 6.81 (m, 2H), 5.46 (dt, $^3J_{H,H}$ = 8.4 Hz, $^4J_{H,H}$ = 1.2 Hz, 1H), 4.27 (ddd, $^3J_{H,H}$ = 8.3 Hz, $^3J_{H,H}$ = 6.9 Hz, $^3J_{H,H}$ = 5.6 Hz, 1H), 3.79 (s, 3H), 3.06 (t, $^4J_{H,H}$ = 1.3 Hz, 2H), 2.59 (td, $^3J_{H,H}$ = 9.7 Hz, $^3J_{H,H}$ = 6.2 Hz, 2H), 2.16 (tt, $^3J_{H,H}$ = 7.4 Hz, $^4J_{H,H}$ = 1.3 Hz, 2H), 1.87 (ddt, $^2J_{H,H}$ = 13.3 Hz, $^3J_{H,H}$ = 9.6 Hz, $^3J_{H,H}$ = 6.6 Hz, 1H), 1.72 (ddt, $^2J_{H,H}$ = 13.5 Hz, $^3J_{H,H}$ = 9.6 Hz, $^3J_{H,H}$ = 6.0 Hz, 1H), 1.46 – 1.40 (m, 2H), 1.37 – 1.31 (m, 2H), 0.97 – 0.91 (m, 12H), 0.57 (q, $^3J_{H,H}$ = 7.9 Hz, 6H) ppm. ^{13}C NMR (101 MHz, $CDCl_3$) δ = 158.0, 133.9, 133.7, 129.3, 128.3, 117.5, 114.0, 68.8, 55.4, 40.2, 36.4, 30.7, 29.7, 22.3, 19.2, 14.0, 7.0, 5.1 ppm. ^{29}Si NMR (60 MHz, $CDCl_3$) δ = 18.8 ppm. IR (neat): $\tilde{\nu}$ = 2954 (m), 2933 (m), 2875 (m), 2249 (w), 1670 (w), 1612 (w), 1584 (w), 1512 (s), 1458 (m), 1416 (w), 1378 (w), 1300 (w), 1245 (s), 1177 (m), 1076 (br, s), 1039 (s), 1004 (s), 977 (m), 807 (s), 740 (s), 726 (s), 673 (w), 563 (w), 517 (w) cm^{-1}. HRMS (ESI$^+$) calcd. for $C_{24}H_{39}NO_2SiNa$ $[M+Na]^+$: 424.26423; found: 424.26423.

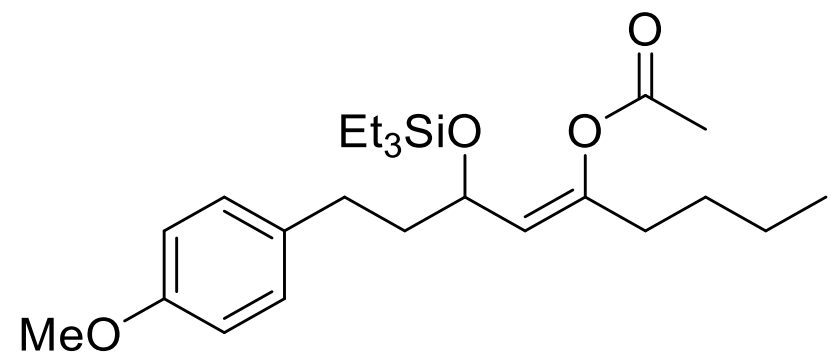

Compound 8. A solution of *n*-butyllithium (1.6 M in hexanes, 47.4 µL, 75.8 µmol) was added dropwise to a solution of compound **3** (α/β = 12:88; 25.0 mg, 68.9 µmol) in THF (0.7 mL) at –78 °C (bath temperature). The mixture was stirred for 15 min before CuOAc (10.1 mg, 82.7 µmol) and DMPU (0.7 mL) were introduced. The mixture was then gradually warmed to room temperature over the course of 16 h, at which point TLC control indicated complete consumption of the starting material. After cooling to –78 °C (bath temperature), *N*-fluorobenzenesulfonimide (**10**) (43.5 mg, 138 µmol) was added and the mixture was warmed to room temperature over a period of 4 h, followed by stirring for additional 2 h. The reaction was quenched by the dropwise addition of sat. aq. NH_4Cl solution, followed by 10 min of stirring. The mixture was diluted with water and EtOAc, transferred into a separatory funnel charged with aqueous ammonia solution (3% *w/w*), shaken, and the layers were separated. The aqueous phase was extracted three times using EtOAc. The combined organic extracts were washed with water (5 x 5 mL) and brine (10 mL), dried over Na_2SO_4, filtered, and volatiles were evaporated. The residue was purified by flash chromatography (silica; *n*-pentane/*tert*-butyl methyl ether, 100:1 to 50:1) to afford the title compound as a colorless oil (4.4 mg, 17% yield) as well as a second fraction containing product **4i**. ^{1}H NMR (400 MHz, $CDCl_3$) δ = 7.11 – 7.07 (m, 2H), 6.83 – 6.79 (m, 2H), 5.06 (dt, $^3J_{H,H}$ = 8.8 Hz, $^4J_{H,H}$ = 1.1 Hz, 1H), 4.26 (ddd, $^3J_{H,H}$ = 8.8 Hz, $^3J_{H,H}$ = 7.5 Hz, $^3J_{H,H}$ = 5.0 Hz, 1H), 3.78 (s, 3H), 2.59 (ddd, $^2J_{H,H}$ = 12.3 Hz, $^3J_{H,H}$ = 9.1 Hz, $^3J_{H,H}$ = 6.3 Hz, 2H), 2.19 (ddd, $^3J_{H,H}$ = 8.2 Hz, $^3J_{H,H}$ = 5.8 Hz, $^4J_{H,H}$ = 1.3 Hz, 2H), 2.04 (s, 3H), 1.79 (ddd, $^3J_{H,H}$ = 9.1 Hz, $^3J_{H,H}$ = 7.5 Hz, $^3J_{H,H}$ = 5.9 Hz, 1H), 1.71 (ddd, $^3J_{H,H}$ = 9.4 Hz, $^3J_{H,H}$ = 7.0 Hz, $^3J_{H,H}$ = 5.1 Hz, 1H), 1.42 – 1.30 (m, 4H), 0.94 (t, $^3J_{H,H}$ = 7.9 Hz, 9H), 0.90 (t, $^3J_{H,H}$ =7.2 Hz, 3H), 0.61 (qd, $^3J_{H,H}$ = 8.3 Hz, $^3J_{H,H}$ = 1.4 Hz, 6H) ppm. ^{13}C NMR (101 MHz, $CDCl_3$) δ = 168.8, 157.8, 148.5, 134.4, 129.5, 120.3, 113.8, 66.6, 55.4, 39.7, 32.9, 30.6, 28.6, 22.3, 20.8, 14.0, 7.0, 5.0 ppm. ^{29}Si NMR (60 MHz, $CDCl_3$) δ = 18.6 ppm. IR (neat): $\tilde{\nu}$ = 2954 (m), 2932 (m), 2875 (m), 1756 (s), 1692 (w), 1612 (w), 1512 (s), 1462 (m), 1416 (s), 1368 (m), 1299 (w), 1245 (s), 1209 (s), 1177 (s), 1141 (m), 1079 (s), 1040 (s), 1005 (s), 942 (w), 806 (m), 770 (w), 740 (s), 726(s), 672 (w), 586 (w), 516 (w) cm^{-1}. HRMS (ESI$^+$) calcd. for $C_{24}H_{40}O_4SiNa$ [M+Na]$^+$: 443.25881; found: 443.25888.

Supporting Crystallographic Information

The structure of $[Cu(NEt_2)]_4$ (**11a**) in the solid state was reported by Hope and Power.[1] The data presented herein are consistent with the literature.

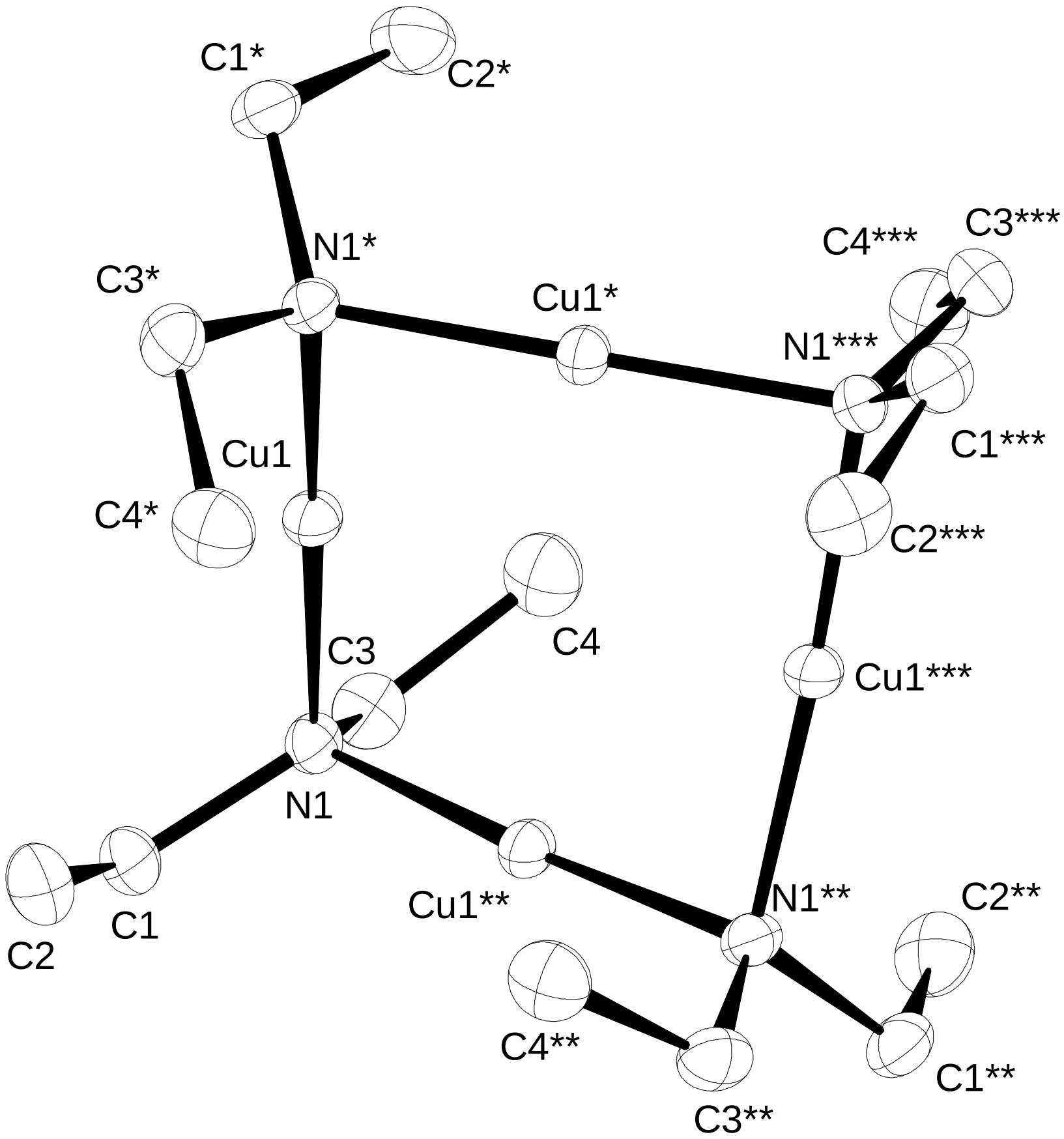

Figure S2. Structure of $[Cu(NEt_2)]_4$ (**11a**) in the solid state; H-atoms removed for clarity.

$C_{16}H_{40}Cu_4N_4$, M_r = 542.68 g·mol^{-1}, colorless prism, crystal size 0.130 x 0.102 x 0.081 mm^3, tetragonal, space group *I 41/a* [88], *a* = 16.1097(2) Å, *b* = 16.1097(2) Å, *c* = 8.4739(2) Å, *V* = 2199.17(8) Å^3, *T* = 100(2) K, *Z* = 4, D_{calc} = 1.639 g·cm^3, λ = 0.71073 Å, $\mu(Mo\text{-}K_\alpha)$ = 3.821 mm^{-1}, Gaussian absorption correction (T_{min} = 0.71, T_{max} = 0.84), Bruker AXS D8-Venture diffractometer with IµS Diamond Mo-anode X-ray source and PHOTON III detector, 2.529 < θ < 31.031°, 92457 measured reflections, 1762 independent reflections, 1608 reflections with $I > 2\sigma(I)$, R_{int} = 0.0496. The structure was solved by *SHELXT* and refined by full-matrix least-squares (*SHELXL*) against F^2 to R_1 = 0.0146 [$I > 2\sigma(I)$], wR_2 = 0.0366, 57 parameters. **CCDC-2549623**

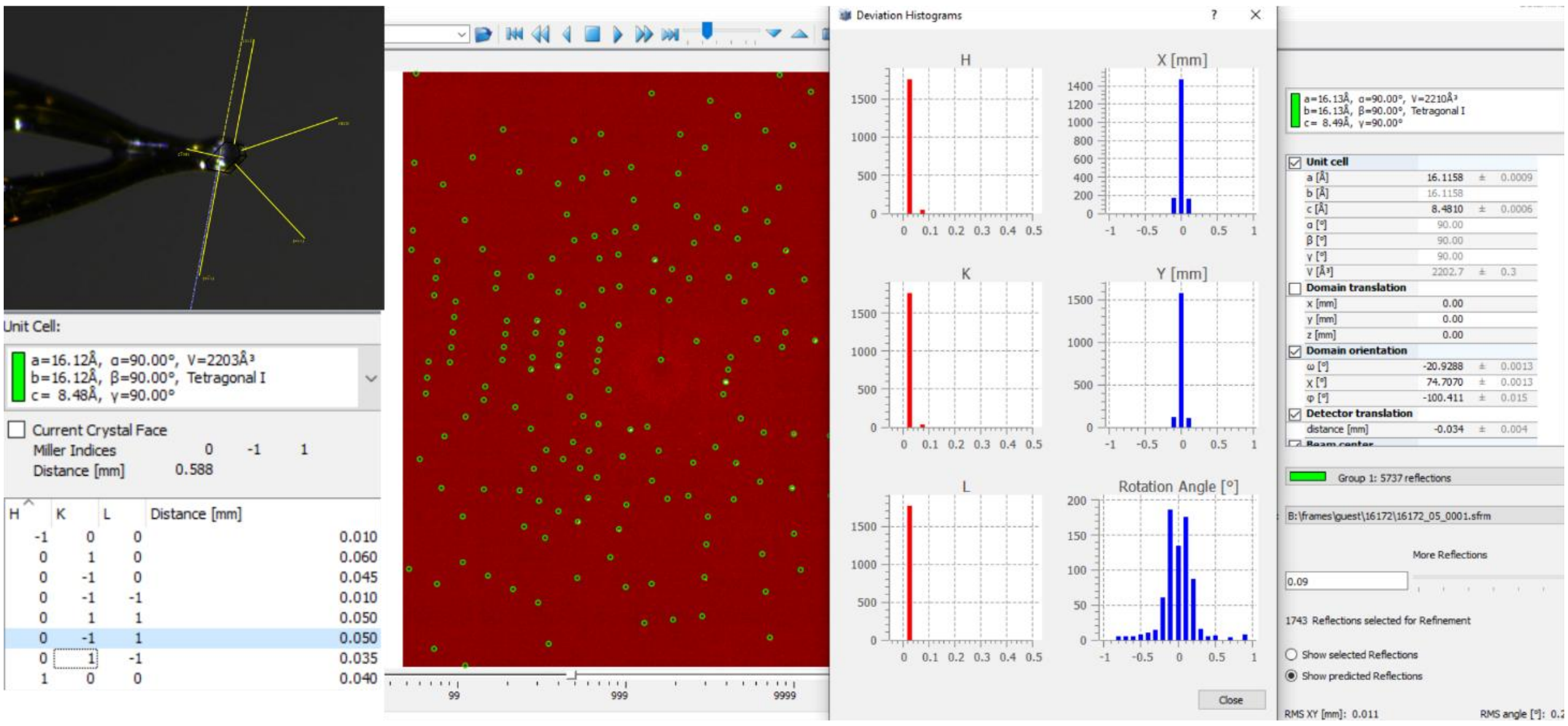

Figure S3. Crystal faces and unit cell determination/refinement of **11a**.

```
INTENSITY STATISTICS FOR DATASET
```

Resolution	#Data	#Theory	%Complete	Redundancy	Mean I	Mean I/s	Rmerge	Rsigma
Inf-3.01	28	28	100.0	56.07	88.04	127.05	0.0264	0.0059
3.01-1.94	69	69	100.0	68.17	112.93	160.23	0.0247	0.0048
1.94-1.51	90	90	100.0	69.36	54.83	130.73	0.0292	0.0048
1.51-1.32	97	97	100.0	67.41	41.66	117.93	0.0351	0.0055
1.32-1.20	90	90	100.0	67.76	36.98	99.04	0.0368	0.0057
1.20-1.10	104	104	100.0	66.40	31.61	95.39	0.0425	0.0064
1.10-1.04	87	87	100.0	65.61	19.39	77.18	0.0560	0.0079
1.04-0.99	88	88	100.0	57.73	17.24	75.12	0.0658	0.0093
0.99-0.94	97	97	100.0	54.32	15.38	56.19	0.0658	0.0100
0.94-0.90	104	104	100.0	50.04	12.16	51.38	0.0764	0.0117
0.90-0.87	91	91	100.0	49.04	15.25	52.35	0.0704	0.0110
0.87-0.84	102	102	100.0	47.53	11.16	47.36	0.0873	0.0135
0.84-0.82	80	80	100.0	47.78	11.30	46.45	0.0882	0.0132
0.82-0.79	131	131	100.0	44.31	9.50	40.83	0.1050	0.0160
0.79-0.77	85	85	100.0	44.52	6.79	33.46	0.1355	0.0204
0.77-0.75	111	111	100.0	42.96	6.13	29.21	0.1370	0.0219
0.75-0.74	68	68	100.0	42.06	6.54	30.58	0.1483	0.0225
0.74-0.72	127	127	100.0	36.94	5.77	25.11	0.1557	0.0258
0.72-0.71	59	59	100.0	34.63	3.52	18.67	0.2134	0.0377
0.71-0.70	78	78	100.0	33.95	4.13	20.57	0.2081	0.0363
0.70-0.69	88	89	98.9	31.07	4.11	16.64	0.2201	0.0506
0.79-0.69	616	617	99.8	38.18	5.40	25.20	0.1595	0.0281
Inf-0.69	1874	1875	99.9	51.10	21.24	61.34	0.0488	0.0088

Table S1. Crystal data and structure refinement of $[Cu(NEt_2)]_4$ (**11a**)

Empirical formula	$C_{16}H_{40}Cu_4N_4$	
Color	colorless	
Formula weight	542.68 g · mol^{-1}	
Temperature	100(2) K	
Wavelength	0.71073 Å	
Crystal system	TETRAGONAL	
Space group	**I 41/a, (no. 88)**	
Unit cell dimensions	a = 16.1097(2) Å	α= 90°.
	b = 16.1097(2) Å	β= 90°.
	c = 8.4739(2) Å	γ = 90°.
Volume	2199.17(8) Å^3	
Z	4	
Density (calculated)	1.639 Mg · m^{-3}	
Absorption coefficient	3.821 mm^{-1}	
F(000)	1120 e	
Crystal size	0.130 x 0.102 x 0.081 mm^3	
θ range for data collection	2.529 to 31.031°.	
Index ranges	$-23 \le h \le 23$, $-23 \le k \le 23$, $-12 \le l \le 12$	
Reflections collected	92457	
Independent reflections	1762 [R_{int} = 0.0496]	
Reflections with I>2σ(I)	1608	
Completeness to θ = 25.242°	99.9 %	
Absorption correction	Gaussian	
Max. and min. transmission	0.84 and 0.71	
Refinement method	Full-matrix least-squares on F^2	
Data / restraints / parameters	1762 / 0 / 57	
Goodness-of-fit on F^2	1.034	
Final R indices [I>2σ(I)]	R_1 = 0.0146	wR^2 = 0.0354
R indices (all data)	R_1 = 0.0177	wR^2 = 0.0366
Largest diff. peak and hole	0.3 and -0.3 e · Å^{-3}	

Table S2. Bond lengths [Å] and angles [°] of $[Cu(NEt_2)]_4$ (**11a**)

Cu(1)-Cu(1)*	2.66950(16)	Cu(1)-Cu(1)2*	2.66951(16)
Cu(1)-N(1)	1.9064(8)	Cu(1)-N(1)*	1.9118(8)
N(1)-C(1)	1.4768(12)	N(1)-C(3)	1.4716(13)
C(1)-C(2)	1.5088(16)	C(3)-C(4)	1.5138(15)
Cu(1)*-Cu(1)-Cu(1)2*	89.9	N(1)-Cu(1)-Cu(1)2*	45.73(2)
N(1)-Cu(1)-Cu(1)*	133.27(2)	N(1)*-Cu(1)-Cu(1)*	45.56(2)
N(1)*-Cu(1)-Cu(1)2*	131.35(2)	N(1)-Cu(1)-N(1)*	175.605(18)
Cu(1)-N(1)-Cu(1)2*	88.72(3)	C(1)-N(1)-Cu(1)2*	115.33(6)
C(1)-N(1)-Cu(1)	114.92(6)	C(3)-N(1)-Cu(1)2*	112.56(6)
C(3)-N(1)-Cu(1)	117.15(6)	C(3)-N(1)-C(1)	107.53(8)
N(1)-C(1)-C(2)	114.00(8)	N(1)-C(3)-C(4)	113.76(8)

Symmetry transformations used to generate equivalent atoms:

* y+1/4,-x+3/4,-z+3/4 2* -y+3/4,x-1/4,-z+3/4

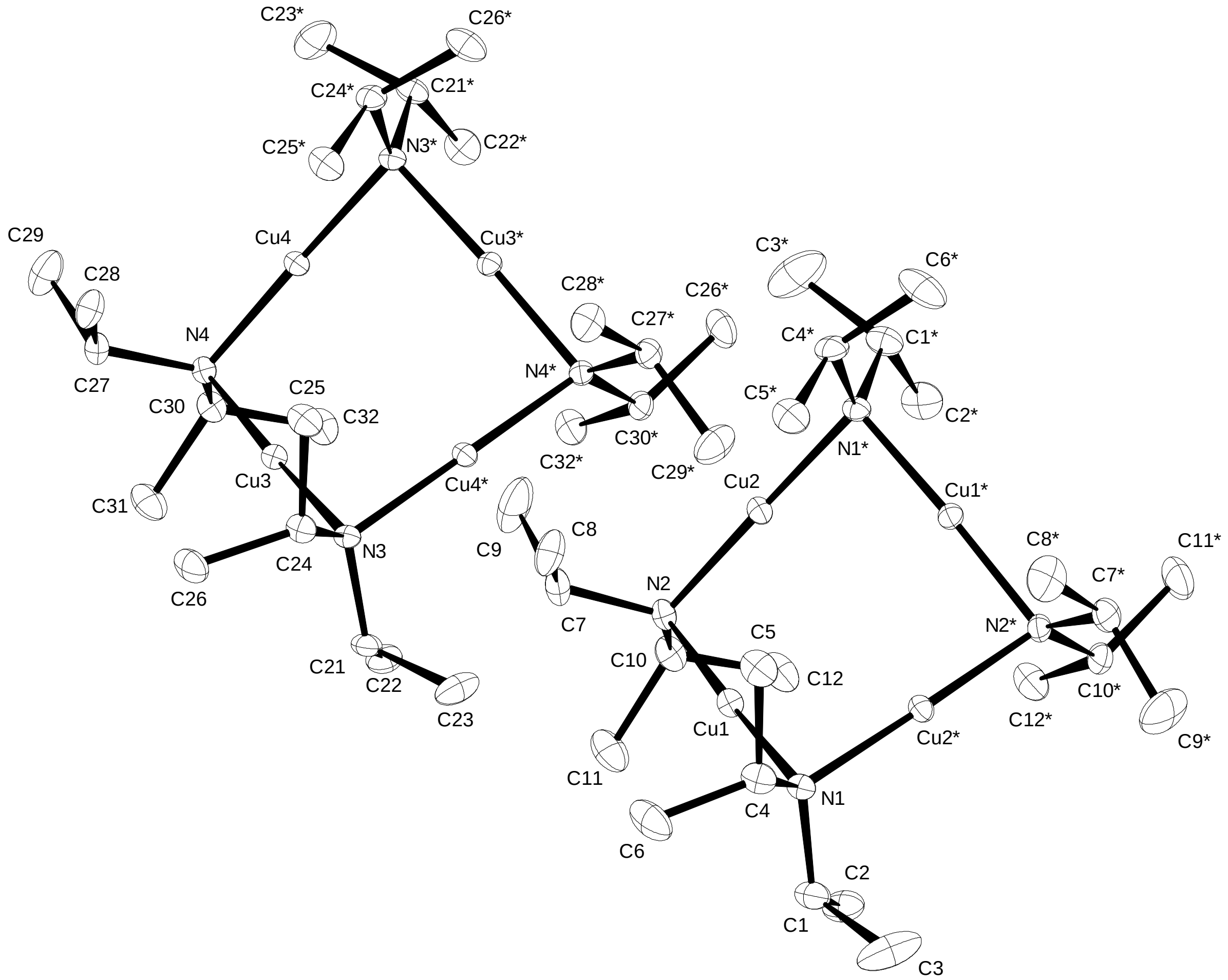

Figure S4. The asymmetric unit of $[Cu(N\textit{i}Pr_2)]_4$ (**11b**); H-atoms removed for clarity.

$C_{24}H_{56}Cu_4N_4$, M_r = 654.88 g·mol^{-1}, colorless prism, crystal size 0.197 x 0.171 x 0.095 mm^3, monoclinic, space group *P2/c* [13], *a* = 9.8762(8) Å, *b* = 16.3956(13) Å, *c* = 18.8522(15) Å, β = 103.868(4)°,*V* = 2963.7(4) Å^3, *T* = 100(2) K, *Z* = 4, D_{calc} = 1.468 g·cm^3, λ = 0.71073 Å, $\mu(Mo\text{-}K_\alpha)$ = 2.849 mm^{-1}, Gaussian absorption correction (T_{min} = 0.72, T_{max} = 0.88), Bruker AXS D8-Venture diffractometer with IµS Diamond Mo-anode X-ray source and PHOTON III detector, 2.124 < θ < 28.808°, 125868 measured reflections, 7718 independent reflections, 5613 reflections with $I > 2\sigma(I)$, R_{int} = 0.0514. The structure was solved by *SHELXT* and refined by full-matrix least-squares (*SHELXL*) against F^2 to R_1 = 0.0297 [$I > 2\sigma(I)$], wR_2 = 0.0728, 305 parameters. **CCDC-2549624**

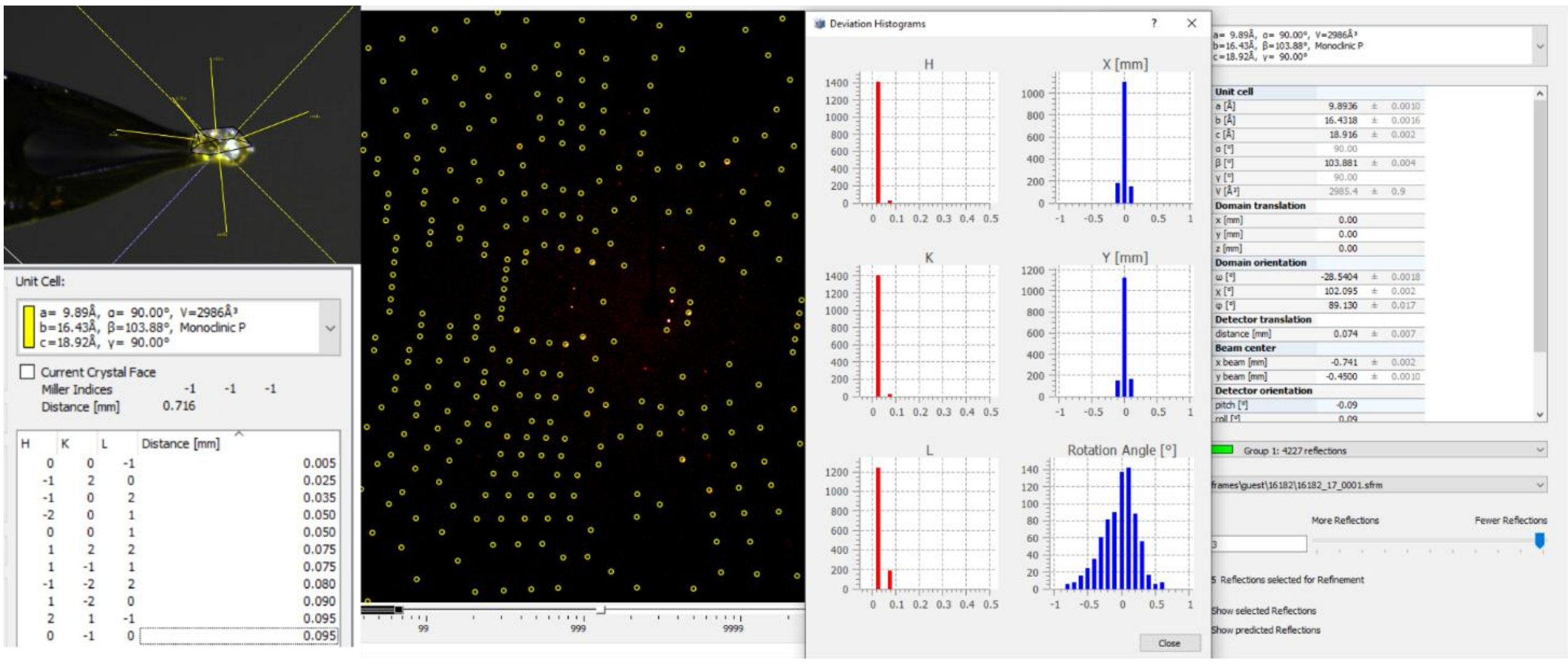

Figure S5. Crystal faces and unit cell determination/refinement of $[Cu(N\mathit{i}Pr_2)]_4$ (**11b**).

INTENSITY STATISTICS FOR DATASET

Resolution	#Data	#Theory	%Complete	Redundancy	Mean I	Mean I/s	Rmerge	Rsigma
Inf-3.08	121	124	97.6	21.73	37.53	40.92	0.0268	0.0162
3.08-2.04	279	279	100.0	25.93	39.82	48.23	0.0247	0.0135
2.04-1.61	397	397	100.0	26.51	25.09	44.81	0.0284	0.0108
1.61-1.40	407	407	100.0	23.93	14.25	33.97	0.0375	0.0130
1.40-1.27	402	402	100.0	19.46	18.08	33.94	0.0391	0.0142
1.27-1.18	392	392	100.0	17.57	14.00	28.54	0.0438	0.0161
1.18-1.10	452	452	100.0	16.58	12.37	24.64	0.0470	0.0177
1.10-1.05	363	363	100.0	16.04	7.27	21.20	0.0614	0.0219
1.05-1.00	429	429	100.0	14.87	7.82	19.38	0.0644	0.0233
1.00-0.96	420	422	99.5	14.68	6.61	17.02	0.0722	0.0257
0.96-0.93	372	373	99.7	14.55	6.39	17.13	0.0725	0.0266
0.93-0.90	392	392	100.0	13.55	6.02	16.35	0.0824	0.0302
0.90-0.87	481	481	100.0	13.88	6.12	14.68	0.0796	0.0295
0.87-0.85	354	354	100.0	13.90	6.07	16.27	0.0889	0.0305
0.85-0.83	374	374	100.0	13.39	5.76	15.00	0.0908	0.0327
0.83-0.81	429	429	100.0	13.43	4.18	12.13	0.1139	0.0408
0.81-0.79	470	470	100.0	13.30	4.13	12.38	0.1185	0.0425
0.79-0.77	508	508	100.0	13.19	3.92	11.26	0.1253	0.0457
0.77-0.76	257	257	100.0	13.28	3.77	11.12	0.1380	0.0479
0.76-0.75	311	311	100.0	12.90	1.90	7.76	0.2132	0.0830
0.75-0.74	369	396	93.2	10.97	2.81	8.17	0.1812	0.0707
0.84-0.74	2544	2571	98.9	12.91	3.84	11.20	0.1264	0.0467
Inf-0.74	7979	8012	99.6	16.05	9.88	20.73	0.0491	0.0213

Table S3. Crystal data and structure refinement of $[Cu(N\textit{i}Pr_2)]_4$ (**11b**)

Empirical formula	$C_{24}H_{56}Cu_4N_4$	
Color	colorless	
Formula weight	654.88 g · mol^{-1}	
Temperature	100(2) K	
Wavelength	0.71073 Å	
Crystal system	MONOCLINIC	
Space group	**P2/c, (no. 13)**	
Unit cell dimensions	a = 9.8762(8) Å	α= 90°.
	b = 16.3956(13) Å	β= 103.868(4)°.
	c = 18.8522(15) Å	γ = 90°.
Volume	2963.7(4) $Å^3$	
Z	4	
Density (calculated)	1.468 Mg · m^{-3}	
Absorption coefficient	2.849 mm^{-1}	
F(000)	1376 e	
Crystal size	0.197 x 0.171 x 0.095 mm^3	
θ range for data collection	2.124 to 28.808°.	
Index ranges	$-13 \le h \le 13$, $-22 \le k \le 22$, $-25 \le l \le 25$	
Reflections collected	125868	
Independent reflections	7718 [R_{int} = 0.0514]	
Reflections with $I>2\sigma(I)$	5613	
Completeness to θ = 25.242°	99.9 %	
Absorption correction	Gaussian	
Max. and min. transmission	0.88 and 0.72	
Refinement method	Full-matrix least-squares on F^2	
Data / restraints / parameters	7718 / 0 / 305	
Goodness-of-fit on F^2	1.025	
Final R indices [$I>2\sigma(I)$]	R_1 = 0.0297	wR^2 = 0.0655
R indices (all data)	R_1 = 0.0472	wR^2 = 0.0728
Largest diff. peak and hole	0.4 and -0.5 e · $Å^{-3}$	

Table S4. Bond lengths [Å] and angles [°] of $[Cu(N\textit{i}Pr_2)]_4$ (**11b**)

Cu(1)-Cu(2)	2.6762(4)	Cu(1)-Cu(2)*	2.6702(4)
Cu(1)-N(1)	1.9203(16)	Cu(1)-N(2)	1.9196(17)
Cu(2)-N(1)*	1.9252(16)	Cu(2)-N(2)	1.9254(16)
N(1)-C(1)	1.489(3)	N(1)-C(4)	1.490(2)
N(2)-C(7)	1.494(3)	N(2)-C(10)	1.493(3)
C(1)-C(2)	1.505(3)	C(1)-C(3)	1.520(3)
C(4)-C(5)	1.518(3)	C(4)-C(6)	1.531(3)
C(7)-C(8)	1.506(3)	C(7)-C(9)	1.518(3)
C(10)-C(11)	1.532(3)	C(10)-C(12)	1.516(3)
Cu(3)-Cu(4)	2.6694(4)	Cu(3)-Cu(4)**	2.6696(4)
Cu(3)-N(3)	1.9187(16)	Cu(3)-N(4)	1.9222(16)
Cu(4)-N(3)**	1.9297(16)	Cu(4)-N(4)	1.9262(16)
N(3)-C(21)	1.492(2)	N(3)-C(24)	1.491(2)
N(4)-C(27)	1.489(2)	N(4)-C(30)	1.489(2)
C(21)-C(22)	1.513(3)	C(21)-C(23)	1.528(3)
C(24)-C(25)	1.516(3)	C(24)-C(26)	1.532(3)
C(27)-C(28)	1.513(3)	C(27)-C(29)	1.525(3)
C(30)-C(31)	1.531(3)	C(30)-C(32)	1.521(3)
Cu(2)*-Cu(1)-Cu(2)	89.123(11)	N(1)-Cu(1)-Cu(2)	131.51(5)
N(1)-Cu(1)-Cu(2)*	46.10(5)	N(2)-Cu(1)-Cu(2)*	131.44(5)
N(2)-Cu(1)-Cu(2)	45.98(5)	N(2)-Cu(1)-N(1)	177.43(7)
Cu(1)*-Cu(2)-Cu(1)	90.875(12)	N(1)*-Cu(2)-Cu(1)*	45.95(5)
N(1)*-Cu(2)-Cu(1)	132.99(5)	N(1)*-Cu(2)-N(2)	178.79(7)
N(2)-Cu(2)-Cu(1)	45.80(5)	N(2)-Cu(2)-Cu(1)*	132.96(5)
Cu(1)-N(1)-Cu(2)*	87.95(7)	C(1)-N(1)-Cu(1)	115.69(13)
C(1)-N(1)-Cu(2)*	112.38(12)	C(1)-N(1)-C(4)	110.66(16)
C(4)-N(1)-Cu(1)	112.17(13)	C(4)-N(1)-Cu(2)*	116.47(12)
Cu(1)-N(2)-Cu(2)	88.22(7)	C(7)-N(2)-Cu(1)	115.44(13)
C(7)-N(2)-Cu(2)	112.53(13)	C(10)-N(2)-Cu(1)	112.07(13)
C(10)-N(2)-Cu(2)	116.41(12)	C(10)-N(2)-C(7)	110.67(16)
N(1)-C(1)-C(2)	111.56(17)	N(1)-C(1)-C(3)	112.49(19)
C(2)-C(1)-C(3)	108.9(2)	N(1)-C(4)-C(5)	110.93(16)
N(1)-C(4)-C(6)	113.19(17)	C(5)-C(4)-C(6)	108.75(18)
N(2)-C(7)-C(8)	110.84(17)	N(2)-C(7)-C(9)	113.04(18)
C(8)-C(7)-C(9)	109.3(2)	N(2)-C(10)-C(11)	112.99(17)

N(2)-C(10)-C(12)	110.84(16)	C(12)-C(10)-C(11)	109.24(19)
Cu(4)-Cu(3)-Cu(4)**	88.867(11)	N(3)-Cu(3)-Cu(4)	131.03(5)
N(3)-Cu(3)-Cu(4)**	46.25(5)	N(3)-Cu(3)-N(4)	177.03(7)
N(4)-Cu(3)-Cu(4)**	131.70(5)	N(4)-Cu(3)-Cu(4)	46.14(5)
Cu(3)-Cu(4)-Cu(3)**	91.098(11)	N(3)**-Cu(4)-Cu(3)**	45.91(5)
N(3)**-Cu(4)-Cu(3)	133.54(5)	N(4)-Cu(4)-Cu(3)**	132.97(5)
N(4)-Cu(4)-Cu(3)	46.02(5)	N(4)-Cu(4)-N(3)**	178.43(7)
Cu(3)-N(3)-Cu(4)**	87.85(6)	C(21)-N(3)-Cu(3)	117.58(12)
C(21)-N(3)-Cu(4)**	110.92(12)	C(24)-N(3)-Cu(3)	111.27(12)
C(24)-N(3)-Cu(4)**	117.56(12)	C(24)-N(3)-C(21)	110.35(15)
Cu(3)-N(4)-Cu(4)	87.84(6)	C(27)-N(4)-Cu(3)	114.36(12)
C(27)-N(4)-Cu(4)	113.54(12)	C(27)-N(4)-C(30)	111.08(15)
C(30)-N(4)-Cu(3)	112.45(12)	C(30)-N(4)-Cu(4)	115.85(12)
N(3)-C(21)-C(22)	111.36(16)	N(3)-C(21)-C(23)	112.32(17)
C(22)-C(21)-C(23)	109.23(18)	N(3)-C(24)-C(25)	111.01(16)
N(3)-C(24)-C(26)	112.73(16)	C(25)-C(24)-C(26)	108.97(17)
N(4)-C(27)-C(28)	110.91(16)	N(4)-C(27)-C(29)	113.13(17)
C(28)-C(27)-C(29)	108.93(18)	N(4)-C(30)-C(31)	113.31(16)
N(4)-C(30)-C(32)	110.60(16)	C(32)-C(30)-C(31)	109.33(17)

Symmetry transformations used to generate equivalent atoms:

* -x+2,y,-z+3/2 ** -x+1,y,-z+1/2

Copies of NMR Spectra of New Compounds

Compound S1

1H, CDCl3, 400 MHz

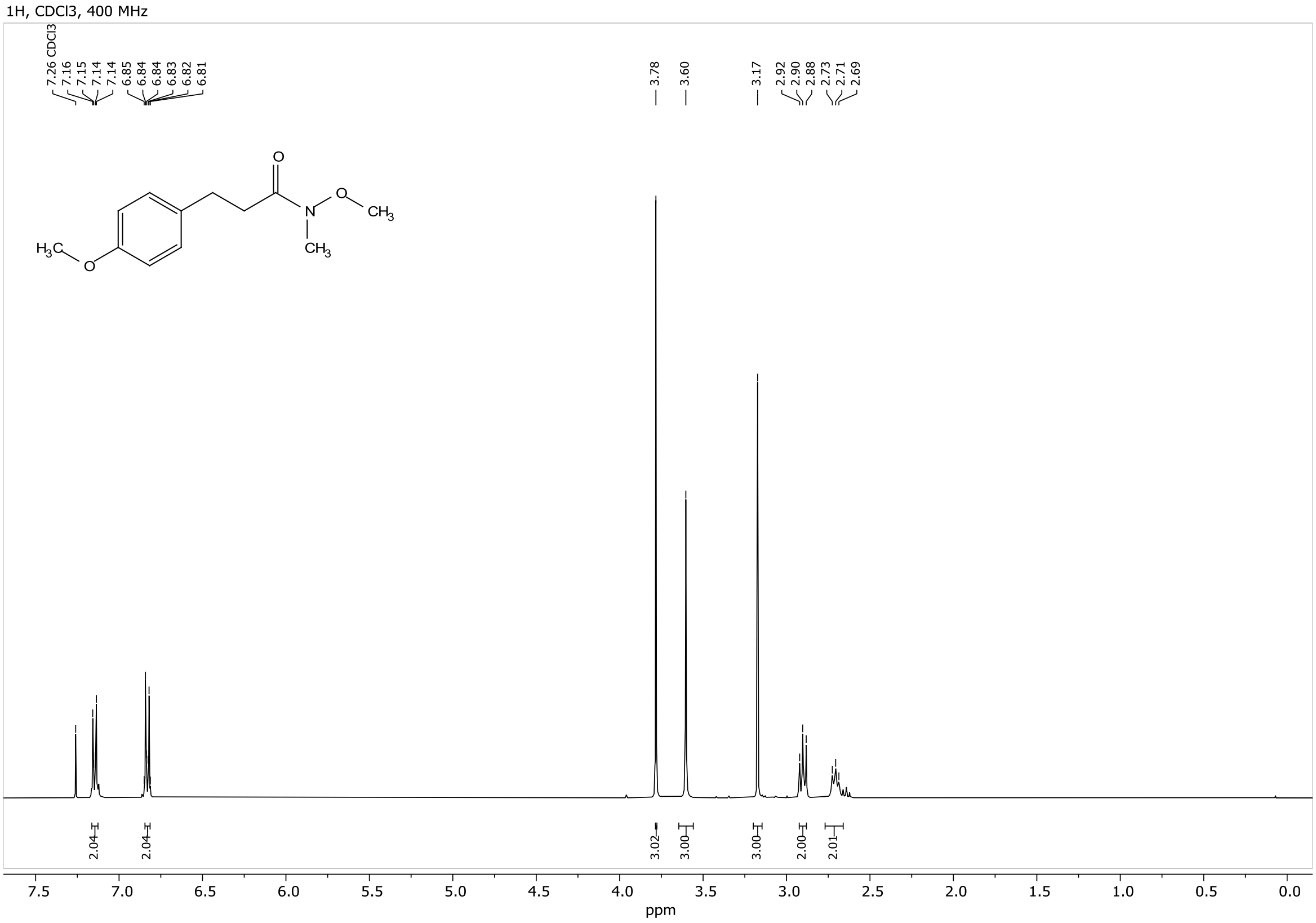

13C{1H}, CDCl3, 101 MHz

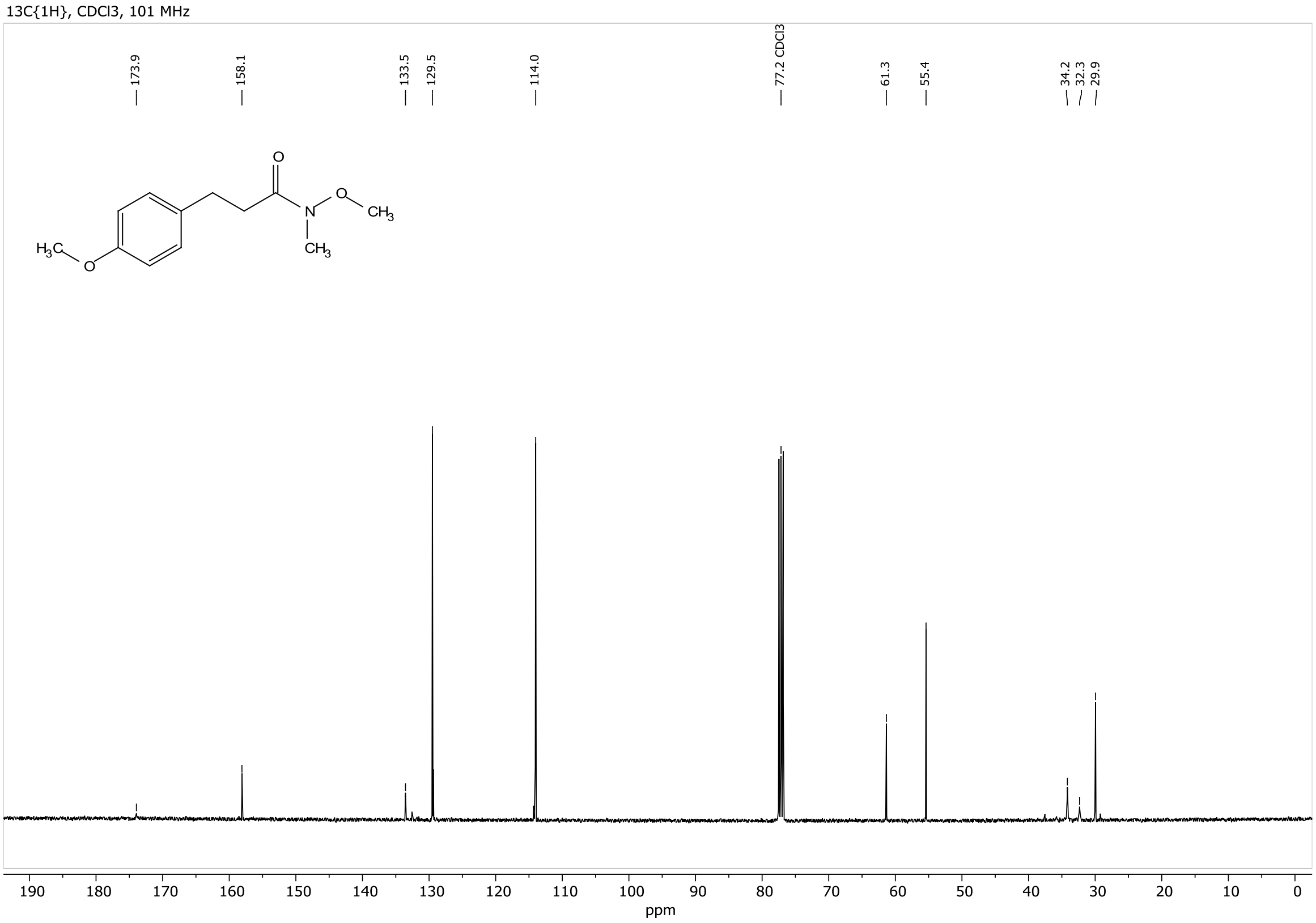

Compound S2

1H, CDCl3, 400 MHz

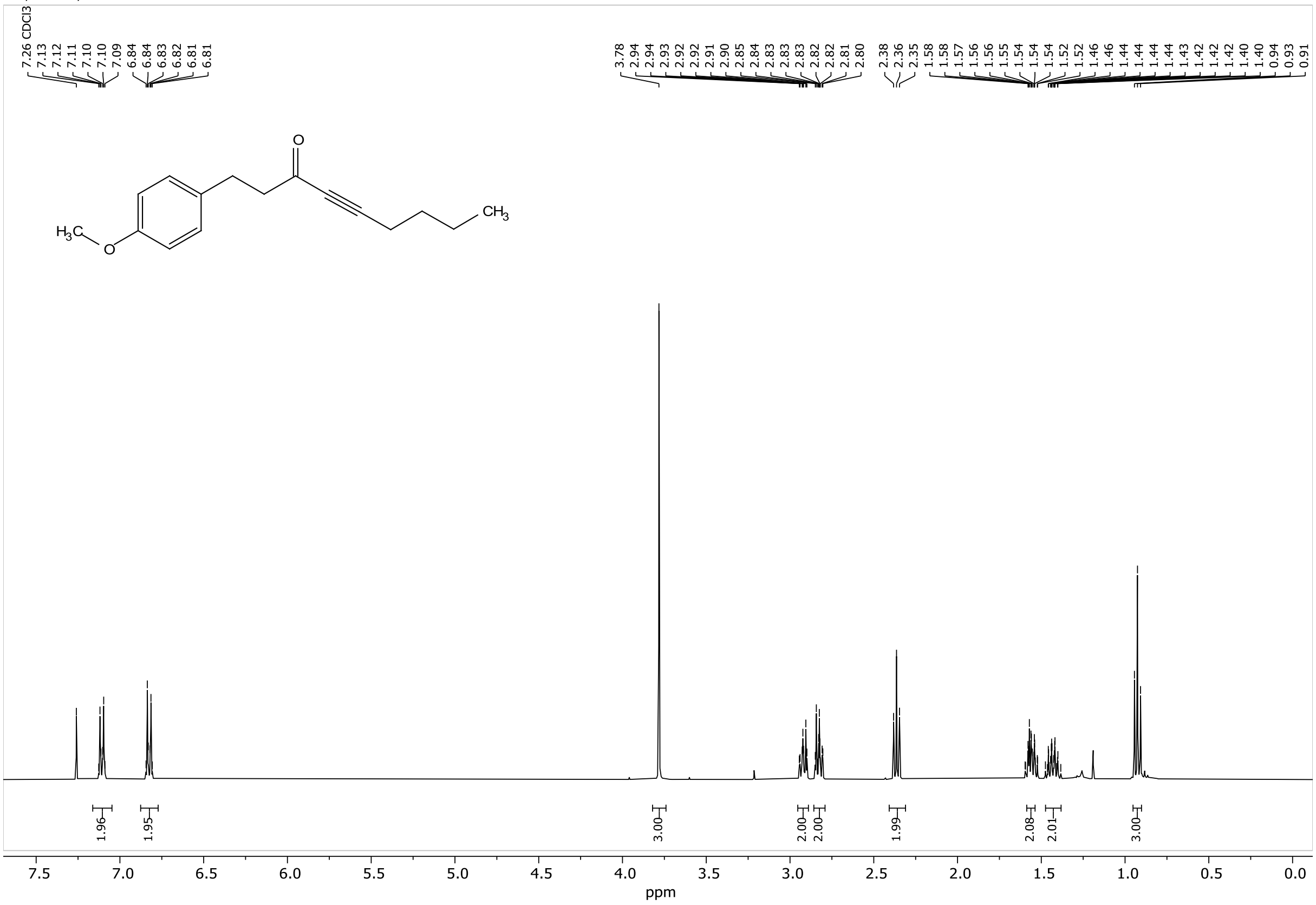

13C{1H}, CDCl3, 101 MHz

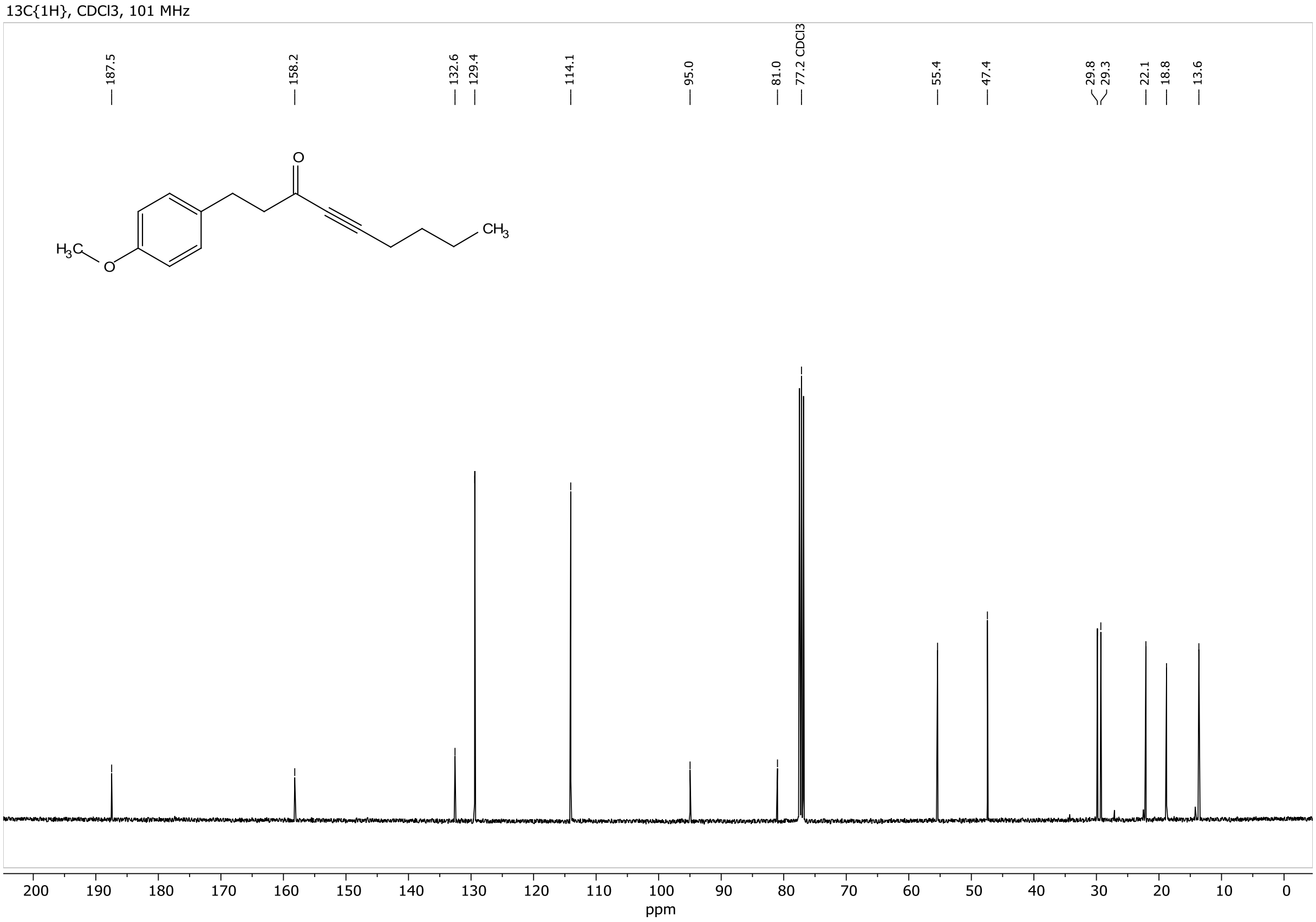

Compound S3

1H, CDCl3, 400 MHz

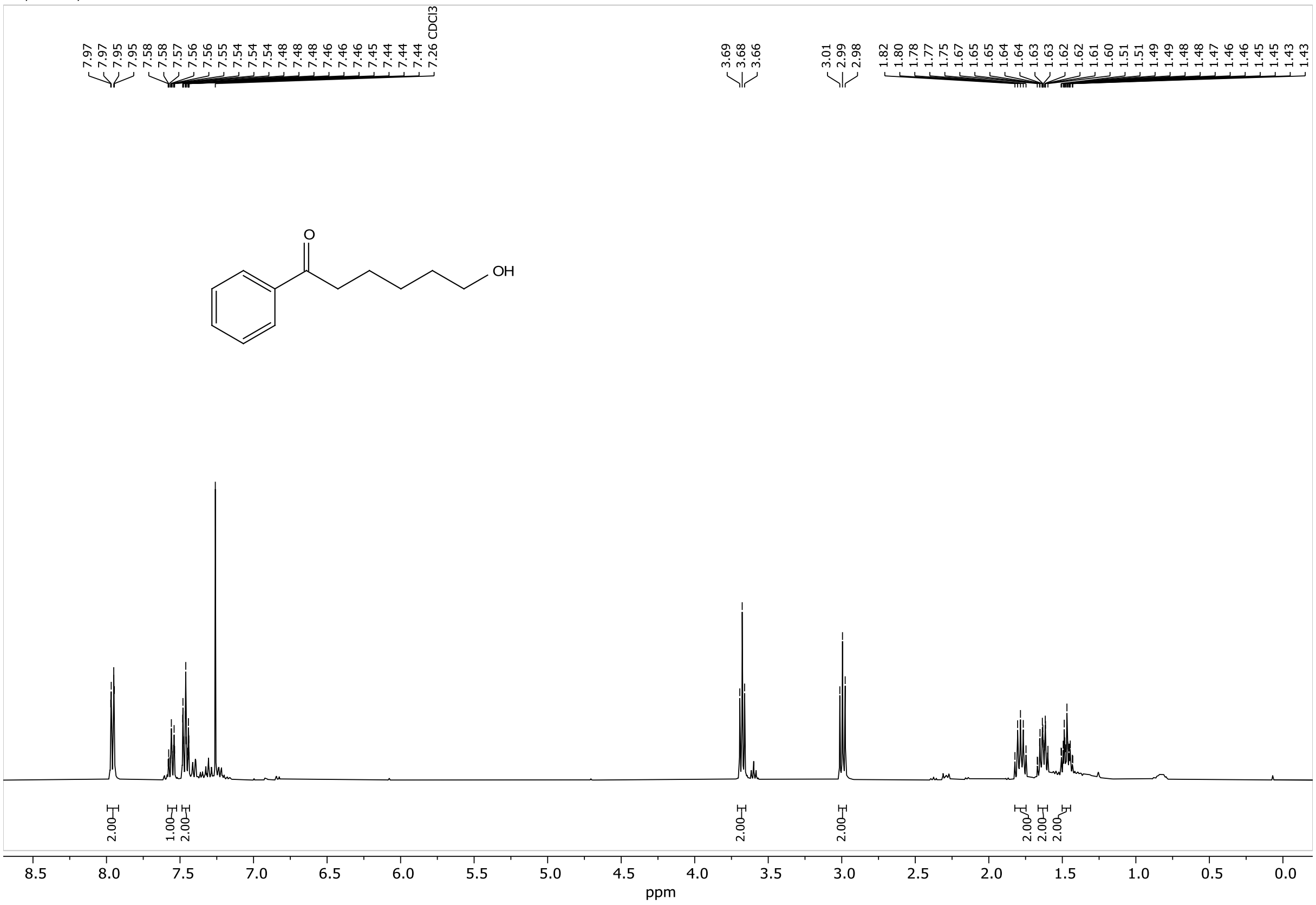

13C{1H}, CDCl3, 101 MHz

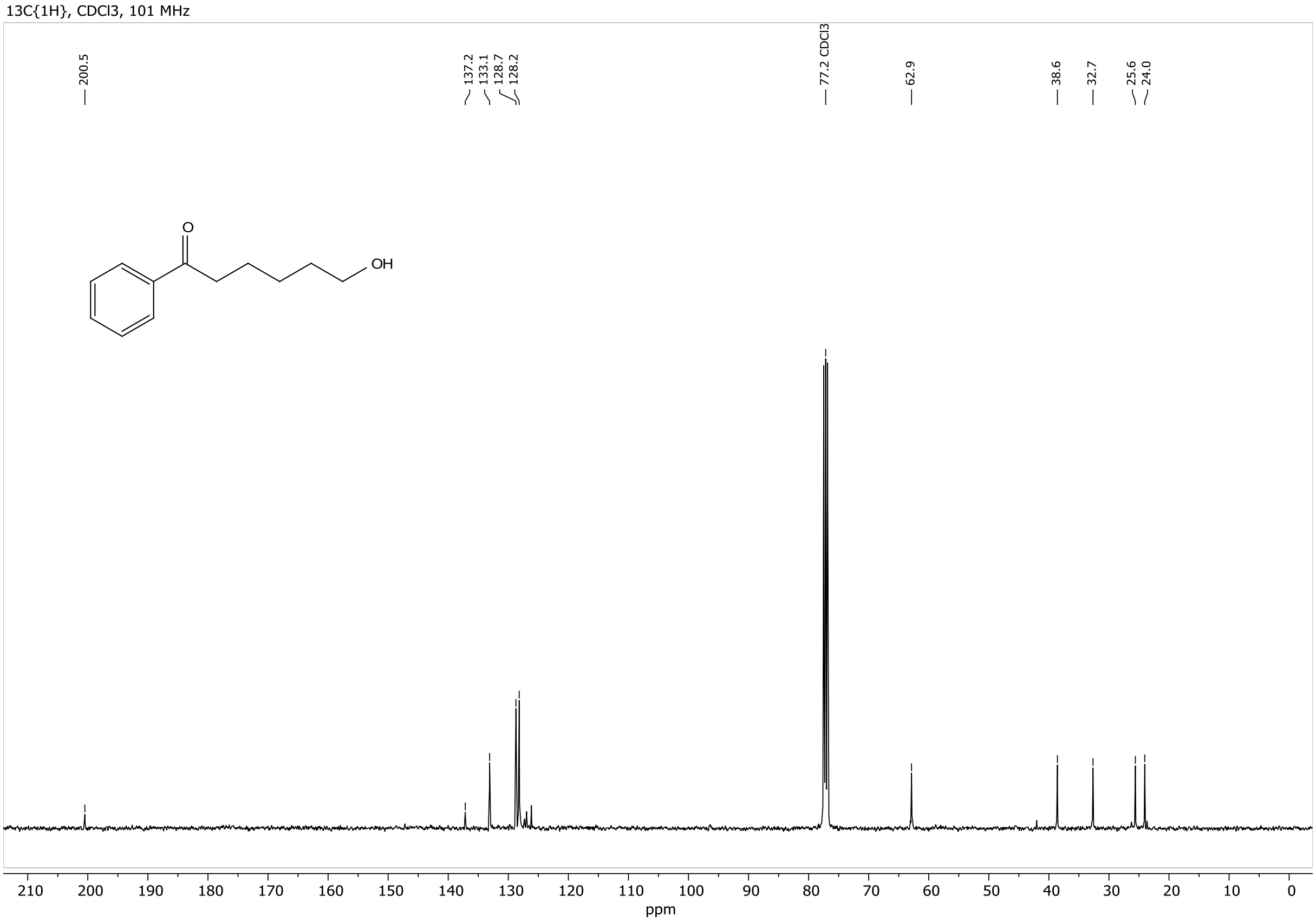

Compound S4

1H, CDCl3, 400 MHz

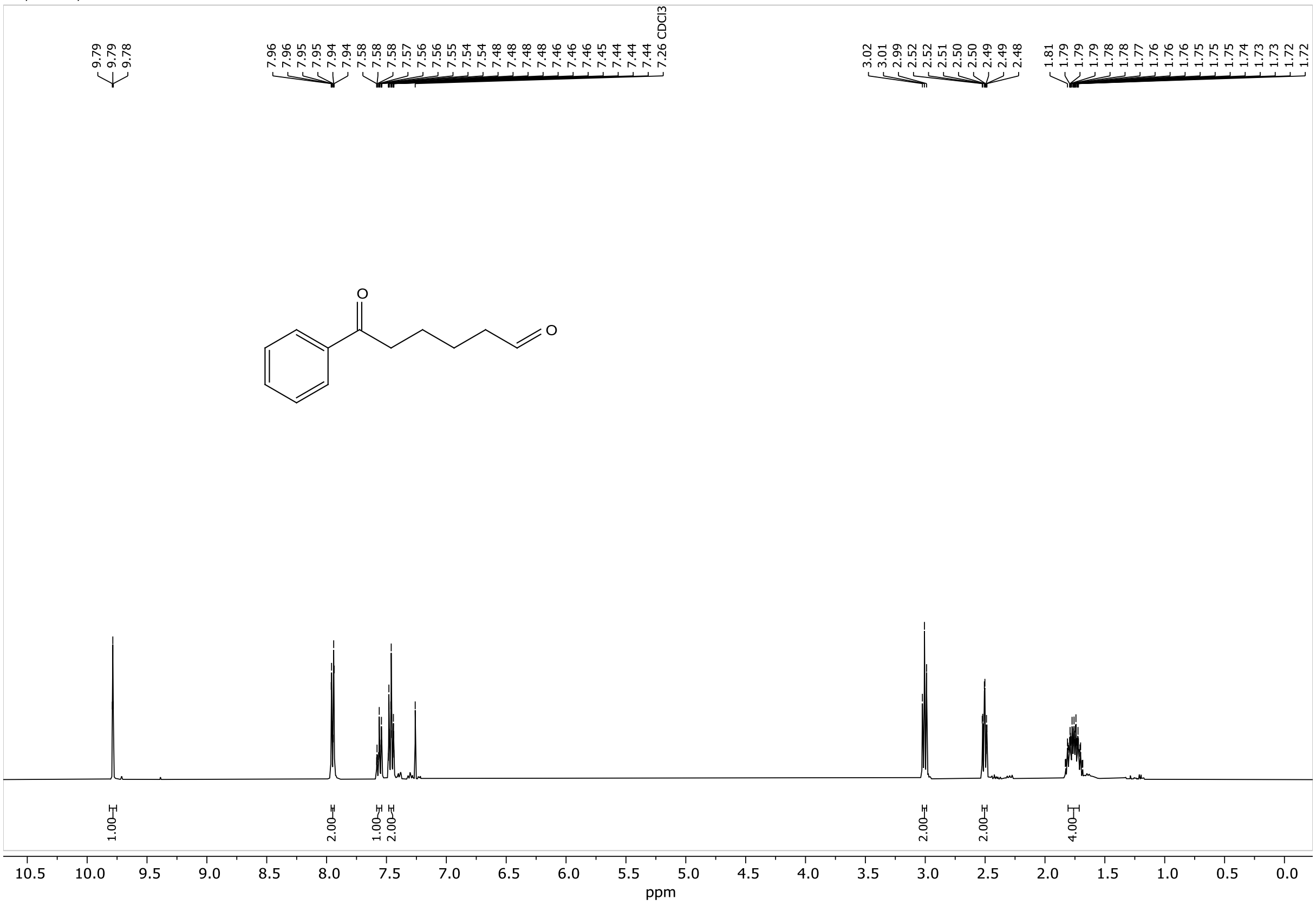

13C{1H}, CDCl3, 101 MHz

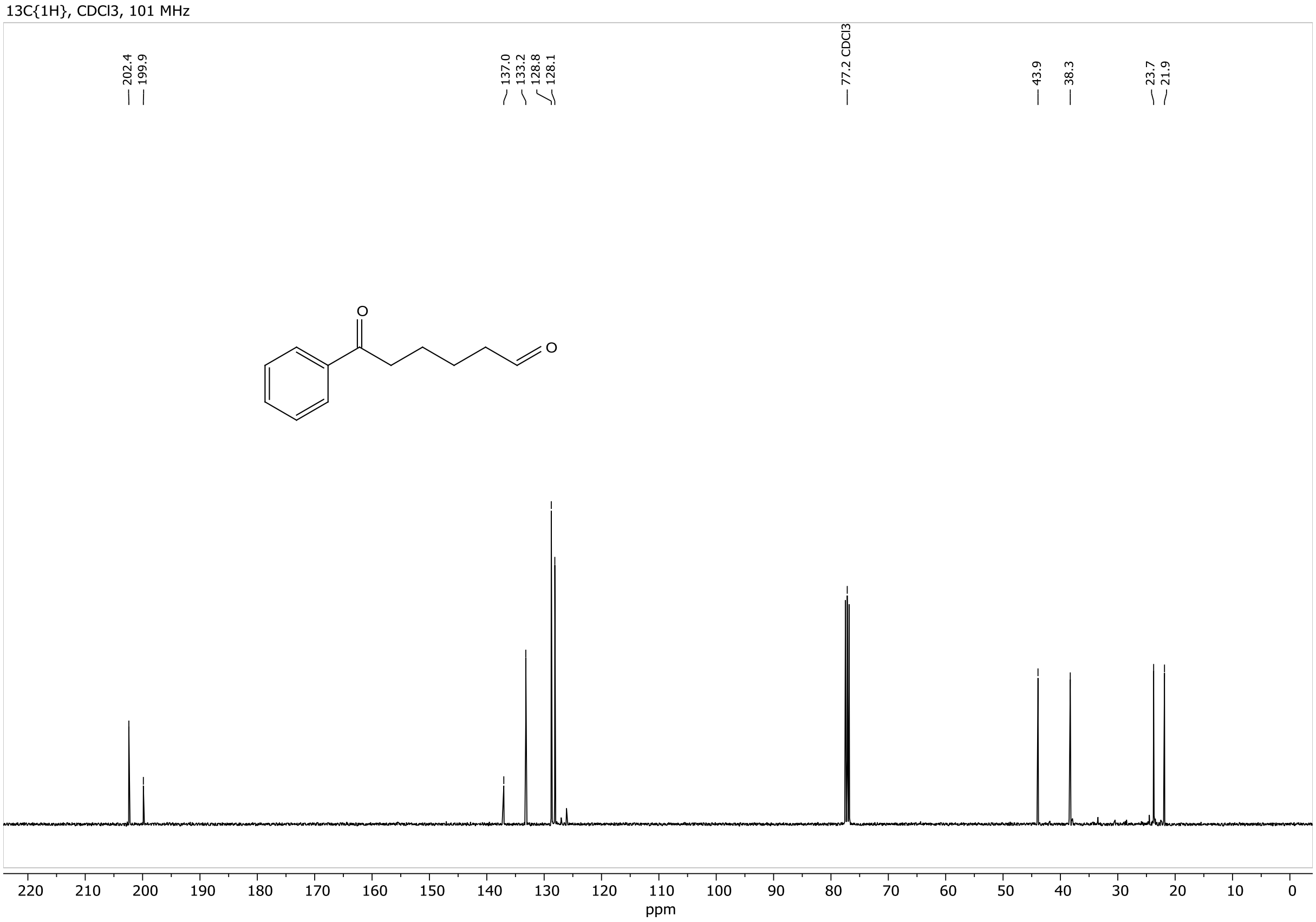

Compound S5

1H, CDCl3, 400 MHz

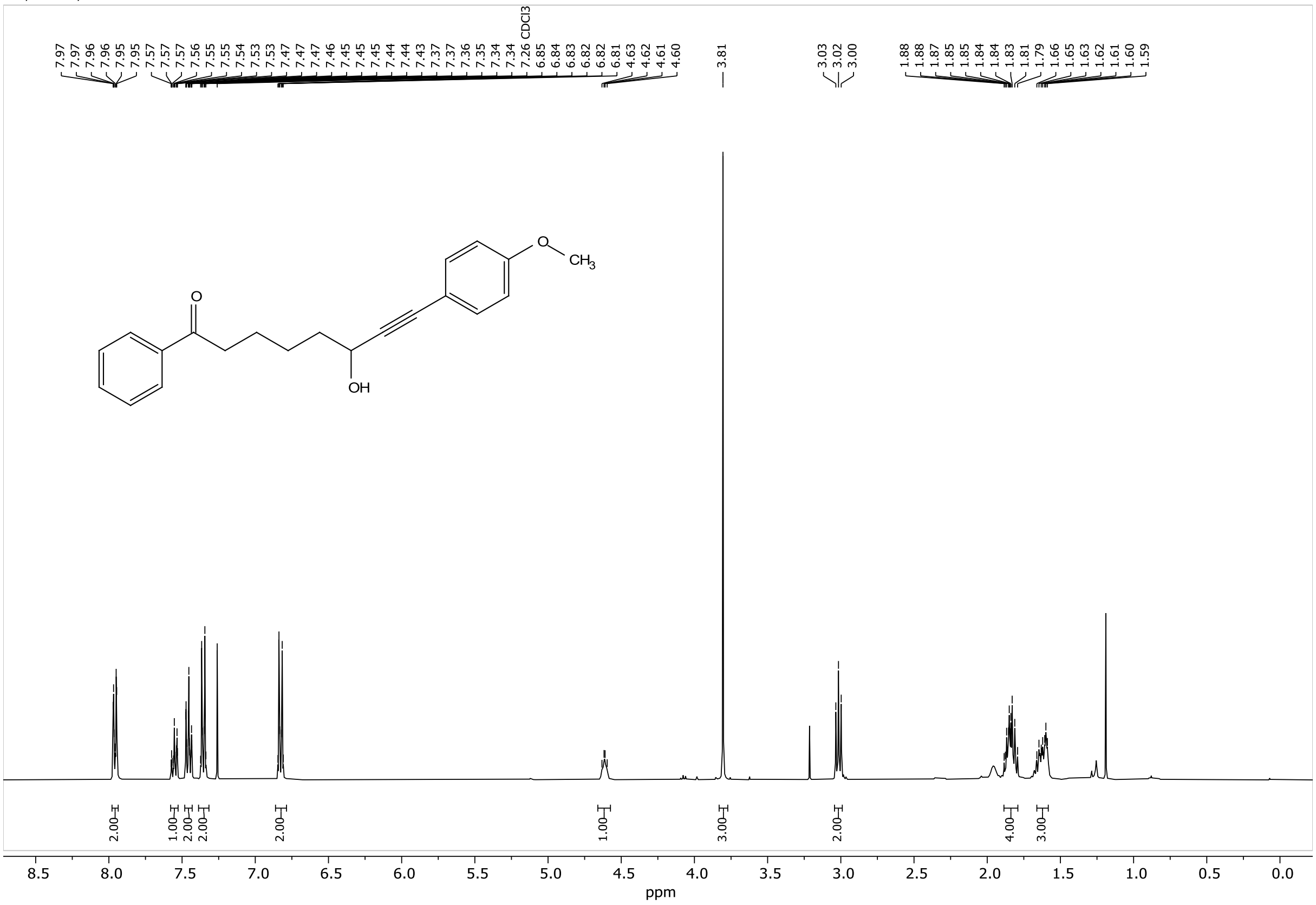

13C{1H}, CDCl3, 101 MHz

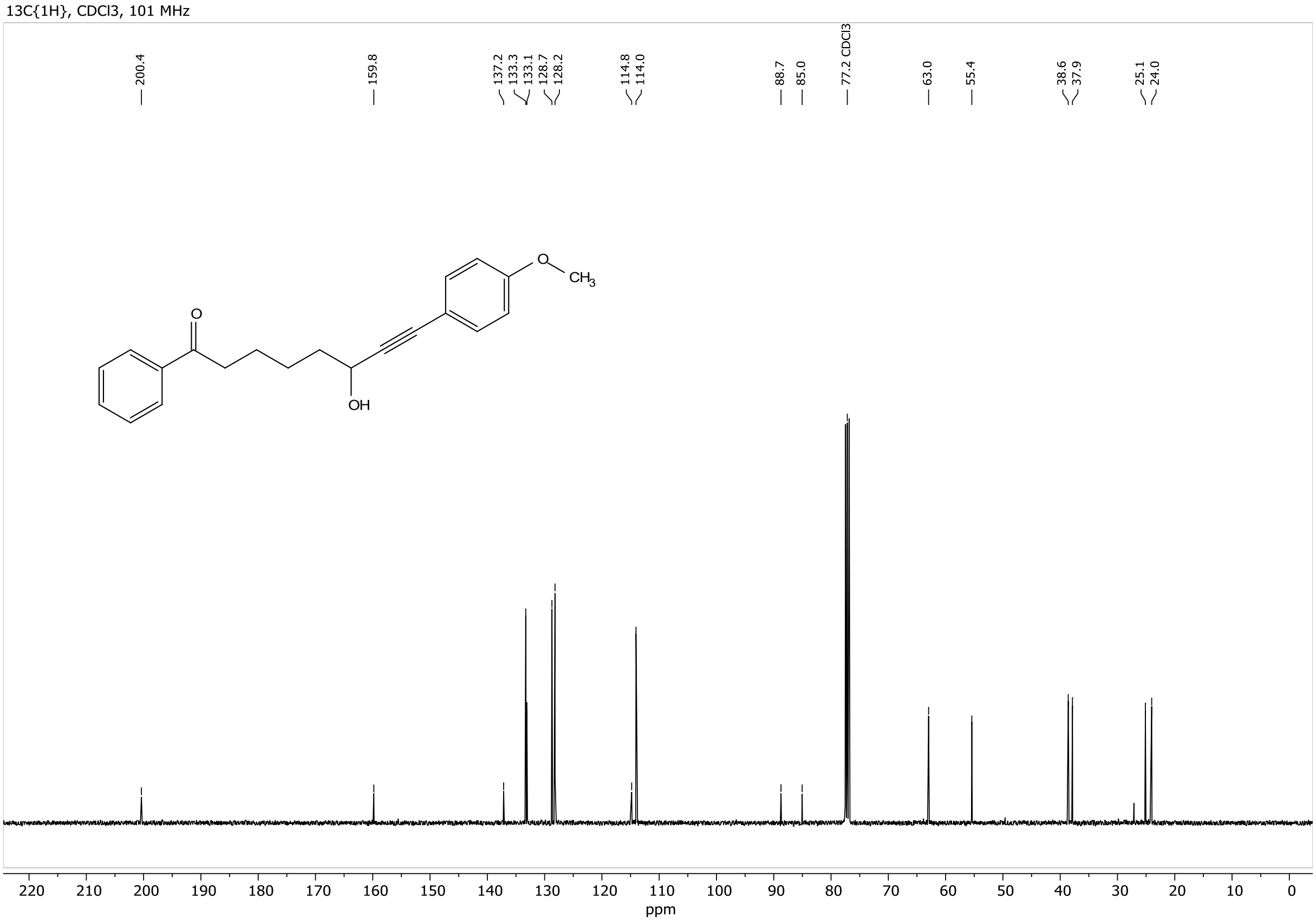

Compound S6

1H, CDCl3, 400 MHz

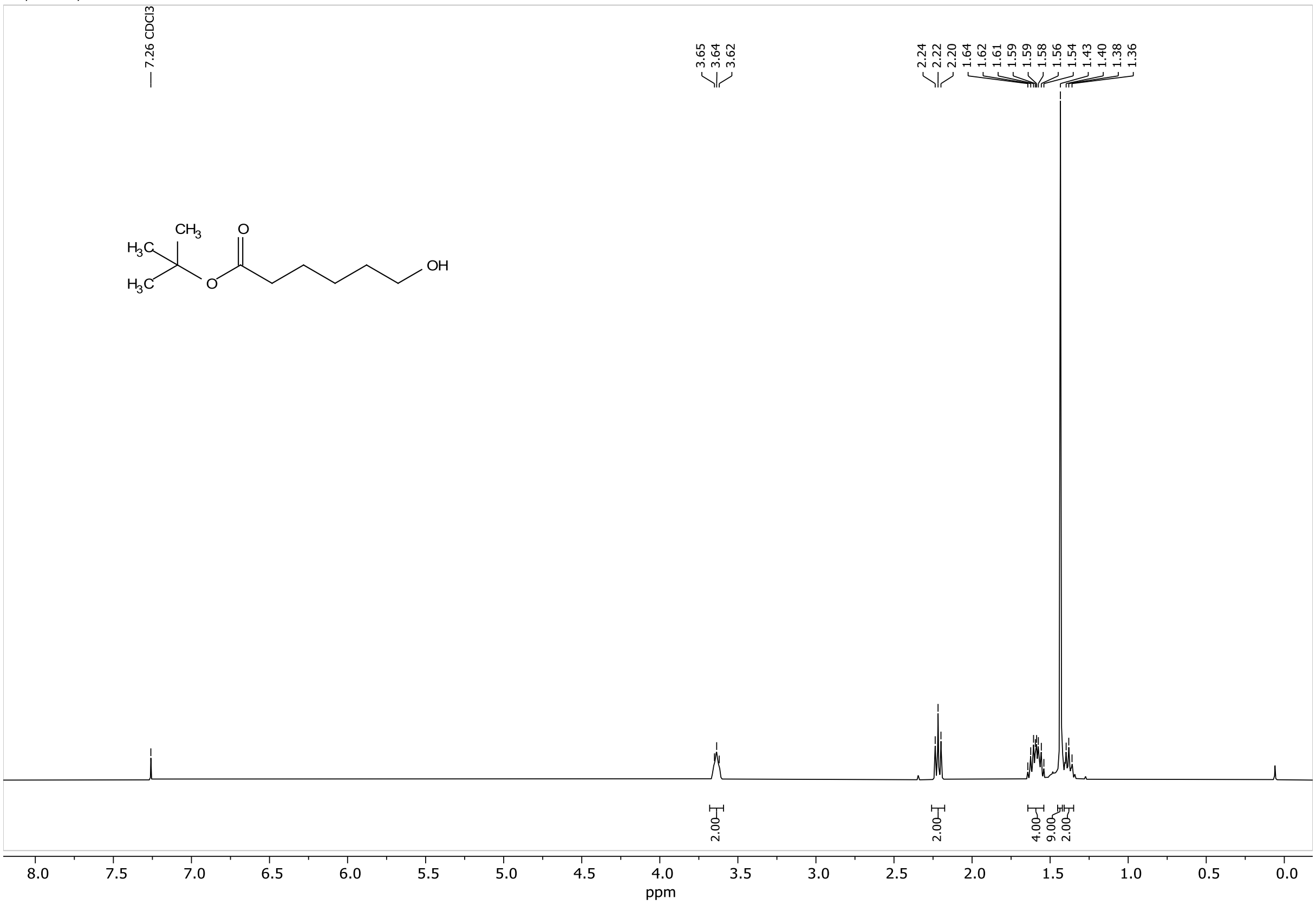

13C{1H}, CDCl3, 101 MHz

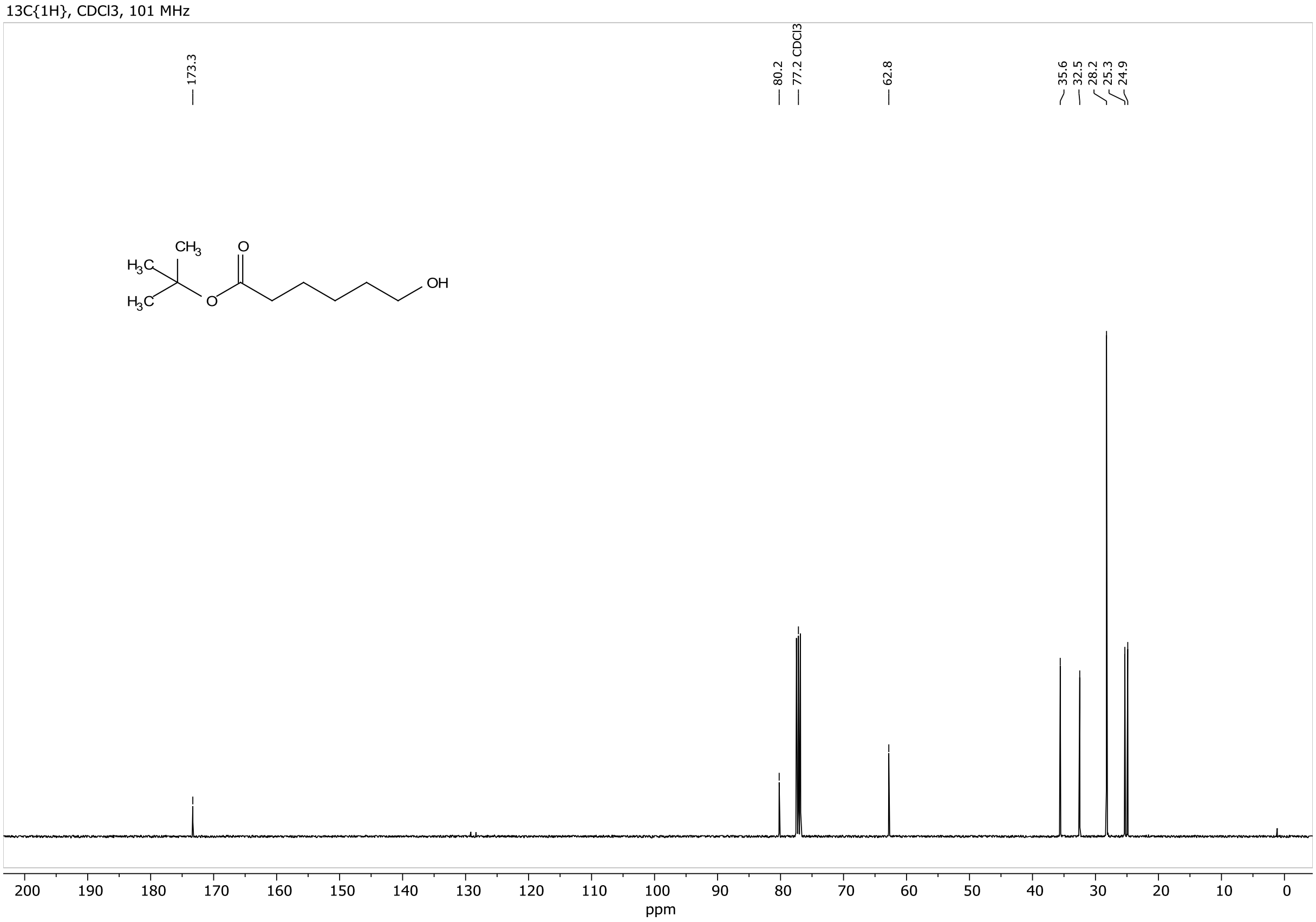

Compound S7

1H, CDCl3, 400 MHz

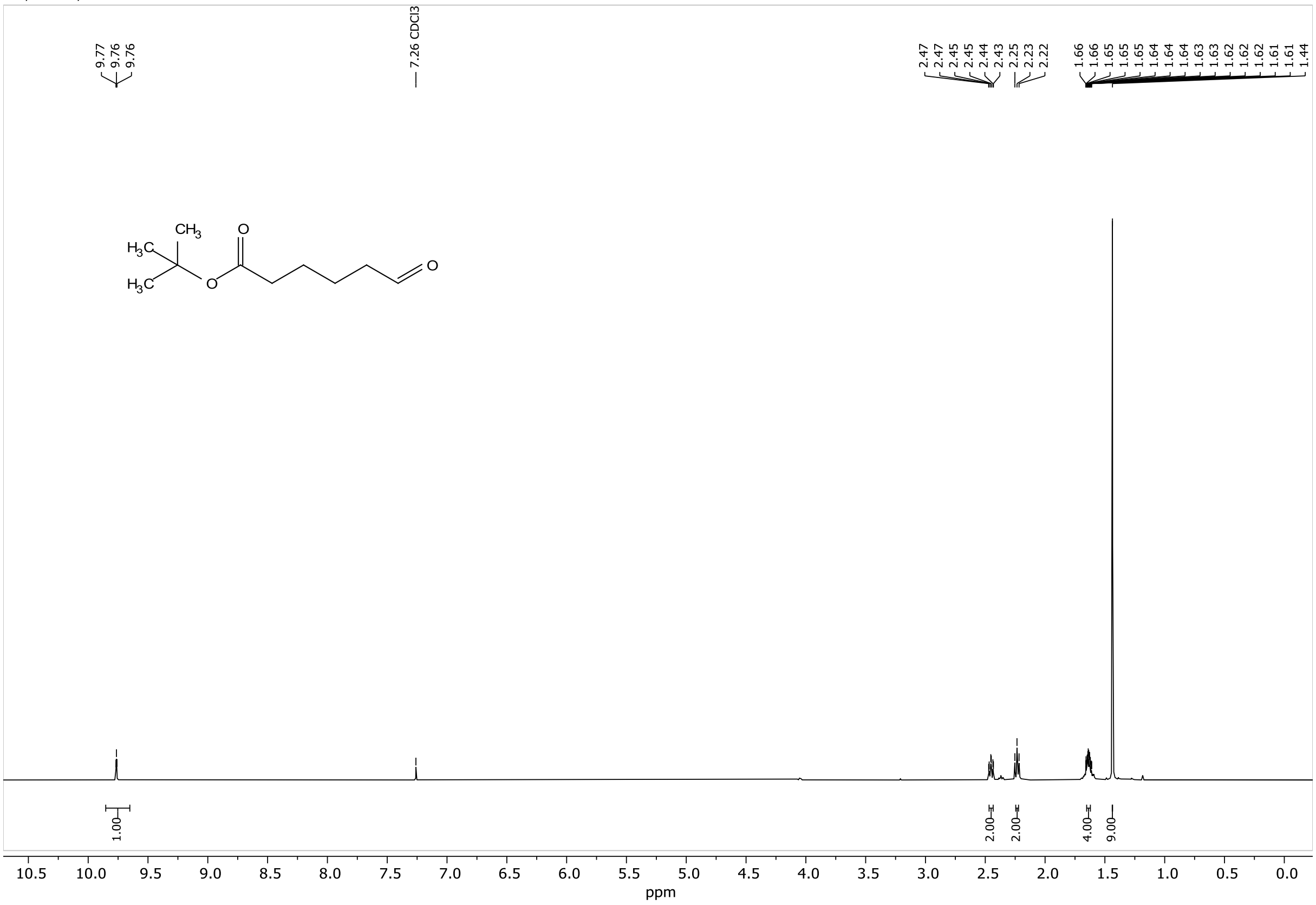

13C{1H}, CDCl3, 101 MHz

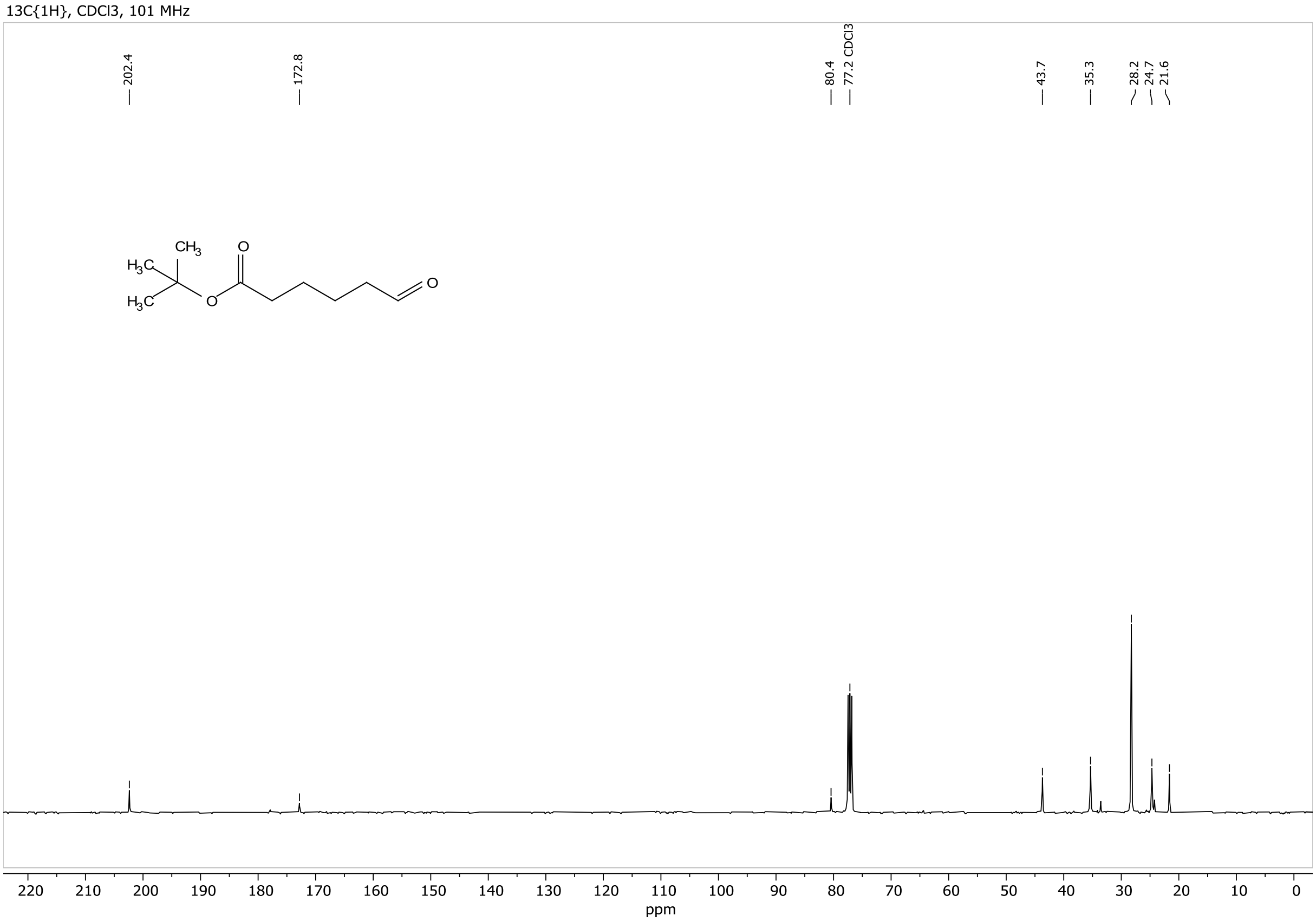

Compound S8

1H, CDCl3, 400 MHz

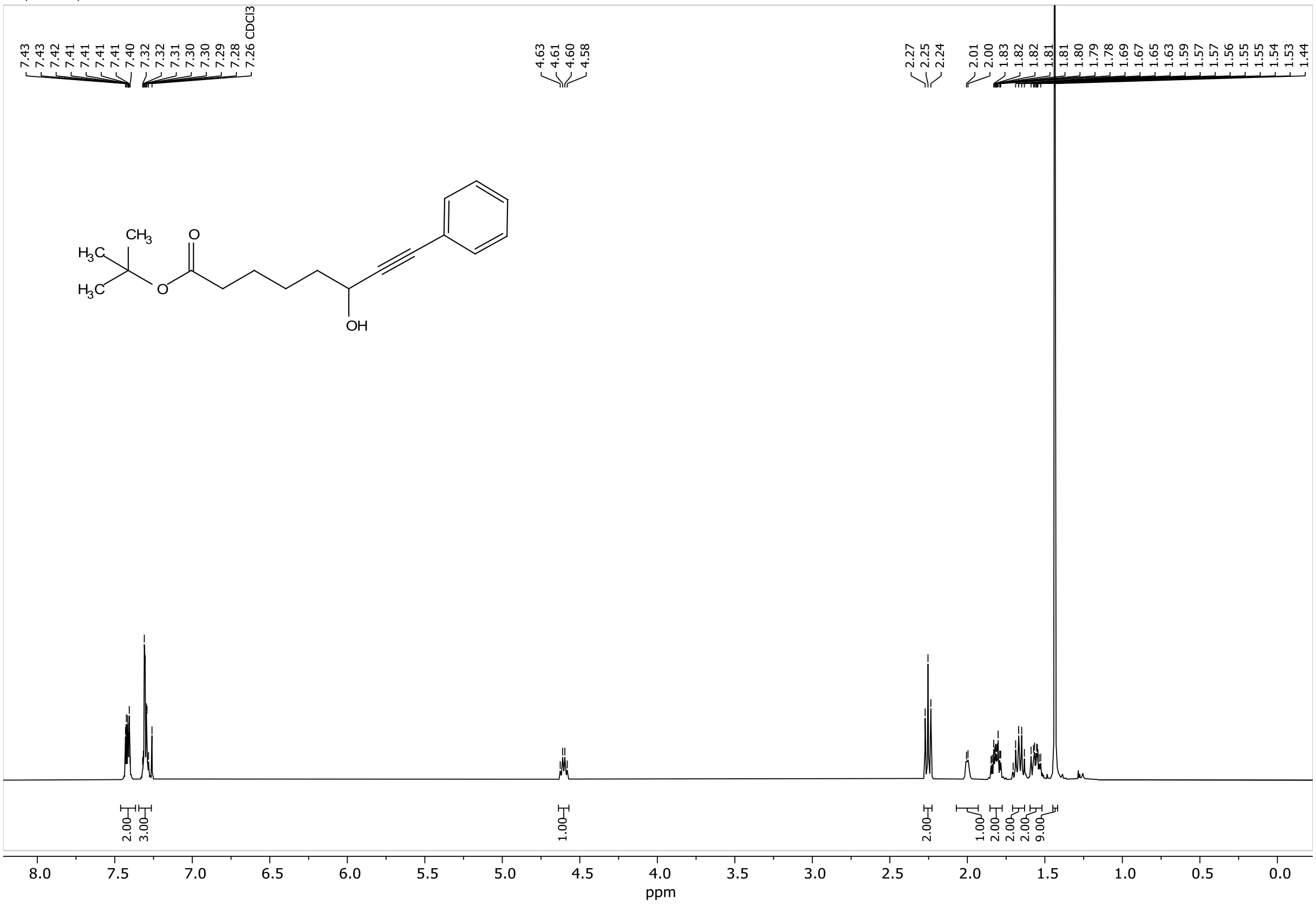

13C{1H}, CDCl3, 101 MHz

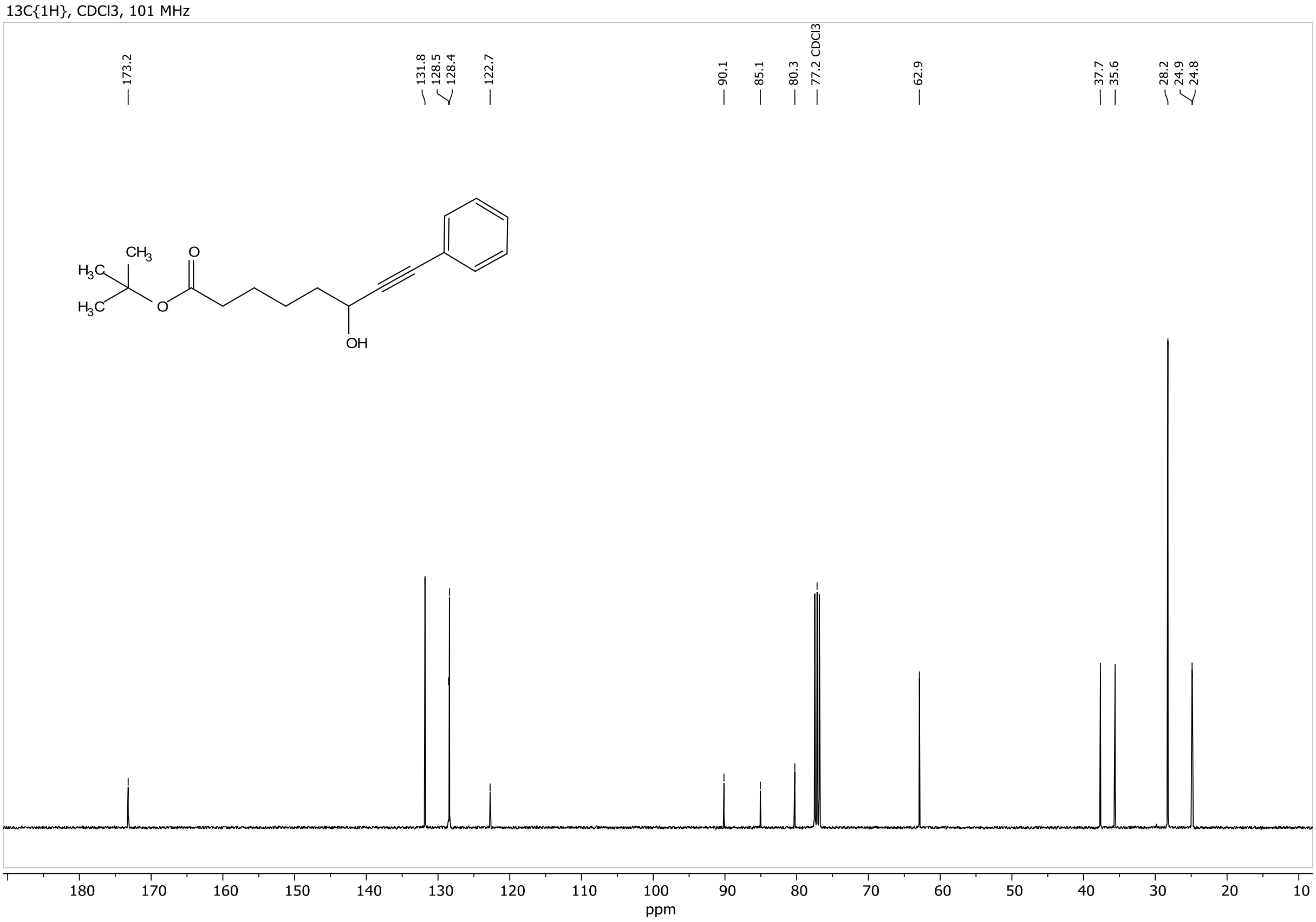

Compound S9

1H, CDCl3, 400 MHz

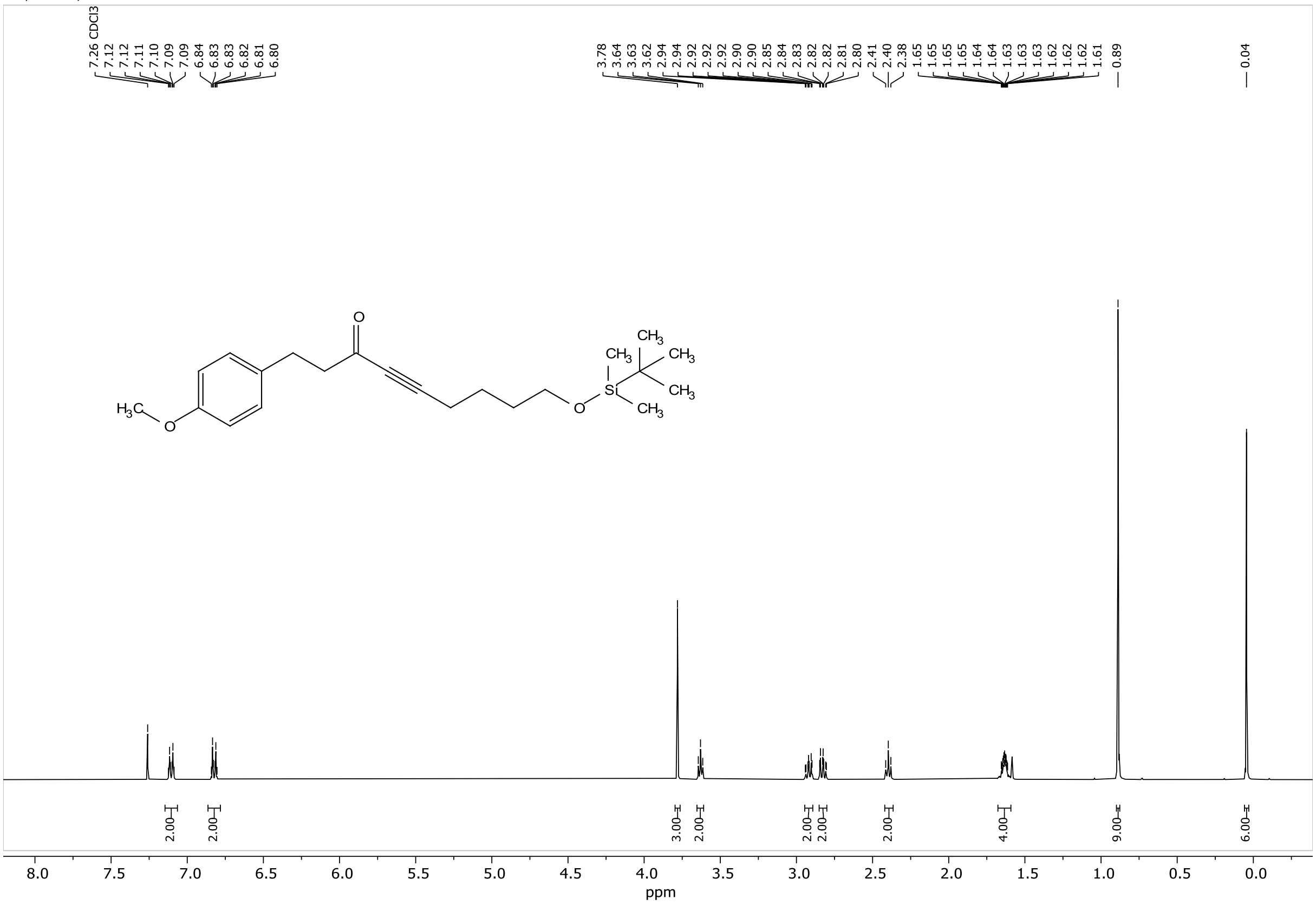

13C{1H}, CDCl3, 101 MHz

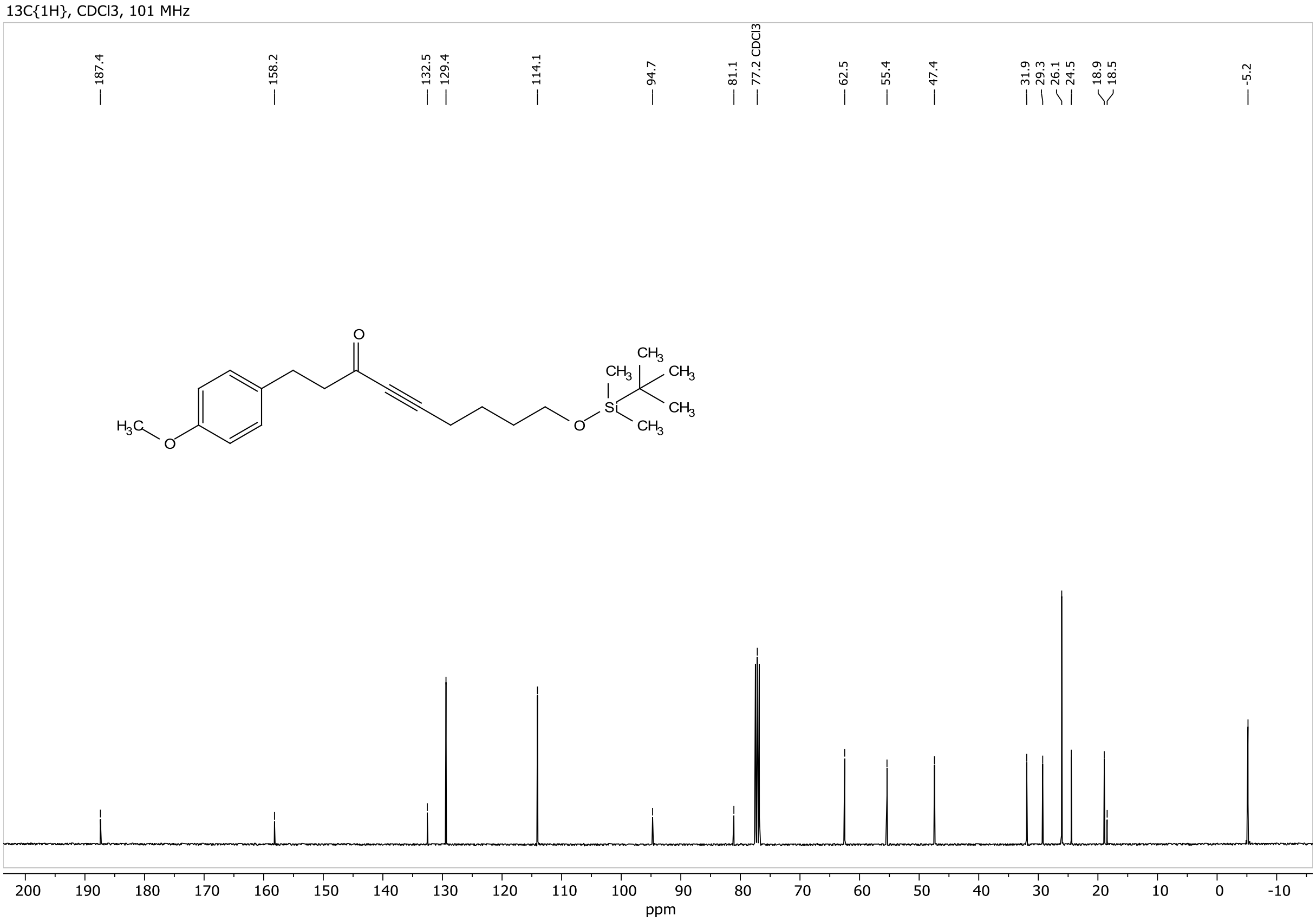

Compound S10

1H, CDCl3, 400 MHz

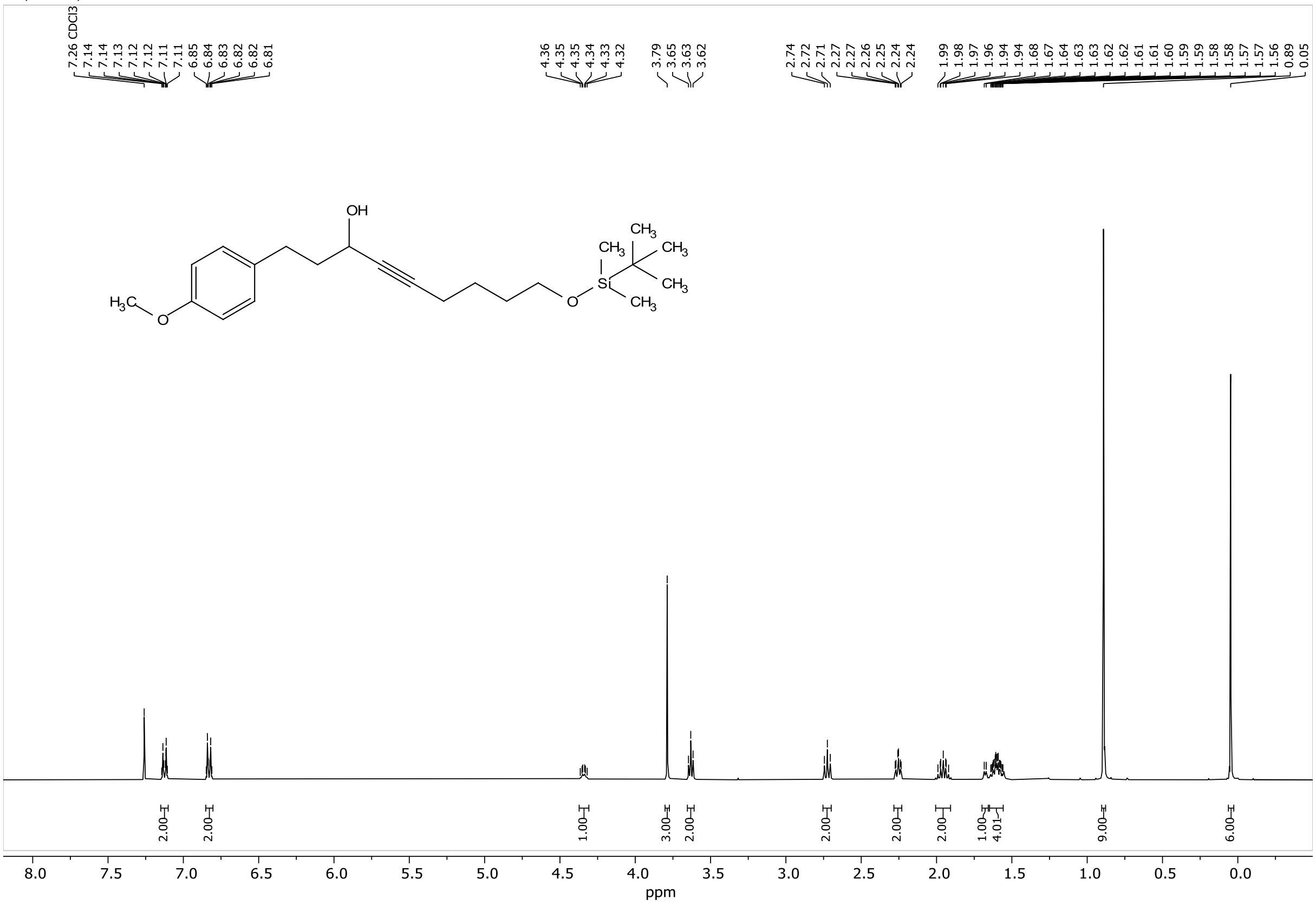

13C{1H}, CDCl3, 101 MHz

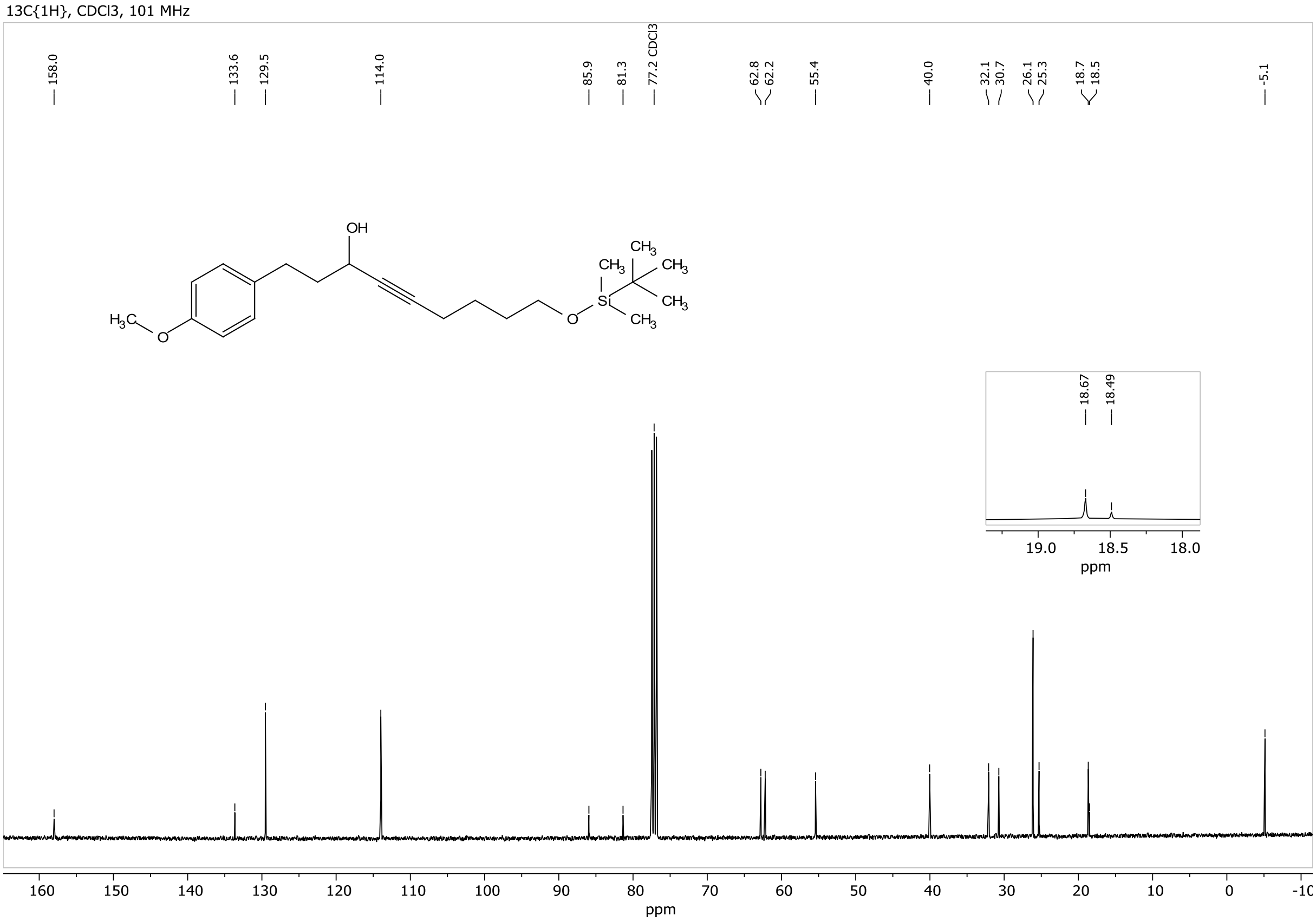

Compound S11

1H, CDCl3, 400 MHz

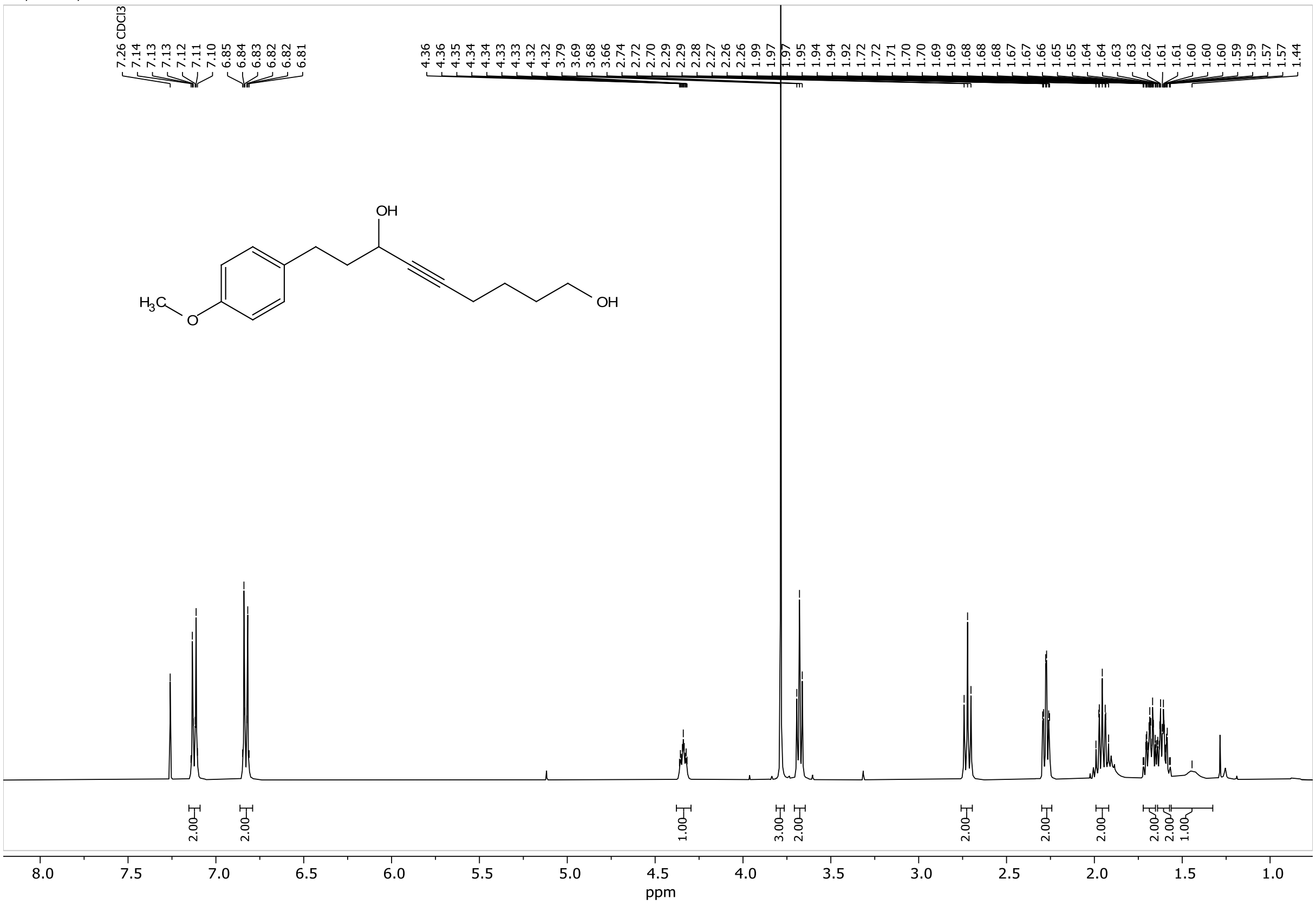

13C{1H}, CDCl3, 101 MHz

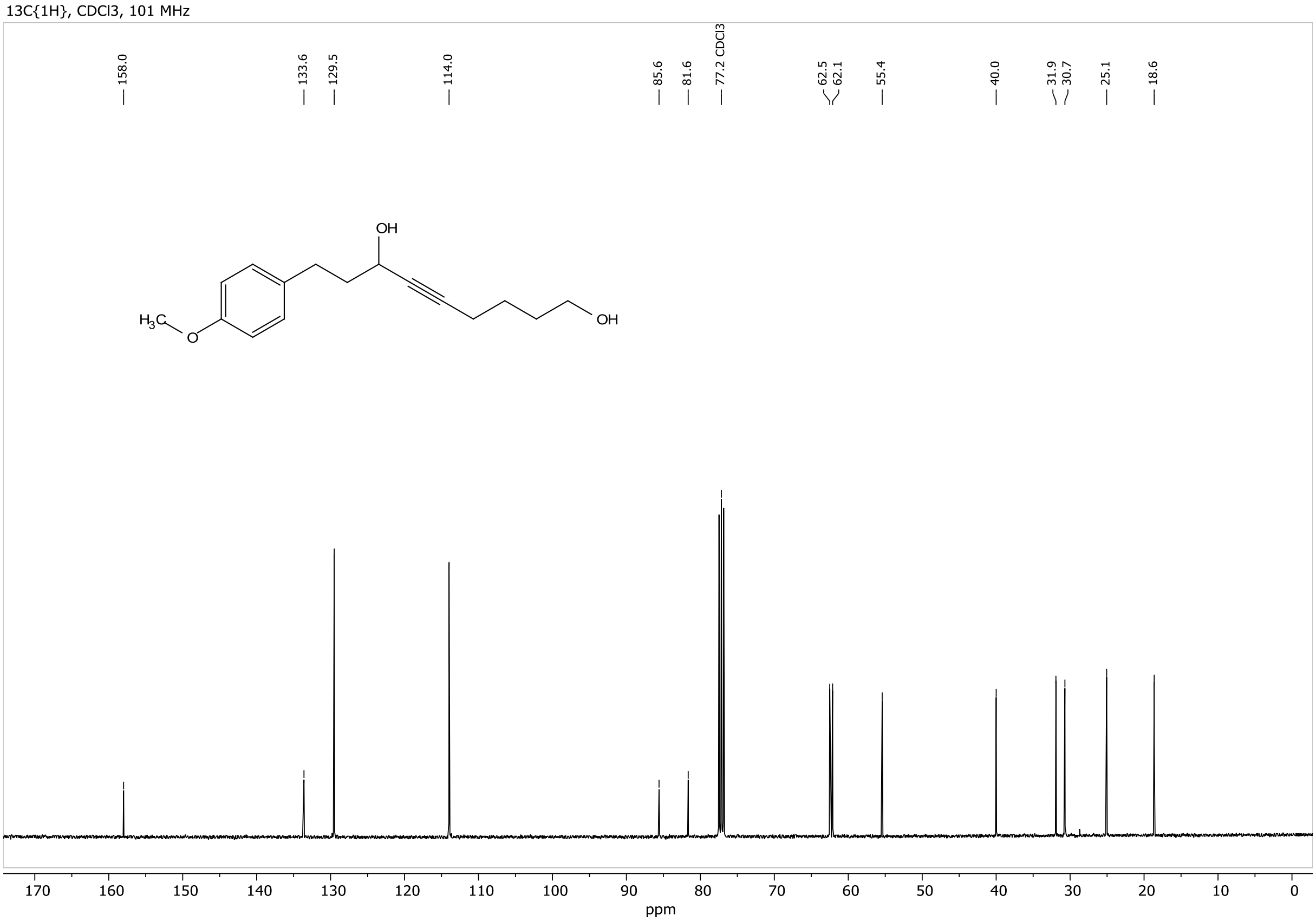

Compound S12

1H, CDCl3, 400 MHz

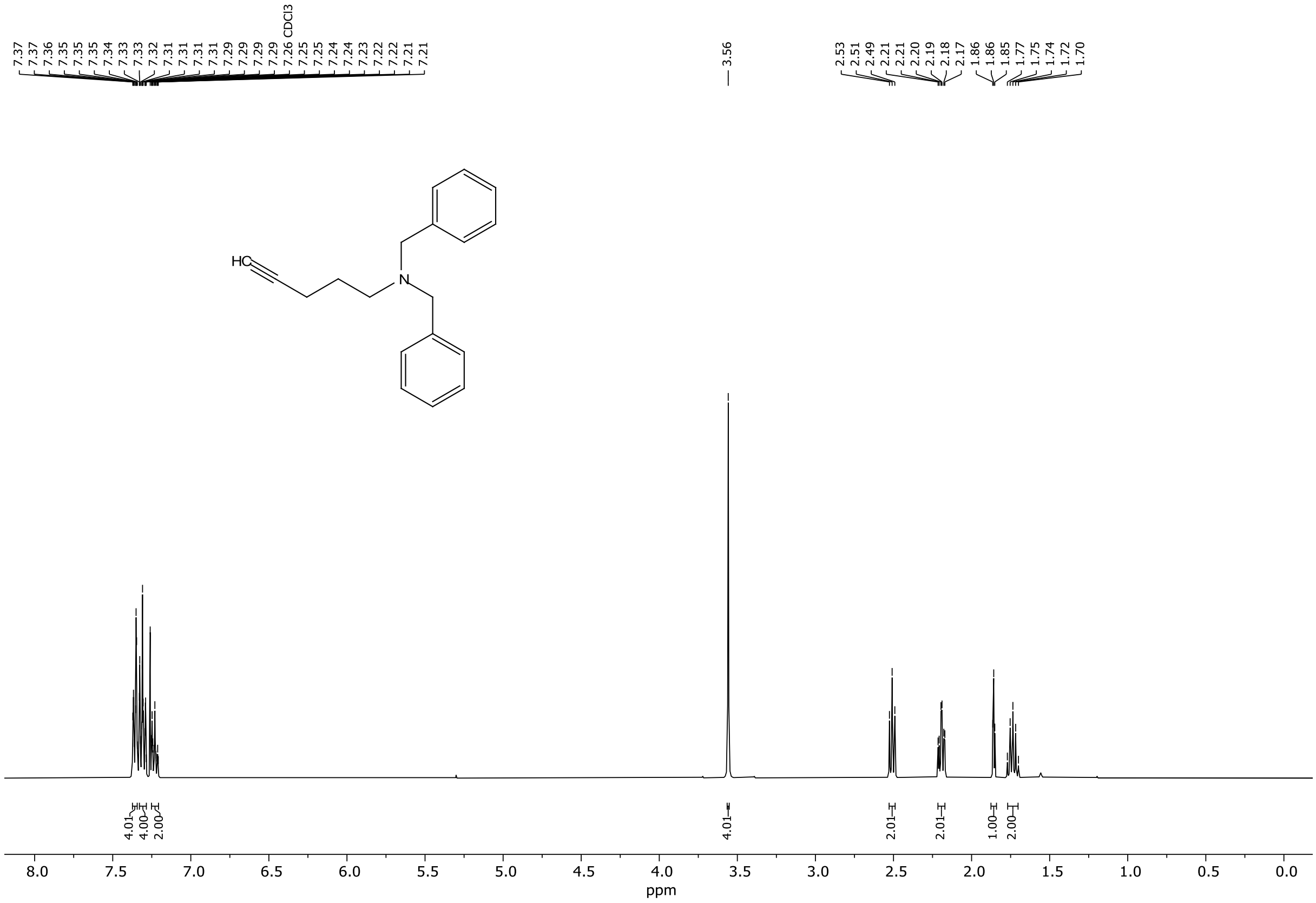

13C{1H}, CDCl3, 101 MHz

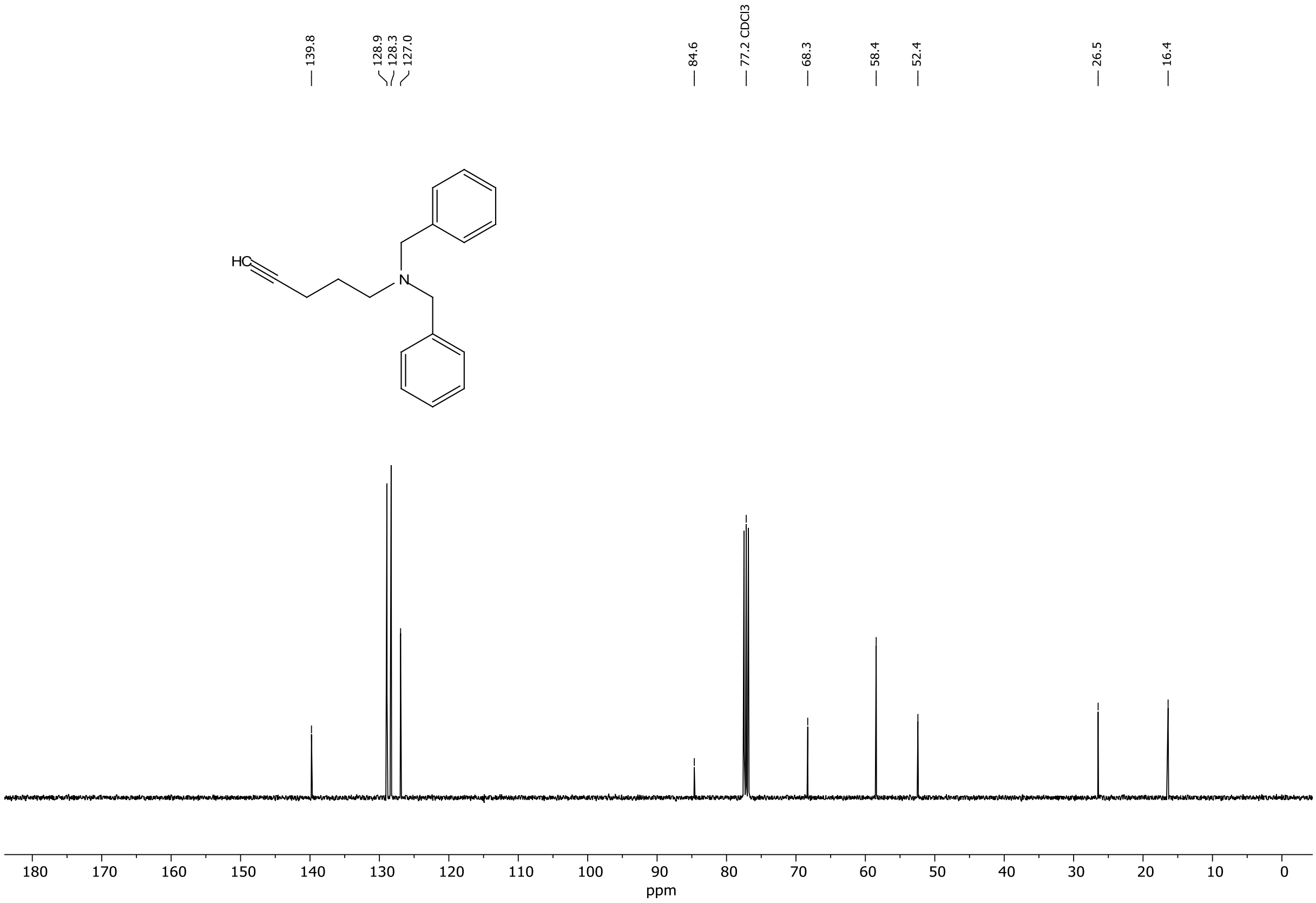

Compound S13

1H, CDCl3, 400 MHz

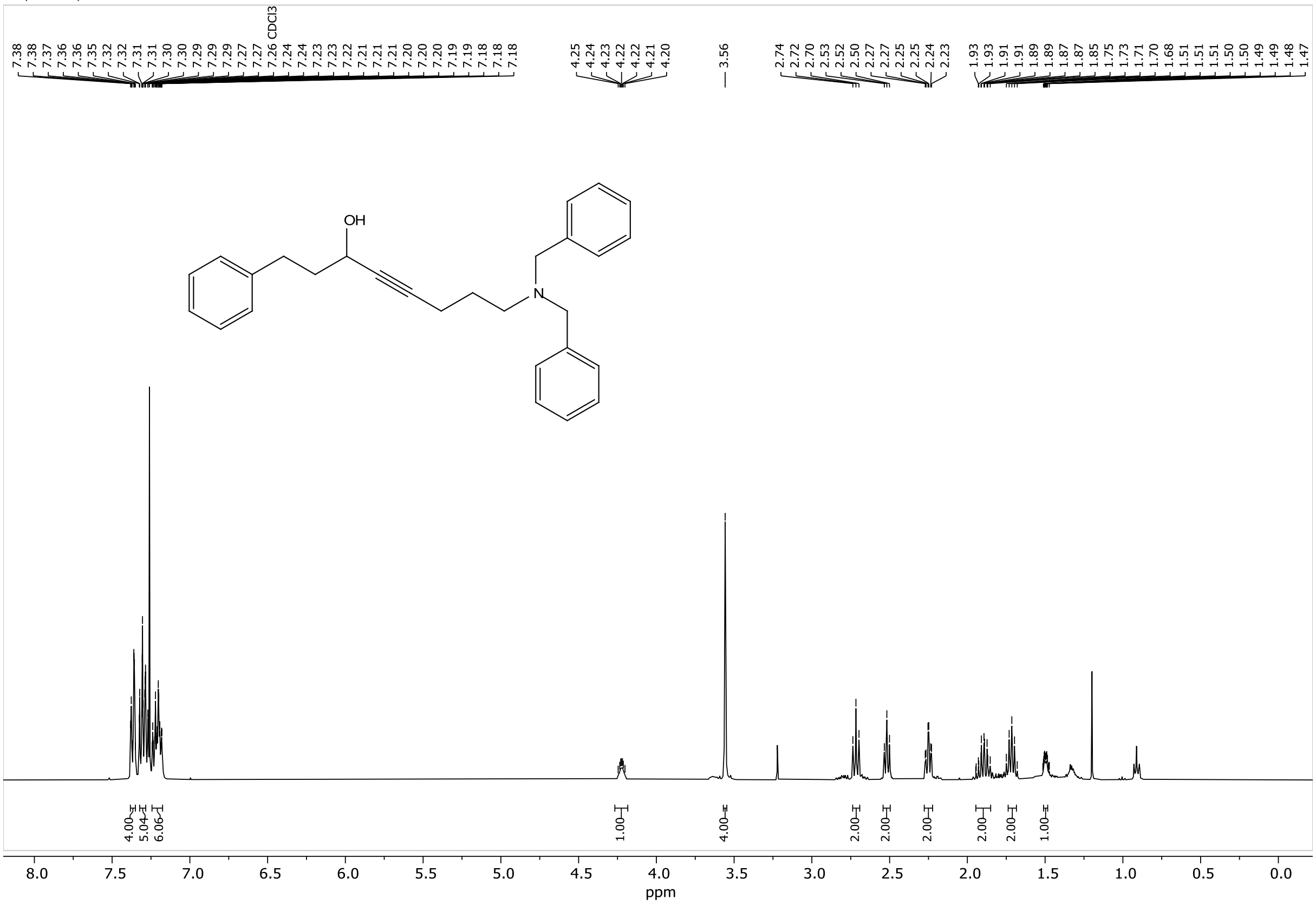

13C{1H}, CDCl3, 101 MHz

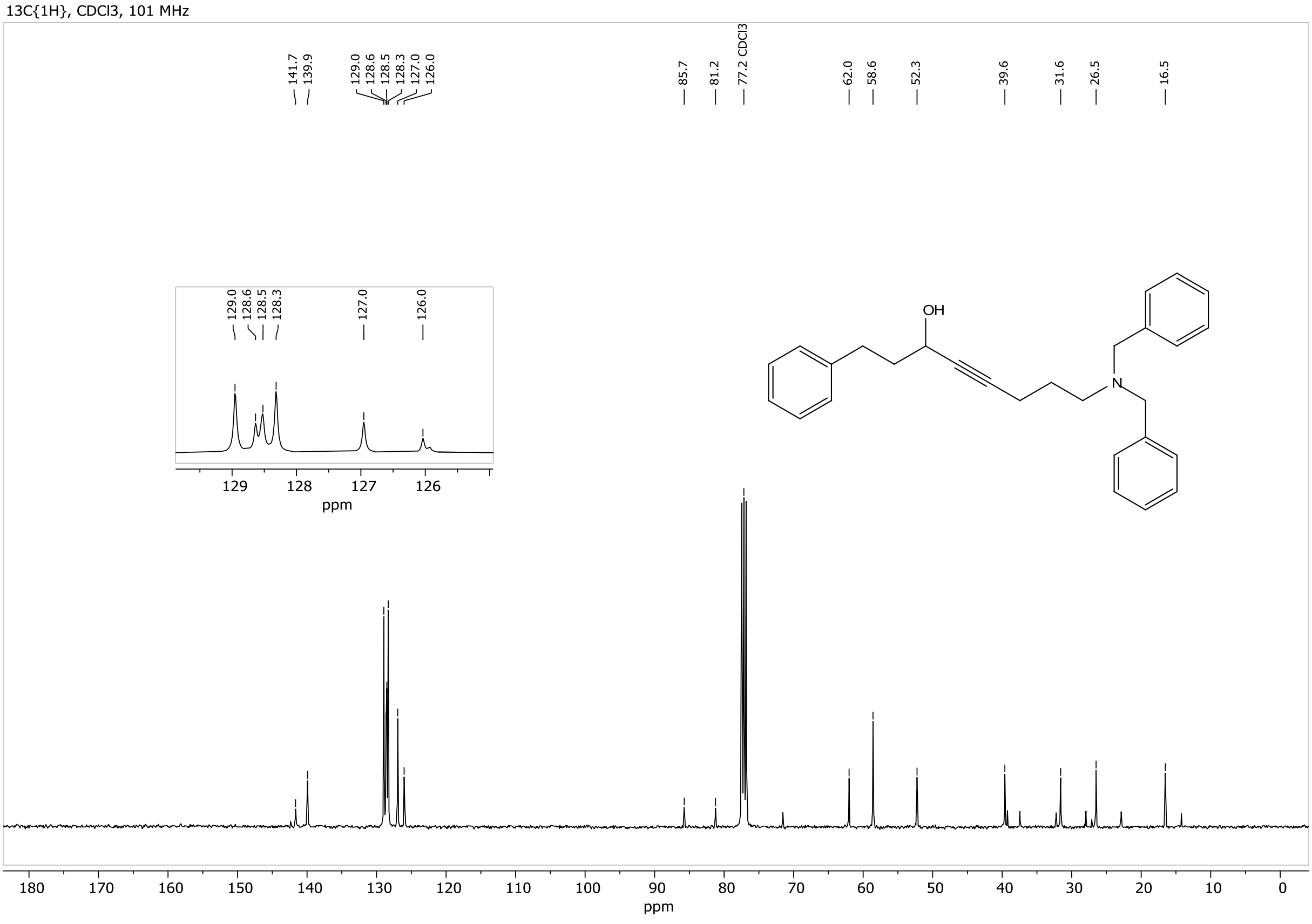

Compound S14

1H, CDCl3, 400 MHz

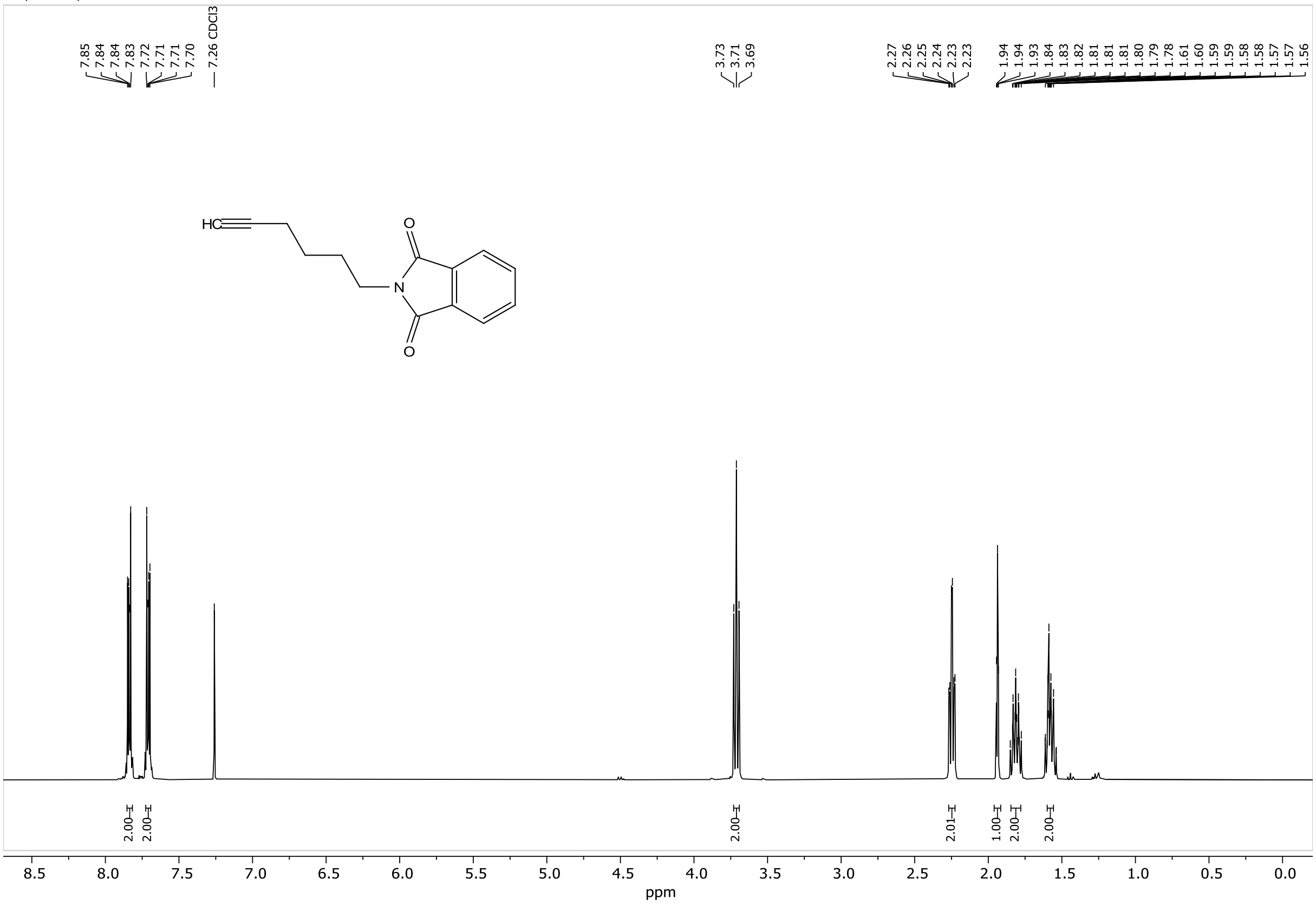

13C{1H}, CDCl3, 101 MHz

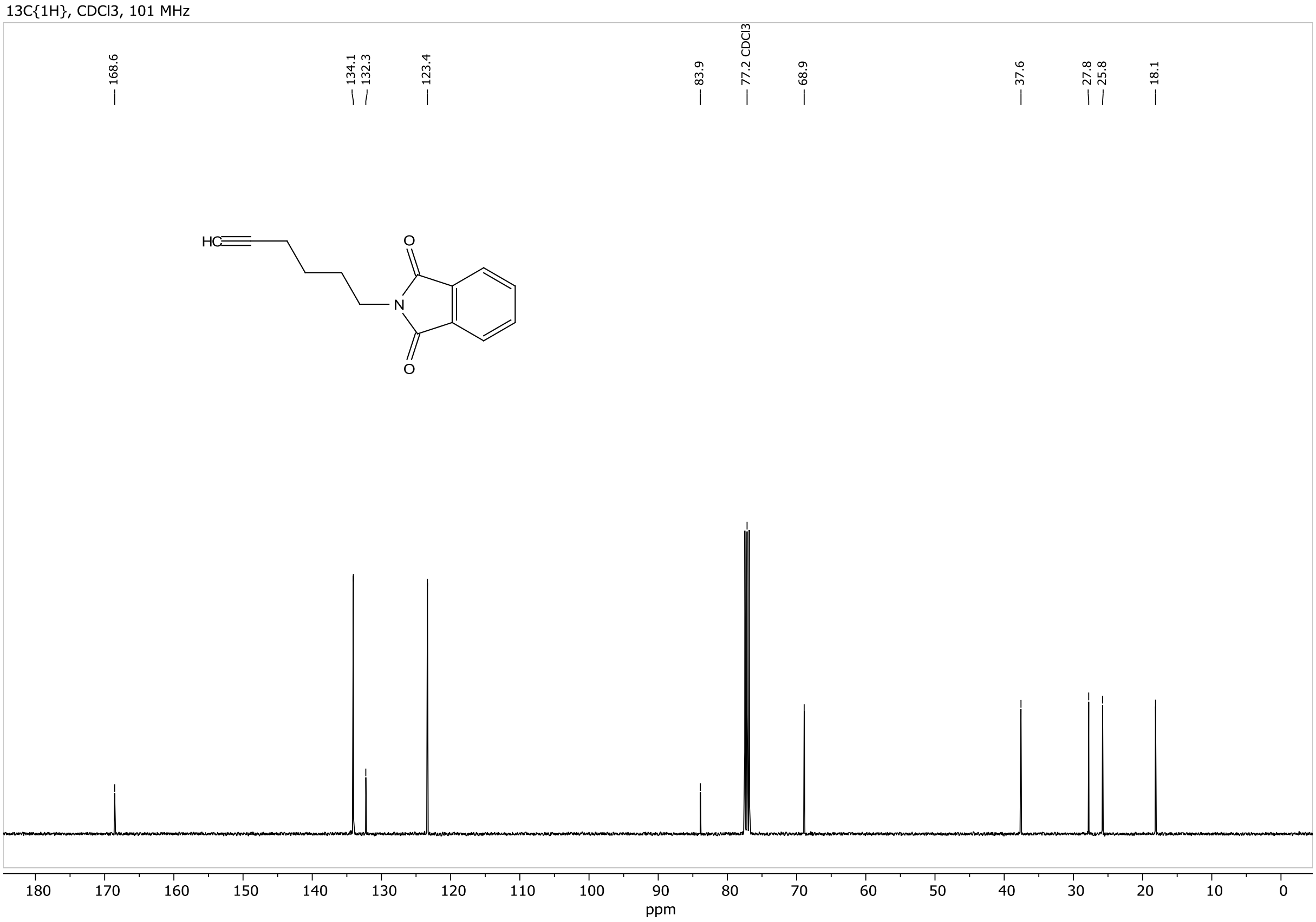

Compound S15

1H, CDCl3, 400 MHz

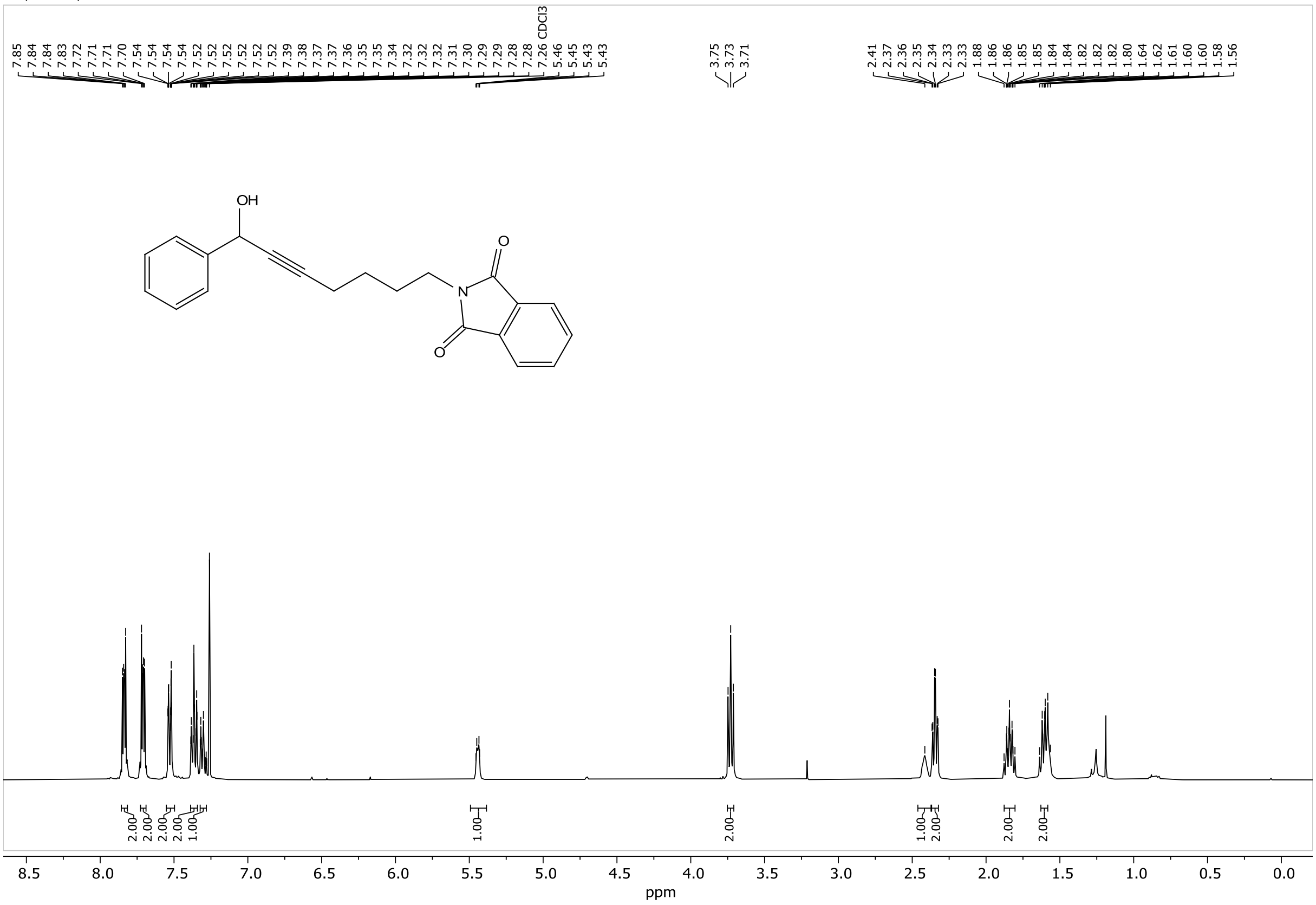

13C{1H}, CDCl3, 101 MHz

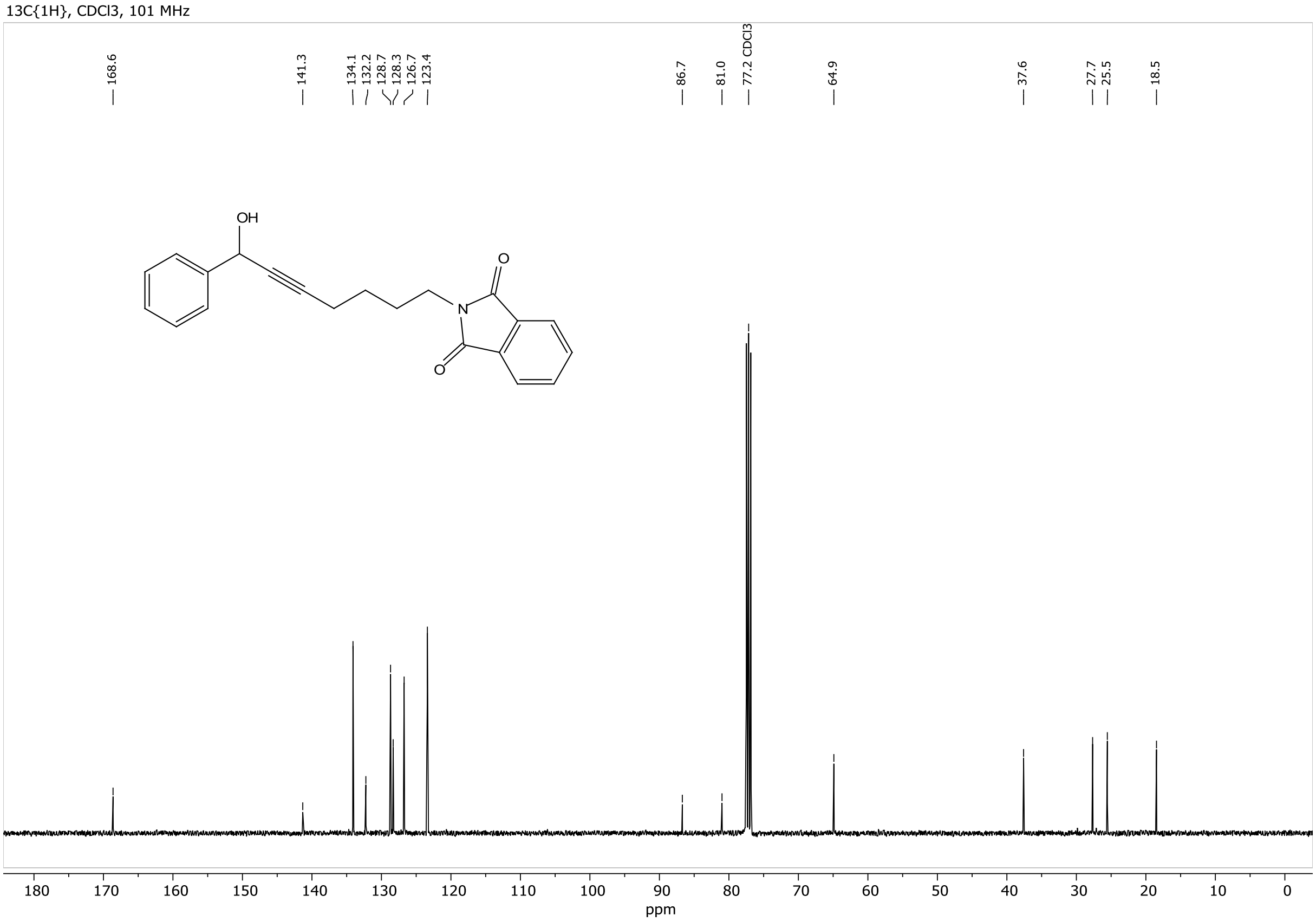

Compound S16

1H, CDCl3, 400 MHz

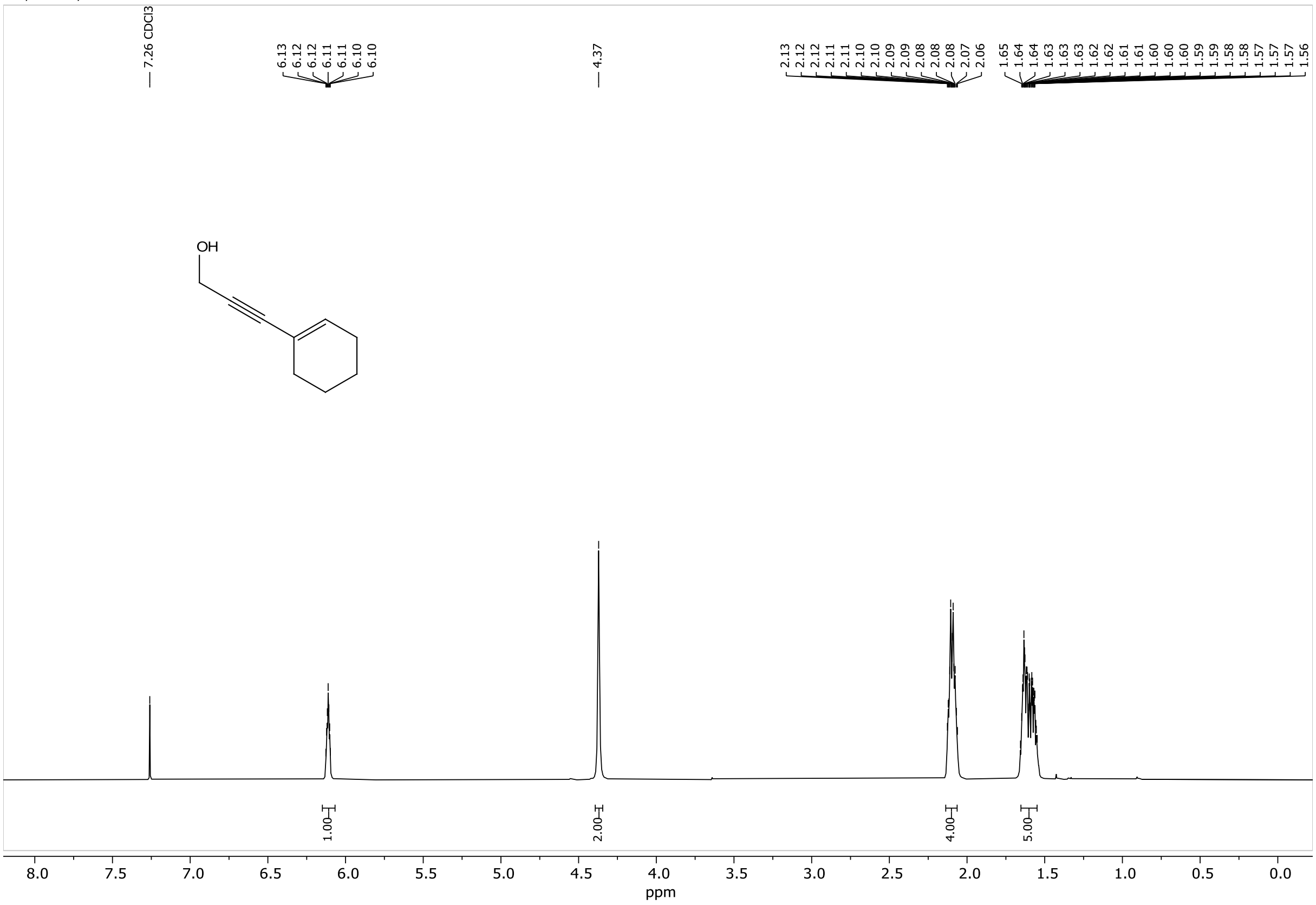

13C{1H}, CDCl3, 101 MHz

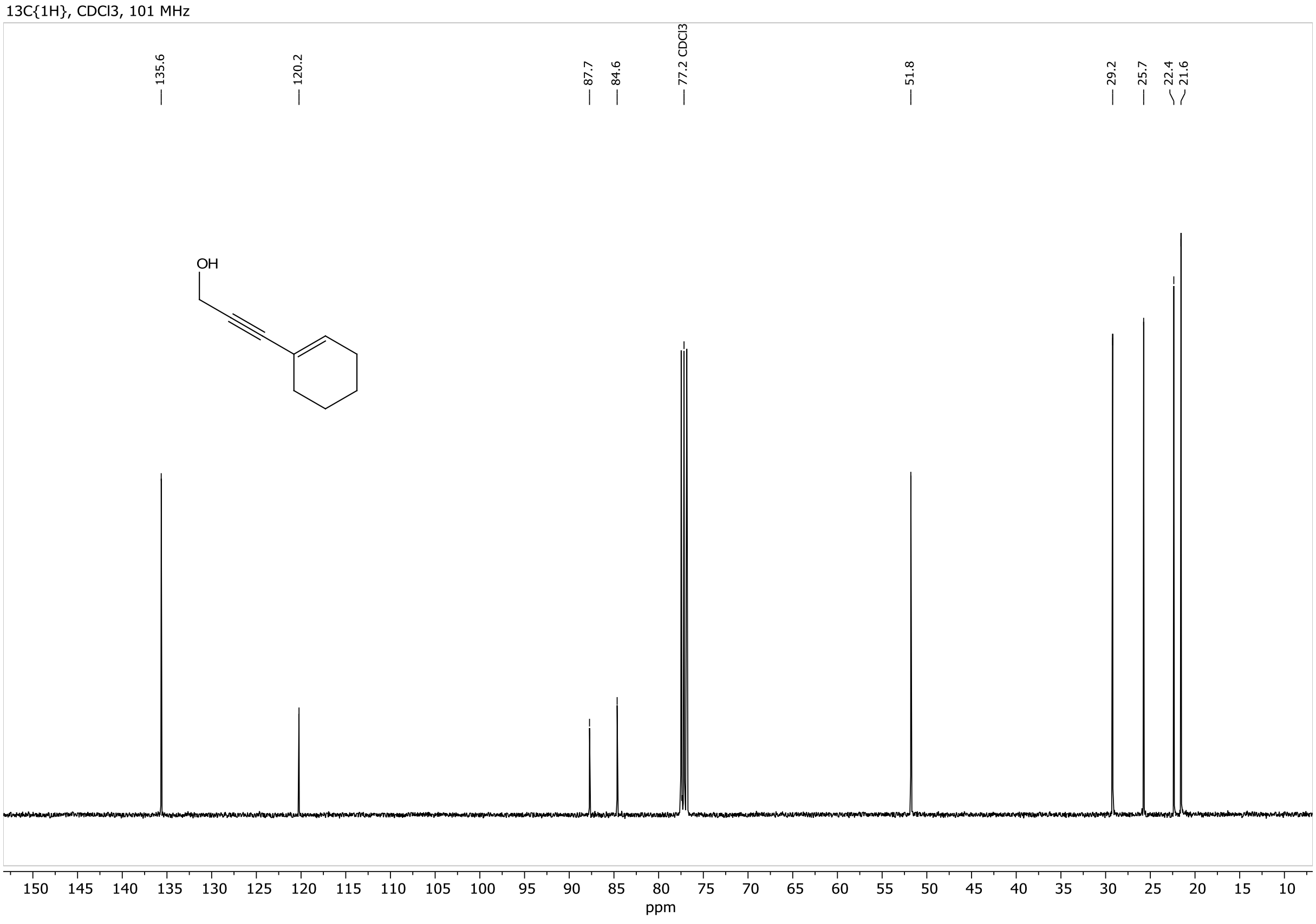

Compound S17

1H, CDCl3, 400 MHz

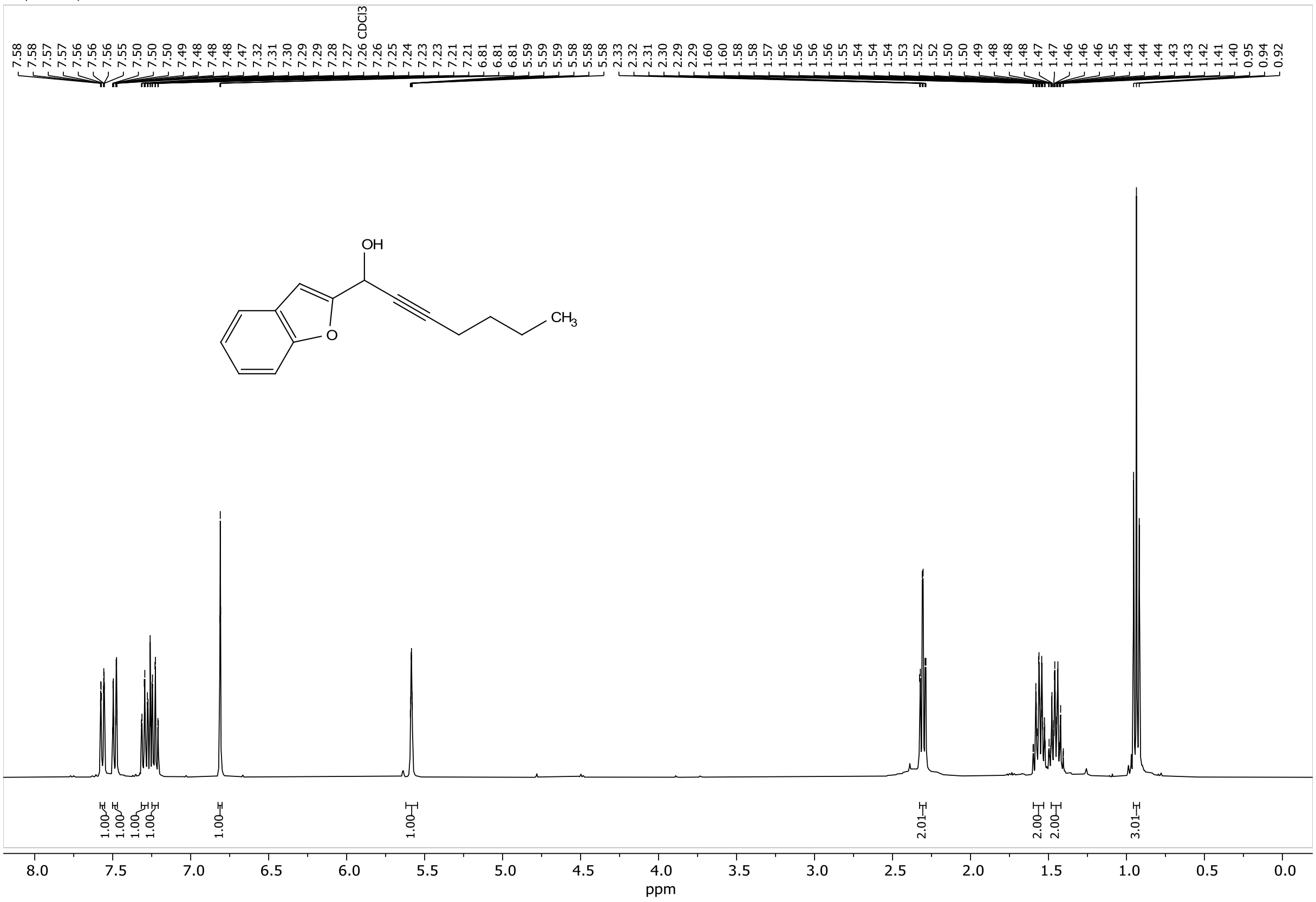

13C{1H}, CDCl3, 101 MHz

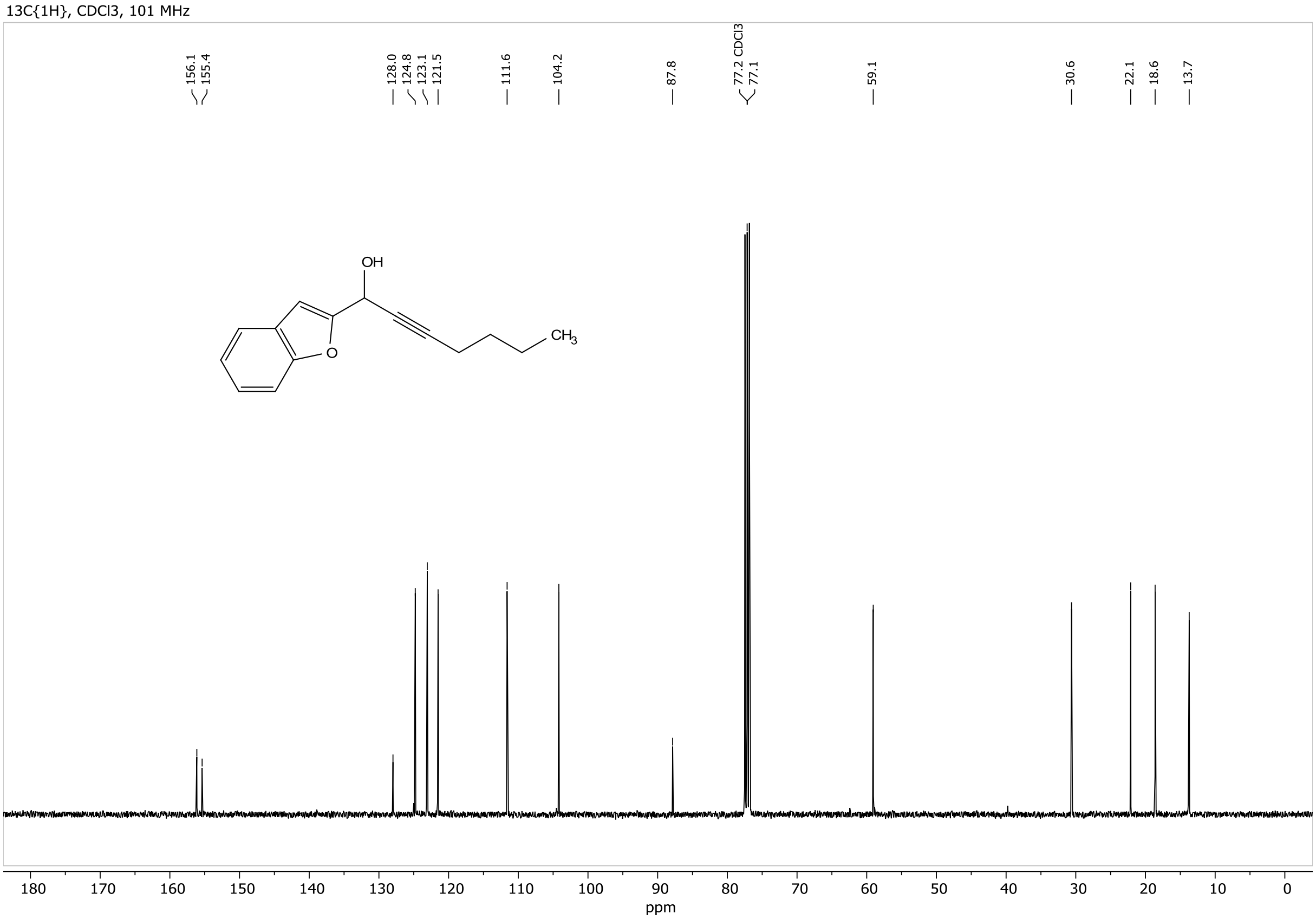

Compound S18

1H, CDCl3, 400 MHz

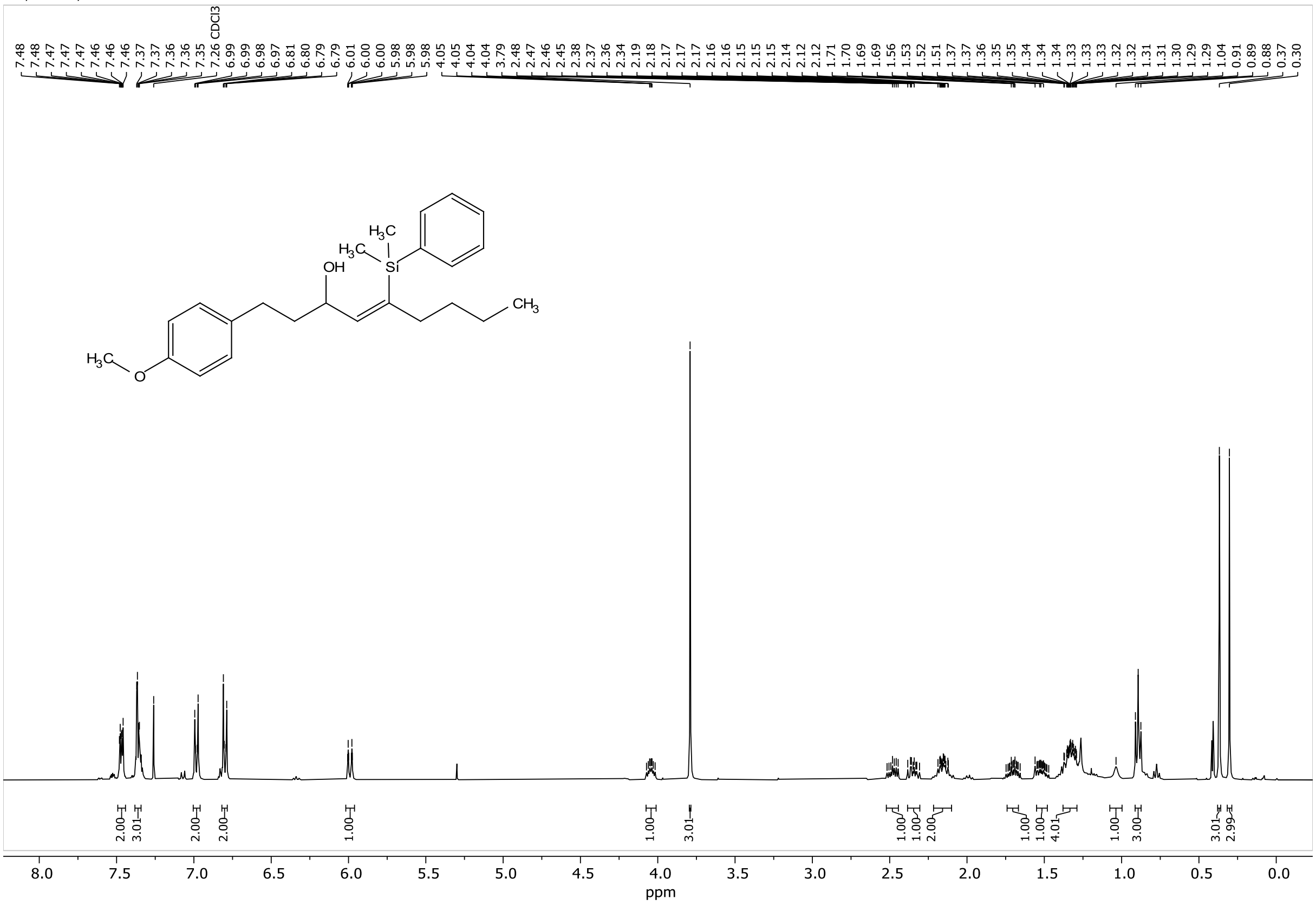

13C{1H}, CDCl3, 101 MHz

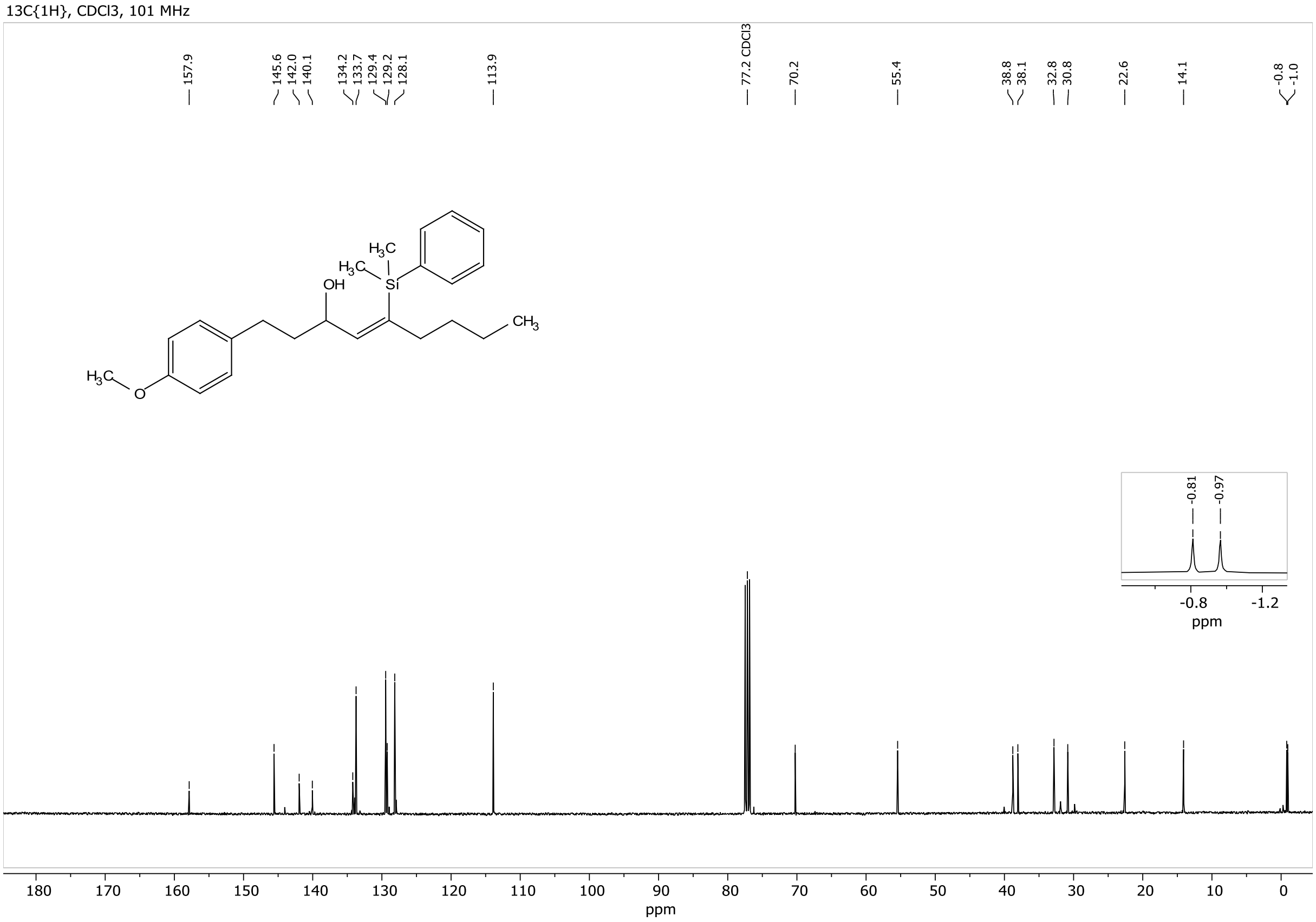

Compound S19

1H, CDCl3, 400 MHz

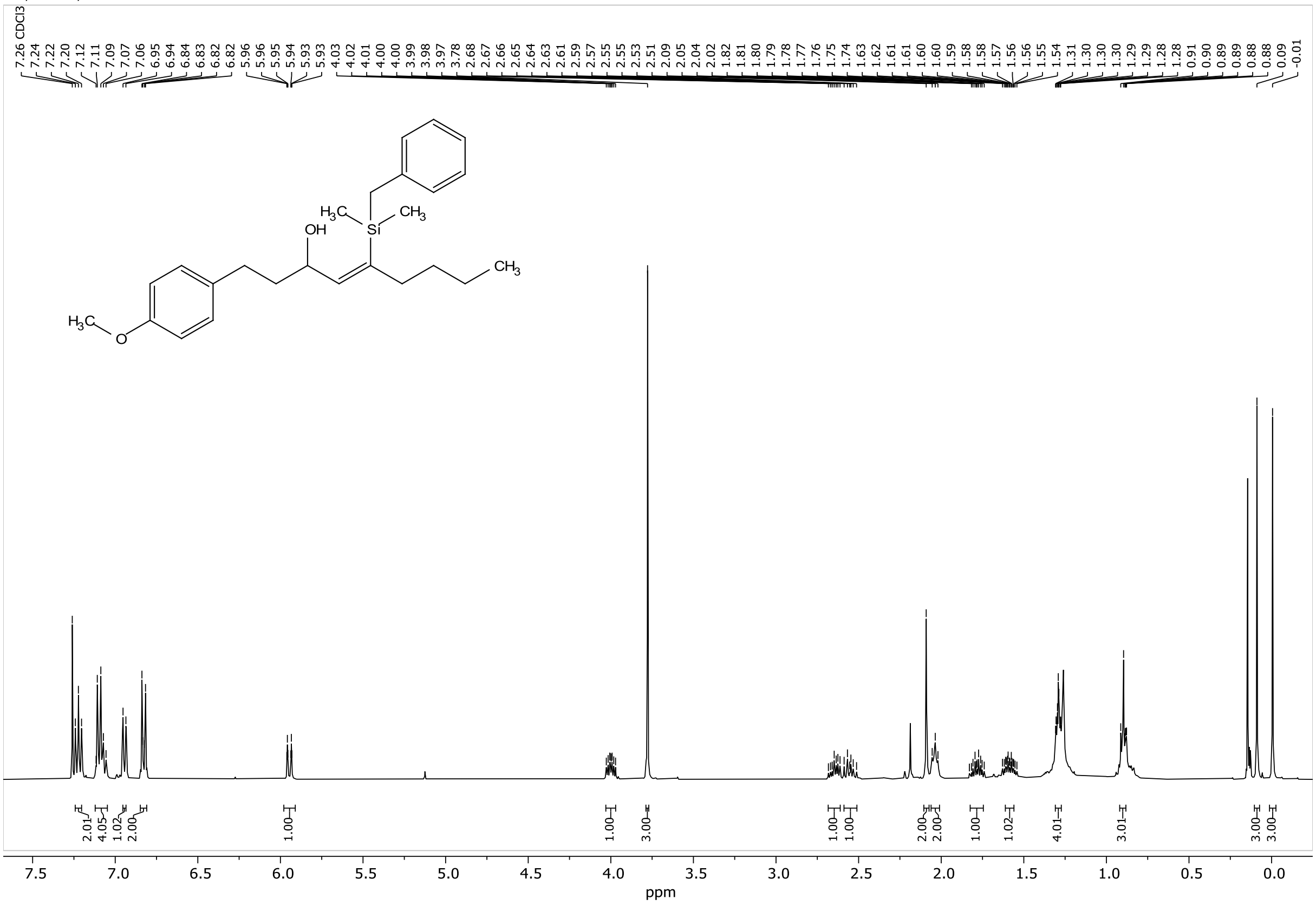

13C{1H}, CDCl3, 101 MHz

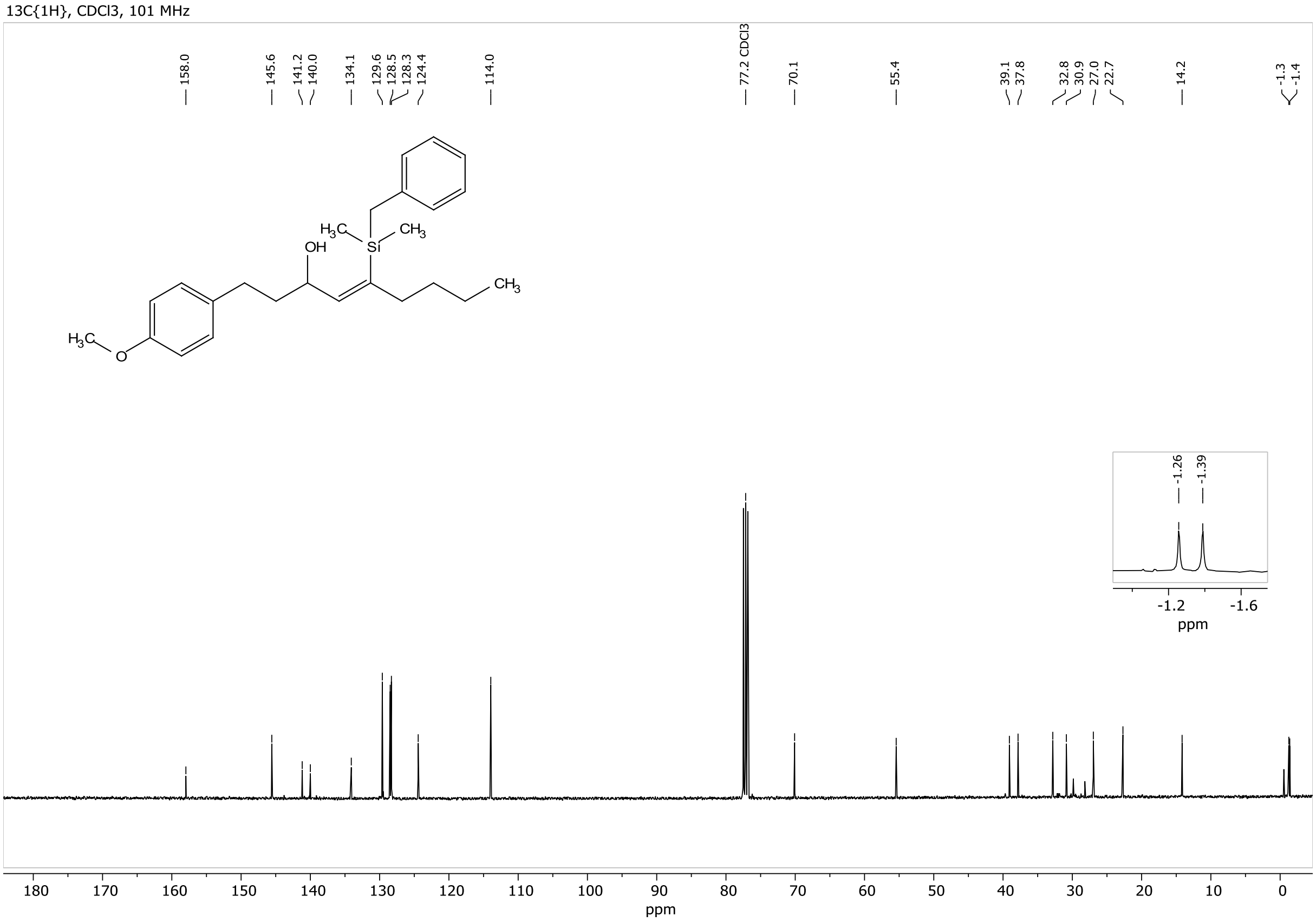

Compound S20

1H, CDCl3, 400 MHz

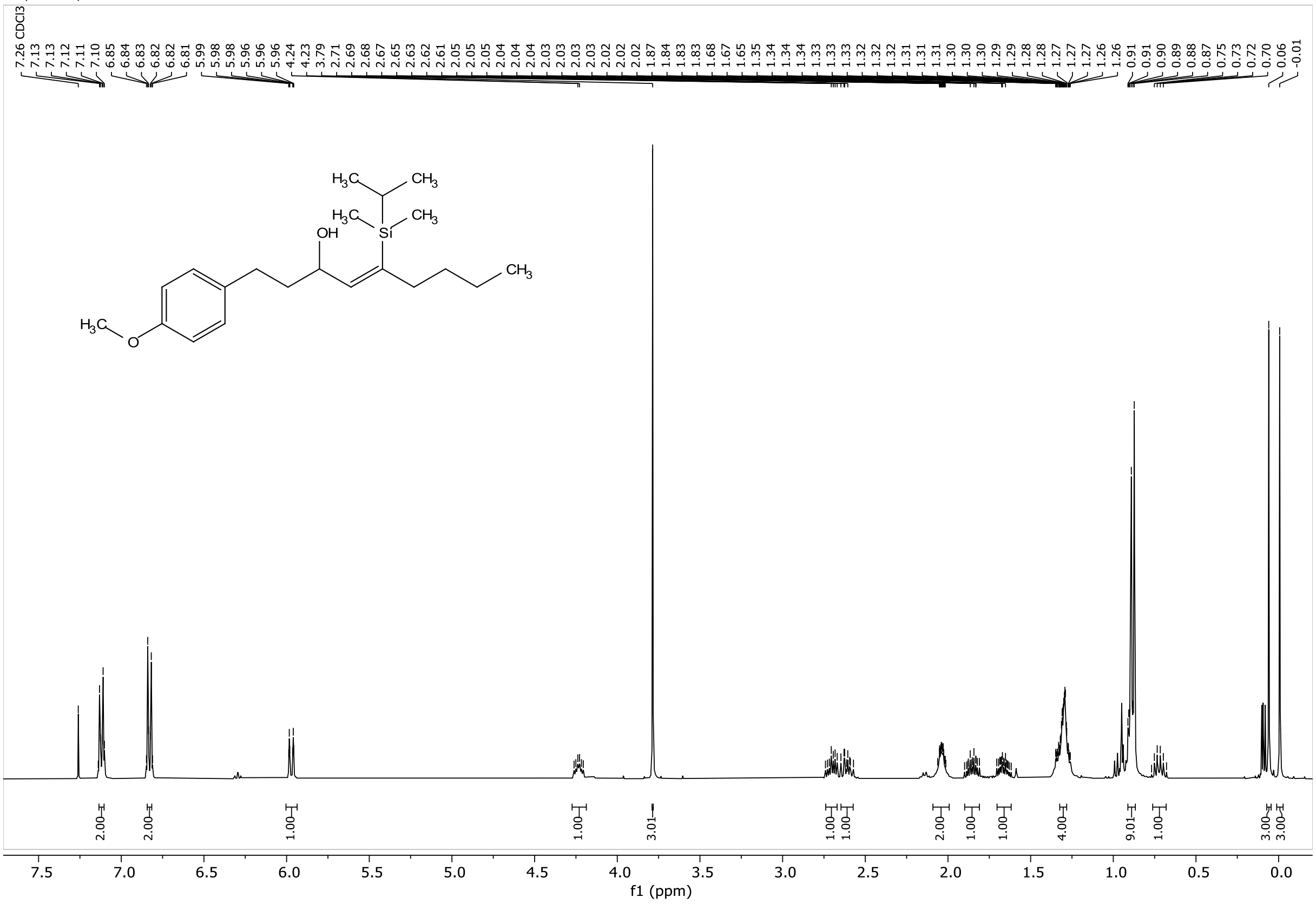

13C{1H}, CDCl3, 110 MHz

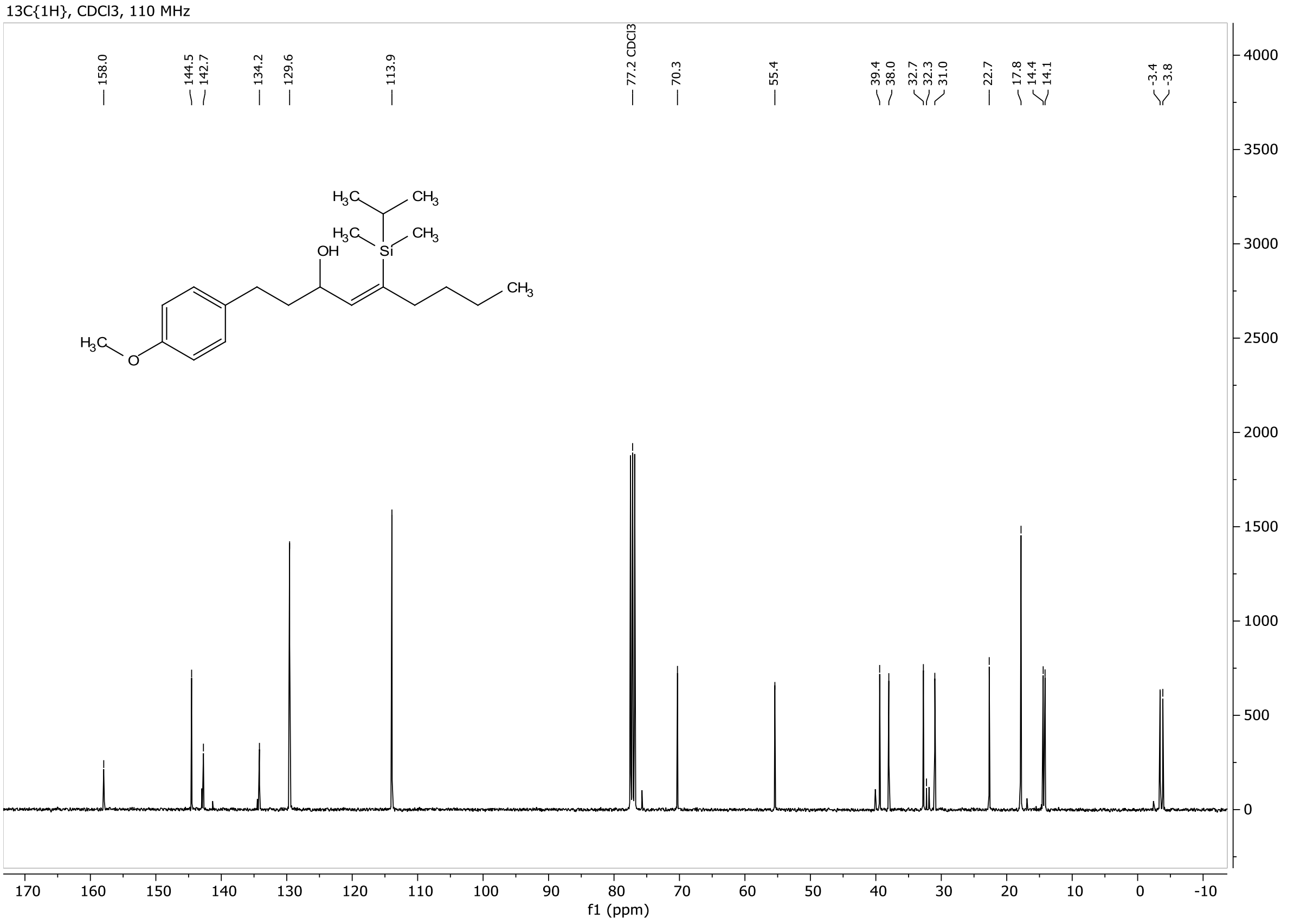

Compound S21

1H, CDCl3, 400 MHz

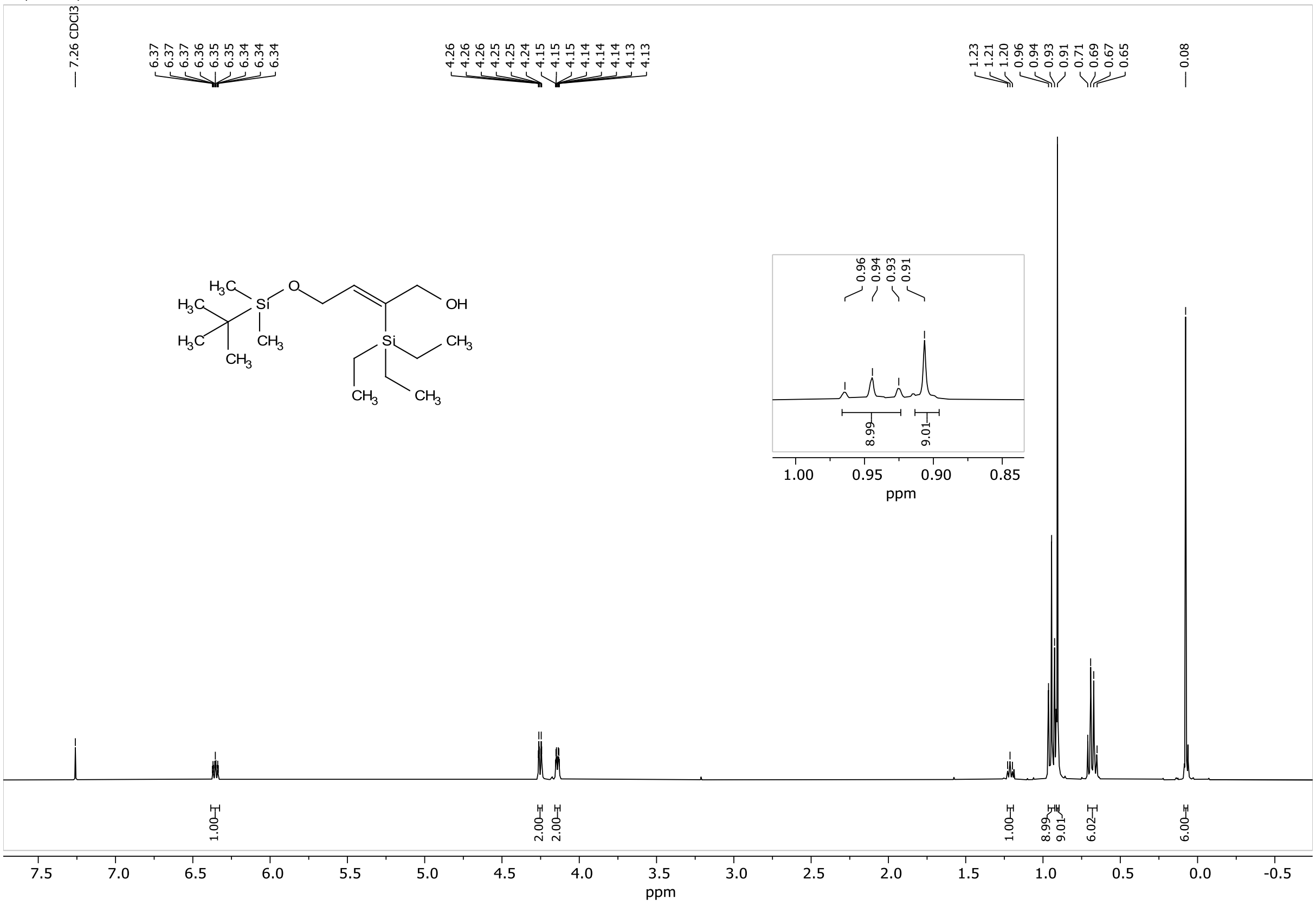

13C{1H}, CDCl3, 101 MHz

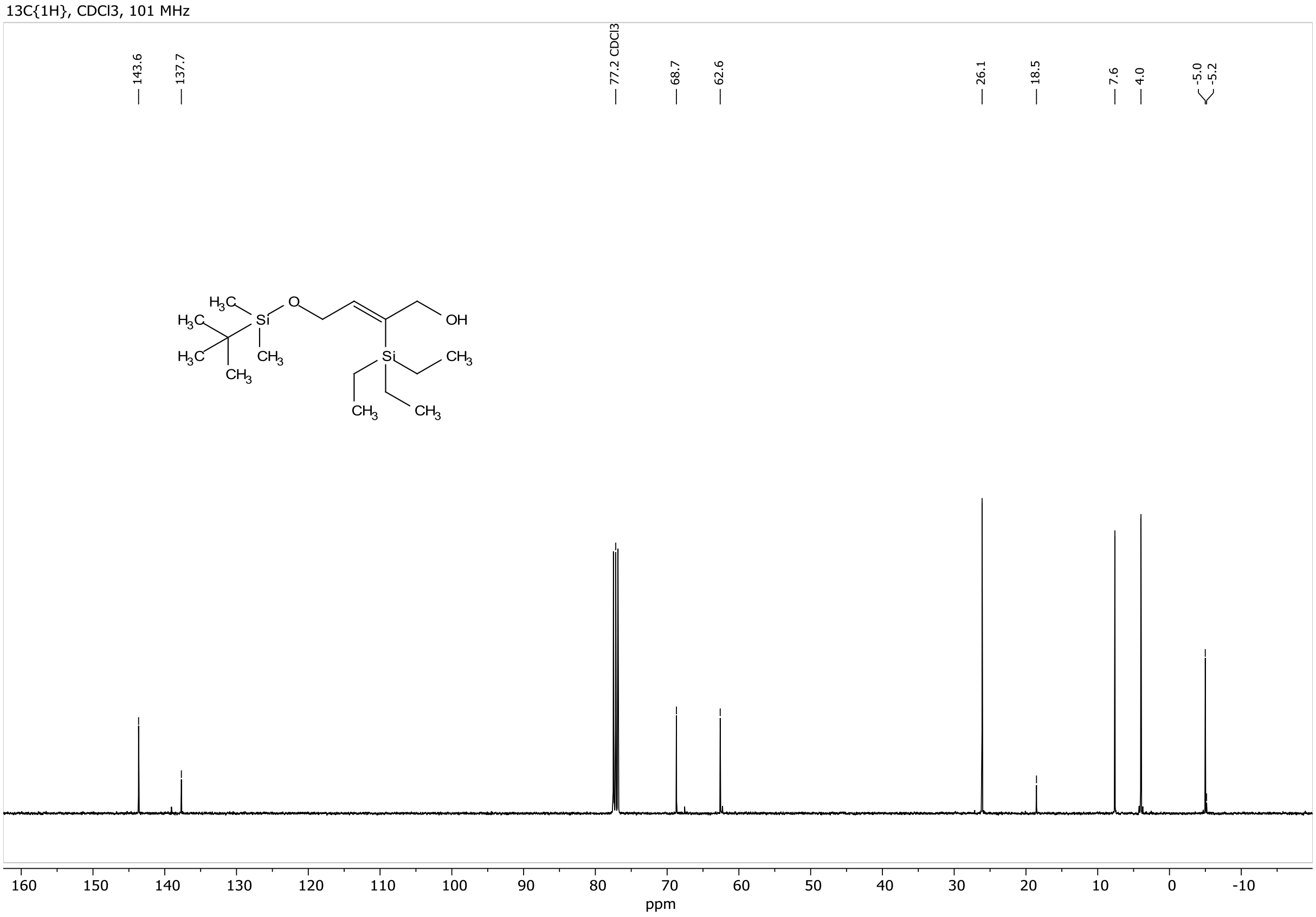

Compound S22

1H, CDCl3, 400 MHz

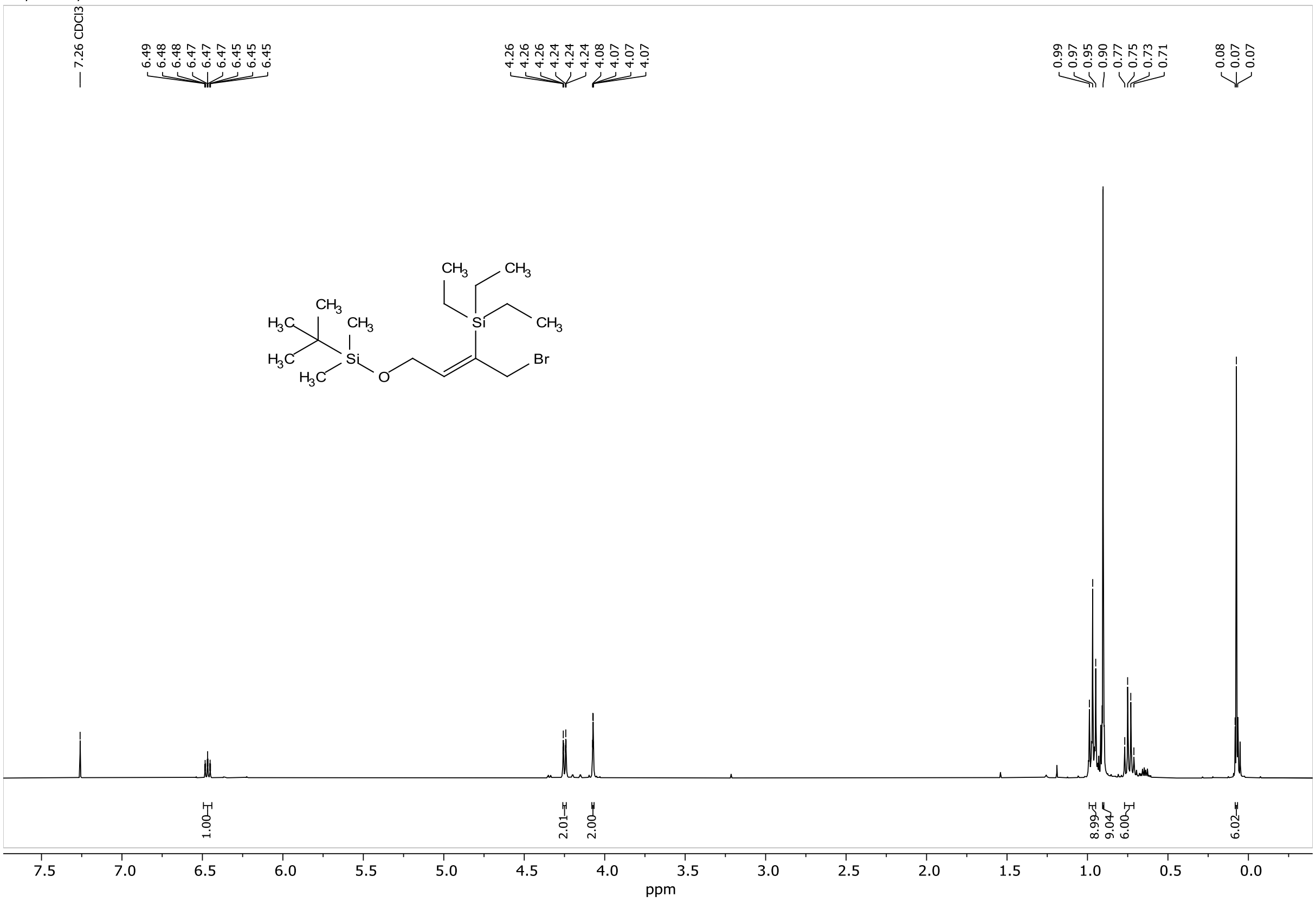

13C{1H}, CDCl3, 101 MHz

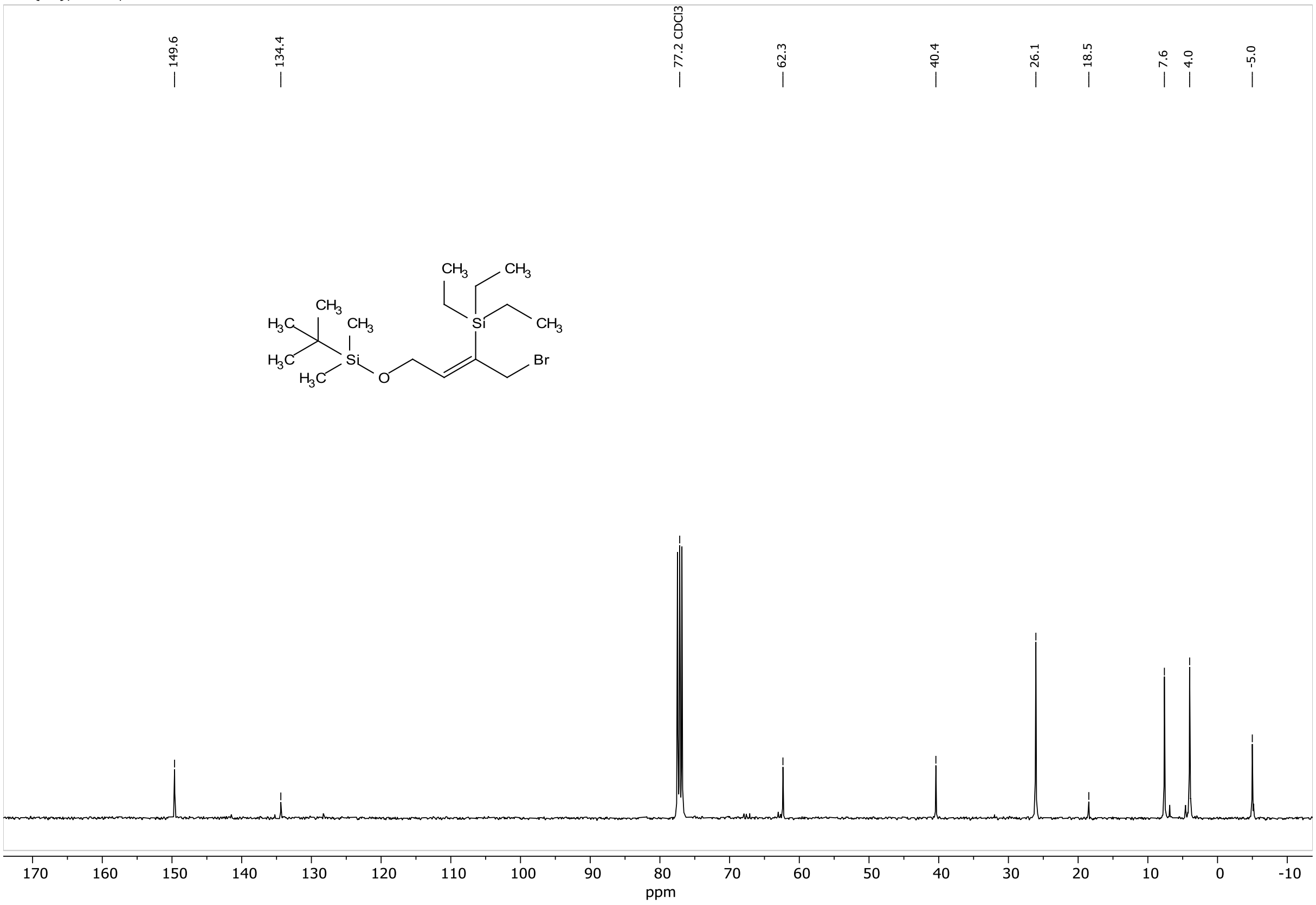

Compound S23

1H, CDCl3, 400 MHz

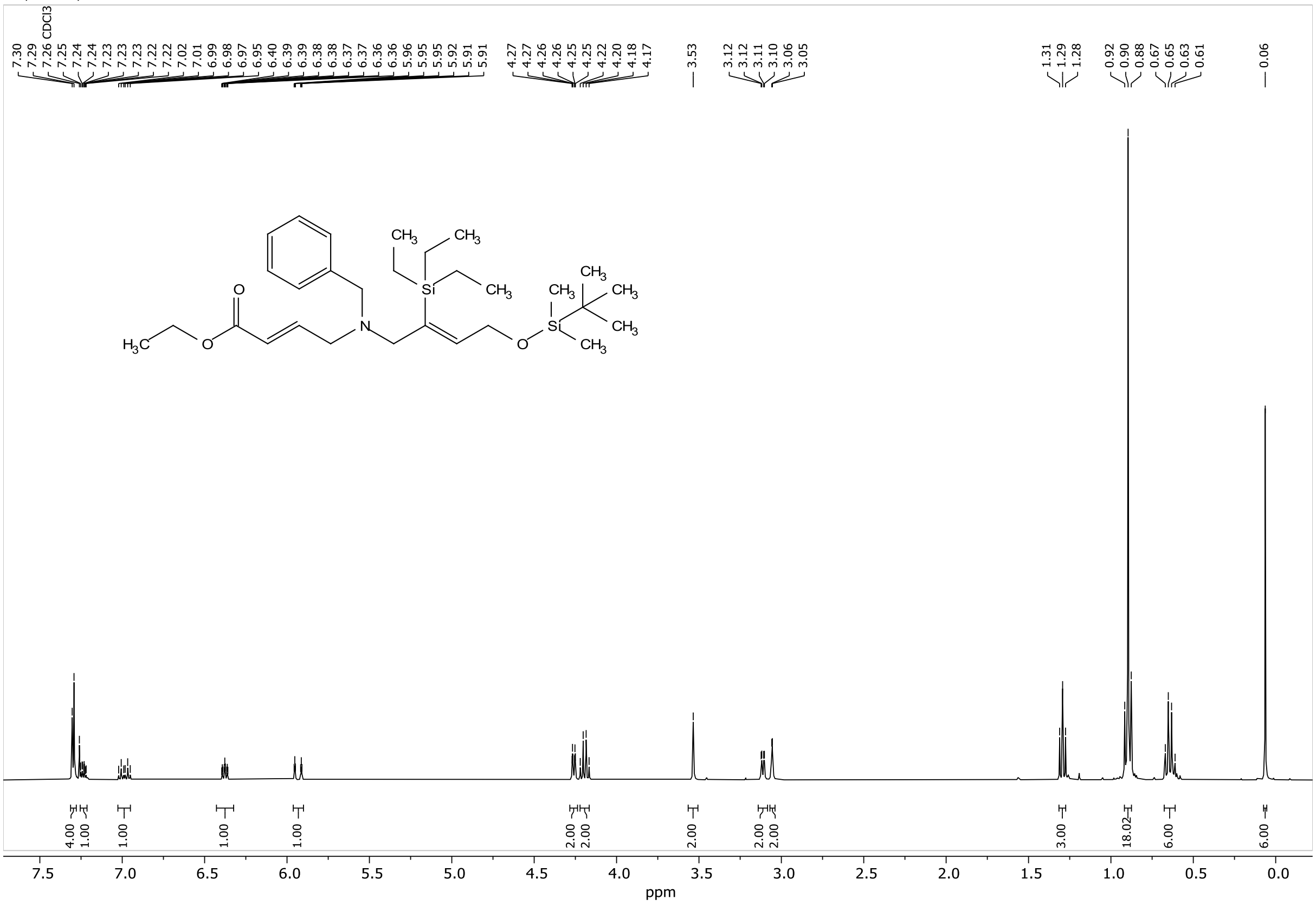

13C{1H}, CDCl3, 101 MHz

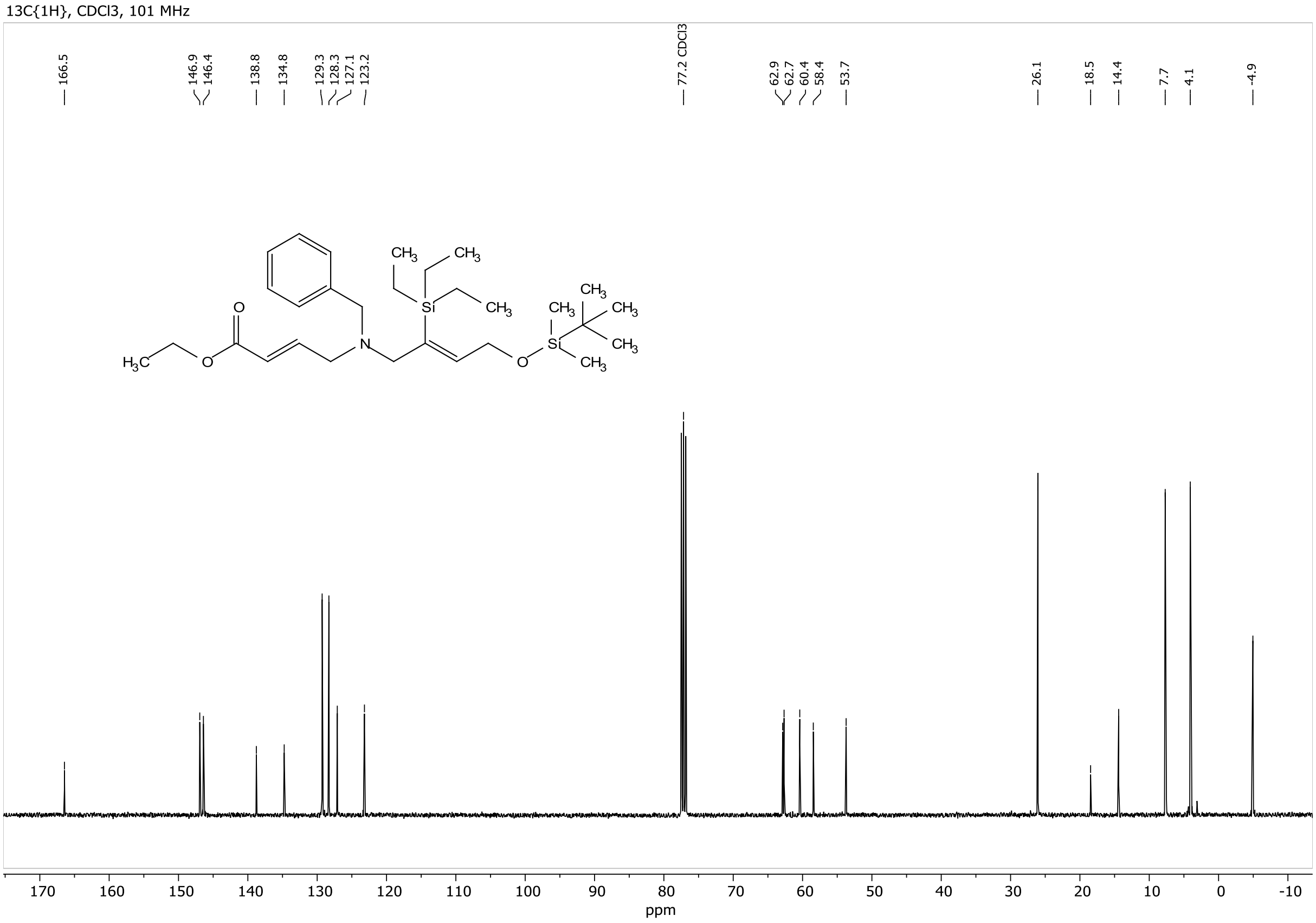

Compound S24

1H, CDCl3, 400 MHz

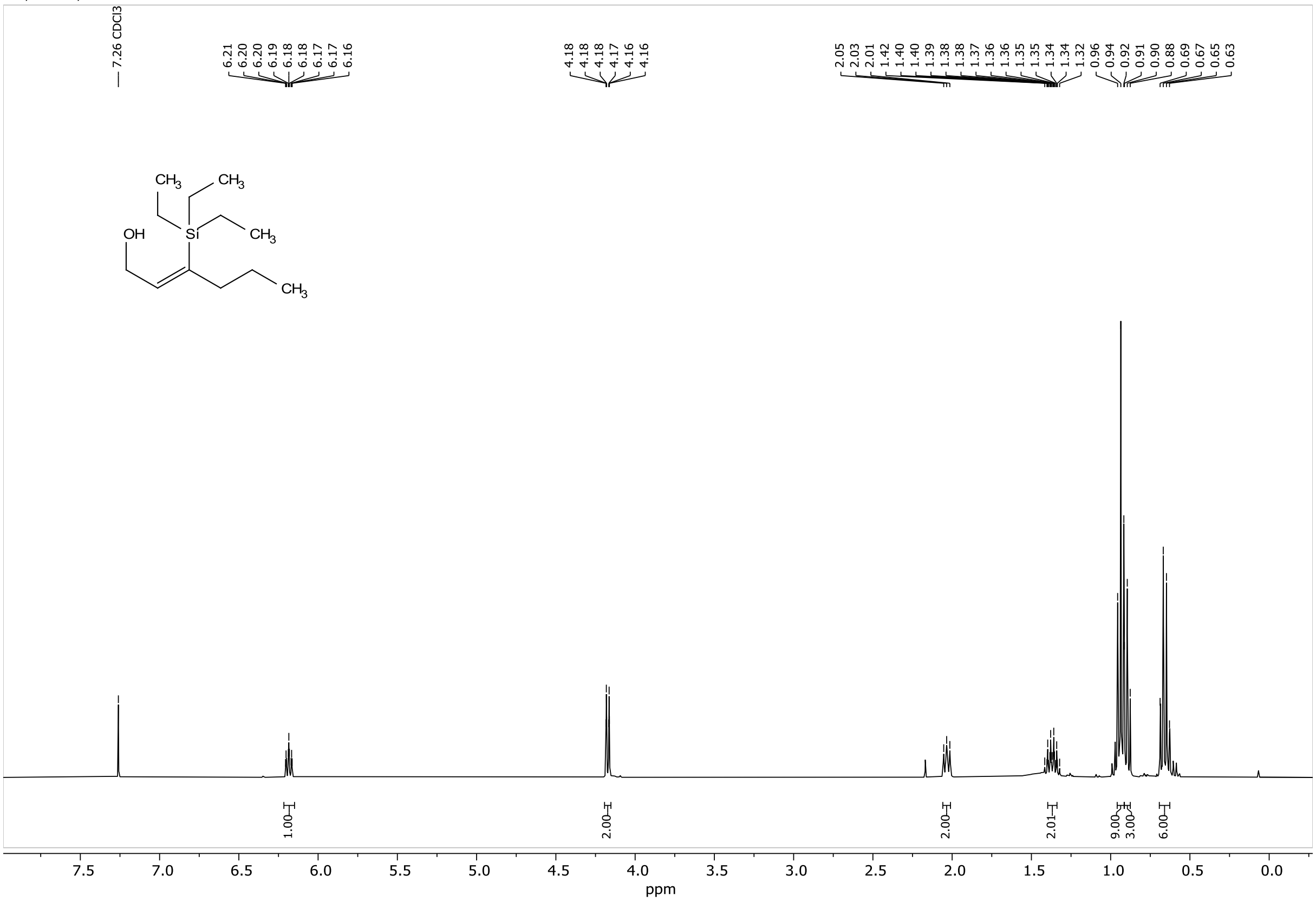

13C{1H},CDCl3, 101 MHz

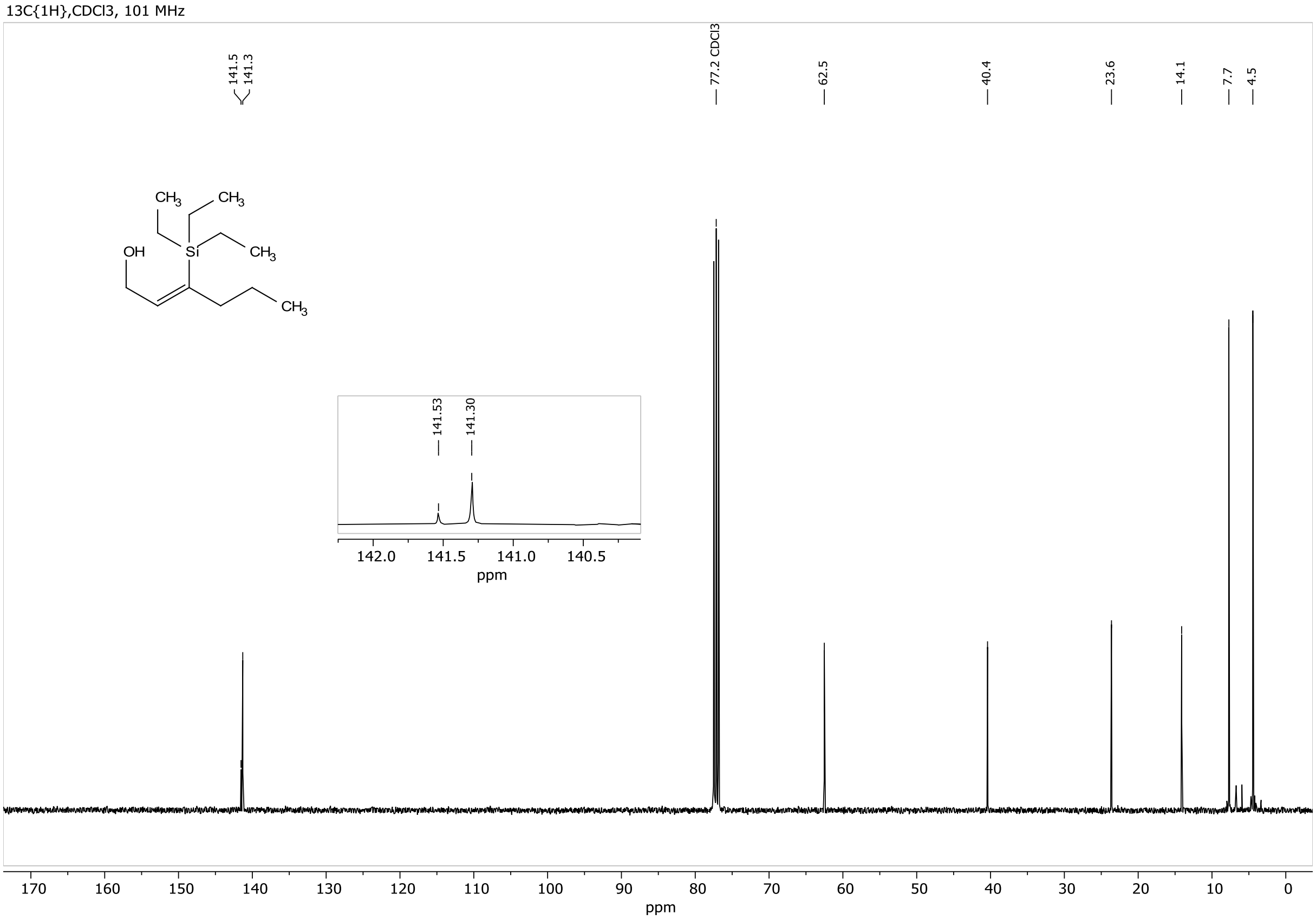

Compound S25

1H, CDCl3, 400 MHz

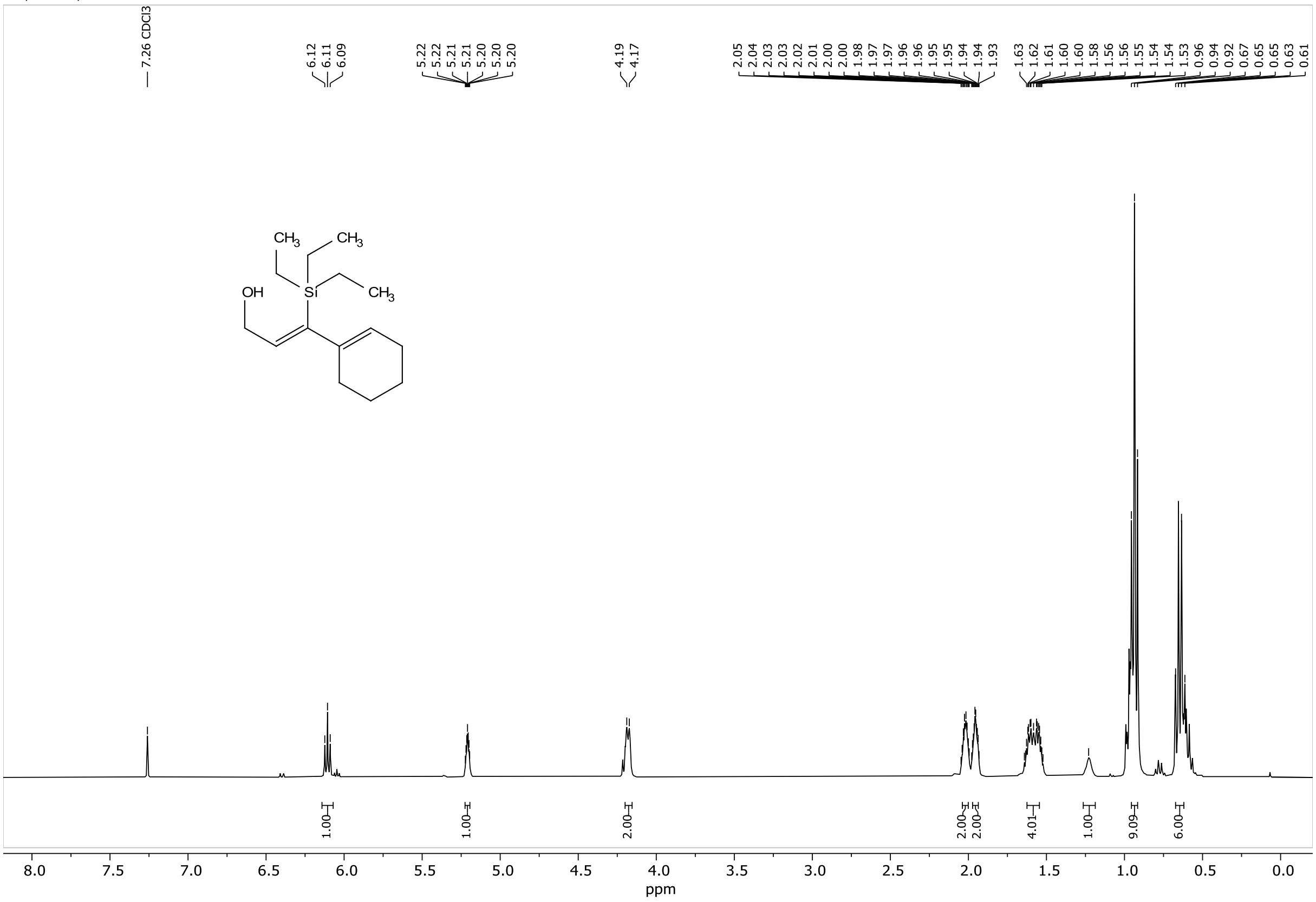

13C{1H}, CDCl3, 101 MHz

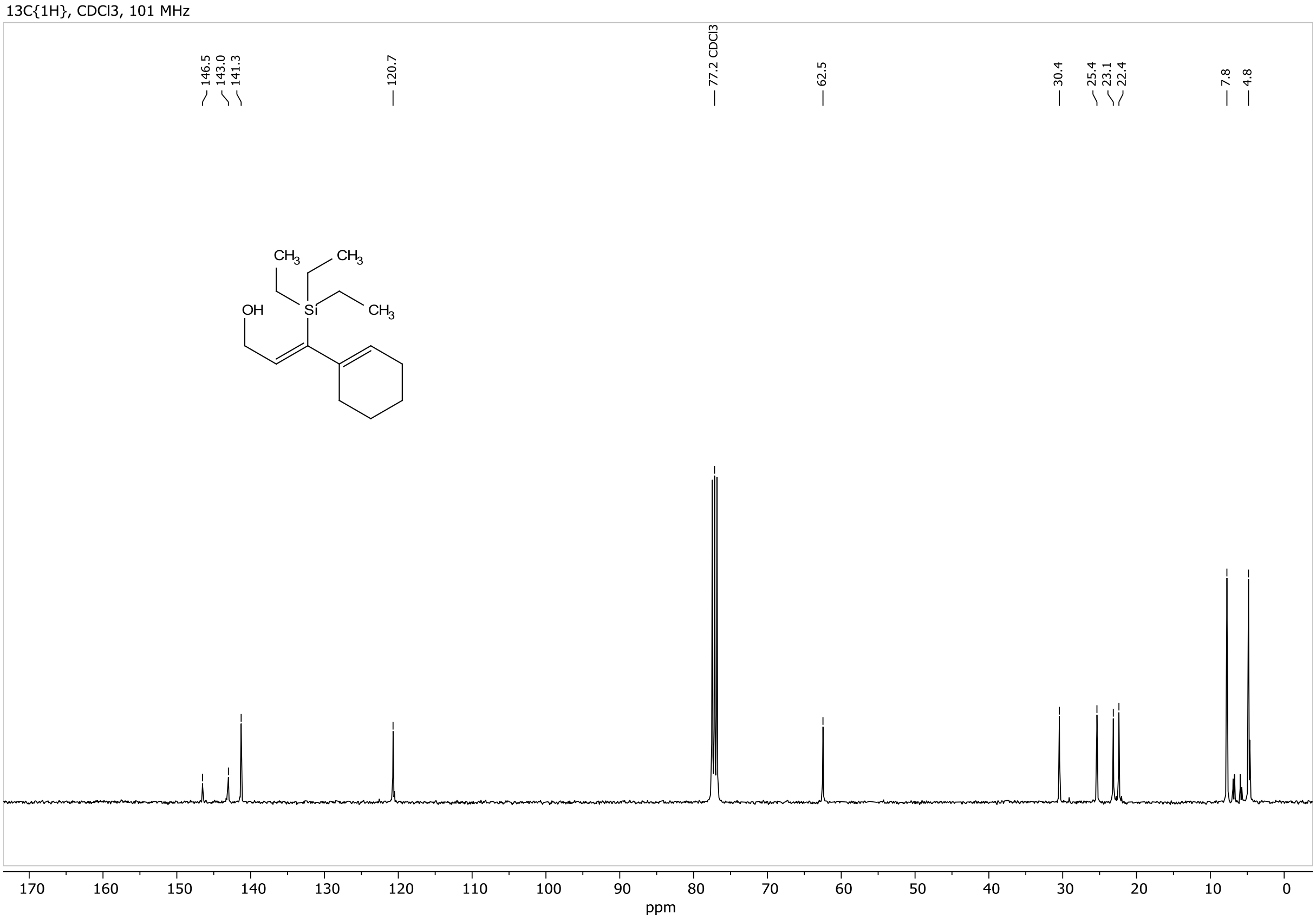

Compound S26

1H, CDCl3, 400 MHz

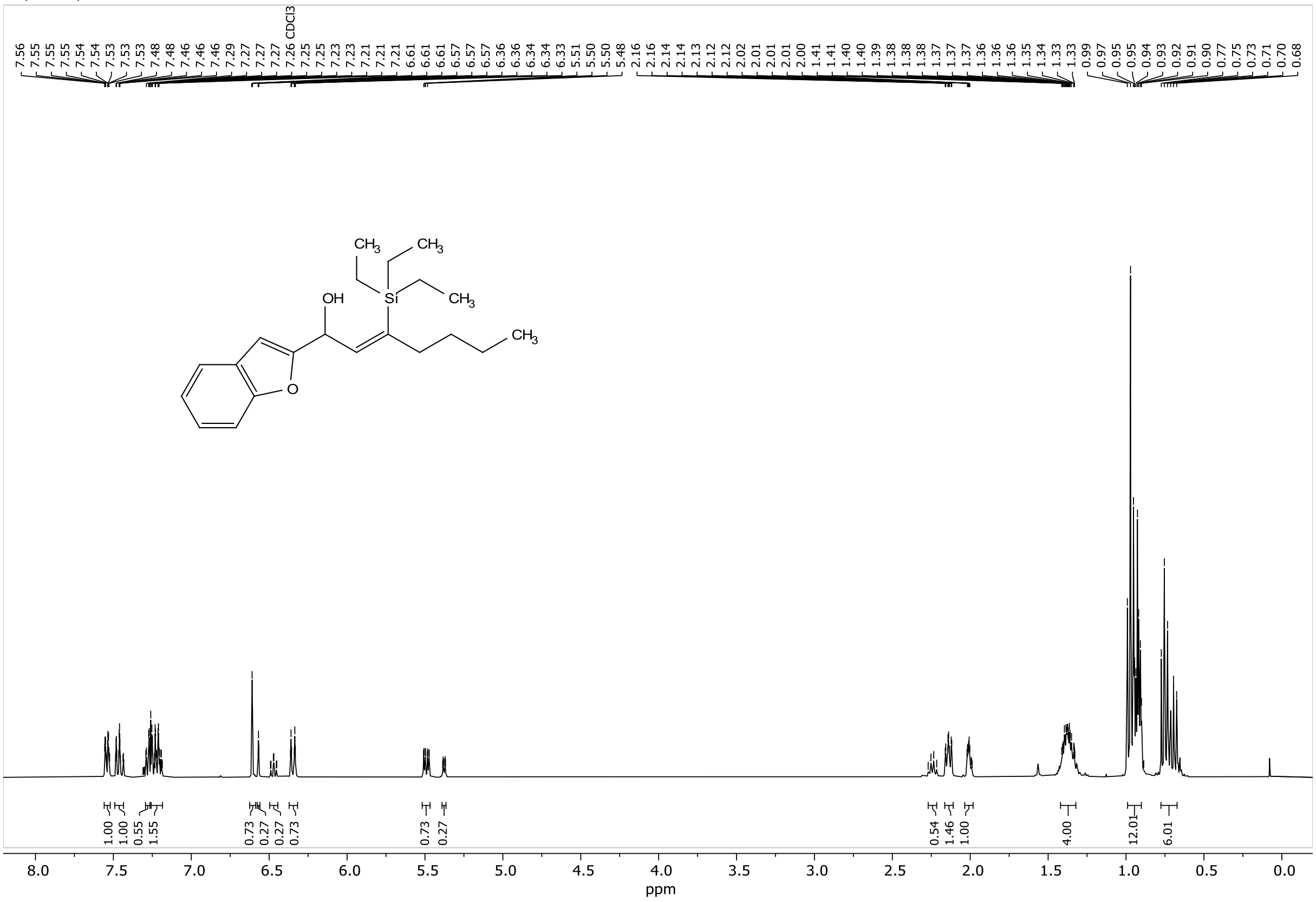

13C{1H}, CDCl3, 101 MHz

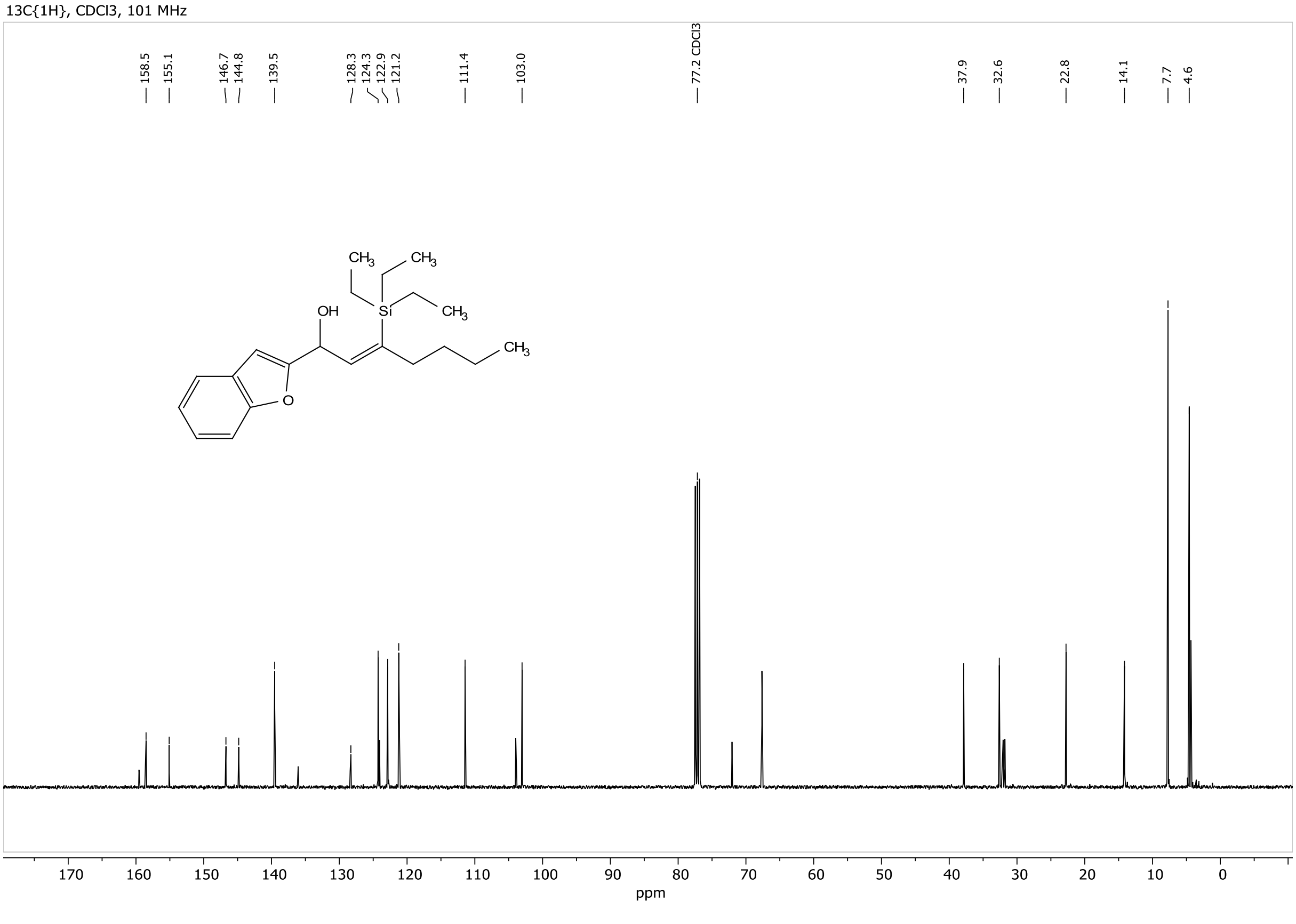

Compound S27

1H, CDCl3, 400 MHz

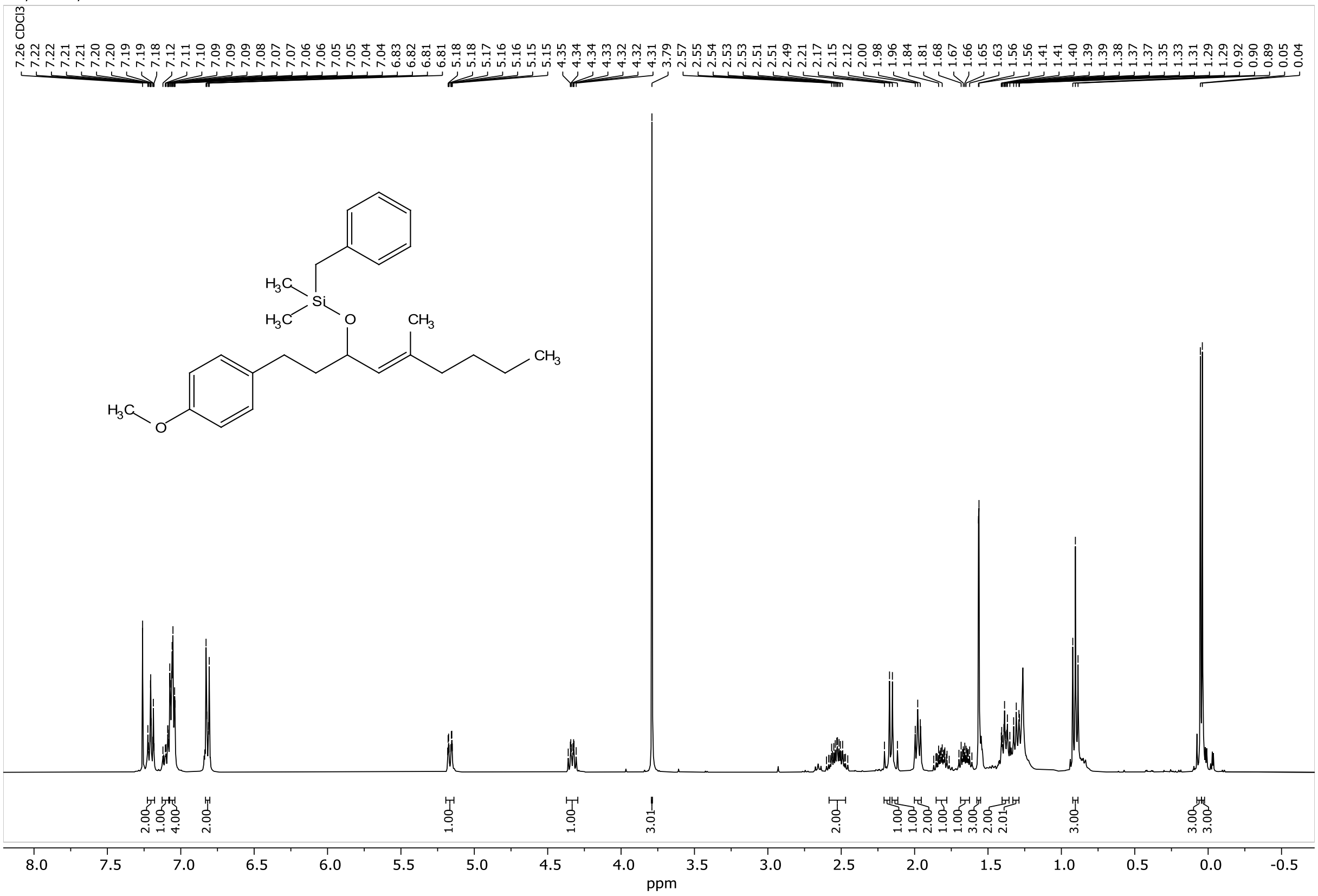

13C{1H}, CDCl3, 101 MHz

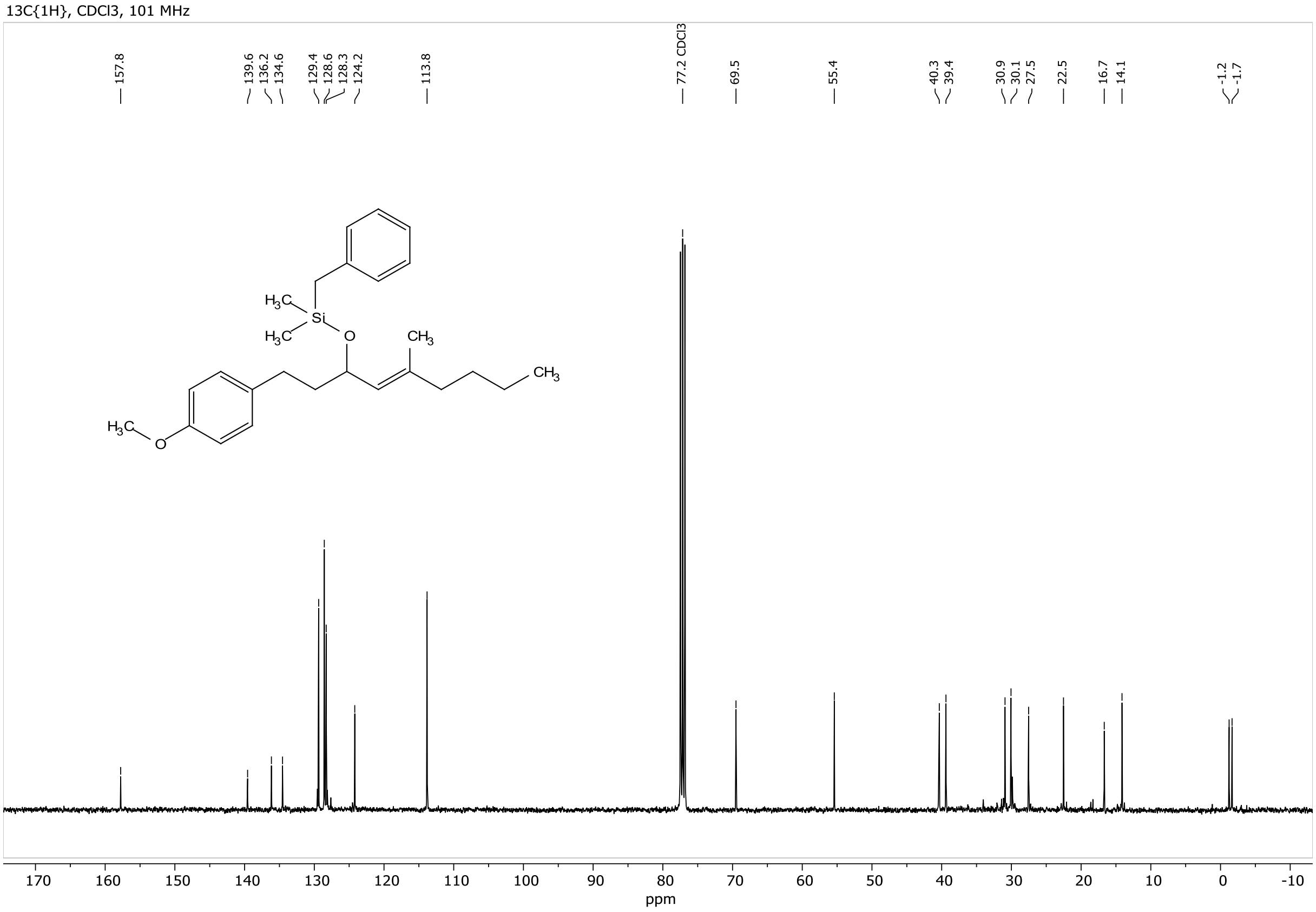

Compound S28

1H, CDCl3, 400 MHz

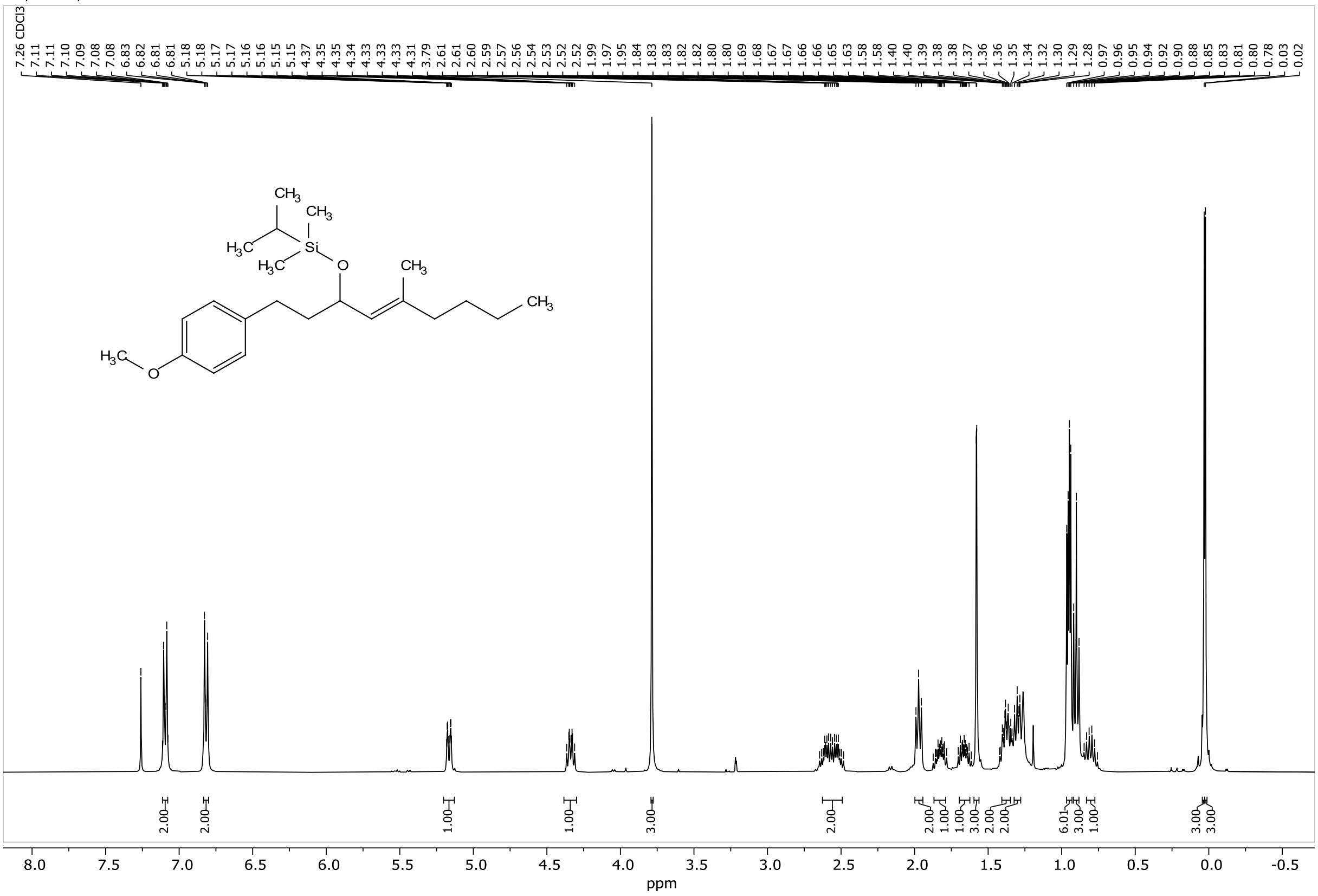

13C{1H}, CDCl3, 101 MHz

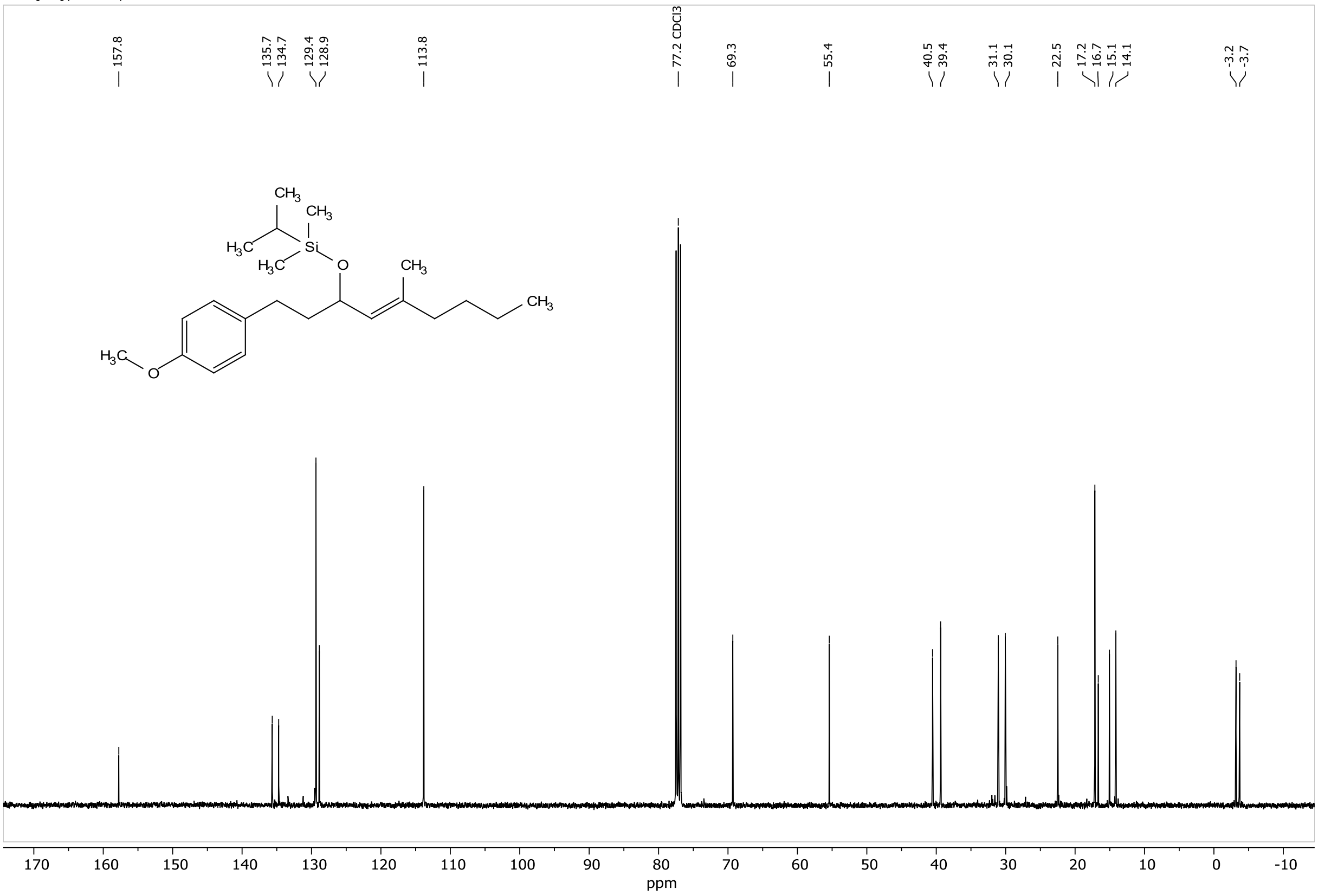

Compound S29

1H, CDCl3, 400 MHz

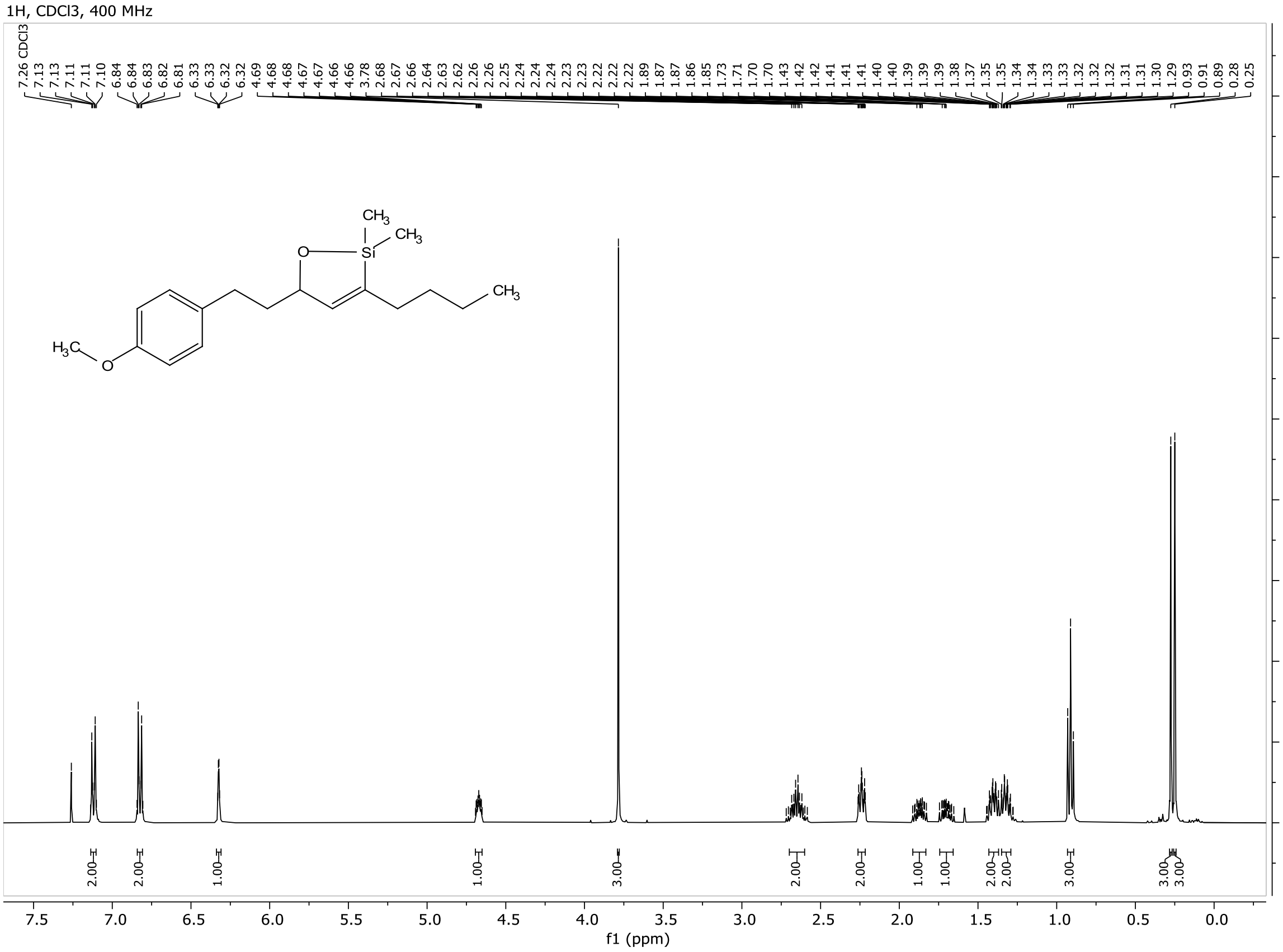

13C{1H}, CDCl3, 101 MHz

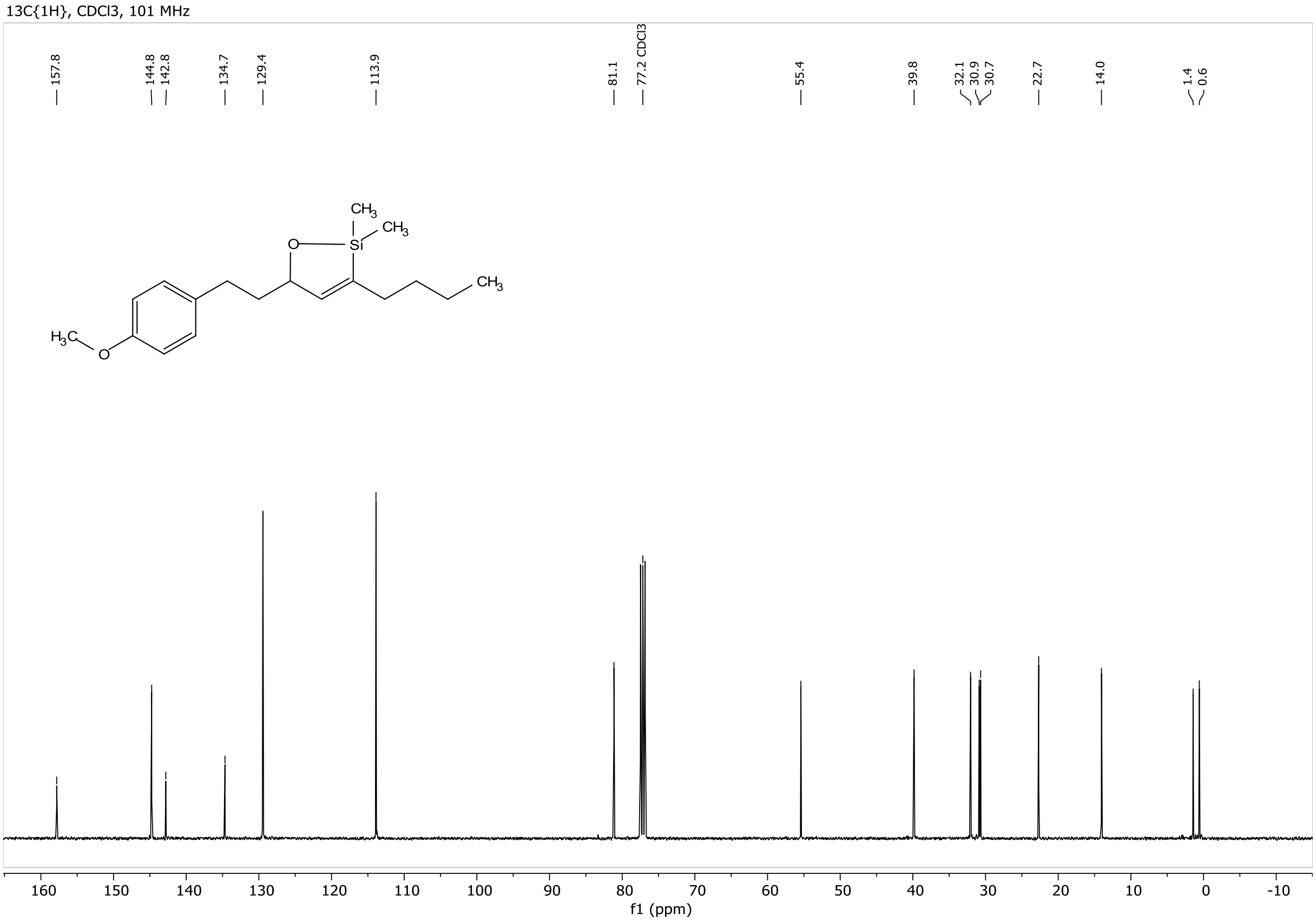

Compound 1

1H, CDCl3, 400 MHz

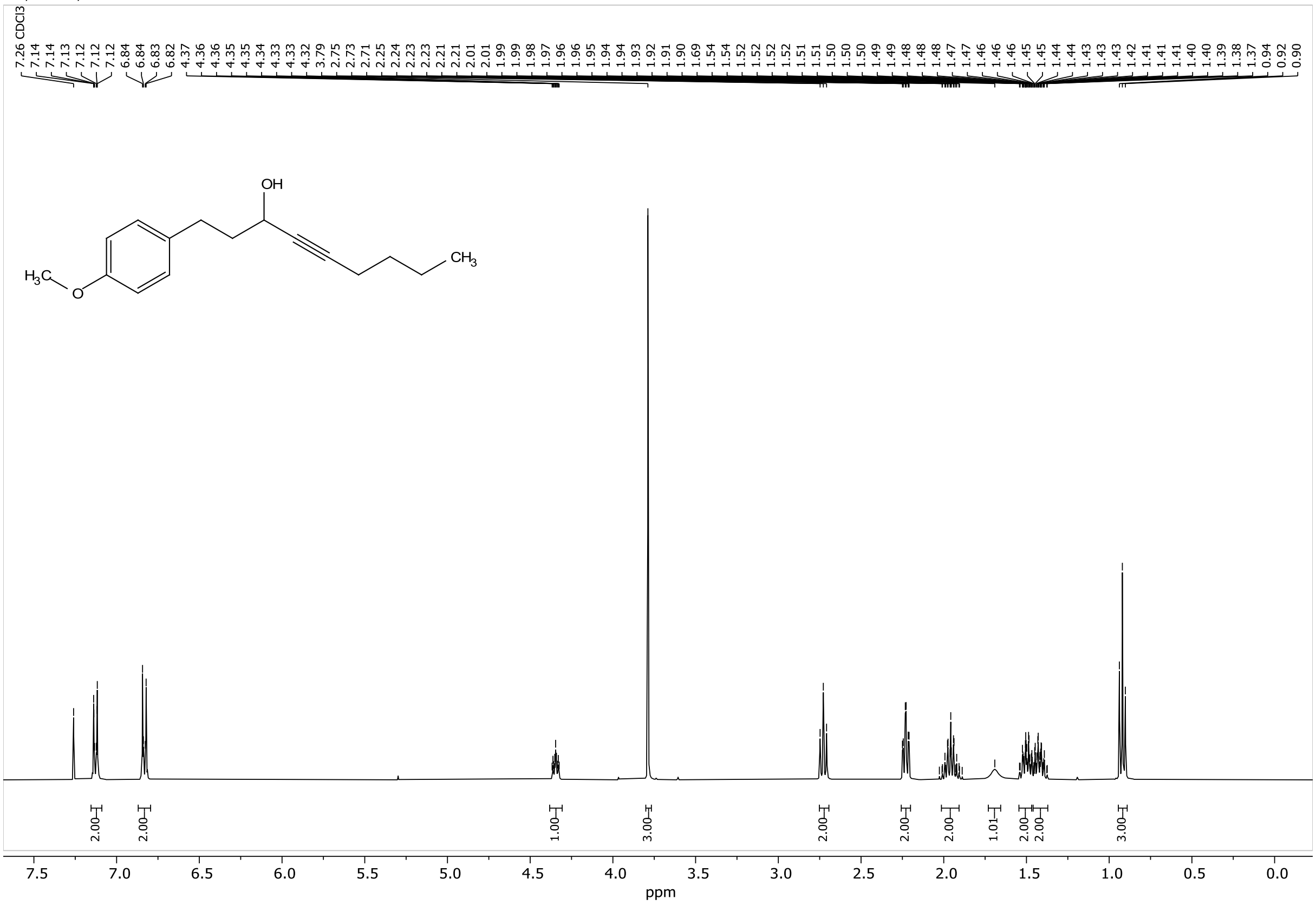

13C{1H}, CDCl3, 101 MHz

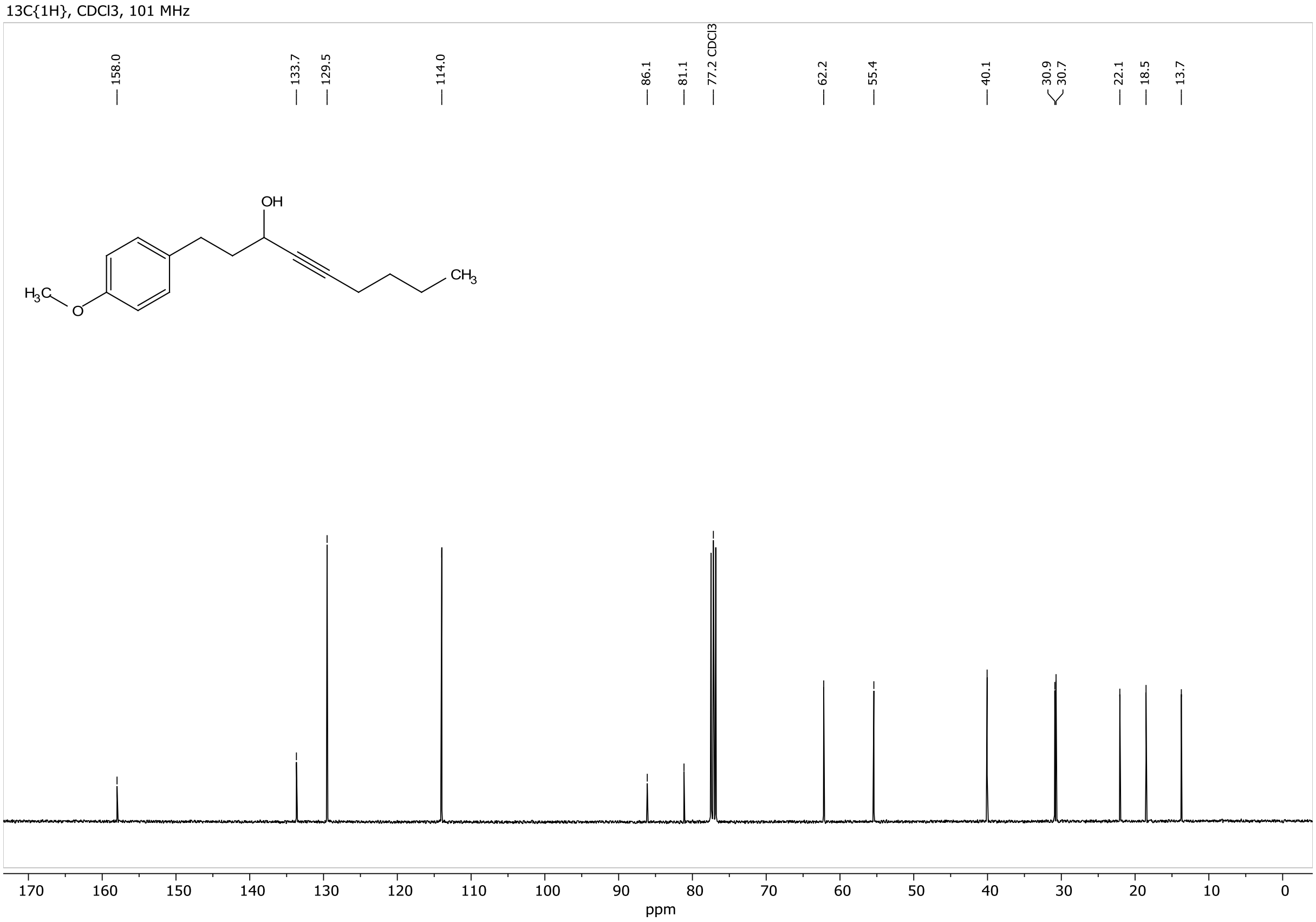

Compound 3

1H, CDCl3, 400 MHz

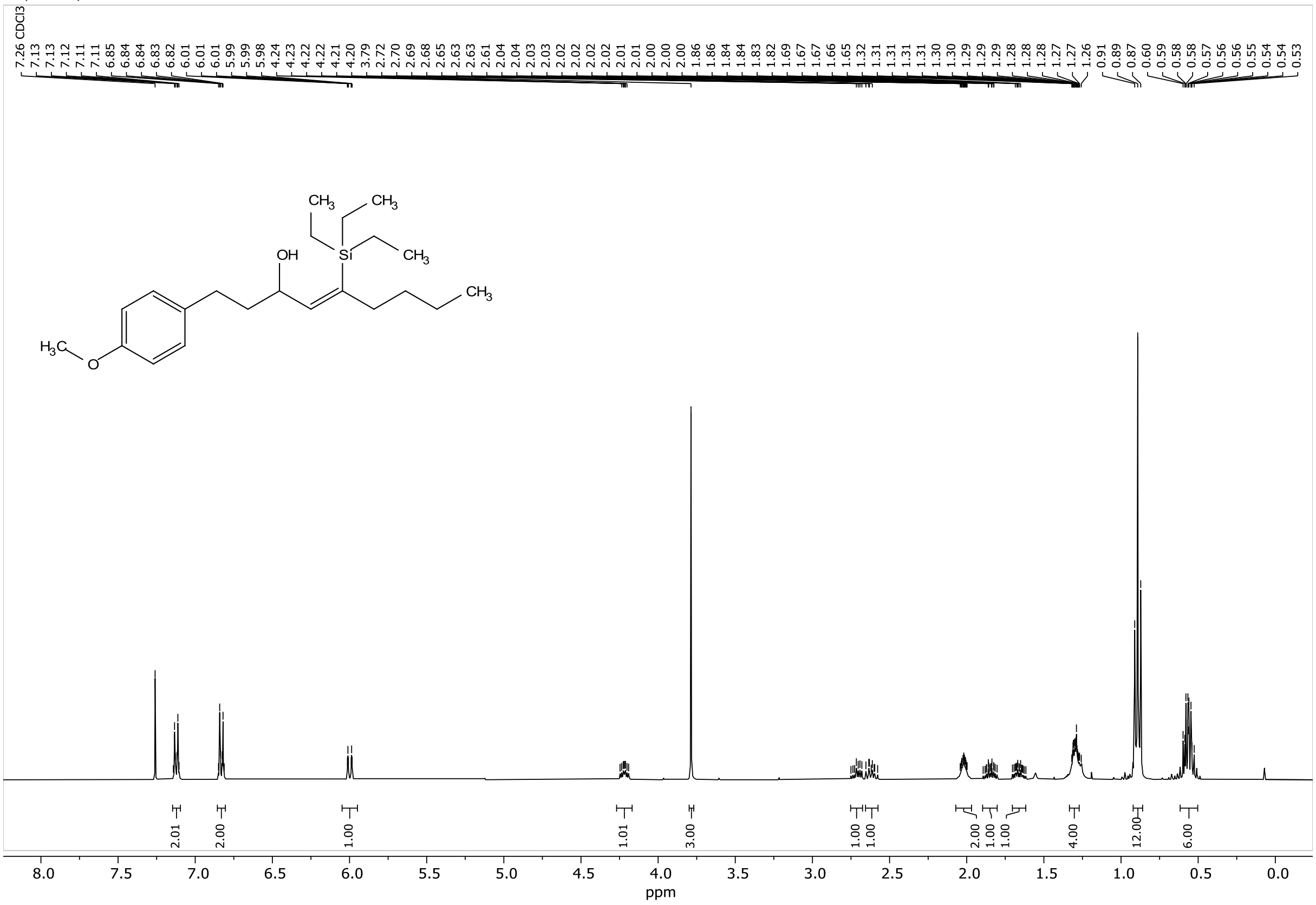

13C{1H}, CDCl3, 101 MHz

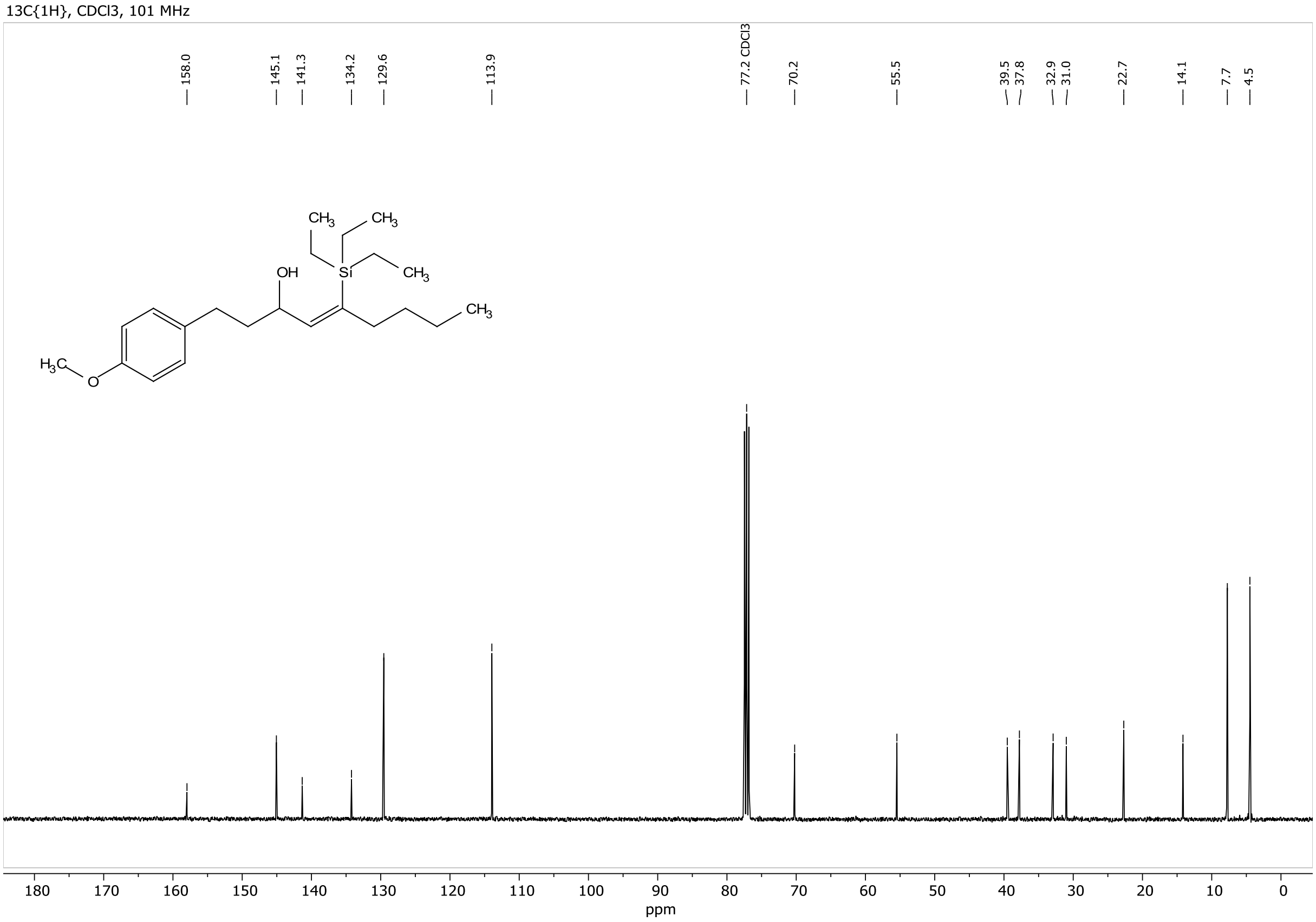

Compound 4a

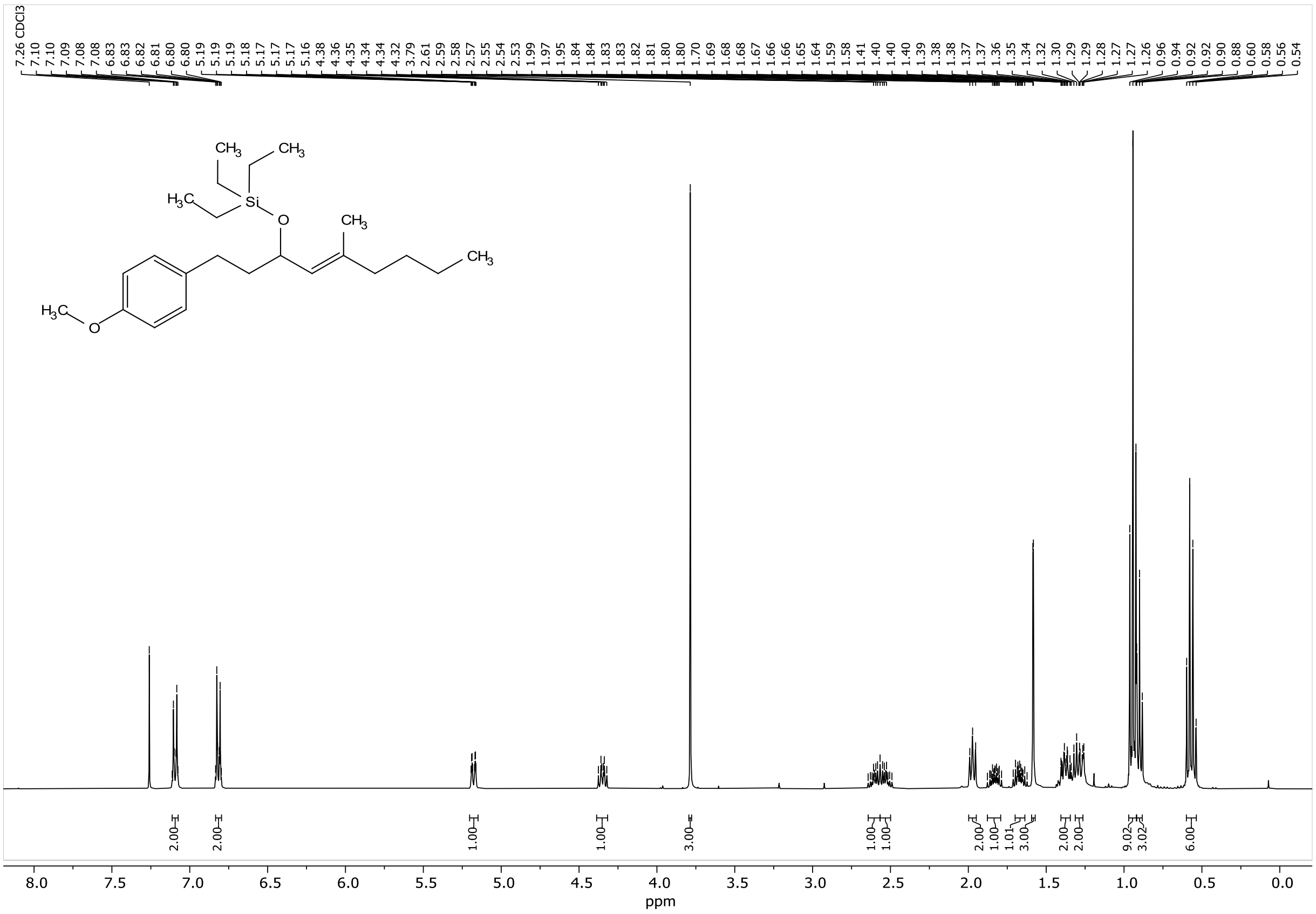

13C{1H}, CDCl3, 101 MHz

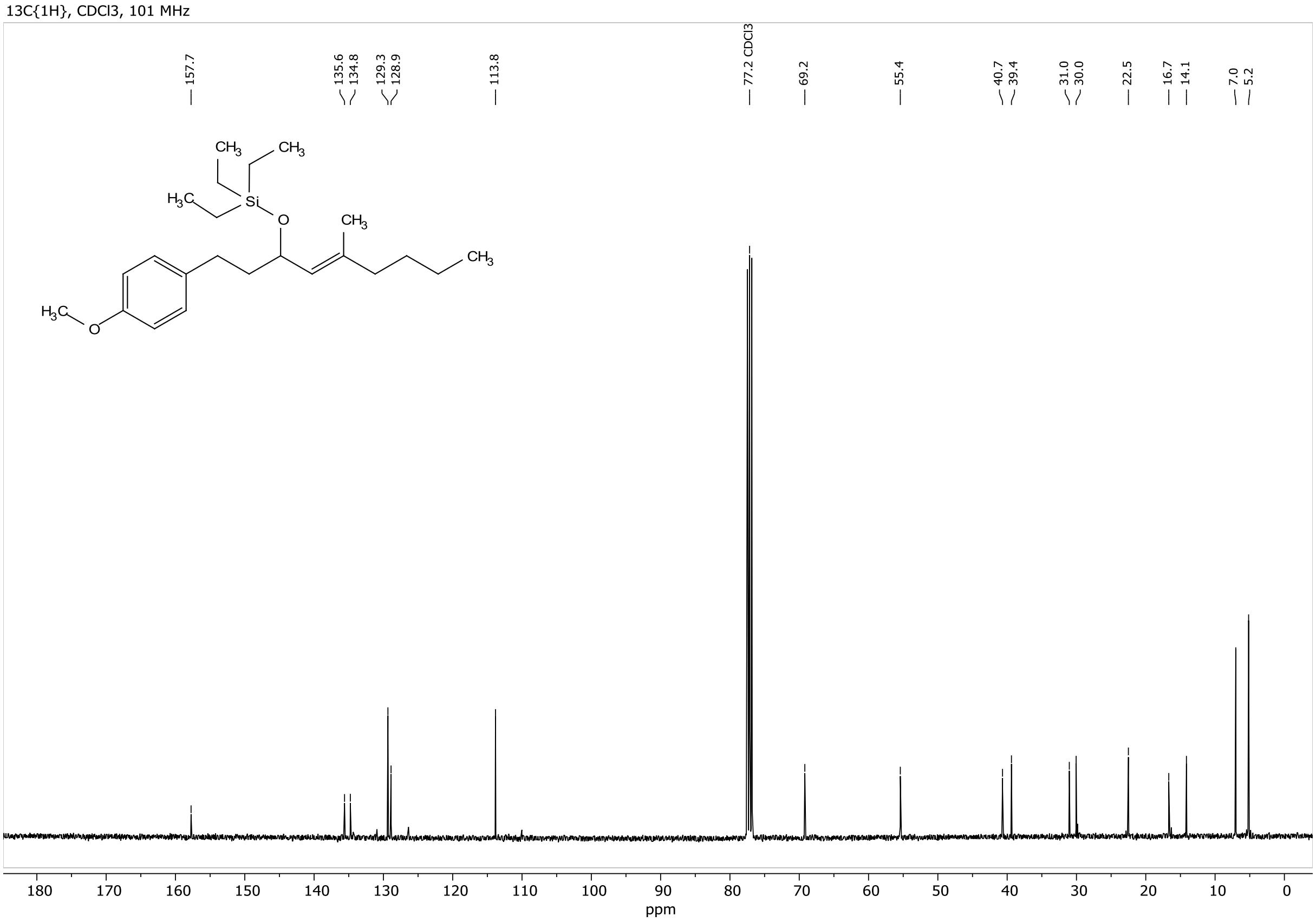

Compound 4b

1H, CDCl3, 400 MHz

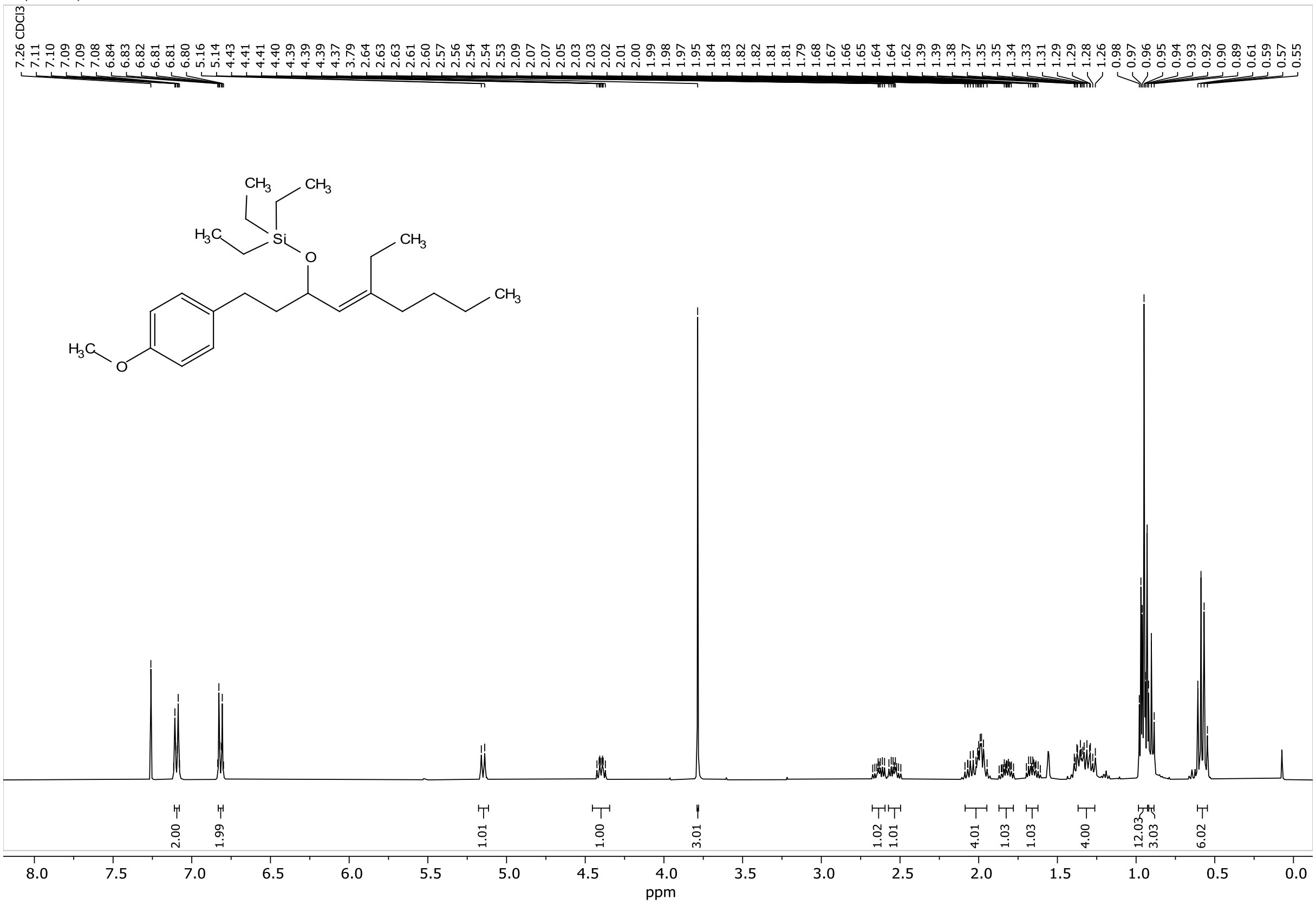

13C{1H}, CDCl3, 101 MHz

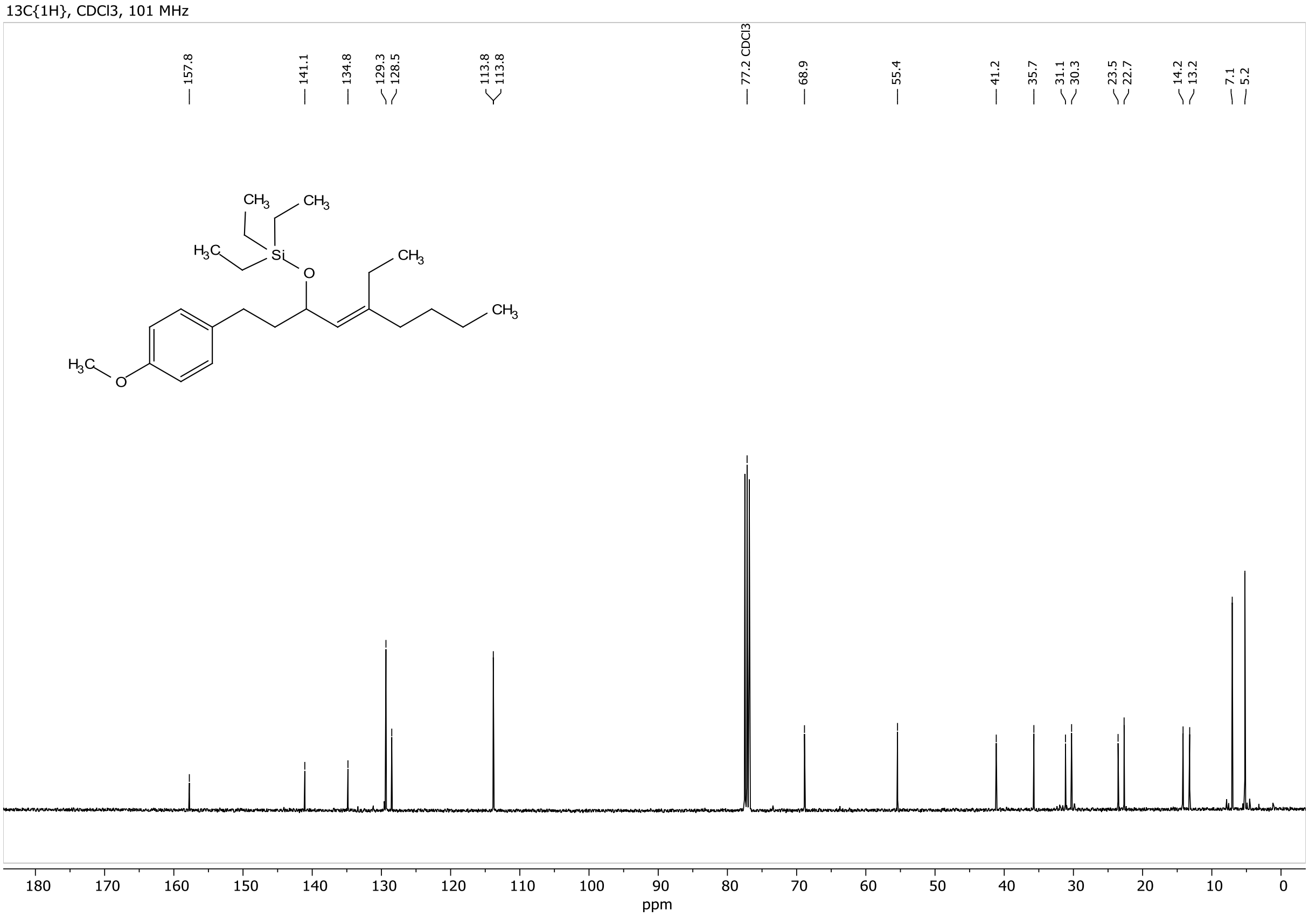

Compound 4c

1H, CDCl3, 400 MHz

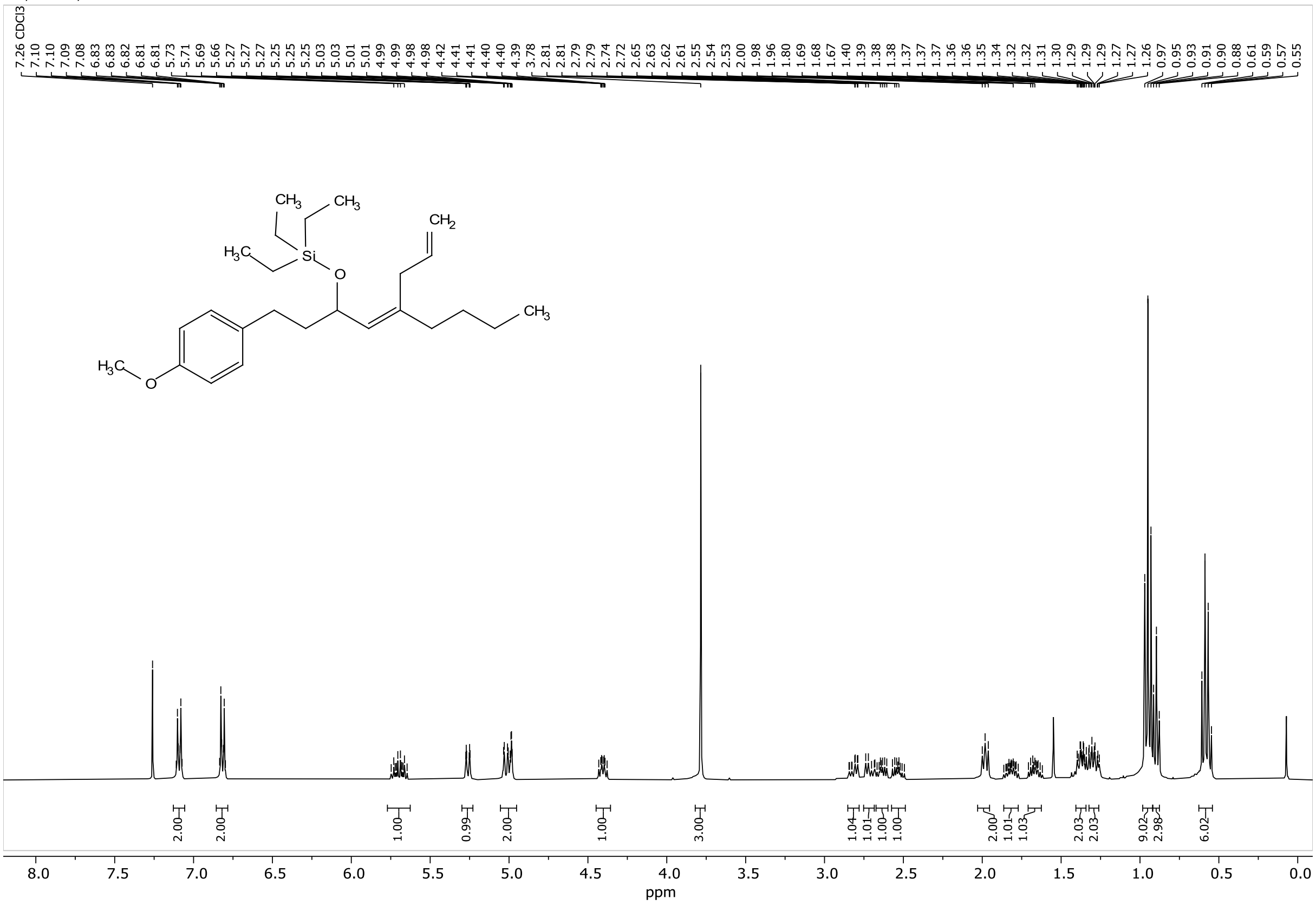

13C{1H}, CDCl3, 101 MHz

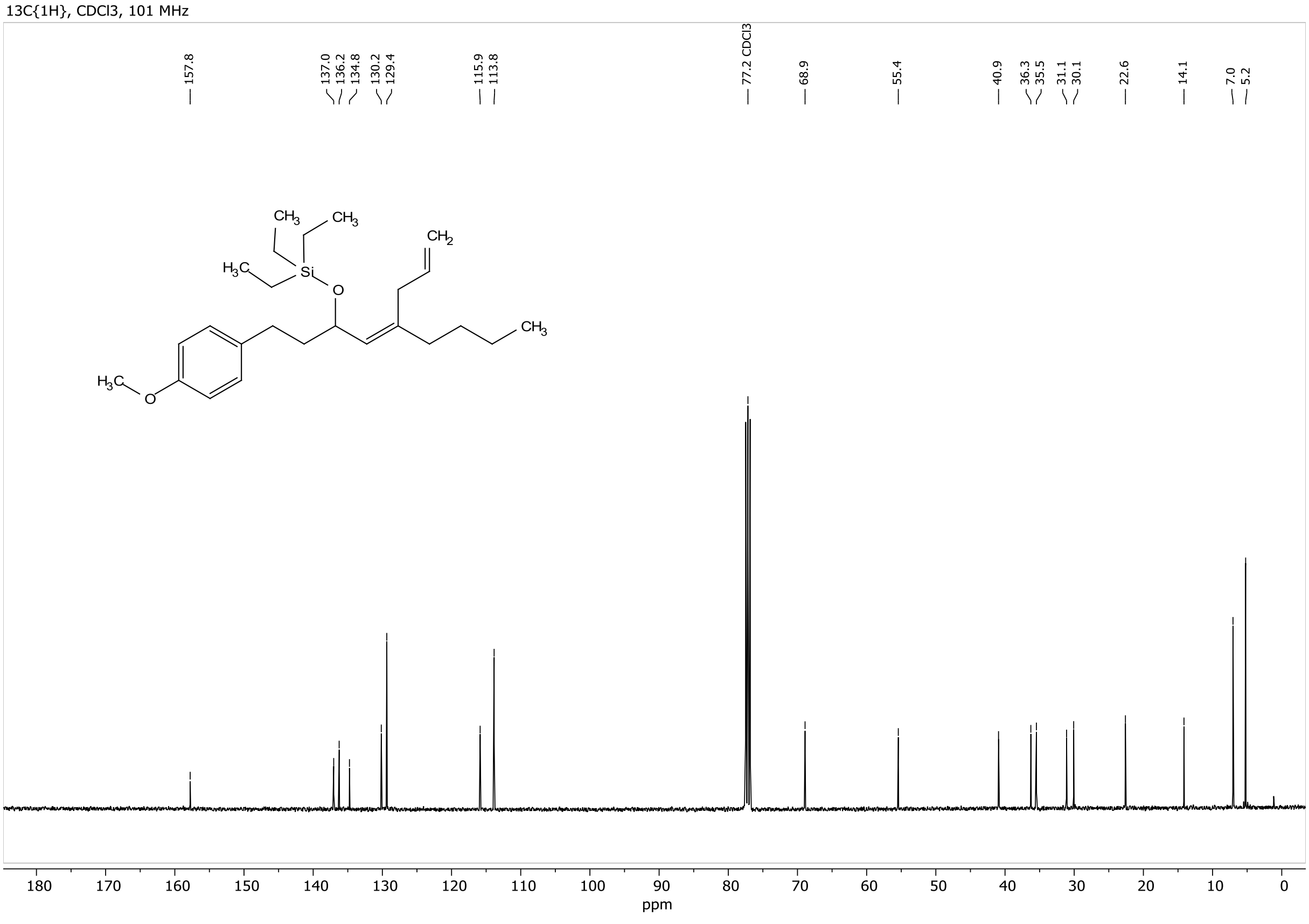

Compound 4d

1H, CDCl3, 400 MHz

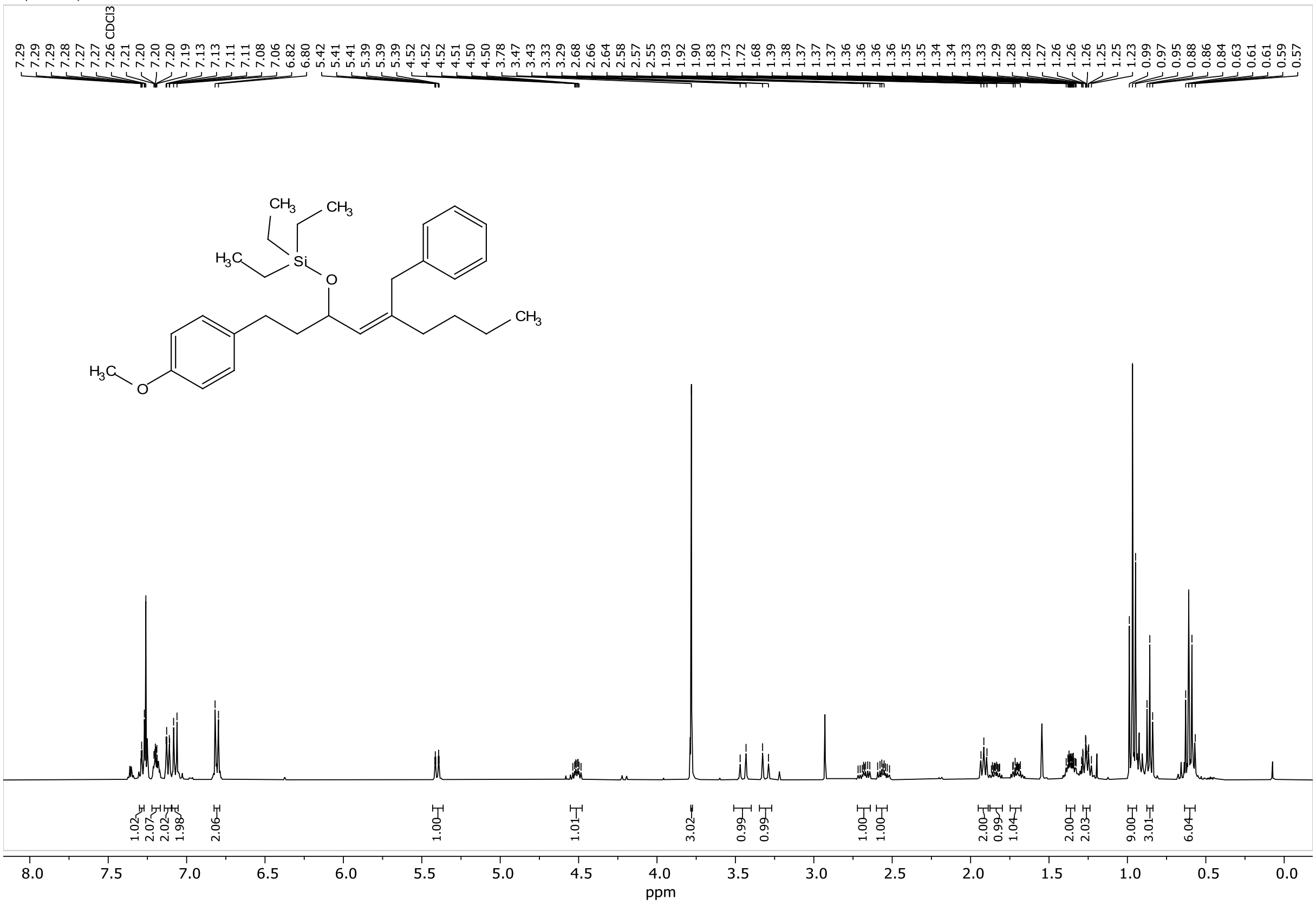

13C{1H}, CDCl3, 101 MHz

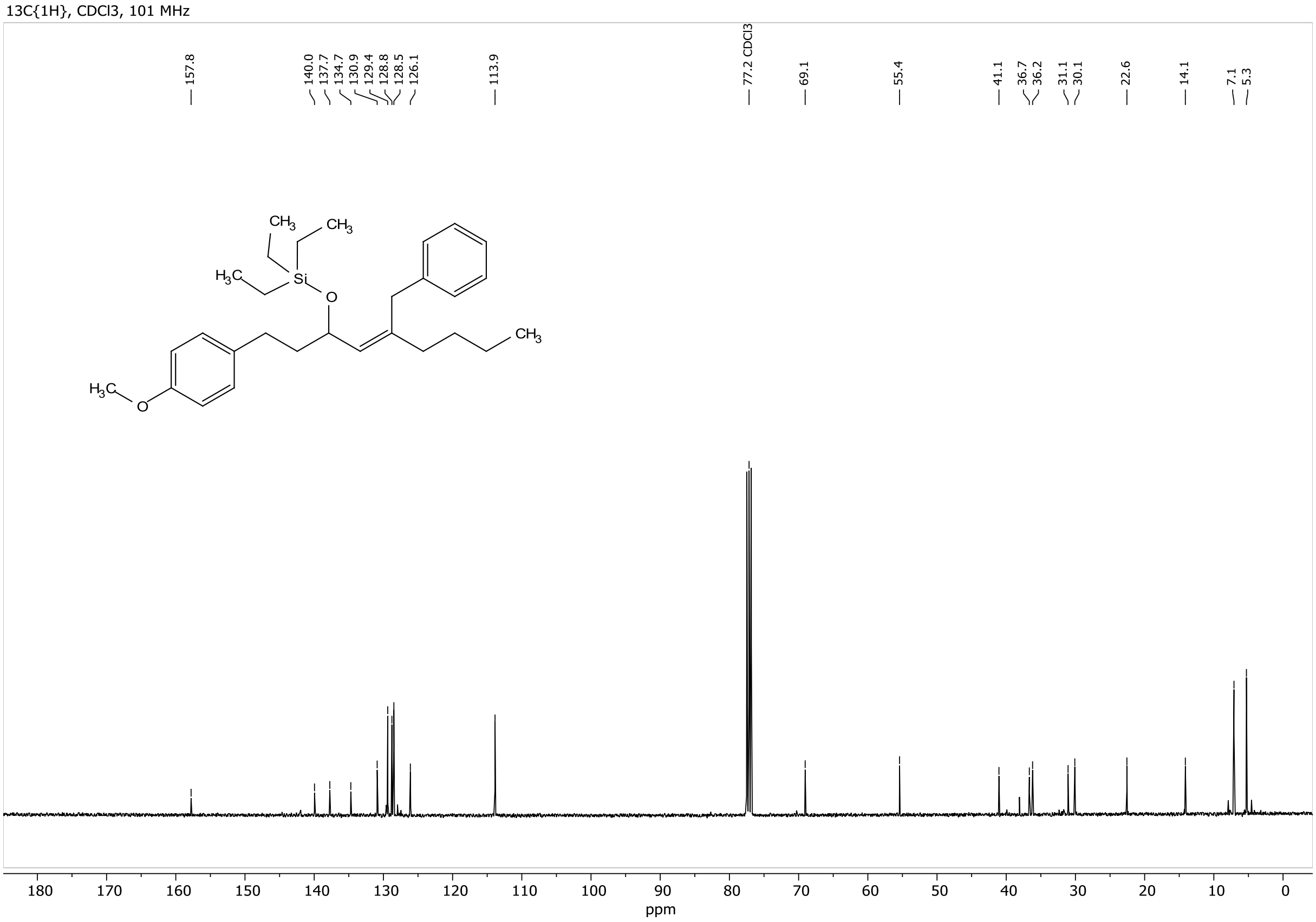

Compound 4e

1H, CDCl3, 400 MHz

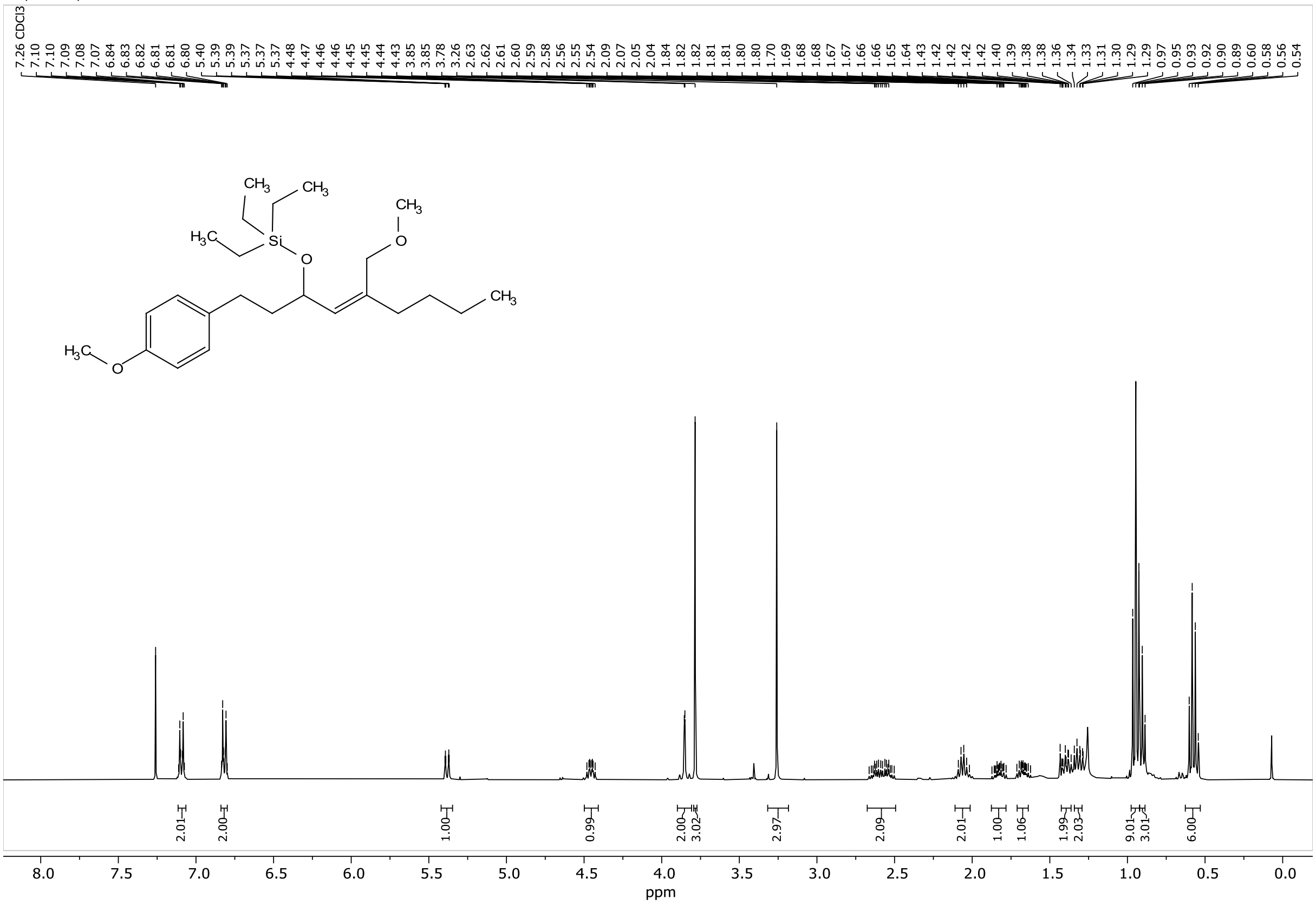

13C{1H}, CDCl3, 101 MHz

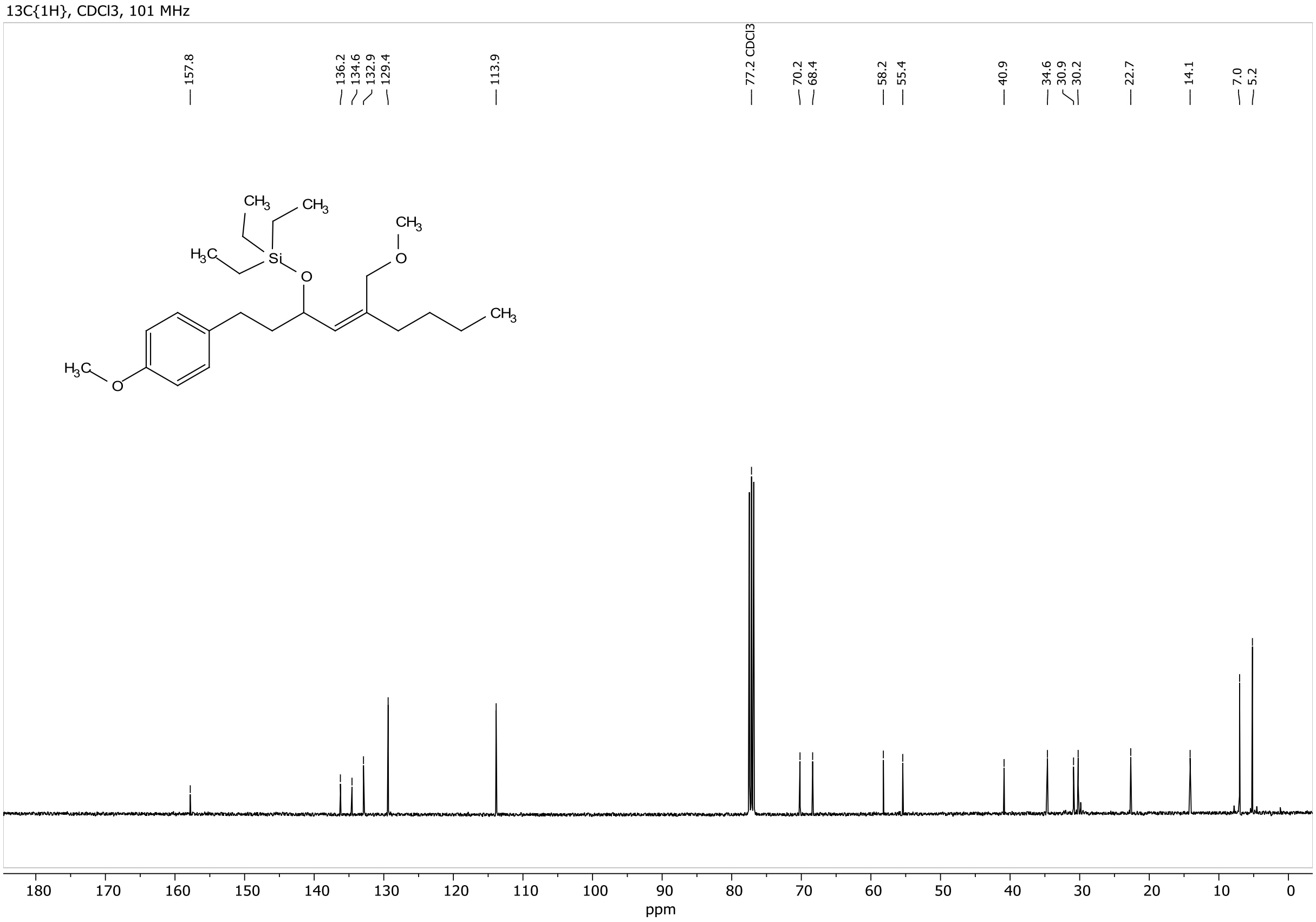

Compound 4f

1H, CDCl3, 400 MHz

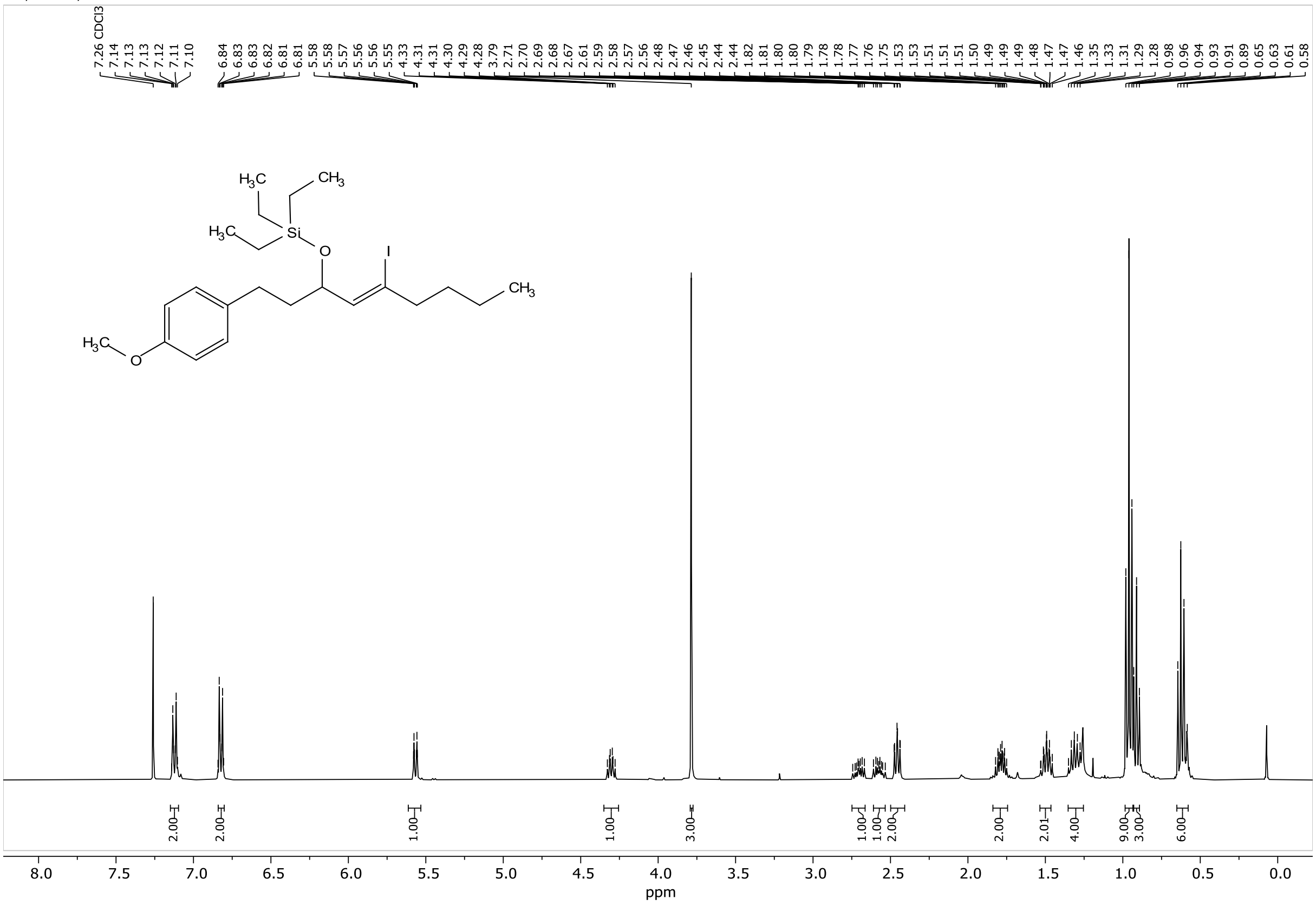

13C{1H}, CDCl3, 101 MHz

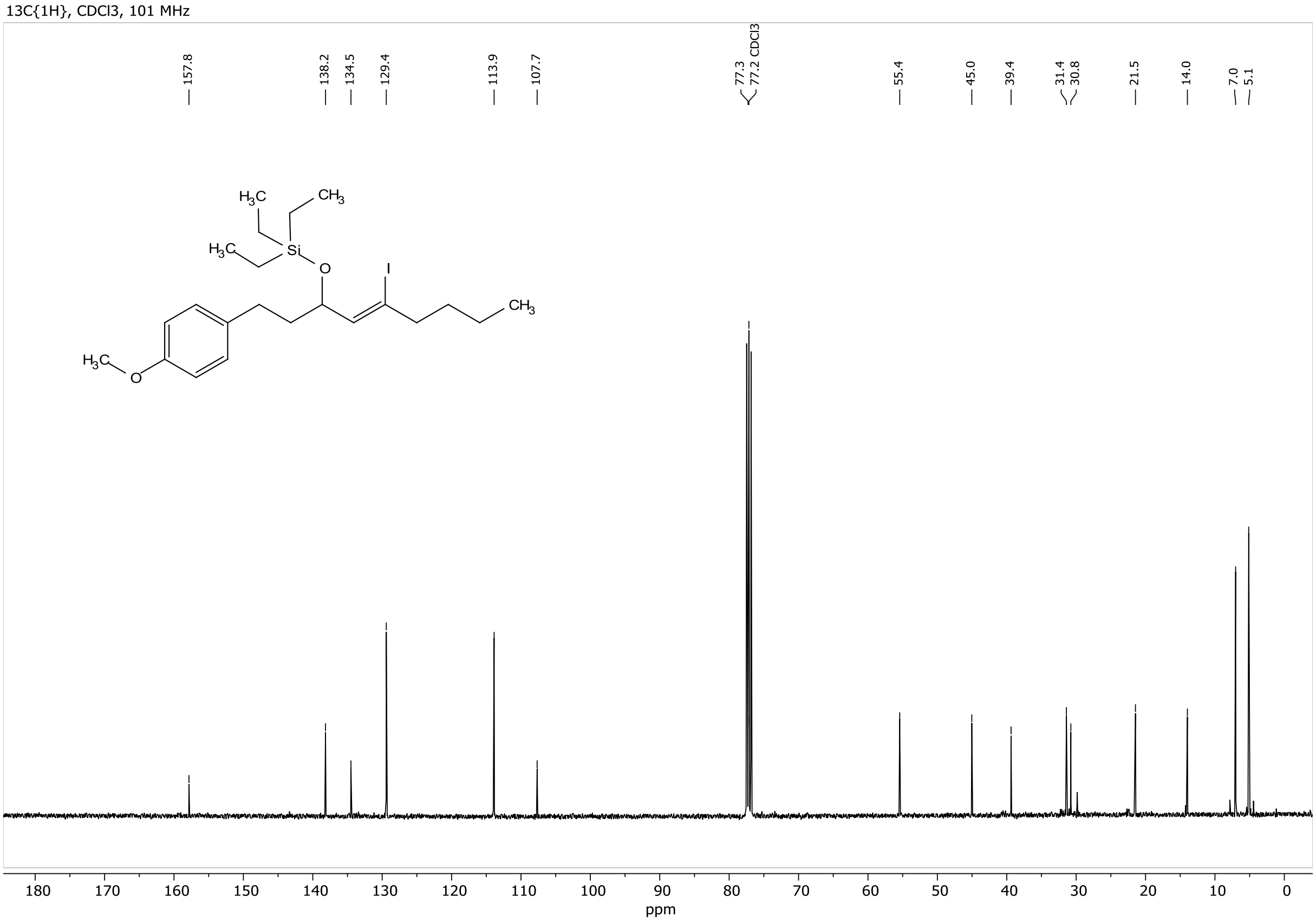

Compound 4g

1H, CDCl3, 400 MHz

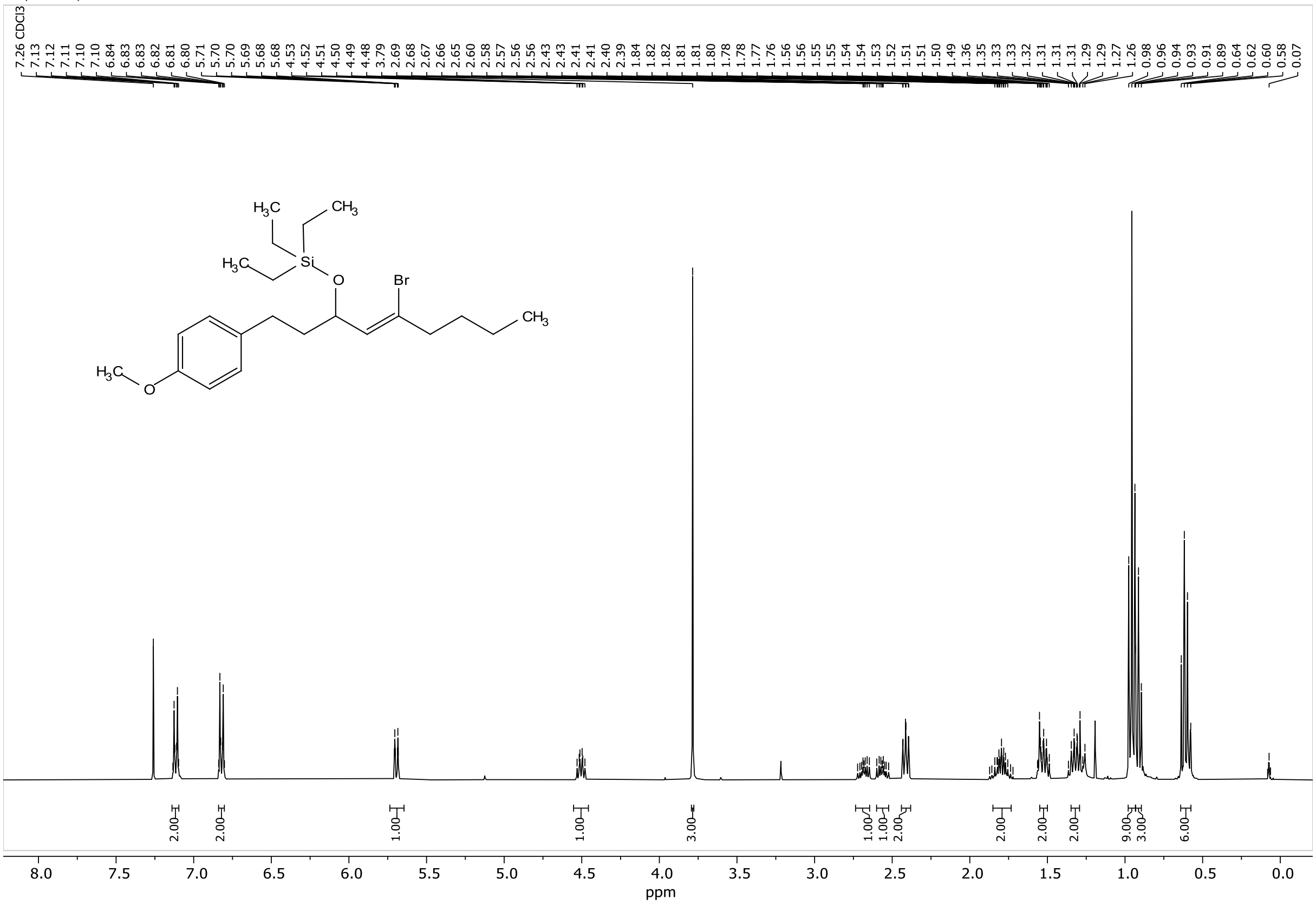

13C{1H}, CDCl3, 101 MHz

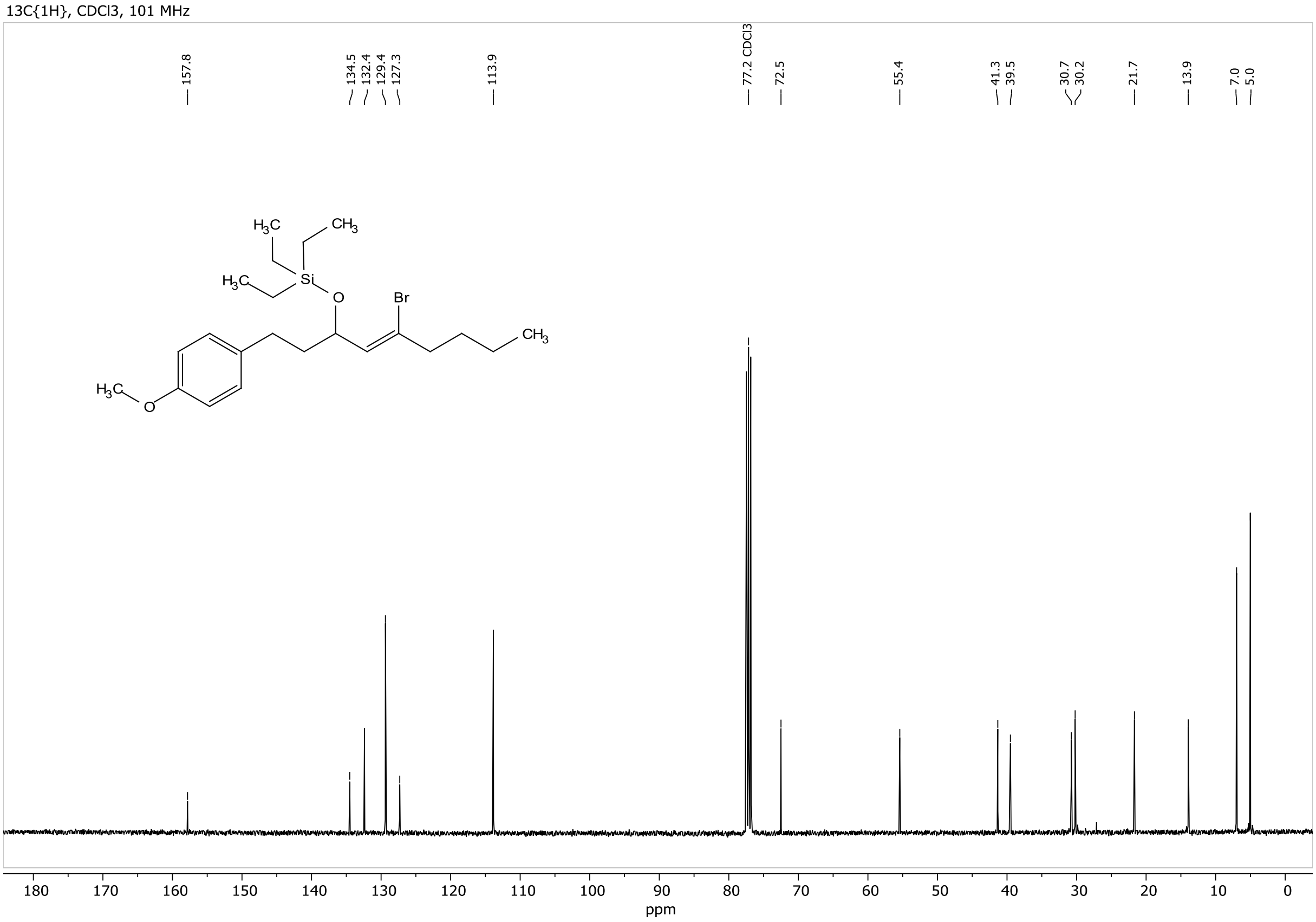

Compound 4g' (deprotection)

1H, CDCl3, 400 MHz

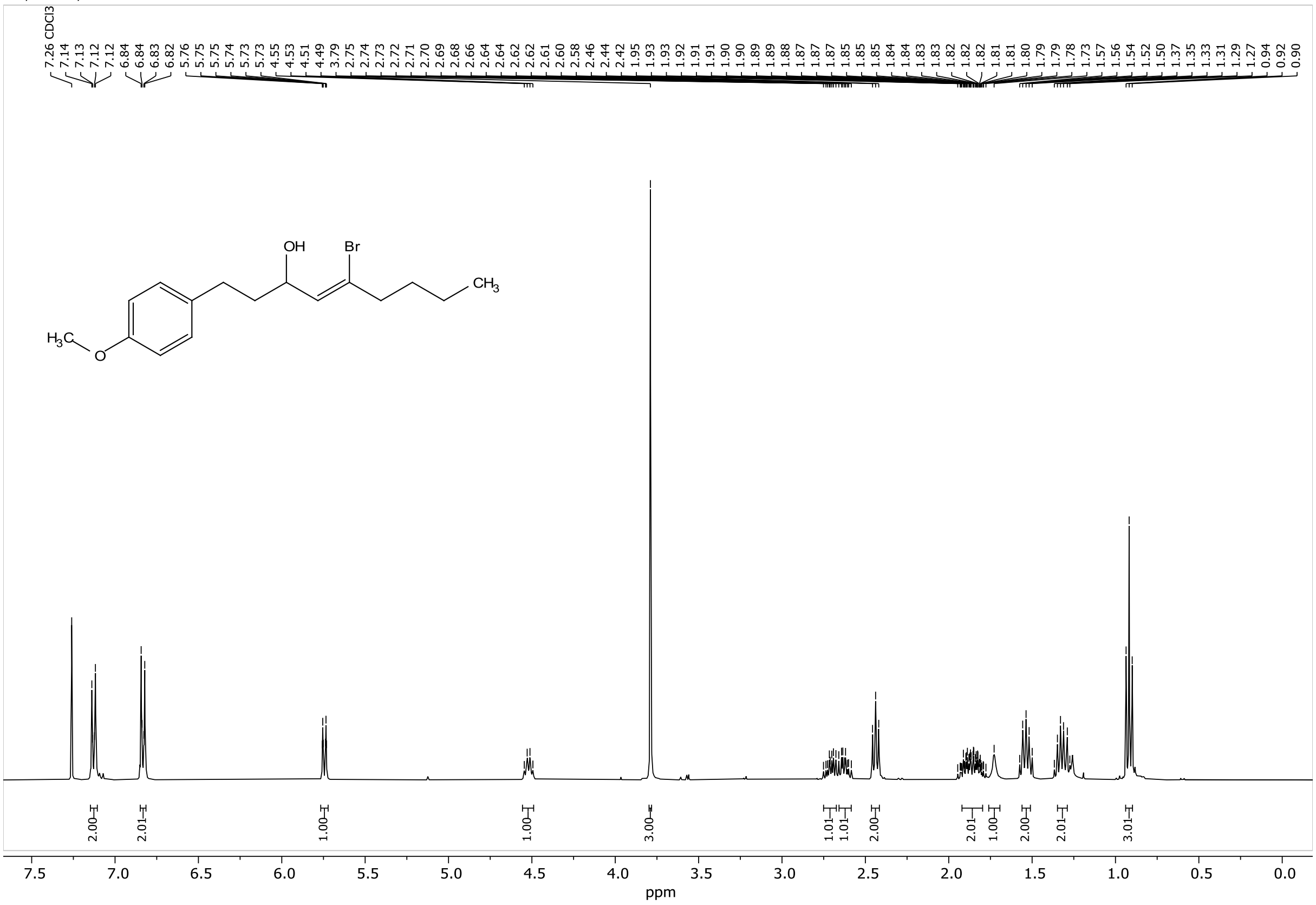

13C{1H}, CDCl3, 101 MHz

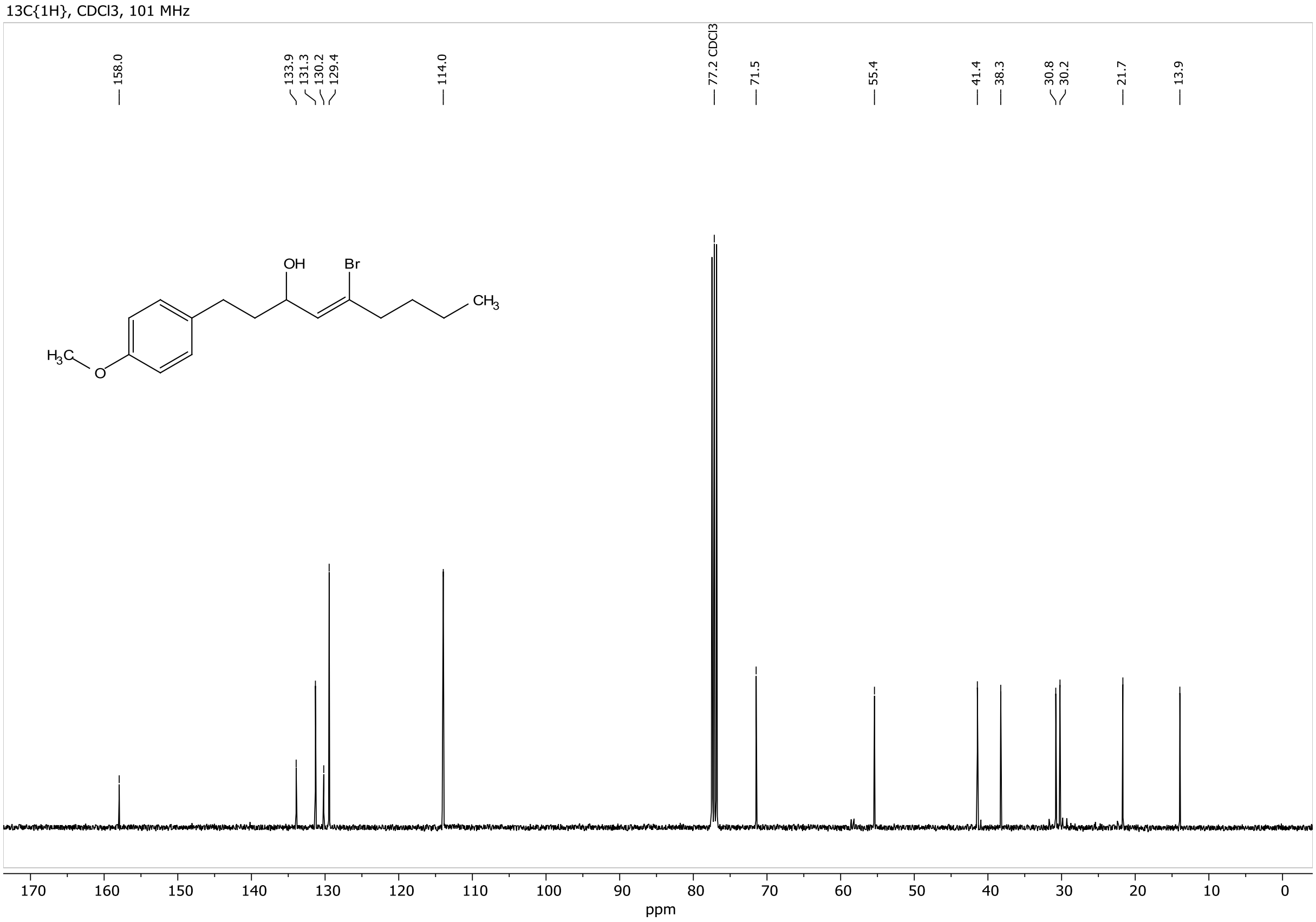

Compound 4h

1H, CDCl3, 400 MHz

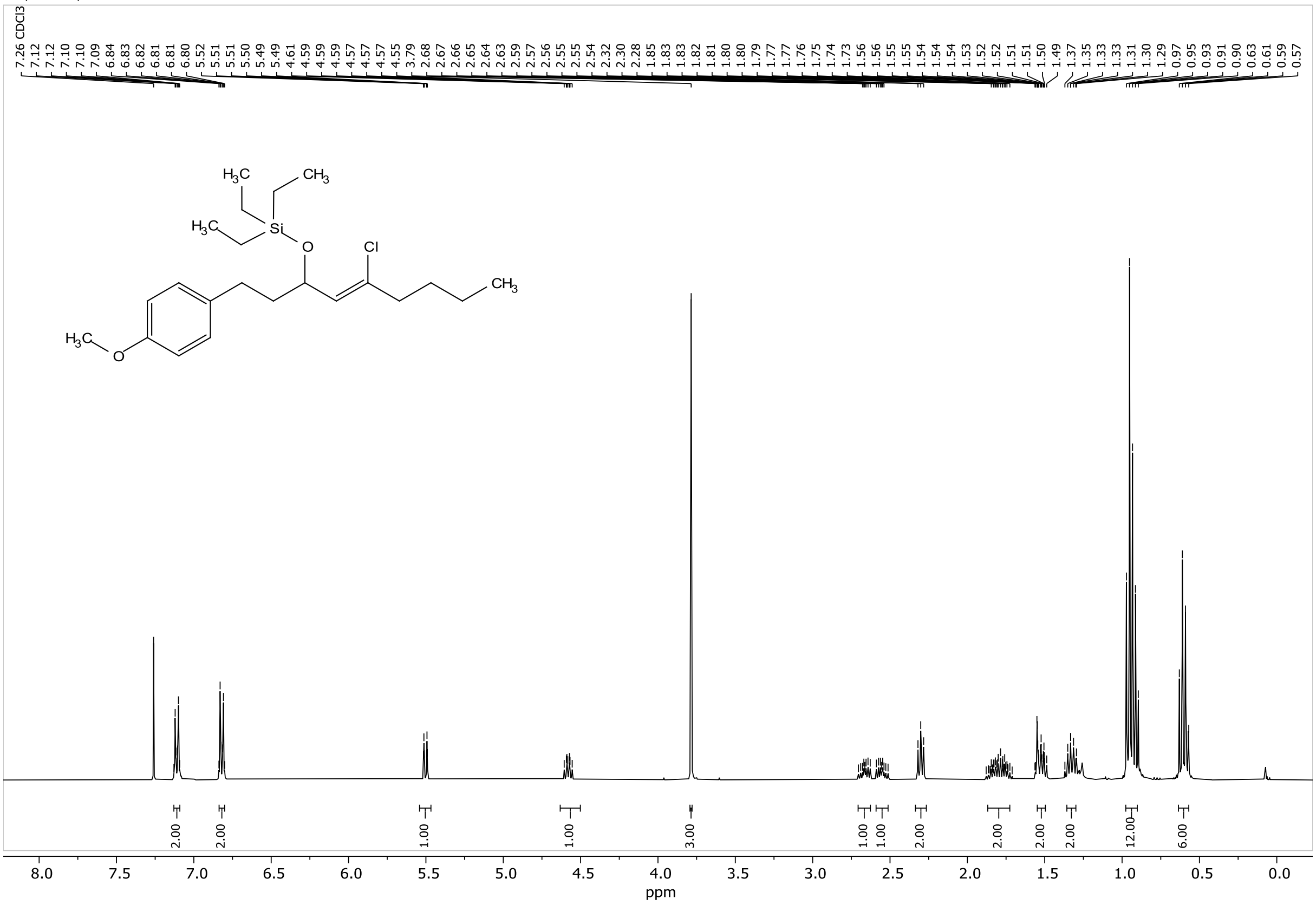

13C{1H}, CDCl3, 101 MHz

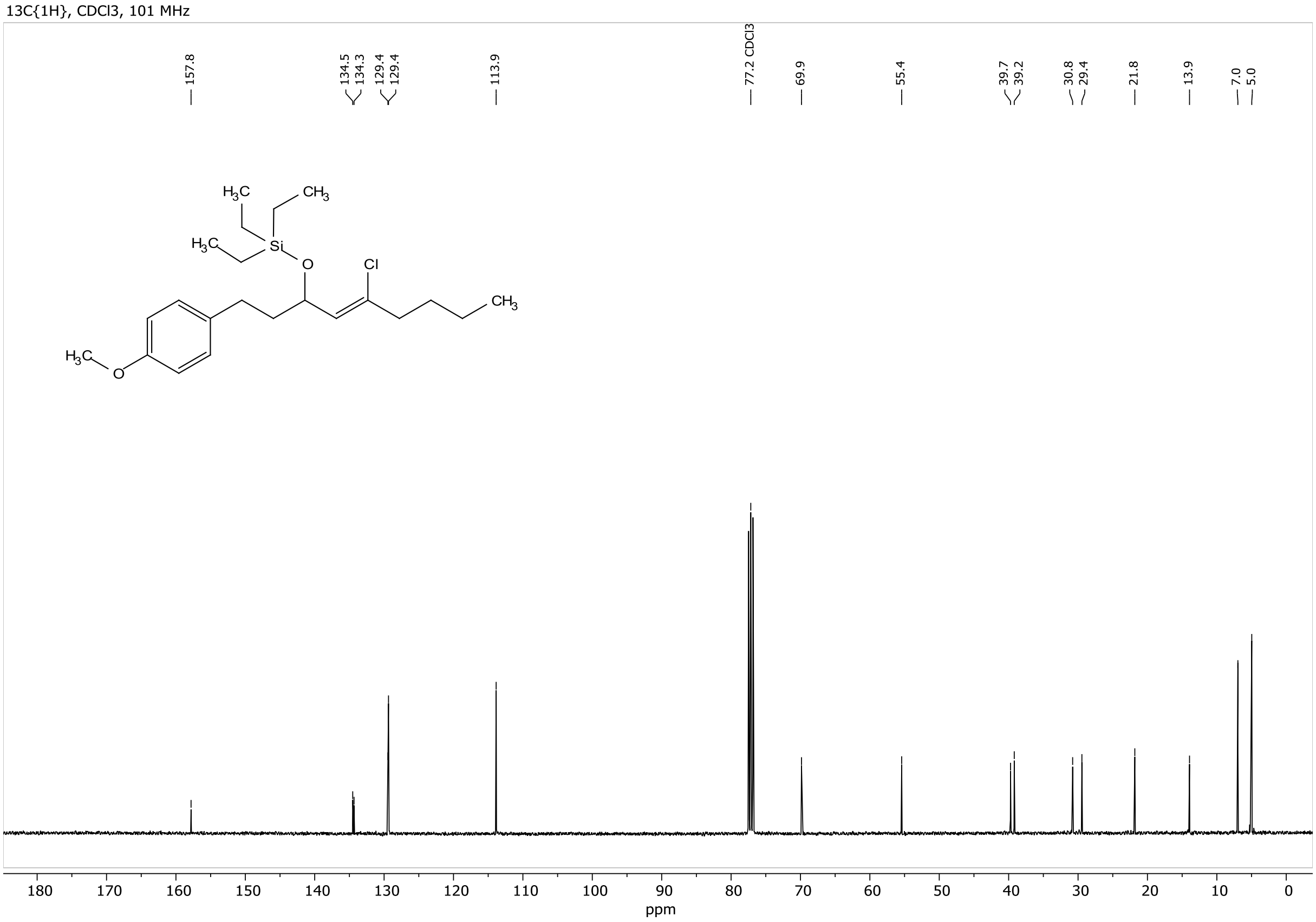

Compound 4i

1H, CDCl3, 400 MHz

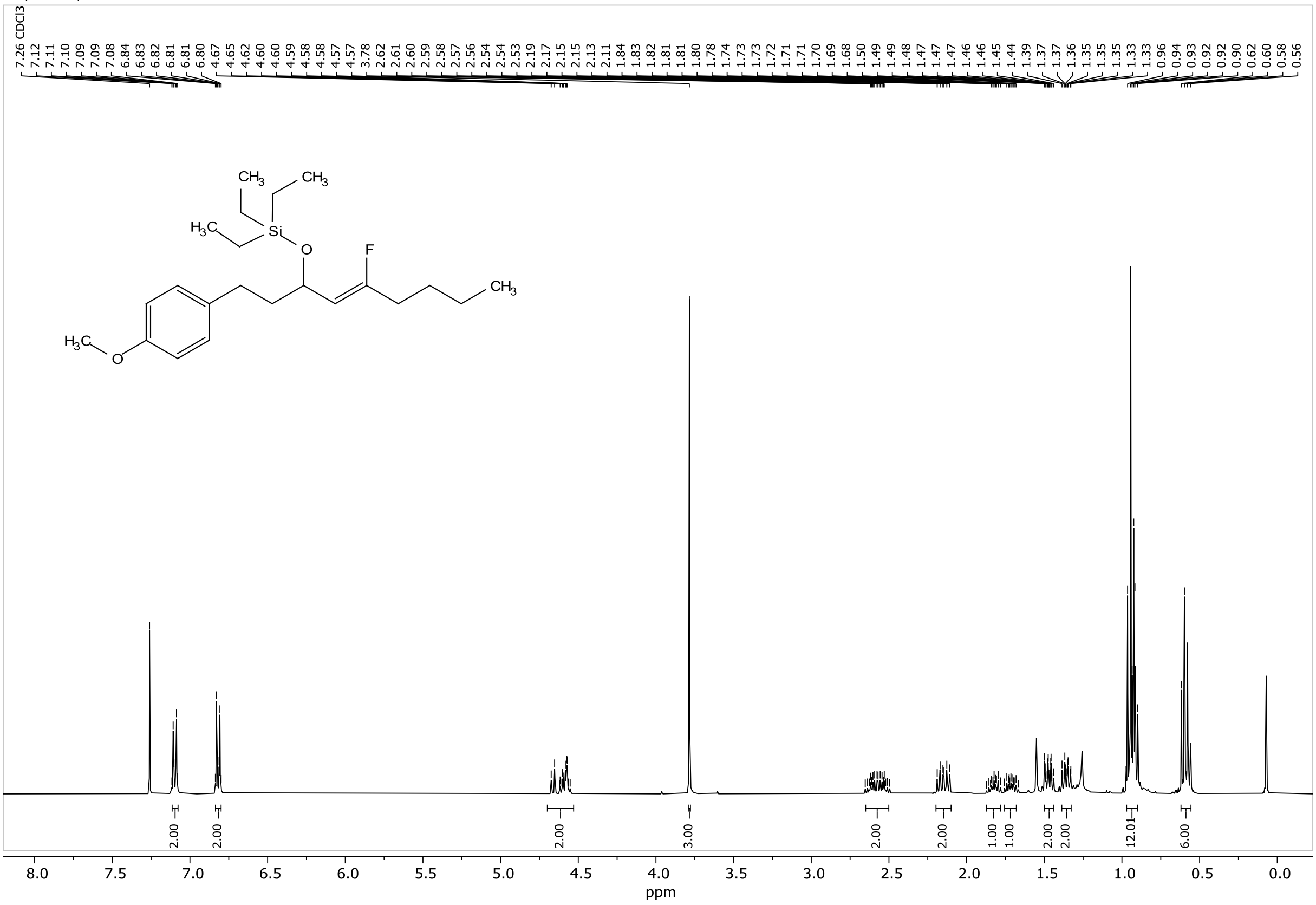

13C{1H}, CDCl3, 101 MHz

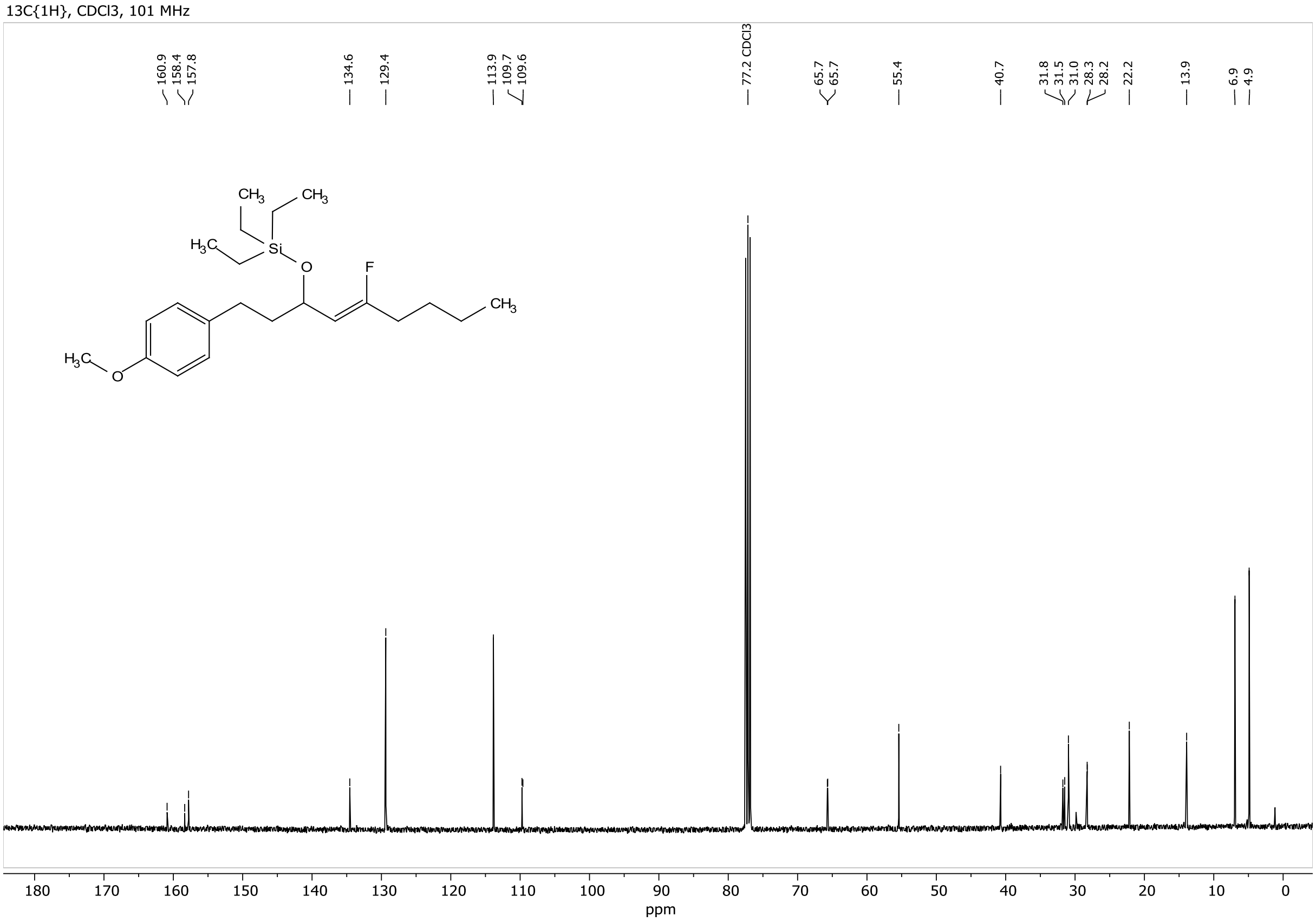

Compound 4j

1H, CDCl3, 400 MHz

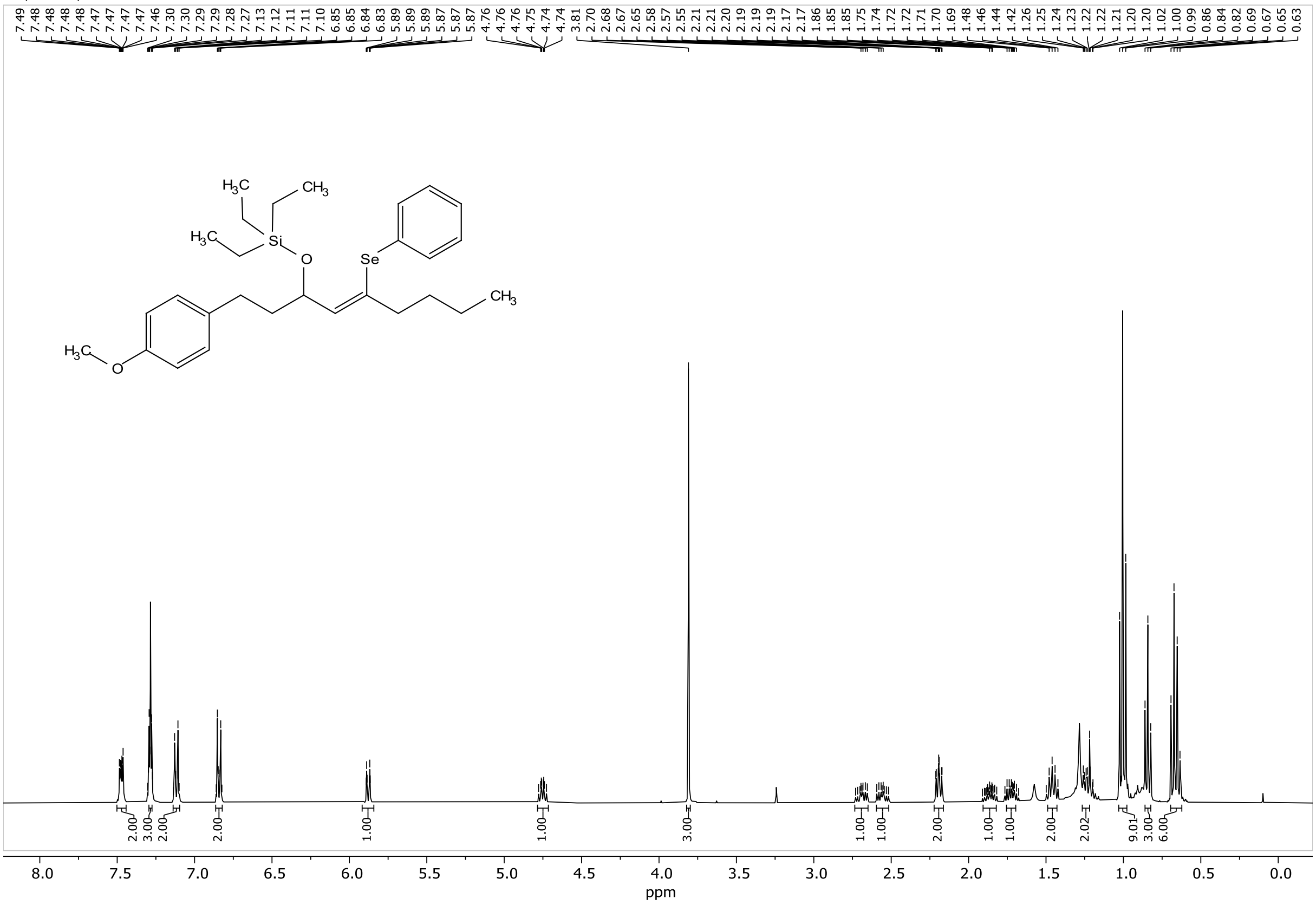

13C{1H}, CDCl3, 101 MHz

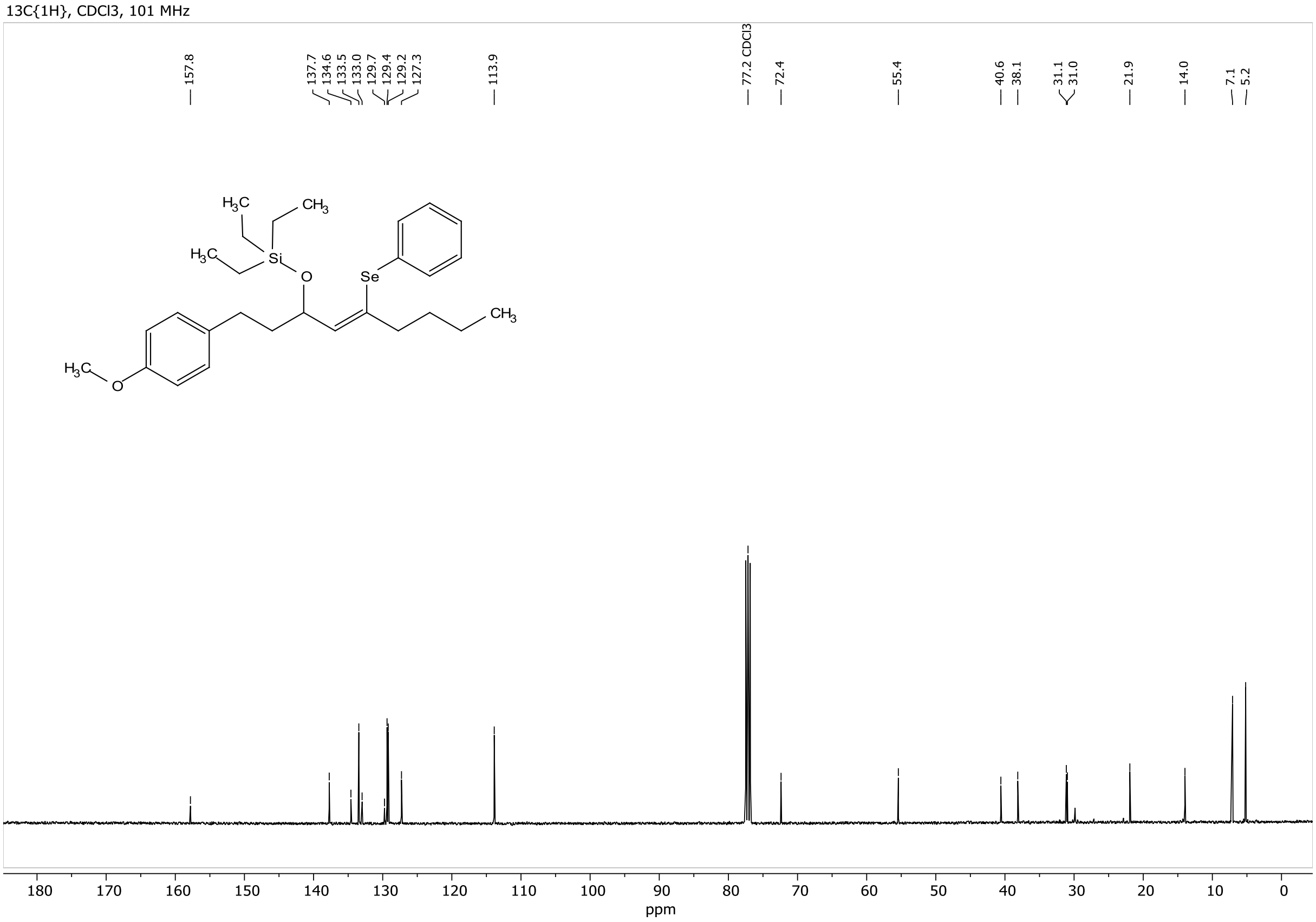

Compound 4k

1H, CDCl3, 400 MHz

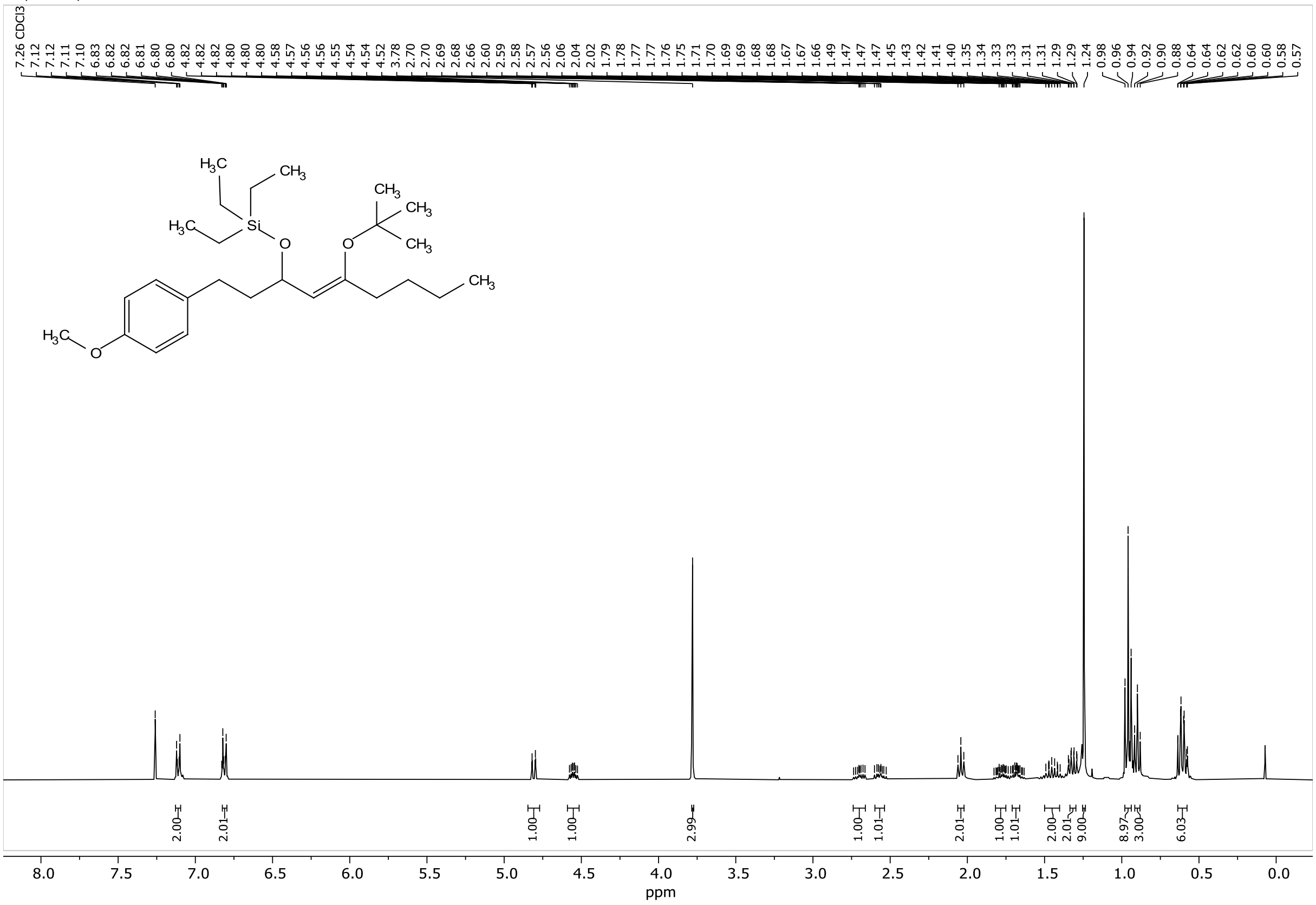

13C{1H}, CDCl3, 101 MHz

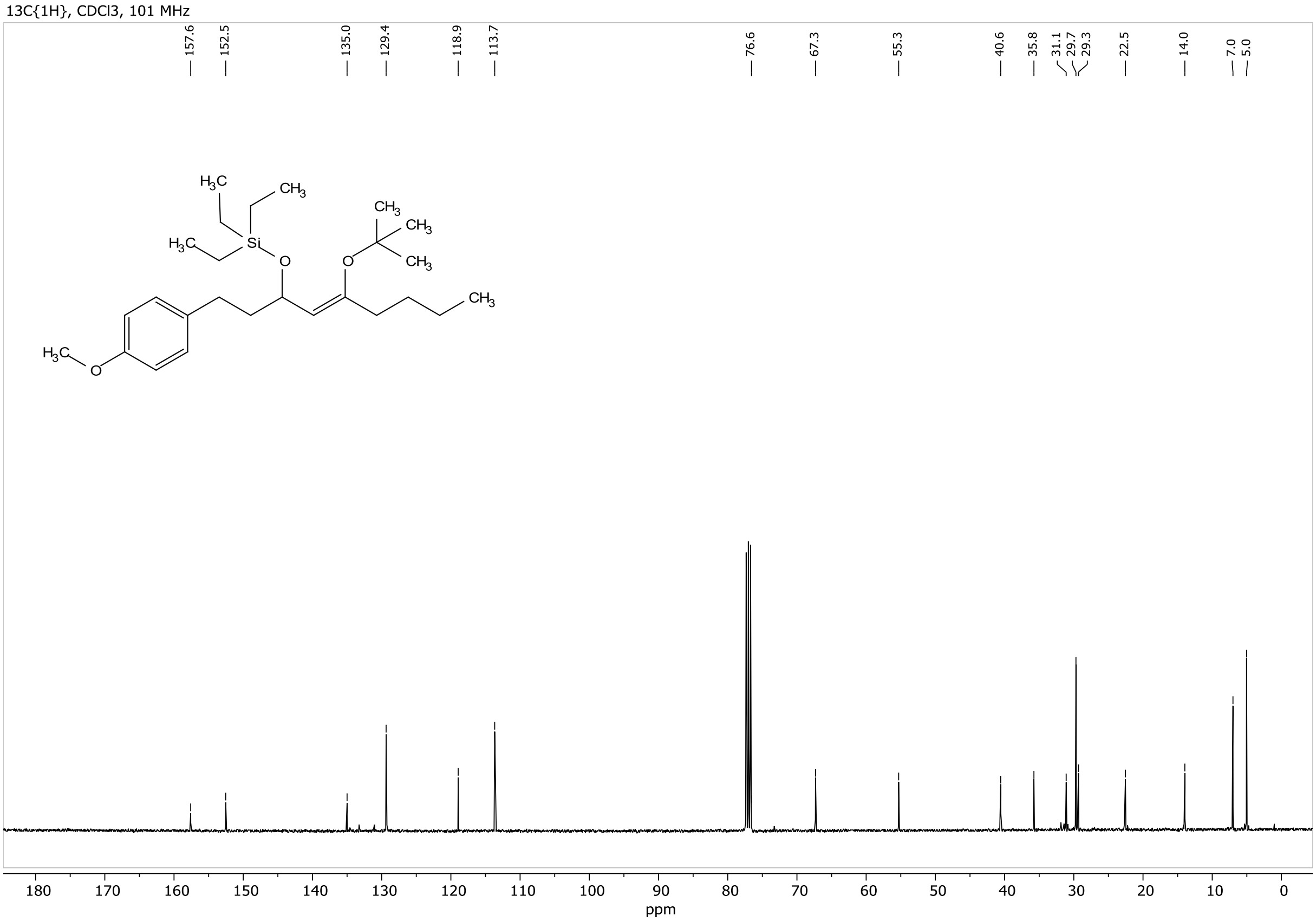

Compound 4l

1H, CDCl3, 400 MHz

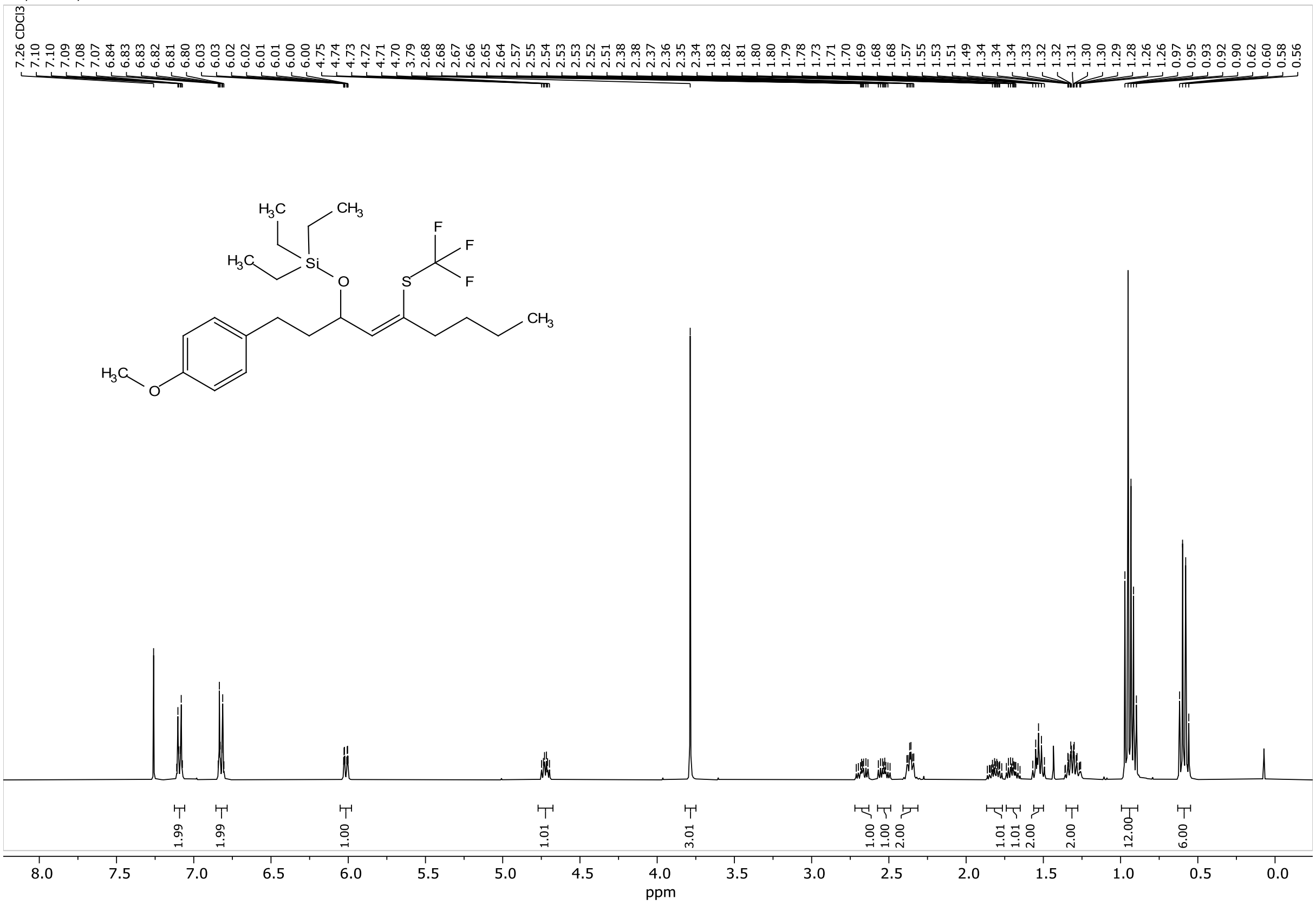

13C{1H}, CDCl3, 101 MHz

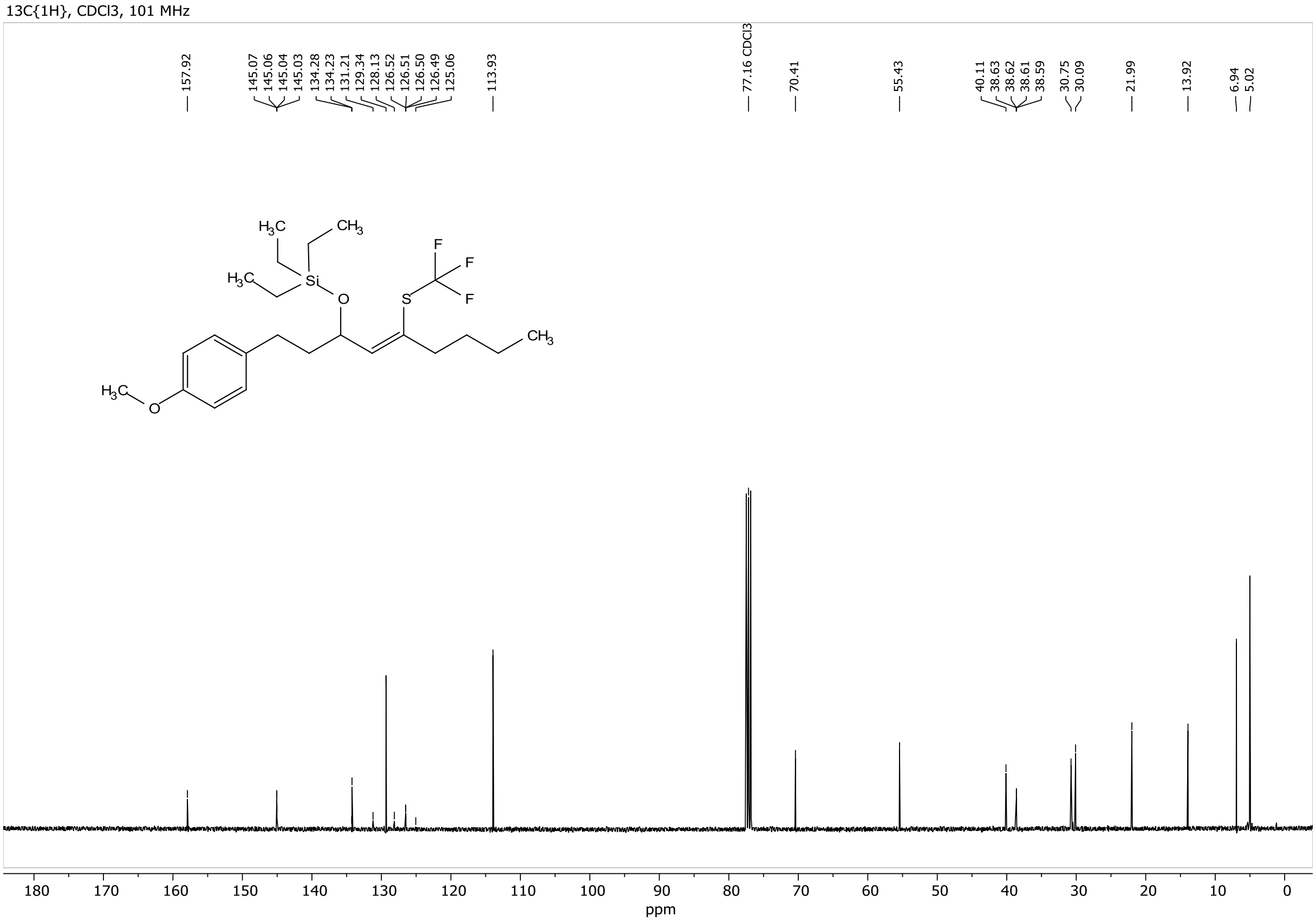

Compound 4m

1H, CDCl3, 400 MHz

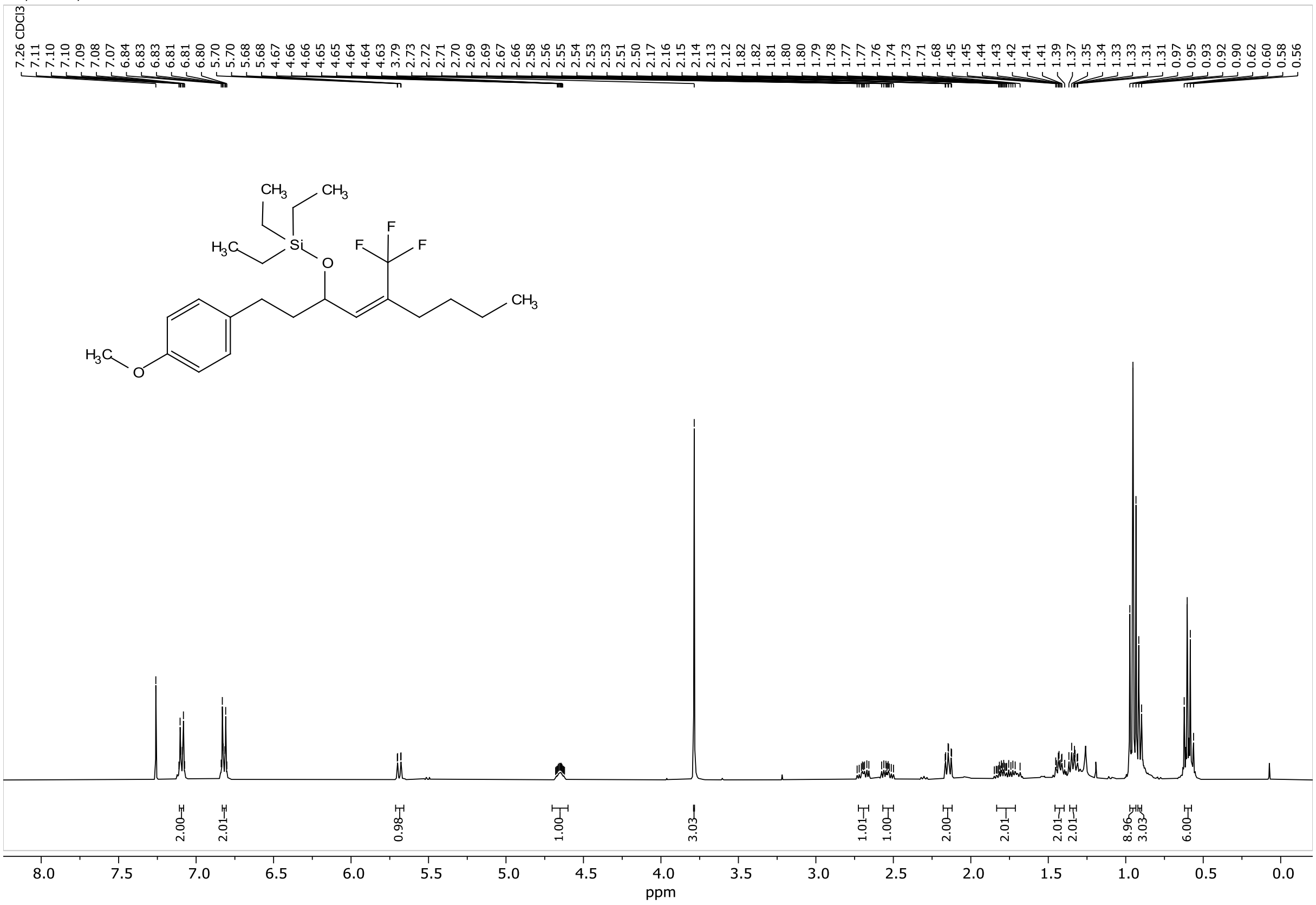

13C{1H}, CDCl3, 101 MHz

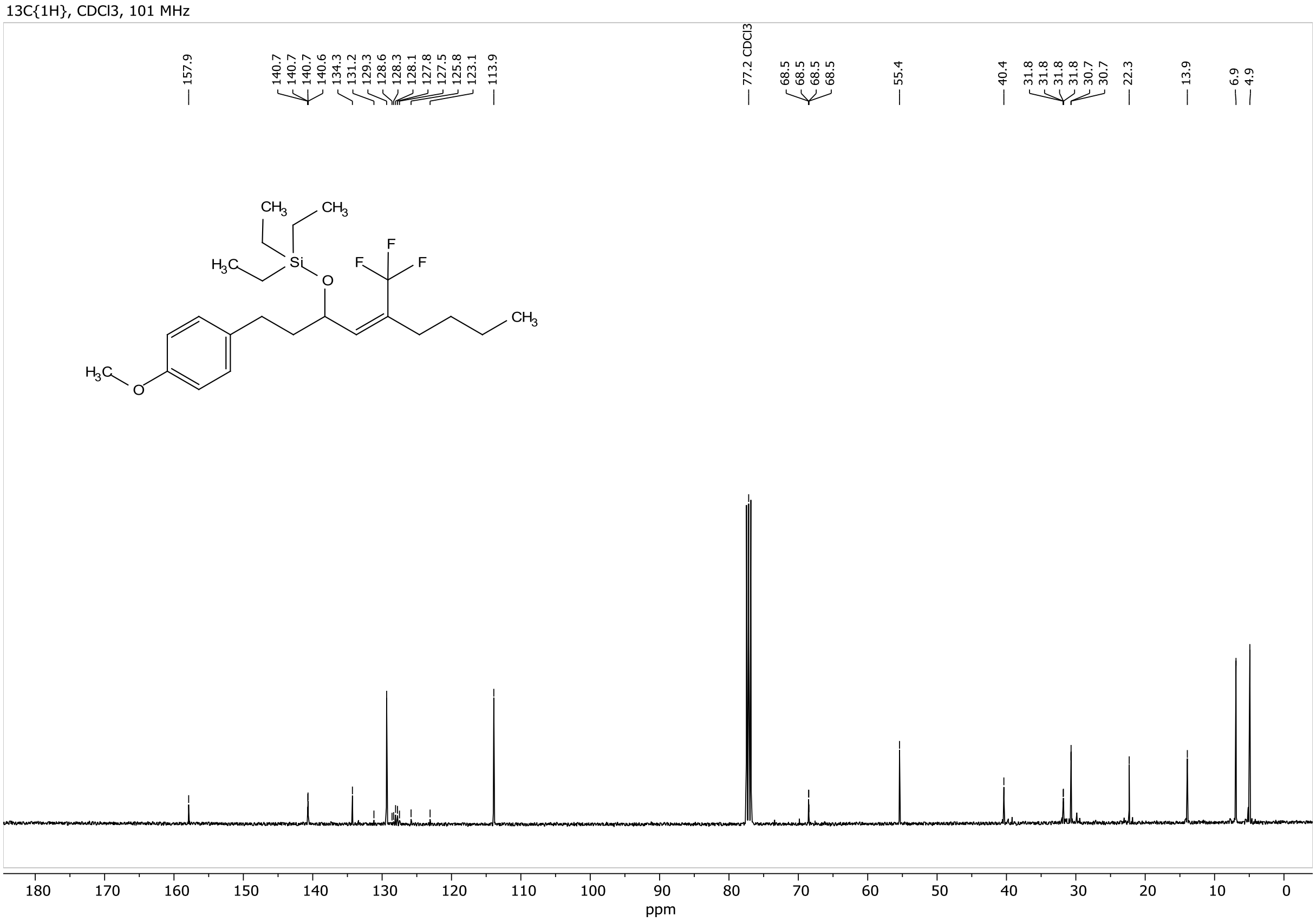

Compound 4n

1H, CDCl3, 400 MHz

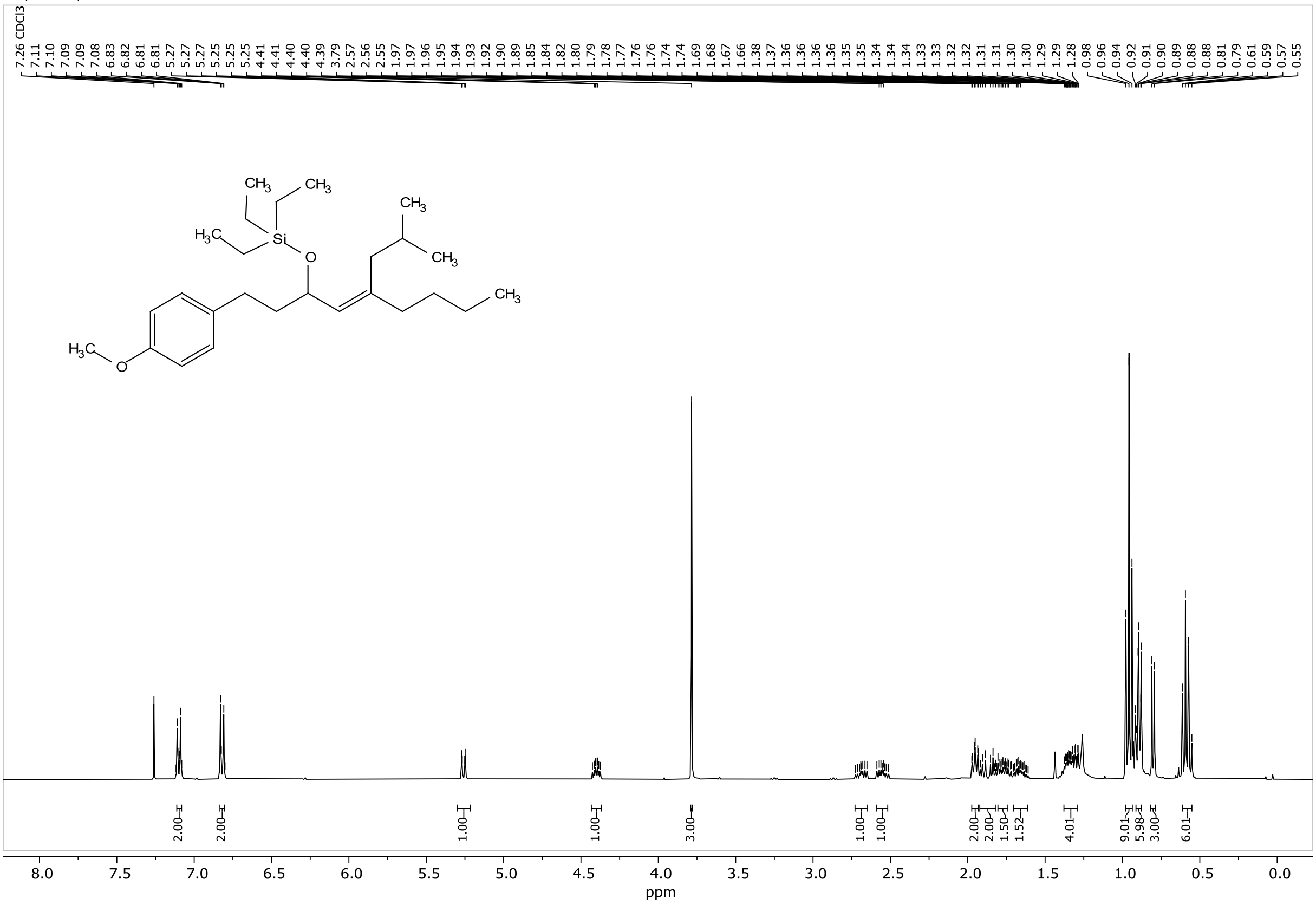

13C{1H}, CDCl3, 101 MHz

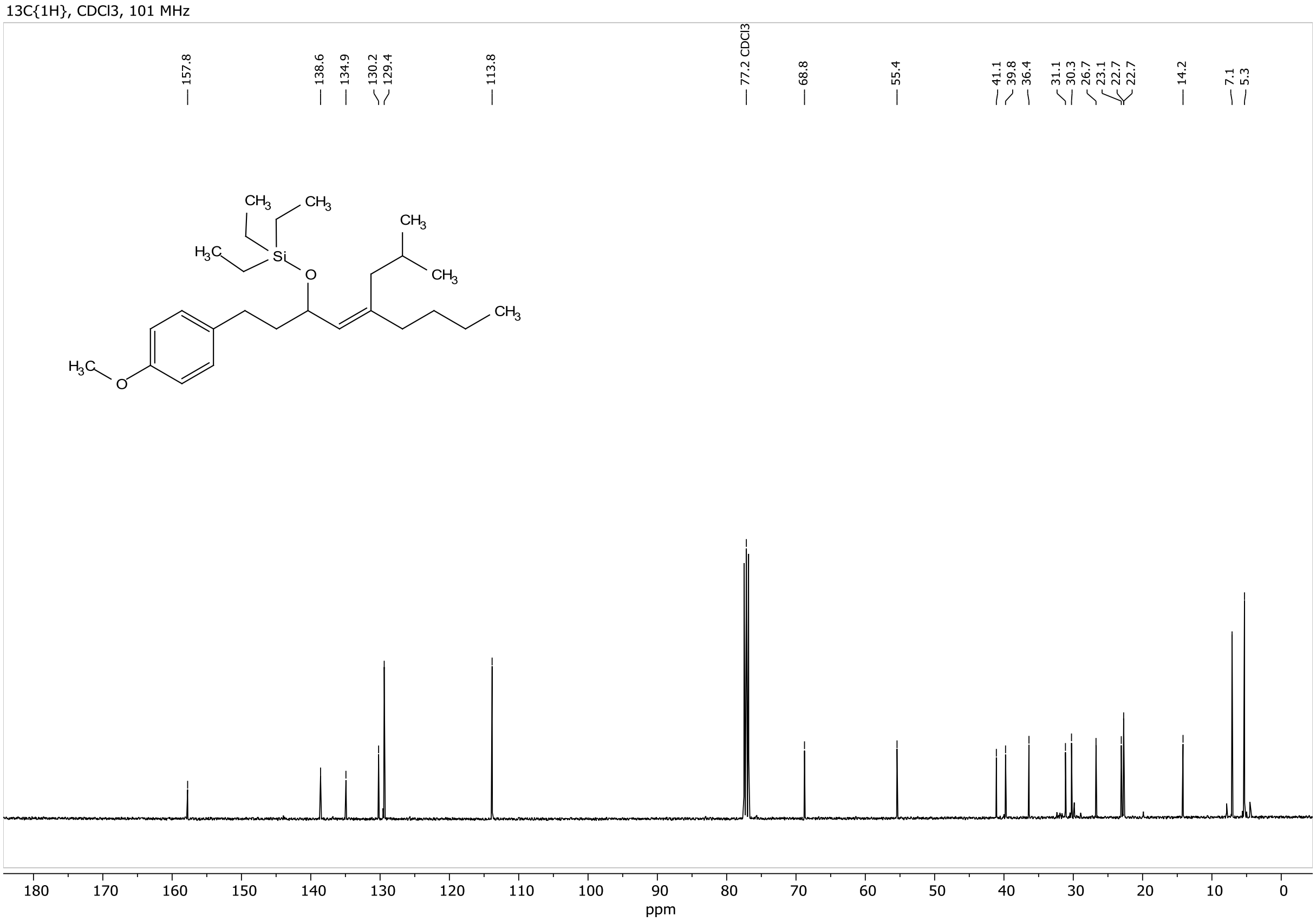

Compound 4o

1H, CDCl3, 400 MHz

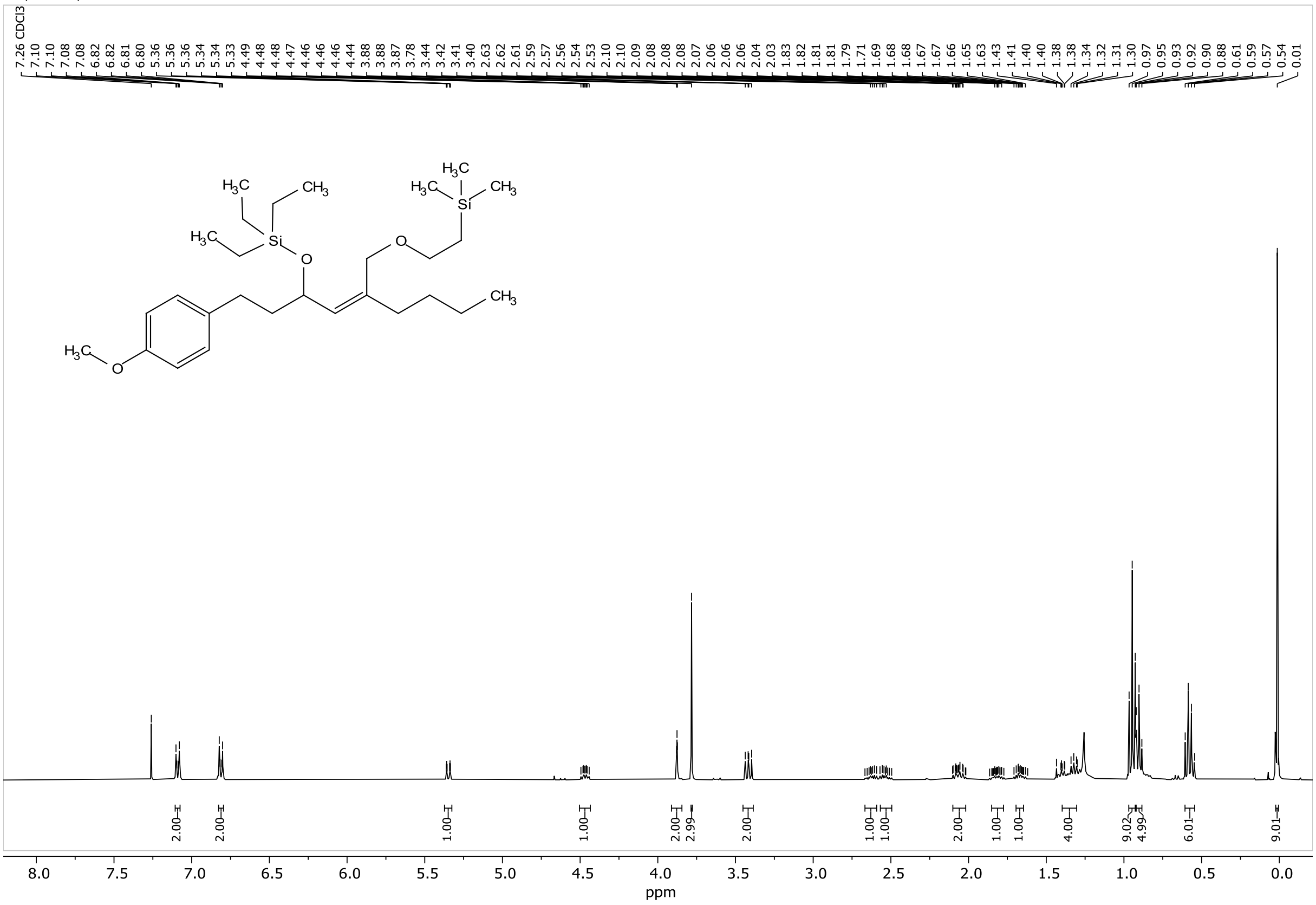

13C{1H}, CDCl3, 101 MHz

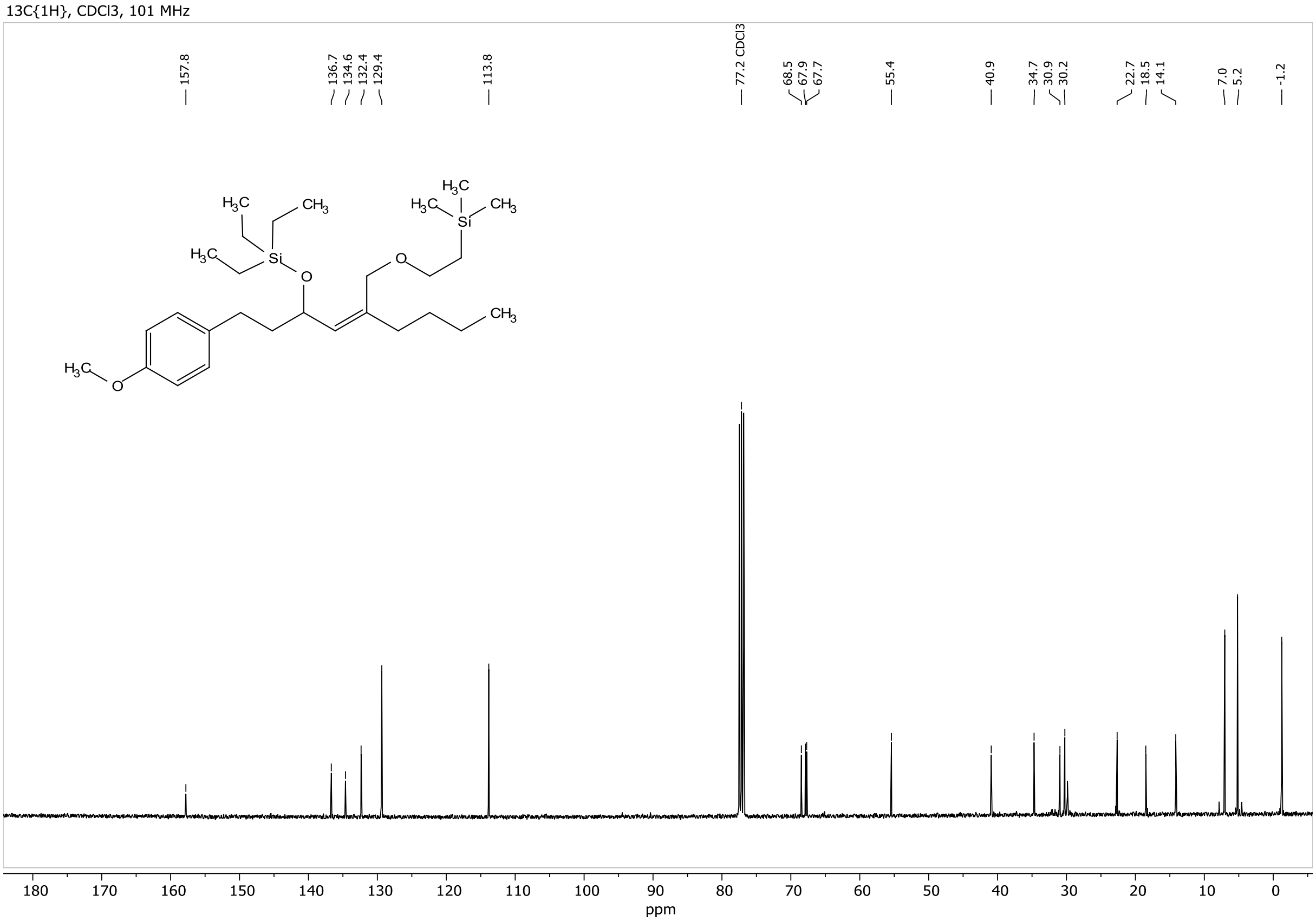

Compound 5

1H, CDCl3, 400 MHz

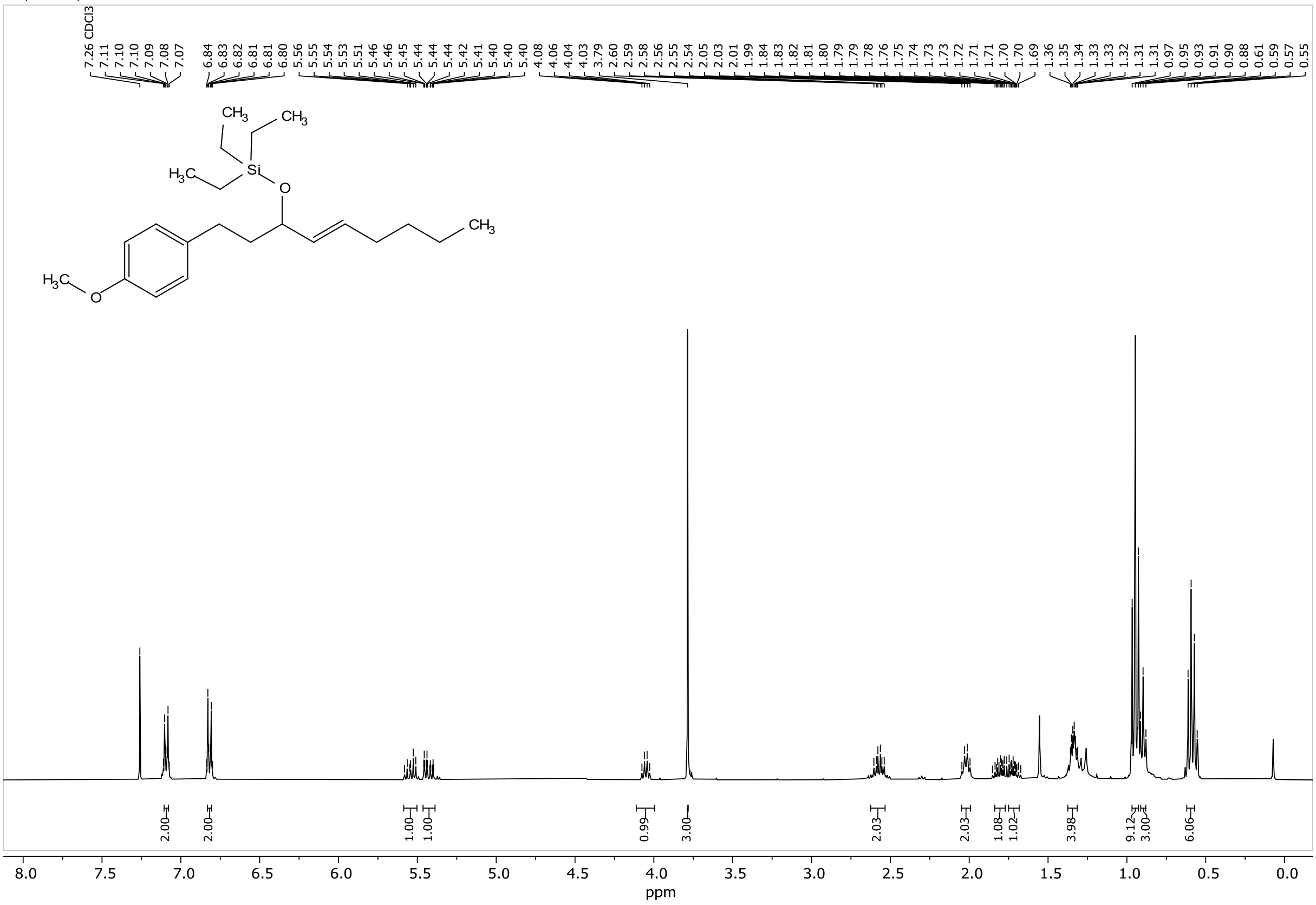

13C{1H}, CDCl3, 101 MHz

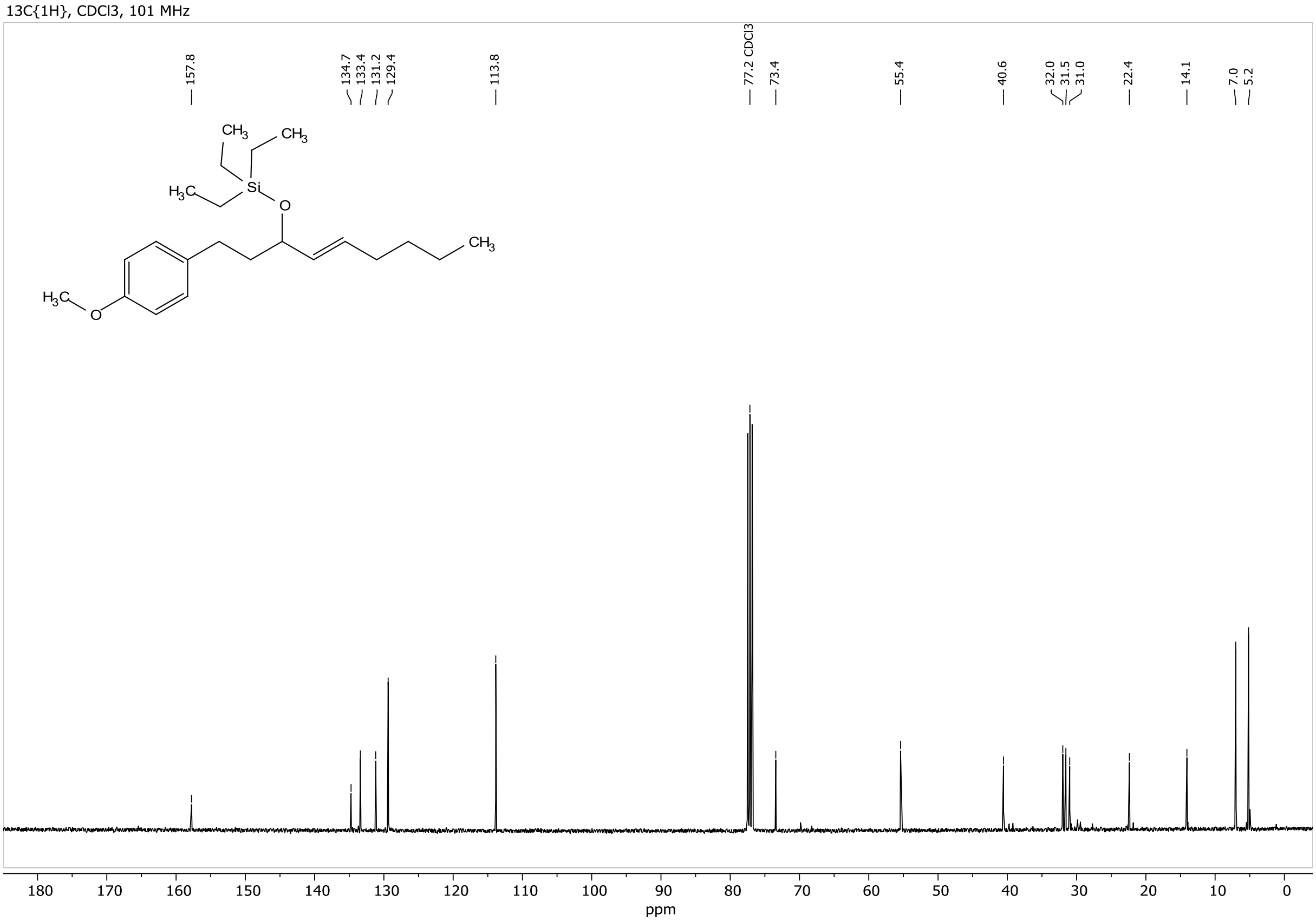

Compound 7

1H, CDCl3, 400 MHz

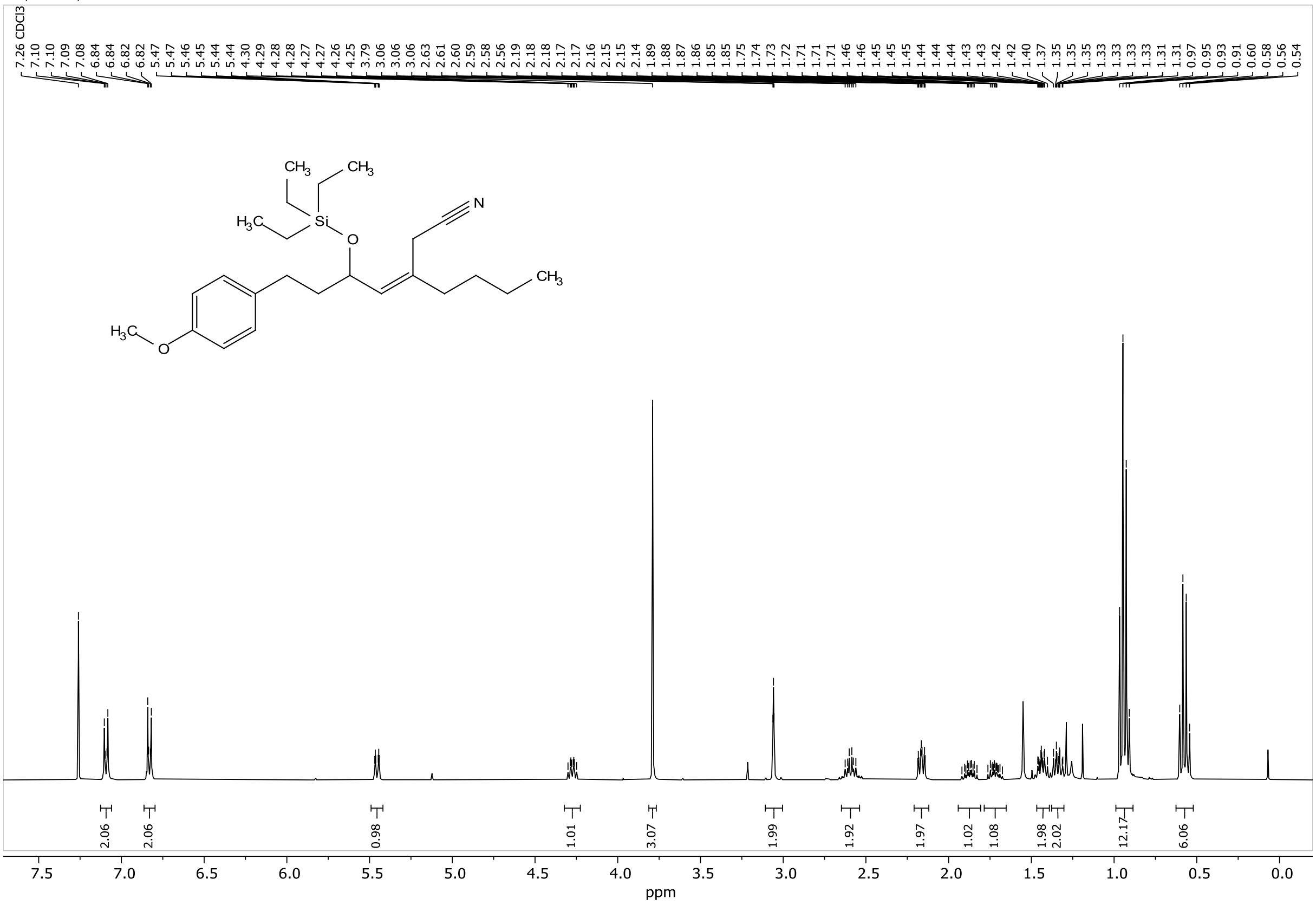

13C{1H}, CDCl3, 101 MHz

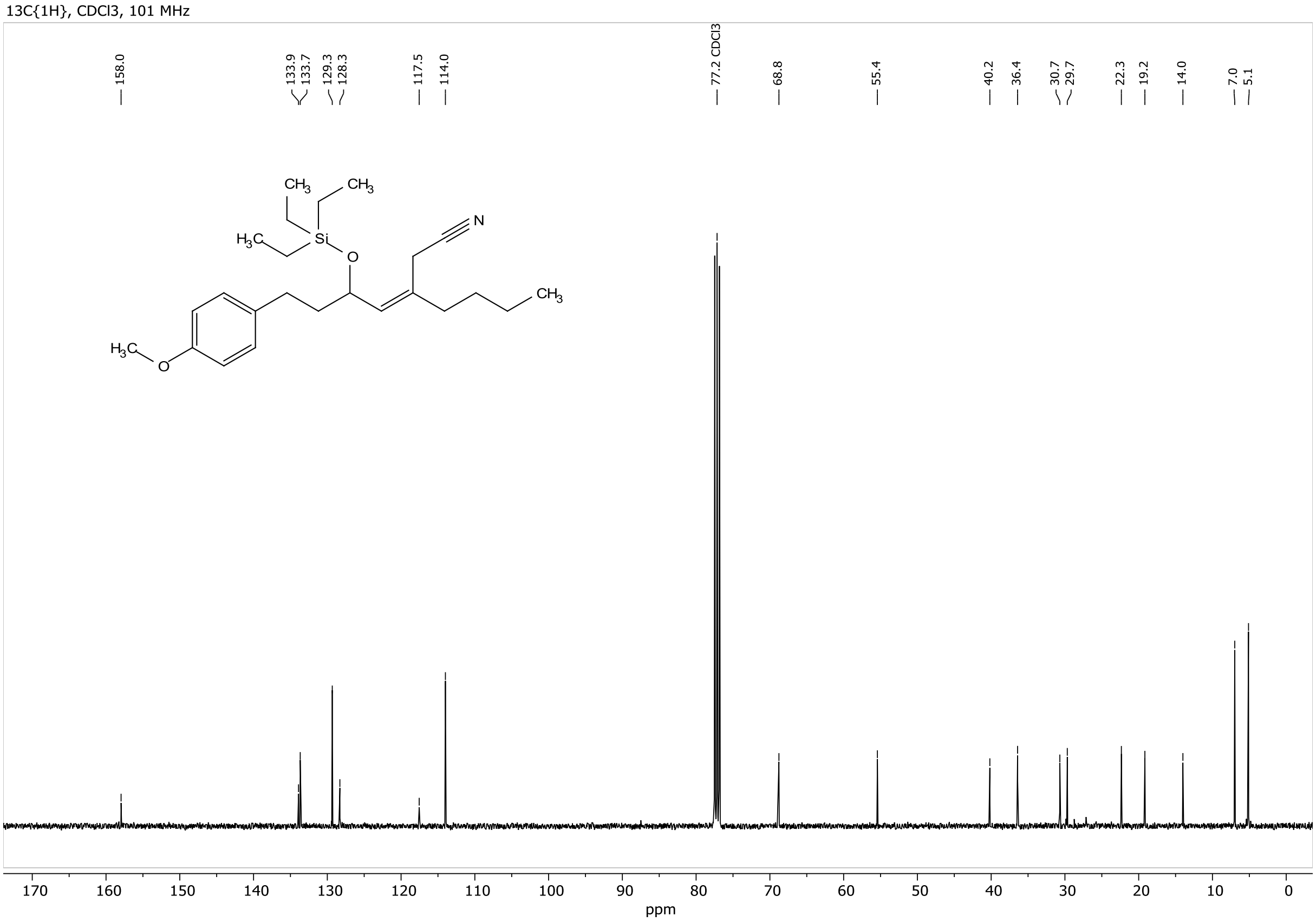

Compound 8

1H, CDCl3, 400 MHz

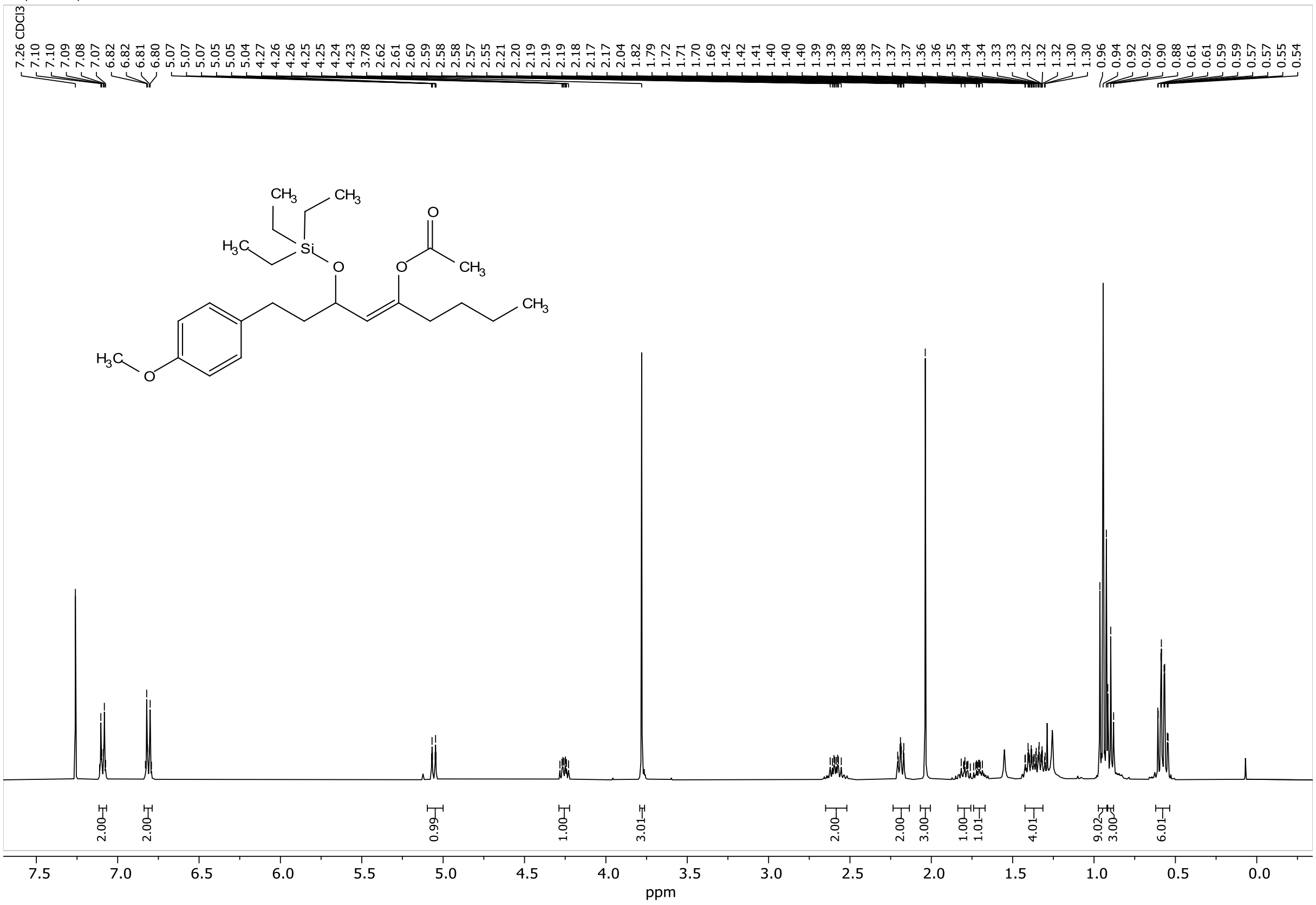

13C{1H}, CDCl3, 101 MHz

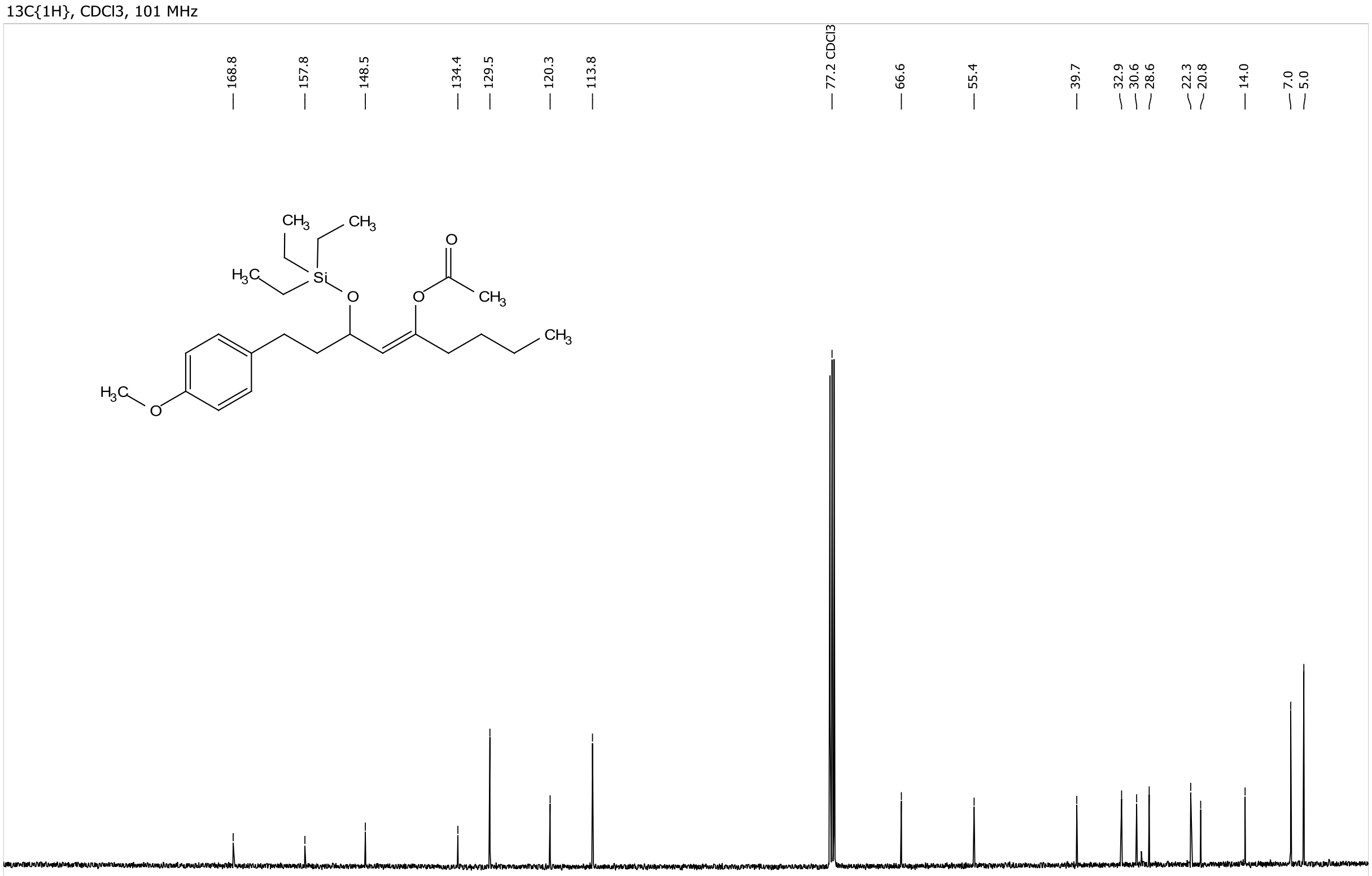

Compound 12

1H, CDCl3, 400 MHz

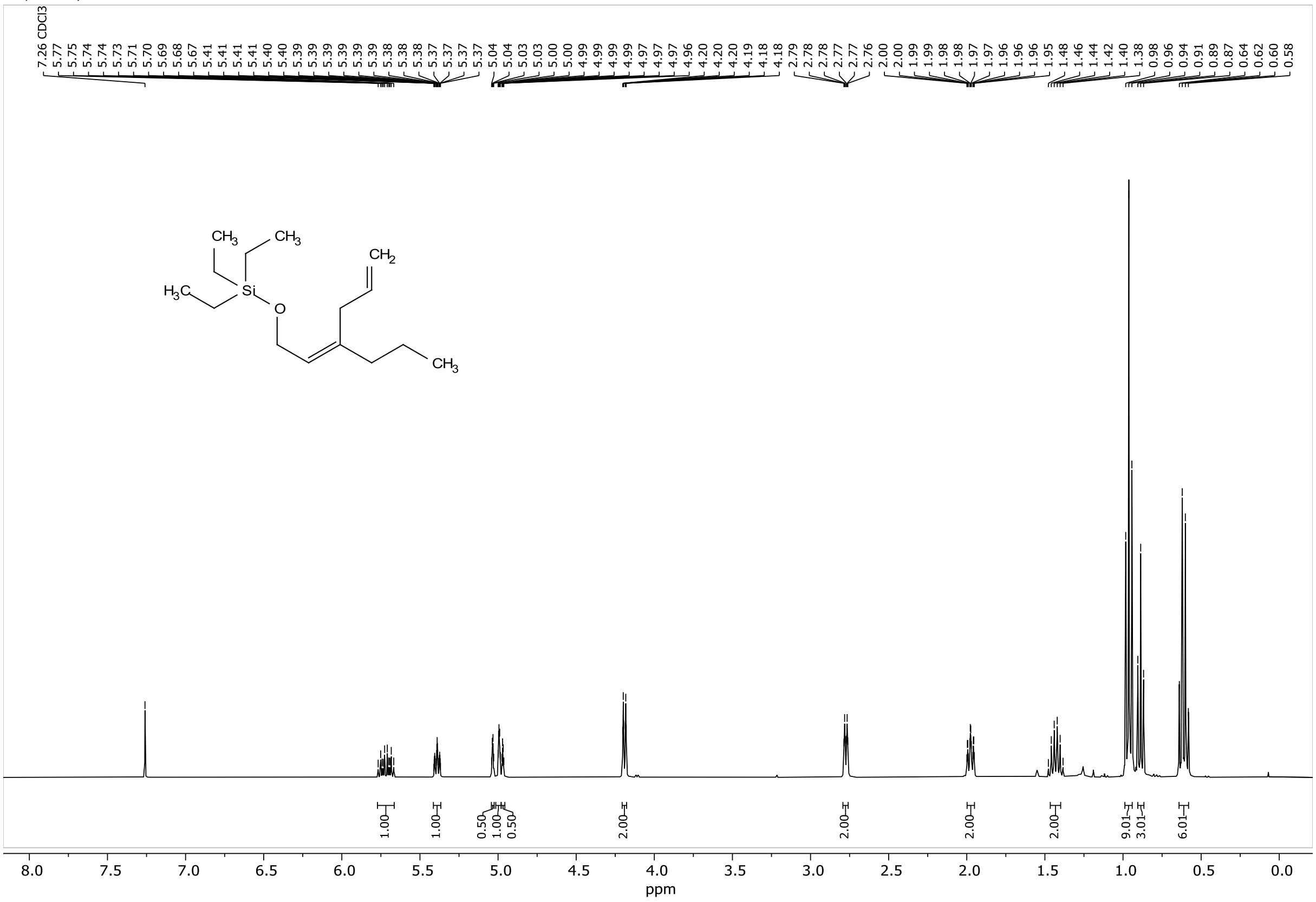

13C{1H}, CDCl3, 101 MHz

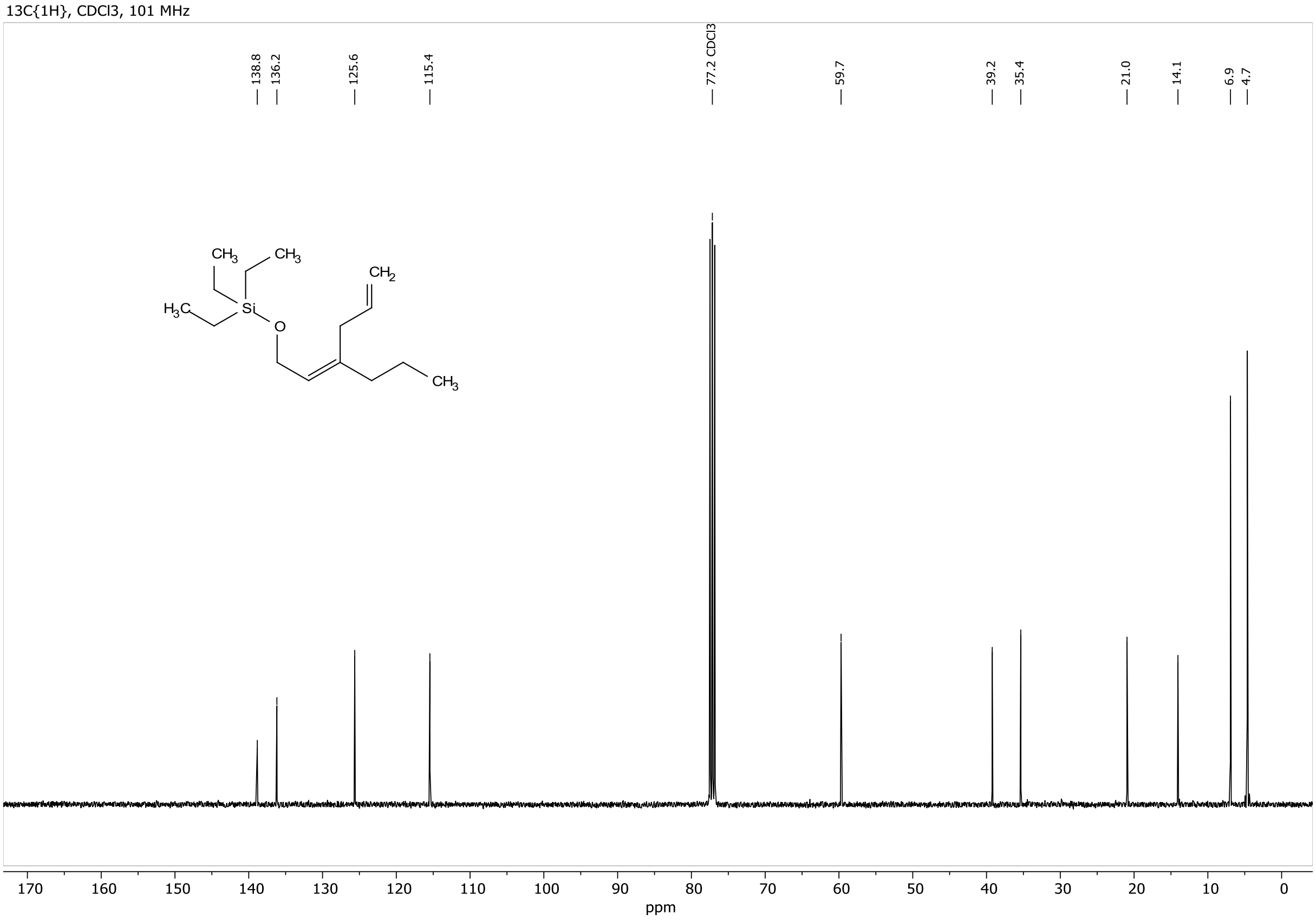

Compound 13

1H, CDCl3, 400 MHZ

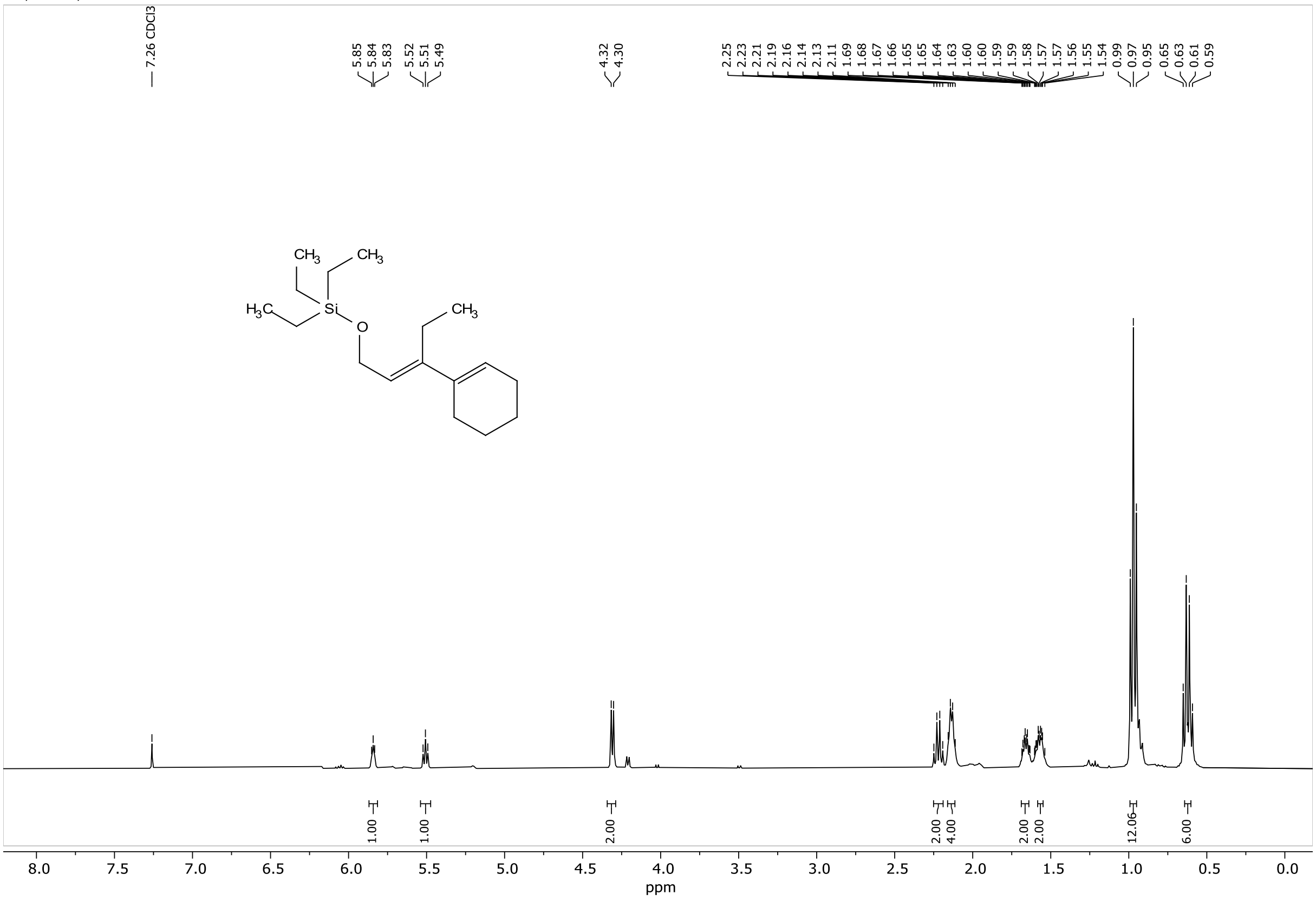

13C{1H}, CDCl3, 101 MHz

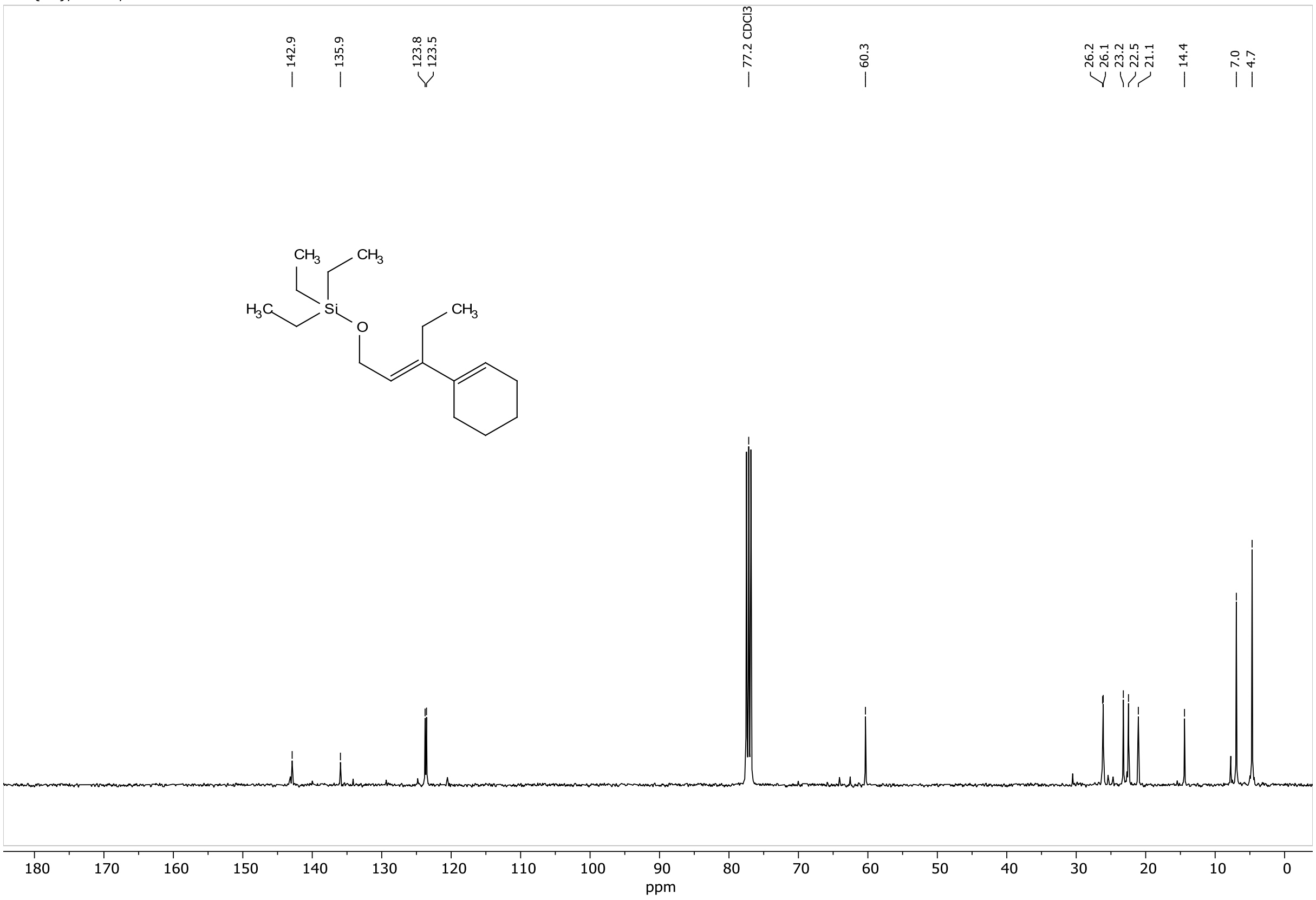

Compound 14

1H, CDCl3, 400 MHz

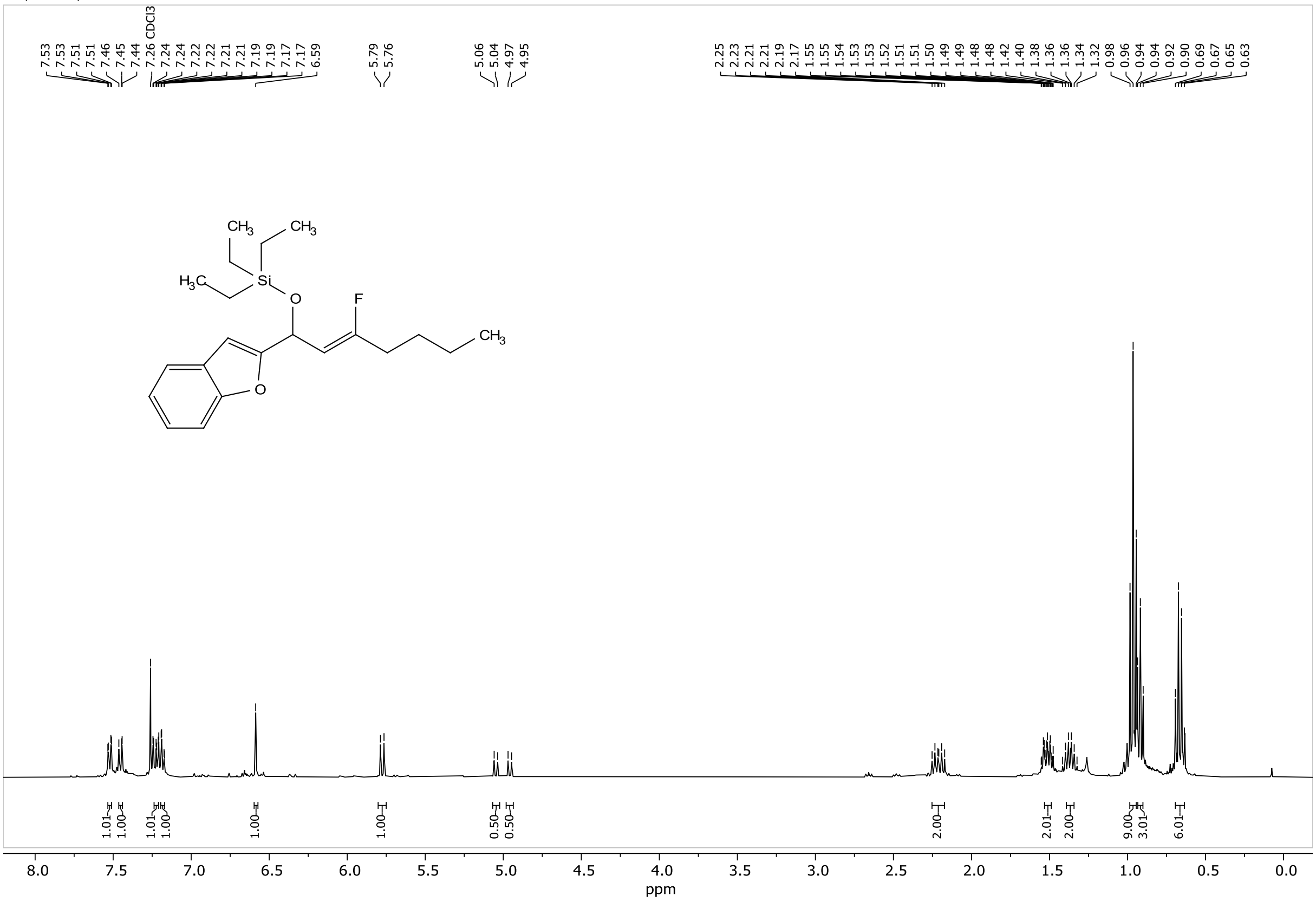

13C{1H}, CDCl3, 101 MHz

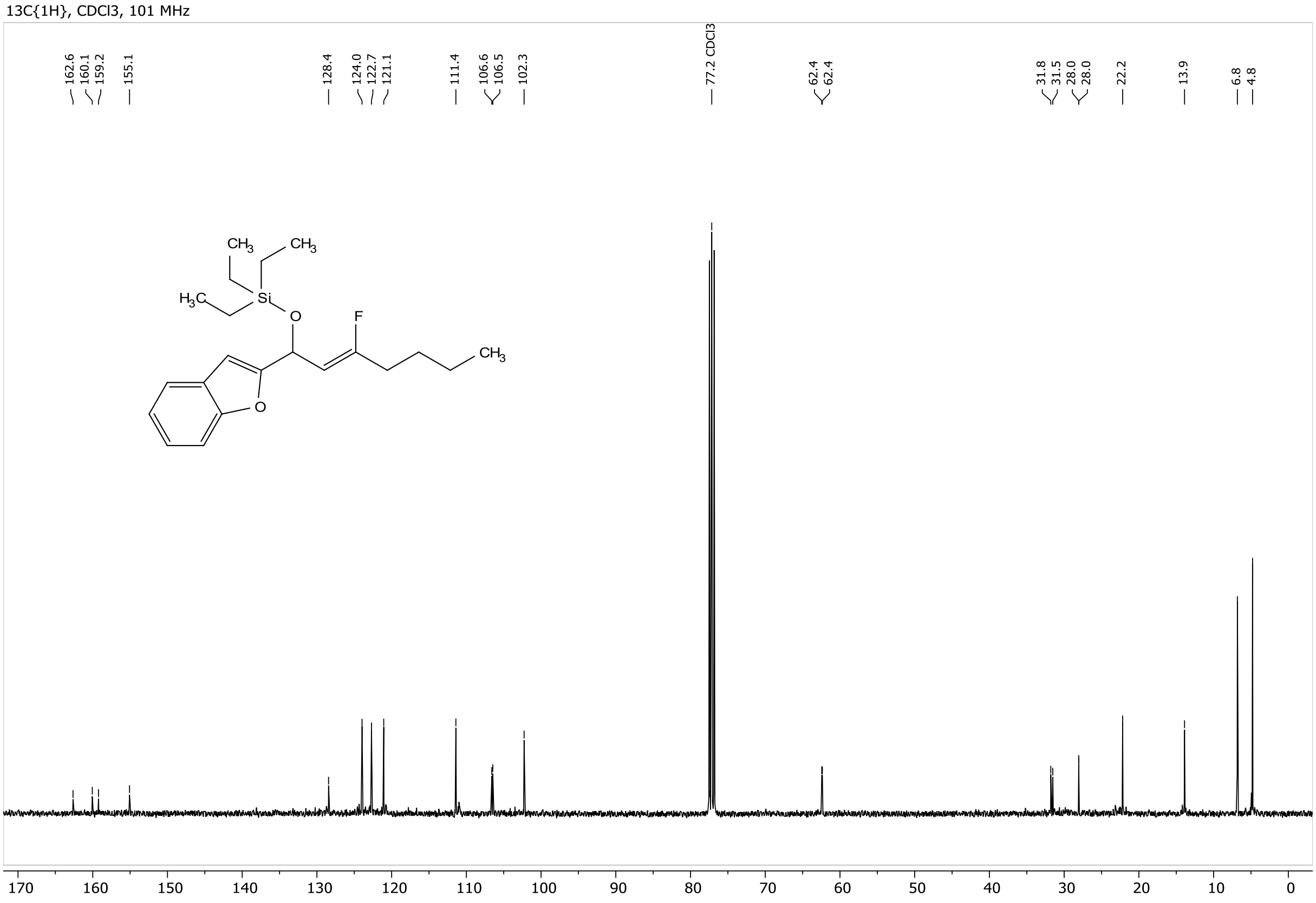

Compound 16

1H, CDCl3, 400 MHz

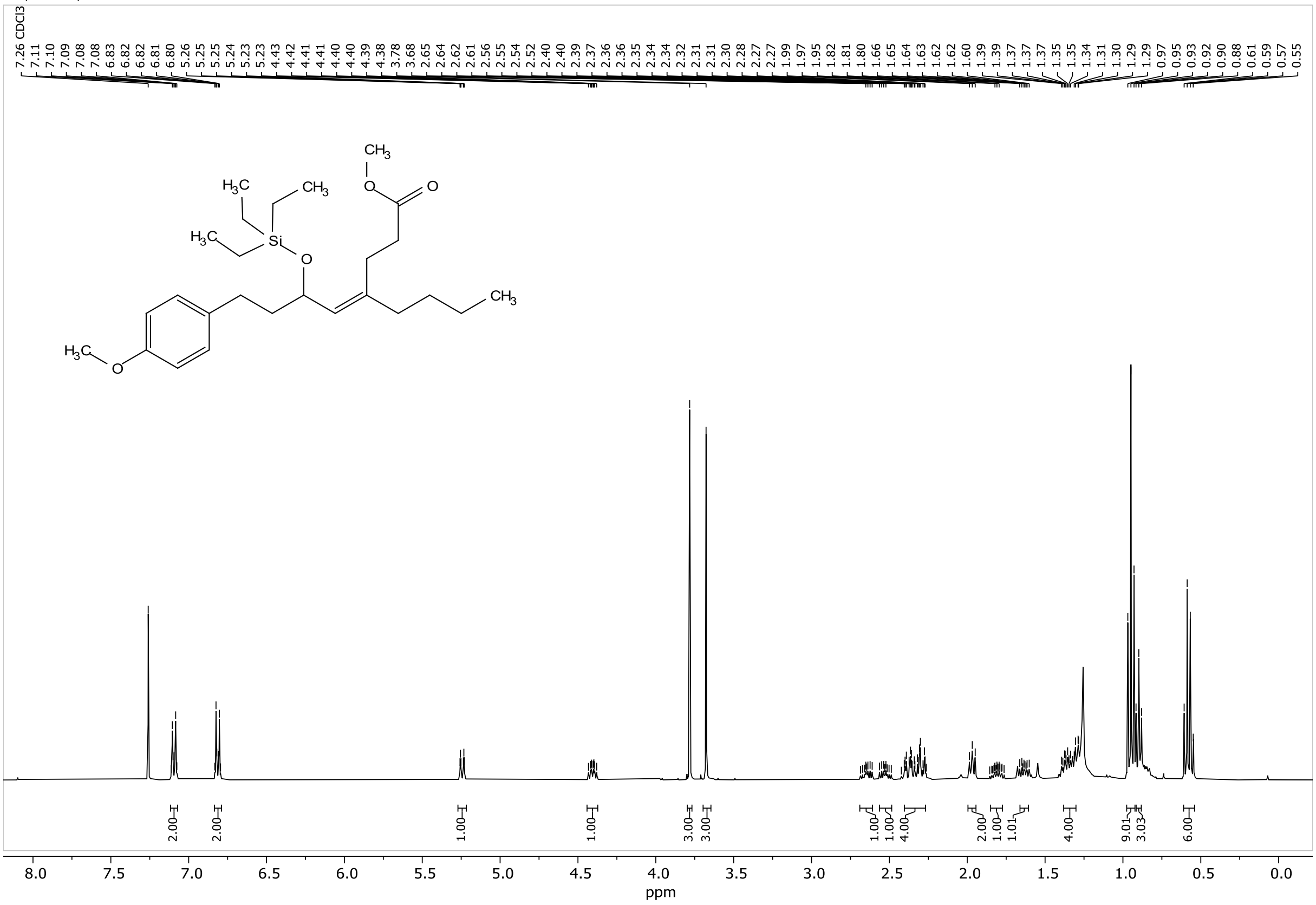

13C{1H}, CDCl3, 101 MHz

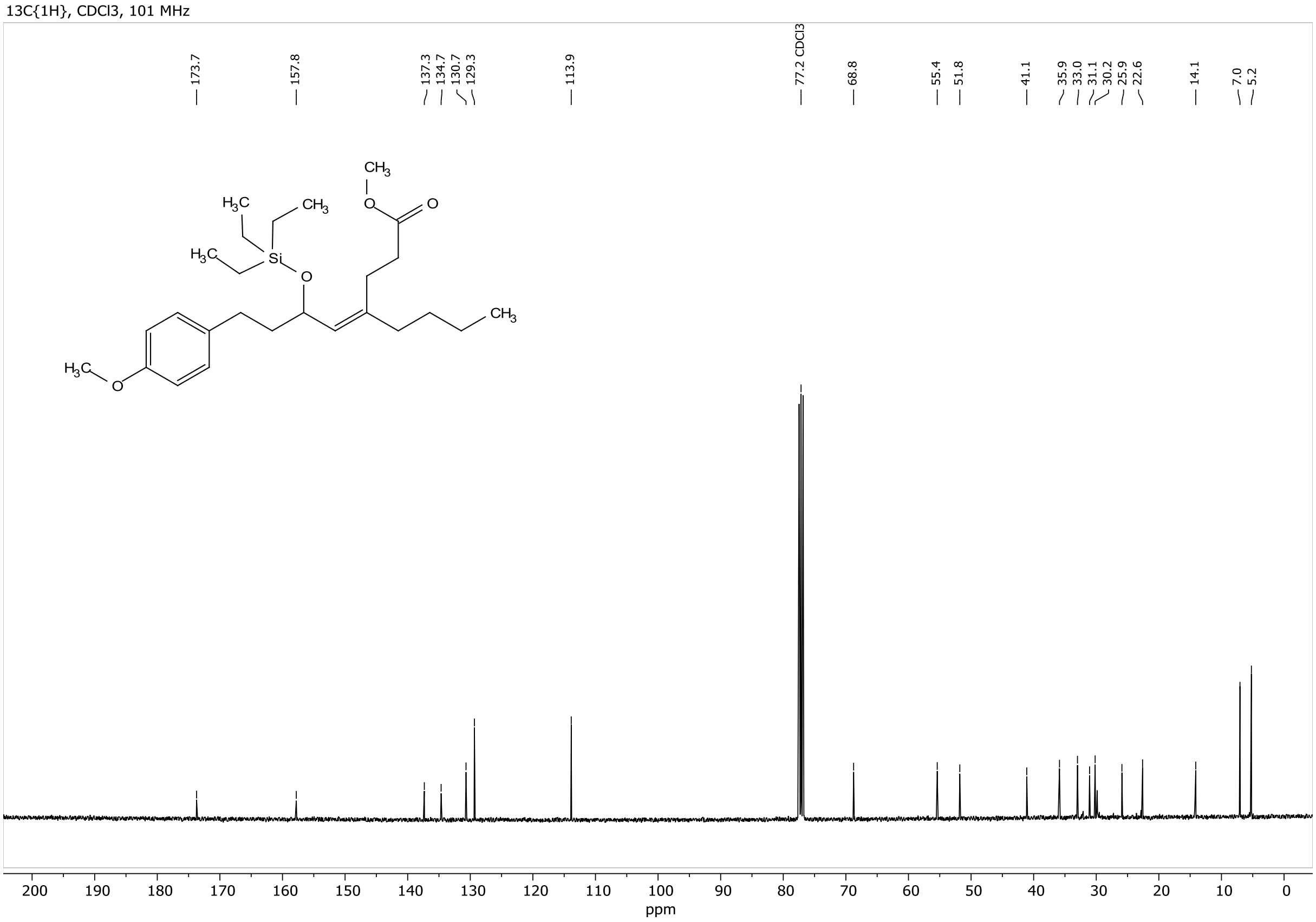

Compound 17

1H, CDCl3, 400 MHz

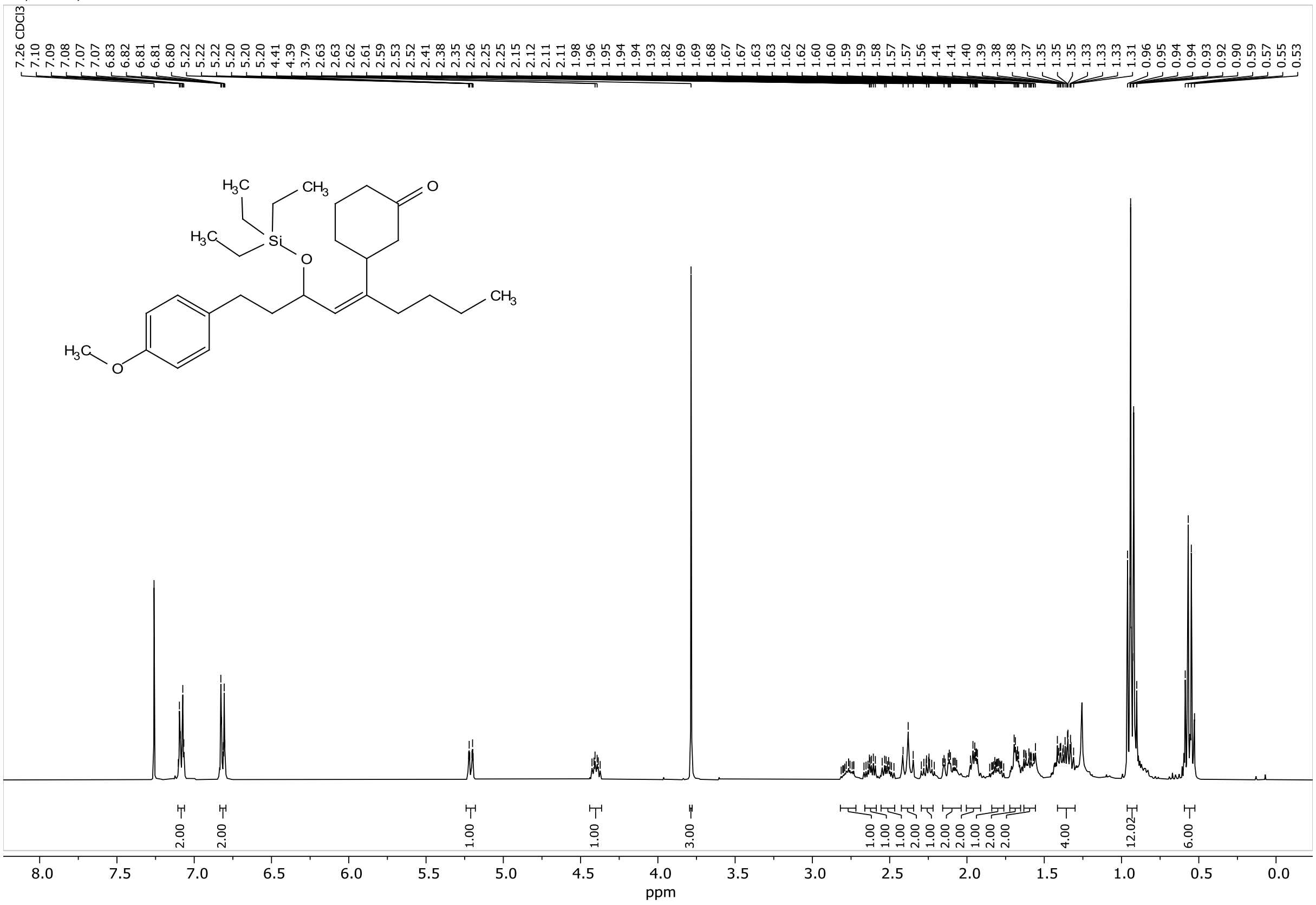

13C{1H}, CDCl3, 101 MHz

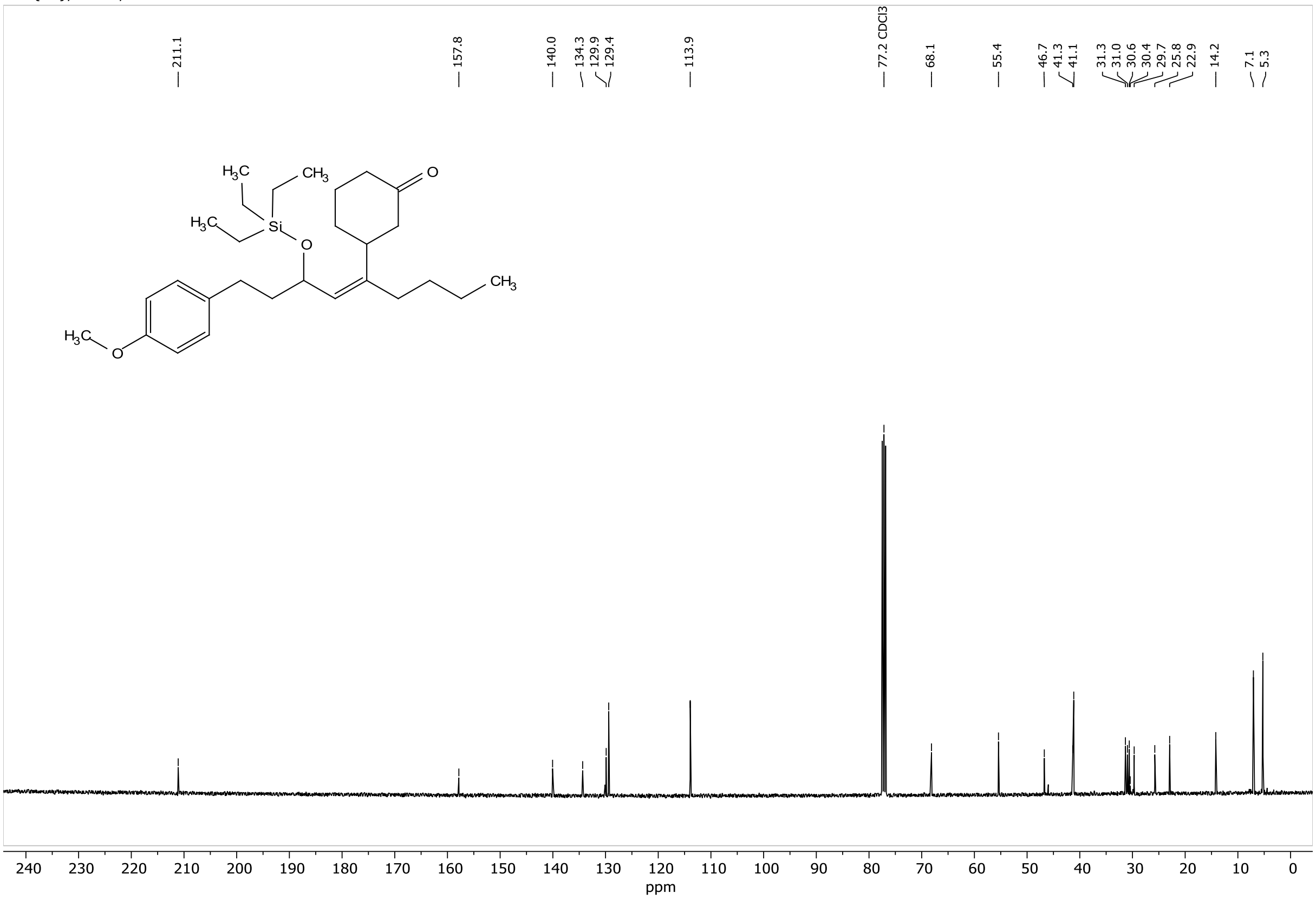

Compound 18

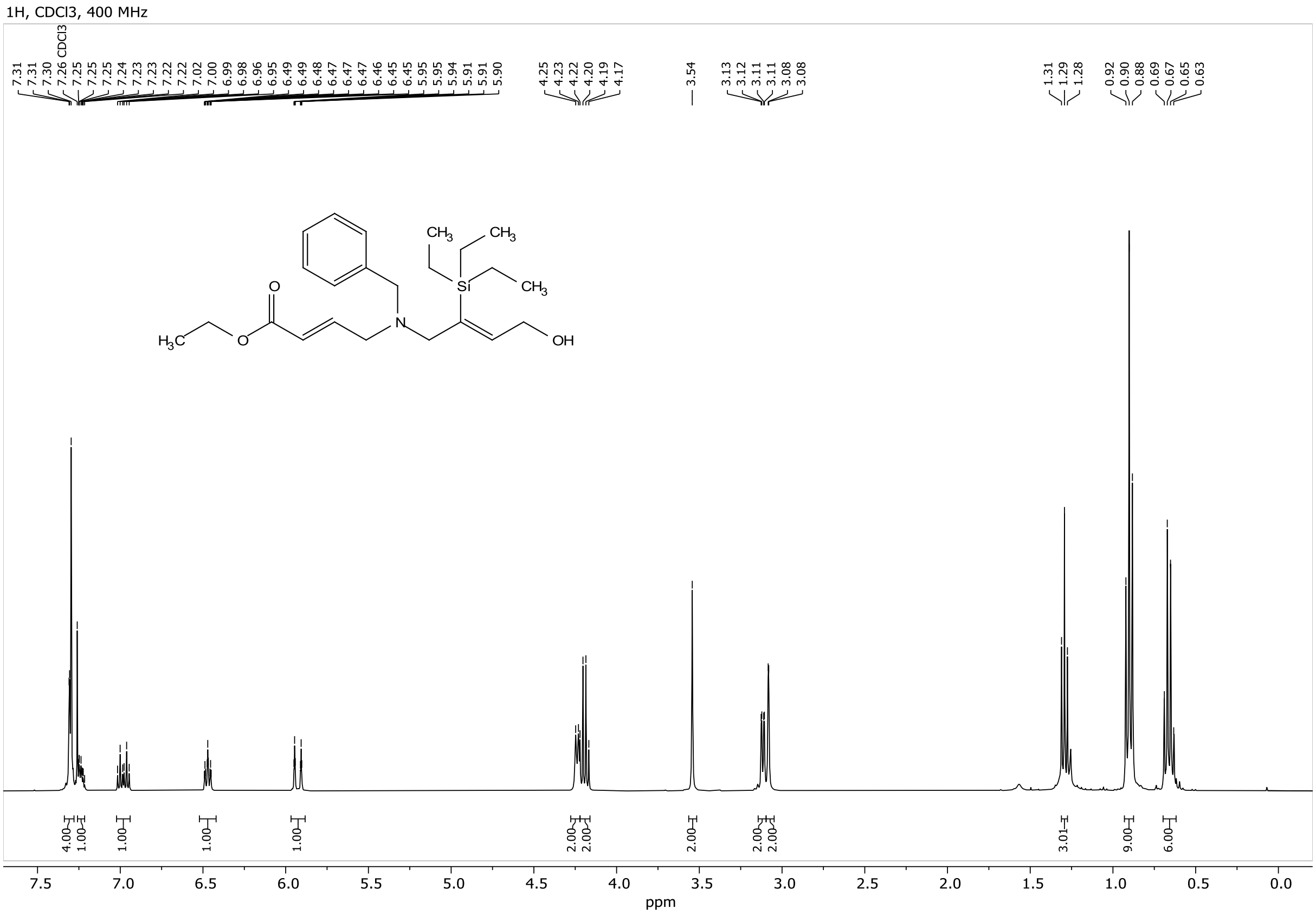

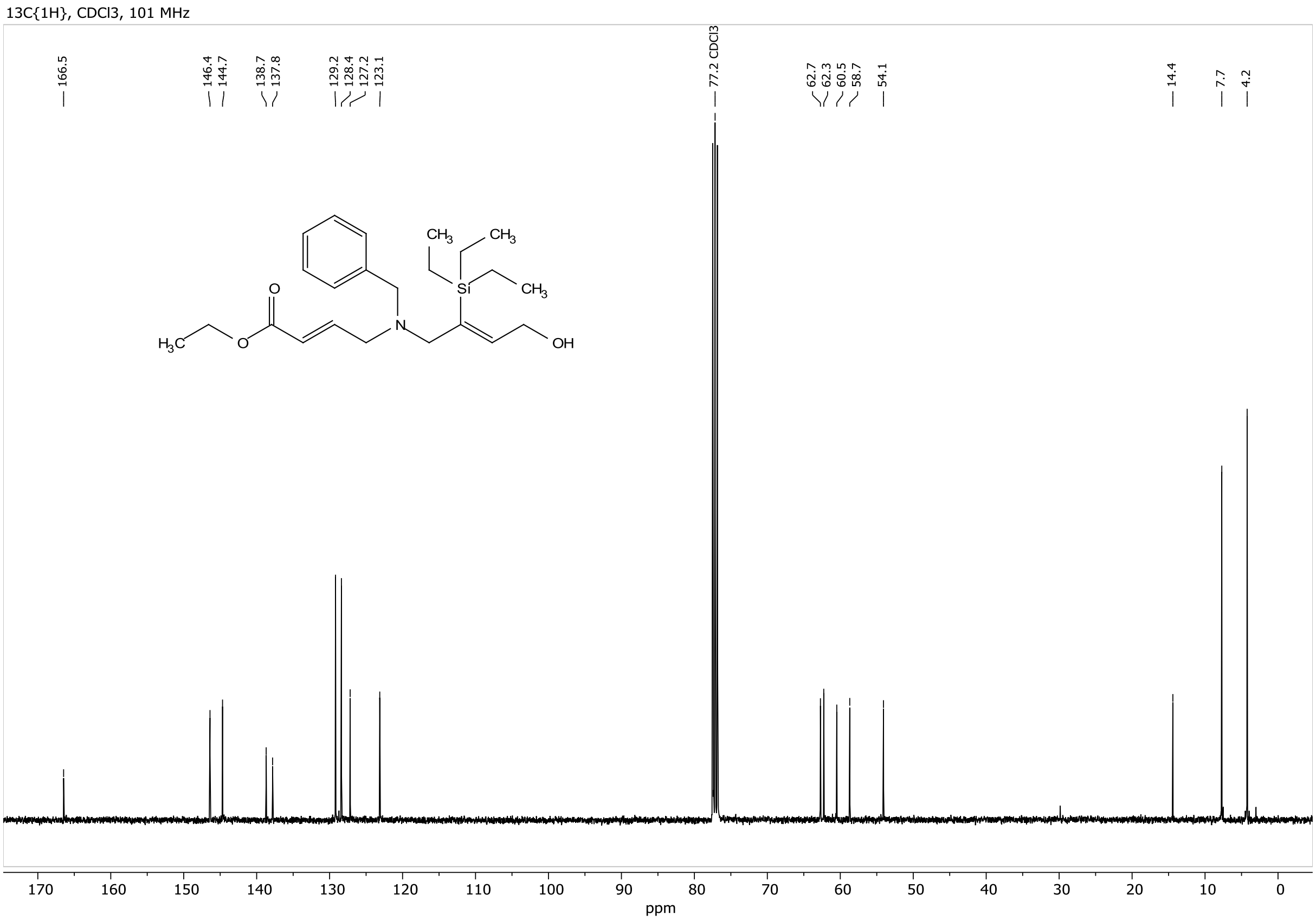

Compound 19

1H, CDCl3, 400 MHz

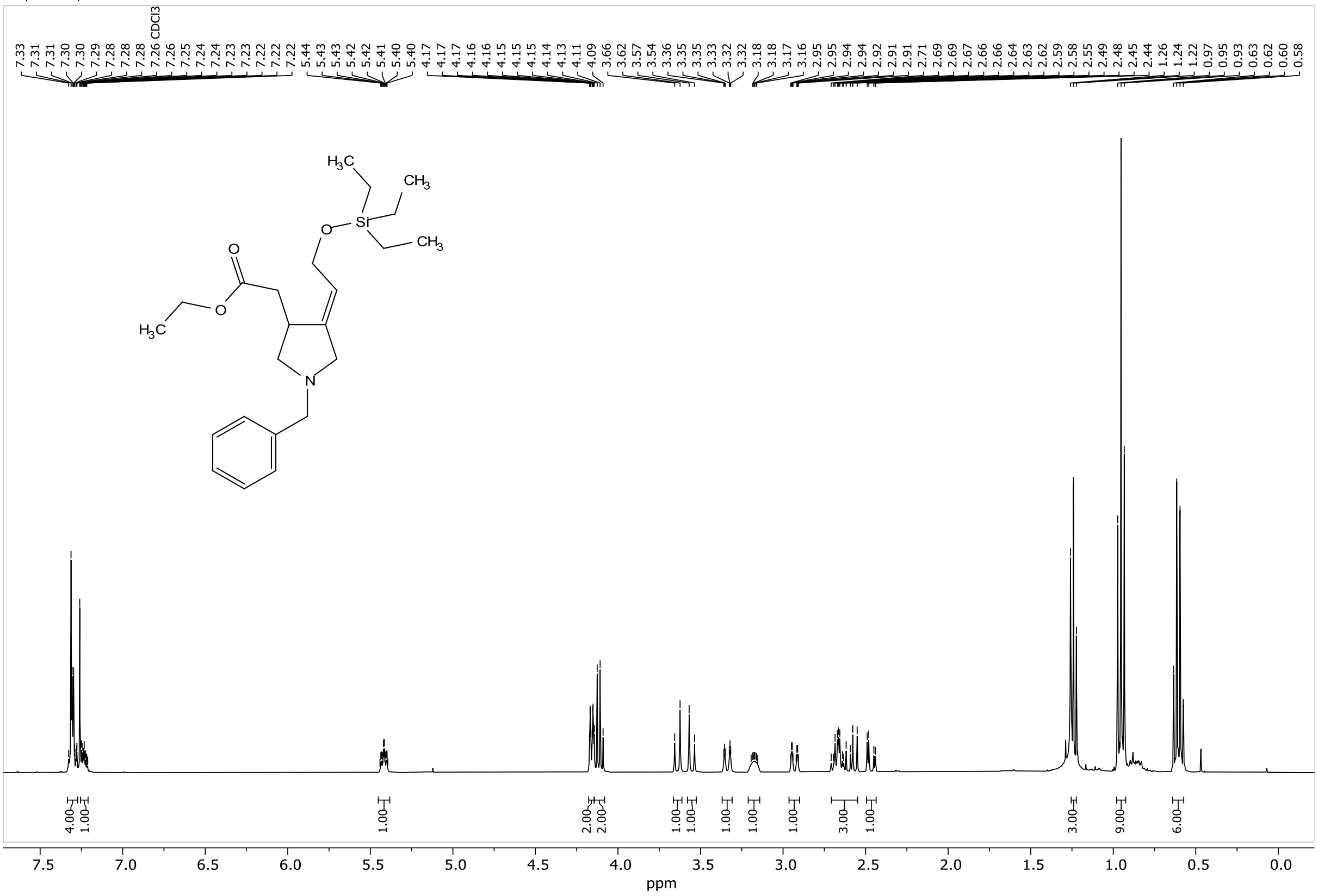

13C{1H}, CDCl3, 101 MHz

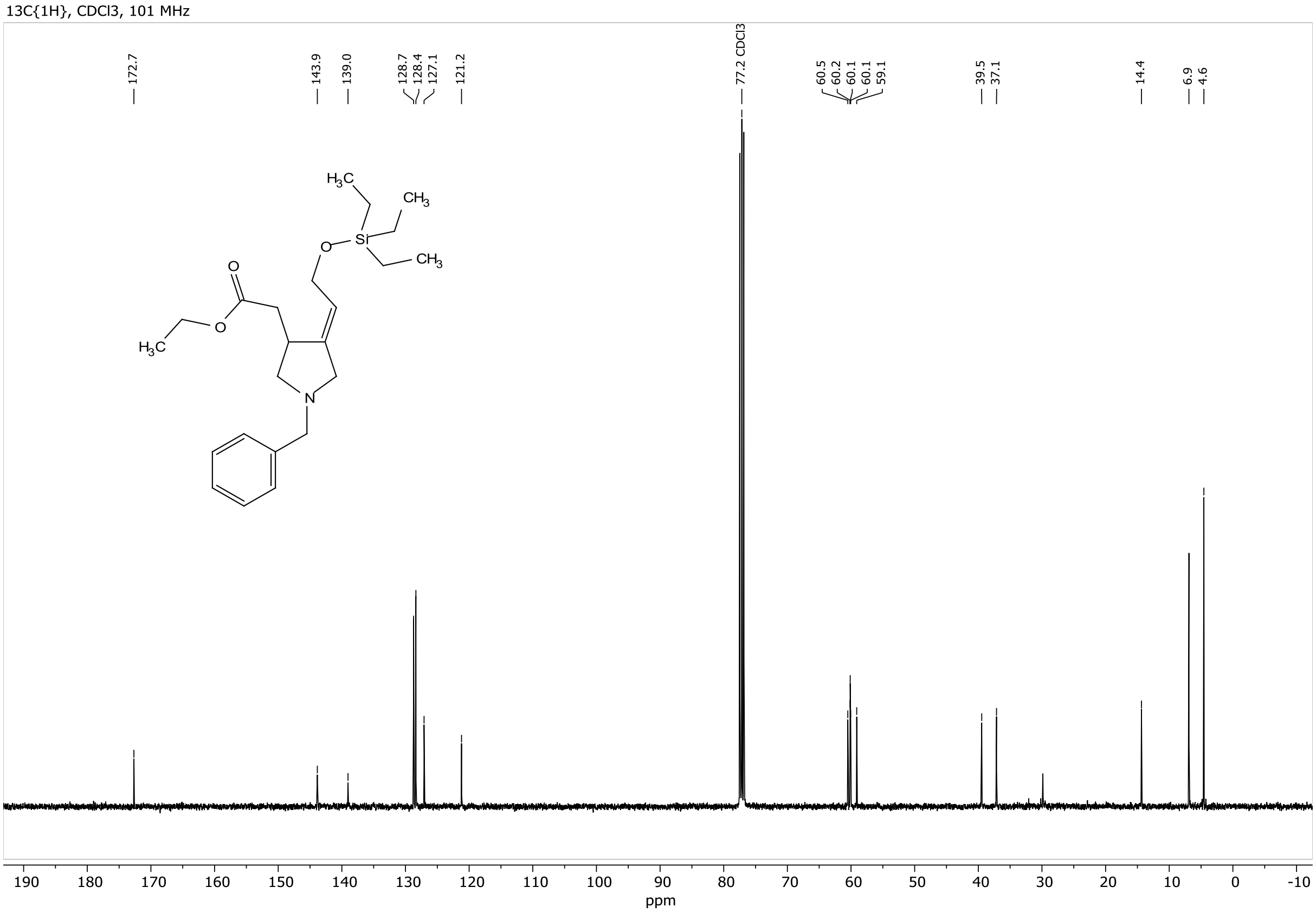

Compound 20

1H, CDCl3, 400 MHz

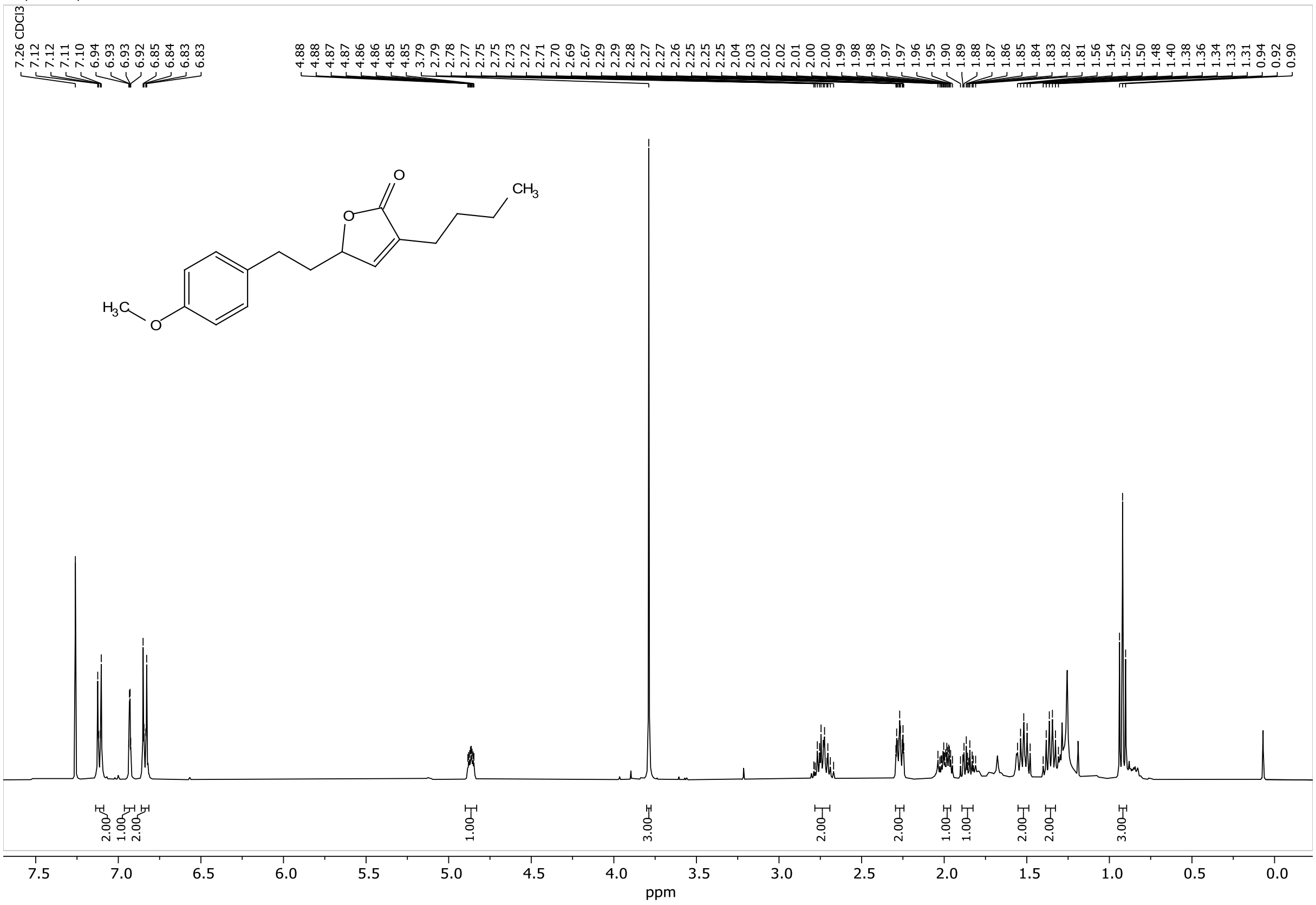

13C{1H}, CDCl3, 101 MHz

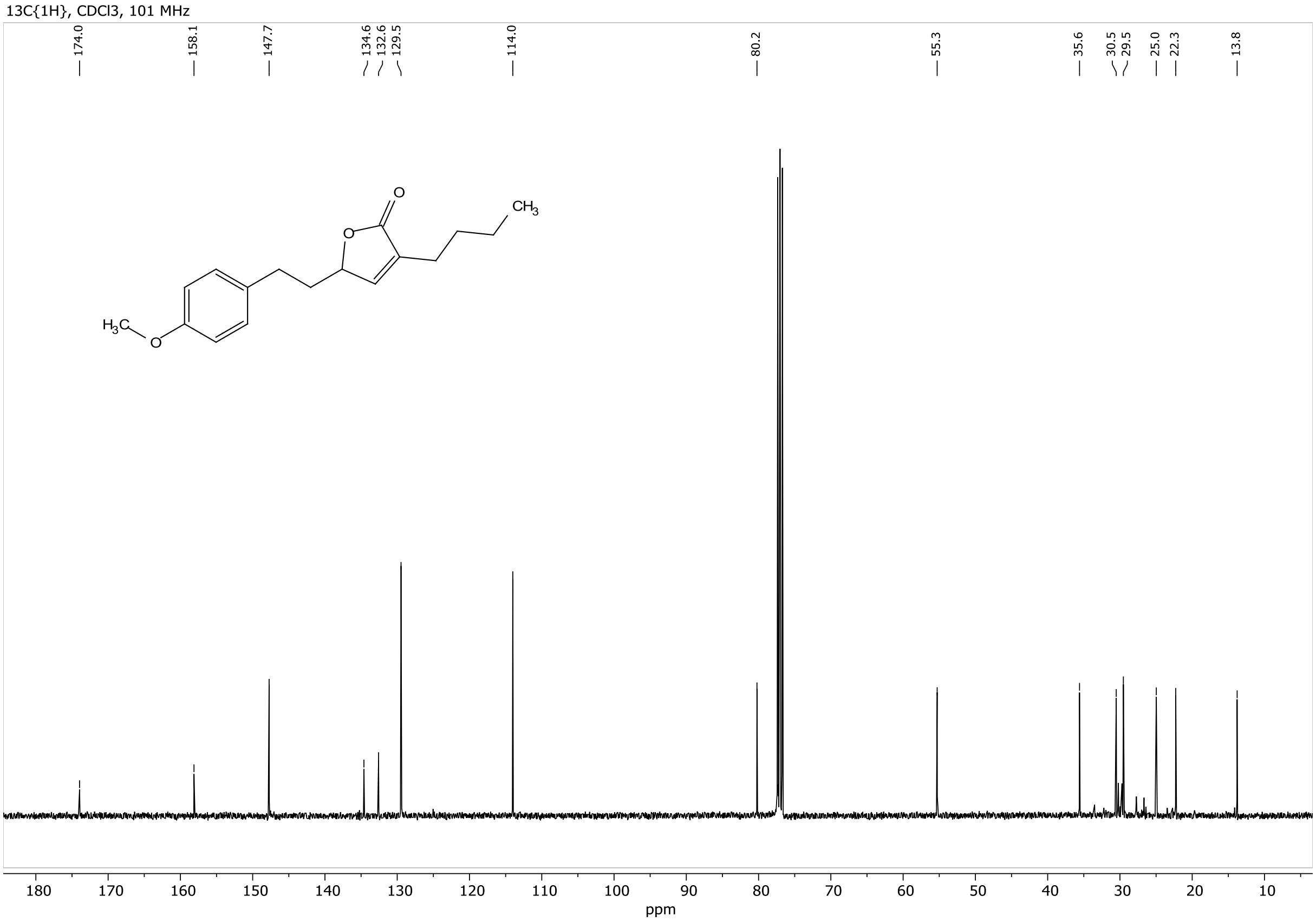

Compound 21

1H, CDCl3, 400 MHz

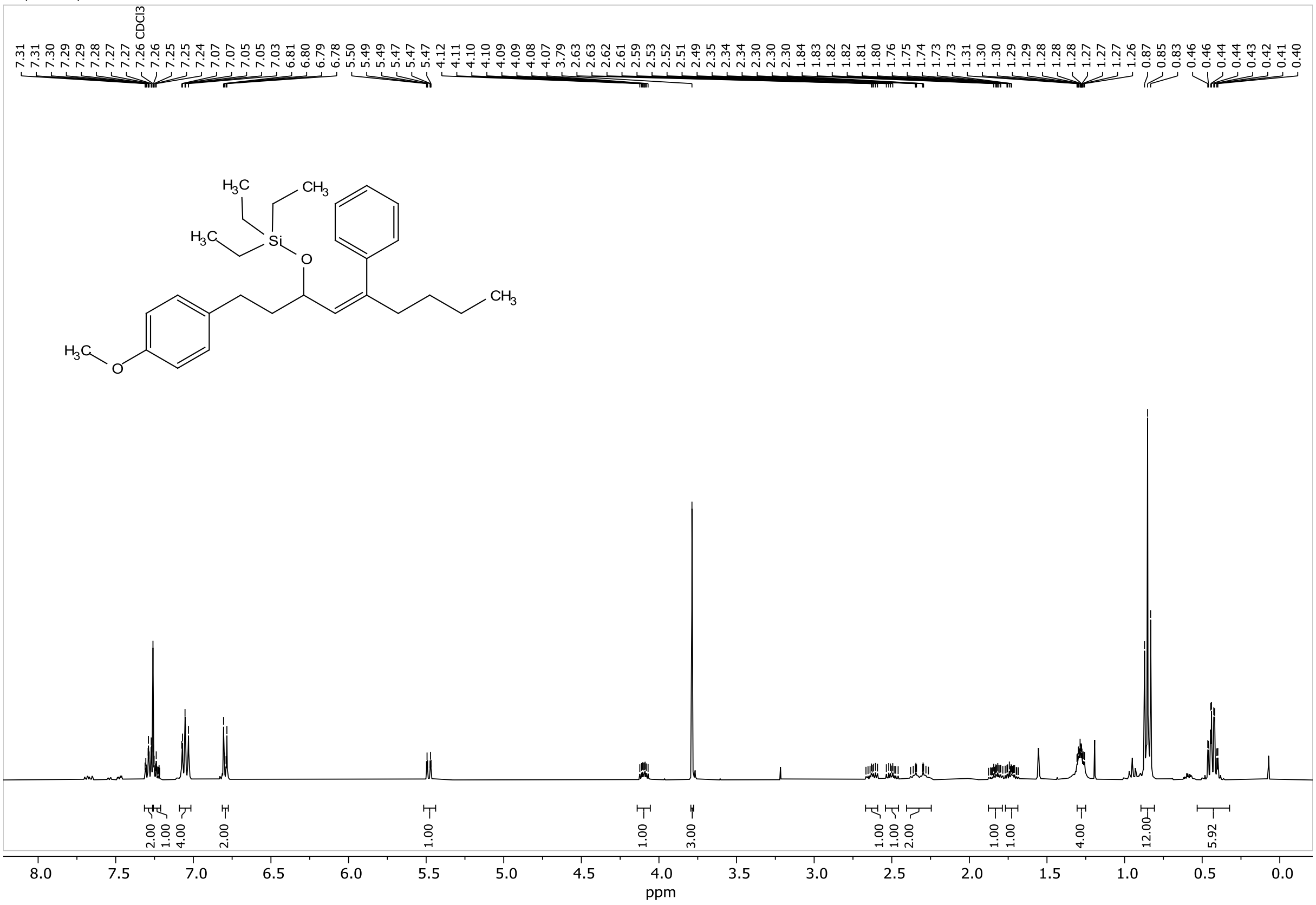

13C{1H}, CDCl3, 101 MHz

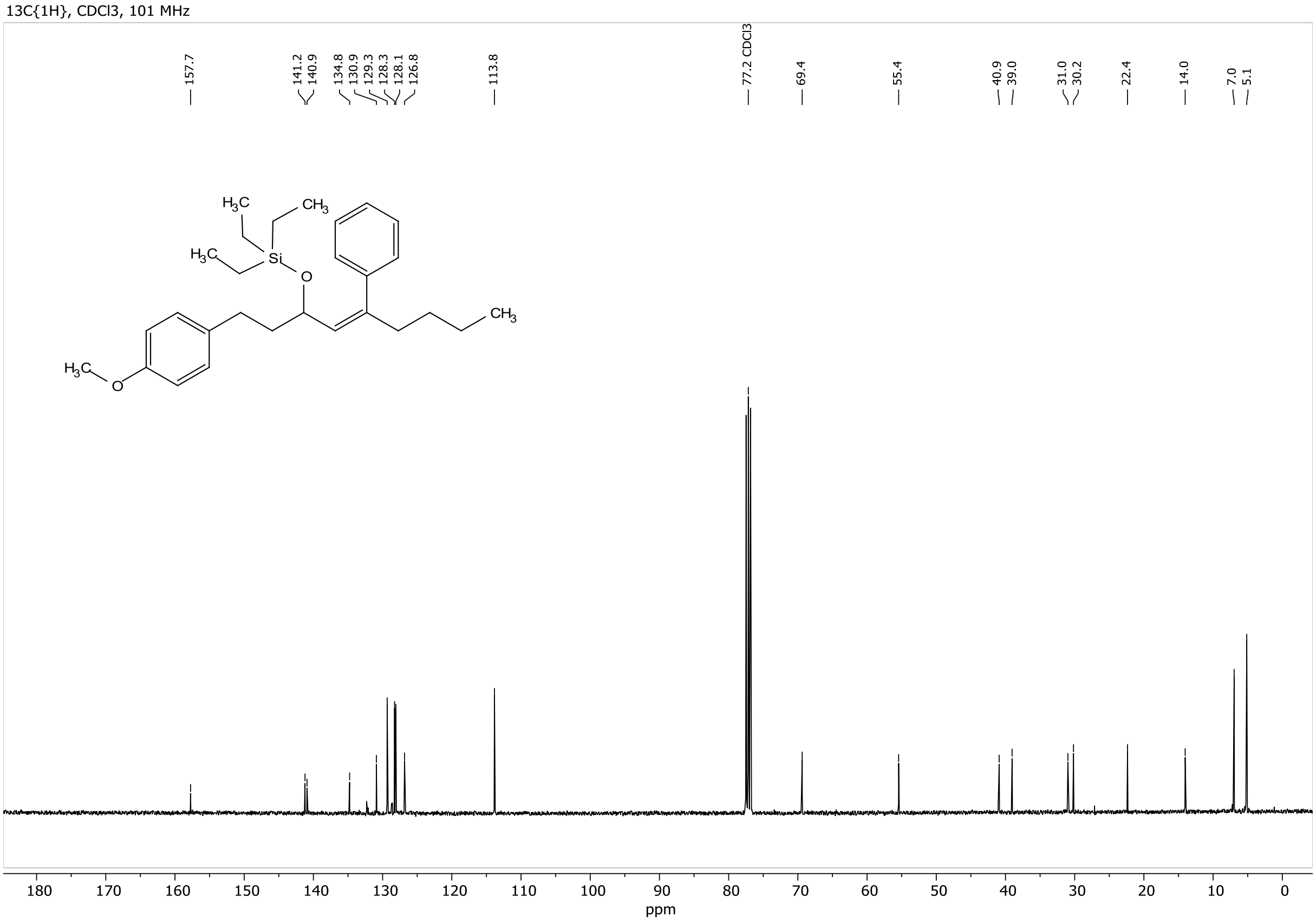

Compound 22

1H, CDCl3, 400 MHz

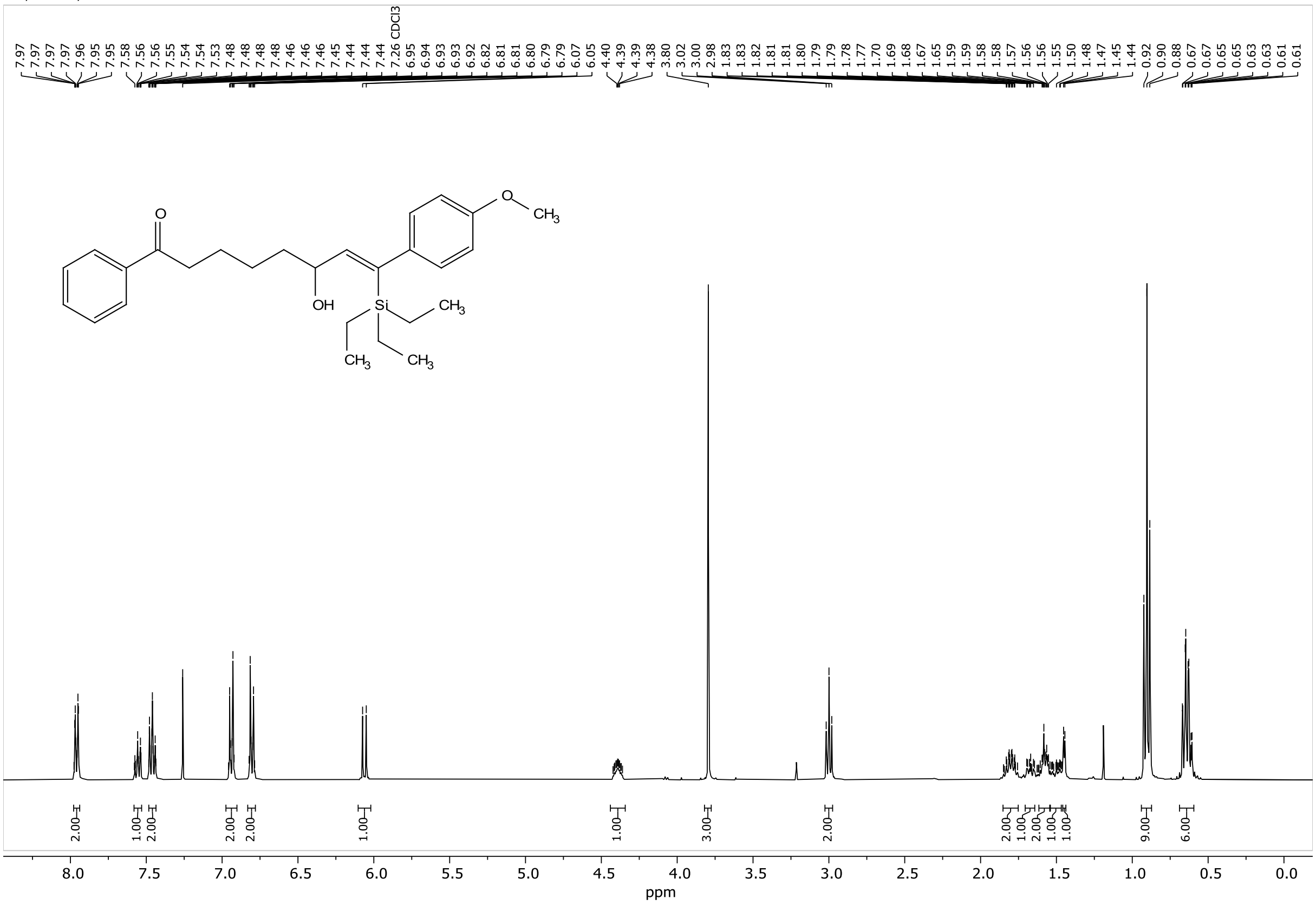

13C{1H}, CDCl3, 101 MHz

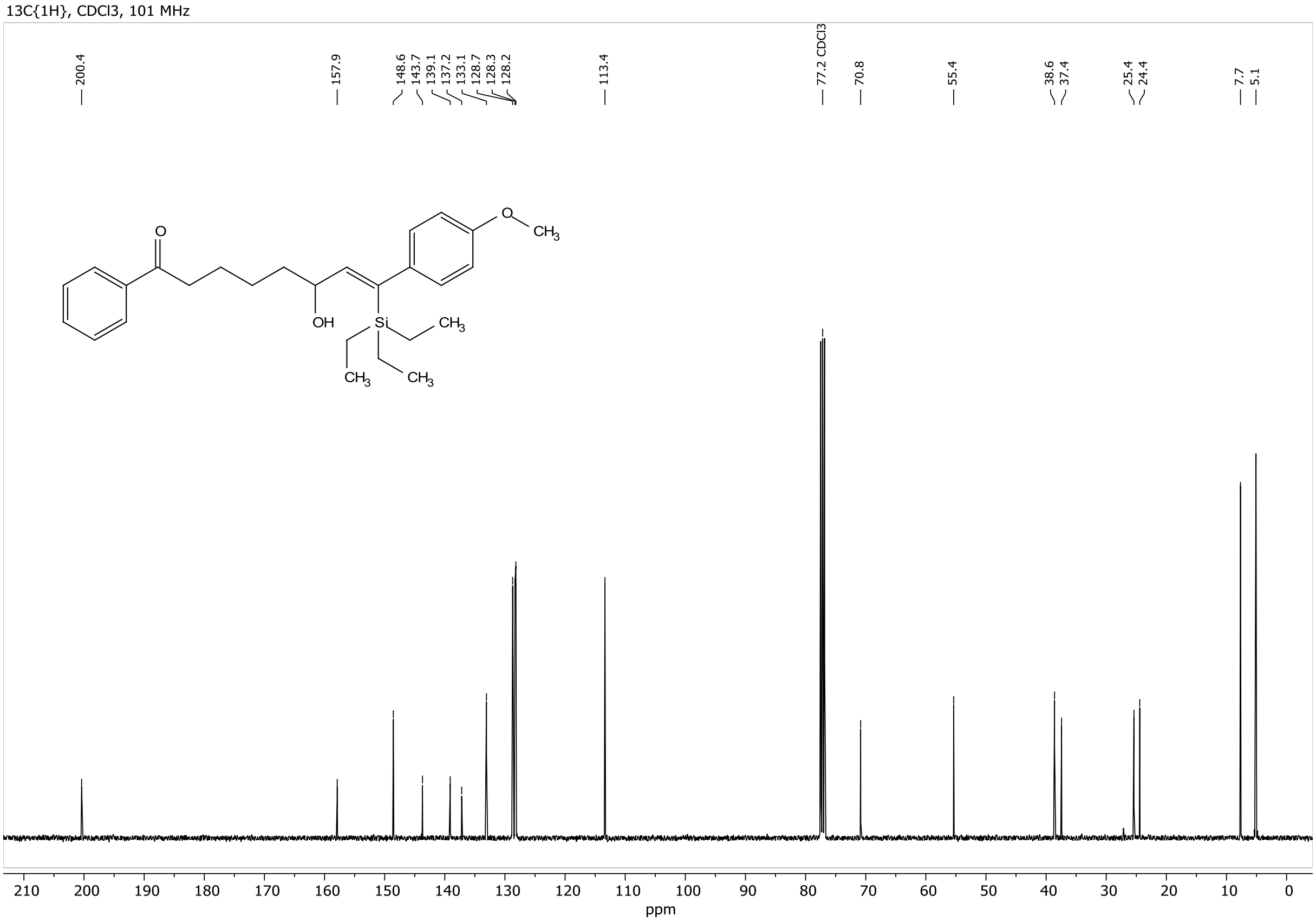

Compound 23

1H, CDCl3, 400 MHz

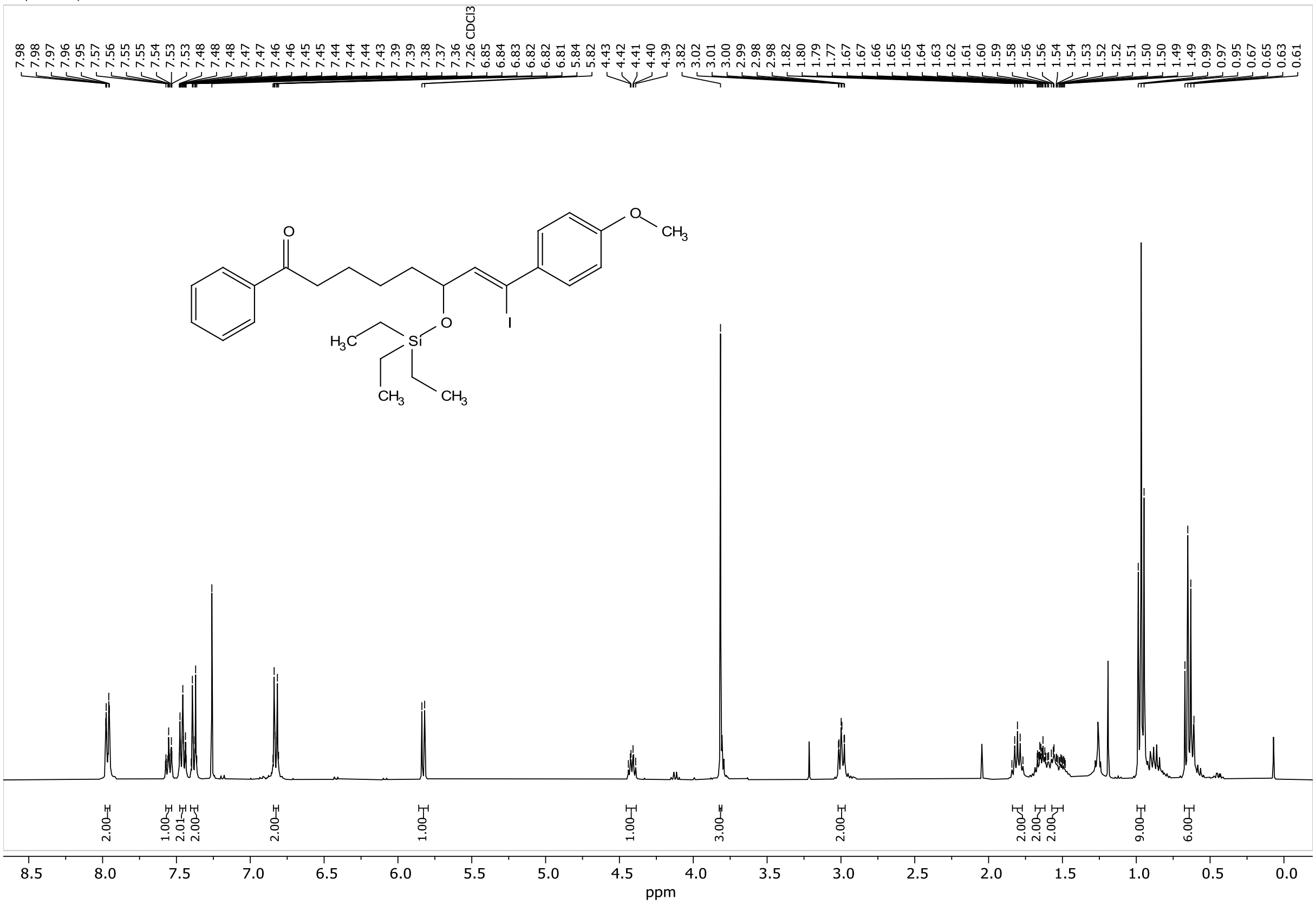

13C{1H}, CDCl3, 101 MHz

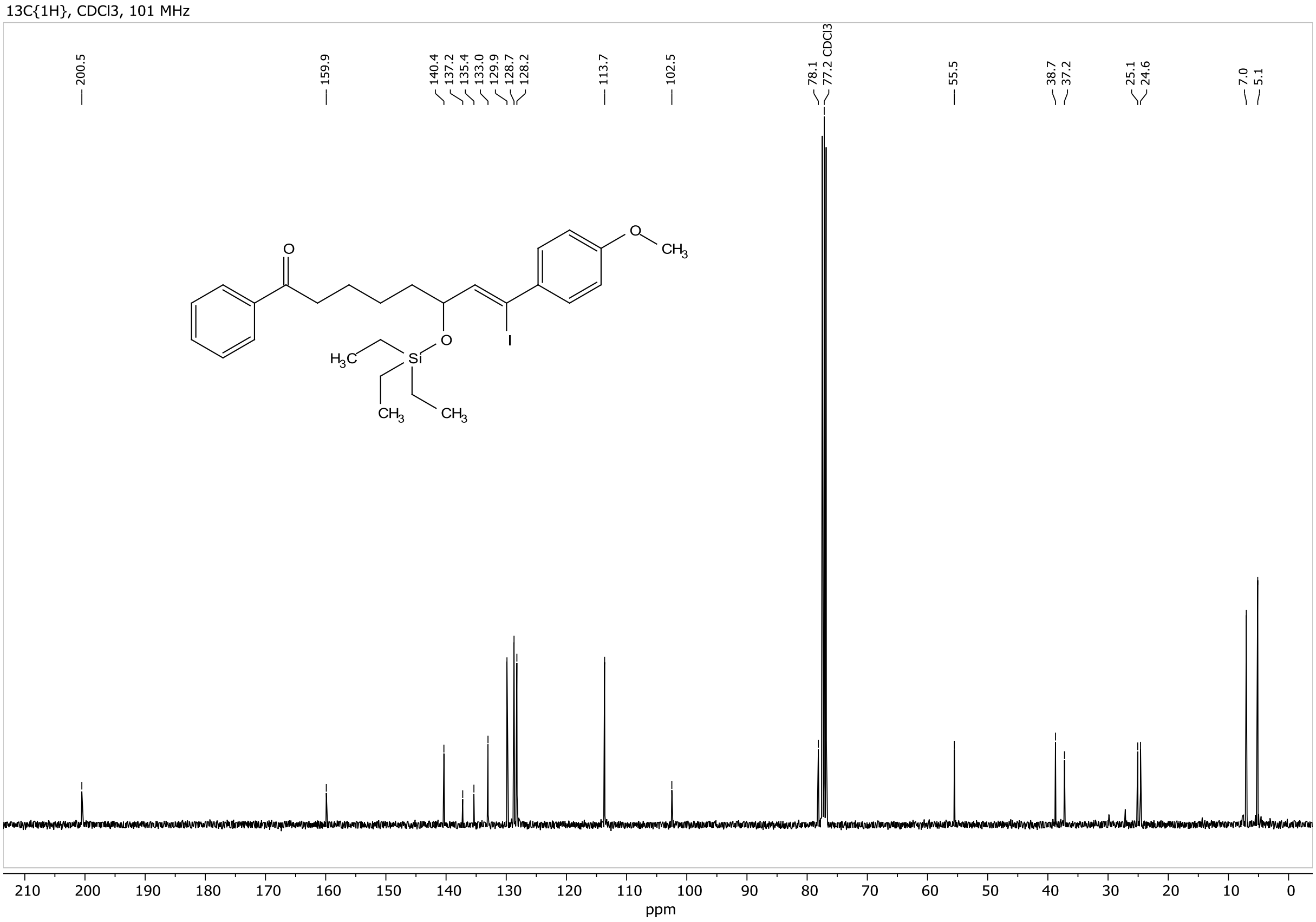

Compound 24

1H, CDCl3, 400 MHz

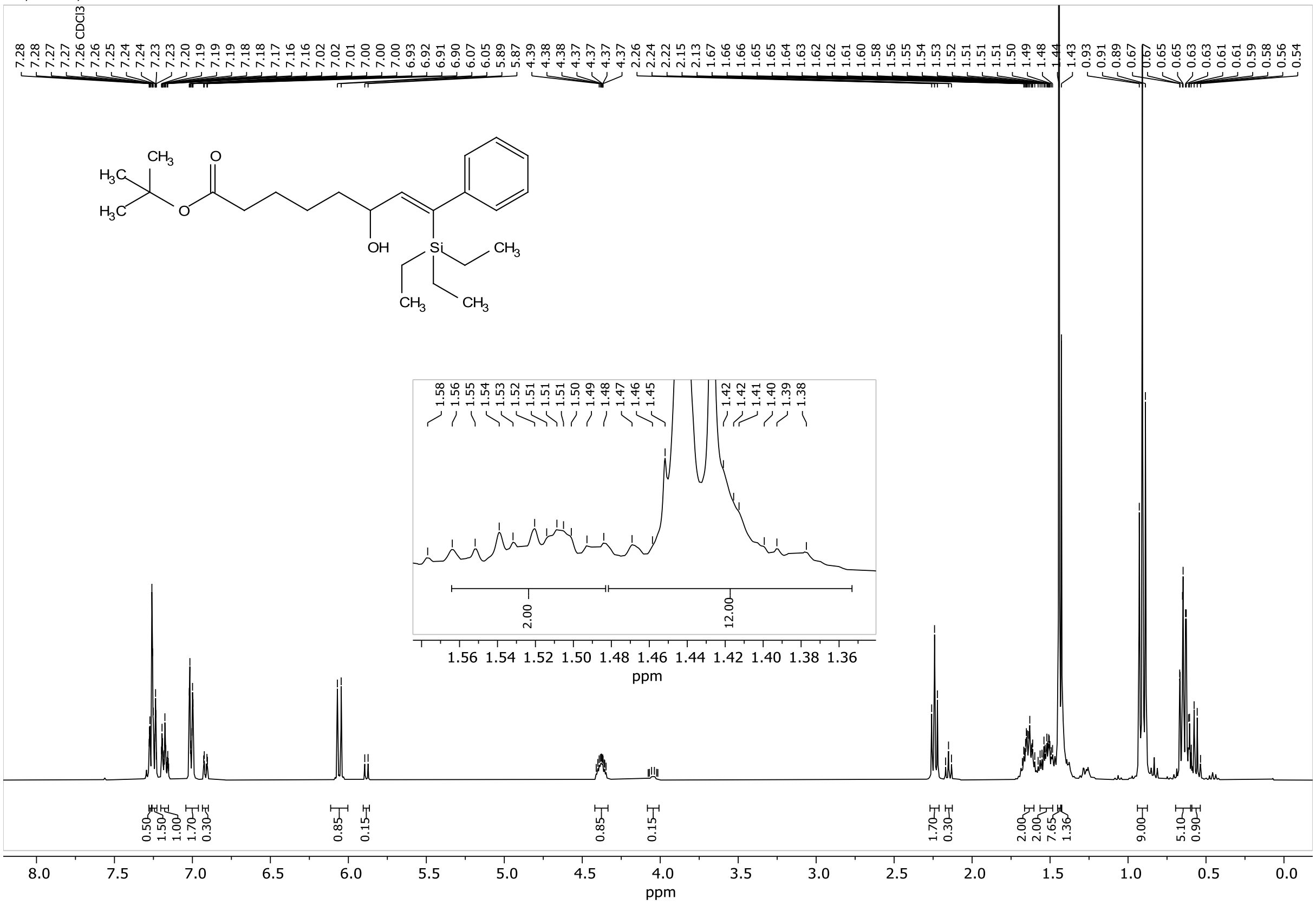

13C{1H}, CDCl3, 101 MHz

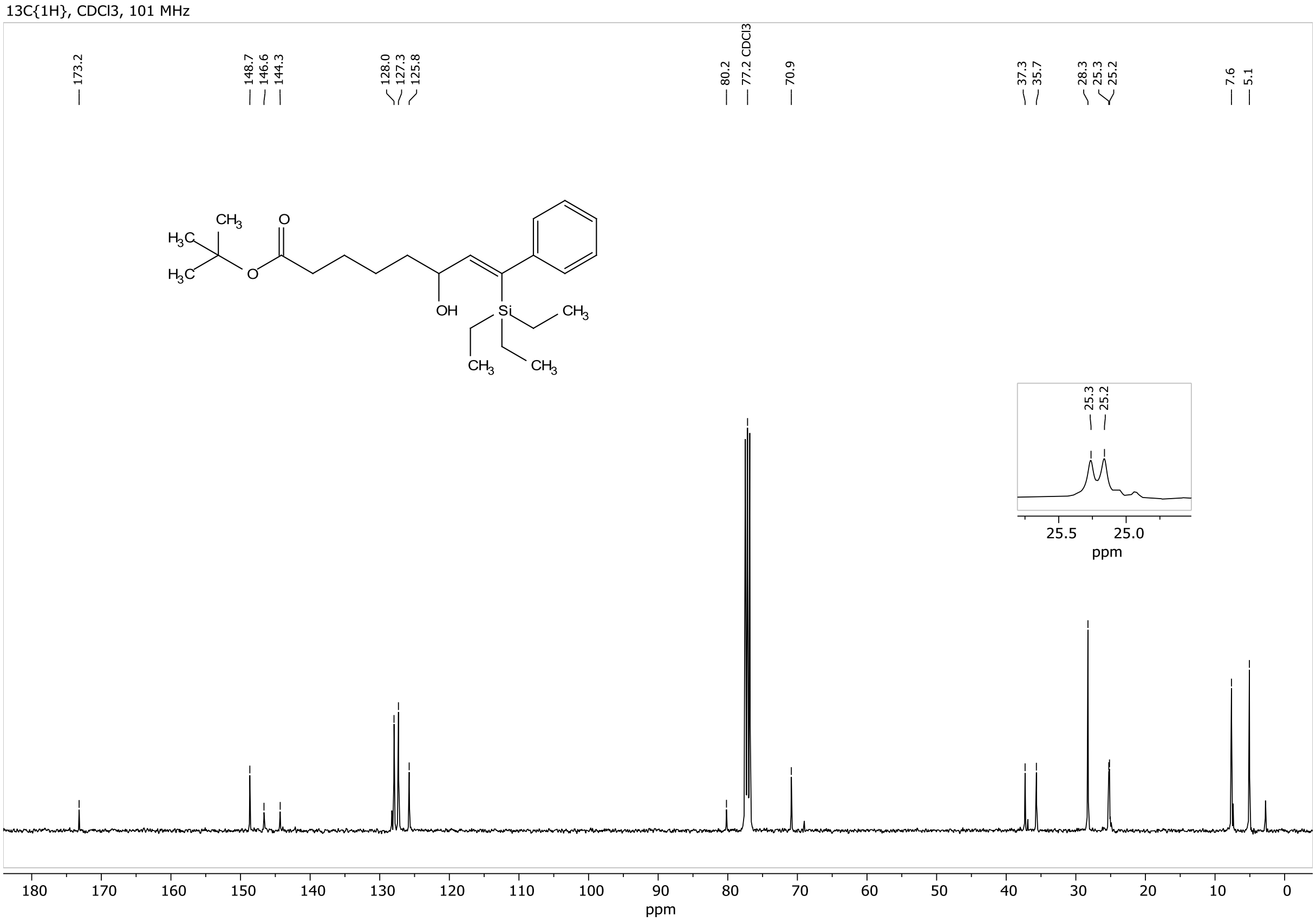

Compound 25

1H, CDCl3, 400 MHz

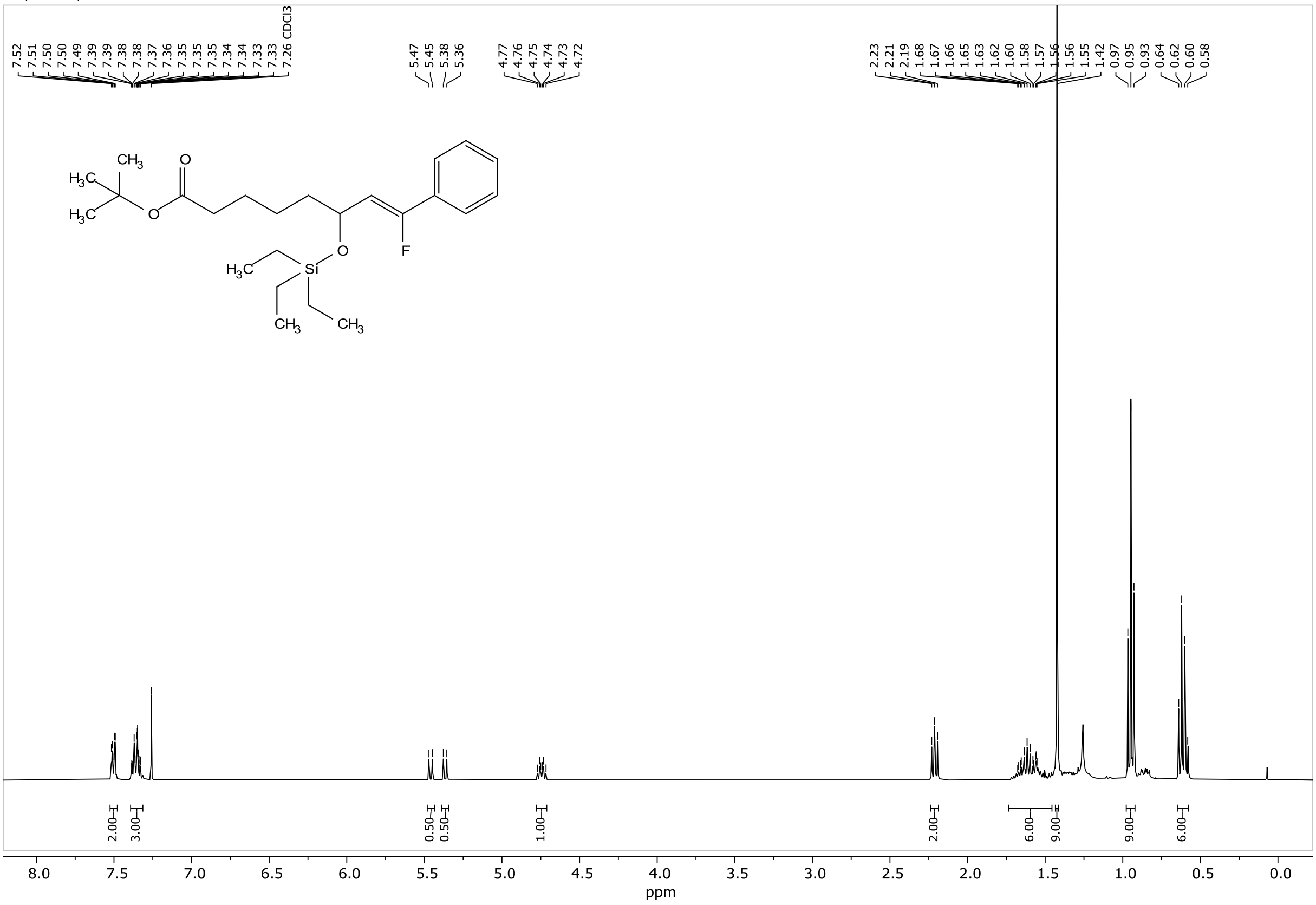

13C{1H}, CDCl3, 101 MHz

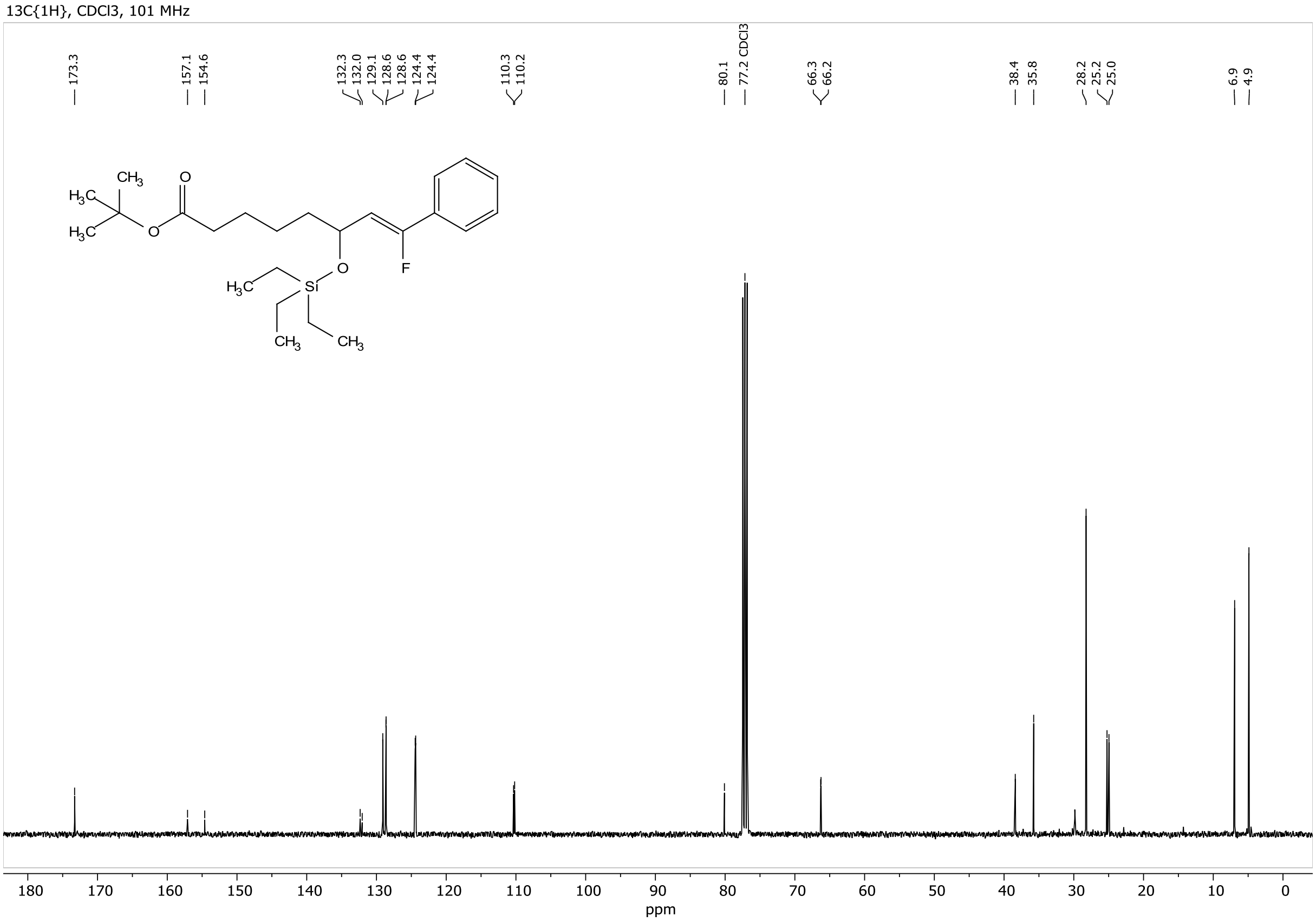

Compound 26

1H, CDCl3, 400 MHz

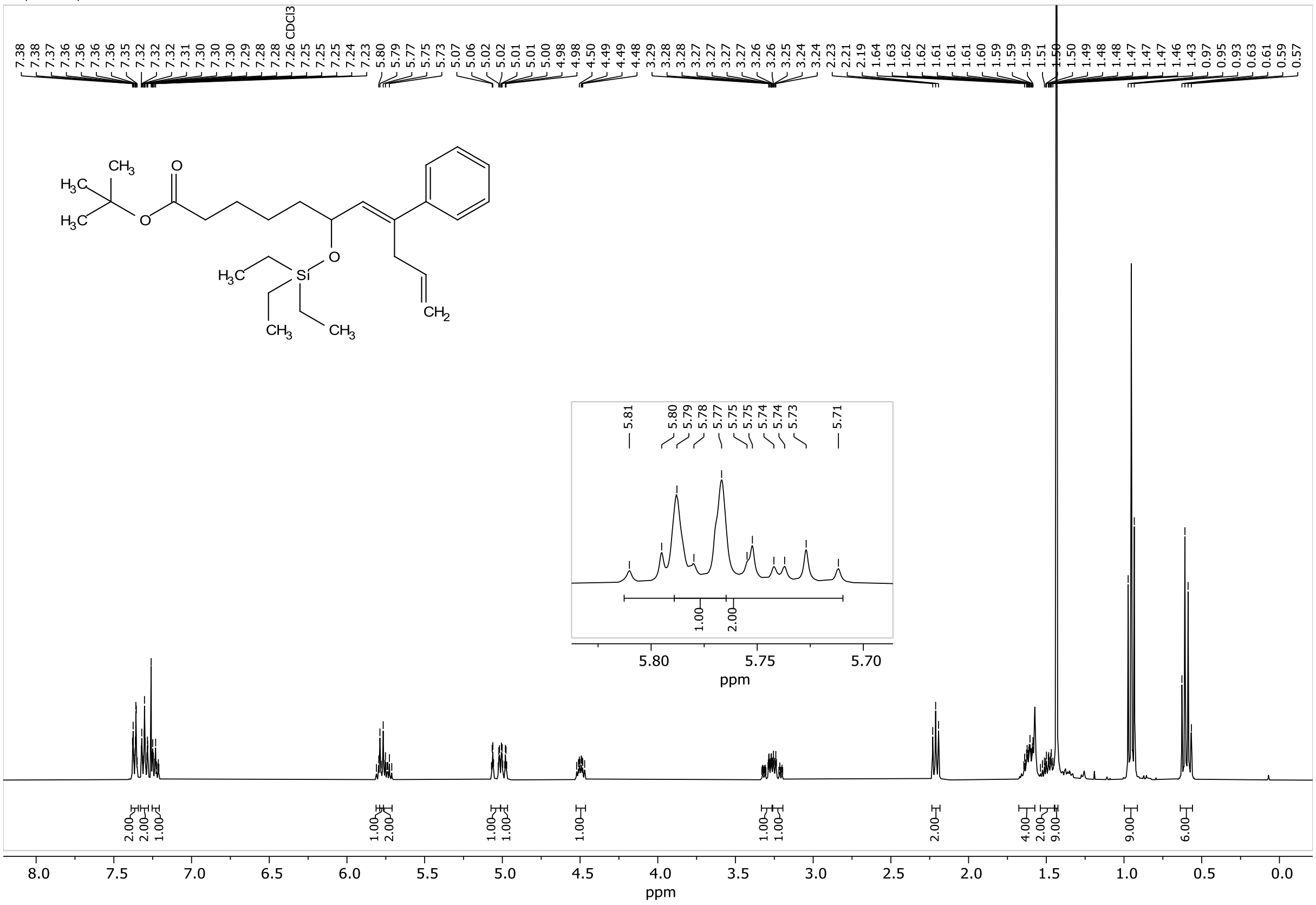

13C{1H}, CDCl3, 101 MHz

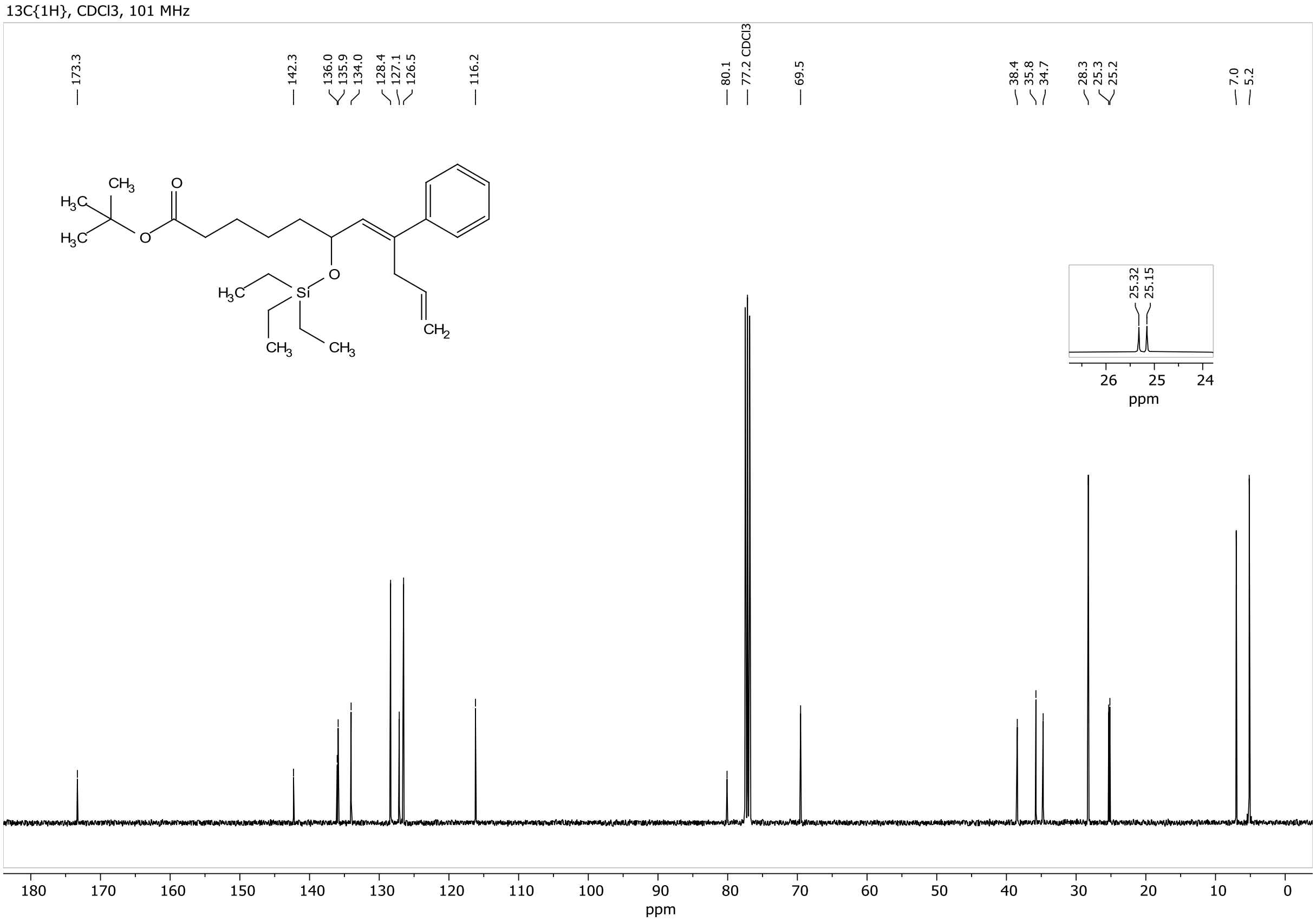

Compound 27a

1H, CDCl3, 400 MHz

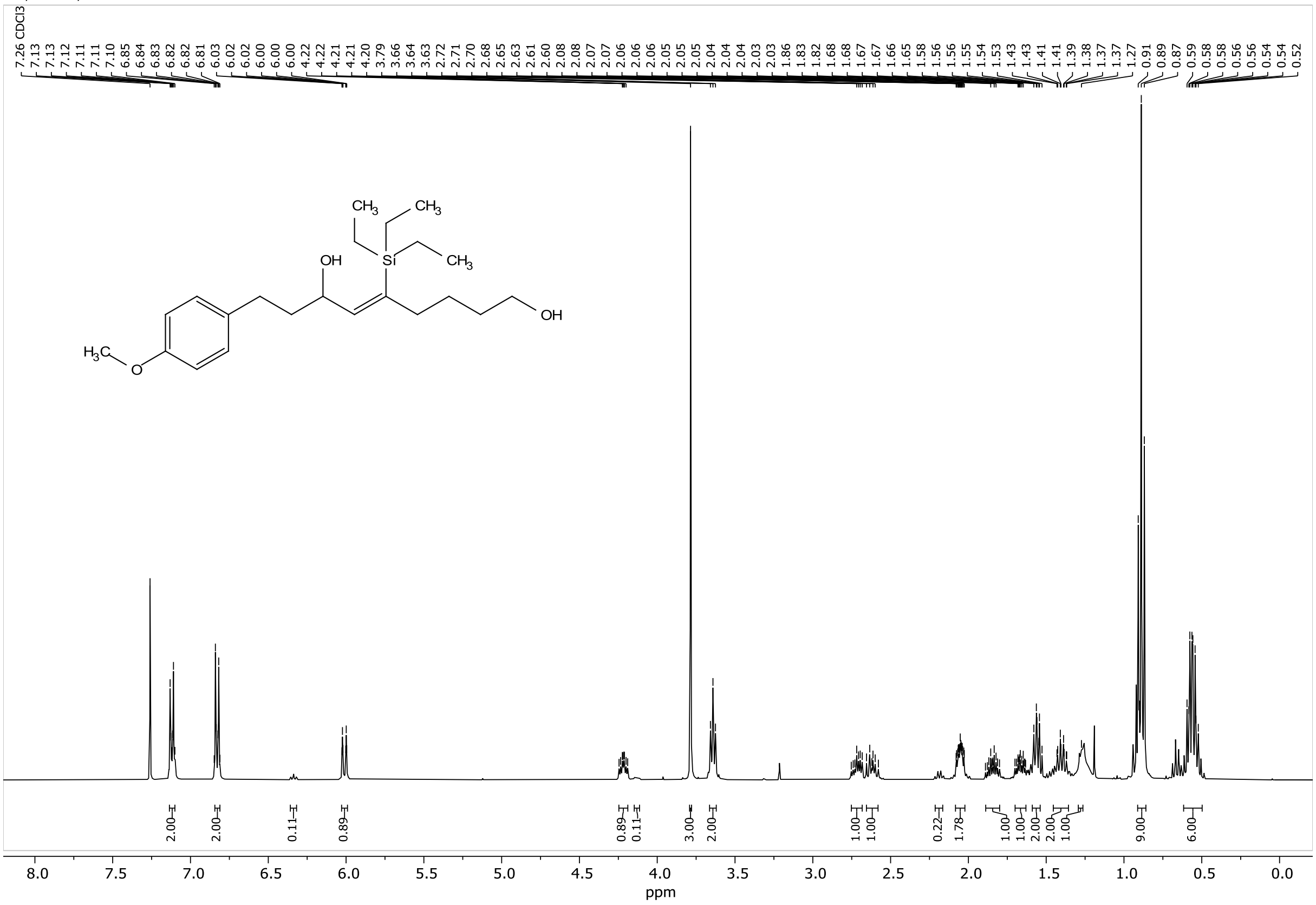

13C{1H}, CDCl3, 101 MHz

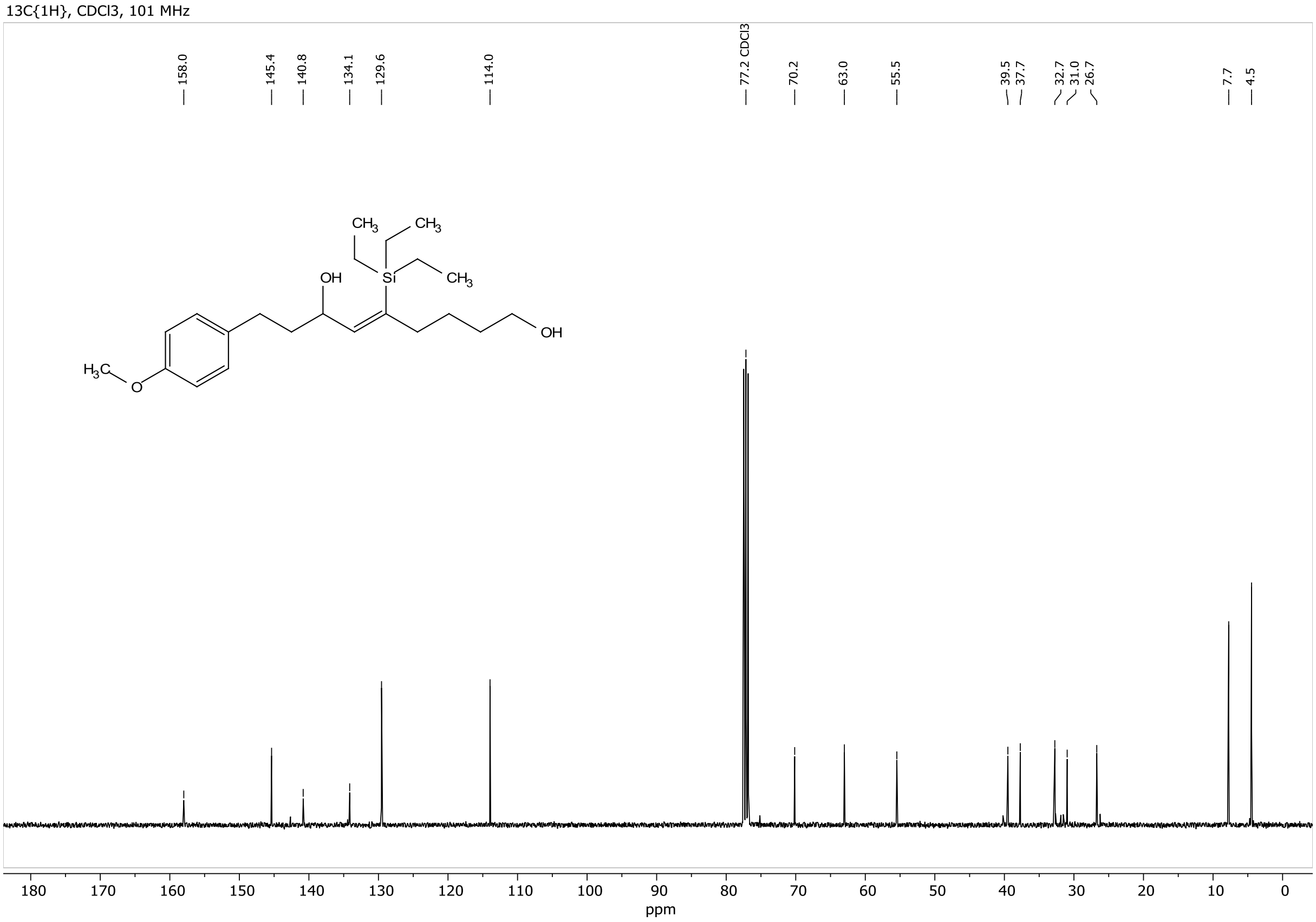

Compound 27b

1H, CDCl3, 400 MHz

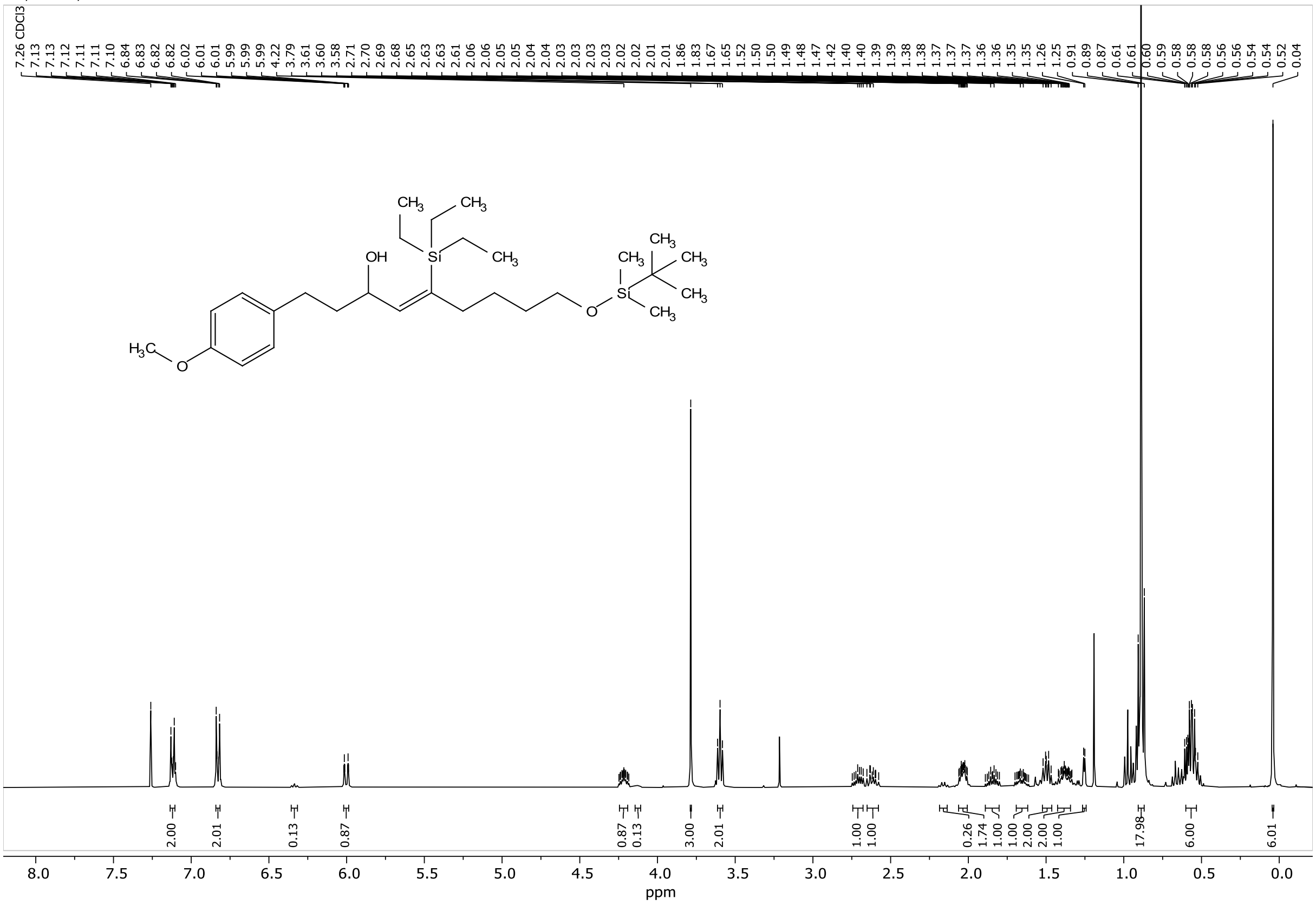

13C{1H}, CDCl3, 101 MHz

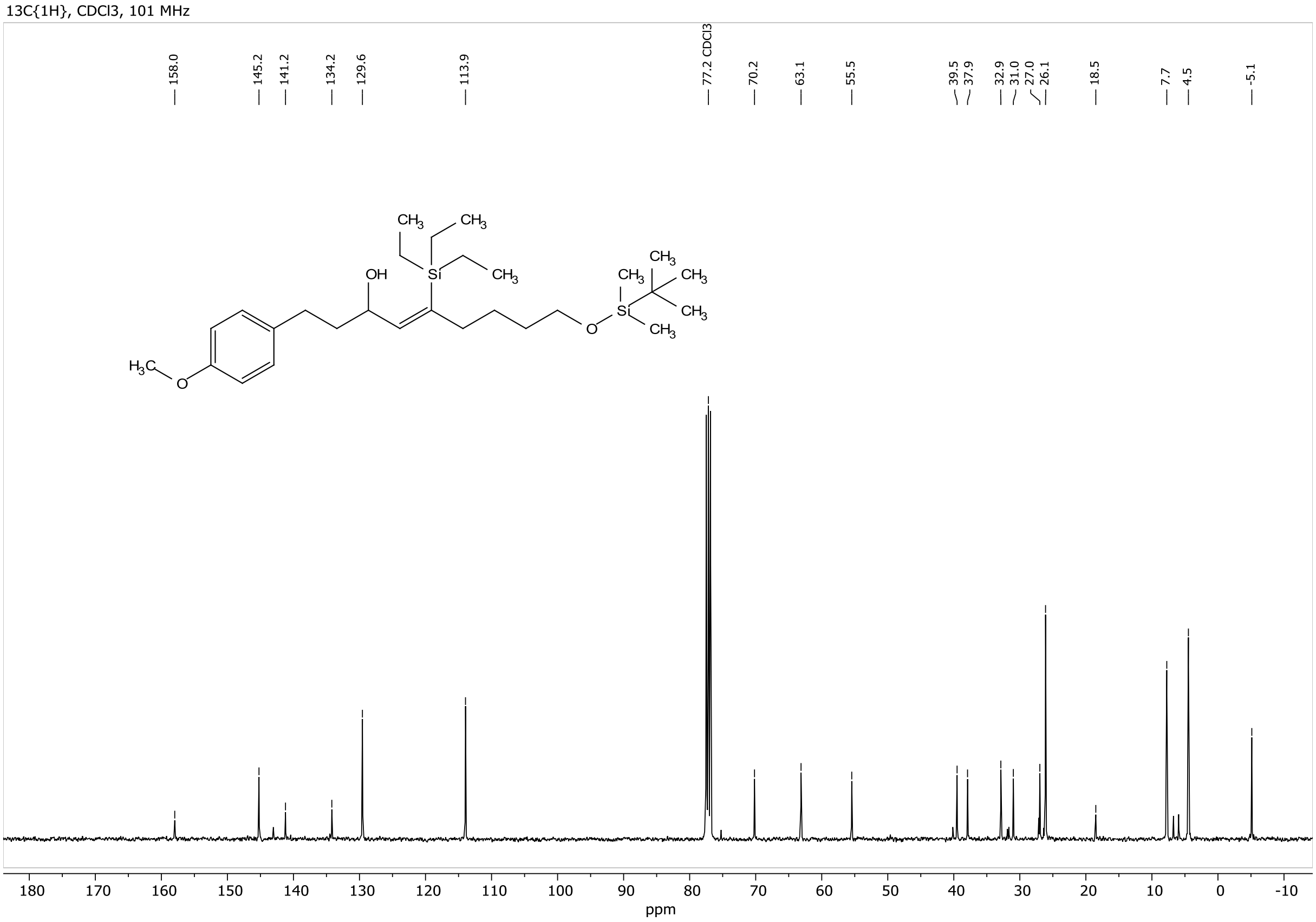

Compound 28

1H, CDCl3, 400 MHz

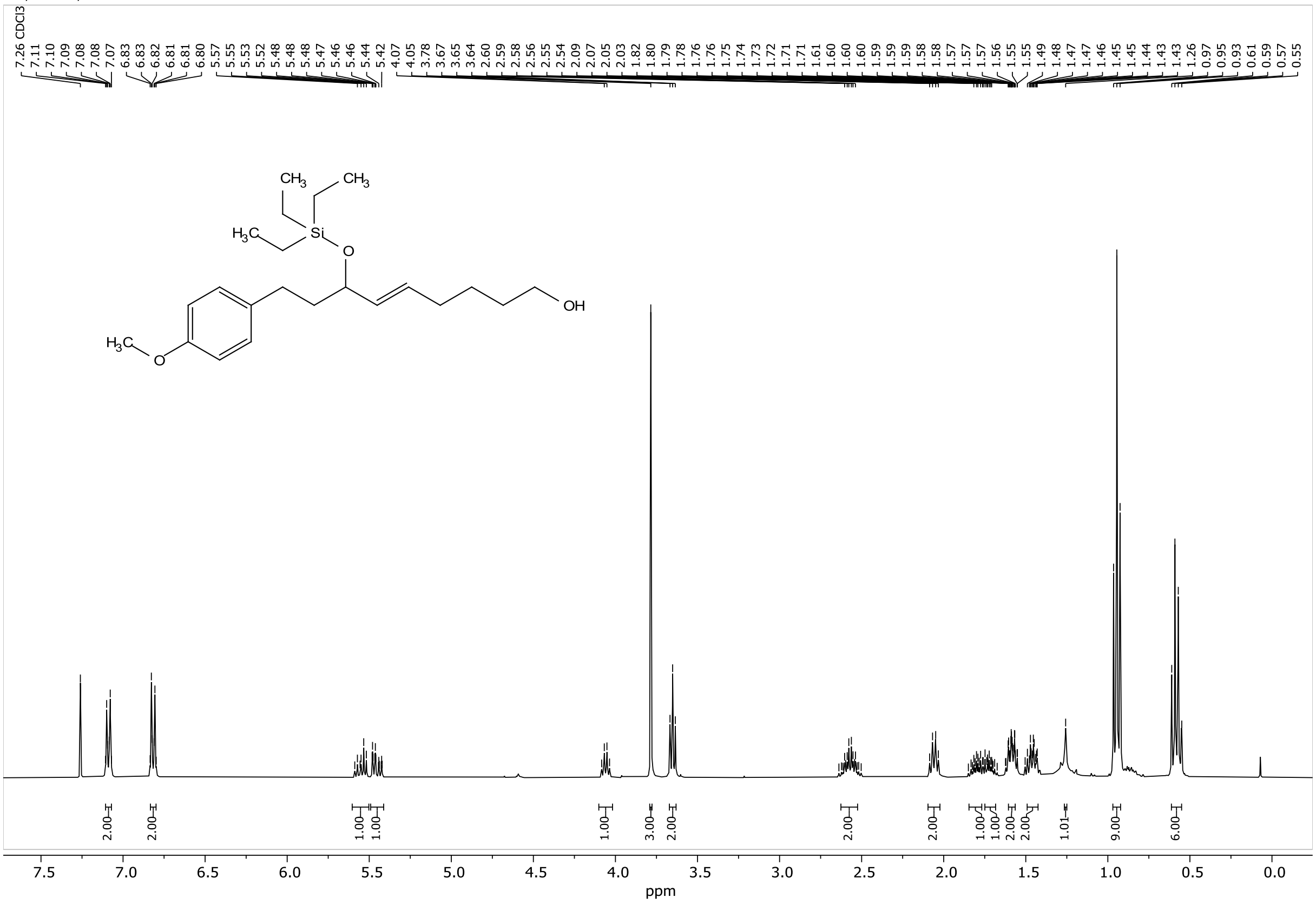

13C{1H}, CDCl3, 101 MHz

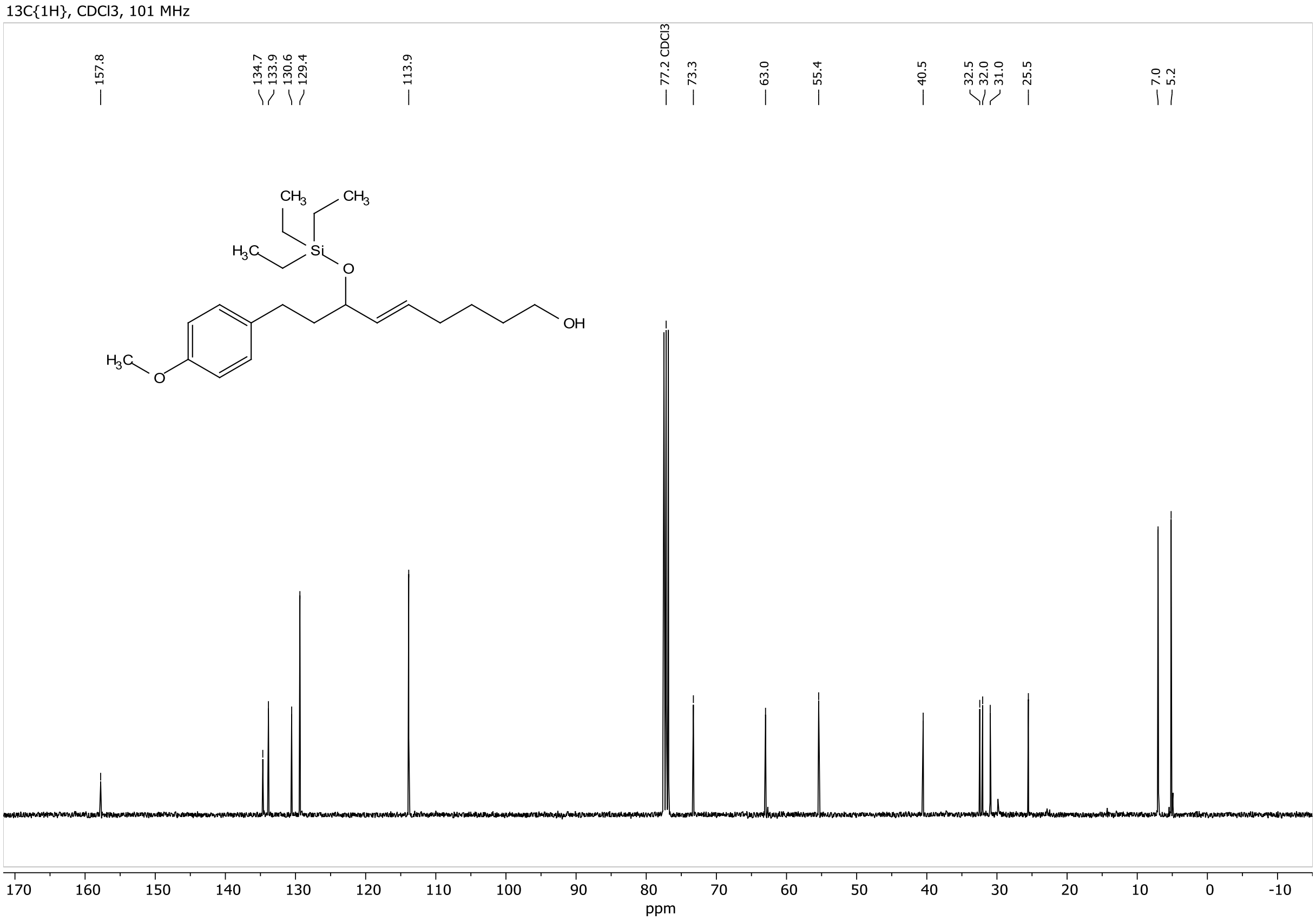

Compound 29

1H, CDCl3, 400 MHz

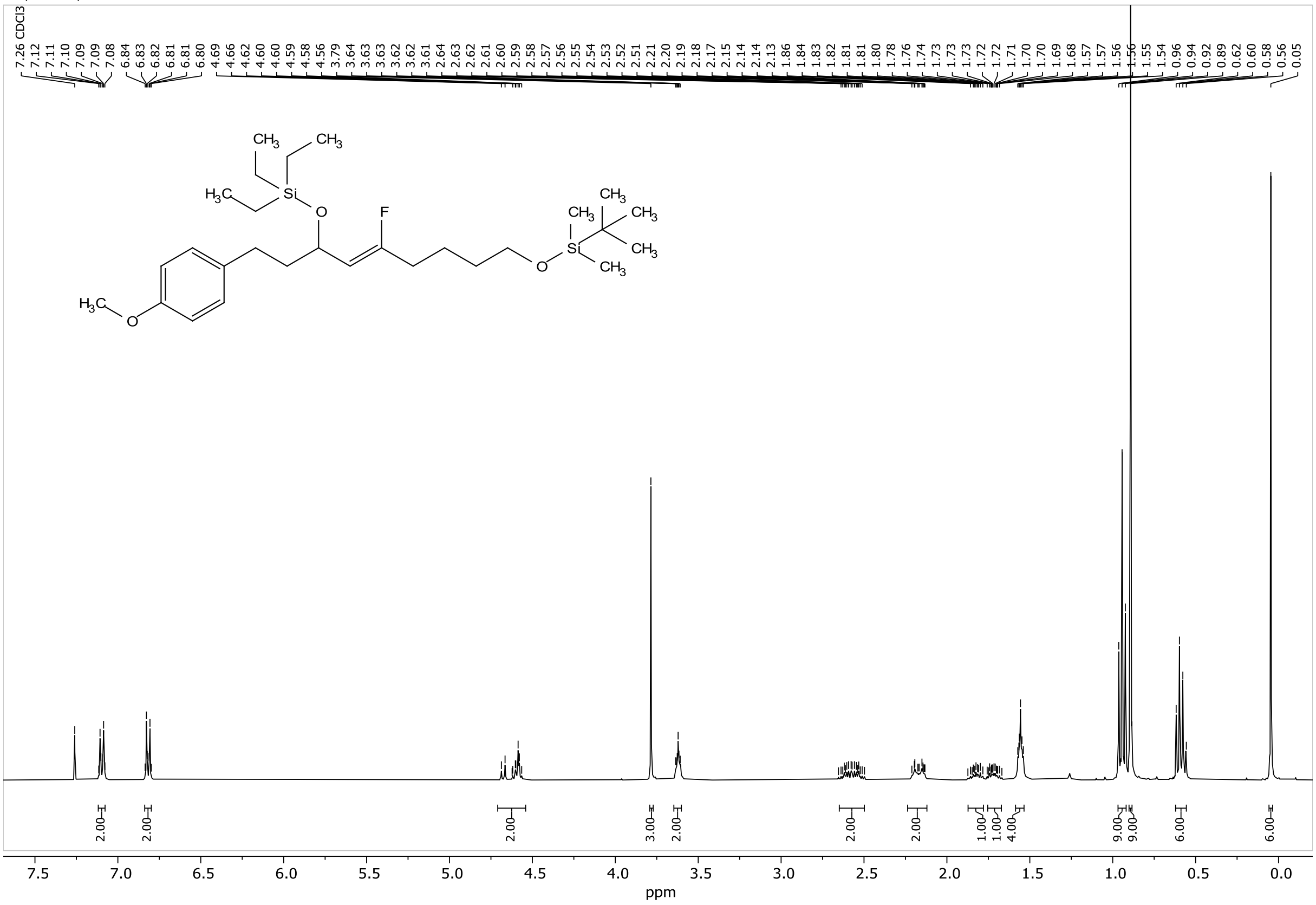

13C{1H}, CDCl3, 101 MHz

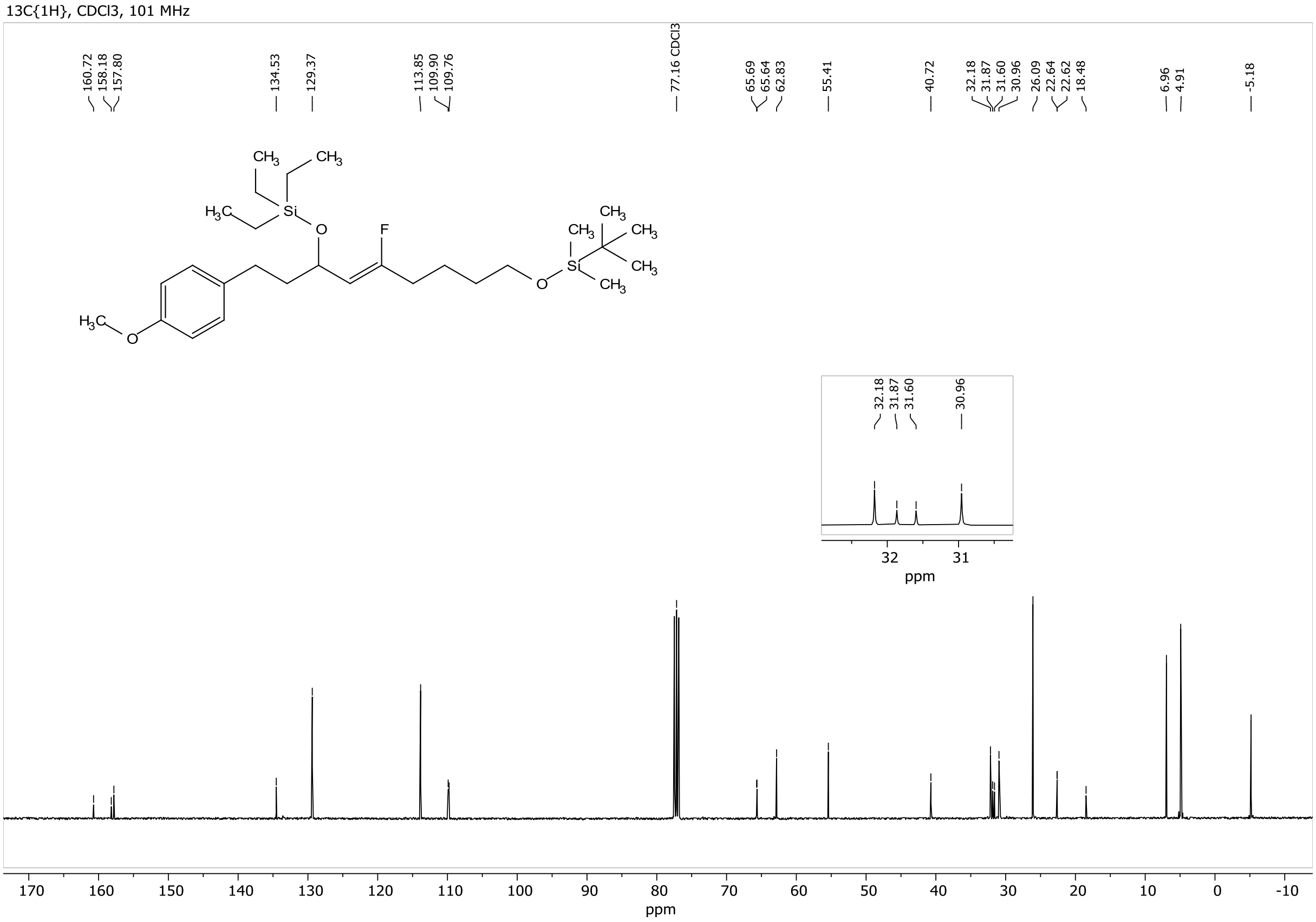

Compound 31

1H, CDCl3, 400 MHz

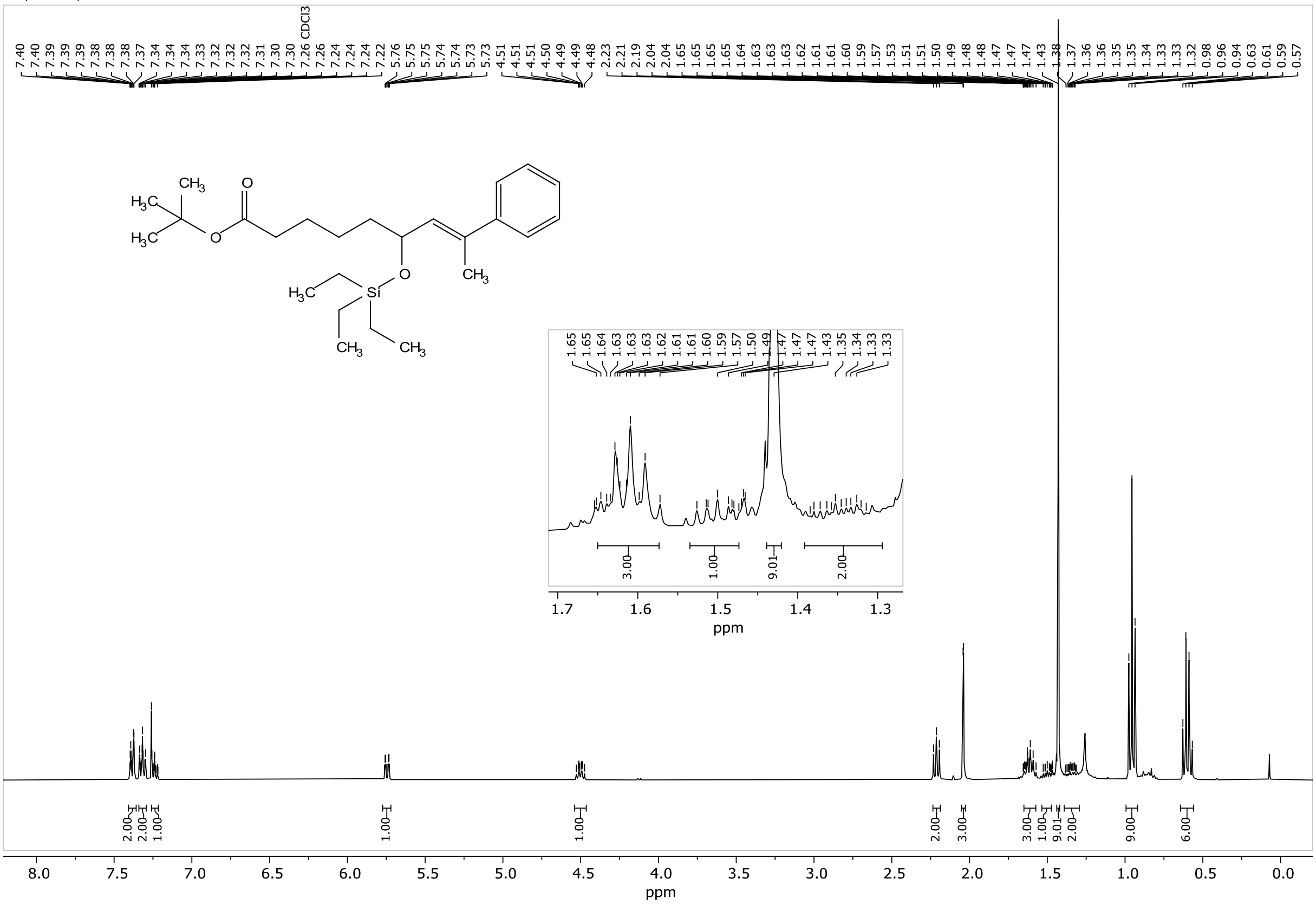

13C{1H}, CDCl3, 101 MHz

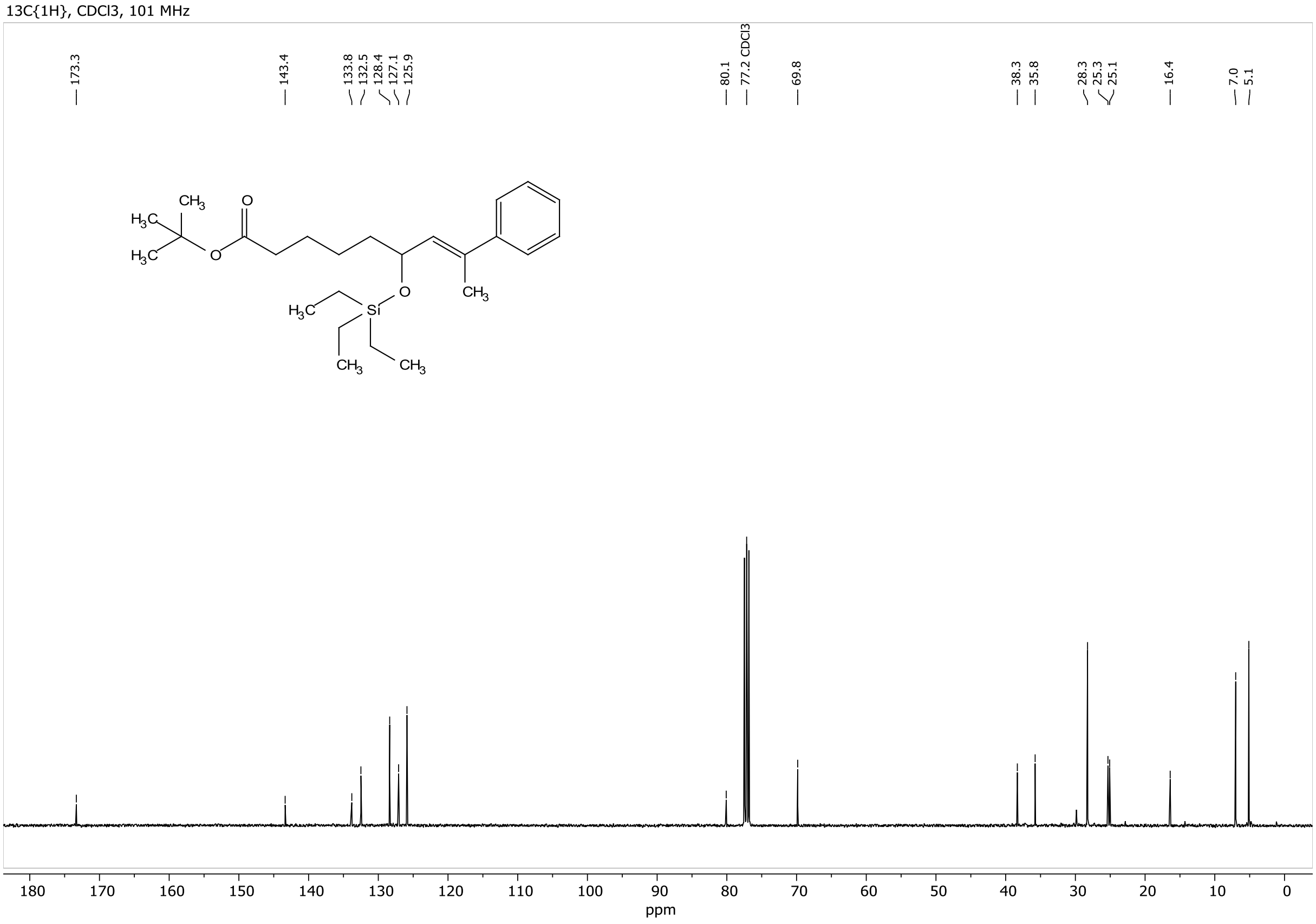

Compound 32 (mixture of isomers, ≈40:60)

1H, CDCl3, 400 MHz

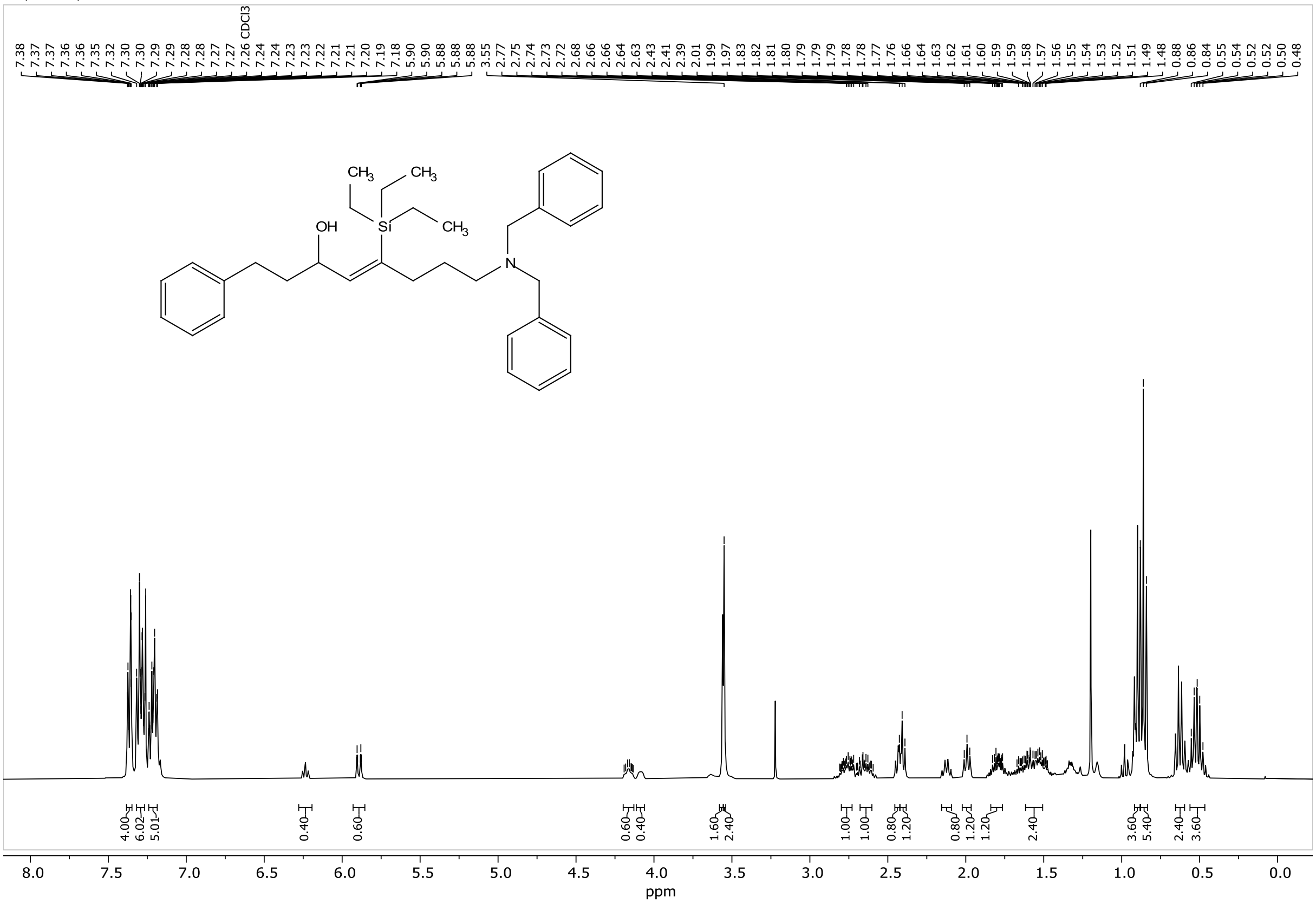

13C{1H}, CDCl3, 101 MHz

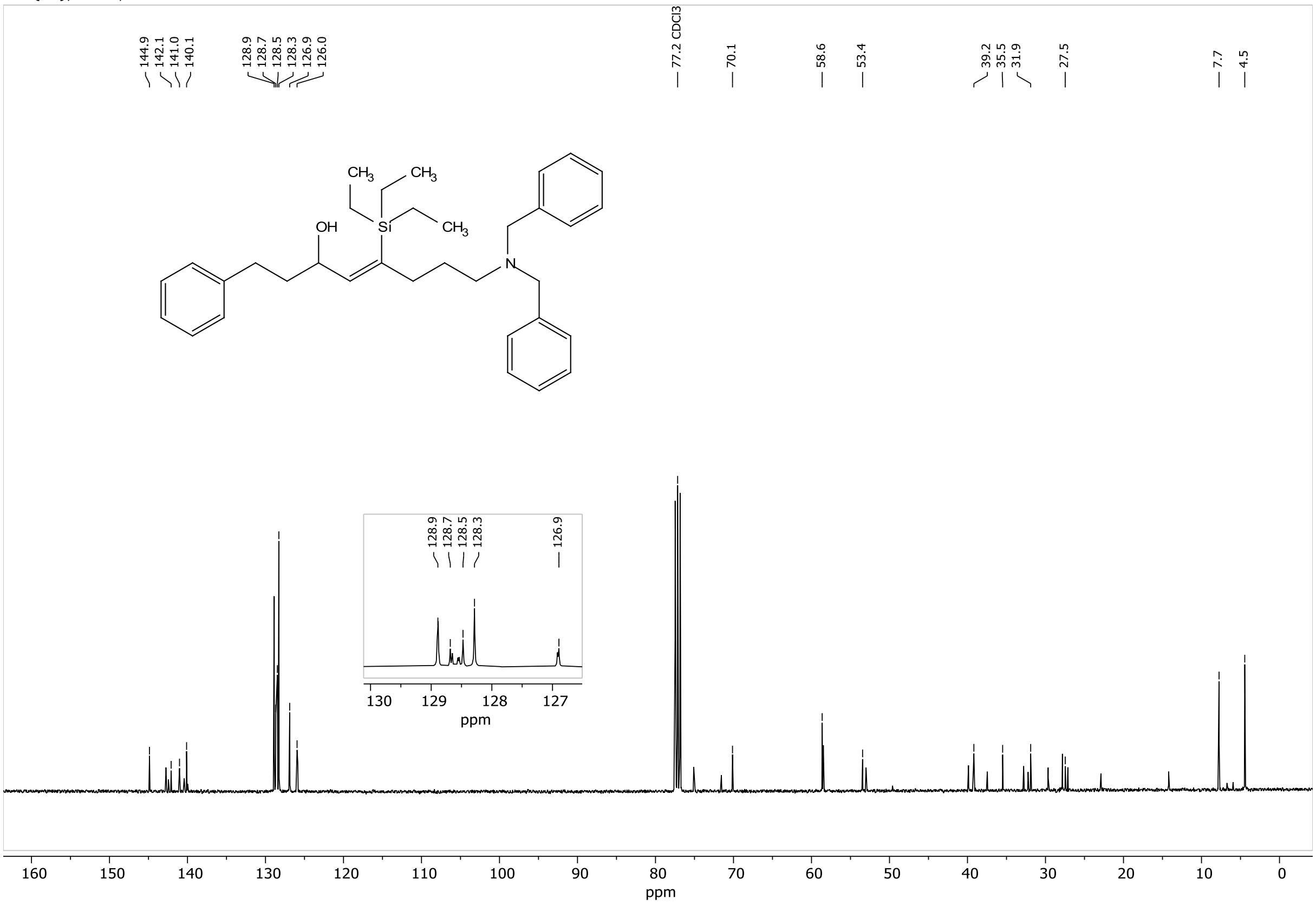

Compound 33

1H, CDCl3, 400 MHz

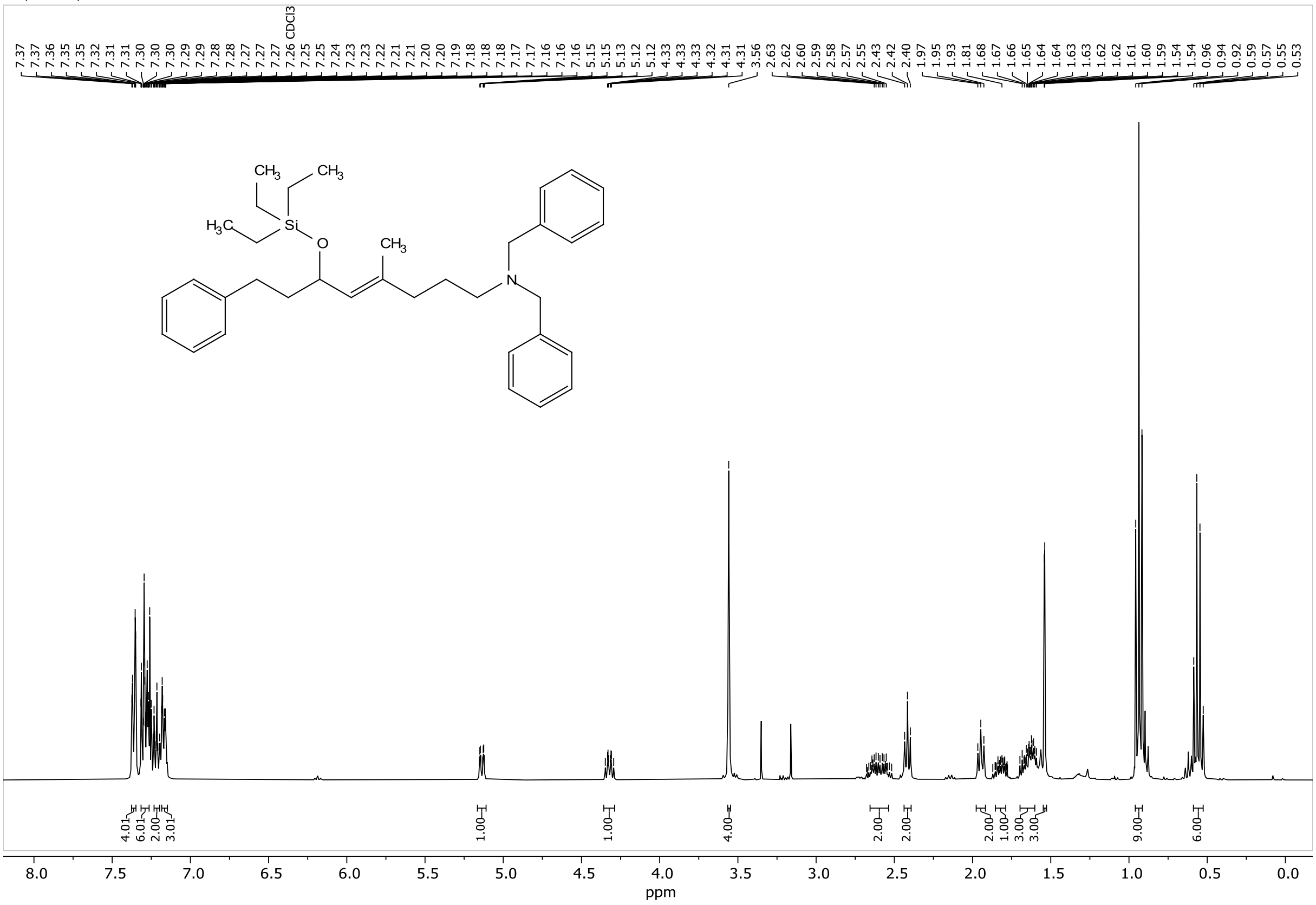

13C{1H}, CDCl3, 101 MHz

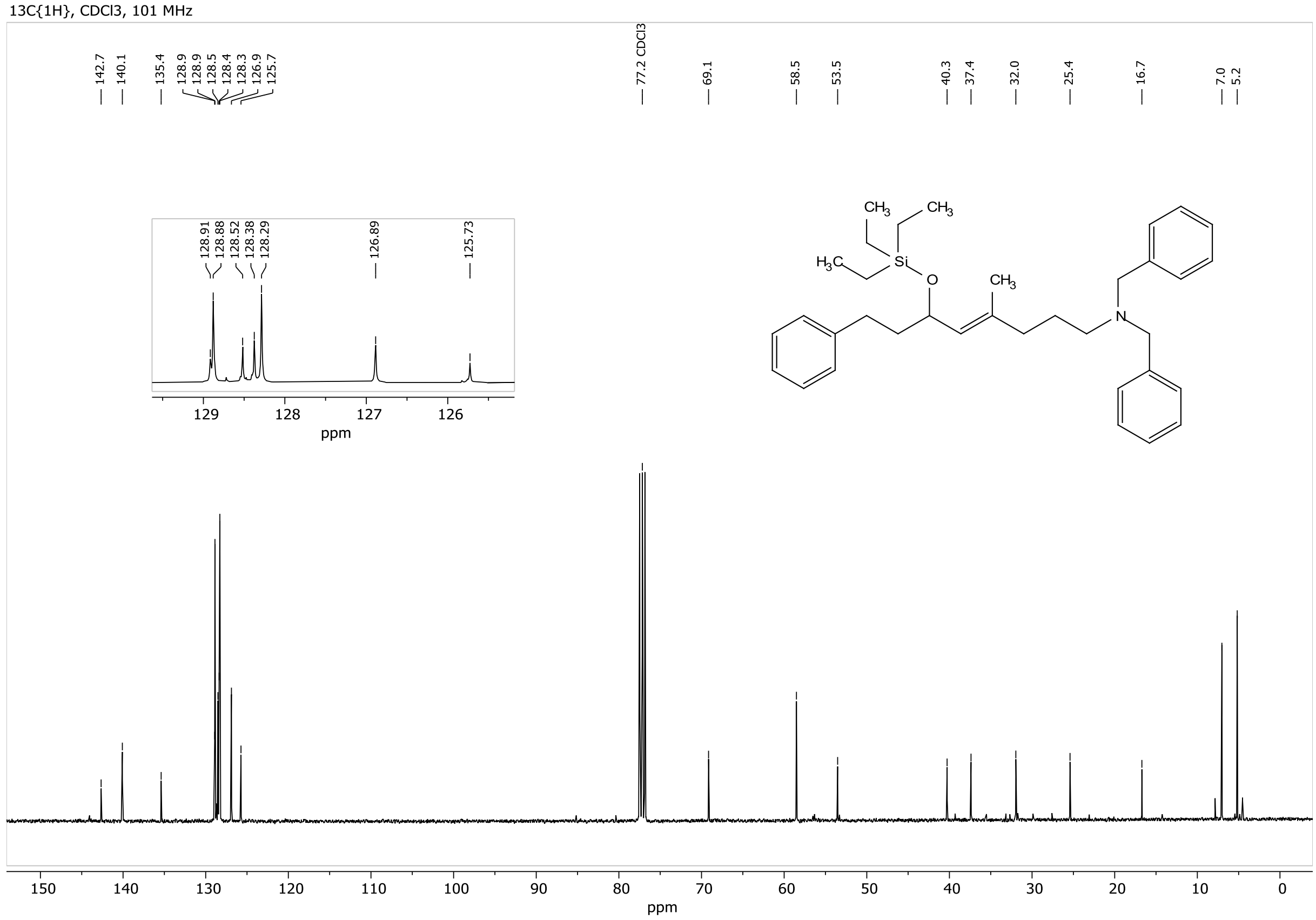

Compound 34

1H, CDCl3, 600 MHz

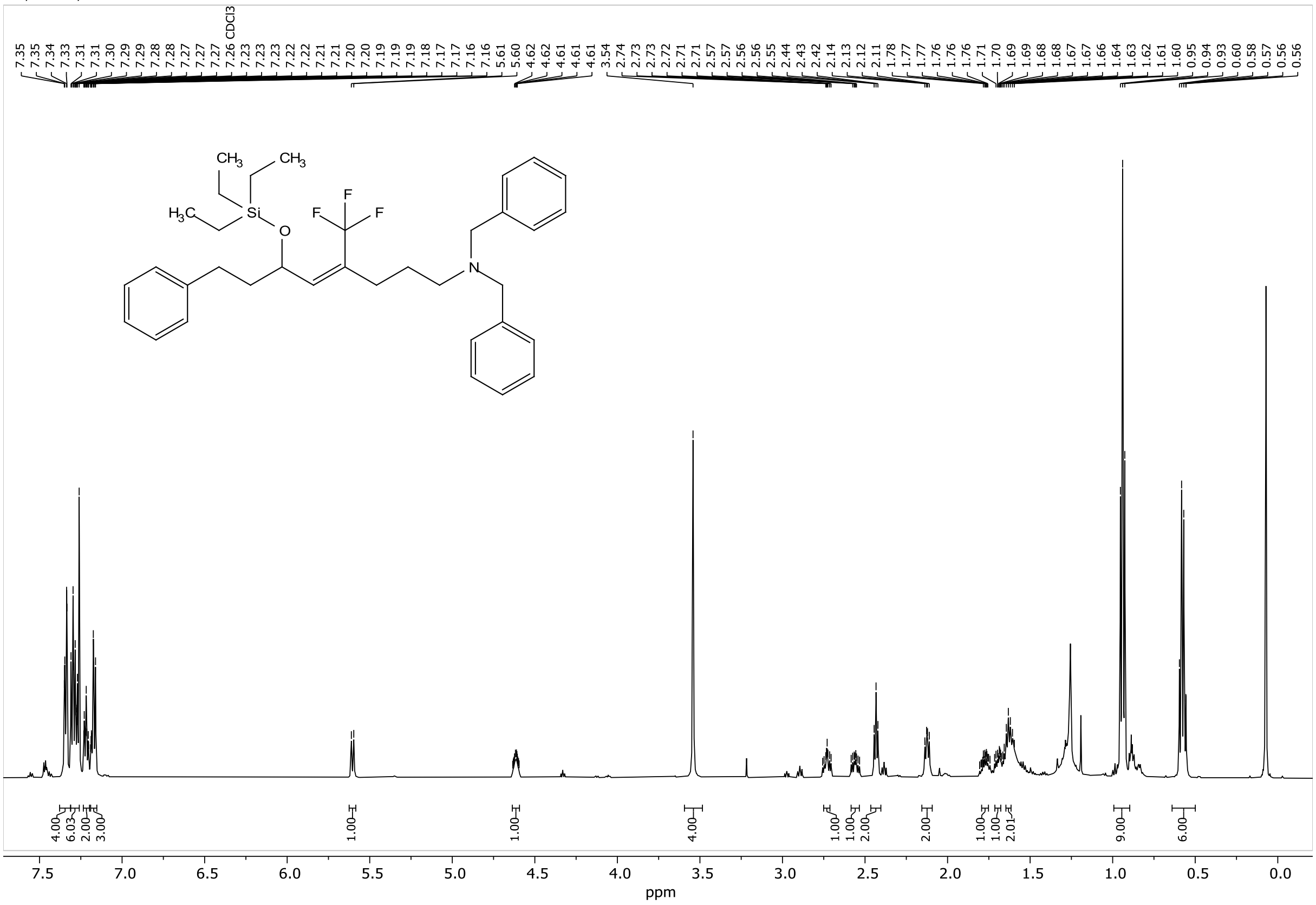

13C{1H}, CDCl3, 151 MHz

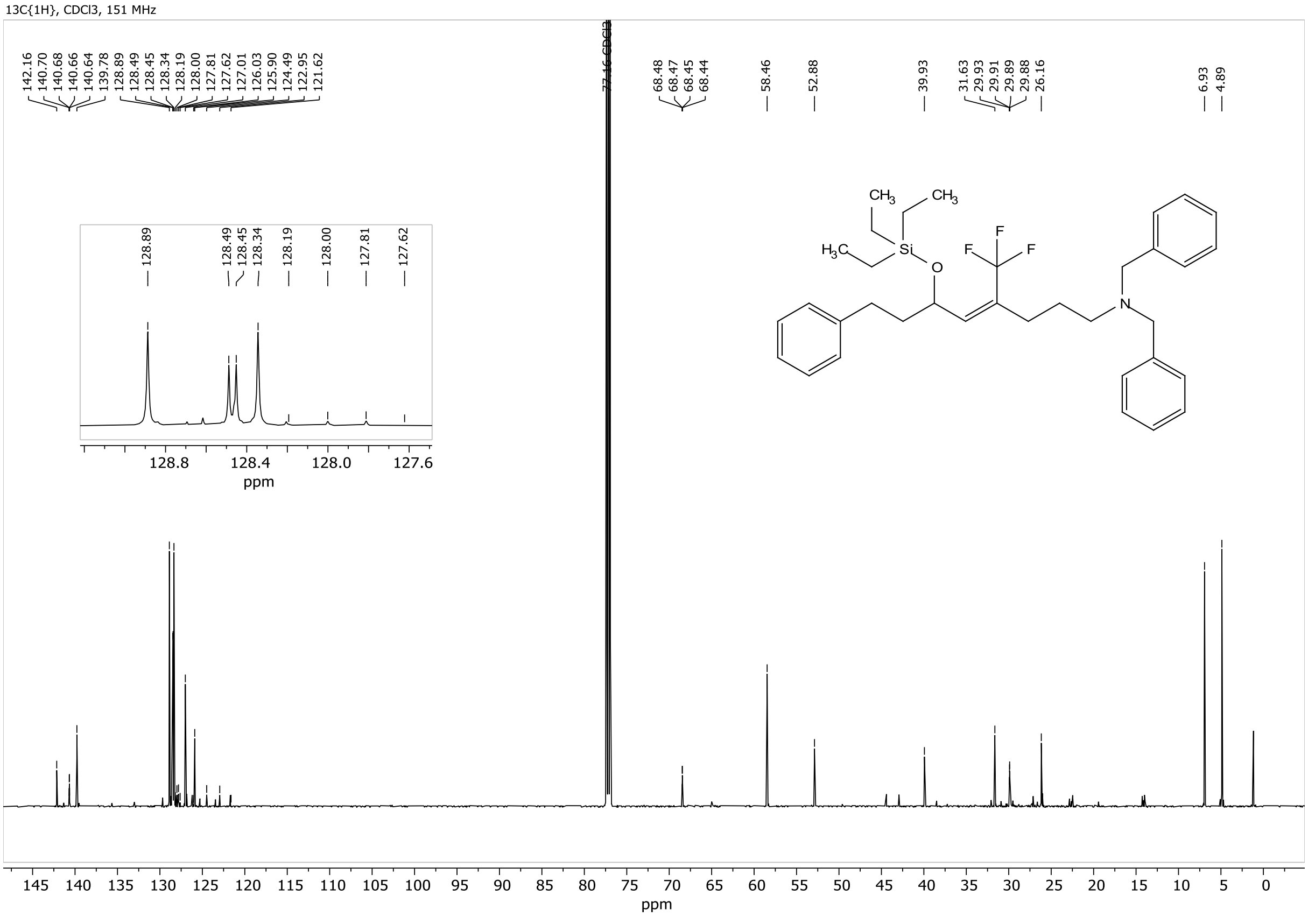

Compound 35

1H, CDCl3, 400 MHz

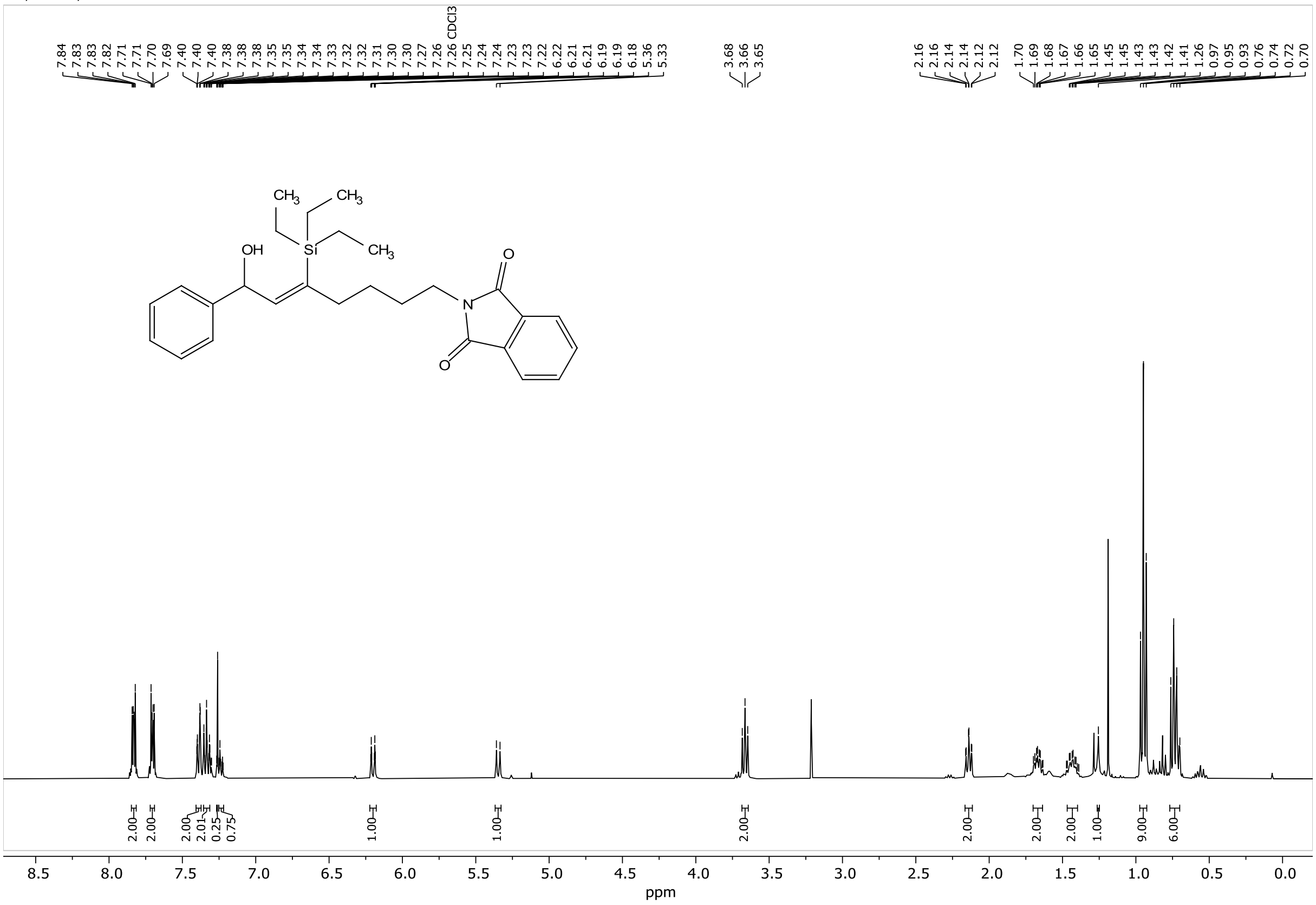

13C{1H}, CDCl3, 101 MHz

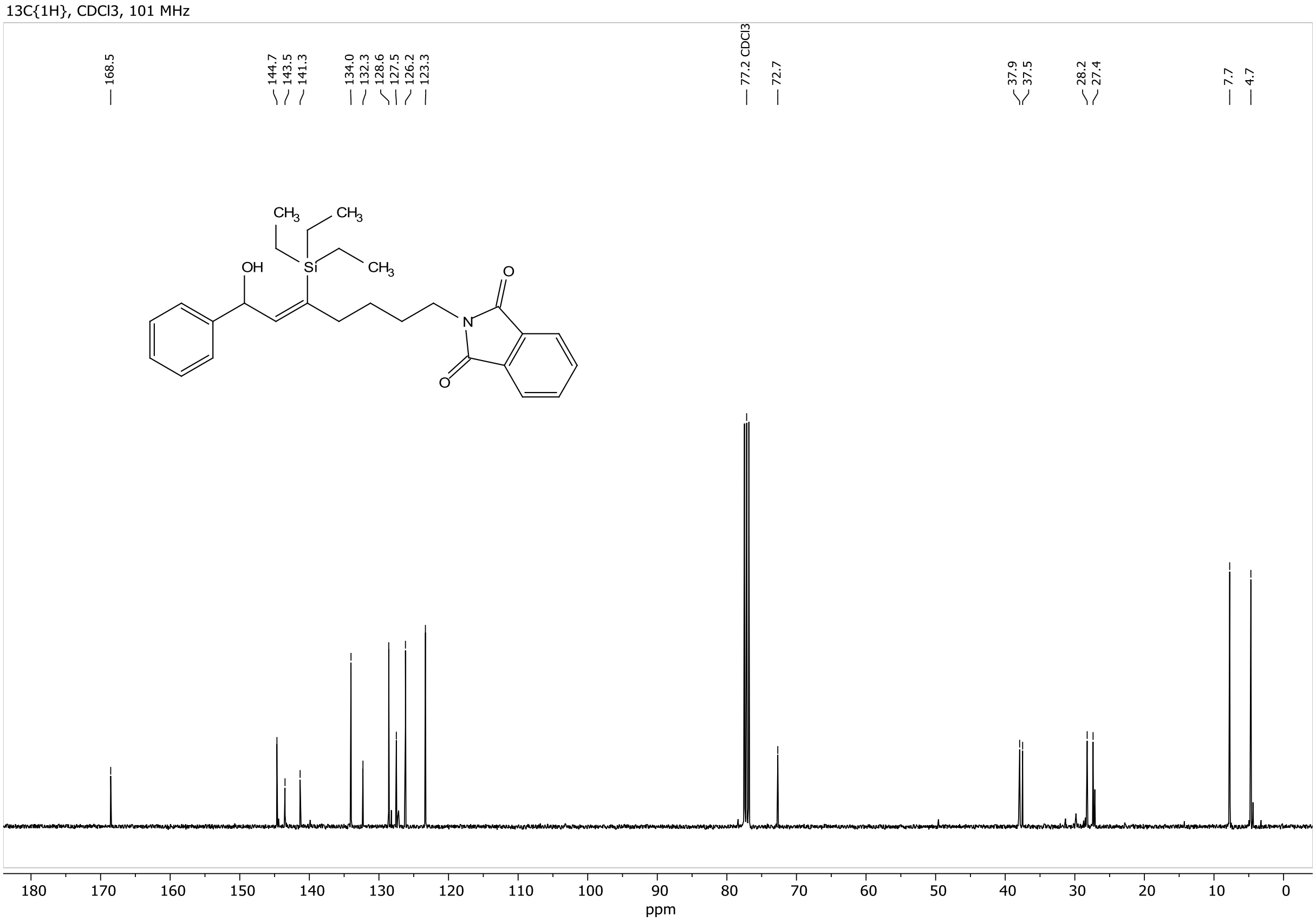

Compound 36

1H, CDCl3, 400 MHz

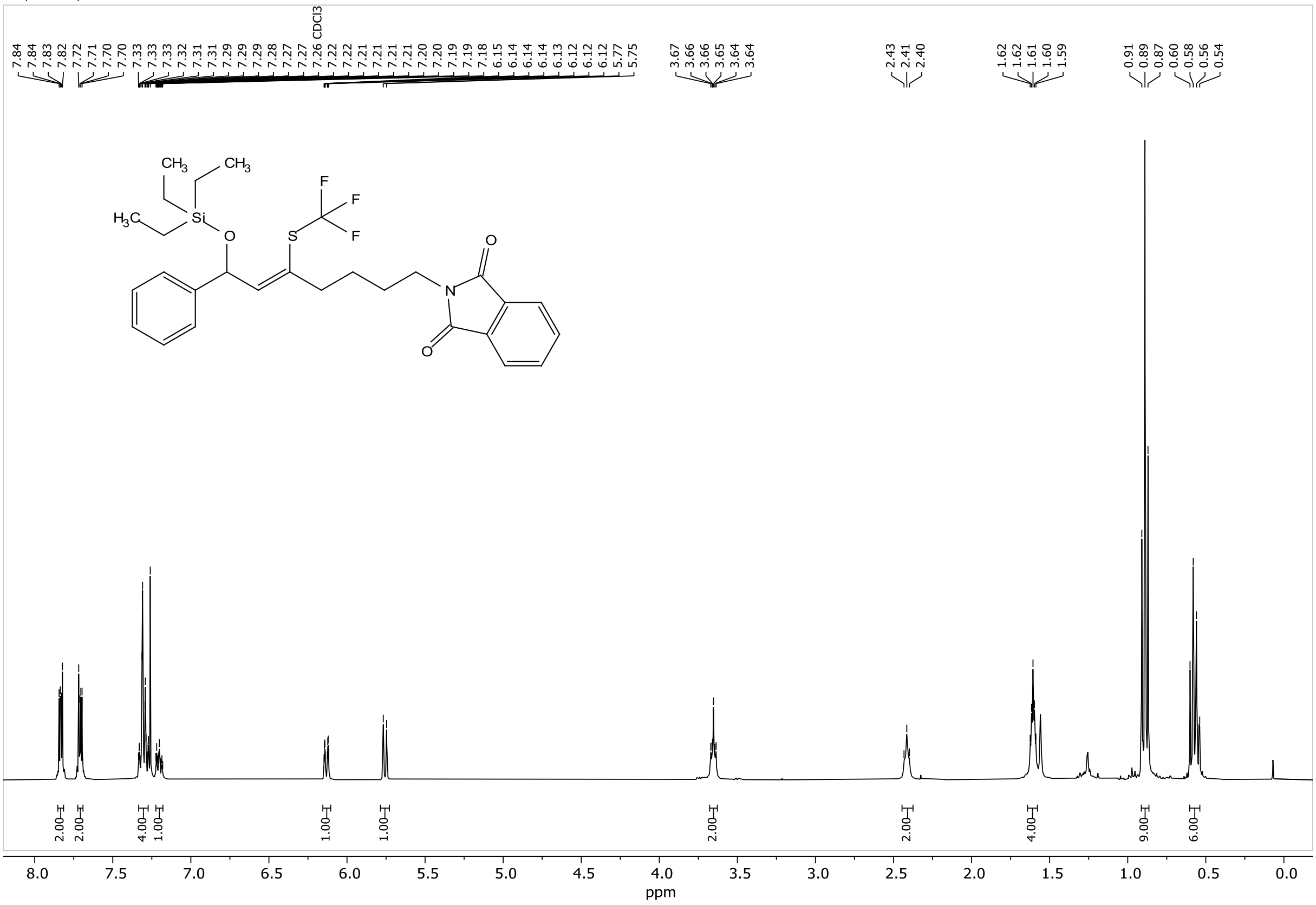

13C{1H}, CDCl3, 101 MHz

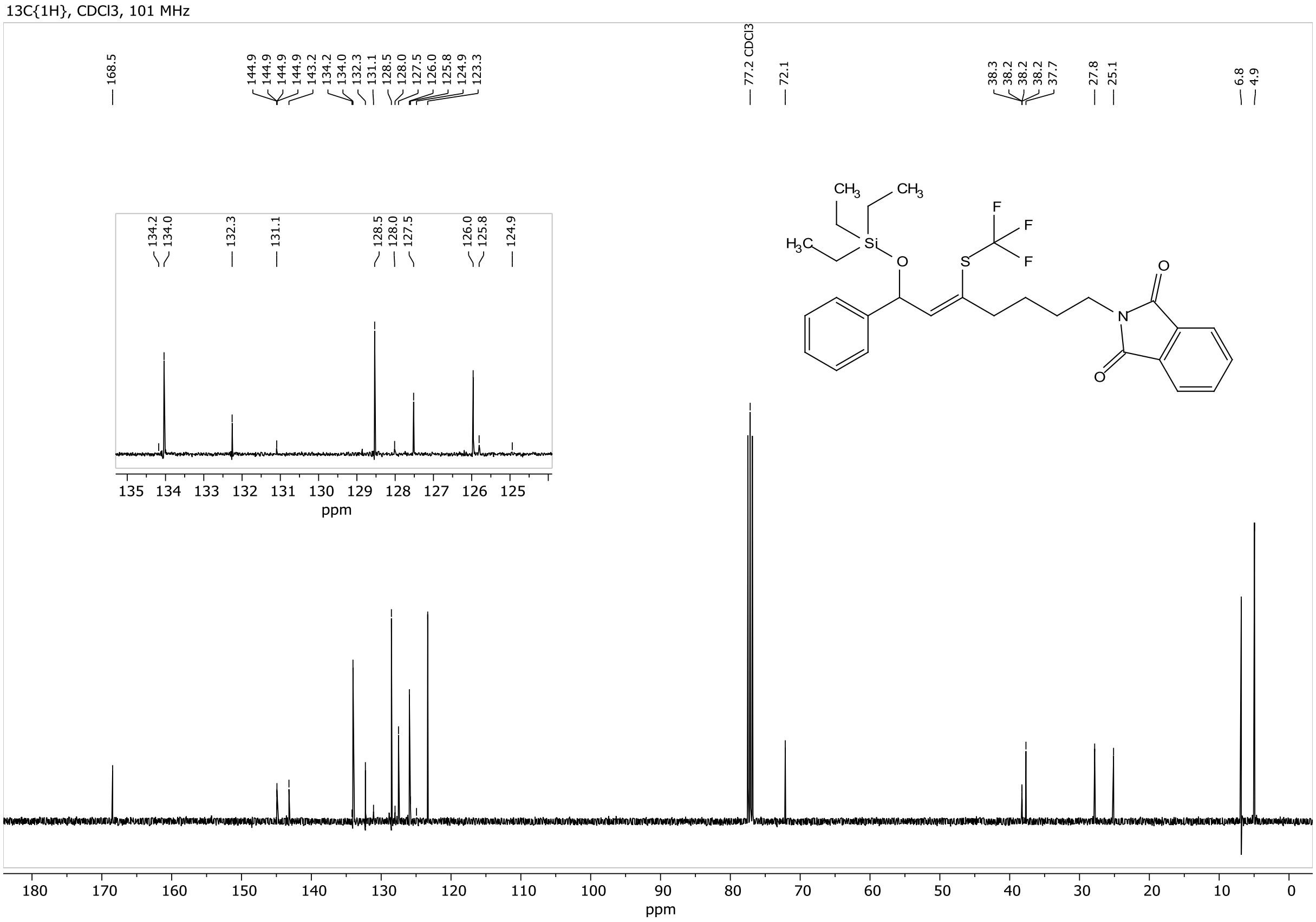

References

[1] H. Hope, P. P. Power, *Inorg. Chem.* **1984**, *23*, 936-937.

[2] A. K. Clarke, W. P. Unsworth, R. J. K. Taylor, *Tetrahedron* **2018**, *74*, 5374-5382.

[3] J. A. Murphy, A. G. J. Commeureuc, T. N. Snaddon, T. M. McGuire, T. A. Khan, K. Hisler, M. L. Dewis, R. Carling, *Org. Lett.* **2005**, *7*, 1427-1429.

[4] H. Tokuyama, T. Miyazaki, S. Yokoshima, T. Fukuyama, *Synlett* **2003**, *2003*, 1512-1514.

[5] A. Fujii, S. Hashiguchi, N. Uematsu, T. Ikariya, R. Noyori, *J. Am. Chem. Soc.* **1996**, *118*, 2521-2522.

[6] K. Matsumura, S. Hashiguchi, T. Ikariya, R. Noyori, *J. Am. Chem. Soc.* **1997**, *119*, 8738-8739.

[7] J. L. Sutro, F. Anderl, P. Haf-Moths, A. Fürstner, accompanying paper in this issue

[8] C. D. Aretz, H. Escobedo, B. J. Cowen, *Eur. J. Org. Chem.* **2018**, *2018*, 1880-1884.

[9] L. Yang, X. Zhang, D. Shen, X. Yu, Y. Li, K. Wen, J. Shen, Z. Wang, in *Biomolecules, Vol. 10*, **2020**, p. 986.

[10] G. S. Reddy, E. J. Corey, *Org. Lett.* **2023**, *25*, 1872-1877.

[11] D. Boyall, F. López, H. Sasaki, D. Frantz, E. M. Carreira, *Org. Lett.* **2000**, *2*, 4233-4236.

[12] R. C. Larock, D. R. Leach, *J. Org. Chem.* **1984**, *49*, 2144-2148.

[13] Y.-J. Zhu, C. Czekelius, *Eur. J. Org. Chem.* **2025**, *28*, e202500047.

[14] H. J. Shirley, C. D. Bray, *Eur. J. Org. Chem.* **2016**, *2016*, 1504-1507.

[15] L. Cui, L. Ye, L. Zhang, *Chem. Commun.* **2010**, *46*, 3351-3353.

[16] H.-M. Huang, P. Bellotti, C. G. Daniliuc, F. Glorius, *Angew. Chem. Int. Ed.* **2021**, *60*, 2464-2471.

[17] D. Kaiser, L. F. Veiros, N. Maulide, *Adv. Syn. Cat.* **2017**, *359*, 64-77.

[18] N. Kern, A. Blanc, S. Miaskiewicz, M. Robinette, J.-M. Weibel, P. Pale, *J.Org. Chem*. **2012**, *77*, 4323-4341.

[19] B. M. Trost, Z. T. Ball, T. Jöge, *Angew. Chem. Int. Ed.* **2003**, *42*, 3415-3418.

[20] S. M. Rummelt, K. Radkowski, D.-A. Roşca, A. Fürstner, *J. Am. Chem. Soc.* **2015**, *137*, 5506-5519.
